# Supplementary material for: N-heterocyclic carbene-catalyzed atroposelective synthesis of N-Aryl phthalimides and maleimides via activation of carboxylic acids
Source: Nat Commun. 2024 Jul 9;15:5755. doi: 10.1038/s41467-024-49799-5 (PMC11233592; doi:10.1038/s41467-024-49799-5)
Supplement: Supplementary file 1 — Supplementary Information [file 41467_2024_49799_MOESM1_ESM.pdf]

N-Heterocyclic Carbene-Catalyzed Atroposelective Synthesis of *N*-Aryl Phthalimides and Maleimides via Activation of Carboxylic Acids

Soumen Barik, Sowmya Shree Ranganathappa, Akkattu T. Biju*

Department of Organic Chemistry, Indian Institute of Science, Bangalore-560012

E-mail: atbiju@iisc.ac.in

Supplementary Information

1. Supplementary Methods S2
   1.1 General Information S2
   1.2 General Procedure for the Synthesis of Phthalamic/Maleamic Acid Derivatives S3
   1.3 General Procedure for the Optimization of Reaction Conditions S3
   1.4 General Procedure for the Atroposelective Synthesis of C-N Axially Chiral *N*-Aryl Phthalimides/Maleimides S5
2. Supplementary Discussion S7
   2.1 Mechanistic Experiments S7
   2.2 Studies on Rotation Barrier of C-N Axially Chiral Phthalimides S11
   2.3 X-Ray Data S26
   2.4 Synthesis and Characterization of Phthalamic/Maleamic Acid Derivatives S32
   2.5 Synthesis and Characterization of *N*-Aryl Phthalimides/Maleimides S51
   2.6 Product Functionalization S74
3. Supplementary Figures S81
   3.1 ^{1}H and ^{13}C NMR Spectra of Phthalamic/Maleamic Acid Derivatives S81
   3.2 ^{1}H and ^{13}C NMR Spectra of *N*-Aryl Phthalimides/Maleimides S118
   3.3 HPLC-Chromatogram of *N*-Aryl Phthalimides/Maleimides S161
4. Supplementary References S206

1. Supplementary Methods

1.1 General Information

Unless otherwise specified, all reactions were carried out under an atmosphere of argon in flame-dried reaction vessels with Teflon screw caps. THF was freshly distilled over Na-benzophenone and was transferred under agon. Dry CH_2Cl_2 was purchased from commercial sources and stored under argon atmosphere. The triazolium salt **3** was synthesized following the literature procedure.[1] K_2CO_3 was dried by heating at 120 °C under vacuum and cooling under argon atmosphere.

Analytical thin layer chromatography was performed on TLC Silica gel 60 F254. Visualization was accomplished with short wave UV light or KMnO4 staining solutions followed by heating. Flash chromatography was performed on silica gel (230-400 mesh) by standard techniques eluting with Pet. Ether-EtOAc solvent system.

All compounds were fully characterized. ^{1}H and ^{13}C NMR spectra were recorded on Bruker AV 400 and Bruker Ultrashield spectrometer in $CDCl_3$ and DMSO-d_6 as solvent. Chemical shifts (δ) are given in ppm (broad singlets are represented as bs). The residual solvent signals were used as references and the chemical shifts converted to the TMS scale ($CDCl_3$: δH = 7.26 ppm, δC = 77.16 ppm; DMSO-D_6: δH = 2.50 ppm, δC = 39.52 ppm). Infrared (FT-IR) spectra were recorded on a Bruker alpha FT-IR spectrophotometer, ν-max in cm^{-1}. HRMS (ESI) data were recorded on a Waters Xevo G2-XS Q-TOF instrument. Optical rotations were measured on JASCO P-2000 polarimeter at 25 °C using 50 mm cell of 1.0 mL capacity. HPLC analysis was performed on Agilent Technologies 1260 Infinity with a Variable Wavelength Detector.

1.2 General Procedure for the Synthesis of Phthalamic/Maleamic Acid Derivatives

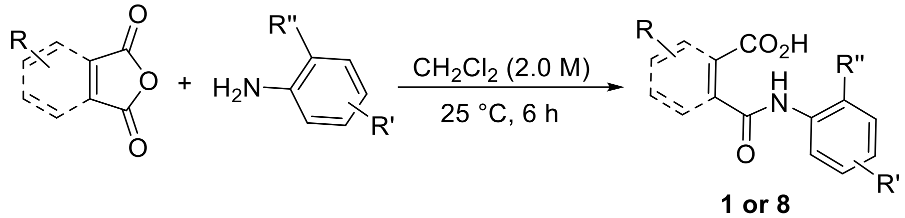

The carboxylic acid derivatives used in the present study were prepared by a modified literature procedure.[2] Phthalic anhydride (1.0 mmol, 1.0 equiv) was dissolved in CH_2Cl_2 (0.5 mL, 2.0 M). Then equimolecular amount of 2-substituted aniline derivative (1.0 mmol, 1.0 equiv) was added and reaction mixture was stirred for 6 h at 25 °C. The solvent was removed under reduced pressure, and then the residue was purified by flash chromatography (silica gel; elusion typically using 50% EtOAc-Pet. Ether) to form the desired carboxylic acid derivatives **1** or **8** in moderate to good yields.

1.3 General Procedure for the Optimization of Reaction Conditions

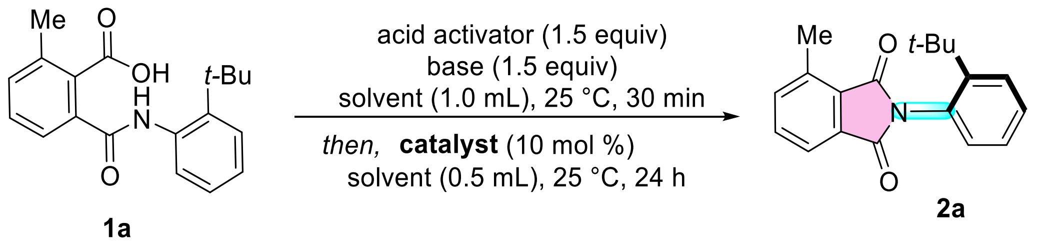

In a flame-dried screw-capped test tube equipped with a magnetic stir bar was taken K_2CO_3 (26.0 mg, 0.188 mmol, 1.5 equiv) from the glovebox, then 2-((2-(*tert*-butyl)phenyl)carbamoyl)-6-methylbenzoic acid **1a** (39.0 mg, 0.125 mmol, 1.0 equiv) was added. Then, the screw-capped tube was evacuated and backfilled with argon. To this mixture was added THF (1.0 mL) under argon atmosphere followed by PivCl (23 μL, 0.188 mmol, 1.5 equiv). The resultant reaction mixture was kept stirring at 25 °C until the full conversion of acid to the corresponding anhydride (monitored by TLC; typically, 30 minutes). To this mixture, the triazolium salt **3** (4.6 mg, 0.0125 mmol, 10 mol %) and THF (0.5 mL) were successively added and stirred for 24 h. Then the reaction was quenched by adding CH_2Cl_2 (2.0 mL) and filtered through a short pad of silica gel and eluted with EtOAc (2x10 mL), the solvent was evaporated to get the crude residue, which was analyzed using ^{1}H NMR using CH_2Br_2 (9 μL, 0.125 mmol) as the internal standard. The yield of **2a** was determined by ^{1}H NMR

analysis of the crude reaction mixture. The enantiomeric excess was determined by HPLC analysis on a chiral stationary phase.

Supplementary Table 1: Optimization of Reaction Conditions

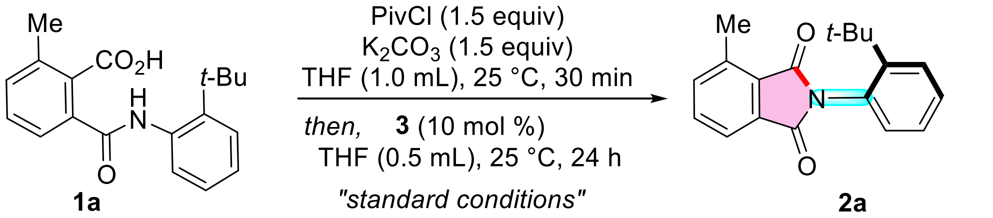

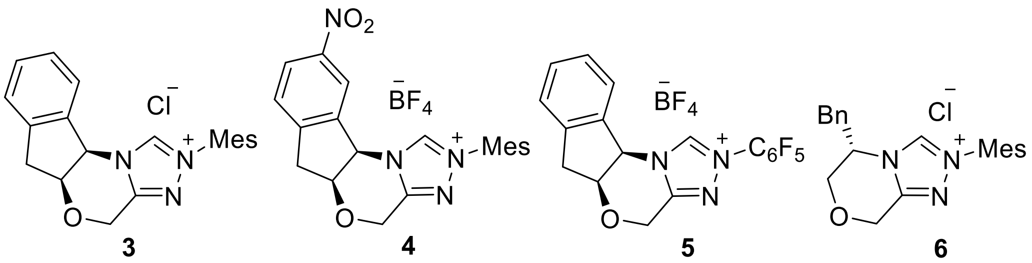

entry	variation on the standard condition[a]	yield (%)[b]	er[c]
1	***none***	***99 (99)[d]***	***98:2***
2	without **3**	<5	ND
3	without PivCl	<5	ND
4	**4** instead of **3**	99	97:3
5	**5** instead of **3**	<5	ND
6	**6** instead of **3**	96	5:95
7	HATU instead of PivCl	92	87:13
8	Cs_2CO_3 instead of K_2CO_3	99	89:11
9	Na_2CO_3 instead of K_2CO_3	90	92:8
10	DMAP instead of K_2CO_3	75	81:19
11	DABCO instead of K_2CO_3	99	95:5
12	DBU instead of K_2CO_3	90	87:13
13	Et_3N instead of K_2CO_3	52	93:7
14	*i*-Pr_2NH instead of K_2CO_3	9	52:48
15	DIPEA instead of K_2CO_3	20	79:21
16	KO*t*-Bu instead of K_2CO_3	<5	ND
17	MTBE instead of THF	91	93:7
18	DME instead of THF	92	90:10

19	Et_2O instead of THF	96	91:9
20	1,4-dioxane instead of THF	<5	-
21	$CHCl_3$ instead of THF	80	77:23
22	toluene instead of THF	70	96:4
23	CH_3CN instead of THF	96	58:42
24	DMF instead of THF	35	61:39
25	mesitylene instead of THF	76	81:19
26	chlorobenzene instead of THF	81	82:18
27	*tert*-butylbenzene instead of THF	87	83:17
28	DCM instead of THF	92	85:15
29	DCE instead of THF	34	68:32
30	EtOAc instead of THF	95	72:28
31	12 h instead of 24 h	82	98:2
32	0 °C instead of 25 °C	93	98:2

[a] Standard conditions: **1a** (0.125 mmol), **3** (10 mol %), K_2CO_3 (1.5 equiv.), THF (1.5 mL), 25 °C and 24 h. [b] Determined by ^{1}H NMR analysis of crude products using CH_2Br_2 as the internal standard. [c] The er value was determined by HPLC analysis on a chiral stationary phase. [d] Isolated yield.

1.4 General Procedure for the Atroposelective Synthesis of *N*-Aryl Phthalimides/Maleimides

Procedure for the Atroposelective Synthesis of N-Aryl Phthalimides

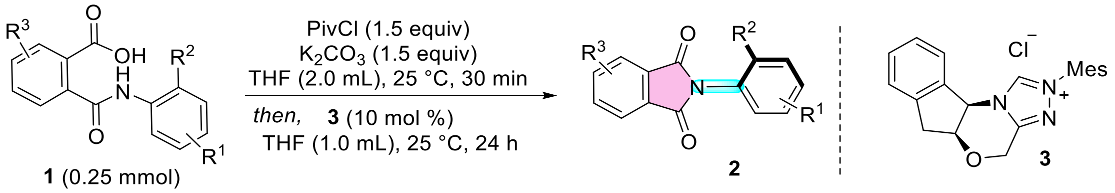

In a flame-dried screw-capped test tube equipped with a magnetic stir bar was taken K_2CO_3 (52 mg, 0.375 mmol, 1.5 equiv) from the glovebox, then phthalamic acid derivative **1** (0.25 mmol, 1.0 equiv) was added. Then, the screw-capped tube was evacuated and backfilled with argon. To this mixture was added THF (2.0 mL) under argon atmosphere followed by PivCl (43 μL, 0.375 mmol, 1.5 equiv). The resultant reaction mixture was kept stirring at 25 °C until the full conversion of acid to the corresponding anhydride (monitored by TLC; typically, 30 minutes). To this mixture, the triazolium salt **3** (9.2 mg, 0.025 mmol, 10 mol %) and THF (1.0 mL) were successively added and stirred for 24 h. Then the solvent was

evaporated to get the crude residue, which was purified by flash column chromatography on silica gel to afford the corresponding C-N axially chiral phthalimides.

Procedure for the 1.0 mmol Scale Synthesis of 2a

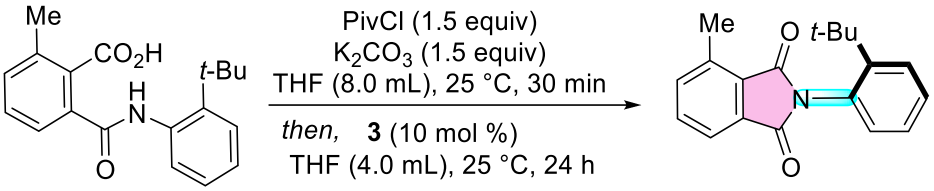

1a (1.0 mmol) 99% yield, 98:2 er **2a**

In a flame-dried screw-capped test tube equipped with a magnetic stir bar was taken K_2CO_3 (208 mg, 1.5 mmol, 1.5 equiv) from the glovebox, then 2-((2-(*tert*-butyl)phenyl)carbamoyl)-6-methylbenzoic acid **1a** (311.4 mg, 1.0 mmol, 1.0 equiv) was added. Then, the screw-capped tube was evacuated and backfilled with argon. To this mixture was added THF (8.0 mL) under argon atmosphere followed by PivCl (172 µL, 1.0 mmol, 1.5 equiv). The resultant reaction mixture was kept stirring at 25 °C until the full conversion of acid to the corresponding anhydride (monitored by TLC; typically, 30 minutes). To this mixture, the triazolium salt **3** (36 mg, 0.1 mmol, 10 mol %) and THF (4.0 mL) were successively added and stirred for 24 h. Then the solvent was evaporated to get the crude residue, which was purified by flash column chromatography on silica gel (elusion typically using 10% EtOAc-Pet. Ether) to afford the C-N axially chiral phthalimide **2a** in 99% yield and 98:2 er.

Procedure for the Low Catalyst Loading

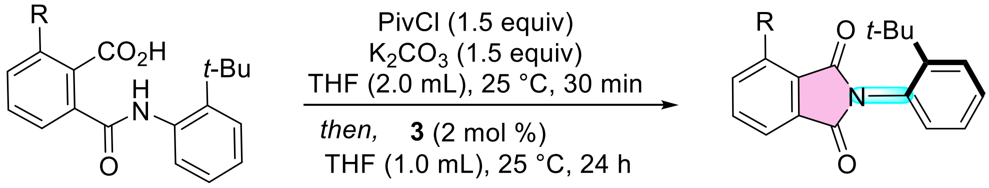

1 (0.25 mmol) **2a** or **2w**

R = Me, **2a**, 90% yield, 95:5 er
R = Cl, **2w**, 92% yiled, 96:4 er

In a flame-dried screw-capped test tube equipped with a magnetic stir bar was taken K_2CO_3 (52 mg, 0.375 mmol, 1.5 equiv) from the glovebox, then 2-((2-(*tert*-butyl)phenyl) carbamoyl)-6-methylbenzoic acid **1a** (78.0 mg, 0.25 mmol, 1.0 equiv) or 2-((2-(*tert*-butyl)phenyl)carbamoyl)-6-chlorobenzoic acid **1w** (82.9 mg, 0.25 mmol, 1.0 equiv) was added. Then, the screw-capped tube was evacuated and backfilled with argon. To this mixture was

added THF (2.0 mL) under argon atmosphere followed by PivCl (43 µL, 0.375 mmol, 1.5 equiv). The resultant reaction mixture was kept stirring at 25 °C until the full conversion of acid to the corresponding anhydride (monitored by TLC; typically, 30 minutes). To this mixture, the triazolium salt **3** (1.84 mg, 0.005 mmol, 2.0 mol %) and THF (1.0 mL) were successively added and stirred for 24 h. Then the solvent was evaporated to get the crude residue, which was purified by flash column chromatography on silica gel (elusion typically using 10% EtOAc-Pet. Ether) to afford the C-N axially chiral phthalimide **2a** in 90% yield and 95:5 er or **2w** in 92% yield and 96:4 er.

2. Supplementary Discussion

2.1 Mechanistic Experiments

(a) Isolation of Isoimide Intermediate 7a

(*E*)-3-((2-(*tert*-Butyl)phenyl)imino)-7-methylisobenzofuran-1(3*H*)-one (7a)

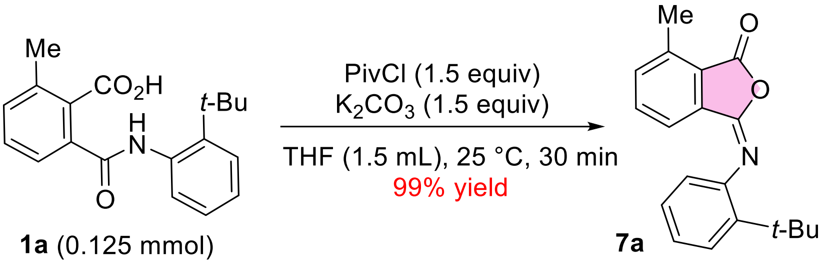

In a flame-dried screw-capped test tube equipped with a magnetic stir bar was taken K_2CO_3 (26.0 mg, 0.188 mmol, 1.5 equiv) from the glovebox, then 2-((2-(*tert*-butyl)phenyl)carbamoyl)-6- methylbenzoic acid **1a** (39.0 mg, 0.125 mmol, 1.0 equiv) followed by THF was added under argon atmosphere. To the reaction mixture, PivCl (23 µL, 0.188 mmol, 1.5 equiv) was added. Then the resultant reaction mixture was kept stirring at 25 °C for 30 minutes. The reaction mixture was diluted by adding 2.0 mL of EtOAc and filtered through a short pad of celite by eluting with EtOAc (2x5 mL). The solvent was evaporated under reduced pressure and kept under high vacuum (to remove trace solvents) for 1h to afford (*E*)-3-((2-(*tert*-butyl)phenyl)imino)-7-methyl isobenzofuran-1(3*H*)-one **7a** as pale yellow solid (36.2 mg, 99% yield).

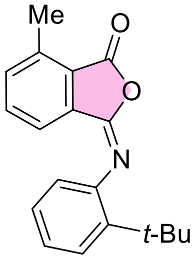

7a

R

R_f (Pet. Ether /EtOAc = 90/10): 0.72. **^{1}H NMR (400 MHz, CDCl$_3$)** δ 7.92 (d, *J* = 7.5 Hz, 1H), 7.72 (t, *J* = 7.5 Hz, 1H), 7.52 (d, *J* = 7.5 Hz, 1H), 7.43 (d, *J* = 7.8 Hz, 1H), 7.25 – 7.21 (m, 1H), 7.17-7.14 (m, 2H), 2.74 (s, 3H), 1.44 (s, 9H). **^{13}C NMR (100 MHz, CDCl$_3$)** δ 165.5, 145.9, 143.1, 139.9,

137.4, 135.3, 135.0, 126.5, 126.4, 125.8, 125.6, 123.7, 121.2, 35.5, 30.2, 17.7. **HRMS (ESI)** calculated $[M+H]^+$ for $C_{19}H_{20}NO_2$: 294.1489, found: 294.1492. **FTIR (cm^{-1})** 2960, 2919, 2870, 1768, 1701, 1599, 1563, 1481, 1213, 919, 762.

Supplementary Figure 1:

^{1}H NMR of (*E*)-3-((2-(*tert*-Butyl)phenyl)imino)-7-methylisobenzofuran-1(3*H*)-one (7a)

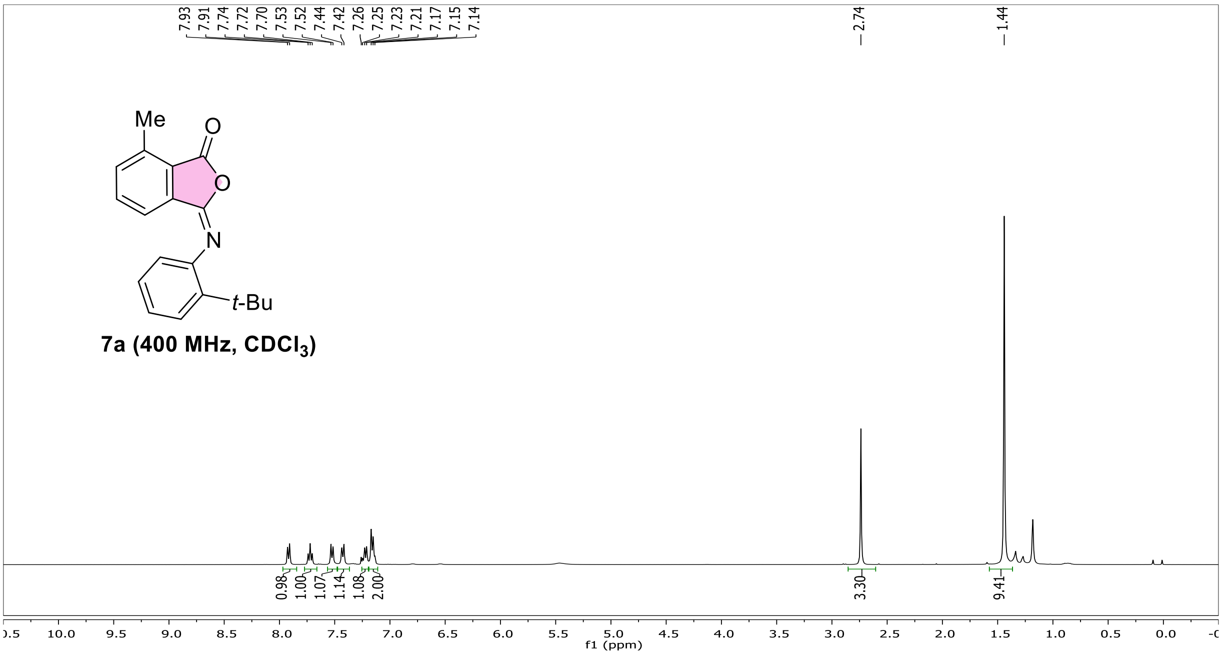

^{13}C NMR of (*E*)-3-((2-(*tert*-Butyl)phenyl)imino)-7-methylisobenzofuran-1(3*H*)-one (7a)

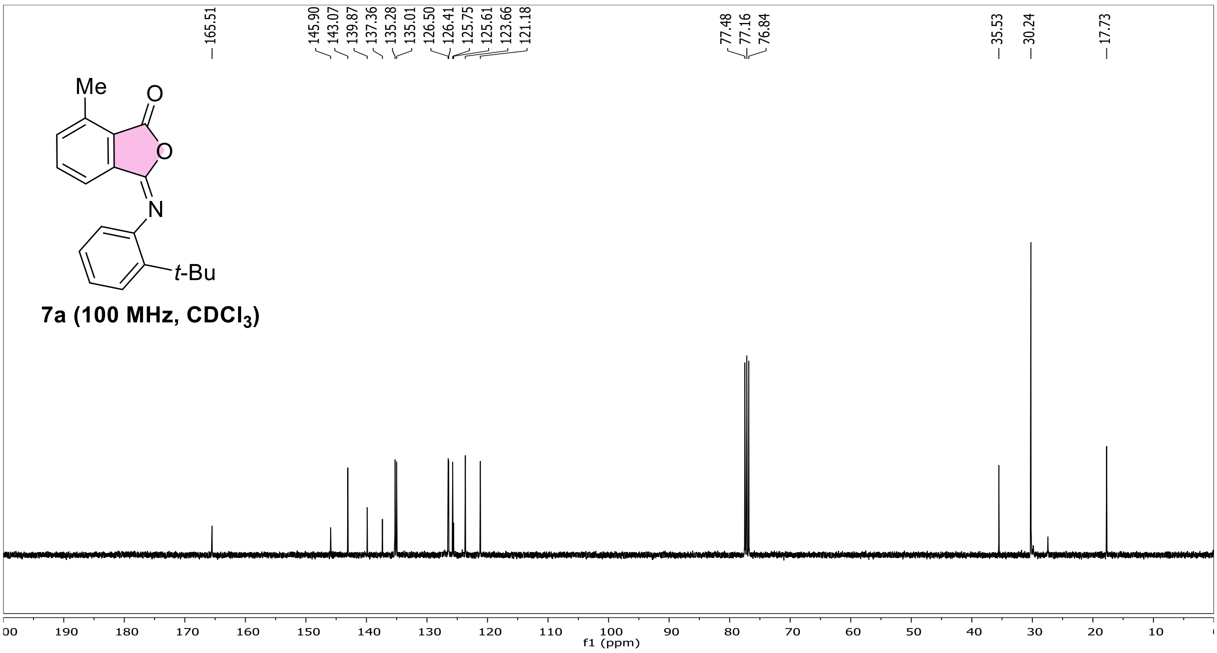

*The formation of isoimide **7a** under NHC-free condition is likely an indication that isoimide **7a** is probably an intermediate in the present reaction. NHC likely adds to the isoimide to form the desired product.*

(b) Reaction with Isoimide Intermediate 7a with NHC

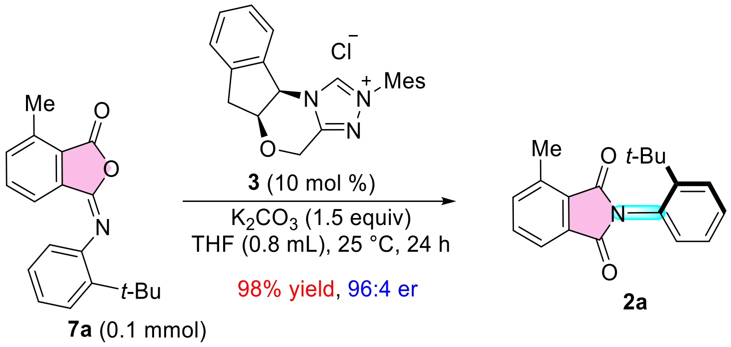

In a flame-dried screw-capped test tube equipped with a magnetic stir bar was taken K_2CO_3 (21.0 mg, 0.15 mmol, 1.5 equiv) from the glovebox, then (*E*)-3-((2-(*tert*-butyl)phenyl)imino)-7-methyl isobenzofuran-1(3*H*)-one **7a** (29.3 mg, 0.1 mmol, 1.0 equiv) followed by THF (0.8 mL) was added under argon atmosphere. To the reaction mixture azolium salt **3** (3.7 mg, 0.01 mmol, 0.1 equiv) was added and the resulting reaction mixture was stirred for 24 h. Then, the solvent was evaporated to get the crude residue, which was purified by flash column chromatography on silica gel to afford the 2-(2-(*tert*-butyl)phenyl)-4-methylisoindoline-1,3-dione **2a** (28.8 mg, 98% yield with 96:4 er).

*The formation of **2a** from isoimide **7a** under NHC-catalyzed conditions indicate that the isoimide **7a** is an intermediate in the present reaction.*

(c) Reaction without Base

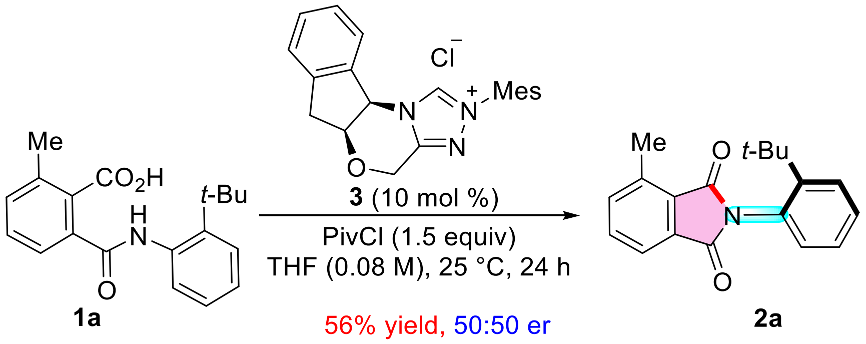

In a flame-dried screw-capped test tube equipped with a magnetic stir bar was taken 2-((2-(*tert*-butyl)phenyl)carbamoyl)-6- methylbenzoic acid **1a** (31.1 mg, 0.1 mmol, 1.0 equiv) followed by THF was added under argon atmosphere. To the reaction mixture, PivCl (18 μL, 0.15 mmol, 1.5 equiv) was added. Then the resultant reaction mixture was kept stirring at 25 °C for 60 minutes. To the reaction mixture azolium salt **3** (3.7 mg, 0.01 mmol, 0.1 equiv) was added and the resulting reaction mixture was stirred for 24 h. Then the solvent was evaporated

to get the crude residue, which was purified by flash column chromatography on silica gel to afford the 2-(2-(*tert*-butyl)phenyl)-4-methylisoindoline-1,3-dione **2a** (16.2 mg, 56% yield with 50:50 er).

The involvement of the chiral azolium salt via H-bonding interaction may be ruled out from this experiment. Moreover, as the reaction is performed in the absence of base, the free carbene may not have generated. The background reaction may be mediated by the in situ generated HCl during the pivoloyl anhydride formation, which leads to the 56% yield of product ***2a*** *with 50:50 er.*

(d) Reaction without NHC Precursor and Base

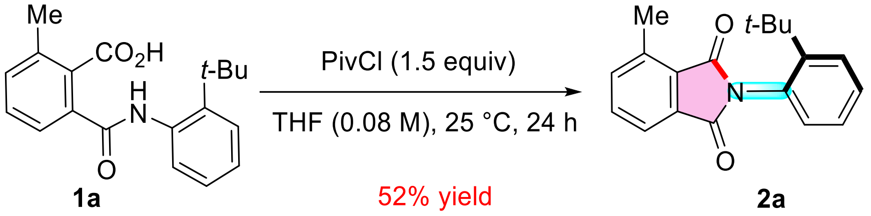

In a flame-dried screw-capped test tube equipped with a magnetic stir bar was taken 2-((2-(*tert*-butyl)phenyl)carbamoyl)-6- methylbenzoic acid **1a** (31.1 mg, 0.1 mmol, 1.0 equiv) followed by THF was added under argon atmosphere. To the reaction mixture, PivCl (18 µL, 0.15 mmol, 1.5 equiv) was added. Then the resultant reaction mixture was kept stirring at 25 °C for 24 h. The solvent was evaporated to get the crude residue, which was purified by flash column chromatography on silica gel to afford the 2-(2-(*tert*-butyl)phenyl)-4-methylisoindoline-1,3-dione **2a** (12.0 mg, 56% yield).

This experiment indicates that the generaed HCl during the pivolyl anhydride fromation may mediate the formation of ***2a****. To confirm the role of HCl for the formation of phthalimide, we have performed the reaction of isomide* ***7a*** *with HCl (see below).*

(e) Reaction of Isoimide Intermediate 7a with 4M HCl in Dioxane

In a flame-dried screw-capped test tube equipped with a magnetic stir bar was taken (*E*)-3-((2-(*tert*-butyl)phenyl)imino)-7-methyl isobenzofuran-1(3*H*)-one **7a** (29.3 mg, 0.1 mmol, 1.0 equiv) followed by THF (0.8 mL) was added under argon atmosphere. To the reaction, 4M HCl in dioxane (100 µL, 0.2 mmol, 0.2 equiv) was added and the resulting reaction mixture was stirred for 24 h. The solvent was evaporated to get the crude residue, which was purified by flash column chromatography on silica gel to afford the 2-(2-(*tert*-butyl)phenyl)-4-methylisoindoline-1,3-dione **2a** (16.9 mg, 58% yield).

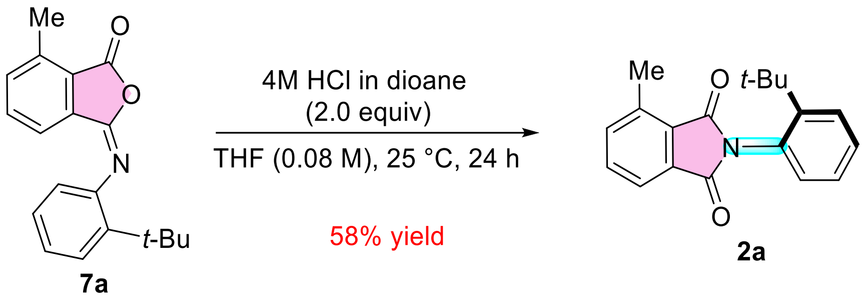

*The formation of phthalimide **2a** formed concludes that HCl can mediate the formation of **2a**.*

2.2 Studies on Rotation Barrier of C-N Axially Chiral Phthalimides

(a) Dependence of Enantioselectivity on Temperature

Compound **2a/2n** was dissolved in toluene to make a 2.0 mg/mL solution in a Schlenk tube. Then the solution was allowed to stir at 40 °C. After two hours, a small amount of sample was taken out from solution (without disturbing) and enantioselectivity was determined using HPLC. Then the temperature was increased by 10 °C and allowed to stir for two more hours and checked the enantioselectivity, and same procedure was repeated up to 100 °C.

Supplementary Table 2: The variation of enantiomeric excess vs temperature

Temperature (°C)	ee(%) for **2a**	ee(%) for **2n**
40	98	90
50	98	90
60	98	88
70	98	85
80	96	76
90	90	58
100	76	24
110	44	4
120	24	0
130	8	-
140	0	-

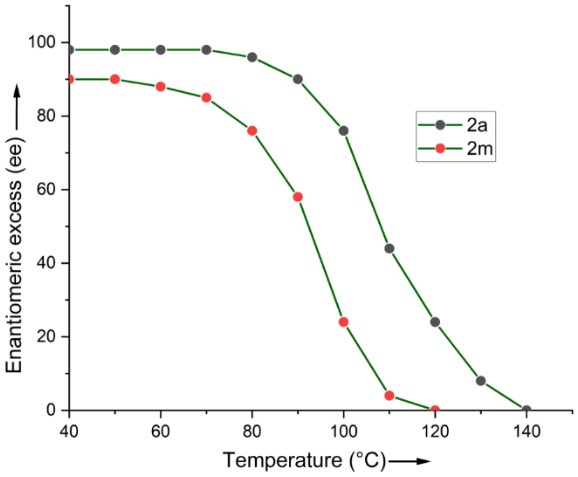

Supplementary Figure 2: the plot of variation of enantiomeric excess vs temperature

*In this experiment, the restricted rotation around the axial C-N bond of (P)-2-(2-(tert-Butyl)phenyl)-4-methylisoindoline-1,3-dione (**2a**) was investigated at variable temperature while acquiring the HPLC data. The C-N bond rotation was restricted up to 70 °C, from 80 °C*

onwards, the enantioselectivity of ***2a*** *started decreasing and at ~140 °C the free rotation around the C-N bond is fully allowed, resulting in racemic product.*

*In case of (P)-4-Methyl-2-(4-methyl-2-(2-phenylpropan-2-yl)phenyl)isoindoline-1,3-dione (**2n**), the C-N bond rotation was restricted up to 50 °C, from 60 °C the enantioselectivity of* ***2n*** *started decreasing and at ~120 °C the free rotation around the C-N bond is fully allowed, resulting in racemic product.*

(b) Experimental Determination of Axially Chiral C-N Bond Rotation Barriers

Compound **2a/2n** was dissolved in toluene to make a 2.0 mg/mL solution in a Schlenk tube. The tube was sealed and placed in a pre-heated oil bath at 100 °C. At 30 min time intervals, the Schlenk tube was removed briefly (~1 min) from the bath and a 20 μL aliquot was removed via syringe and injected onto the appropriate analytical HPLC column to measure the enantiomeric ratio. This ratio was used to plot against time, and the barrier to rotation was calculated from the plot.[3] In the y-axis of the plot, m denotes the ratio of the minor enantiomer, and M denotes the ratio of the major enantiomer. A sample full data treatment is shown for the experimental set at 373.15 K (100 °C). k_B = Boltzmann's constant [1.381×10^{-23}J/K], T = temperature in kelvin, h = Planck's constant [6.626×10^{-34} J s], R = universal gas constant [8.3145 J/mol], t is time in sec. The half-life of racemization ($t_{1/2}$) at room temperature is determined by calculating the rate constant for racemization (k_{rac}) from the barrier of rotation at 25 °C, assuming that $\Delta G^{\ddagger}_{rot}$is mostly constant over a large temperature range.

Then the simplified supplementary equation for racemization is:

$$\ln\frac{M+m}{M-m} = k_{rac}t + c = 2k_{rot}t + c$$ ………… Supplementary equation (1)

where, $$k_{rot} = \frac{slope}{2}$$ ………… Supplementary equation (2)

and $$k_{rac} = 2k_{rot} = \frac{2k_BT}{h}e^{\left(-\frac{\Delta G^{\ddagger}_{rot}}{RT}\right)}$$ ………… Supplementary equation (3)

Supplementary Table 3: Study of racemization of 2a, trial 1

time (s)	% of major enantiomer (M)	% of minor enantiomer (m)	ln[(M + m) / (M - m)]
0	99.29	0.71	0.0143
1800	95.6998	4.3002	0.0899
3600	95.0663	4.9337	0.1038
5400	93.9074	6.0926	0.1299

7200	92.3655	7.6345	0.1656
9000	90.3583	9.6417	0.2142
10800	88.6307	11.3693	0.2579
12600	86.9039	13.0961	0.3037
14400	84.939	15.061	0.3584
16200	84.036	15.964	0.3846
18000	82.8012	17.1988	0.4215
19800	81.4386	18.5614	0.4639
21,600	79.8388	20.1612	0.5162
23400	78.7478	21.2522	0.5534

Supplementary Table 4: Study of racemization of 2n, trial 1

time (s)	% of major enantiomer (M)	% of minor enantiomer (m)	ln[(M + m) / (M - m)]
0	94	6	0.1278
1800	91.5	8.5	0.1863
3600	84.579	15.421	0.3688
5400	78.94	21.06	0.5468
7200	73.413	26.587	0.7587
9000	69.831	30.169	0.9248
10800	66.478	33.522	1.1020
12600	63.401	36.599	1.3167
14400	60.031	39.969	1.6063
16200	58.677	41.323	1.7513
18000	56.777	43.223	1.9985
19800	55.871	44.129	2.1420
21,600	54.736	45.264	2.3568
23400	54.097	45.903	2.5018

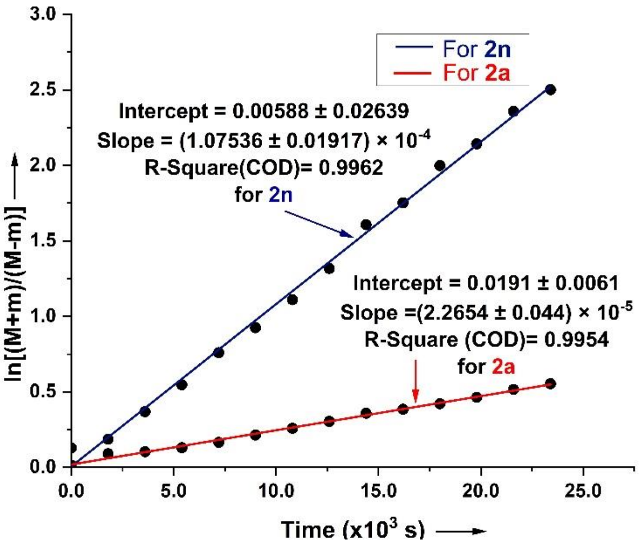

Supplementary Figure 3: Plot on variation of enantioselectivity with time for 2a and 2n, trial 1

Calculation for ***2a***,

$$k_{rot} = \frac{2.2654\times10^{-5}s^{-1}}{2} = 1.1327\times\ 10^{-5}\ \mathrm{s}^{-1}$$ .... Supplementary equation (2)

$$K^{\ddagger}_{rot} = \frac{k_{rot}h}{k_BT}$$ ………………….. Supplementary equation (4)

$$= \frac{(1.1327\times10^{-5}\ \mathrm{s}^{-1})\times(6.626\times10^{-34}\ \mathrm{J.s})}{(1.381\times10^{-23}\ \mathrm{J.K}^{-1})\times(373.15\ \mathrm{K})}$$

$$= 1.4564\times10^{-18}$$

$$\Delta\boldsymbol{G}^{\ddagger}_{\boldsymbol{rot}} = -RTlnK^{\ddagger}_{rot}$$ ………………….. Supplementary equation (5)

$$= -(8.314\,J\ \mathrm{mol}^{-1}\mathrm{K}^{-1})\times(373.15\ \mathrm{K})\times\ln(1.4564\times10^{-18})$$

$$= \mathbf{127.415\ kJ.mol^{-1}}$$

$$= \mathbf{30.5\ kcal.mol^{-1}}$$

Calculation for ***2n***,

$$k_{rot} = \frac{1.0754\times10^{-4}s^{-1}}{2} = 5.377\times\ 10^{-5}\ \mathrm{s}^{-1}$$ .... Supplementary equation (2)

$$K^{\ddagger}_{rot} = \frac{k_{rot}h}{k_BT} = \frac{(5.377\times10^{-5}\ \mathrm{s}^{-1})\times(6.626\times10^{-34}\ \mathrm{J.s})}{(1.381\times10^{-23}\ \mathrm{J.K}^{-1})\times(373.15\ \mathrm{K})}$$ ………Supplementary equation (4)

$$= 6.9138\times10^{-18}$$

$$\Delta\boldsymbol{G}^{\ddagger}_{\boldsymbol{rot}} = -RTlnK^{\ddagger}_{rot}$$ ……………….. Supplementary equation (5)

$$= -(8.314\,J\ \mathrm{mol}^{-1}\mathrm{K}^{-1})\times(373.15\ \mathrm{K})\times\ln(6.9138\times10^{-18})$$

$$= \mathbf{122.583\ kJ.mol^{-1}}$$

$$= \mathbf{29.3\ kcal.mol^{-1}}$$

The kinetic experiment for both 2a and 2n have been repeated 4 more times.

Supplementary Table 5: Study of racemization of 2a, trial 2

time (s)	% of major enantiomer (M)	% of minor enantiomer (m)	ln[(M + m) / (M - m)]
0	99.63	0.37	0.0074
1800	97.61	2.39	0.0489

3600	95.77	4.23	0.0884
5400	94.3	5.7	0.1210
7200	92.47	7.53	0.1632
9000	90.93	9.07	0.2001
10800	89.19	10.81	0.2436
12600	87.77	12.23	0.2805
14400	85.26	14.74	0.3492
16200	84.17	15.83	0.3806
18000	83.2	16.8	0.4094

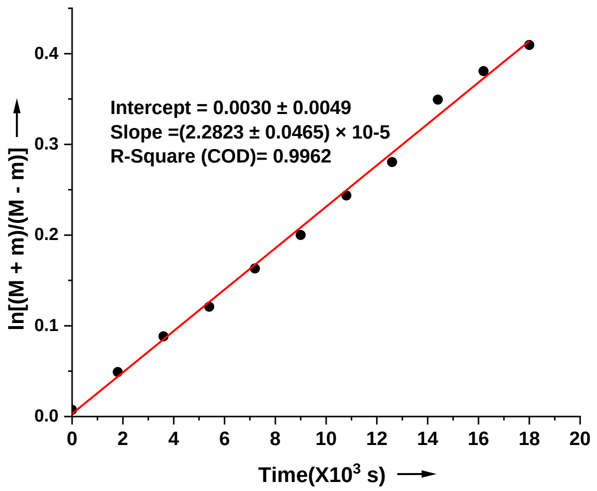

Supplementary Figure 4: Plot on variation of enantioselectivity with time for 2a trial 2

Calculation,

$k_{rot} = \frac{2.2823\times10^{-5}s^{-1}}{2} = 1.1411\times\ 10^{-5}\ \text{s}^{-1}$........... Supplementary equation (2)

$K^{\ddagger}_{rot} = \frac{k_{rot}h}{k_BT}$.........Supplementary equation (4)

$= \frac{(1.1411\times10^{-5}\ \text{s}^{-1})\times(6.626\times10^{-34}\ \text{J.s})}{(1.381\times10^{-23}\ \text{J.K}^{-1})\times(373.15\ \text{K})} = 1.4682\times10^{-18}$

$\boldsymbol{\Delta G^{\ddagger}_{rot}} = -RTlnK^{\ddagger}_{rot}$ ....Supplementary equations (5)

$$= -(8.314\,J\ \text{mol}^{-1}\text{K}^{-1})\times(373.15\ \text{K})\times\ln(1.4682\times10^{-18})$$

$$= \mathbf{127.3903\ kJ.mol^{-1}}$$

$$= \mathbf{30.4761\ kJ.mol^{-1}}$$

Supplementary Table 6: Study of racemization of 2a, trial 3

time (s)	% of major enantiomer (M)	% of minor enantiomer (m)	ln[(M + m) / (M - m)]
0	99.63	0.37	0.0074
1800	97.47	2.53	0.0519
3600	95.51	4.49	0.0941
5400	93.69	6.31	0.1349
7200	91.98	8.02	0.1748
9000	90.93	9.07	0.2001
10800	88.82	11.18	0.2531
12600	87.24	12.76	0.2946
14400	85.56	14.44	0.3408
16200	84.32	15.68	0.3763

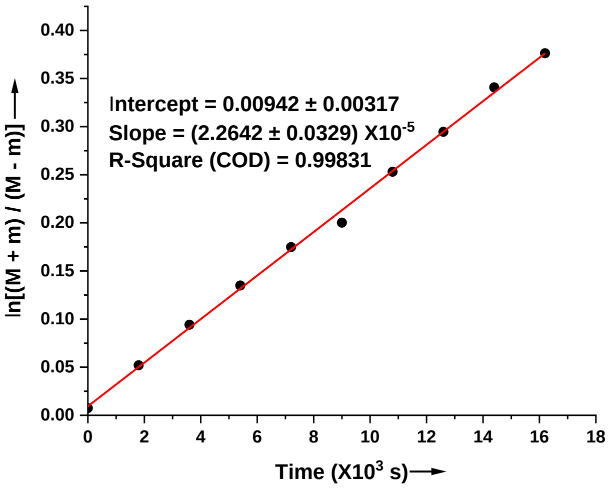

Supplementary Figure 5: Plot on variation of enantioselectivity with time for 2a trial 3

Calculation,

$k_{rot} = \frac{2.2642 \times 10^{-5} s^{-1}}{2} = 1.1321 \times 10^{-5}\ \mathrm{s}^{-1}$ .... Supplementary equation (2)

$K^{\ddagger}_{rot} = \frac{k_{rot}h}{k_B T} = \frac{(1.1321 \times 10^{-5}\ \mathrm{s}^{-1}) \times (6.626 \times 10^{-34}\ \mathrm{J.s})}{(1.381 \times 10^{-23}\ \mathrm{J.K}^{-1}) \times (373.15\ \mathrm{K})}$ .... Supplementary equation (4)

$= 1.4556 \times 10^{-18}$

$\Delta G^{\ddagger}_{rot} = -RT ln K^{\ddagger}_{rot}$ .... Supplementary equation (5)

$= -(8.314\, J\ \mathrm{mol}^{-1}\mathrm{K}^{-1}) \times (373.15\ \mathrm{K}) \times \ln(1.4556 \times 10^{-18})$

$$= \mathbf{127.4172\ kJ.mol^{-1}}$$

$$= \mathbf{30.4825\ kJ.mol^{-1}}$$

Supplementary Table 7: Study of racemization of 2a, trial 4

time (s)	% of major enantiomer (M)	% of minor enantiomer (m)	ln[(M + m) / (M - m)]
0	99.63	0.37	0.0074
1800	98.11	1.89	0.03853295
3600	96.12	3.88	0.08077631
5400	94.14	5.86	0.124656605
7200	92.13	7.87	0.171262929
9000	90.14	9.86	0.219649662
10800	88.59	11.41	0.25902983
12600	87.03	12.97	0.300294611
14400	85.56	14.44	0.340801595
16200	84	16	0.385662481

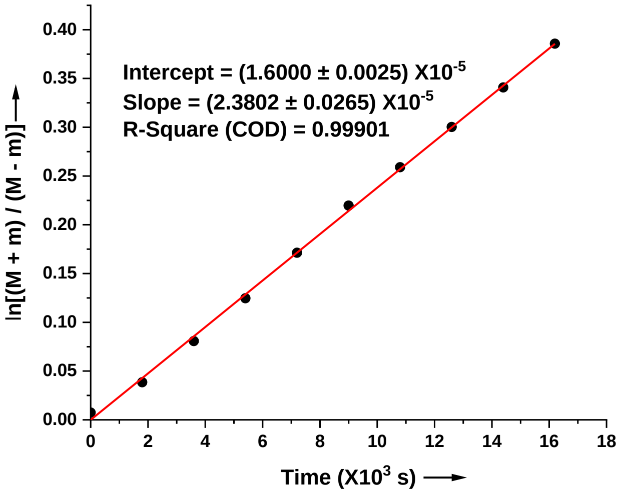

Supplementary Figure 6: Plot on variation of enantioselectivity with time for 2a trial 4

Calculation,

$k_{rot} = \frac{2.3802\times10^{-5}s^{-1}}{2} = 1.1901\times\ 10^{-5}\ \mathrm{s}^{-1}$ .... Supplementary equation (2)

$K_{rot}^{\ddagger} = \frac{k_{rot}h}{k_B T} = \frac{(1.1901\times10^{-5}\ \mathrm{s}^{-1})\times(6.626\times10^{-34}\ \mathrm{J.s})}{(1.381\times10^{-23}\ \mathrm{J.K}^{-1})\times(373.15\ \mathrm{K})}$ .... Supplementary equation (4)

$= 1.5313\times10^{-18}$

$\boldsymbol{\Delta G_{rot}^{\ddagger}} = -RT ln K_{rot}^{\ddagger}$ .... Supplementary equation (5)

$$= -(8.314\,J\ \text{mol}^{-1}\text{K}^{-1}) \times (373.15\ \text{K}) \times \ln(1.5313 \times 10^{-18})$$

$$= \mathbf{127.2603\ kJ.mol^{-1}}$$

$$= \mathbf{30.445\ kcal.mol^{-1}}$$

Supplementary Table 8: Study of racemization of 2a, trial 5

time (s)	% of major enantiomer (M)	% of minor enantiomer (m)	ln[(M + m) / (M - m)]
0	99.63	0.37	0.0074
1800	97.21	2.79	0.0574
3600	95.28	4.72	0.0991
5400	93.2	6.8	0.1462
7200	91.48	8.52	0.1868
9000	89.51	10.49	0.2355
10800	87.9	12.1	0.2771
12600	86.18	13.82	0.3235
14400	84.73	15.27	0.3644
16200	83.32	16.68	0.4059

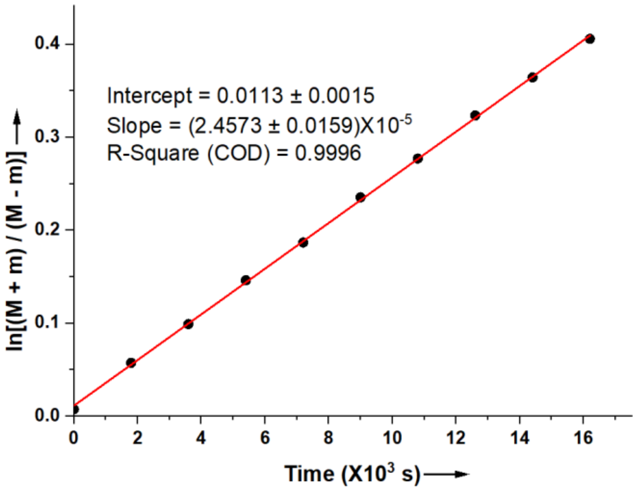

Supplementary Figure 7: Plot on variation of enantioselectivity with time for 2a trial 5

Calculation,

$$k_{rot} = \frac{2.4573\times10^{-5}s^{-1}}{2} = 1.22865\times\ 10^{-5}\ \text{s}^{-1}\ \ldots\ldots\ldots\ \text{Supplementary equation (2)}$$

$$K^{\ddagger}_{rot} = \frac{k_{rot}h}{k_B T} = \frac{(1.22865\times10^{-5}\ \text{s}^{-1})\times(6.626\times10^{-34}\ \text{J.s})}{(1.381\times10^{-23}\ \text{J.K}^{-1})\times(373.15\ \text{K})}\ \ldots\ldots\ldots\ \text{Supplementary equation (4)}$$

$= 1.5809 \times 10^{-18}$

$\Delta G^{\ddagger}_{rot} = -RTlnK^{\ddagger}_{rot}$ ………… Supplementary equation (5)

$$= -(8.314\,J\ \text{mol}^{-1}\text{K}^{-1}) \times (373.15\ \text{K}) \times \ln(1.5809 \times 10^{-18})$$

$$= \mathbf{127.1615\ kJ.mol^{-1}}$$

$$= \mathbf{30.4214\ kJ.mol^{-1}}$$

Supplementary Table 9: Study of racemization of 2n, trial 2

time (s)	% of major enantiomer (M)	% of minor enantiomer (m)	ln[(M + m) / (M - m)]
0	94	6	0.09123848
1800	91.5	8.5	0.216416231
3600	84.579	15.421	0.392746336
5400	78.94	21.06	0.590590592
7200	73.413	26.587	0.807436327
9000	69.831	30.169	0.995875211
10800	66.478	33.522	1.205974807
12600	63.401	36.599	1.38309947
14400	60.031	39.969	1.598497972
16200	58.677	41.323	1.814005078

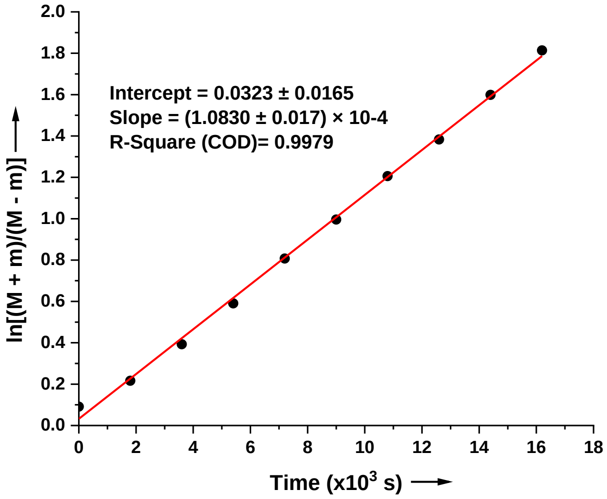

Supplementary Figure 8: Plot on variation of enantioselectivity with time for 2n trial 2

Calculation for 2n,

$$k_{rot} = \frac{1.0830\times10^{-4}s^{-1}}{2} = 5.415 \times 10^{-5}\ s^{-1} \text{........... Supplementary equation (2)}$$

$$K^{\ddagger}_{rot} = \frac{k_{rot}h}{k_B T} = \frac{(5.415\times10^{-5}\ s^{-1})\times(6.626\times10^{-34}\ J.s)}{(1.381\times10^{-23}\ J.K^{-1})\times(373.15\ K)} \text{........... Supplementary equation (4)}$$

$$= 6.9626 \times 10^{-18}$$

$$\Delta G^{\ddagger}_{rot} = -RTlnK^{\ddagger}_{rot} \text{........... Supplementary equation (4)}$$

$$= -(8.314\ J\ mol^{-1}K^{-1}) \times (373.15\ K) \times \ln(6.9138 \times 10^{-18})$$

$$= \mathbf{122.5623\ kJ.mol^{-1}}$$

$$= \mathbf{29.3211\ kJ.mol^{-1}}$$

Supplementary Table 10: Study of racemization of 2n, trial 3

time (s)	% of major enantiomer (M)	% of minor enantiomer (m)	ln[(M + m) / (M - m)]
0	95.64	4.36	0.09123848
1800	92.01	7.99	0.17411532
3600	84.73	15.27	0.364419139
5400	77.34	22.66	0.603672174
7200	72.85	27.15	0.783071888
9000	68.61	31.39	0.988323935
10800	66.76	33.24	1.09302791
12600	63.92	36.08	1.278696351
14400	61.59	38.41	1.461880348
16200	59.5	41.5	1.724748759

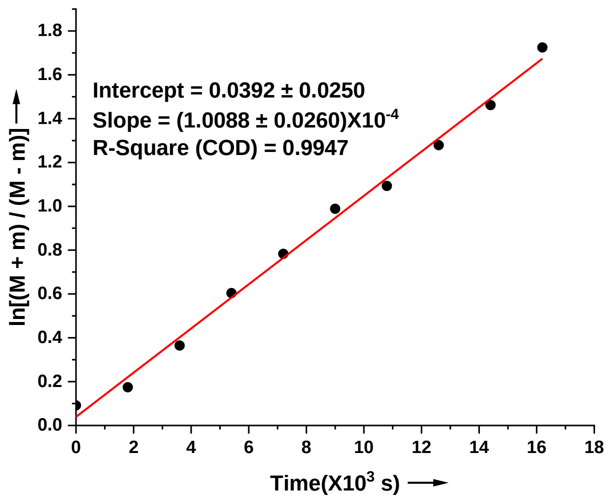

Supplementary Figure 9: Plot on variation of enantioselectivity with time for 2n trial 3

Calculation for 2n,

$k_{rot} = \frac{1.0088\times10^{-4}s^{-1}}{2} = 5.044\times 10^{-5}\ s^{-1}$ .... Supplementary equation (2)

$K^{\ddagger}_{rot} = \frac{k_{rot}h}{k_B T} = \frac{(5.044\times10^{-5}\ s^{-1})\times(6.626\times10^{-34}\ J.s)}{(1.381\times10^{-23}\ J.K^{-1})\times(373.15\ K)}$ .... Supplementary equation (4)

$= 6.4902\times10^{-18}$

$\Delta G^{\ddagger}_{rot} = -RTlnK^{\ddagger}_{rot}$ .... Supplementary equation (5)

$= -(8.314\ J\ mol^{-1}K^{-1})\times(373.15\ K)\times\ln(6.4902\times10^{-18})$

$= \mathbf{122.7803\ kJ.mol^{-1}}$

$= \mathbf{29.3732\ kJ.mol^{-1}}$

Supplementary Table 11: Study of racemization of 2n, trial 4

time (s)	% of major enantiomer (M)	% of minor enantiomer (m)	ln[(M + m) / (M - m)]
0	95.64	4.36	0.0912
1800	92.49	7.51	0.1627
3600	87.2	12.8	0.2957
5400	81.05	18.95	0.4764
7200	76.3	23.7	0.6424
9000	73.37	26.63	0.7606
10800	68.22	31.78	1.010
12600	64.72	35.28	1.2228
14400	62.24	37.76	1.4073
16200	60.03	39.97	1.6064

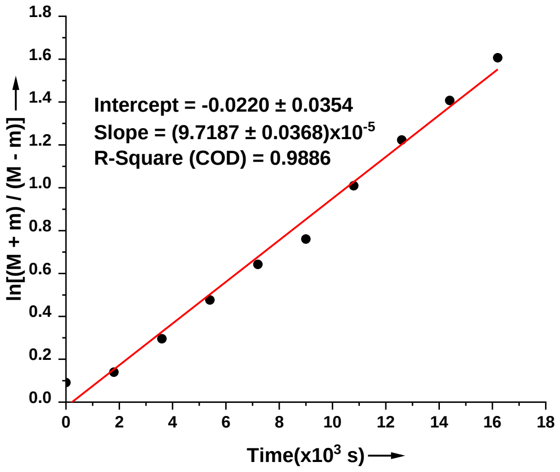

Supplementary Figure 10: Plot on variation of enantioselectivity with time for 2n trial 4

Calculation,

$$k_{rot} = \frac{9.7187\times10^{-5}s^{-1}}{2} = 4.8594\times 10^{-5}\ s^{-1} \text{ .... Supplementary equation (2)}$$

$$K^{\ddagger}_{rot} = \frac{k_{rot}h}{k_B T} = \frac{(4.8594\times10^{-5}\ s^{-1})\times(6.626\times10^{-34}\ J.s)}{(1.381\times10^{-23}\ J.K^{-1})\times(373.15\ K)} \text{........... Supplementary equation (4)}$$

$$= 6.2527\times10^{-18}$$

$$\Delta G^{\ddagger}_{rot} = -RT ln K^{\ddagger}_{rot} \text{ .... Supplementary equation (5)}$$

$$= -(8.314\ J\ mol^{-1}K^{-1})\times(373.15\ K)\times \ln(6.2527\times10^{-18})$$

$$= \mathbf{122.8957\ kJ.mol^{-1}}$$

$$= \mathbf{29.4008\ kcal.mol^{-1}}$$

Supplementary Table 12: Study of racemization of 2n, trial 5

time (s)	% of major enantiomer (M)	% of minor enantiomer (m)	ln[(M + m) / (M - m)]
0	95.64	4.36	0.0912
1800	89.51	10.49	0.2355
3600	82.63	17.37	0.4268
5400	76.35	23.65	0.6406
7200	71.96	28.04	0.8228
9000	68.17	31.83	1.0122
10800	65.06	34.94	1.1999
12600	62.05	37.95	1.4229

14400	60.02	39.98	1.6074
16200	58.14	41.86	1.8152

The plot for **2n** is,

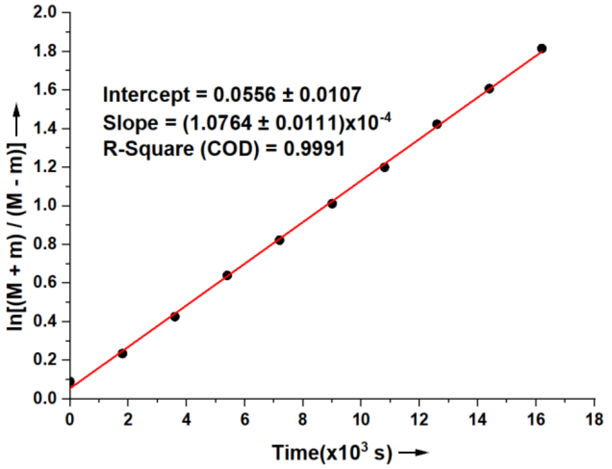

Supplementary Figure 11: Plot on variation of enantioselectivity with time for 2n trial 5

Calculation,

$k_{rot} = \frac{1.0764\times10^{-4}s^{-1}}{2} = 4.382\times 10^{-5}\ s^{-1}$........... Supplementary equation (2)

$K^{\ddagger}_{rot} = \frac{k_{rot}h}{k_BT} = \frac{(4.382\times10^{-5}\ s^{-1})\times(6.626\times10^{-34}\ J.s)}{(1.381\times10^{-23}\ J.K^{-1})\times(373.15\ K)}$ .... Supplementary equation (4)

$= 6.9252\times 10^{-18}$

$\Delta G^{\ddagger}_{rot} = -RTlnK^{\ddagger}_{rot}$ .... Supplementary equation (5)

$= -(8.314\ J\ mol^{-1}K^{-1})\times(373.15\ K)\times \ln(6.9252\times10^{-18})$

$= \mathbf{122.5788\ kJ.mol^{-1}}$

$= \mathbf{29.3250\ kcal.mol^{-1}}$

Supplementary Table 13: Study of racemization of 2a, all trial

No. of experiment	$\Delta G^{\ddagger}_{rot}$ (kcal./mol)
1	30.4822
2	30.4761
3	30.4825
4	30.445
5	30.4214

Mean = 30.4614 kcal/mol

Standard deviation (SD) = 0.0272

$\Delta G^{\ddagger}_{rot}$ **= Mean ± SD = (30.46 ± 0.03) kcal/mol** .... Supplementary equation (6)

$$k_{rac}\, at\ 25\ °C = \frac{2 \times (1.381 \times\ 10^{-23}\ \mathrm{J.K^{-1}}) \times (298.15\ \mathrm{K})}{(6.626 \times\ 10^{-34}\ \mathrm{J.s})} e^{\left(-\frac{127322.8\ J.mol^{-1}}{(8.314\ J\ \mathrm{mol^{-1}K^{-1}})\times(298.15\ \mathrm{K})}\right)}$$

$= 6.1259 \times\ 10^{-10}\ s^{-1}$ ……… Supplementary equation (3)

***t*1/2 at 25 °C** = $\frac{ln2}{k_{rac}} = \frac{0.693}{6.1259\times\ 10^{-10}\ s^{-1}} = 1.1312 \times\ 10^{9}\ s = \mathbf{35.8\ year}$ ……… Supplementary equations (7)

Supplementary Table 14: Study of racemization of 2n, all trial

No. of experiment	$\Delta G^{\ddagger}_{rot}$ **(kcal/mol)**
1	29.3260
2	29.3211
3	29.3732
4	29.4008
5	29.3250

Mean = 29.3492 kcal/mol

Standard deviation (SD) = 0.0359

$\Delta G^{\ddagger}_{rot}$ **= Mean ± SD = (29.35 ± 0.04) kcal/mol** ……… Supplementary equation (6)

$$k_{rac}\, at\ 25\ °C = \frac{2 \times (1.381 \times\ 10^{-23}\ \mathrm{J.K^{-1}}) \times (298.15\ \mathrm{K})}{(6.626 \times\ 10^{-34}\ \mathrm{J.s})} e^{\left(-\frac{122683\ J.mol^{-1}}{(8.314\ J\ \mathrm{mol^{-1}K^{-1}})\times(298.15\ \mathrm{K})}\right)}$$

$= 3.9817 \times\ 10^{-9}\ s^{-1}$ ……… Supplementary equation (3)

***t*1/2 at 25 °C** = $\frac{ln2}{k_{rac}} = \frac{0.693}{3.9817\times\ 10^{-9}\ s^{-1}} = 1.7404 \times\ 10^{8}\ s = \mathbf{5.5\ year}$ ……… Supplementary equation (7)

The results of trail 1 for both **2a** and **2n** have been presented in the manuscript (See Scheme 3B ii)

(c) Computational Details of C-N Bond Rotation Barriers

The theoretical rotational free energy barriers for the substrates **2a** and **2n** were computed using the Gaussian 16 software.[4] All the stationary points were optimized using hybrid functional B3LYP-D3 and standard split valence Pople's type basis set 6-31G(d,p).[5,6]

Frequency calculations on these structures reveal the vibrational normal modes which were used to ascertain the nature of the geometries (minima/saddle point) as well as provide the thermal corrections to the electronic energy to obtain the free energy values. Intrinsic Reaction Coordinate (IRC)[7] calculations were carried out on the TSs (Transition States) to make sure that the located TS can connect to related intermediates (**2a**$_R$ and **2n**$_R$). On these optimized structures, we carried out single point calculations at the B3LYP-D3BJ/6-311++G(d,p) level of theory with the solvation effects of toluene being simulated by an SMD[8](solvation model density) model.

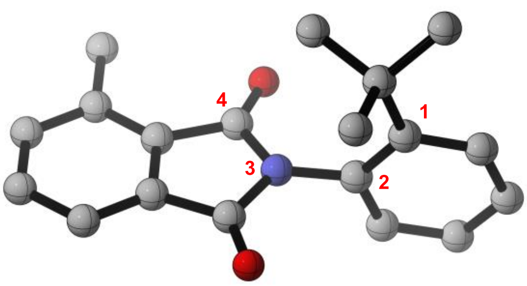

2a

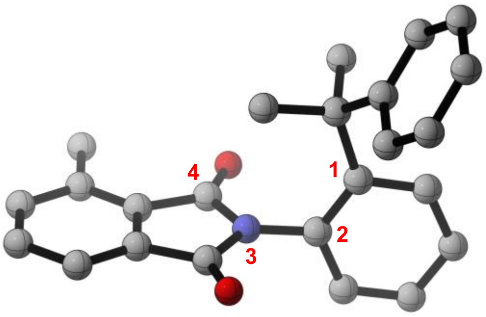

2n

Rotational Free Energy (kcal/mol)	**2a**	**2n**
	31.9	30.2

Supplementary Figure 12: Optimized geometries of rotational TSs (**2a** and **2n**) and the corresponding free energy barriers (kcal/mol) at the PCM$_{(toluene)}$/B3LYP-D3BJ/6-311++G(d,p)//B3LYP-D3/6-31G(d,p) level of theory.

2.3 X-Ray Data

X-ray data of 1a

X-ray intensity data measurements of compound **1a** (crystallized from MeOH at 25 °C; a sample of chromatographically pure **1a** was dissolved in MeOH and was allowed to evaporate slowly at 25 °C) was carried out on a Bruker D8 Quest diffractometer. The intensity measurements were carried out with Mo rotating anode diffraction source (Mo-Kα= 0.71073 Å) at 120(2) K temperature. The X-ray generator was operated at 45 kV and 30 mA. A preliminary set of cell constants and an orientation matrix were calculated from a set of fast scans (180 frames). The X-ray data collection was monitored by the APEX4 software (Bruker). All the data were corrected for Lorentzian, polarization, and absorption effects using SAINT and SADABS programs (Bruker). The structure was solved using the APEX4 (Bruker) program suite using direct methods with the SHELXS – 97 (Sheldrick, 2008) structure refinement program. Using Least Squares minimization, the model was refined with a version of SHELXL - 2019/1 (Sheldrick, 2019). All the hydrogen atoms were placed in geometrically idealized positions and constrained to ride on their parent atoms. An ORTEP view of the structure was shown with the 50% probability displacement ellipsoids and the packing diagram was made using Mercury software.

Identification code	**1a**
CCDC	2261808
Empirical formula	$C_{20}H_{25}NO_4$
Formula weight	343.41
Temperature/K	120(2)
Crystal system	monoclinic
Space group	C2/c
a/Å	19.776(10)
b/Å	8.310(4)
c/Å	23.426(12)
α/°	90
β/°	104.821(11)
γ/°	90
Volume/Å3	3722(3)
Z	8
ρcalcg/cm3	1.226
μ/mm-1	0.085

F(000)	1472.0
Crystal size/mm^3	0.31 × 0.27 × 0.22
Radiation	MoKα (λ = 0.71073)
2Θ range for data collection/°	5.346 to 54.164
Index ranges	-25 ≤ h ≤ 25, -10 ≤ k ≤ 10, -29 ≤ l ≤ 29
Reflections collected	46224
Independent reflections	4098 [R_{int} = 0.0619, R_{sigma}= 0.0276]
Data/restraints/parameters	4098/0/237
Goodness-of-fit on F2	1.145
Final R indexes [I>=2σ (I)]	R_1 = 0.0527, wR_2 = 0.1207
Final R indexes [all data]	R_1 = 0.0615, wR_2 = 0.1257
Largest diff. peak/hole / e Å-3	0.28/-0.25

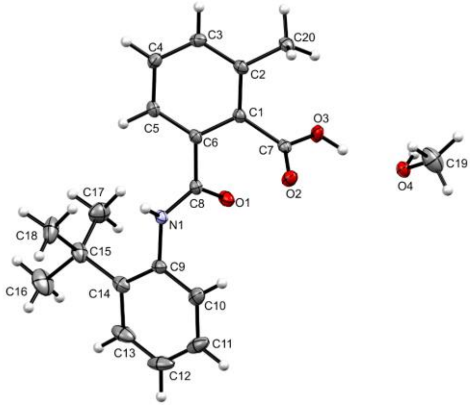

Supplementary Figure 13: ORTEP representation of the X-ray structure of **1a** (thermal ellipsoids at 50% probability)

X-ray data of 1w

X-ray intensity data measurements of compound **1w** (crystallized from MeOH at 25 °C; a sample of chromatographically pure **1w** was dissolved in MeOH and was allowed to evaporate slowly at 25 °C) was carried out on a Bruker D8 Quest diffractometer. The intensity measurements were carried out with Mo rotating anode diffraction source (Mo-Kα= 0.71073 Å) at 120(2) K temperature. The X-ray generator was operated at 45 kV and 30 mA. A preliminary set of cell constants and an orientation matrix were calculated from a set of fast scans (180 frames). The X-ray data collection was monitored by the APEX4 software (Bruker). All the data were corrected for Lorentzian, polarization, and absorption effects using SAINT and SADABS programs (Bruker). The structure was solved using the APEX4 (Bruker) program suite using direct methods with the SHELXS–97 (Sheldrick, 2008) structure refinement program. Using Least Squares minimization, the model was refined with a version of SHELXL

- 2019/1 (Sheldrick, 2019). All the hydrogen atoms were placed in geometrically idealized positions and constrained to ride on their parent atoms. An ORTEP view of the structure was shown with the 50% probability displacement ellipsoids and the packing diagram was made using Mercury software.

Identification code	**1w**
CCDC	2261807
Empirical formula	$C_{19}H_{22}ClNO$
Formula weight	363.82
Temperature/K	120(2)
Crystal system	monoclinic
Space group	C2/c
a/Å	19.7349(16)
b/Å	8.3084(5)
c/Å	23.2543(19)
α/°	90
β/°	104.379(3)
γ/°	90
Volume/Å3	3693.5(5)
Z	8
ρcalcg/cm3	1.309
μ/mm-1	0.230
F(000)	1536.0
Crystal size/mm^3	0.37 × 0.32 × 0.25
Radiation	MoKα (λ = 0.71073)
2Θ range for data collection/°	5.812 to 49.996
Index ranges	-23 ≤ h ≤ 23, -9 ≤ k ≤ 9, -27 ≤ l ≤ 27
Reflections collected	10432
Independent reflections	3159 [R_{int} = 0.0914, R_{sigma} = 0.0881]
Data/restraints/parameters	3159/0/236
Goodness-of-fit on F2	1.027
Final R indexes [I>=2σ (I)]	R_1 = 0.0487, wR_2 = 0.0974
Final R indexes [all data]	R_1 = 0.0924, wR_2 = 0.1125
Largest diff. peak/hole / e Å-3	0.26/-0.25

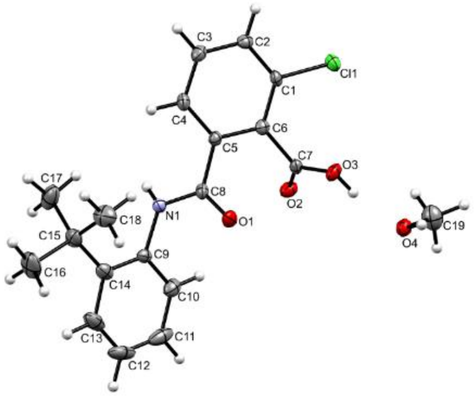

Supplementary Figure 14: ORTEP representation of the X-ray structure of **1w** (thermal ellipsoids at 50% probability)

X-ray data of 2w

X-ray intensity data measurements of compound **2w** (crystallized from $CDCl_3$ at 25 °C; a sample of chromatographically pure **2w** was dissolved in $CDCl_3$ and was allowed to evaporate slowly at 25 °C) was carried out on Bruker APEX II Ultra diffractometer. The intensity measurements were carried out with Mo rotating anode diffraction source (Mo-Kα= 0.71073 Å) at 100(2) K temperature. The X-ray generator was operated at 45 kV and 80 mA. A preliminary set of cell constants and an orientation matrix were calculated from two matrix sets of 40 frames (each matrix run consists of 20 frames). Data were collected with ω scan width of 0.5° at different settings of φ and 2θ with a frame time of 10 secs keeping the sample-to-detector distance fixed at 5.82 cm. The X-ray data collection was monitored by APEX3 program (Bruker, 2016).5All the data were corrected for Lorentzian, polarization and absorption effects using SAINT and SADABS programs (Bruker, 2016). Using the APEX3 (Bruker) program suite, the structure was solved with the ShelXS-97 (Sheldrick, 2008) structure solution program, using direct methods. The model was refined with a version of ShelXL-2018/3 (Sheldrick, 2015) using Least Squares minimization. All the hydrogen atoms were placed in a geometrically idealized position and constrained to ride on its parent atoms, An ORTEP III8 view of the compound was drawn with 50% probability displacement ellipsoids, and H atoms are shown as small spheres of arbitrary radii.

Identification code	**2w**
CCDC	2252546
Empirical formula	$C_{18}H_{16}NO_2Cl$
Formula weight	313.77
Temperature/K	100(2)

Crystal system	orthorhombic
Space group	P212121
a/Å	7.8236(3)
b/Å	13.1423(5)
c/Å	15.1898(6)
α/°	90
β/°	90
γ/°	90
Volume/Å3	1561.82(10)
Z	4
ρcalcg/cm3	1.334
μ/mm-1	0.251
F(000)	656.0
Radiation	MoKα (λ = 0.71073)
2Θ range for data collection/°	4.098 to 61.162
Index ranges	-11 ≤ h ≤ 11, -18 ≤ k ≤ 18, -21 ≤ l ≤ 21
Reflections collected	45987
Independent reflections	4751 [R_{int} = 0.0568, R_{sigma} = 0.0328]
Data/restraints/parameters	4751/0/199
Goodness-of-fit on F2	1.077
Final R indexes [I>=2σ (I)]	R_1 = 0.0346, wR_2 = 0.0860
Final R indexes [all data]	R_1 = 0.0409, wR_2 = 0.0890
Largest diff. peak/hole / e Å-3	0.18/-0.33
Flack parameter	-0.03(2)

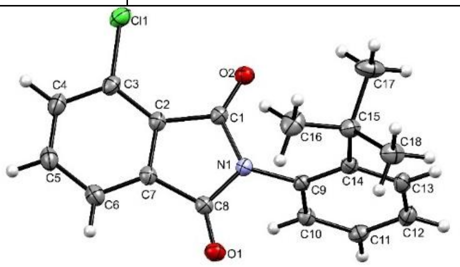

Supplementary Figure 15: ORTEP representation of the X-ray structure of **2w** (thermal ellipsoids at 50% probability)

X-ray data of 2u

X-ray intensity data measurements of compound **2x** (crystallized from $CDCl_3$ at 25 °C; a sample of chromatographically pure **2x** was dissolved in $CDCl_3$ and was allowed to evaporate slowly at 25 °C) was carried out on a Bruker D8 Quest diffractometer. The intensity measurements were carried out with Mo rotating anode diffraction source (Mo-Kα= 0.71073

Å) at 120(2) K temperature. The X-ray generator was operated at 45 kV and 30 mA. A preliminary set of cell constants and an orientation matrix were calculated from a set of fast scans (180 frames). Data was collected with ω scan width of 0.5° at different settings of φ and 2θ with a frame time of 15 secs keeping the sample to detector distance fixed at 45 mm, and 3-axis goniometer with fixed Kappa/Chi axis (54.736°). The X-ray data collection was monitored by the APEX4 software (Bruker). All the data were corrected for Lorentzian, polarization, and absorption effects using SAINT and SADABS programs (Bruker). The structure was solved using the APEX4 (Bruker) program suite using direct methods with the SHELXS – 97 (Sheldrick, 2008) structure refinement program. Using Least Squares minimization, the model was refined with a version of SHELXL - 2019/1 (Sheldrick, 2019). All the hydrogen atoms were placed in geometrically idealized positions and constrained to ride on their parent atoms. An ORTEP view of the structure was shown with the 50% probability displacement ellipsoids and the packing diagram was made using Mercury software 2022.3.0.

Identification code	**2x**
CCDC	2252545
Empirical formula	$C_{18}H_{16}BrNO_2$
Formula weight	358.23
Temperature/K	120(2)
Crystal system	orthorhombic
Space group	P212121
a/Å	7.8739(13)
b/Å	13.2497(18)
c/Å	15.195(2)
α/°	90
β/°	90
γ/°	90
Volume/Å3	1585.3(4)
Z	4
ρcalcg/cm3	1.501
μ/mm-1	2.599
F(000)	728.0
Radiation	MoKα (λ = 0.71076)
2Θ range for data collection/°	5.828 to 64.444
Index ranges	-11 ≤ h ≤ 11, -19 ≤ k ≤ 19, -22 ≤ l ≤ 22
Reflections collected	102979
Independent reflections	5593 [Rint = 0.0616, Rsigma = 0.0229]

Data/restraints/parameters	5593/0/202
Goodness-of-fit on F2	1.069
Final R indexes [I>=2σ (I)]	R1 = 0.0275, wR2 = 0.0657
Final R indexes [all data]	R1 = 0.0307, wR2= 0.0669
Largest diff. peak/hole / e Å-3	0.45/-0.54
Flack parameter	0.002(3)

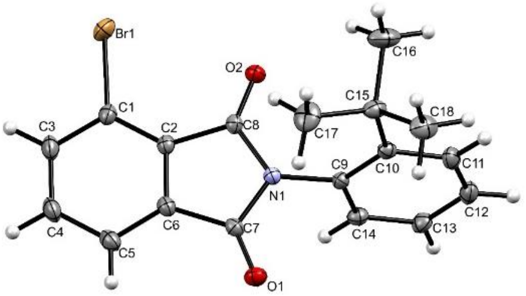

Supplementary Figure 16: ORTEP representation of the X-ray structure of **2x** (thermal ellipsoids at 50% probability)

2.4 Synthesis and Characterization of Phthalamic/Maleamic Acid Derivatives

2-((2-(*tert*-Butyl)phenyl)carbamoyl)-6-methylbenzoic acid (1a)

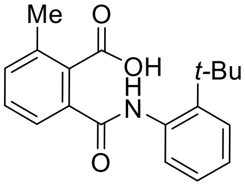

1a

Following the general procedure, 4-methylisobenzofuran-1,3-dione (486.4 mg, 3.0 mmol, 1.0 equiv) was dissolved in CH_2Cl_2 (1.5 mL, 2M). Then equimolecular amount of 2-*tert* butyl aniline (0.48 mL, 3.0 mmol, 1.0 equiv) was added and reaction mixture was stirred for 6 h at 25 °C. Then the reaction mixture was purified by flash column chromatography (Pet. ether- EtOAc: 50:50) to afford (2-((2-(*tert*-butyl)phenyl)carbamoyl)-6-methylbenzoic acid **1a** as a white solid (523 mg, 56% yield).

R_f (Pet. ether /EtOAc = 30/70): 0.23.

^{1}H NMR (400 MHz, DMSO-*d_6*) δ 13.00 (bs, 1H), 9.86 (bs, 1H), 7.56-7.54 (m, 1H), 7.49-7.41 (m, 3H), 7.27-7.25 (m, 2H), 7.13-7.11 (m, 1H), 2.37 (s, 3H), 1.38 (s, 9H). **^{13}C NMR (100 MHz, DMSO-*d_6*)** δ 169.7, 167.6, 147.0, 136.3, 135.3, 135.2, 134.3, 132.1, 131.9, 128.8, 127.2, 126.7, 126.4, 124.3, 34.9, 30.8, 19.3. **HRMS (ESI)** calculated $[M+Na]^+$ for $C_{19}H_{21}NO_3Na$: 334.1414, found: 334.1416. **FTIR (cm^{-1})** 3250, 2972, 2871, 1710, 1657, 1586, 1513, 1443, 1300, 1055, 1024. The structure of molecule confirmed by **single crystal XRD**; **CCDC**: 2261808.

2-((2-(*tert*-Butyl)phenyl)carbamoyl)-3-methylbenzoic acid (1a′)

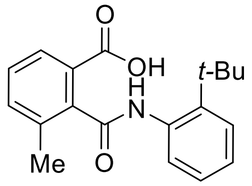

1a′

Following the general procedure, 4-methylisobenzofuran-1,3-dione (486.4 mg, 3.0 mmol, 1.0 equiv) was dissolved in CH_2Cl_2 (1.5 mL, 2M). Then equimolecular amount of 2-*tert* butyl aniline (0.48 mL, 3.0 mmol, 1.0 equiv) was added and reaction mixture was stirred for 6 h at 25 °C. Then the reaction mixture was purified by flash column chromatography (Pet. ether- EtOAc: 50:50) to afford (2-((2-(*tert*-butyl)phenyl)carbamoyl)-6-methylbenzoic acid **1a′** as a white solid (112 mg, 12% yield).

R_f (Pet. ether /EtOAc = 30/70): 0.30.

^{1}H NMR (400 MHz, DMSO-*d_6*) δ 13.10 (bs, 1H), 9.40 (s, 1H), 7.81 (d, *J* = 7.8 Hz, 1H), 7.75-7.73 (m. 1H), 7.51 (d, *J* = 7.0 Hz, 1H), 7.45-7.40 (m, 2H), 7.26-7.20 (m, 2H), 2.44 (s, 3H), 1.38 (s, 9H). **^{13}C NMR (100 MHz, DMSO-*d_6*)** δ 168.4, 167.5, 146.1, 139.1, 136.6, 135.4, 134.1, 130.8, 129.3, 128.2, 127.4, 126.6, 126.4, 126.1, 34.9, 31.2, 19.4. **HRMS (ESI)** calculated $[M+Na]^+$ for $C_{19}H_{21}NO_3Na$: 334.1419, found: 334.1416. **FTIR (cm^{-1})** 3174, 2957, 2873, 1697, 1655, 1592, 1509, 1441, 1294, 1182 1155.

2-((2-(*tert*-Butyl)-4-methoxyphenyl)carbamoyl)-6-methylbenzoic acid (1b)

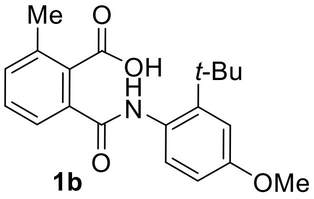

1b

Following the general procedure, 4-methylisobenzofuran-1,3-dione (162.1 mg, 1.0 mmol, 1.0 equiv) was dissolved in CH_2Cl_2 (0.5 mL, 2M). Then equimolecular amount of 2-(*tert*-butyl)-4-methoxyaniline (179.3 mg, 1.0 mmol, 1.0 equiv) was added and reaction mixture was stirred for 12 h at 25 °C. Then the reaction mixture was purified by flash column chromatography (Pet. ether- EtOAc: 50:50) to afford 2-((2-(*tert*-butyl)-4-methoxyphenyl)carbamoyl)-6-methylbenzoic acid **1b** as a white solid (133.1 mg, 39% yield).

R_f (Pet. ether /EtOAc = 30/70): 0.22.

^{1}H NMR (400 MHz, DMSO- *d_6* δ 13.00 (bs, 1H), 9.72 (bs, 1H), 7.54 (d, *J* = 7.2 Hz, 1H), 7.48-7.40 (m, 2H), 7.03 (d, *J* = 8.5 Hz, 1H), 6.92 (d, *J* = 2.6 Hz, 1H), 6.84 (dd, J_1 = 8.6 Hz, J_2 = 2.6 Hz, 1H), 3.77 (s, 3H), 2.37 (s, 3H), 1.37 (s, 9H). **^{13}C NMR (100 MHz, DMSO- *d_6*)** δ 169.8, 167.8, 157.9, 148.4, 135.4, 135.1, 134.4, 132.8, 132.0, 128.9, 128.7, 124.3, 112.8, 110.8, 55.1, 34.9, 30.6, 19.2. **HRMS (ESI)** calculated $[M+Na]^+$ for $C_{20}H_{23}NO_4Na$: 364.1519, found: 364.1521. **FTIR (cm^{-1})** 3255, 2960, 2872, 1711, 1652, 1510, 1288, 1263, 1047, 807.

2-((4-(Benzyloxy)-2-(*tert*-butyl)phenyl)carbamoyl)-6-methylbenzoic acid (1c)

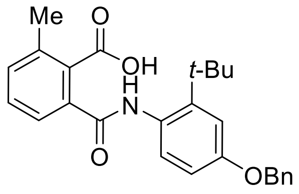

1c

Following the general procedure, 4-methylisobenzofuran-1,3-dione (486.4 mg, 3.0 mmol, 1.0 equiv) was dissolved in CH_2Cl_2 (1.5 mL, 2M). Then equimolecular amount of 4-(benzyloxy)-2-(*tert*-butyl)aniline (0.76 mL, 3.0 mmol, 1.0 equiv) was added and reaction mixture was stirred for 6 h at 25 °C. Then the reaction mixture was purified by flash column chromatography (Pet. ether- EtOAc: 50:50) to afford 2-((4-(benzyloxy)-2-(*tert*-butyl)phenyl)carbamoyl)-6-methylbenzoic acid **1c** as a white solid (642 mg, 51% yield).

R_f (Pet. ether /EtOAc = 30/70): 0.21.

^{1}H NMR (400 MHz, DMSO- d_6) δ 12.94 (bs, 1H), 9.70 (bs, 1H), 7.53-7.32 (m, 8H), 7.02-6.90 (m, 3H), 5.11 (s, 2H), 2.36 (s, 3H), 1.34 (s, 9H). **^{13}C NMR (100 MHz, DMSO- d_6)** δ 169.7, 167.7, 156.9, 148.4, 137.2, 135.4, 135.1, 134.2, 132.7, 132.0, 129.0, 128.7, 128.4, 127.8, 127.7, 124.2, 113.6, 111.8, 69.3, 34.9, 30.6, 19.2. **HRMS (ESI)** calculated $[M+H]^+$ for $C_{26}H_{28}NO_4$: 418.2013, found: 418.2018. **FTIR (cm^{-1})** 3250, 2962, 2870, 1712, 1657, 1507, 1457, 1290, 1217, 738.

2-((4-Bromo-2-(*tert*-butyl)phenyl)carbamoyl)-6-methylbenzoic acid (1d)

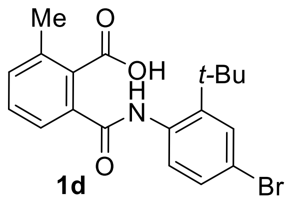

1d

Following the general procedure, 4-methylisobenzofuran-1,3-dione (162.1 mg, 1.0 mmol, 1.0 equiv) was dissolved in CH_2Cl_2 (0.5 mL, 2M). Then equimolecular amount of 4-bromo-2-(*tert*-butyl)aniline (228.1 mg, 1.0 mmol, 1.0 equiv) was added and reaction mixture was stirred for 12 h at 25 °C. Then the reaction mixture was purified by flash column chromatography (Pet. ether- EtOAc: 50:50) to afford 2-((4-bromo-2-(*tert*-butyl)phenyl)carbamoyl)-6-methylbenzoic acid **1d** as a white solid (179.5 mg, 46% yield).

R_f (Pet. ether /EtOAc = 30/70): 0.30.

^{1}H NMR (400 MHz, DMSO- d_6) δ 13.06 (bs, 1H), 9.91 (bs, 1H), 7.54-7.38 (m, 5H), 7.08 (d, J = 8.2 Hz, 1H), 2.38 (s, 3H), 1.37 (s, 9H). **^{13}C NMR (100 MHz, DMSO- d_6)** δ 169.7, 167.8, 149.8, 135.8, 135.3, 135.1, 134.2, 134.0, 132.3, 129.6, 129.4, 128.9, 124.3, 120.3, 35.1, 30.5, 19.3. **HRMS (ESI)** calculated $[M+H]^+$ for $C_{19}H_{21}BrNO_3$: 390.0699, found: 390.0706. **FTIR (cm^{-1})** 3270, 2963, 2872, 1715, 1653, 1586, 1562, 1511, 1443, 1297, 1227, 1020.

2-((2-(*tert*-Butyl)-4-chlorophenyl)carbamoyl)-6-methylbenzoic acid (1e)

Following the general procedure, 4-methylisobenzofuran-1,3-dione (162.1 mg, 1.0 mmol, 1.0 equiv) was dissolved in CH_2Cl_2 (0.5 mL, 2 M). Then equimolecular amount of 2-(*tert*-butyl)-4-chloroaniline (183.7 mg, 1.0 mmol, 1.0 equiv) was added and reaction mixture was stirred for 12 h at 25 °C. Then the reaction mixture was purified by flash column chromatography (Pet. ether- EtOAc: 50:50) to afford 2-((4-bromo-2-(*tert*-butyl)phenyl)carbamoyl)-6-methylbenzoic acid **1e** as a white solid (179.0 mg, 52% yield).

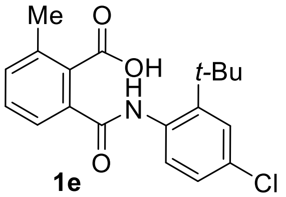

1e

R$_f$ (Pet. ether /EtOAc = 30/70): 0.31.

^{1}H NMR (400 MHz, DMSO- *d*$_6$) δ 13.06 (bs, 1H), 9.92 (s, 1H), 7.53 (d, *J* = 7.2 Hz, 1H), 7.47 (t, *J* = 7.5 Hz, 1H), 7.44-7.40 (m, 2H), 7.37-7.35 (m, 1H), 7.13 (d, *J* = 8.3 Hz, 1H), 2.37 (s, 3H), 1.37 (s, 9H). **^{13}C NMR (100 MHz, DMSO- *d*$_6$)** δ 169.7, 167.8, 149.5, 135.3, 135.3, 135.1, 134.2, 133.7, 132.3, 131.6, 128.9, 126.7, 126.4, 124.3, 35.2, 30.5, 19.3. **HRMS (ESI)** calculated [M+Na]$^+$ for $C_{19}H_{20}ClNO_3Na$: 368.1024, found: 368.1028. **FTIR (cm^{-1})** 3254, 2964, 2876, 1692, 1627, 1510, 1442, 1301, 1243, 1111, 936, 857.

2-((2-(*tert*-Butyl)-4-iodophenyl)carbamoyl)-6-methylbenzoic acid (1f)

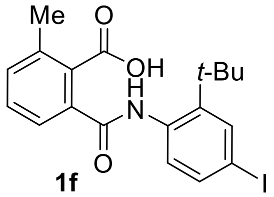

1f

Following the general procedure, 4-methylisobenzofuran-1,3-dione (162.1 mg mg, 3.0 mmol, 1.0 equiv) was dissolved in CH_2Cl_2 (0.5 mL, 2M). Then equimolecular amount of 2-(*tert*-butyl)-4-iodoaniline (275.1 mg, 1.0 mmol, 1.0 equiv) was added and reaction mixture was stirred for 6 h at 25 °C. Then the reaction mixture was purified by flash column chromatography (Pet. ether- EtOAc: 50:50) to afford 2-((2-(*tert*-butyl)-4-iodophenyl)carbamoyl)-6-methylbenzoic acid **1f** as a white solid (228 mg, 52% yield).

R$_f$ (Pet. ether /EtOAc = 30/70): 0.4.

^{1}H NMR (400 MHz, DMSO- *d*$_6$) δ 13.04 (bs, 1H), 9.89 (bs, 1H), 7.69 (d, *J* = 1.9 Hz, 1H), 7.63 (dd, J_1 = 8.1 Hz, J_2 = 1.7 Hz, 1H), 7.52-7.41 (m, 3H), 6.90 (d, *J* = 8.2 Hz, 1H), 2.36 (s, 3H), 1.35 (s, 9H). **^{13}C NMR (100 MHz, DMSO- *d*$_6$)** δ 169.7, 167.7, 149.8, 136.3, 135.5, 135.4, 135.2, 135.1, 134.3, 134.1, 132.3, 128.9, 124.3, 93.5, 34.9, 30.5, 19.3. **HRMS (ESI)** calculated [M+Na]$^+$ for $C_{19}H_{20}INO_3Na$: 460.0380, found: 460.0388. **FTIR (cm^{-1})** 3468, 3234, 2961, 2871, 1698, 1665, 1501, 1387, 1290, 764.

2-((3-(*tert*-Butyl)-[1,1'-biphenyl]-4-yl)carbamoyl)-6-methylbenzoic acid (1g)

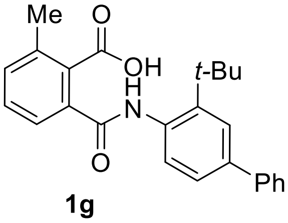

1g

Following the general procedure, 4-methylisobenzofuran-1,3-dione (162.1 mg, 1.0 mmol, 1.0 equiv) was dissolved in CH_2Cl_2 (0.5 mL, 2M). Then equimolecular amount of 3-(*tert*-butyl)-[1,1'-biphenyl]-4-amine (225.3 mg, 1.0 mmol, 1.0 equiv) was added and reaction mixture was stirred for 12 h at 25 °C. Then the reaction mixture was purified by flash column chromatography (Pet. ether- EtOAc: 50:50) to afford 2-((3-(*tert*-butyl)-[1,1'-biphenyl]-4-yl)carbamoyl)-6-methylbenzoic acid **1g** as a white solid (135.6 mg, 35% yield).

R_f (Pet. ether /EtOAc = 30/70): 0.24.

^{1}H NMR (400 MHz, DMSO- *d_6*) δ 13.05 (bs, 1H), 9.91 (bs, 1H), 7.69-7.67 (m, 2H), 7.64 (d, *J* = 2.0 Hz, 1H), 7.57-7.47 (m, 5H), 7.44-7.36 (m, 2H), 7.22 (d, *J* = 8.1 Hz, 1H), 2.38 (s, 3H), 1.44 (s, 9H). **^{13}C NMR (100 MHz, DMSO- *d_6*)** δ 169.8, 167.8, 147.4, 140.4, 139.0, 135.8, 135.4, 135.2, 134.3, 132.4, 132.2, 129.0, 128.9, 127.4, 126.9, 125.2, 124.8, 124.3, 35.1, 30.8, 19.3. **HRMS (ESI)** calculated $[M+Na]^+$ for $C_{25}H_{25}NO_3Na$: 410.1727, found: 410.1732. **FTIR (cm^{-1})** 3427, 2961, 2869, 1712, 1663, 1515, 1487, 1298, 1055, 1025, 764.

2-((2-(*tert*-Butyl)-4-(ethoxycarbonyl)phenyl)carbamoyl)-6-methylbenzoic acid (1h)

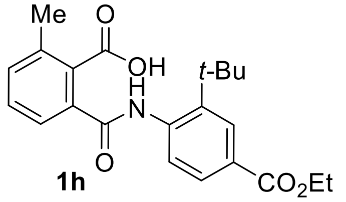

Following the general procedure, 4-methylisobenzofuran-1,3-dione (162.1 mg, 1.0 mmol, 1.0 equiv) was dissolved in CH_2Cl_2 (0.5 mL, 2M). Then equimolecular amount of ethyl 4-amino-3-(*tert*-butyl)benzoate (221.3 mg, 1.0 mmol, 1.0 equiv) was added and reaction mixture was stirred for 12 h at 25 °C. Then the reaction mixture was purified by flash column chromatography (Pet. ether- EtOAc: 50:50) to afford 2-((2-(*tert*-butyl)-4-(ethoxycarbonyl)phenyl)carbamoyl)-6-methylbenzoic acid **1h** as a white solid (149.7 mg, 39% yield).

R_f (Pet. ether /EtOAc = 30/70): 0.22.

^{1}H NMR (400 MHz, DMSO- *d_6*) δ 13.08 (bs, 1H), 10.01 (s, 1H), 8.05 (s, 1H), 7.85 (d, *J* = 8.1 Hz, 1H), 7.56 (d, *J* = 7.3 Hz, 1H), 7.50-7.43 (m, 2H), 7.29 (d, *J* = 8.1 Hz, 1H), 4.33 (q, *J* = 7.0 Hz, 2H), 2.37 (s, 3H), 1.41 (s, 9H), 1.33 (t, *J* = 7.1 Hz, 3H). **^{13}C NMR (100 MHz, DMSO-*d_6*)** δ 169.7, 167.6, 165.6, 147.2, 141.1, 135.3, 134.9, 134.3, 132.3, 132.1, 128.8, 128.4, 127.7, 127.2, 124.4, 60.8, 35.0, 30.6, 19.3, 14.2. **HRMS (ESI)** calculated $[M+H]^+$ for $C_{22}H_{25}NO_5Na$: 406.1625, found: 406.1627. **FTIR (cm^{-1})** 3251, 2975, 2869, 1709, 1652, 1587, 1515, 1445, 1303, 1054, 1025.

2-((2-(*tert*-Butyl)-4-(thiophen-3-yl)phenyl)carbamoyl)-6-methylbenzoic acid (1i)

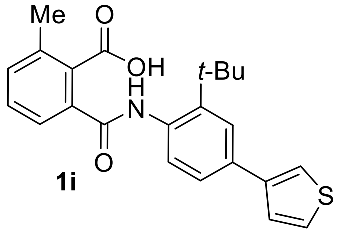

1i

Following the general procedure, 4-methylisobenzofuran-1,3-dione (162.1 mg, 1.0 mmol, 1.0 equiv) was dissolved in CH_2Cl_2 (0.5 mL, 2M). Then equimolecular amount of 2-(*tert*-butyl)-4-(thiophen-3-yl)aniline (231.3 mg, 1.0 mmol, 1.0 equiv) was added and reaction mixture was stirred for 6 h at 25 °C. Then the reaction mixture was purified by flash column chromatography (Pet. ether- EtOAc: 50:50) to afford 2-((2-(*tert*-butyl)-4-(thiophen-3-yl)phenyl)carbamoyl)-6-methylbenzoic acid **1i** as a white solid (185 mg, 47% yield).

$\boldsymbol{R_f}$ (Pet. ether /EtOAc = 30/70): 0.30

^{1}H NMR (400 MHz, DMSO- *d*$_6$) δ 13.02 (bs, 1H), 9.87 (bs, 1H), 7.88-7.87 (m, 1H), 7.69 (d, J = 1.9 Hz, 1H), 7.66-7.64 (m, 1H), 7.59-7.55 (m, 3H), 7.49-7.42 (m, 2H), 7.16 (d, J = 8.1 Hz, 1H), 2.38 (s, 3H), 1.43 (s, 9H). **^{13}C NMR (100 MHz, DMSO- *d*$_6$)** δ 169.7, 167.7, 147.3, 141.5, 135.3, 135.3, 135.2, 134.3, 134.0, 132.3, 132.1, 128.8, 127.1, 126.4, 124.5, 124.3, 124.2, 121.0, 35.0 30.8, 19.2. **HRMS (ESI)** calculated $[M+Na]^+$ for $C_{23}H_{23}NO_3SNa$: 416.1291, found: 416.1298. **FTIR (cm^{-1})** 3288, 2967, 2868, 1711, 1659, 1504, 1300, 1055, 1010, 778.

(*E*)-2-((2-(*tert*-Butyl)-4-styrylphenyl)carbamoyl)-6-methylbenzoic acid (1j)

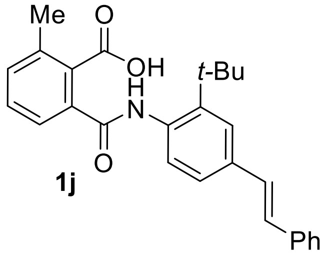

1j

Following the general procedure, 4-methylisobenzofuran-1,3-dione (162.1 mg, 1.0 mmol, 1.0 equiv) was dissolved in CH_2Cl_2 (0.5 mL, 2 M). Then equimolecular amount of (*E*)-2-(*tert*-butyl)-4-styrylaniline (251.3 mg, 1.0 mmol, 1.0 equiv) was added and reaction mixture was stirred for 12 h at 25 °C. Then the reaction mixture was purified by flash column chromatography (Pet. ether-EtOAc: 50:50) to afford (*E*)-2-((2-(*tert*-butyl)-4-styrylphenyl)carbamoyl)-6-methylbenzoic acid **1j** as a white solid (194.3 mg, 47% yield).

$\boldsymbol{R_f}$ (Pet. ether /EtOAc = 30/70): 0.24.

^{1}H NMR (400 MHz, DMSO- *d*$_6$) δ 13.03 (bs, 1H), 9.85 (bs, 1H), 7.64-7.60 (m, 3H), 7.56-7.54 (m, 2H), 7.49-7.37 (m, 4H), 7.34-7.22 (m, 3H), 7.13 (d, J = 8.2 Hz, 1H), 2.37 (s, 3H), 1.42 (s, 9H). **^{13}C NMR (100 MHz, DMSO- *d*$_6$)** δ 169.6, 167.6, 147.1, 137.1, 135.6, 135.5, 135.1, 135.1, 132.1, 132.1, 128.8, 128.7, 128.4, 128.3, 127.6, 126.4, 125.6, 124.2, 123.7, 34.9, 30.8, 19.2. **HRMS (ESI)** calculated $[M+H]^+$ for $C_{27}H_{28}NO_3$: 414.2064, found: 414.2068. **FTIR (cm^{-1})** 3381, 2961, 2929, 2867, 1712, 1664, 1510, 1298, 1054, 1025, 1010, 762.

(*E*)-2-((2-(*tert*-Butyl)-4-(3-methoxy-3-oxoprop-1-en-1-yl)phenyl)carbamoyl)-6-methylbenzoic acid (1k)

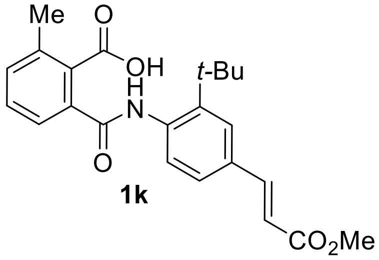

Following the general procedure, 4-methylisobenzofuran-1,3-dione (162.1 mg, 1.0 mmol, 1.0 equiv) was dissolved in CH_2Cl_2 (0.5 mL, 2 M). Then equimolecular amount of (*E*)-2-(*tert*-butyl)-4-styrylaniline (251.3 mg, 1.0 mmol, 1.0 equiv) was added and reaction mixture was stirred for 12 h at 25 °C. Then the reaction mixture was purified by flash column chromatography (Pet. ether- EtOAc: 50:50) to afford (*E*)-2-((2-(*tert*-butyl)-4-(3-methoxy-3-oxoprop-1-en-1-yl)phenyl)carbamoyl)-6-methylbenzoic acid **1k** as a white solid (221.45 mg, 56% yield).

R_f (Pet. ether /EtOAc = 30/70): 0.24.

^{1}H NMR (400 MHz, DMSO- *d_6*) δ 13.06 (bs, 1H), 9.92 (s, 1H), 7.74-7.66 (m, 3H), 7.49-7.42 (m, 3H), 7.18 (d, *J* = 8.0 Hz, 1H), 6.66 (d, *J* = 16.0 Hz, 1H), 3.74 (s, 3H), 2.37 (s, 3H), 1.40 (s, 9H). **^{13}C NMR (100 MHz, DMSO- *d_6*)** δ 169.7, 167.8, 166.8, 147.5, 144.6, 138.4, 135.3, 135.3, 134.2, 132.7, 132.4, 132.2, 128.9, 128.0, 125.6, 124.3, 117.8, 51.5, 35.1, 30.8, 19.3. **HRMS (ESI)** calculated $[M+H]^+$ for $C_{23}H_{25}NO_5Na$: 418.1625, found: 418.1631. **FTIR (cm^{-1})** 3373, 2952, 2930, 2870, 1725, 1712, 1660, 1570, 1298, 1055, 1027, 762.

2-((2-(*tert*-Butyl)-4-(phenylethynyl)phenyl)carbamoyl)-6-methylbenzoic acid (1l)

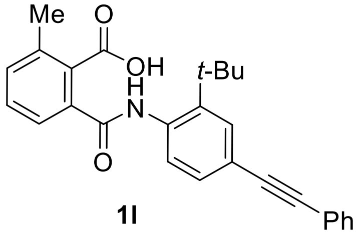

1l

Following the general procedure, 4-methylisobenzofuran-1,3-dione (162.1 mg, 1.0 mmol, 1.0 equiv) was dissolved in CH_2Cl_2 (0.5 mL, 2M). Then equimolecular amount of 2-(*tert*-butyl)-4-(phenylethynyl)aniline (249.3 mg, 1.0 mmol, 1.0 equiv) was added and reaction mixture was stirred for 6 h at 25 °C. Then the reaction mixture was purified by flash column chromatography (Pet. ether- EtOAc: 50:50) to afford 2-((2-(*tert*-butyl)-4-(phenylethynyl)phenyl)carbamoyl)-6-methylbenzoic acid **1l** as a white solid (230 mg, 56% yield).

R_f (Pet. ether /EtOAc = 30/70): 0.38.

^{1}H NMR (400 MHz, DMSO- *d_6*) δ 13.07 (bs, 1H), 9.94 (bs, 1H), 7.60-7.53 (m, 4H), 7.50-7.53 (m, 6H), 7.18 (d, *J* = 8.0 Hz, 1H), 2.38 (s, 3H), 1.40 (s, 9H). **^{13}C NMR (100 MHz, DMSO- *d_6*)** δ 169.8, 167.8, 147.6, 137.0, 135.4, 135.2, 135.2, 134.3, 132.4, 132.3, 131.5, 130.0, 129.5, 129.0, 128.9, 124.4, 122.4, 121.1, 89.6, 89.3, 35.1, 30.7, 19.4. **HRMS (ESI)** calculated

$[M+Na]^+$ for $C_{27}H_{25}NO_3Na$: 434.1727, found: 434.1731. **FTIR (cm^{-1})** 3702, 3657, 2970, 2868, 1711, 1658, 1504, 1299, 1055, 1024, 759.

2-((2-(*tert*-Butyl)-5-nitrophenyl)carbamoyl)-6-methylbenzoic acid (1m)

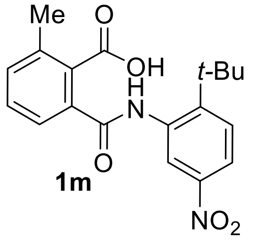

1m

Following the general procedure, 4-methylisobenzofuran-1,3-dione (162.1 mg, 1.0 mmol, 1.0 equiv) was dissolved in CH_2Cl_2 (0.5 mL, 2M). Then equimolecular amount of 2-(*tert*-butyl)-5-nitroaniline (194.2 mg, 1.0 equiv) was added and reaction mixture was stirred for 6 h at 25 °C. Then the reaction mixture was purified by flash column chromatography (Pet. ether- EtOAc: 50:50) to afford 2-((2-(*tert*-butyl)-5-nitrophenyl)carbamoyl)-6-methylbenzoic acid **1m** as a white solid (163.9 mg, 46% yield).

R

$\boldsymbol{R_f}$ (Pet. ether /EtOAc = 30/70): 0.20.

^{1}H NMR (400 MHz, DMSO- d_6) δ 13.24 (bs, 1H), 10.21 (s, 1H), 8.14-8.12 (m, 1H), 7.99 (s, 1H), 7.74 (d, *J* = 7.4 Hz, 1H), 7.54-7.46 (m, 3H), 2.39 (s, 3H), 1.42 (s, 9H). **^{13}C NMR (100 MHz, DMSO- d_6)** δ 169.8, 168.2, 155.2, 145.8, 137.5, 135.5, 135.0, 134.2, 132.5, 129.0, 128.6, 126.3, 124.4, 121.8, 35.8, 30.4, 19.5. **HRMS (ESI)** calculated $[M+Na]^+$ for $C_{19}H_{20}N_2O_5Na$: 379.1264, found: 379.1271. **FTIR (cm^{-1})** 3247, 2962, 2877, 1723, 1667, 1532, 1444, 1353, 1298, 1260, 1140.

2-Methyl-6-((4-methyl-2-(2-phenylpropan-2-yl)phenyl)carbamoyl)benzoic acid (1n)

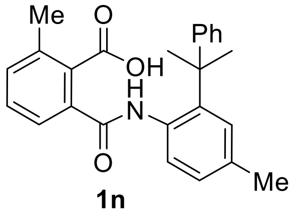

1n

Following the general procedure, 4-methylisobenzofuran-1,3-dione (162.1 mg, 1.0 mmol, 1.0 equiv) was dissolved in CH_2Cl_2 (0.5 mL, 2 M). Then equimolecular amount of 4-methyl-2-(2-phenylpropan-2-yl)aniline (225.3 mg, 1.0 mmol, 1.0 equiv) was added and reaction mixture was stirred for 12 h at 25 °C. Then the reaction mixture was purified by flash column chromatography (Pet. ether- EtOAc: 50:50) to afford 2-methyl-6-((4-methyl-2-(2-phenylpropan-2-yl)phenyl)carbamoyl)benzoic acid **1n** as a white solid (178.2 mg, 46% yield).

$\boldsymbol{R_f}$ (Pet. ether /EtOAc = 30/70): 0.25.

^{1}H NMR (400 MHz, DMSO- d_6) δ 12.94 (bs, 1H), 7.59 (s, 1H), 7.48-7.46 (m, 1H), 7.42-7.41 (m, 1H), 7.33 (d, *J* = 7.6 Hz, 1H), 7.29-7.25 (m, 2H), 7.22-7.18 (m, 4H), 7.12 (d, *J* = 7.7 Hz, 1H), 6.39 (d, *J* = 7.5 Hz, 1H), 2.37 (s, 3H), 2.29 (s, 3H), 1.61 (s, 6H). **^{13}C NMR (100 MHz, DMSO- d_6)** δ 169.4, 165.3, 148.9, 141.4, 135.0, 134.6, 134.3, 134.1, 132.6, 132.3, 128.9, 128.5, 127.3, 127.2, 126.6, 126.2, 125.5, 123.3, 42.3, 30.2, 21.0, 19.0. **HRMS (ESI)** calculated

$[M+Na]^+$ for $C_{25}H_{25}NO_3Na$: 410.1727, found: 410.1731. **FTIR (cm^{-1})** 3388, 2969, 2926, 1713, 1677, 1589, 1514, 1451, 1301, 1268, 1025.

2-((2-(1-Methoxy-2-methylpropan-2-yl)phenyl)carbamoyl)-6-methylbenzoic acid (1o)

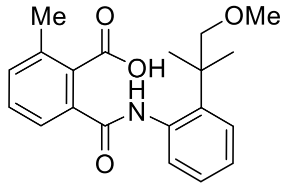

1o

Following the general procedure, 4-methylisobenzofuran-1,3-dione (162.1 mg, 1.0 mmol, 1.0 equiv) was dissolved in CH_2Cl_2 (0.5 mL, 2 M). Then equimolecular amount of 2-(1-methoxy-2-methylpropan-2-yl)aniline (190.0 mg, 1.0 mmol, 1.0 equiv) was added and reaction mixture was stirred for 12 h at 25 °C. Then the reaction mixture was purified by flash column chromatography (Pet. ether- EtOAc: 50:50) to afford 2-((2-(1-methoxy-2-methylpropan-2-yl)phenyl)carbamoyl)-6-methylbenzoic acid **1o** as a white solid (197 mg, 58% yield).

$\boldsymbol{R_f}$ (Pet. ether /EtOAc = 30/70): 0.20.

^{1}H NMR (400 MHz, DMSO- *d*$_6$) δ 13.10 (bs, 1H), 9.98 (s, 1H), 8.04-8.02 (m, 1H), 7.79 (d, *J* = 7.4 Hz, 1H), 7.53 (d, *J* = 7.5 Hz, 1H), 7.44 (t, *J* = 8.0 Hz, 1H), 7.38-7.36 (m, 1H), 7.28-7.24 (m, 1H), 7.15-7.11 (m, 1H), 3.38 (s, 2H), 3.00 (s, 3H), 2.38 (s, 3H), 1.34 (s, 6H). **^{13}C NMR (100 MHz, DMSO- *d*$_6$)** δ 167.4, 166.8, 139.4, 138.9, 137.2, 135.1, 134.2, 129.0, 128.6, 127.5, 127.4, 126.6, 125.9, 124.9, 82.2, 58.2, 39.2, 26.2, 18.9. **HRMS (ESI)** calculated $[M+Na]^+$ for $C_{20}H_{23}NO_4Na$: 364.1519, found: 364.1523. **FTIR (cm^{-1})** 3370, 2975, 2920, 1714, 1678, 1592, 1510, 1425, 1312, 1208, 1020.

2-((2-(Methoxydiphenylmethyl)phenyl)carbamoyl)-6-methylbenzoic acid (1p)

Following the general procedure, 4-methylisobenzofuran-1,3-dione (324.2 mg , 2.0 mmol, 1.0 equiv) was dissolved in CH_2Cl_2 (1.0 mL, 2 M). Then equimolecular amount of 2-(methoxydiphenylmethyl)aniline (578 mg, 2.0 mmol, 1.0 equiv) was added and reaction mixture was stirred for 24 h at 25 °C. Then the reaction mixture was purified by flash column chromatography (Pet. ether- EtOAc: 50:50) to afford 2-((2-(methoxydiphenylmethyl)phenyl)carbamoyl)-6-methylbenzoic acid **1p** as a white solid (325.6 mg, 36% yield).

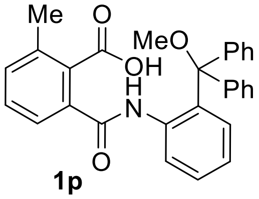

1p

$\boldsymbol{R_f}$ (Pet. ether /EtOAc = 30/70): 0.22.

^{1}H NMR (400 MHz, CDCl$_3$) δ 13.07 (s, 1H), 9.11 (s, 1H), 8.32 (d, *J* = 7.7 Hz, 1H), 7.46 (t, *J* = 7.6 Hz, 1H), 7.37 – 7.24 (m, 12H), 7.22 – 7.15 (m, 2H), 6.28 (d, *J* = 7.5 Hz, 1H), 3.14 (s, 3H), 2.31 (s, 3H). **^{13}C NMR (100 MHz, CDCl$_3$)** δ 169.4, 164.8, 142.8, 137.5, 135.3, 134.2, 134.1,

132.7, 130.7, 129.0, 128.7, 128.3, 127.3, 127.2, 123.4, 122.8, 122.2, 88.0, 53.1, 19.1. **HRMS (ESI)** calculated $[M+Na]^+$ for $C_{29}H_{25}NO_4Na$: 474.1676, found: 474.1684. **FTIR (cm^{-1})** 3333, 3162, 3061, 1742, 1660, 1580, 1516, 1436, 1229, 1054.

2-((2-(Di(thiophen-2-yl)((trimethylsilyl)oxy)methyl)phenyl)carbamoyl)-6-methylbenzoic acid (1q)

Following the general procedure, 4-methylisobenzofuran-1,3-dione (324.2 mg, 2.0 mmol, 1.0 equiv) was dissolved in CH_2Cl_2 (1.0 mL, 2 M). Then equimolecular amount of 2-(di(thiophen-2-yl)((trimethylsilyl)oxy)methyl)aniline (719.1 mg, 2.0 mmol, 1.0 equiv) was added and reaction mixture was stirred for 24 h at 25 °C. Then the reaction mixture was purified by flash column chromatography (Pet. ether- EtOAc: 50:50) to afford 2-((2-(di(thiophen-2-yl)((trimethylsilyl)oxy)methyl)phenyl)carbamoyl)-6-methylbenzoic acid **1q** as a white solid (522.3 mg, 50% yield).

R$_f$ (Pet. ether /EtOAc = 30/70): 0.28.

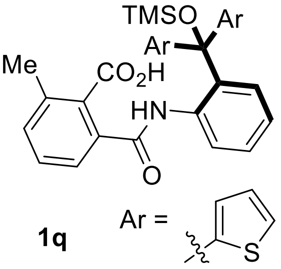

1q

^{1}H NMR (400 MHz, CDCl$_3$) δ 13.07 (s, 1H), 9.80 (s, 1H), 8.57 (d, J = 8.2 Hz, 1H), 7.77 (d, J = 7.1 Hz, 1H), 7.58 (d, J = 5.0 Hz, 2H), 7.46-7.37 (m, 3H), 7.06-6.97 (m, 4H), 6.74 (s, 2H), 1.81 (s, 3H), -0.35 (s, 9H). **^{13}C NMR (100 MHz, CDCl$_3$)** δ 166.9, 166.2, 149.5, 138.5, 137.7, 134.9, 134.1, 133.4, 128.8, 128.6, 128.4, 127.8, 127.5, 126.9, 122.2, 120.7, 81.8, 17.7, 0.7. **HRMS (ESI)** calculated $[M+Na]^+$ for $C_{27}H_{27}NO_4S_2SiNa$: 544.1043, found: 544.1049. **FTIR (cm^{-1})** 3306, 3068, 2957, 1687, 1583, 1526, 1434, 1299, 1252, 1033.

2-Methyl-6-((2-(phenylsulfonyl)phenyl)carbamoyl)benzoic acid (1r)

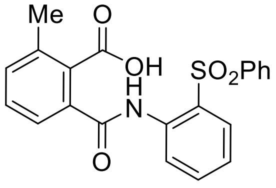

1r

Following the general procedure, 4-methylisobenzofuran-1,3-dione (324.2 mg, 2.0 mmol, 1.0 equiv) was dissolved in AcOH (1.0 mL, 2.0 M). Then equimolecular amount of 2-(phenylsulfonyl)aniline (446.0 mg, 2.0 mmol, 1.0 equiv) was added and reaction mixture was stirred for 6 h at 25 °C. Then the reaction mixture was purified by flash column chromatography (Pet. ether- EtOAc: 50:50) to afford 2-methyl-6-((2-(phenylsulfonyl)phenyl)carbamoyl)benzoic acid **1r** as a white solid (181.2 mg, 22% yield).

R$_f$ (Pet. ether /EtOAc = 30/70): 0.22.

^{1}H NMR (400 MHz, DMSO- *d$_6$*) δ 13.29 (s, 1H), 9.78 (s, 1H), 8.53 (d, J = 7.7 Hz, 1H), 8.03 (d, J = 7.6 Hz, 1H), 7.90-7.71 (m, 5H), 7.62 – 7.50 (m, 4H), 7.39 (t, J = 7.3 Hz, 1H), 2.20 (s,

3H). **^{13}C NMR (100 MHz, DMSO- *d*$_6$)** δ 167.4, 166.9, 140.6, 137.9, 137.0, 135.4, 135.1, 134.6, 134.2, 130.0, 129.9, 129.4, 128.4, 128.0, 127.9, 126.7, 124.9, 123.1, 18.4. **HRMS (ESI)** calculated $[M+Na]^+$ for $C_{21}H_{18}NO_5S$: 396.0900, found: 396.0901. **FTIR (cm^{-1})** 3444, 2959, 2869, 1701, 1684, 1574, 1502, 1369, 1291, 1250, 760.

2-((2-(*tert*-Butyl)phenyl)carbamoyl)-6-methoxybenzoic acid (1s)

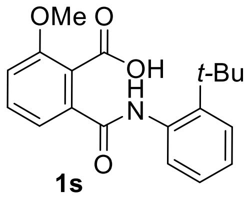

1s

Following the general procedure, 4-methoxyisobenzofuran-1,3-dione (178.1 mg, 1.0 mmol, 1.0 equiv) was dissolved in CH_2Cl_2 (0.5 mL, 2 M). Then equimolecular amount of 2-(*tert*-butyl)aniline (149.2 mg, 0.15 mL, 1.0 mmol, 1.0 equiv) was added and reaction mixture was stirred for 12 h at 25 °C. Then the reaction mixture was purified by flash column chromatography (Pet. ether- EtOAc: 50:50) to afford 2-((2-(*tert*-butyl)phenyl)carbamoyl)-6-methoxybenzoic acid **1s** as a white solid (170.2 mg, 52% yield).

R$_f$ (Pet. ether /EtOAc = 30/70): 0.15.

^{1}H NMR (400 MHz, DMSO- *d*$_6$) δ 13.12 (bs, 1H), 9.43 (s, 1H), 7.58-7.56 (m, 1H), 7.51-7.46 (m, 2H), 7.40-7.38 (m, 1H), 7.34-7.32 (m, 1H), 7.23-7.21 (m, 2H), 3.83 (s, 3H), 1.40 (s, 9H). **^{13}C NMR (100 MHz, DMSO- *d*$_6$)** δ 167.3, 166.1, 156.4, 147.0, 136.8, 131.2, 130.4, 129.5, 129.2, 126.7, 126.2, 126.1, 121.5, 115.3, 55.7, 34.8, 30.6. **HRMS (ESI)** calculated $[M+Na]^+$ for $C_{19}H_{21}NO_4Na$: 350.1363, found: 350.1366. **FTIR (cm^{-1})** 3142, 2962, 2871, 1698, 1671, 1586, 1511, 1464, 1394, 1272, 1055, 1024.

2-(Benzyloxy)-6-((2-(*tert*-butyl)phenyl)carbamoyl)benzoic acid (1t)

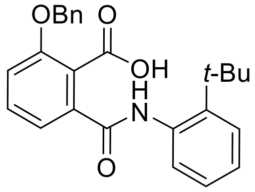

1t

Following the general procedure, 4-(benzyloxy)isobenzofuran-1,3-dione (254.1 mg, 1.0 mmol, 1.0 equiv) was dissolved in CH_2Cl_2 (0.5 mL, 2M). Then equimolecular amount of 2-*tert* butyl aniline (0.16 mL, 1.0 mmol, 1.0 equiv) was added and reaction mixture was stirred for 12 h at 25 °C. Then the reaction mixture was purified by flash column chromatography (Pet. ether- EtOAc: 50:50) to afford 2-(benzyloxy)-6-((2-(*tert*-butyl)phenyl)carbamoyl) benzoic acid **1t** as a white solid (197.7 mg, 49 % yield).

R$_f$ (Pet. ether /EtOAc = 30/70): 0.30.

^{1}H NMR (400 MHz, DMSO- *d*$_6$) δ 12.86 (bs, 1H), 9.87 (s, 1H), 7.53-7.38 (m, 6H), 7.36-7.32 (m, 3H), 7.27-7.25 (m, 2H), 7.11-7.09 (m, 1H), 5.23 (s, 2H), 1.38 (s, 9H). **^{13}C NMR (100 MHz, DMSO- *d*$_6$)** δ 167.8, 166.7, 155.0, 146.9, 136.8, 136.1, 135.6, 131.8, 130.0, 128.4, 127.8, 127.2, 126.7, 126.4, 125.1, 119.0, 115.4, 69.8, 34.9, 30.8. **HRMS (ESI)** calculated $[M+Na]^+$

for $C_{25}H_{25}NO_4Na$: 426.1676, found: 426.1683. **FTIR (cm^{-1})** 3245, 2962, 2870, 1712, 1659, 1584, 1511, 1452, 1276, 754.

2-(Allyloxy)-6-((2-(*tert*-butyl)phenyl)carbamoyl)benzoic acid (1u)

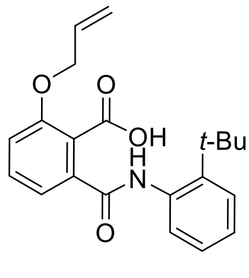

1u

Following the general procedure, 4-(allyloxy)isobenzofuran-1,3-dione (204 mg, 1.0 mmol, 1.0 equiv) was dissolved in CH_2Cl_2 (0.5 mL, 2M). Then equimolecular amount of 2-*tert* butyl aniline (0.48 mL, 3.0 mmol, 1.0 equiv) was added and reaction mixture was stirred for 6 h at 25 °C. Then the reaction mixture was purified by flash column chromatography (Pet. ether- EtOAc: 50:50) to afford 2-(allyloxy)-6-((2-(*tert*-butyl)phenyl)carbamoyl)benzoic acid **1u** as a white solid (166.8 mg, 47 % yield).

$\boldsymbol{R_f}$ (Pet. ether /EtOAc = 30/70):0.25.

^{1}H NMR (400 MHz, DMSO- d_6) δ 12.77 (bs, 1H), 9.85 (s, 1H), 7.50 (t, J = 8.0 Hz, 1H), 7.44-7.42 (m, 1H), 7.32 (d, J = 7.6 Hz, 1H), 7.26-7.24 (m, 3H), 7.09-7.07 (m, 1H), 6.06-5.96 (m, 1H), 5.42 (dq, J_1 = 17.2 Hz, J_2 = 1.7 Hz, 1H), 5.25 (dq, J_1 = 10.6 Hz, J_2 = 1.5 Hz, 1H), 4.66 (t, J = 4.6 Hz, 2H), 1.37 (s, 9H). **^{13}C NMR (100 MHz, DMSO- d_6)** δ 167.9, 166.9, 155.0, 147.0, 136.2, 135.6, 133.3, 131.9, 130.1, 127.3, 126.8, 126.5, 125.1, 119.0, 117.3, 115.3, 68.9, 34.9, 30.9. **HRMS (ESI)** calculated $[M+Na]^+$ for $C_{21}H_{23}NO_4Na$: 376.1519, found: 376.1528. **FTIR (cm^{-1})** 3225, 3082, 2995, 2962, 2869,1700, 1624, 1582, 1350, 760.

2-((2-(*tert*-Butyl)phenyl)carbamoyl)-6-hydroxybenzoic acid (1v)

Following the general procedure, 4-hydroxyisobenzofuran-1,3-dione (227.0 mg , 3.0 mmol, 1.0 equiv) was dissolved in CH_2Cl_2 (1.5 mL, 2 M). Then equimolecular amount of 2-(*tert*-butyl)aniline (447.6 mg, 0.45 mL, 3.0 mmol, 1.0 equiv) was added and reaction mixture was stirred for 24 h at 25 °C. Then the reaction mixture was purified by flash column chromatography (Pet. ether- EtOAc: 50:50) to afford 2-((2-(*tert*-butyl)phenyl)carbamoyl)-6-hydroxybenzoic acid **1v** as a white solid (375.6 mg, 40% yield).

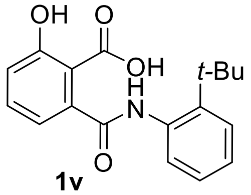

1v

$\boldsymbol{R_f}$ (Pet. ether /EtOAc = 30/70): 0.21.

^{1}H NMR (400 MHz, $CDCl_3$) δ 13.32 (s, 1H), 10.88 (s, 1H), 9.67 (s, 1H), 7.47-7.41 (m, 2H), 7.26-7.18 (m, 3H), 7.04-7.02 (m, 2H), 1.37 (s, 9H). **^{13}C NMR (100 MHz, $CDCl_3$)** δ 169.8, 167.7, 157.7, 146.9, 138.7, 136.3, 132.1, 131.4, 127.1, 126.6, 126.4, 117.9, 117.5, 116.9, 34.9, 30.8. **HRMS (ESI)** calculated $[M+Na]^+$ for $C_{18}H_{19}NO_4Na$: 336.1206, found: 336.1211. **FTIR (cm^{-1})** 3245, 2963, 2875, 1709, 1653, 1588, 1507, 1389, 1297, 1125, 1081.

2-((2-(*tert*-Butyl)phenyl)carbamoyl)-6-chlorobenzoic acid (1w)

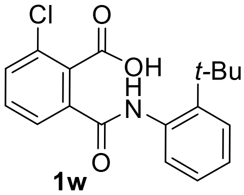

1w

Following the general procedure, 4-chloroisobenzofuran-1,3-dione (182.6 mg, 1.0 mmol, 1.0 equiv) was dissolved in CH_2Cl_2 (0.5 mL, 2 M). Then equimolecular amount of 2-(*tert*-butyl)aniline (0.15 mL, 1.0 mmol, 1.0 equiv) was added and reaction mixture was stirred for 12 h at 25 °C. Then the reaction mixture was purified by flash column chromatography (Pet. ether- EtOAc: 50:50) to afford 2-((2-(*tert*-butyl)phenyl)carbamoyl)-6-chlorobenzoic acid **1w** as a white solid (146.0 mg, 44% yield).

R_f (Pet. ether /EtOAc = 30/70): 0.30.

^{1}H NMR (400 MHz, DMSO- *d_6*) δ 13.44 (bs, 1H), 10.02 (s, 1H), 7.75-7.69 (m, 2H), 7.63-7.59 (m, 1H), 7.47-7.43 (m, 1H), 7.30-7.24 (m, 2H), 7.10-7.08 (m, 1H), 1.37 (s, 9H). **^{13}C NMR (100 MHz, DMSO- *d_6*)** δ 167.0, 166.0, 147.0, 136.3, 135.8, 134.3, 131.8, 131.5, 130.4, 130.3, 127.4, 126.8, 126.5, 125.7, 34.9, 30.9. **HRMS (ESI)** calculated $[M+Na]^+$ for $C_{18}H_{18}ClNO_3Na$: 354.0867, found: 354.0871. **FTIR (cm^{-1})** 3251, 2962, 2875, 1718, 1655, 1581, 1514, 1445, 1298, 1149, 1055. The structure of molecule confirmed by **single crystal XRD**; **CCDC**: 2261807.

2-Bromo-6-((2-(*tert*-butyl)phenyl)carbamoyl)benzoic acid (1x)

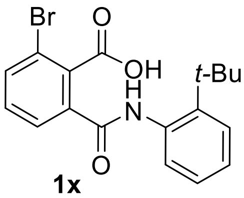

1x

Following the general procedure, 4-bromoisobenzofuran-1,3-dione (227.0 mg mg, 1.0 mmol, 1.0 equiv) was dissolved in CH_2Cl_2 (0.5 mL, 2 M). Then equimolecular amount of 2-(*tert*-butyl)aniline (149.2 mg, 0.15 mL, 1.0 mmol, 1.0 equiv) was added and reaction mixture was stirred for 12 h at 25 °C. Then the reaction mixture was purified by flash column chromatography (Pet. ether- EtOAc: 50:50) to afford 2-bromo-6-((2-(*tert*-butyl)phenyl)carbamoyl)benzoic acid **1x** as a white solid (207.1 mg, 55% yield).

R_f (Pet. ether /EtOAc = 30/70): 0.20.

^{1}H NMR (400 MHz, DMSO- *d_6*) δ 13.41 (bs, 1H), 10.00 (s, 1H), 7.85 (d, *J* = 8.0 Hz, 1H), 7.77 (d, *J* = 7.3 Hz, 1H), 7.53 (t, *J* = 7.8 Hz, 1H), 7.45-7.44 (m, 1H), 7.29-7.25 (m, 2H), 7.09-7.07 (m, 1H), 1.37 (s, 9H). **^{13}C NMR (100 MHz, DMSO- *d_6*)** δ 167.8, 166.1, 147.0, 136.4, 136.3, 135.9, 134.6, 131.8, 130.6, 127.4, 126.8, 126.6, 126.2, 119.3, 34.9, 30.9. **HRMS (ESI)** calculated $[M+Na]^+$ for $C_{18}H_{18}BrNO_3Na$: 398.0362, found: 398.0368. **FTIR (cm^{-1})** 3245, 2963, 2875, 1709, 1653, 1588, 1507, 1389, 1297, 1125, 1081.

2-((2-(*tert*-Butyl)phenyl)carbamoyl)-6-nitrobenzoic acid (1y)

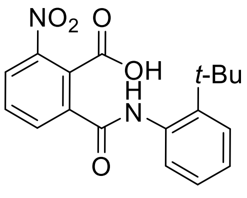

1y

Following the general procedure, 4-nitroisobenzofuran-1,3-dione (193 mg, 3.0 mmol, 1.0 equiv) was dissolved in CH_2Cl_2 (0.5 mL, 2M). Then equimolecular amount of 2-*tert* butyl aniline (0.16 mL, 1.0 mmol, 1.0 equiv) was added and reaction mixture was stirred for 6 h at 25 °C. Then the reaction mixture was purified by flash column chromatography (Pet. ether- EtOAc: 50:50) to afford 2-((2-(*tert*-butyl)phenyl)carbamoyl)-6-nitrobenzoic acid **1y** as a white solid (154 mg, 45 % yield).

R_f (Pet. ether /EtOAc = 30/70): 0.15.

^{1}H NMR (400 MHz, DMSO- *d*$_6$) δ 13.85 (bs, 1H), 10.19 (s, 1H), 8.19 (d, *J* = 8.0 Hz, 1H), 7.99 (d, *J* = 7.5 Hz, 1H), 7.86 (t, *J* = 8.0 Hz, 1H), 7.46-7.44 (m, 1H), 7.29-7.27 (m, 2H), 7.17-7.14 (m, 1H), 1.39 (s, 9H). **^{13}C NMR (100 MHz, DMSO- *d*$_6$)** δ 165.9, 165.9, 147.5, 147.0, 137.4, 135.7, 132.0, 131.6, 130.9, 129.1, 127.5, 126.8, 126.6, 125.4, 34.9, 30.9. **HRMS (ESI)** calculated $[M+Na]^+$ for $C_{18}H_{18}N_2O_5Na$: 365.1108, found: 365.1111. **FTIR (cm^{-1})** 3244, 2961, 2874, 1702, 1589, 1500, 1386, 1288, 1184, 763.

2-((2-(*tert*-Butyl)phenyl)carbamoyl)-1-naphthoic acid (1z)

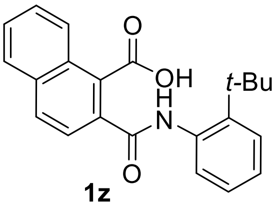

1z

Following the general procedure, naphtho[1,2-c]furan-1,3-dione (396.0 mg, 2.0 mmol, 1.0 equiv) was dissolved in CH_2Cl_2 (1.0 mL, 2 M). Then equimolecular amount of 2-(*tert*-butyl)aniline (298.4 mg, 0.3 mL, 2.0 mmol, 1.0 equiv) was added and reaction mixture was stirred for 12 h at 25 °C. Then the reaction mixture was purified by flash column chromatography (Pet. ether- EtOAc: 50:50) to afford 2-((2-(*tert*-butyl)phenyl)carbamoyl)-1-naphthoic acid **1z** as a white solid (305.44 mg, 44% yield).

R_f (Pet. ether /EtOAc = 30/70): 0.27.

^{1}H NMR (400 MHz, DMSO- *d*$_6$) δ 13.45 (s, 1H), 10.02 (s, 1H), 8.18 (d, *J* = 8.4 Hz, 1H), 8.09 (t, *J* = 8.5 Hz, 2H), 7.85 (d, *J* = 8.4 Hz, 1H), 7.71-7.65 (m, 2H), 7.48-7.45 (m, 1H), 7.30-7.28 (m, 2H), 7.21-7.19 (m, 1H), 1.42 (s, 9H). **^{13}C NMR (100 MHz, DMSO- *d*$_6$)** δ 169.4, 167.4, 147.1, 136.2, 133.5, 132.6, 132.5, 131.9, 129.5, 128.9, 128.3, 127.8, 127.5, 127.3, 126.8, 126.5, 125.6, 123.5, 35.0, 30.9. **HRMS (ESI)** calculated $[M+H]^+$ for $C_{22}H_{22}NO_3$: 348.1594, found: 348.1609. **FTIR (cm^{-1})** 3271, 2959, 2910, 1700, 1633, 1506, 1442, 1282, 1219, 1052, 752.

6-((2-(*tert*-Butyl)phenyl)carbamoyl)-2-methoxy-3-nitrobenzoic acid (1aa)

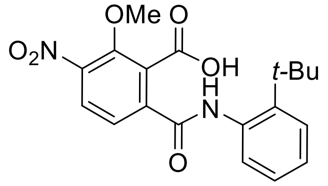

1aa

Following the general procedure, 4-methoxy-5-nitroisobenzofuran-1,3-dione (223.0 mg, 1.0 mmol, 1.0 equiv) was dissolved in CH_2Cl_2 (0.5 mL, 2 M). Then equimolecular amount of 2-(*tert*-butyl)aniline (149.2 mg, 0.15 mL, 2.0 mmol, 1.0 equiv) was added and reaction mixture was stirred for 12 h at 25 °C. Then the reaction mixture was purified by flash column chromatography (Pet. ether- EtOAc: 50:50 to Pet. ether- EtOAc: 1:100) to afford 6-((2-(*tert*-butyl)phenyl)carbamoyl)-2-methoxy-3-nitrobenzoic acid **1aa** as a white solid (178.7 mg, 48% yield).

R_f (Pet. ether /EtOAc = 30/70): 0.22.

^{1}H NMR (400 MHz, DMSO- *d_6*) δ 13.55 (s, 1H), 10.17 (s, 1H), 8.19 (d, *J* = 8.0 Hz, 1H), 7.65 (d, *J* = 8.1 Hz, 1H), 7.46-7.45 (m, 1H), 7.29-7.28 (m, 2H), 7.13 (s, 1H), 3.91 (s, 3H), 1.37 (s, 9H). **^{13}C NMR (100 MHz, DMSO- *d_6*)** δ 166.1, 165.4, 149.8, 147.0, 144.7, 140.1, 135.6, 131.7, 131.6, 127.6, 126.9, 126.6, 126.0, 123.2, 64.0, 34.9, 30.9. HRMS (ESI) calculated [M+H]+ for $C_{19}H_{21}N_2O_6$: 373.1394, found: 373.1408. **FTIR (cm^{-1})** 3252, 2957, 2874, 1718, 1656, 1587, 1524, 1354, 1270, 1023, 756.

2-((2-(*tert*-Butyl)phenyl)carbamoyl)-6-methoxy-3-nitrobenzoic acid (1ab)

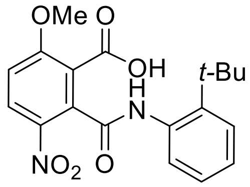

1ab

Following the general procedure, 4-methoxy-7-nitroisobenzofuran-1,3-dione (223.0 mg, 1.0 mmol, 1.0 equiv) was dissolved in CH_2Cl_2 (0.5 mL, 2 M). Then equimolecular amount of 2-(*tert*-butyl)aniline (149.2 mg, 0.15 mL, 2.0 mmol, 1.0 equiv) was added and reaction mixture was stirred for 12 h at 25 °C. Then the reaction mixture was purified by flash column chromatography (Pet. ether- EtOAc: 50:50 to Pet. ether- EtOAc: 1:100) to afford 2-((2-(*tert*-butyl)phenyl)carbamoyl)-6-methoxy-3-nitrobenzoic acid **1ab** as a white solid (249.3 mg, 67% yield).

R_f (Pet. ether /EtOAc = 1/100): 0.12.

^{1}H NMR (400 MHz, DMSO- *d_6*) δ 13.44 (s, 1H), 9.65 (s, 1H), 8.29 (d, *J* = 9.2 Hz, 1H), 7.48-7.46 (m, 1H), 7.43-7.37 (m, 2H), 7.27-7.21 (m, 2H), 3.97 (s, 3H), 1.34 (s, 9H). **^{13}C NMR (100 MHz, DMSO- *d_6*)** δ 167.5, 164.5, 158.6, 145.4, 140.9, 135.7, 130.2, 128.4, 126.7, 126.5, 126.4, 123.8, 123.8, 111.6, 56.3, 34.9, 31.0. **HRMS (ESI)** calculated $[M+H]^+$ for $C_{19}H_{21}N_2O_6$: 373.1394, found: 373.1414. **FTIR (cm^{-1})** 3317, 2957, 2866, 1710, 1655, 1576, 1516, 1336, 1273, 1047 756.

2-((2-(*tert*-Butyl)phenyl)carbamoyl)-4-phenyl-1-naphthoic acid (1ac)

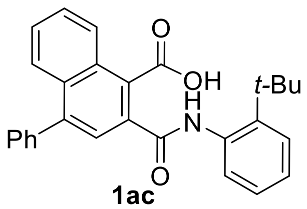

1ac

Following the general procedure, 5-phenylnaphtho[1,2-c]furan-1,3-dione (548.0 mg, 2.0 mmol, 1.0 equiv) was dissolved in CH_2Cl_2 (1.0 mL, 2 M). Then equimolecular amount of 2-(*tert*-butyl)aniline (298.4 mg, 0.3 mL, 2.0 mmol, 1.0 equiv) was added and reaction mixture was stirred for 12 h at 25 °C. Then the reaction mixture was purified by flash column chromatography (Pet. ether- EtOAc: 50:50) to afford 2-((2-(*tert*-butyl)phenyl)carbamoyl)-4-phenyl-1-naphthoic acid **1ac** as a white solid (186.0 mg, 21% yield).

Rf (Pet. ether /EtOAc = 30/70): 0.29.

^{1}H NMR (400 MHz, DMSO- *d*$_6$) δ 13.41 (s, 1H), 9.68 (s, 1H), 8.28 – 8.25 (m, 1H), 7.94 – 7.86 (m, 3H), 7.69 – 7.67 (m, 2H), 7.62 – 7.53 (m, 5H), 7.47 (d, *J* = 6.9 Hz, 1H), 7.32-7.25 (m, 2H), 1.41 (s, 9H). **^{13}C NMR (100 MHz, DMSO- *d*$_6$)** δ 167.7, 167.1, 146.0, 140.2, 139.2, 138.2, 136.2, 132.6, 130.8, 130.1, 129.7, 128.8, 128.6, 128.1, 127.2, 127.0, 126.8, 126.6, 126.4, 126.2, 125.8, 125.5, 35.0, 31.4. **HRMS (ESI)** calculated $[M+H]^+$ for $C_{28}H_{26}NO_3$: 424.1907, found: 424.1923. **FTIR (cm^{-1})** 3446, 2958, 2922, 1705, 1681, 1506, 1437, 1368, 1293, 1251, 1105, 752.

3-((2-(*tert*-Butyl)phenyl)carbamoyl)picolinic acid (1ad)

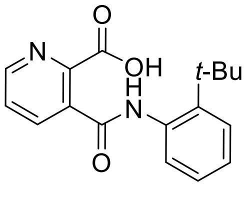

1ad

Following the general procedure, furo[3,4-b]pyridine-5,7-dione (447.3 mg, 3.0 mmol, 1.0 equiv) was dissolved in CH_2Cl_2 (1.0 mL, 0.33M). Then equimolecular amount of 2-*tert* butyl aniline (0.48 mL, 3.0 mmol, 1.0 equiv) was added and reaction mixture was stirred for 6 h at 25 °C. Then the reaction mixture was purified by flash column chromatography (Pet. ether- EtOAc: 50:50) to afford 3-((2-(*tert*-butyl)phenyl)carbamoyl)picolinic acid **1ad** as a white solid (381 mg, 42 % yield).

Rf (Pet. ether /EtOAc = 30/70): 0.40.

^{1}H NMR (400 MHz, DMSO- *d*$_6$) δ 13.40 (bs, 1H), 10.01 (s, 1H), 8.80 (dd, J_1 = 4.5 Hz, J_2 = 1.1 Hz, 1H), 8.17 (dd, J_1 = 7.8 Hz, J_2 = 1.2 Hz, 1H), 7.70-7.66 (m, 1H), 7.55 (d, *J* = 7.6 Hz, 1H), 7.44 (d, *J* = 7.8 Hz, 1H), 7.29-7.22 (m, 2H), 1.42 (s, 9H). **^{13}C NMR (100 MHz, DMSO-*d*$_6$)** δ 167.8, 163.8, 150.7, 150.1, 144.7, 137.2, 135.5, 128.9, 128.7, 126.5, 126.4, 125.4, 34.6, 30.6. **HRMS (ESI)** calculated $[M+Na]^+$ for $C_{17}H_{18}N_2O_3Na$: 321.1210, found: 321.1212. **FTIR (cm^{-1})** 3250, 2960, 2862, 1710, 1660, 1585, 1512, 1450, 1270, 754.

(*Z*)-4-((2-(*tert*-Butyl)phenyl)amino)-2-methyl-4-oxobut-2-enoic acid (8a)

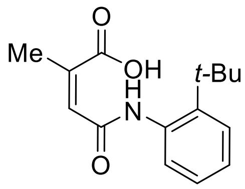

8a

Following the general procedure, 3-methylfuran-2,5-dione (93 μL, 1.0 mmol, 1.0 equiv) was dissolved in CH_2Cl_2 (0.5 mL, 2M). Then equimolecular amount of 2-*tert* butyl aniline (0.16 mL, 1.0 mmol, 1.0 equiv) was added and reaction mixture was stirred for 6 h at 25 °C. Then the reaction mixture was purified by flash column chromatography (Pet. ether- EtOAc: 50:50) to afford (*Z*)-4-((2-(*tert*-butyl)phenyl)amino)-2-methyl-4-oxobut-2-enoic acid **8a** as a white solid (183.8 mg, 71% yield).

R_f (Pet. ether /EtOAc = 30/70): 0.22.

^{1}H NMR (400 MHz, DMSO- *d_6*) δ 13.42 (bs, 1H), 9.62 (s, 1H), 7.42-7.41 (m, 1H), 7.23-7.21 (m, 2H), 7.10-7.09 (m, 1H), 6.34 (s, 1H), 2.50 (s, 3H), 1.32 (s, 9H). **^{13}C NMR (100 MHz, DMSO- *d_6*)** δ 169.3, 164.4, 146.4, 142.3, 135.2, 131.2, 127.2, 126.7, 126.4, 124.3, 34.8, 30.8, 21.0. **HRMS (ESI)** calculated $[M+H]^+$ for $C_{15}H_{20}NO_3$: 262.1438, found: 262.1446. **FTIR (cm^{-1})** 3250, 2968, 2869, 1706, 1630, 1526, 1485, 1368, 1303, 1055, 1025.

(*Z*)-4-((4-Bromo-2-(*tert*-butyl)phenyl)amino)-2-methyl-4-oxobut-2-enoicacid (8b)

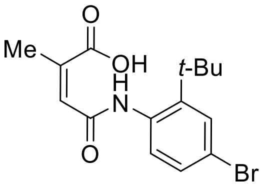

8b

Following the general procedure, 3-methylfuran-2,5-dione (93 μL, 1.0 mmol, 1.0 equiv) was dissolved in CH_2Cl_2 (0.5 mL, 2M). Then equimolecular amount of 4-bromo-2-(*tert*-butyl)aniline (227 mg, 1.0 mmol, 1.0 equiv) was added and reaction mixture was stirred for 6 h at 25 °C. Then the reaction mixture was purified by flash column chromatography (Pet. ether- EtOAc: 50:50) to afford (*Z*)-4-((4-bromo-2-(*tert*-butyl)phenyl) amino)-2-methyl-4-oxobut-2-enoic acid **8b** as a white solid (304 mg, 89% yield).

R_f (Pet. ether /EtOAc = 30/70): 0.35.

^{1}H NMR (400 MHz, DMSO- *d_6*) δ 13.11 (bs, 1H), 9.53 (bs, 1H), 7.49 (d, J = 1.9 Hz, 1H), 7.42 (dd, J_1 = 8.3 Hz, J_2 = 1.9 Hz, 1H), 7.04 (d, J = 8.3 Hz, 1H), 6.26 (s, 1H), 1.98 (s, 3H), 1.29 (s, 9H). **^{13}C NMR (100 MHz, DMSO- *d_6*)** δ 169.9, 164.5, 149.3, 142.4, 134.9, 133.6, 129.7, 129.5, 123.9, 120.2, 35.1, 30.5, 20.8. **HRMS (ESI)** calculated $[M+H]^+$ for $C_{15}H_{18}BrNO_3$: 340.0543, found: 340.0550. **FTIR (cm^{-1})** 3283, 3239,2965, 2874, 1706, 1699, 1629, 1520,1486, 1301, 792.

(*Z*)-4-((2-(*tert*-Butyl)-4-iodophenyl)amino)-2-methyl-4-oxobut-2-enoic acid (8c)

Following the general procedure, 3-methylfuran-2,5-dione (93 μL, 1.0 mmol, 1.0 equiv) was dissolved in CH_2Cl_2 (0.5 mL, 2M). Then equimolecular amount of 2-(*tert*-butyl)-4-iodoaniline (275.1 mg, 1.0 mmol, 1.0 equiv) was added and reaction mixture was stirred for 6 h at 25 °C. Then the reaction mixture was purified by flash column chromatography (Pet. ether-EtOAc: 50:50) to afford (*Z*)-4-((2-(*tert*-butyl)-4-iodophenyl)amino)-2-methyl-4-oxobut-2-enoic acid **8c** as a white solid (259.5 mg, 67% yield).

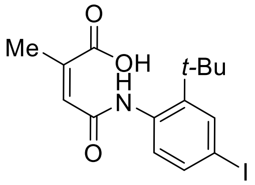

8c

***R**f* (Pet. ether /EtOAc = 30/70): 0.25.

^{1}H NMR (400 MHz, DMSO- *d*6) δ 13.13 (bs, 1H), 9.52 (s, 1H), 7.65 (s, 1H), 7.58 (d, *J* = 8.2 Hz, 1H), 6.88 (d, *J* = 8.2 Hz, 1H), 6.25 (s, 1H), 1.98 (s, 3H), 1.28 (s, 9H). **^{13}C NMR (100 MHz, DMSO- *d*6)** δ 169.5, 164.3, 149.1, 142.2, 135.4, 135.4, 133.5, 124.0, 93.2, 34.8, 30.5, 20.7. **HRMS (ESI)** calculated $[M+Na]^+$ for $C_{15}H_{18}INO_3Na$: 410.0224, found: 410.0230. **FTIR (cm^{-1})** 3243, 2968, 2869, 1695, 1628, 1510, 1478, 1369, 1303, 1054, 1013.

(*Z*)-4-((2-(*tert*-Butyl)-4-(ethoxycarbonyl)phenyl)amino)-2-methyl-4-oxobut-2-enoic acid (8d)

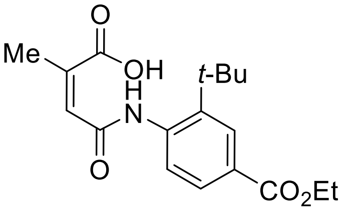

8d

Following the general procedure, 3-methylfuran-2,5-dione (93 μL mg, 1.0 mmol, 1.0 equiv) was dissolved in CH_2Cl_2 (0.5 mL, 2M). Then equimolecular amount of ethyl 4-amino-3-(*tert*-butyl)benzoate (221.1 mg, 1.0 mmol, 1.0 equiv) was added and reaction mixture was stirred for 6 h at 25 °C. Then the reaction mixture was purified by flash column chromatography (Pet. ether- EtOAc: 50:50) to afford (Z)-4-((2-(*tert*-butyl)-4-(ethoxycarbonyl)phenyl)amino)-2-methyl-4-oxobut-2-enoic acid **8d** as a white solid (281.7 mg, 84% yield).

***R**f* (Pet. ether /EtOAc = 30/70): 0.20.

^{1}H NMR (400 MHz, DMSO- *d*6) δ 13.04 (bs, 1H), 9.56 (bs, 1H), 8.01 (s, 1H), 7.80 (d, *J* = 8.1 Hz, 1H), 7.28 (d, *J* = 8.1 Hz, 1H), 6.28 (s, 1H), 4.30 (q, *J* = 7.1 Hz, 2H), 1.99 (s, 3H), 1.34-1.30 (m, 12H). **^{13}C NMR (100 MHz, DMSO- *d*6)** δ 171.0, 166.6, 165.0, 147.3, 143.7, 140.6, 132.1, 128.9, 128.3, 127.9, 123.8, 61.8, 35.5, 31.0, 21.3, 14.8. **HRMS (ESI)** calculated $[M+H]^+$ for $C_{15}H_{18}BrNO_3$: 334.1649, found: 334.1654. **FTIR (cm^{-1})** 3238, 2971, 2871, 1715, 1632, 1522, 1400, 1369, 1242, 762.

(*Z*)-2-Methyl-4-((4-methyl-2-(2-phenylpropan-2-yl)phenyl)amino)-4-oxobut-2-enoic acid (8e)

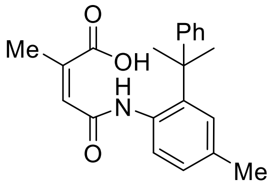

8e

Following the general procedure, 3-methylfuran-2,5-dione (93 μL, 1.0 mmol, 1.0 equiv) was dissolved in CH_2Cl_2 (0.5 mL, 2M). Then equimolecular amount of 4-methyl-2-(2-phenylpropan-2-yl)aniline (225.3 mg, 1.0 mmol, 1.0 equiv) was added and reaction mixture was stirred for 6 h at 25 °C. Then the reaction mixture was purified by flash column chromatography (Pet. ether- EtOAc: 50:50) to afford (*Z*)-2-methyl-4-((4-methyl-2-(2-phenylpropan-2-yl)phenyl)amino)-4-oxobut-2-enoic acid **8e** as a white solid (182.2 mg, 54% yield).

Rf (Pet. ether /EtOAc = 30/70): 0.22.

^{1}H NMR (400 MHz, DMSO- *d6*) δ 13.71 (bs, 1H), 7.97 (s, 1H), 7.40 (s, 1H), 7.27-7.20 (m, 3H), 7.15-7.11 (m, 3H), 7.07 (d, *J* = 7.9 Hz, 1H), 5.70 (s, 1H), 2.35 (s, 3H), 1.86 (s, 3H), 1.60 (s, 6H). **^{13}C NMR (100 MHz, DMSO- *d6*)** δ 168.2, 163.0, 149.0, 142.8, 141.7, 135.3, 131.7, 128.4, 127.8, 127.3, 127.3, 125.8, 125.5, 124.8, 42.1, 30.2, 21.0, 21.0. **HRMS (ESI)** calculated $[M+Na]^+$ for $C_{21}H_{23}NO_3Na$: 360.1570, found: 360.1577. **FTIR (cm^{-1})** 3240, 2986, 2860, 1692, 1630, 1515, 1470, 1371, 1300, 1050, 1012.

(*E*)-3-Bromo-4-((2-(*tert*-butyl)phenyl)amino)-2-methyl-4-oxobut-2-enoic acid (8f)

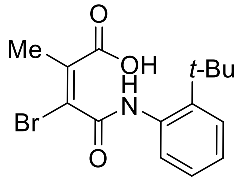

8f

Following the general procedure, 3-bromo-4-methylfuran-2,5-dione (382 mg, 2.0 mmol, 1.0 equiv) was dissolved in CH_2Cl_2 (1.0 mL, 2M). Then equimolecular amount of 2-*tert* butyl aniline (0.32 mL, 1.0 mmol, 1.0 equiv) was added and reaction mixture was stirred for 2 h at 25 °C. Then the reaction mixture was purified by flash column chromatography (neutralize with Et_3N then DCM-MeOH: 95:5) to afford (*E*)-3-bromo-4-((2-(*tert*-butyl)phenyl)amino)-2-methyl-4-oxobut-2-enoic acid **8f** as a white solid (190.2 mg, 28% yield).

Rf (DCM /MeOH = 10/90): 0.20.

^{1}H NMR (400 MHz, DMSO-*d6*) δ 13.12 (s, 1H), 9.68 (s, 1H), 7.40-7.39 (m, 1H), 7.25–7.23 (m, 3H), 2.07 (s, 3H), 1.33 (s, 9H). **^{13}C NMR (100 MHz, DMSO-*d6*)** δ 165.7, 164.2, 147.0, 135.3, 131.1, 130.7, 129.0, 127.3, 126.5, 126.3, 34.8, 30.7, 18.9. **HRMS (ESI)** calculated $[M+H]^+$ for $C_{15}H_{19}BrNO_3$: 340.0543, found: 340.0556. **FTIR (cm^{-1})** 3250, 2968, 2869, 1706, 1630, 1526, 1485, 1368, 1303, 1055, 1025.

2.5 Synthesis and Characterization of *N*-Aryl Phthalimides/Maleimides

(*P*)-2-(2-(*tert*-Butyl)phenyl)-4-methylisoindoline-1,3-dione (2a)

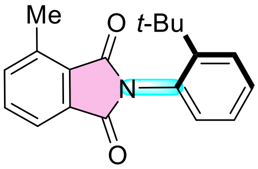

2a

Following the general procedure, K_2CO_3 (52 mg, 0.375 mmol) and 2-((2-(*tert*-butyl)phenyl)carbamoyl)-6-methylbenzoic acid **1a** (77.8 mg, 0.25 mmol) were taken in THF (2.0 mL), and then PivCl (46 μL, 0.375 mmol) was added to the reaction mixture, and it was stirred at 25 °C for 30 min (until the full conversion of **1a**, monitored by TLC). Then, the triazolium salt **3** (9.2 mg, 0.025 mmol, 10 mol %) and THF (1.0 mL) were successively added and stirred for 24 h. Then the reaction mixture was purified by flash column chromatography (Pet. ether- EtOAc: 95:5) to afford (*P)*-2-(2-(*tert*-butyl)phenyl)-4-methylisoindoline-1,3-dione **2a** as a light-yellow solid (73.0 mg, 99% yield).

R R_f (Pet. ether /EtOAc = 90/10): 0.52; er = 98:2, $[\alpha]_D^{25}$ = -4.2 (c 1.0, $CHCl_3$). **HPLC** (CHIRALPAK IA, *n*-hexane/IPA = 98:2, flow rate = 1.0 mL/min, λ = 254 nm) t_R = 6.0 min (minor), 6.8 min (major).

^{1}H NMR (400 MHz, $CDCl_3$) δ 7.79 (d, *J* = 7.3 Hz, 1H), 7.66-7.62 (m, 2H), 7.54 (d, *J* = 7.8 Hz, 1H), 7.45-7.41 (m, 1H), 7.33-7.29 (m, 1H), 7.00 (dd, J_1 = 7.7 Hz, J_2 = 1.4 Hz, 1H), 2.75 (s, 3H), 1.33 (s, 9H). **^{13}C NMR (100 MHz, $CDCl_3$)** δ 169.5, 168.8, 149.5, 138.6, 136.8, 133.9, 133.0, 131.6, 130.1, 129.8, 129.2, 128.8, 127.4, 121.5, 35.7, 31.8, 17.8. **HRMS (ESI)** calculated $[M+Na]^+$ for $C_{19}H_{19}NO_2Na$: 316.1308, found: 316.1312. **FTIR (cm^{-1})** 2963, 2924, 1713, 1615, 1486, 1442, 1375, 1196, 1103, 806.

(*M*)-2-(2-(*tert*-butyl)phenyl)-4-methylisoindoline-1,3-dione (*ent*-2a)

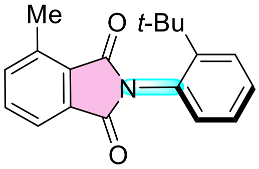

***ent*-2a**

Following the general procedure, K_2CO_3 (52 mg, 0.375 mmol) and 2-((2-(*tert*-butyl)phenyl)carbamoyl)-6-methylbenzoic acid **1a′** (77.8 mg, 0.25 mmol) were taken in THF (2.0 mL), and then PivCl (46 μL, 0.375 mmol) was added to the reaction mixture, and it was stirred at 25 °C for 30 min (until the full conversion of **1a′**, monitored by TLC). Then, the triazolium salt **3** (9.2 mg, 0.025 mmol, 10 mol %) and THF (1.0 mL) were successively added and stirred for 24 h. Then the reaction mixture was purified by flash column chromatography (Pet. ether- EtOAc: 95:5) to afford (*M*)-2-(2-(*tert*-butyl)phenyl)-4-methylisoindoline-1,3-dione ***ent*-2a** as a light-yellow solid (73.0 mg, 99% yield).

R_f (Pet. ether /EtOAc = 90/10): 0.52; er = 97:3, $[\alpha]_D^{25}$ = 2.1 (c 1.0, $CHCl_3$). **HPLC** (CHIRALPAK IA, *n*-hexane/IPA = 98:2, flow rate = 1.0 mL/min, λ = 254 nm) t_R = 6.3 min (major), 7.0 min (minor).

(*P*)-2-(2-(*tert*-Butyl)-4-methoxyphenyl)-4-methylisoindoline-1,3-dione (2b)

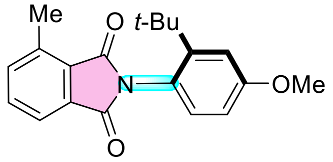

2b

Following the general procedure, K_2CO_3 (52 mg, 0.375 mmol) and 2-((2-(*tert*-butyl)-4-methoxyphenyl)carbamoyl)-6-methylbenzoic acid **1b** (85.4 mg, 0.25 mmol) were taken in THF (2.0 mL), and then PivCl (46 µL, 0.375 mmol) was added to the reaction mixture, and it was stirred at 25 °C for 30 min (until the full conversion of **1b**, monitored by TLC). Then, the triazolium salt **3** (9.2 mg, 0.025 mmol, 10 mol %) and THF (1.0 mL) were successively added and stirred for 24 h. Then the reaction mixture was purified by flash column chromatography (Pet. ether- EtOAc: 95:5) to afford (*P*)- 2-(2-(*tert*-butyl)-4-methoxyphenyl)-4-methylisoindoline-1,3-dione **2b** as a light-yellow solid (80.0 mg, 99% yield).

R_f (Pet. ether /EtOAc = 90/10): 0.34; er = 98:2, $[\alpha]_D^{25}$ = -14.3 (c 1.0, $CHCl_3$). **HPLC** (CHIRALCELL OD-H, *n*-hexane/IPA = 99:1, flow rate = 1.0 mL/min, λ = 254 nm) t_R = 13.5 min (minor), 15.2 min (major).

^{1}H NMR (400 MHz, $CDCl_3$) δ 7.80 (d, *J* = 7.3 Hz, 1H), 7.65 (t, *J* = 7.6 Hz, 1H), 7.55 (d, *J* = 7.8 Hz, 1H), 7.16 (d, *J* = 2.8 Hz, 1H), 6.96 (d, *J* = 8.6 Hz, 1H), 6.85 (dd, J_1 = 8.6 Hz, J_2 = 2.8 Hz, 1H), 3.86 (s, 3H), 2.77 (s, 3H), 1.33 (s, 9H). **^{13}C NMR (100 MHz, $CDCl_3$)** δ 169.7, 169.1, 160.2, 150.9, 138.5, 136.7, 133.8, 132.9, 132.5, 129.2, 122.7, 121.5, 115.4, 111.5, 55.4, 35.7, 31.6, 17.8. **HRMS (ESI)** calculated $[M+H]^+$ for $C_{20}H_{22}NO_3$: 324.1594, found: 324.1597. **FTIR (cm^{-1})** 2990, 2965, 1712, 1495, 1470, 1387, 1218, 1057, 748.

(*P*)-2-(4-(Benzyloxy)-2-(*tert*-butyl)phenyl)-4-methylisoindoline-1,3-dione (2c)

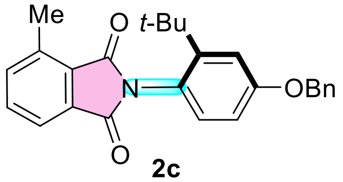

2c

Following the general procedure, K_2CO_3 (52 mg, 0.375 mmol) and 2-((4-(benzyloxy)-2-(*tert*-butyl)phenyl)carbamoyl)-6-methylbenzoic acid **1c** (104.4 mg, 0.25 mmol) were taken in THF (2.0 mL), and then PivCl (46 µL, 0.375 mmol) was added to the reaction mixture, and it was stirred at 25 °C for 30 min (until the full conversion of **1c**, monitored by TLC). Then, the triazolium salt **3** (9.2 mg, 0.025 mmol, 10 mol %) and THF (1.0 mL) were successively added and stirred for 24 h. Then the reaction mixture was purified by

flash column chromatography (Pet. ether- EtOAc: 95:5) to afford (*P)*-2-(4-(benzyloxy)-2-(*tert*-butyl)phenyl)-4-methylisoindoline-1,3-dione **2c** as a light-yellow solid (99.6 mg, 99% yield).

Rf (Pet. ether /EtOAc = 90/10): 0.45; er = 97:3, $[\alpha]_D^{25}$ = -7.9 (c 1.0, $CHCl_3$). **HPLC** (CHIRALPAK IA, *n*-hexane/IPA = 98:2, flow rate = 1.0 mL/min, λ = 254 nm) t_R = 13.9 min (minor), 14.0 min (major).

^{1}H NMR (400 MHz, $CDCl_3$) δ 7.78 (d, *J* = 7.3 Hz, 1H), 7.64 (t, *J* = 7.6 Hz, 1H), 7.54 (d, *J* = 7.5 Hz, 1H), 7.48-7.46 (m, 2H), 7.44-7.40 (m, 2H), 7.37-7.34 (m, 1H), 7.24 (d, *J* = 2.4 Hz, 1H), 6.95-6.89 (m, 2H) 5.09 (s, 2H), 2.75 (s, 3H), 1.31 (s, 9H). **^{13}C NMR (100 MHz, $CDCl_3$)** δ 169.8, 169.1, 159.5, 151.0, 138.6, 136.9, 136.8, 133.9, 132.9, 132.6, 129.2, 128.8, 128.2, 127.7, 122.9, 121.5, 116.2, 112.4, 70.4, 35.7, 31.6, 17.8. **HRMS (ESI)** calculated $[M+Na]^+$ for $C_{26}H_{25}NO_3Na$: 422.1727, found: 422.1731 . **FTIR (cm^{-1})** 2962, 2925, 1714, 1607, 1571, 1413, 1222, 1103, 771, 743.

(*P*)-2-(4-Bromo-2-(*tert*-butyl)phenyl)-4-methylisoindoline-1,3-dione (2d)

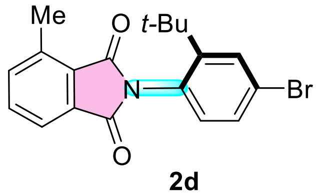

2d

Following the general procedure, K_2CO_3 (52 mg, 0.375 mmol) and 2-((4-bromo-2-(*tert*-butyl)phenyl)carbamoyl)-6-methylbenzoic acid **1d** (97.6 mg, 0.25 mmol) were taken in THF (2.0 mL), and then PivCl (46 μL, 0.375 mmol) was added to the reaction mixture, and it was stirred at 25 °C for 30 min (until the full conversion of **1d**, monitored by TLC). Then, the triazolium salt **3** (9.2 mg, 0.025 mmol, 10 mol %) and THF (1.0 mL) were successively added and stirred for 24 h. Then the reaction mixture was purified by flash column chromatography (Pet. ether- EtOAc: 95:5) to (*P*)-2-(4-bromo-2-(*tert*-butyl)phenyl)-4-methylisoindoline-1,3-dione (**2d**) as a light-yellow solid (93.0 mg, 99% yield).

Rf (Pet. ether /EtOAc = 90/10): 0.52; er = 97:3, $[\alpha]_D^{25}$ = -7.1 (c 1.0, $CHCl_3$). **HPLC** (CHIRALPAK AD, *n*-hexane/IPA = 95:5, flow rate = 1.0 mL/min, λ = 254 nm) t_R = 6.2 min (minor), 7.2 min (major).

^{1}H NMR (400 MHz, $CDCl_3$) δ 7.78 (d, *J* = 7.4 Hz, 1H), 7.74 (d, *J* = 2.1 Hz, 1H), 7.65 (t, *J* = 7.7 Hz, 1H), 7.55 (d, *J* = 7.9 Hz, 1H), 7.43 (dd, J_1 = 8.3 Hz, J_2 = 2.2 Hz, 1H), 6.87 (d, *J* = 8.3 Hz, 1H), 2.74 (s, 3H), 1.31 (s, 9H). **^{13}C NMR (100 MHz, $CDCl_3$)** δ 169.2, 168.5, 151.9, 138.8, 137.0, 134.1, 133.2, 132.8, 132.2, 130.6, 129.4, 129.1, 124.2, 121.7, 35.9, 31.6, 17.9. **HRMS (ESI)** calculated $[M+Na]^+$ for $C_{19}H_{18}BrNO_2Na$: 394.0413, found: 394.0417. **FTIR (cm^{-1})** 3019, 2963, 1713, 1497, 1479, 1383, 1212, 1097, 747.

(*P*)- 2-(2-(*tert*-Butyl)-4-chlorophenyl)-4-methylisoindoline-1,3-dione (2e)

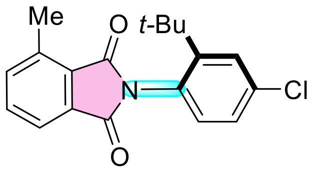

2e

Following the general procedure, K_2CO_3 (26 mg, 0.188 mmol) and 2-((2-(*tert*-butyl)-4-chlorophenyl)carbamoyl)-6-methylbenzoic acid **1e** (43.2 mg, 0.125 mmol) were taken in THF (1.0 mL), and then PivCl (23 μL, 0.188 mmol) was added to the reaction mixture, and it was stirred at 25 °C for 30 min (until the full conversion of **1e**, monitored by TLC). Then, the triazolium salt **3** (0.0046 g, 0.0125 mmol, 10 mol %) and THF (0.5 mL) were successively added and stirred for 24 h. Then the reaction mixture was purified by flash column chromatography (Pet. ether- EtOAc: 95:5) to (*P*)-2-(2-(*tert*-butyl)-4-chlorophenyl)-4-methylisoindoline-1,3-dione (**2e**) as a light-yellow solid (41.8 mg, 99% yield).

R_f (Pet. ether /EtOAc = 90/10): 0.55; er = 97:3, $[\alpha]_D^{25}$ = -4.7 (c 1.0, $CHCl_3$). **HPLC** (CHIRALPAK IA, *n*-hexane/IPA = 98:2, flow rate = 1.0 mL/min, λ = 254 nm) t_R = 6.6 min (minor), 8.0 min (major).

^{1}H NMR (400 MHz, $CDCl_3$) δ 7.77 (d, *J* = 7.3 Hz, 1H), 7.64 (t, *J* = 7.5 Hz, 1H), 7.58-7.58 (m, 1H), 7.54 (d, *J* = 7.7 Hz, 1H), 7.27 (dd, J_1 = 8.4 Hz, J_2 = 1.9 Hz, 1H), 6.94 (d, *J* = 8.3 Hz, 1H), 2.73 (s, 3H), 1.31 (s, 9H). **^{13}C NMR (100 MHz, $CDCl_3$)** δ 169.2, 168.6, 151.5, 138.8, 137.0, 135.7, 134.1, 132.9, 132.7, 129.1, 129.0, 128.8, 127.5, 121.6, 35.9, 31.5, 17.9. **HRMS (ESI)** calculated $[M+H]^+$ for $C_{19}H_{19}ClNO_2$: 328.1099, found: 328.1098. **FTIR (cm^{-1})** 2961, 2923, 1714, 1482, 1369, 1262, 1102, 930, 743.

(*P*)-2-(2-(*tert*-Butyl)-4-iodophenyl)-4-methylisoindoline-1,3-dione (2f)

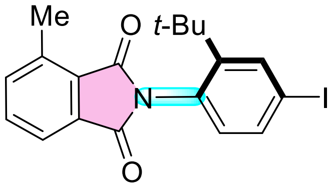

2f

Following the general procedure, K_2CO_3 (52 mg, 0.375 mmol) and 2-((2-(*tert*-butyl)-4-iodophenyl)carbamoyl)-6-methyl benzoic acid **1f** (109.2 mg, 0.25 mmol) were taken in THF (2.0 mL), and then PivCl (46 μL, 0.375 mmol) was added to the reaction mixture, and it was stirred at 25 °C for 30 min (until the full conversion of **1f**, monitored by TLC). Then, the triazolium salt **3** (9.2 mg, 0.025 mmol, 10 mol %) and THF (1.0 mL) were successively added and stirred for 24 h. Then the reaction mixture was purified by flash column chromatography (Pet. ether- EtOAc: 95:5) to afford (*P)*-2-(2-(*tert*-butyl)-4-iodophenyl)-4-methylisoindoline-1,3-dione **2f** as a light-yellow solid (104.0 mg, 99% yield).

Rf (Pet. ether /EtOAc = 90/10): 0.38; er = 95:5, $[\alpha]_D^{25}$ = +4.5 (c 1.0, $CHCl_3$). **HPLC** (CHIRALPAK IA, *n*-hexane/IPA = 99:1, flow rate = 1.0 mL/min, λ = 254 nm) t_R = 9.9 min (minor), 13.2 min (major).

^{1}H NMR (400 MHz, $CDCl_3$) δ 7.92 (d, *J* = 1.8 Hz, 1H), 7.77 (d, *J* = 7.6 Hz, 1H), 7.66-7.62 (m, 2H), 7.54 (d, *J* = 7.8 Hz, 1H), 6.71 (d, *J* = 8.0 Hz, 1H), 2.74 (s, 3H), 1.30 (s, 9H). **^{13}C NMR (100 MHz, $CDCl_3$)** δ 169.2, 168.5, 152.0, 138.8, 138.2, 137.0, 136.6, 134.1, 133.3, 132.8, 130.2, 129.1, 121.7, 96.4, 35.8, 31.6, 17.9. **HRMS (ESI)** calculated $[M+Na]^+$ for $C_{19}H_{18}INO_2Na$: 442.0274, found: 442.0282. **FTIR (cm^{-1})** 2962, 2922, 2857, 1774, 1713, 1614, 1582, 1481, 1387, 1197, 746.

(*P*)-2-(3-(*tert*-Butyl)-[1,1'-biphenyl]-4-yl)-4-methylisoindoline-1,3-dione (2g)

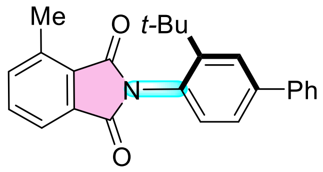

2g

Following the general procedure, K_2CO_3 (52 mg, 0.375 mmol) and 2-((3-(*tert*-butyl)-[1,1'-biphenyl]-4-yl)carbamoyl)-6-methylbenzoic acid **1g** (96.9 mg, 0.25 mmol) were taken in THF (2.0 mL), and then PivCl (46 µL, 0.375 mmol) was added to the reaction mixture, and it was stirred at 25 °C for 30 min (until the full conversion of **1g**, monitored by TLC). Then, the triazolium salt **3** (9.2 mg, 0.025 mmol, 10 mol %) and THF (1.0 mL) were successively added and stirred for 24 h. Then the reaction mixture was purified by flash column chromatography (Pet. ether- EtOAc: 95:5) to (*P*)-2-(3-(*tert*-butyl)-[1,1'-biphenyl]-4-yl)-4-methylisoindoline-1,3-dione **2g** as a light-yellow solid (89.4 mg, 97% yield).

Rf (Pet. ether /EtOAc = 90/10): 0.50; er = 96:4, $[\alpha]_D^{25}$ = -10.6 (c 1.0, $CHCl_3$). **HPLC** (CHIRALPAK IA, *n*-hexane/IPA = 99:1, flow rate = 1.0 mL/min, λ = 254 nm) t_R = 16.0 min (minor), 17.6 min (major).

^{1}H NMR (400 MHz, $CDCl_3$) δ 7.83-7.81 (m, 2 H), 7.68-7.61 (m, 3H), 7.57-7.46 (m, 4H), 7.41-7.37 (m, 1H), 7.09 (d, *J* = 8.0 Hz, 1H), 2.78 (s, 3H), 1.40 (s, 9H). **^{13}C NMR (100 MHz, $CDCl_3$)** δ 169.6, 168.9, 149.7, 142.7, 141.0, 138.7, 136.9, 134.0, 132.9, 131.9, 129.2, 129.2, 128.9, 128.0, 127.7, 127.5, 126.2, 121.6, 35.9, 31.8, 17.9. **HRMS (ESI)** calculated $[M+Na]^+$ for $C_{25}H_{23}NO_2Na$: 392.1621, found: 392.1325. **FTIR (cm^{-1})** 2960, 2923, 1714, 1482, 1375, 1238, 1197, 1102, 750.

(*P*)-Ethyl 3-(*tert*-butyl)-4-(4-methyl-1,3-dioxoisoindolin-2-yl)benzoate (2h)

Following the general procedure, K_2CO_3 (52 mg, 0.375 mmol) and 2-((2-(*tert*-butyl)-4-(ethoxycarbonyl)phenyl)carbamoyl)-6-methylbenzoic acid **1h** (95.8 mg, 0.25 mmol) were

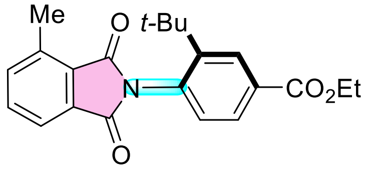

2h

taken in THF (2.0 mL), and then PivCl (46 µL, 0.375 mmol) was added to the reaction mixture, and it was stirred at 25 °C for 30 min (until the full conversion of **1h**, monitored by TLC). Then, the triazolium salt **3** (9.2 mg, 0.025 mmol, 10 mol %) and THF (1.0 mL) were successively added and stirred for 24 h. Then the reaction mixture was purified by flash column chromatography (Pet. ether-EtOAc: 95:5) to (*P*)-ethyl 3-(*tert*-butyl)-4-(4-methyl-1,3-dioxoisoindolin-2-yl)benzoate **2h** as a light-yellow solid (91.0 mg, 99% yield).

R_f (Pet. ether /EtOAc = 90/10): 0.49; er = 92:8, $[\alpha]_D^{25}$ = -5.5 (c 1.0, $CHCl_3$). **HPLC** (CHIRALCELL OD-H, *n*-hexane/IPA = 95:5, flow rate = 1.0 mL/min, λ = 254 nm) t_R = 6.9 min (minor), 8.1 min (major).

^{1}H NMR (400 MHz, $CDCl_3$) δ 8.33 (s, 1H), 7.96 (d, *J* = 8.1 Hz, 1H), 7.78 (d, *J* = 7.7 Hz, 1H), 7.65 (t, *J* = 6.8 Hz, 1H), 7.55 (d, *J* = 7.4 Hz, 1H), 7.08 (d, *J* = 6.8 Hz, 1H), 4.41-4.40 (m, 2 H), 2.74 (s, 3H), 1.42-1.25 (m, 12 H). **^{13}C NMR (100 MHz, $CDCl_3$)** δ 169.1, 168.4, 166.2, 150.0, 138.8, 137.0, 134.5, 134.1, 132.8, 131.9, 131.7, 130.3, 129.1, 128.3, 121.7, 61.4, 35.9, 31.7, 17.9, 14.5. **HRMS (ESI)** calculated $[M+Na]^+$ for $C_{22}H_{23}NO_4Na$: 388.1519, found: 388.1525. **FTIR (cm^{-1})** 2965, 2924, 1715, 1480, 1372, 1242, 1109, 1020, 805.

(*P*)-2-(2-(*tert*-Butyl)-4-(thiophen-3-yl)phenyl)-4-methylisoindoline-1,3-dione (2i)

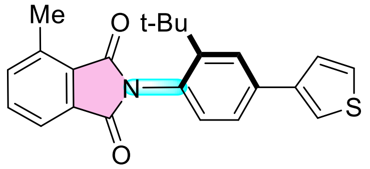

2i

Following the general procedure, K_2CO_3 (52 mg, 0.375 mmol) and 2-((2-(*tert*-butyl)-4-(thiophen-3-yl)phenyl)carbamoyl)-6-methylbenzoic acid **1i** (98.4 mg, 0.25 mmol) were taken in THF (2.0 mL), and then PivCl (46 µL, 0.375 mmol) was added to the reaction mixture, and it was stirred at 25 °C for 30 min (until the full conversion of **1i**, monitored by TLC). Then, the triazolium salt **3** (9.2 mg, 0.025 mmol, 10 mol %) and THF (1.0 mL) were successively added and stirred for 24 h. Then the reaction mixture was purified by flash column chromatography (Pet. ether- EtOAc: 95:5) to afford (*P*)-2-(2-(*tert*-butyl)-4-(thiophen-3-yl)phenyl)-4-methylisoindoline-1,3-dione **2i** as a light-yellow solid (92.1 mg, 98% yield).

R_f (Pet. ether /EtOAc = 90/10): 0.34; er = 92:8, $[\alpha]_D^{25}$ = -8.7 (c 1.0, $CHCl_3$). **HPLC** (CHIRALPAK IF, *n*-hexane/IPA = 95:5, flow rate = 1.0 mL/min, λ = 254 nm) t_R = 14.9 min (minor), 24.0 min (major).

^{1}H NMR (400 MHz, CDCl$_3$) δ 7.83 (d, *J* = 1.7 Hz, 1H), 7.80 (d, *J* = 7.3 Hz, 1H), 7.65 (t, *J* = 7.7 Hz, 1H), 7.56-7.48 (m, 3H), 7.42-7.39 (m, 2H), 7.05 (d, *J* = 8.0 Hz, 1H), 2.76 (s, 3H), 1.37 (s, 9H). **^{13}C NMR (100 MHz, CDCl$_3$)** δ 169.5, 168.9, 149.8, 142.1, 138.7, 137.4, 136.9, 133.9, 132.9, 132.0, 129.2, 129.0, 127.1, 126.7, 126.5, 125.6, 121.6, 121.2, 35.8, 31.8, 17.9. **HRMS (ESI)** calculated $[M+Na]^+$ for $C_{23}H_{21}NO_2SNa$: 398.1185, found: 398.1190. **FTIR (cm^{-1})** 2960, 2923, 2856, 1771, 1711, 1608, 1485, 1375, 1263, 774.

(*P*, *E*)-2-(2-(*tert*-Butyl)-4-styrylphenyl)-4-methylisoindoline-1,3-dione (2j)

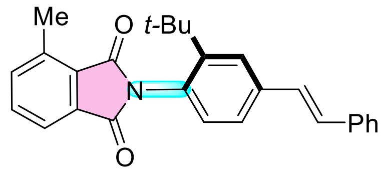

2j

Following the general procedure, K_2CO_3 (52 mg, 0.375 mmol) and (*E*)-2-((2-(*tert*-butyl)-4-styrylphenyl)carbamoyl)-6-methylbenzoic acid **1j** (103.4 mg, 0.25 mmol) were taken in THF (2.0 mL), and then PivCl (46 μL, 0.375 mmol) was added to the reaction mixture, and it was stirred at 25 °C for 30 min (until the full conversion of **1j**, monitored by TLC). Then, the triazolium salt **3** (9.2 mg, 0.025 mmol, 10 mol %) and THF (1.0 mL) were successively added and stirred for 24 h. Then the reaction mixture was purified by flash column chromatography (Pet. ether- EtOAc: 95:5) to (*P*, *E*)-2-(2-(*tert*-butyl)-4-styrylphenyl)-4-methylisoindoline-1,3-dione **2j** as a light-yellow solid (96.7 mg, 98% yield).

R_f (Pet. ether /EtOAc = 90/10): 0.50; er = 93:7, $[\alpha]_D^{25}$ = -13.1 (c 1.0, CHCl$_3$). **HPLC** (CHIRALPAK IA, *n*-hexane/IPA = 98:2, flow rate = 1.0 mL/min, λ = 254 nm) t_R = 15.3 min (minor), 16.9 min (major).

^{1}H NMR (400 MHz, CDCl$_3$) δ 7.80 (d, *J* = 7.2 Hz, 1H), 7.71 (d, *J* = 1.8 Hz, 1H), 7.65 (d, *J* = 7.3 Hz, 1H), 7.56-7.54 (m, 3H), 7.50 (dd, J_1 = 7.9 Hz, J_2 = 1.7 Hz, 1H), 7.40-7.37 (m, 2H), 7.31-7.27 (m, 1H), 7.20-7.11 (m, 2H), 7.00 (d, *J* = 8.1 Hz, 1H) 2.76 (s, 3H), 1.37 (m, 9 H). **^{13}C NMR (100 MHz, CDCl$_3$)** δ 169.5, 168.8, 149.7, 138.8, 138.7, 137.2, 136.8, 133.9, 133.0, 131.9, 130.0, 129.3, 129.2, 128.8, 128.3, 128.0, 127.6, 126.8, 124.8, 121.6, 35.7, 31.8, 17.9. **HRMS (ESI)** calculated $[M+Na]^+$ for $C_{27}H_{25}NO_2Na$: 418.1778, found: 418.1784. **FTIR (cm^{-1})** 3020, 2964, 1712, 1488, 1375, 1212, 1100, 925, 747.

(*P*)-Methyl-(*E*)-3-(3-(*tert*-butyl)-4-(4-methyl-1,3-dioxoisoindolin-2-yl)phenyl)acrylate (2k)

Following the general procedure, K_2CO_3 (52 mg, 0.375 mmol) and (*E*)-2-((2-(*tert*-butyl)-4-(3-methoxy-3-oxoprop-1-en-1-yl)phenyl)carbamoyl)-6-methylbenzoic acid **1k** (98.8 mg, 0.25

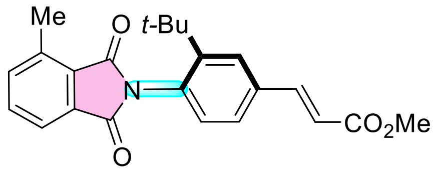

2k

mmol) were taken in THF (2.0 mL), and then PivCl (46 μL, 0.375 mmol) was added to the reaction mixture, and it was stirred at 25 °C for 30 min (until the full conversion of **1k**, monitored by TLC). Then, the triazolium salt **3** (9.2 mg, 0.025 mmol, 10 mol %) and THF (1.0 mL) were successively added and stirred for 24 h. Then the reaction mixture was purified by flash column chromatography (Pet. ether- EtOAc: 95:5) to (*P*) methyl (*E*)-3-(3-(*tert*-butyl)-4-(4-methyl-1,3-dioxoisoindolin-2-yl)phenyl)acrylate (**2k**) as a light-yellow solid (84.0 mg, 89% yield).

Rf (Pet. ether /EtOAc = 90/10): 0.50; er = 97:3, $[\alpha]_D^{25}$ = -10.9 (c 1.0, $CHCl_3$). **HPLC** (CHIRALPAK IA, *n*-hexane/IPA = 98:2, flow rate = 1.0 mL/min, λ = 254 nm) t_R = 22.7 min (major), 25.7 min (minor).

^{1}H NMR (400 MHz, $CDCl_3$) δ 7.78 (d, *J* = 7.5 Hz, 1H), 7.74-7.70 (m, 2H), 7.64 (t, *J* = 7.8 Hz, 1H), 7.54 (d, *J* = 7.6 Hz, 1H), 7.48 (dd, J_1 = 8.0 Hz, J_2 = 1.7 Hz, 1H), 7.02(d, *J* = Hz, 1H), 6.47 (d, *J* = 15.9 Hz, 1H), 3.82 (s, 3H), 2.74 (s, 3H), 1.33 (m, 9H). **^{13}C NMR (100 MHz, $CDCl_3$)** δ 169.3, 168.6, 167.3, 150.3, 144.3, 138.8, 136.9, 135.7, 134.0, 132.9, 132.3, 132.0, 129.2, 129.1, 126.2, 121.6, 119.1, 51.9, 35.8, 31.6, 17.8. **HRMS (ESI)** calculated $[M+Na]^+$ for $C_{23}H_{23}NO_4Na$: 400.1519, found: 400.1523. **FTIR (cm^{-1})** 3020, 2961, 1712, 1485, 1374, 1212, 1100, 927, 747.

(*P*)-2-(2-(*tert*-Butyl)-4-(phenylethynyl)phenyl)-4-methylisoindoline-1,3-dione (2l)

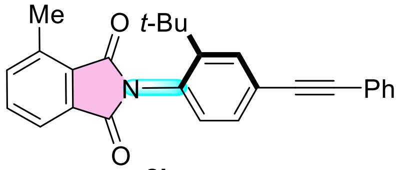

2l

Following the general procedure, K_2CO_3 (52 mg, 0.375 mmol) and 2-((2-(*tert*-butyl)-4-(phenylethynyl)phenyl)carbamoyl)-6-methylbenzoic acid **1l** (102.8 mg, 0.25 mmol) were taken in THF (2.0 mL), and then PivCl (46 μL, 0.375 mmol) was added to the reaction mixture, and it was stirred at 25 °C for 30 min (until the full conversion of **1l**, monitored by TLC). Then, the triazolium salt **3** (9.2 mg, 0.025 mmol, 10 mol %) and THF (1.0 mL) were successively added and stirred for 24 h. Then the reaction mixture was purified by flash column chromatography (Pet. ether- EtOAc: 95:5) to afford *(P)*-2-(2-(*tert*-butyl)-4-(phenylethynyl)phenyl)-4-methylisoindoline-1,3-dione **2l** as a light-yellow solid (98.0 mg, 99% yield).

Rf (Pet. ether /EtOAc = 90/10): 0.33; er = 97:3, $[\alpha]_D^{25}$ = -10.4 (c 1.0, $CHCl_3$). **HPLC** (CHIRALPAK IA, *n*-hexane/IPA = 98:2, flow rate = 1.0 mL/min, λ = 254 nm) t_R = 8.9 min (minor), 9.7 min (major).

^{1}H NMR (400 MHz, CDCl$_3$) δ 7.80-7.78 (m, 2H), 7.65 (t, *J* = 7.5 Hz, 1H), 7.59-7.54 (m, 3H), 7.47 (dd, J_1 = 8.0 Hz, J_2 = 1.9 Hz, 1H), 7.39-7.36 (m, 3H), 7.01 (d, *J* = 8.0 Hz, 1H), 2.75 (s, 3H), 1.35 (s, 9H). **^{13}C NMR (100 MHz, CDCl$_3$)** δ 169.3, 168.6, 149.8, 138.7, 136.9, 134.0, 132.9, 132.2, 131.8, 131.8, 130.3, 130.3, 129.6, 128.6, 128.5, 124.9, 123.1, 121.6, 90.3, 89.1, 35.8, 31.7, 17.9. **HRMS (ESI)** calculated $[M+Na]^+$ for $C_{27}H_{23}NO_2Na$: 416.1621, found: 416.1624. **FTIR (cm^{-1})** 2963, 2922, 1772, 1712, 1602, 1493, 1371, 1262, 1099, 749.

(*P*)-2-(2-(*tert*-Butyl)-5-nitrophenyl)-4-methylisoindoline-1,3-dione (2m)

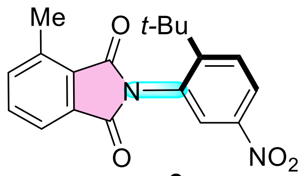

2m

Following the general procedure, K_2CO_3 (52 mg, 0.375 mmol) and 2-((2-(*tert*-butyl)-5-nitrophenyl)carbamoyl)-6-methylbenzoic acid **1m** (89.1 mg, 0.25 mmol) were taken in THF (2.0 mL), and then PivCl (46 μL, 0.375 mmol) was added to the reaction mixture, and it was stirred at 25 °C for 30 min (until the full conversion of **1m**, monitored by TLC). Then, the triazolium salt **3** (9.2 mg, 0.025 mmol, 10 mol %) and THF (1.0 mL) were successively added and stirred for 24 h. Then the reaction mixture was purified by flash column chromatography (Pet. ether- EtOAc: 95:5) to (*P*)-2-(2-(*tert*-butyl)-5-nitrophenyl)-4-methylisoindoline-1,3-dione **2m** as a light-yellow solid (84.6 mg, 99% yield).

Rf (Pet. ether /EtOAc = 90/10): 0.50; er = 97:3, $[\alpha]_D^{25}$ = -26.1 (c 1.0, $CHCl_3$). **HPLC** (CHIRALPAK IF, *n*-hexane/IPA = 95:5, flow rate = 1.0 mL/min, λ = 254 nm) t_R = 14.0 min (major), 15.4 min (minor).

^{1}H NMR (400 MHz, CDCl$_3$) δ 8.25 (dd, J_1 =2.5 Hz, J_2 = 8.9 Hz, 1H), 7.92 (d, *J* = 2.5 Hz, 1H), 7.82-7.79 (m, 2H), 7.68 (t, *J* = 7.6 Hz, 1H), 7.58 (d, *J* = 7.8 Hz, 1H), 2.74 (s, 3H), 1.36 (m, 9H). **^{13}C NMR (100 MHz, CDCl$_3$)** δ 168.9, 168.2, 157.8, 146.6, 139.2, 137.2, 134.4, 132.6, 131.4, 130.0, 128.9, 127.2, 124.3, 121.9, 36.6, 31.5, 17.9. **HRMS (ESI)** calculated $[M+H]^+$ for $C_{19}H_{19}N_2O_4$: 339.1339, found: 339.1342. **FTIR (cm^{-1})** 2964, 2924, 1715, 1521, 1483, 1346, 1199, 745.

(*P*)-4-Methyl-2-(4-methyl-2-(2-phenylpropan-2-yl)phenyl)isoindoline-1,3-dione (2n)

Following the general procedure, K_2CO_3 (52 mg, 0.375 mmol) and 2-methyl-6-((4-methyl-2-(2-phenylpropan-2-yl)phenyl)carbamoyl)benzoic acid **1n** (96.9 mg, 0.25 mmol) were taken in

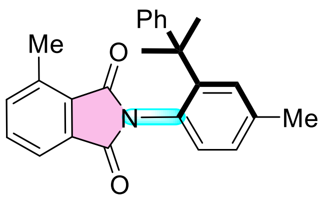

2n

THF (2.0 mL), and then PivCl (46 μL, 0.375 mmol) was added to the reaction mixture, and it was stirred at 25 °C for 30 min (until the full conversion of **1n**, monitored by TLC). Then, the triazolium salt **3** (9.2 mg, 0.025 mmol, 10 mol %) and THF (1.0 mL) were successively added and stirred for 24 h. Then the reaction mixture was purified by flash column chromatography (Pet. ether- EtOAc: 95:5) to (*P*)-4-methyl-2-(4-methyl-2-(2-phenylpropan-2-yl)phenyl)isoindoline-1,3-dione **2n** as a light-yellow solid (58.3 mg, 63% yield).

Rf (Pet. ether /EtOAc = 90/10): 0.50; er = 96:4, $[\alpha]_D^{25}$ = -3.2 (c 1.0, $CHCl_3$). **HPLC** (CHIRALPAK IA, *n*-hexane/IPA = 98:2, flow rate = 1.0 mL/min, λ = 254 nm) t_R = 6.6 min (minor), 9.5 min (major).

^{1}H NMR (400 MHz, $CDCl_3$) δ 7.55-7.48 (m, 3H), 7.39-7.37 (m, 1H), 7.16-7.14 (m, 1H), 7.08-7.06 (m, 2H), 6.95-6.92 (m, 2H), 6.85-6.81 (m, 2H), 2.53 (s, 3H), 2.45 (s, 3H), 1.68 (m, 3H), 1.66 (m, 3H). **^{13}C NMR (100 MHz, $CDCl_3$)** δ 168.0, 168.0, 148.8, 148.5, 139.6, 137.7, 136.0, 133.2, 132.5, 131.4, 129.5, 128.8, 128.4, 128.0, 127.4, 126.3, 125.2, 120.8, 42.8, 32.2, 30.9, 21.8, 17.7. **HRMS (ESI)** calculated $[M+H]^+$ for $C_{25}H_{24}NO_2$: 370.1802, found: 370.1808. **FTIR (cm^{-1})** 2963, 2922, 1716, 1520, 1488, 1352, 1210, 748.

(*P*)-2-(2-(1-Methoxy-2-methylpropan-2-yl)-4-methylphenyl)-4-methylisoindoline-1,3-dione (2o)

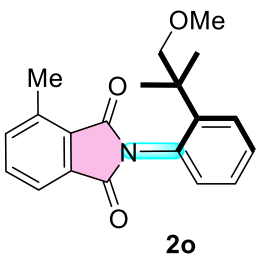

2o

Following the general procedure, K_2CO_3 (52 mg, 0.375 mmol) and 2-methyl-6-((4-methyl-2-(2-phenylpropan-2-yl)phenyl)carbamoyl) benzoic acid **1o** (96.9 mg, 0.25 mmol) were taken in THF (2.0 mL), and then PivCl (46 μL, 0.375 mmol) was added to the reaction mixture, and it was stirred at 25 °C for 30 min (until the full conversion of **1o**, monitored by TLC). Then, the triazolium salt **3** (9.2 mg, 0.025 mmol, 10 mol %) and THF (1.0 mL) were successively added and stirred for 24 h. Then the reaction mixture was purified by flash column chromatography (Pet. ether- EtOAc: 95:5) to (*P*)-2-(2-(1-methoxy-2-methylpropan-2-yl)-4-methylphenyl)-4-methylisoindoline-1,3-dione (**2o**) as a light-yellow solid (79.9 mg, 99% yield).

Rf (Pet. ether /EtOAc = 90/10): 0.48; er = 94:6, $[\alpha]_D^{25}$ = -1.8 (c 1.0, $CHCl_3$). **HPLC** (CHIRALPAK IA, *n*-hexane/IPA = 98:2, flow rate = 1.0 mL/min, λ = 254 nm) t_R = 7.7 min (minor), 8.8 min (major).

^{1}H NMR (400 MHz, CDCl$_3$) δ 7.79 (d, J = 7.4 Hz, 1H), 7.66-7.61 (m, 2H), 7.54 (d, J = 7.6 Hz, 1H), 7.45-7.41 (m, 1H), 7.36-7.30 (m, 1H), 7.01 (dd, J_1 = 7.0 Hz, J_2 =1.5 Hz, 1H), 3.39 (s, 2H), 3.24 (s, 3H), 2.75 (s, 3H), 1.33 (m, 6H). **^{13}C NMR (100 MHz, CDCl$_3$)** δ 169.4, 168.7, 146.3, 138.6, 136.8, 133.9, 132.9, 131.6, 130.5, 129.8, 129.7, 129.2, 127.7, 121.5, 82.1, 59.3, 40.1, 26.7, 26.7, 17.8. **HRMS (ESI)** calculated [M+Na]$^+$ for $C_{20}H_{21}NO_3Na$: 346.1414, found: 346.1421. **FTIR (cm^{-1})** 2973, 2925, 1714, 1486, 1376, 1196, 1104, 745.

(*P*)- 2-(2-(methoxydiphenylmethyl)phenyl)-4-methylisoindoline-1,3-dione (2p)

Following the general procedure, K_2CO_3 (26 mg, 0.1875 mmol) and 2-((2-(methoxydiphenylmethyl)phenyl)carbamoyl)-6-methylbenzoic acid **1p** (59.9 mg, 0.125 mmol) were taken in THF (1.0 mL), and then PivCl (23 µL, 0.1875 mmol) was added to the reaction mixture, and it was stirred at 25 °C for 30 min (until the full conversion of **1p**, monitored by TLC). Then, the triazolium salt **3** (0.0046 g, 0.0125 mmol, 10 mol %) and THF (0.5 mL) were successively added and stirred for 24 h. Then the reaction mixture was purified by flash column chromatography (Pet. ether- EtOAc: 95:5) to (*P*)-2-(2-(methoxydiphenylmethyl)phenyl)-4-methylisoindoline-1,3-dione **2p** as a light-yellow solid (54.0 mg, 99% yield).

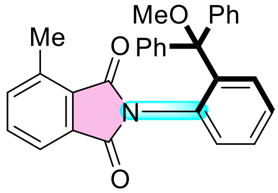

2p

R$_f$ (Pet. ether /EtOAc = 90/10): 0.40; er = >99:1, $[\alpha]_D^{25}$ = +15.1 (c 1.0, CHCl$_3$). **HPLC** (CHIRALCELL OD-H, *n*-hexane/IPA = 95:5, flow rate = 1.0 mL/min, λ = 254 nm) t_R = 10.0 min (major), 14.2 min (minor).

^{1}H NMR (400 MHz, CDCl$_3$) 7.61 – 7.57 (m, 2H), 7.54 – 7.39 (m, 6H), 7.36 – 7.34 (m, 2H), 7.21-7.17 (m, 3H), 7.11 – 7.06 (m, 3H), 7.02-6.99 (m, 1H), 2.99 (s, 3H), 2.59 (s, 3H). **^{13}C NMR (100 MHz, CDCl$_3$)** δ 167.9, 167.5, 144.1, 143.4, 142.1, 137.6, 135.9, 133.2, 133.1, 132.9, 132.6, 132.1, 129.1, 129.1, 128.4, 128.3, 127.9, 127.8, 127.7, 126.8, 126.4, 120.8, 87.1, 52.7, 17.7. **HRMS (ESI)** calculated [M+Na]$^+$ for $C_{29}H_{23}NO_3Na$: 456.1570, found: 456.1577. **FTIR (cm^{-1})** 3021, 2927, 2829, 1712, 1484, 1444, 1376, 1196, 743.

(*P*)-2-(2-(Di(thiophen-2-yl)((trimethylsilyl)oxy)methyl)phenyl)-4-methylisoindoline-1,3-dione (2q)

Following the general procedure, K_2CO_3 (26 mg, 0.1875 mmol) and 2-((2-(di(thiophen-2-yl)((trimethylsilyl)oxy)methyl)phenyl)carbamoyl)-6-methylbenzoic acid **1q** (65.2 mg, 0.125 mmol) were taken in THF (1.0 mL), and then PivCl (23 µL, 0.1875 mmol) was added to the reaction mixture, and it was stirred at 25 °C for 30 min (until the full conversion of **1q**,

monitored by TLC). Then, the triazolium salt **3** (0.0046 g, 0.0125 mmol, 10 mol %) and THF (0.5 mL) were successively added and stirred for 24 h. Then the reaction mixture was purified by flash column chromatography (Pet. ether- EtOAc: 95:5) to (*P*)-2-(2-(di(thiophen-2-yl)((trimethylsilyl)oxy)methyl)phenyl)-4-methylisoindoline-1,3-dione **2q** as a light-yellow solid (28.3 mg, 45% yield).

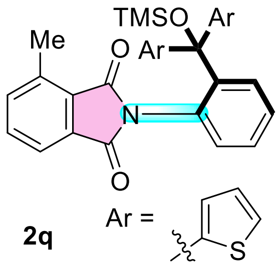

2q

R_f (Pet. ether /EtOAc = 90/10): 0.40; er = 97:3, $[\alpha]_D^{25}$ = -18.2 (c 1.0, $CHCl_3$). **HPLC** (CHIRALPAK IA, *n*-hexane/IPA = 95:5, flow rate = 1.0 mL/min, λ = 254 nm) t_R = 6.6 min (major), 7.6 min (minor).
^{1}H NMR (400 MHz, $CDCl_3$) δ 7.67 (d, *J* = 7.3 Hz, 1H), 7.57 (t, *J* = 7.5 Hz, 1H), 7.48-7.45 (m, 2H), 7.39 -7.37 (m, 2H), 7.20-7.19 (m, 2H), 7.11 – 7.09 (m, 1H), 6.93-6.88 (m, 4H), 2.63 (s, 3H), -0.29 (s, 9H). **^{13}C NMR (100 MHz, $CDCl_3$)** δ 167.8, 167.3, 151.6, 151.4, 146.5, 137.9, 136.1, 133.4, 133.3, 132.0, 130.4, 130.2, 129.5, 129.0, 128.9, 127.9, 127.9, 126.8, 126.7, 125.4, 125.2, 121.2, 80.8, 17.8, 1.3. **HRMS (ESI)** calculated $[M+K]^+$ for $C_{27}H_{25}NO_3S_2SiK$: 542.0677, found: 542.0682. **FTIR (cm^{-1})** 2954, 2923, 2853, 1712, 1485, 1442, 1380, 1245, 1106, 753.

(*P*)-4-Methyl-2-(2-(phenylsulfonyl)phenyl)isoindoline-1,3-dione (2r)

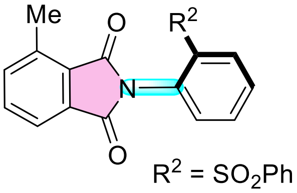

2r

Following the general procedure, K_2CO_3 (26 mg, 0.188 mmol) and 2-((2-(*tert*-butyl)-4-chlorophenyl)carbamoyl)-6-methylbenzoic acid **1r** (49.4 mg, 0.125 mmol) were taken in THF (1.0 mL), and then PivCl (23 µL, 0.188 mmol) was added to the reaction mixture, and it was stirred at 25 °C for 30 min (until the full conversion of **1r**, monitored by TLC). Then, the triazolium salt **3** (0.0046 g, 0.0125 mmol, 10 mol %) and THF (0.5 mL) were successively added and stirred for 24 h. Then the reaction mixture was purified by flash column chromatography (Pet. ether- EtOAc: 70:30) to (*P*)-4-methyl-2-(2-(phenylsulfonyl)phenyl) isoindoline-1,3-dione (**2r**) as a light-yellow solid (33.6 mg, 71% yield).

R_f (Pet. ether /EtOAc = 70/30): 0.35; er = 80:20, $[\alpha]_D^{22}$ = -4.4 (c 1.0, $CHCl_3$). **HPLC** (CHIRALPAK IA, *n*-hexane/IPA = 70:30, flow rate = 1.0 mL/min, λ = 254 nm) t_R = 12.2 min (major), 14.1 min (minor).

^{1}H NMR (400 MHz, $CDCl_3$) δ 8.30 (dd, J_1 = 7.7 Hz, J_2 =1.7 Hz, 1H), 7.75 – 7.64 (m, 6H), 7.56-7.53 (m, 2H), 7.38-7.34 (m, 2H), 7.31-7.29 (m, 1H), 2.64 (s, 3H). **^{13}C NMR (100 MHz, $CDCl_3$)** δ 167.4, 166.9, 140.5, 140.0, 138.7, 136.8, 134.8, 134.1, 133.5, 132.6, 131.1, 130.8,

130.5, 129.2, 128.9, 127.9, 121.7, 17.8. **HRMS (ESI)** calculated $[M+H]^+$ for $C_{21}H_{16}NO_4S$: 378.0795, found: 378.0799. **FTIR (cm^{-1})** 2922, 2854, 1718, 1478, 1445, 1376, 1155, 1096, 732.

(*P*)-2-(2-(*tert*-Butyl)phenyl)-4-methoxyisoindoline-1,3-dione (2s)

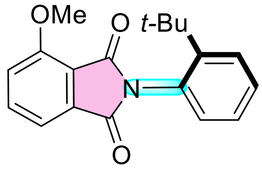

2s

Following the general procedure, K_2CO_3 (52 mg, 0.375 mmol) and 2-((2-(*tert*-butyl)phenyl)carbamoyl)-6-methoxybenzoic acid **1s** (81.8 mg, 0.25 mmol) were taken in THF (2.0 mL), and then PivCl (46 μL, 0.375 mmol) was added to the reaction mixture, and it was stirred at 25 °C for 30 min (until the full conversion of **1s**, monitored by TLC). Then, the triazolium salt **3** (9.2 mg, 0.025 mmol, 10 mol %) and THF (1.0 mL) were successively added and stirred for 24 h. Then the reaction mixture was purified by flash column chromatography (Pet. ether- EtOAc: 95:5) to (*P*)-2-(2-(*tert*-butyl)phenyl)-4-methoxyisoindoline-1,3-dione (**2s**) as a light-yellow solid (77.6 mg, 99% yield).

R_f (Pet. ether /EtOAc = 90/10): 0.46; er = 95:5, $[\alpha]_D^{25}$ = -1.9 (c 1.0, $CHCl_3$). **HPLC** (CHIRALCELL OD-H, *n*-hexane/IPA = 90:10, flow rate = 1.0 mL/min, λ = 254 nm) t_R = 16.2 min (minor), 21.0 min (major).

^{1}H NMR (400 MHz, $CDCl_3$) δ 7.75-7.71 (m, 1H), 7.61 (dd, J_1 = 8.0 Hz, J_2 = 1.1 Hz, 1H), 7.54 (d, J = 7.6 Hz, 1H) 7.43-7.39 (m, 1H), 7.31-7.27 (m, 2H), 6.98 (dd, J_1 = 7.7 Hz, J_2 = 1.5 Hz, 1H), 4.04 (s, 3H), 1.32 (s, 9H). **^{13}C NMR (100 MHz, $CDCl_3$)** δ 168.5, 167.3, 157.1, 149.5, 136.6, 134.6, 131.6, 130.0, 129.8, 128.7, 127.3, 117.8, 117.8, 116.0, 56.5, 35.7, 31.8. **HRMS (ESI)** calculated $[M+H]^+$ for $C_{19}H_{20}NO_3$: 310.1438, found: 310.1443. **FTIR (cm^{-1})** 2962, 2924, 1711, 1610, 1485, 1379, 1283, 1219, 1048, 768.

(*P*)-4-(Benzyloxy)-2-(2-(*tert*-butyl)phenyl)isoindoline-1,3-dione (2t)

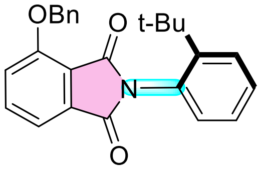

2t

Following the general procedure, K_2CO_3 (52 mg, 0.375 mmol) 2-(benzyloxy)-6-((2-(*tert*-butyl)phenyl)carbamoyl)benzoic acid **1t** (100.8 mg, 0.25 mmol) were taken in THF (2.0 mL), and then PivCl (46 μL, 0.375 mmol) was added to the reaction mixture, and it was stirred at 25 °C for 30 min (until the full conversion of **1t**, monitored by TLC). Then, the triazolium salt **3** (9.2 mg, 0.025 mmol, 10 mol %) and THF (1.0 mL) were successively added and stirred for 24 h. Then the reaction mixture was purified by flash column

chromatography (Pet. ether- EtOAc: 95:5) to afford (*P)*-4-(benzyloxy)-2-(2-(*tert*-butyl)phenyl)isoindoline-1,3-dione **2t** as a light yellow solid (95.5 mg, 99% yield).

R_f (Pet. ether /EtOAc = 90/10): 0.25; er = 95:5, $[\alpha]_D^{25}$ = +11.1 (c 1.0, $CHCl_3$). **HPLC** (CHIRALPAK IA, *n*-hexane/IPA = 98:2, flow rate = 1.0 mL/min, λ = 254 nm) t_R = 16.9 min (minor), 20.9 min (major).

^{1}H NMR (400 MHz, $CDCl_3$) δ 7.68 (t, *J* = 7.8 Hz, 1H), 7.62 (dd, J_1 = 8.1, J_2 = 1.2 Hz, 1H), 7.53 (dd, J_1 = 10.4 Hz, J_2 = 1.4 Hz, 3H), 7.44-7.37 (m, 3H), 7.32-7.26 (m, 3H), 7.00 (dd, J_1 = 7.7 Hz, J_2 = 1.4 Hz, 1H), 5.38 (t, *J* =13.2 Hz, 2H), 1.34 (s, 9H). **^{13}C NMR (100 MHz, $CDCl_3$)** δ 168.5, 167.1, 156.3, 149.6, 136.4, 136.0, 134.8, 131.6, 130.1, 129.8, 128.9, 128.8, 128.3, 127.4, 126.9, 120.0, 118.7, 116.5, 71.2, 35.8, 31.9**. HRMS (ESI)** calculated $[M+Na]^+$ for $C_{25}H_{23}NO_3Na$: 408.1570, found: 408.1578. **FTIR (cm^{-1})** 2960, 2923, 2858, 1773, 1713, 1610, 1484, 1446, 1378, 749.

(*P*)-4-(Allyloxy)-2-(2-(*tert*-butyl)phenyl)isoindoline-1,3-dione (2u)

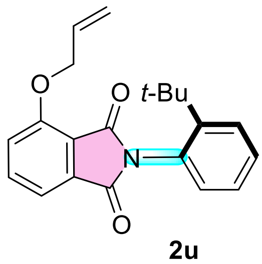

2u

Following the general procedure, K_2CO_3 (52 mg, 0.375 mmol) and 2-(allyloxy)-6-((2-(*tert*-butyl)phenyl)carbamoyl)benzoic acid **1u** (88.3 mg, 0.25 mmol) were taken in THF (2.0 mL), and then PivCl (46 μL, 0.375 mmol) was added to the reaction mixture, and it was stirred at 25 °C for 30 min (until the full conversion of **1u,** monitored by TLC). Then, the triazolium salt **3** (9.2 mg, 0.025 mmol, 10 mol %) and THF (1.0 mL) were successively added and stirred for 24 h. Then the reaction mixture was purified by flash column chromatography (Pet. ether- EtOAc: 95:5) to afford (*P)*-4-(allyloxy)-2-(2-(*tert*-butyl)phenyl)isoindoline-1,3-dione **2u** as a light-yellow solid (83.0 mg, 99% yield).

R_f (Pet. ether /EtOAc = 90/10): 0.2; er = 98:2, $[\alpha]_D^{25}$ = -7.5 (c 1.0, $CHCl_3$). **HPLC** (CHIRALPAK IC, *n*-hexane/IPA = 95:5, flow rate = 1.0 mL/min, λ = 254 nm) t_R = 16.0 min (minor), 24.7 min (major).

^{1}H NMR (400 MHz, $CDCl_3$) δ 7.69 (t, *J* = 7.8 Hz, 1H), 7.61 (dd, J_1 = 6.7 Hz, J_2 = 1.3 Hz, 1H), 7.54 (d, *J* = 7.1 Hz, 1H), 7.42 (dt, J_1 = 7.4 Hz, J_2 = 1.4 Hz, 1H), 7.29-7.25 (m, 2H), 6.98 (dd, J_1 = 7.7 Hz, J_2 = 1.4 Hz, 1H), 6.12-6.03 (m, 1H), 5.55 (dd, J_1 = 17.3 Hz, J_2 = 1.4 Hz, 1H), 5.35 (dd, J_1 = 10.6 Hz, J_2 = 1.3 Hz, 1H), 4.8 (d, *J* = 4.8 Hz, 2H), 1.33 (s, 9H). **^{13}C NMR (100 MHz, $CDCl_3$)** δ 168.5, 167.1, 156.2, 149.5, 136.3, 134.7, 132.0, 131.6, 130.1, 129.8, 128.7, 127.3, 119.6, 118.5, 118.3, 116.2, 70.1, 35.7, 31.8. **HRMS (ESI)** calculated $[M+Na]^+$ for

$C_{21}H_{21}NO_3Na$: 358.1414, found: 358.1420. **FTIR (cm^{-1})** 2962, 1774, 1714, 1610, 1484, 1445, 1380, 1283, 1102, 753.

(*P*)- 2-(2-(*tert*-Butyl)phenyl)-1,3-dioxoisoindolin-4-yl pivalate (2v)

Following the general procedure, K_2CO_3 (86.4 mg, 0.625 mmol) and 2-((2-(*tert*-butyl)phenyl)carbamoyl)-6-hydroxybenzoic acid **1v** (78.3 mg, 0.25 mmol) were taken in THF (2.0 mL), and then PivCl (77 µL, 0.625 mmol) was added to the reaction mixture, and it was stirred at 25 °C for 30 min (until the full conversion of **1v**, monitored by TLC). Then, the triazolium salt **3** (0.0092 g, 0.025 mmol, 10 mol %) and THF (1.0 mL) were successively added and stirred for 24 h. Then the reaction mixture was purified by flash column chromatography (Pet. ether- EtOAc: 95:5) to afford (*P*)-2-(2-(*tert*-butyl)phenyl)-1,3-dioxoisoindolin-4-yl pivalate **2v** as a light yellow liquid (89.7 mg, 95% yield).

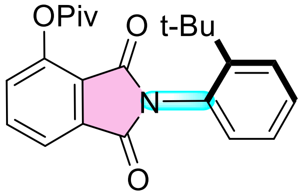

2v

R$_f$ (Pet. ether /EtOAc = 90/10): 0.42; er = 95:5, $[\alpha]_D^{25}$ = +9.08 (c 1.0, $CHCl_3$). **HPLC** (CHIRALCELL OD-H, *n*-hexane/IPA = 99:1, flow rate = 0.7 mL/min, λ = 254 nm) t_R = 13.9 min (minor), 15.0 min (major).

^{1}H NMR (400 MHz, CDCl$_3$) δ 7.84-7.77 (m, 2H), 7.63 (d, *J* = 8.1 Hz, 1H), 7.43-7.40 (m, 2H), 7.30 (t, *J* = 7.4 Hz, 1H), 7.02 (d, *J* = 7.7 Hz, 1H), 1.42 (s, 9H), 1.33 (s, 9H). **^{13}C NMR (100 MHz, CDCl$_3$)** δ 176.0, 167.9, 166.1, 149.4, 147.6, 135.9, 134.0, 131.5, 130.0, 129.5, 128.9, 128.9, 127.4, 123.5, 121.3, 39.3, 35.7, 31.7, 27.2. **HRMS (ESI)** calculated $[M+Na]^+$ for $C_{23}H_{25}NO4Na$: 402.1676, found: 402.1682. **FTIR (cm^{-1})** 2962, 2920, 2823, 1778, 1711, 1589, 1480, 1432, 1255, 752.

(*P*)-2-(2-(*tert*-Butyl)phenyl)-4-chloroisoindoline-1,3-dione (2w)

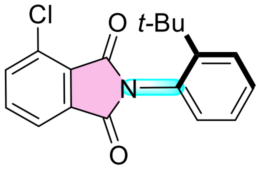

2w

Following the general procedure, K_2CO_3 (52 mg, 0.375 mmol) and 2-((2-(*tert*-butyl)phenyl)carbamoyl)-6-chlorobenzoic acid **1w** (83.0 mg, 0.25 mmol) were taken in THF (2.0 mL), and then PivCl (46 µL, 0.375 mmol) was added to the reaction mixture, and it was stirred at 25 °C for 30 min (until the full conversion of **1w**, monitored by TLC). Then, the triazolium salt **3** (9.2 mg, 0.025 mmol, 10 mol %) and THF (1.0 mL) were successively added and stirred for 24 h. Then the reaction mixture was purified by flash column chromatography (Pet. ether- EtOAc: 95:5) to (*P*)-2-(2-(*tert*-butyl)phenyl)-4-chloroisoindoline-1,3-dione (**2w**) as a light-yellow solid (78.2 mg, 99% yield).

R_f (Pet. ether /EtOAc = 90/10): 0.52; er = 97:3, $[\alpha]_D^{25}$ = -5.8 (c 1.0, $CHCl_3$). **HPLC** (CHIRALPAK IA, *n*-hexane/IPA = 98:2, flow rate = 1.0 mL/min, λ = 254 nm) t_R = 8.6 min (minor), 9.3 min (major).

^{1}H NMR (400 MHz, $CDCl_3$) δ 7.89-7.85 (m, 1H), 7.73-7.69 (m, 2H), 7.63 (dd, J_1 = 8.1 Hz, J_2 = 1.1 Hz, 1H), 7.46-7.42 (m, 1H), 7.33-7.29 (m, 1H), 6.99 (dd, J_1 = 7.7 Hz, J_2 = 1.2 Hz, 1H), 1.32 (s, 9H). **^{13}C NMR (100 MHz, $CDCl_3$)** δ 167.3, 166.2, 149.4, 136.2, 135.3, 134.5, 132.0, 131.4, 130.0, 129.6, 128.9, 128.2, 127.4, 122.4, 35.7, 31.8. **HRMS (ESI)** calculated $[M+Na]^+$ for $C_{18}H_{16}ClNO_2Na$: 336.0762, found: 336.0768. **FTIR (cm^{-1})** 2960, 2920, 1717, 1597, 1487, 1370, 1195, 1104, 747.

(*P*)-4-Bromo-2-(2-(*tert*-butyl)phenyl)isoindoline-1,3-dione (2x)

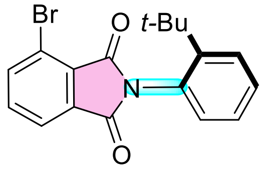

2x

Following the general procedure, K_2CO_3 (52 mg, 0.375 mmol) and 2-bromo-6-((2-(*tert*-butyl)phenyl)carbamoyl)benzoic acid **1x** (94.1 mg, 0.25 mmol) were taken in THF (2.0 mL), and then PivCl (46 μL, 0.375 mmol) was added to the reaction mixture, and it was stirred at 25 °C for 30 min (until the full conversion of **1x**, monitored by TLC). Then, the triazolium salt **3** (9.2 mg, 0.025 mmol, 10 mol %) and THF (1.0 mL) were successively added and stirred for 24 h. Then the reaction mixture was purified by flash column chromatography (Pet. Ether- EtOAc: 95:5) to (*P*) 4-bromo-2-(2-(*tert*-butyl)phenyl)isoindoline-1,3-dione (**2x**) as a light-yellow solid (89.5 mg, 99% yield).

R_f (Pet. Ether /EtOAc = 90/10): 0.52; er = 97:3, $[\alpha]_D^{25}$ = -7.2 (c 1.0, $CHCl_3$). **HPLC** (CHIRALPAK IA, *n*-hexane/IPA = 98:2, flow rate = 1.0 mL/min, λ = 254 nm) t_R = 8.6 min (minor), 9.6 min (major).

^{1}H NMR (400 MHz, $CDCl_3$) δ 792-7.81 (m, 2H), 7.64-7.60 (m, 2H), 7.46-7.42 (m, 1H), 7.33-7.29 (m, 1H), 6.99 (dd, J_1 = 7.7 Hz, J_2 = 1.5 Hz, 1H), 1.32 (s, 9H). **^{13}C NMR (100 MHz, $CDCl_3$)** δ 167.1, 166.6, 149.4, 139.4, 135.3, 134.6, 131.4, 130.0, 130.0, 129.6, 128.9, 127.4, 122.9, 119.1, 35.7, 31.8. **HRMS (ESI)** calculated $[M+Na]^+$ for $C_{18}H_{16}BrNO_2Na$: 380.0257, found: 380.0264. **FTIR (cm^{-1})** 2962, 2923, 1718, 1595, 1488, 1372, 1196, 1113, 768.

(*P*)-2-(2-(*tert*-Butyl)phenyl)-4-nitroisoindoline-1,3-dione (2y)

Following the general procedure, K_2CO_3 (52 mg, 0.375 mmol) and 2-((2-(*tert*-butyl)phenyl)carbamoyl)-6-nitrobenzoic acid **1y** (85.6 mg, 0.25 mmol) were taken in THF (2.0 mL), and then PivCl (46 μL, 0.375 mmol) was added to the reaction mixture, and it was stirred

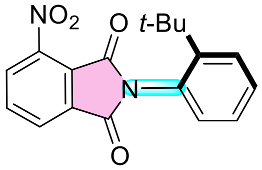

2y

at 25 °C for 30 min (until the full conversion of **1y**, monitored by TLC). Then, the triazolium salt **3** (9.2 mg, 0.025 mmol, 10 mol %) and THF (1.0 mL) were successively added and stirred for 24 h. Then the reaction mixture was purified by flash column chromatography (Pet. Ether-EtOAc: 95:5) to afford *(P)*-2-(2-(*tert*-butyl)phenyl)-4-nitroisoindoline-1,3-dione **2y** as a light yellow solid (80.0 mg, 99% yield).

R_f (Pet. Ether /EtOAc = 90/10): 0.1; er = 88:12, $[\alpha]_D^{25}$ = -47.4 (c 1.0, $CHCl_3$). **HPLC** (CHIRALPAK OD-H, *n*-hexane/IPA = 85:15, flow rate = 1.0 mL/min, λ = 254 nm) t_R = 22.8 min (minor), 19.1 min (major).

^{1}H NMR (400 MHz, CDCl$_3$) δ 8.21 (d, *J* = 7.5 Hz, 1H), 8.17 (d, *J* = 8.1 Hz, 1H), 7.98 (t, *J* = 7.8 Hz, 1H), 7.64 (dd, J_1 = 8.2 Hz, J_2 = 1.3 Hz, 1H), 7.45 (dt, J_1 = 7.3 Hz, J_2 = 1.3 Hz, 1H), 7.31 (dt, J_1 = 7.6 Hz, J_2 = 1.3 Hz, 1H), 7.00 (dd, J_1 = 7.8 Hz, J_2 = 1.3 Hz, 1H), 1.32 (s, 9H). **^{13}C NMR (100 MHz, CDCl$_3$)** δ 166.2, 163.2, 149.2, 145.5, 135.9, 135.8, 134.3, 131.3, 130.3, 129.1, 129.0, 127.6, 127.5, 124.0, 35.7, 31.8. **HRMS (ESI)** calculated $[M+Na]^+$ for $C_{18}H_{16}N_2O_4Na$: 347.1002, found: 347.1008. **FTIR (cm^{-1})** 2964, 2923, 1785, 1722, 1541, 1487, 1443, 1365, 1212, 761.

(*P*)- 2-(2-(*tert*-Butyl)phenyl)-1*H*-benzo[e]isoindole-1,3(2*H*)-dione (2z)

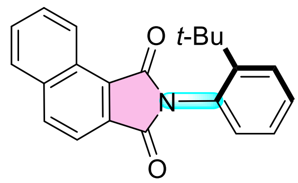

2z

Following the general procedure, K_2CO_3 (52 mg, 0.375 mmol) and 2-((2-(tert-butyl)phenyl)carbamoyl)-1-naphthoic acid **1z** (86.8 mg, 0.25 mmol) were taken in THF (2.0 mL), and then PivCl (46 μL, 0.375 mmol) was added to the reaction mixture, and it was stirred at 25 °C for 30 min (until the full conversion of **1z**, monitored by TLC). Then, the triazolium salt **3** (0.0092 g, 0.025 mmol, 10 mol %) and THF (1.0 mL) were successively added and stirred for 24 h. Then the reaction mixture was purified by flash column chromatography (Pet. Ether- EtOAc: 95:5) to afford *(P)*-2-(2-(*tert*-butyl)phenyl)-1*H*-benzo[e]isoindole-1,3(2*H*)-dione **2z** as a light-yellow solid (78.3 mg, 95% yield).

R_f (Pet. Ether /EtOAc = 90/10): 0.50; er = 96:4, $[\alpha]_D^{20}$ = -0.2 (c 1.0, $CHCl_3$). **HPLC** (CHIRALPAK IA, *n*-hexane/IPA = 98:2, flow rate = 1.0 mL/min, λ = 254 nm) t_R = 10.2 min (minor), 10.7 min (major).

^{1}H NMR (400 MHz, CDCl$_3$) δ 9.01 (d, J = 8.3 Hz, 1H), 8.25 (d, *J* = 8.2 Hz, 1H), 8.02-7.96 (m, 2H), 7.78-7.64 (m, 3H), 7.45 (t, *J* = 7.7 Hz, 1H), 7.33 (t, *J* = 7.5 Hz, 1H), 7.07 (d, *J* = 7.7 Hz, 1H), 1.36 (s, 9H). **^{13}C NMR (100 MHz, CDCl$_3$)** δ 170.0, 169.4, 149.8, 136.9, 135.5, 131.9,

131.7, 130.0, 129.9, 129.8, 129.1, 128.9, 128.8, 128.4, 127.9, 127.5, 125.4, 119.0, 35.7, 31.8. **HRMS (ESI)** calculated $[M+H]^+$ for $C_{22}H_{20}NO_2$: 3330.1489, found: 330.1499. **FTIR (cm^{-1})** 2959, 2860, 1711, 1488, 1444, 1368, 1253, 1101, 762.

(*P)*-2-(2-(*tert*-Butyl)phenyl)-4-methoxy-5-nitroisoindoline-1,3-dione (2aa)

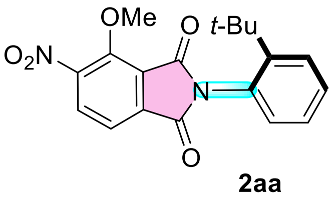

2aa

Following the general procedure, K_2CO_3 (52 mg, 0.375 mmol) and 6-((2-(*tert*-butyl)phenyl)carbamoyl)-2-methoxy-3-nitrobenzoic acid **1aa** (93.0 mg, 0.25 mmol) were taken in THF (2.0 mL), and then PivCl (46 µL, 0.375 mmol) was added to the reaction mixture, and it was stirred at 25 °C for 30 min (until the full conversion of **1aa**, monitored by TLC). Then, the triazolium salt **3** (0.0092 g, 0.025 mmol, 10 mol %) and THF (1.0 mL) were successively added and stirred for 24 h. Then the reaction mixture was purified by flash column chromatography (Pet. Ether- EtOAc: 95:5) to afford *(P)*-2-(2-(*tert*-butyl)phenyl)-4-methoxy-5-nitroisoindoline-1,3-dione **2aa** as a light-yellow solid (81.9 mg, 92% yield).

R$_f$ (Pet. Ether /EtOAc = 80/20): 0.45; er = 96:4, $[\alpha]_D^{20}$ = -1.3 (c 1.0, $CHCl_3$). **HPLC** (CHIRALCELL OD-H, *n*-hexane/IPA = 90:10, flow rate = 1.0 mL/min, λ = 254 nm) t_R = 9.2 min (minor), 11.2 min (major).

^{1}H NMR (400 MHz, CDCl$_3$) δ 8.06 (d, *J* = 7.8 Hz, 1H), 7.75 (d, *J* = 7.8 Hz, 1H), 7.66-7.64 (m, 1H), 7.48-7.44 (m, 1H), 7.35-7.37 (m, 1H), 6.99 (dd, J_1 = 7.7 Hz, J_2 = 1.2 Hz, 1H), 4.33 (s, 3H), 1.32 (s, 9H). **^{13}C NMR (100 MHz, CDCl$_3$)** δ 166.4, 165.2, 150.9, 149.2, 148.7, 136.8, 131.2, 130.9, 130.4, 129.2, 129.1, 127.6, 123.1, 118.5, 64.9, 35.7, 31.8. **HRMS (ESI)** calculated $[M+H]^+$ for $C_{19}H_{19}N_2O_5$: 355.1288, found: 355.1297. **FTIR (cm^{-1})** 2961, 2923, 1718, 1616, 1480, 1415, 1366, 1190, 1023, 750.

(*P)*-2-(2-(*tert*-Butyl)phenyl)-4-methoxy-7-nitroisoindoline-1,3-dione (2ab)

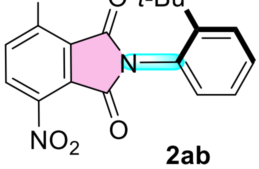

2ab

Following the general procedure, DABCO (23.0 mg, 0.188 mmol) and 2-((2-(*tert*-butyl)phenyl)carbamoyl)-6-methoxy-3-nitrobenzoic acid **1ab** (46.5 mg, 0.125 mmol) were taken in THF (1.0 mL), and then PivCl (23 µL, 0.188 mmol) was added to the reaction mixture, and it was stirred at -20 °C for 5 min (until the full conversion of **1ab**, monitored by TLC). Then, the triazolium salt **3** (0.0046 g, 0.025 mmol, 10 mol %) and THF (0.5 mL) were successively added and stirred for 24 h. Then the reaction mixture was purified by flash column chromatography

(Pet. Ether- EtOAc: 80:20) to afford (*P)*-2-(2-(*tert*-butyl)phenyl)-4-methoxy-7-nitroisoindoline-1,3-dione **2ab** as a light-yellow solid (41.1 mg, 93% yield).

Rf (Pet. Ether /EtOAc = 80/20): 0.32; er = 89:11, $[\alpha]_D^{20}$ = +9.36 (c 1.0, $CHCl_3$). **HPLC** (CHIRALPAK IA, *n*-hexane/IPA = 80:20, flow rate = 1.0 mL/min, λ = 254 nm) t_R = 14.8 min (minor), 16.2 min (major).

^{1}H NMR (400 MHz, $CDCl_3$) δ 8.17 (d, *J* = 9.1 Hz, 1H), 7.61 (d, *J* = 8.1 Hz, 1H), 7.42 (t, *J* = 7.7 Hz, 1H), 7.37 (d, *J* = 9.1 Hz, 1H), 7.28 (t, *J* = 7.6 Hz, 1H), 6.96 (d, *J* = 7.7 Hz, 1H), 4.10 (s, 3H), 1.32 (s, 9H). **^{13}C NMR (100 MHz, $CDCl_3$)** δ 164.9, 163.0, 159.8, 149.3, 138.6, 132.1, 131.4, 130.1, 129.2, 128.9, 127.4, 126.9, 119.0, 117.9, 57.3, 35.7, 31.8. **HRMS (ESI)** calculated $[M+H]^+$ for $C_{19}H_{19}N_2O_5$: 355.1288, found: 355.1308. **FTIR (cm^{-1})** 2923, 2855, 1725, 1604,1535, 1491, 1447, 1375, 1118, 767.

(*P)*-2-(2-(*tert*-butyl)phenyl)-5-phenyl-1*H*-benzo[*e*]isoindole-1,3(2*H*)-dione (2ac)

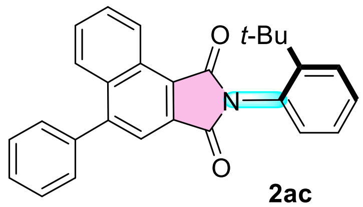

2ac

Following the general procedure, K_2CO_3 (26 mg, 0.188 mmol) and 2-((2-(*tert*-butyl)phenyl)carbamoyl)-4-phenyl-1-naphthoic acid **1ac** (52.8 mg, 0.125 mmol) were taken in THF (1.0 mL), and then PivCl (23 μL, 0.188 mmol) was added to the reaction mixture, and it was stirred at 25 °C for 30 min (until the full conversion of **1ac**, monitored by TLC). Then, the triazolium salt **3** (0.0046 g, 0.0125 mmol, 10 mol %) and THF (0.5 mL) were successively added and stirred for 24 h. Then the reaction mixture was purified by flash column chromatography (Pet. Ether- EtOAc: 95:5) to afford (*P)*-2-(2-(*tert*-butyl)phenyl)-5-phenyl-1*H*-benzo[*e*]isoindole-1,3(2*H*)-dione **2ac** as a light-yellow solid (34.4 mg, 68% yield).

Rf (Pet. Ether /EtOAc = 90/10): 0.40; er = 94:6, $[\alpha]_D^{20}$ = -2.6 (c 1.0, $CHCl_3$). **HPLC** (CHIRALPAK IA, *n*-hexane/IPA = 98:2, flow rate = 1.0 mL/min, λ = 254 nm) t_R = 7.0 min (major), 12.9 min (minor).

^{1}H NMR (400 MHz, $CDCl_3$) δ 9.11 (d, *J* = 8.3 Hz, 1H), 8.04 (d, J = 8.6 Hz, 1H), 7.93 (s, 1H), 7.78-7.74 (m, 1H), 7.97 – 7.61 (m, 2H), 7.58 – 7.48 (m, 5H), 7.46 – 7.44 (m, 1H), 7.36-7.32 (m, 1H), 7.09 (dd, J_1 = 7.7 Hz, J_2 = 1.3 Hz, 1H), 1.38 (s, 9H). **^{13}C NMR (100 MHz, $CDCl_3$)** δ 169.9, 169.3, 149.7, 148.2, 139.3, 135.0, 131.6, 131.2, 130.0, 129.8, 129.4, 128.9, 128.9, 128.7, 128.7, 128.5, 127.4, 127.3, 126.6, 125.5, 119.9, 35.7, 31.7. **HRMS (ESI)** calculated $[M+H]^+$ for $C_{28}H_{24}NO_2$: 406.1802, found: 406.1806. **FTIR (cm^{-1})** 2959, 2923, 1709, 1489, 1442, 1372, 1255, 1101, 757.

(*P*)-6-(2-(*tert*-Butyl)phenyl)-5*H*-pyrrolo[3,4-b]pyridine-5,7(6*H*)-dione (2ad)

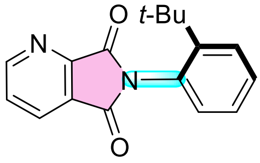

2ad

Following the general procedure, K_2CO_3 (52 mg, 0.375 mmol) and 3-((2-(*tert*-butyl)phenyl)carbamoyl)picolinic acid **1ad** (74.5 mg, 0.25 mmol) were taken in THF (2.0 mL), and then PivCl (46 µL, 0.375 mmol) was added to the reaction mixture, and it was stirred at 25 °C for 30 min (until the full conversion of **1ad**, monitored by TLC). Then, the triazolium salt **3** (9.2 mg, 0.025 mmol, 10 mol %) and THF (1.0 mL) were successively added and stirred for 24 h. Then the reaction mixture was purified by flash column chromatography (Pet. Ether- EtOAc: 95:5) to afford *(P)*-6-(2-(*tert*-butyl)phenyl)-5*H*-pyrrolo[3,4-b]pyridine-5,7(6*H*)-dione **2ad** as a light-yellow solid (69.3 mg, 99% yield).

Rf (Pet. Ether /EtOAc = 90/10): 0.45; er = 71:29, $[\alpha]_D^{25}$ = -1.61 (c 1.0, $CHCl_3$). **HPLC** (CHIRALPAK AD, *n*-hexane/IPA = 95:5, flow rate = 1.0 mL/min, λ = 254 nm) t_R = 20.0 min (major), 35.9 min (minor).

^{1}H NMR (400 MHz, $CDCl_3$) δ 9.05 (d, *J* = 3.7 Hz, 1H), 8.28 (d, *J* = 8.0 Hz, 1H), 7.72-7.69 (m, 1H), 7.64 (d, *J* = 8.1 Hz, 1H), 7.45 (t, *J* = 8.0 Hz, 1H), 7.32 (t, *J* = 7.7 Hz, 1H), 7.02 (d, *J* = 7.7 Hz, 1H), 1.31 (s, 9H). **^{13}C NMR (100 MHz, $CDCl_3$)** δ 166.7, 166.6, 155.9, 151.9, 149.3, 131.9, 131.3, 130.3, 129.1, 129.0, 127.9, 127.7, 127.5, 35.7, 31.8. **HRMS (ESI)** calculated $[M+Na]^+$ for $C_{17}H_{16}N_2O_2Na$: 303.1104, found: 303.1106. **FTIR (cm^{-1})** 2964, 2923, 1762, 1723, 1595, 1487, 1442, 1370, 1219, 817.

***(P)*-2-(2-(*tert*-Butyl)phenyl)-4-methylisoindoline-1,3-dione (9a)**

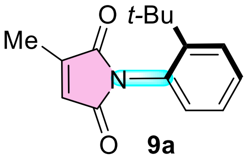

9a

Following the general procedure, K_2CO_3 (52 mg, 0.375 mmol) and (*Z*)-4-((2-(*tert*-butyl)phenyl)amino)-2-methyl-4-oxobut-2-enoic acid **8a** (65.3 mg, 0.25 mmol) were taken in THF (1.0 mL), and then PivCl (46 µL, 0.375 mmol) was added to the reaction mixture, and it was stirred at 25 °C for 30 min (until the full conversion of **8a**, monitored by TLC). Then, the triazolium salt **3** (9.2 mg, 0.025 mmol, 10 mol %) and THF (0.5 mL) were successively added and stirred for 36 h. Then the reaction mixture was purified by flash column chromatography (Pet. Ether-EtOAc: 95:5) to afford *(P)* 2-(2-(*tert*-butyl)phenyl)-4-methylisoindoline-1,3-dione **9a** as a light-yellow solid (40.0 mg, 65% yield).

Rf (Pet. Ether /EtOAc = 90/10): 0.52; er = 93:7, $[\alpha]_D^{25}$ = -4.2 (c 1.0, $CHCl_3$). **HPLC** (CHIRALPAK AD, *n*-hexane/IPA = 99:1, flow rate = 0.7 mL/min, λ = 254 nm) t_R = 12.4 min (minor), 13.9 min (major).

^{1}H NMR (400 MHz, CDCl$_3$) δ 7.59 (dd, J_1 = 8.1 Hz, J_2 = 1.1 Hz, 1H), 7.42-7.37 (m, 1H), 7.29-7.25 (m, 1H), 6.91 (dd, J_1 = 6.3 Hz, J_2 = 1.3 Hz, 1H), 6.52-6.51 (m, 1H), 2.17 (d, J = 1.8 Hz, 3H), 1.30 (s, 9H). **^{13}C NMR (100 MHz, CDCl$_3$)** δ 172.0, 171.0, 149.6, 146.8, 131.5, 129.9, 129.8, 128.6, 128.4, 127.3, 35.5, 31.6, 11.3. **HRMS (ESI)** calculated $[M+Na]^+$ for $C_{15}H_{17}NO_2Na$: 266.1151, found: 266.1151. **FTIR (cm^{-1})** 2962, 2923, 1708, 1642, 1489, 1385, 1285, 1052, 871.

(*P*)-1-(4-Bromo-2-(*tert*-butyl)phenyl)-3-methyl-1*H*-pyrrole-2,5-dione (9b)

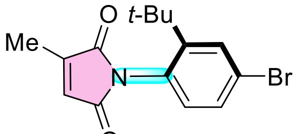

9b

Following the general procedure, K_2CO_3 (52 mg, 0.375 mmol) and (*Z*)-4-((4-bromo-2-(*tert*-butyl)phenyl)amino)-2-methyl-4-oxobut-2-enoic acid **8b** (85.0 mg, 0.25 mmol) were taken in THF (2.0 mL), and then PivCl (46 µL, 0.375 mmol) was added to the reaction mixture, and it was stirred at 25 °C for 30 min (until the full conversion of **8b**, monitored by TLC). Then, the triazolium salt **3** (9.2 mg, 0.025 mmol, 10 mol %) and THF (1.0 mL) were successively added and stirred for 36 h. Then the reaction mixture was purified by flash column chromatography (Pet. Ether- EtOAc: 95:5) to afford (*P)*-1-(4-bromo-2-(*tert*-butyl)phenyl)-3-methyl-1H-pyrrole-2,5-dione **9b** as a light-yellow solid (62.5 mg, 78% yield).

R$_f$ (Pet. Ether /EtOAc = 90/10): 0.25; er = 91:9, $[\alpha]_D^{25}$ = -0.3 (c 1.0, CHCl$_3$). **HPLC** (CHIRALCELL OD-H, *n*-hexane/IPA = 99:1, flow rate = 1.0 mL/min, λ = 254 nm) t_R = 15.2 min (minor), 13.7 min (major).

^{1}H NMR (400 MHz, CDCl$_3$) δ 7.69 (s, 1H), 7.40 (d, J = 8.3 Hz, 1H), 6.77 (d, J = 8.3 Hz, 1H), 6.52 (s, 1H), 2.18 (s, 3H) 1.28 (s, 9H). **^{13}C NMR (100 MHz, CDCl$_3$)** δ 170.7, 168.6, 152.1, 147.0, 133.1, 132.1, 130.6, 129.1, 128.5, 124.2, 35.8, 31.5, 11.4. **HRMS (ESI)** calculated $[M+Na]^+$ for $C_{15}H_{16}BrNO_2Na$: 344.0257, found: 344.0262. **FTIR (cm^{-1})** 2962, 2923, 1760, 1710, 1587, 1483, 1399, 1368, 1096, 767.

(*P*)-1-(2-(*tert*-Butyl)-4-iodophenyl)-3-methyl-1*H*-pyrrole-2,5-dione (9c)

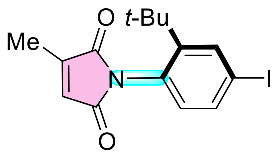

9c

Following the general procedure, K_2CO_3 (52 mg, 0.375 mmol) and (*Z*)-4-((2-(*tert*-butyl)-4-iodophenyl)amino)-2-methyl-4-oxobut-2-enoic acid **8c** (96.8 mg, 0.25 mmol) were taken in THF (1.0 mL), and then PivCl (46 µL, 0.375 mmol) was added to the reaction mixture, and it was stirred at 25 °C for 30 min (until the full conversion of **8c**, monitored by TLC). Then, the triazolium salt **3** (9.2 mg, 0.025 mmol, 10 mol %) and THF (0.5 mL) were successively added

and stirred for 36 h. Then the reaction mixture was purified by flash column chromatography (Pet. Ether- EtOAc: 95:5) to afford (*P*)-1-(2-(*tert*-butyl)-4-iodophenyl)-3-methyl-1*H*-pyrrole-2,5-dione **9c** as a light-yellow solid (80.4 mg, 87% yield).

Rf (Pet. Ether /EtOAc = 90/10): 0.54; er = 94:6, $[\alpha]_D^{25}$ = -2.7 (c 1.0, $CHCl_3$). **HPLC** (CHIRALCELL OD-H, *n*-hexane/IPA = 99:1, flow rate = 1.0 mL/min, λ = 254 nm) t_R = 13.7 min (minor), 14.4 min (major).

^{1}H NMR (400 MHz, $CDCl_3$) δ 7.90-7.90 (m, 1H), 7.62-7.60 (m, 1H), 6.65-6.63 (m, 1H), 6.53 (s, 1H), 2.19-2.19 (m, 3H), 1.29 (s, 9H). **^{13}C NMR (100 MHz, $CDCl_3$)** δ 171.7, 170.7, 152.1, 147.0, 138.1, 136.6, 133.2, 129.9, 128.5, 96.5, 35.6, 31.5, 11.4. **HRMS (ESI)** calculated $[M+Na]^+$ for $C_{15}H_{16}INO_2Na$: 392.0118, found: 392.0121. **FTIR (cm^{-1})** 2961, 2923, 1707, 1481, 1395, 1366, 1176, 1102, 876.

(*P*)-Ethyl-3-(*tert*-butyl)-4-(3-methyl-2,5-dioxo-2,5-dihydro-1*H*-pyrrol-1-yl)benzoate (9d)

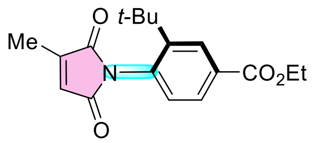

9d

Following the general procedure, K_2CO_3 (52 mg, 0.375 mmol) and (*Z*)-4-((2-(*tert*-butyl)-4-(ethoxycarbonyl)phenyl)amino)-2-methyl-4-oxobut-2-enoic acid **8d** (83.3 mg, 0.25 mmol) were taken in THF (2.0 mL), and then PivCl (46 μL, 0.375 mmol) was added to the reaction mixture, and it was stirred at 25 °C for 30 min (until the full conversion of **8d**, monitored by TLC). Then, the triazolium salt **3** (9.2 mg, 0.025 mmol, 10 mol %) and THF (1.0 mL) were successively added and stirred for 36 h. Then the reaction mixture was purified by flash column chromatography (Pet. Ether- EtOAc: 95:5) to afford (*P)*-Ethyl-3-(*tert*-butyl)-4-(3-methyl-2,5-dioxo-2,5-dihydro-1H-pyrrol-1-yl)benzoate **9d** as a light yellow solid (62.9 mg, 80% yield).

Rf (Pet. Ether /EtOAc = 90/10): 0.20; er = 91:9, $[\alpha]_D^{25}$ = -1.5 (c 1.0, $CHCl_3$). **HPLC** (CHIRALCEL OD-H, *n*-hexane/IPA = 99:1, flow rate = 0.5 mL/min, λ = 254 nm) t_R = 22.2 min (minor), 26.7 min (major).

^{1}H NMR (400 MHz, $CDCl_3$) δ 8.28 (s, 1H), 7.92 (d, *J* = 8.0 Hz, 1H), 6.97 (d, *J* = 8.0 Hz, 1H), 6.53 (s, 1H), 4.39 (q, *J* = 7.0 Hz, 2H), 2.18 (s, 3H), 1.39 (t, *J* = 7.0 Hz, 3H), 1.32 (s, 9H). **^{13}C NMR (100 MHz, $CDCl_3$)** δ 171.6, 170.6, 166.1, 150.2, 147.0, 134.2, 131.8, 131.7, 130.2, 128.5, 128.3, 61.4, 35.8, 31.6, 14.5, 11.4. **HRMS (ESI)** calculated $[M+Na]^+$ for $C_{18}H_{21}NO_4Na$: 338.1363, found: 338.1369. **FTIR (cm^{-1})** 2967, 1711, 1644, 1484, 1377, 1294, 1243, 1177, 1112, 768.

(*P*)-3-Methyl-1-(4-methyl-2-(2-phenylpropan-2-yl)phenyl)-1*H*-pyrrole-2,5-dione (9e)

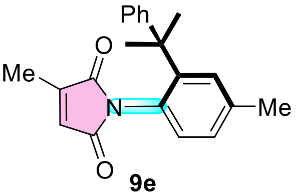

Following the general procedure, K_2CO_3 (52 mg, 0.375 mmol) and (*Z*)-2-methyl-4-((4-methyl-2-(2-phenylpropan-2-yl)phenyl)amino)-4-oxobut-2-enoic acid **8e** (84.3 mg, 0.25 mmol) were taken in THF (1.0 mL), and then PivCl (46 μL, 0.375 mmol) was added to the reaction mixture, and it was stirred at 25 °C for 30 min (until the full conversion of **8e**, monitored by TLC). Then, the triazolium salt **3** (9.2 mg, 0.025 mmol, 10 mol %) and THF (0.5 mL) were successively added and stirred for 36 h. Then the reaction mixture was purified by flash column chromatography (Pet. Ether- EtOAc: 95:5) to afford (*P*)-3-methyl-1-(4-methyl-2-(2-phenylpropan-2-yl)phenyl)-1*H*-pyrrole-2,5-dione **9e** as a light-yellow solid (49.0 mg, 61% yield).

Rf (Pet. Ether /EtOAc = 90/10): 0.52; er = 91:9, $[\alpha]_D^{25}$ = 20.2 (c 1.0, $CHCl_3$). **HPLC** (CHIRALPAK IE, *n*-hexane/IPA = 99:1, flow rate = 1.0 mL/min, λ = 254 nm) t_R = 19.0 min (minor), 20.2 min (major).

^{1}H NMR (400 MHz, $CDCl_3$) δ 7.54 (s, 1H), 7.21-7.17 (m, 2H), 7.13-7.07 (m, 4H), 6.75 (d, *J* = 7.9 Hz, 1H), 6.05 (s, 1H), 2.43 (s, 3H), 1.87 (s, 3H), 1.66 (s, 3H), 1.63 (s, 3H). **^{13}C NMR (100 MHz, $CDCl_3$)** δ 170.5, 170.3, 148.9, 148.7, 145.4, 139.7, 131.4, 129.3, 128.5, 128.3, 127.3, 127.2, 126.5, 125.4, 42.7, 32.0, 31.1, 21.7, 11.0. **HRMS (ESI)** calculated $[M+Na]^+$ for $C_{21}H_{21}NO_2Na$: 342.1465, found: 342.1469. **FTIR (cm^{-1})** 2967, 2923, 1705, 1497, 1381, 1240, 1180, 1030, 856.

(*P*)-3-Bromo-1-(2-(*tert*-butyl)phenyl)-4-methyl-1*H*-pyrrole-2,5-dione (9f)

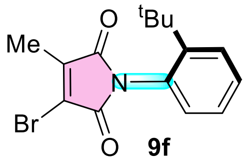

Following the general procedure, K_2CO_3 (52 mg, 0.375 mmol) and (*E*)-3-bromo-4-((2-(*tert*-butyl)phenyl)amino)-2-methyl-4-oxobut-2-enoic acid **8f** (85.1 mg, 0.25 mmol) were taken in THF (1.0 mL), and then PivCl (46 μL, 0.375 mmol) was added to the reaction mixture, and it was stirred at 25 °C for 30 min (until the full conversion of **8f**, monitored by TLC). Then, the triazolium salt **3** (0.0092 g, 0.025 mmol, 10 mol %) and THF (0.5 mL) were successively added and stirred for 24 h. Then the reaction mixture was purified by flash column chromatography (Pet. Ether-EtOAc: 95:5) to afford (*P*)-3-bromo-1-(2-(*tert*-butyl)phenyl)-4-methyl-1*H*-pyrrole-2,5-dione **9f** as a light-yellow solid (55.0 mg, 68% yield).

R_f (Pet. Ether /EtOAc = 90/10): 0.65; er = 85:15, $[\alpha]_D^{25}$ = -1.3 (c 1.0, $CHCl_3$). **HPLC** (CHIRALCELL OJ-H, *n*-hexane/IPA = 99:1, flow rate = 1.0 mL/min, λ = 254 nm) t_R = 12.7 min (minor), 20.2 min (major).

^{1}H NMR (400 MHz, $CDCl_3$) δ 7.59 (d, *J* = 8.4 Hz, 1H), 7.41 (t, *J* = 7.3 Hz, 1H), 7.29-7.26 (m, 1H), 6.91 (d, *J* = 7.8 Hz, 1H), 2.16 (s, 3H), 1.29 (s, 9H). **^{13}C NMR (100 MHz, $CDCl_3$)** 169.7, 165.7, 149.7, 143.4, 131.5, 130.2, 129.6, 128.8, 127.5, 126.1, 35.6, 31.7, 11.1. **HRMS (ESI)** calculated $[M+Na]^+$ for $C_{15}H_{16}BrNO_2Na$: 344.0257, found: 344.0262. **FTIR (cm^{-1})** 2960, 2922, 1791, 1644, 1490, 1379, 1255, 1117, 1033, 764.

2.6 Product Functionalization

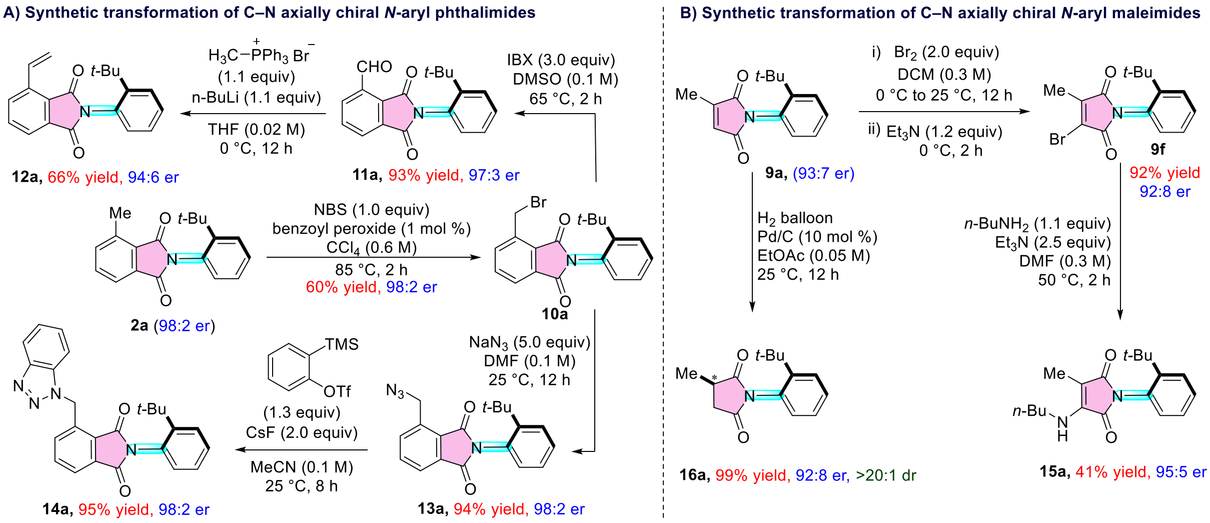

Supplementary Figure 17: Functionalization of C-N axially chiral phthalimides and maleimides

(a) Synthesis of benzyl bromide 10a

(*P*)-4-(Bromomethyl)-2-(2-(*tert*-butyl)phenyl)isoindoline-1,3-dione (10a)

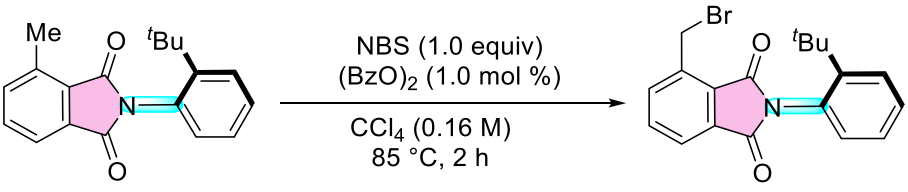

2a **10a**

Compound **10a** was prepared using the modified literature procedure.[9] In an oven-dried screw-capped test tube equipped with a magnetic stir bar, (*P*)-2-(2-(*tert*-butyl)phenyl)-4-methylisoindoline-1,3-dione **2a** (0.088 g, 0.30 mmol), NBS (0.054 g, 0.30 mmol) were

dissolved in dry CCl_4 (1.8 mL). Benzoyl peroxide (0.001 g, 0.003 mmol) was added to the above solution, and the mixture was stirred at 85 °C for 2 h. After completion, the crude residue was purified using flash silica gel column chromatography (Pet. ether /EtOAc = 80/20) to afford (*P*)-4-(bromomethyl)-2-(2-(*tert*-butyl)phenyl)isoindoline-1,3-dione **10a** as pale yellow solid (0.070 g, 60% yield).

Rf (Pet. ether /EtOAc = 90/10): 0.50; er = 98:2, $[\alpha]_D^{25}$ = -0.7 (c 1.0, $CHCl_3$). **HPLC** (CHIRALPAK IA, *n*-hexane/IPA = 95:5, flow rate = 1.0 mL/min, λ = 254 nm) t_R = 8.4 min (minor), 9.4 min (major).

^{1}H NMR (400 MHz, $CDCl_3$) δ 7.90 (d, *J* = 7.2 Hz, 1H), 7.82 (d, *J* = 7.7 Hz, 1H), 7.78-7.74 (m, 1H), 7.65-7.63 (m, 1H), 7.46-7.42 (m, 1H), 7.34-7.30 (m, 1H), 7.02 (dd, J_1 = 6.7Hz, J_2 = 1.1 Hz, 1H), 5.05-4.99 (m, 2H), 1.33 (s, 9H). **^{13}C NMR (100 MHz, $CDCl_3$)** δ 168.4, 168.0, 149.4, 137.5, 136.4, 134.7, 133.0, 131.4, 130.0, 129.6, 128.8, 128.5, 127.4, 123.8, 35.7, 31.7, 26.3. **HRMS (ESI)** calculated $[M+Na]^+$ for $C_{19}H_{18}BrNO_2Na$: 394.0413, found: 394.0419. **FTIR (cm^{-1})** 2922, 2855, 1717, 1595, 1483, 1376, 1219, 1106, 769.

(b) Conversion of Benzyl Bromide to Aldehyde

(*P*)-2-(2-(*tert*-Butyl)phenyl)-1,3-dioxoisoindoline-4-carbaldehyde (11a)

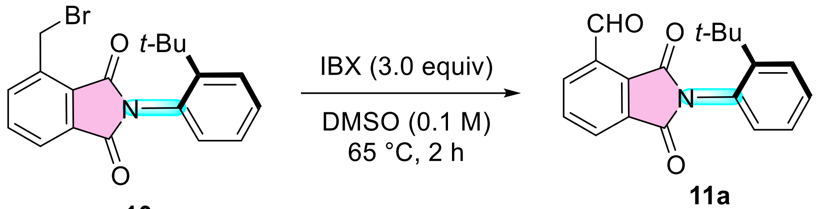

10a **11a**

Compound **11a** was prepared using the modified literature procedure.[10] In an oven-dried screw-capped test tube equipped with a magnetic stir bar, (*P*)-4-(bromomethyl)-2-(2-(*tert*-butyl)phenyl)isoindoline-1,3-dione **10a** (0.037 g, 0.10 mmol) was dissolved in dry DMSO (1.0 mL). IBX (0.085 g, 0.3 mmol) was added to the above solution, and the mixture was stirred at 65 °C for 2 h. After completion, the reaction mixture was quenched with H_2O (5 mL), and then extractedwith EtOAc (3x10 mL). The organic layer collected, and washed with brine (2x5 mL) and dried over Na_2SO_4. The crude residue was purified using flash silica gel column chromatography (Pet. ether /EtOAc = 80/20) to afford (*P*)-2-(2-(*tert*-butyl)phenyl)-1,3-dioxoisoindoline-4-carbaldehyde **11a** as pale yellow solid (0.028 g, 93% yield).

R$_f$ (Pet. ether /EtOAc = 90/10): 0.32; er = 97:3, $[\alpha]_D^{25}$ = -0.7 (c 1.0, $CHCl_3$). **HPLC** (CHIRALPAK IA, *n*-hexane/IPA = 95:5, flow rate = 1.0 mL/min, λ = 254 nm) t_R = 10.5 min (minor), 11.1 min (major).

^{1}H NMR (400 MHz, $CDCl_3$) δ 11.06 (s, 1H), 8.34 (d, *J* = 7.9 Hz, 1H), 8.20 (d, *J* = 7.4 Hz, 1H), 7.93 (d, *J* = 7.7 Hz, 1H), 7.66 (d, *J* = 8.2 Hz, 1H), 7.47 (t, *J* = 7.7 Hz, 1H), 7.33 (t, *J* = 7.7 Hz, 1H), 7.02 (d, *J* = 7.7 Hz, 1H), 1.33 (s, 9H). **^{13}C NMR (100 MHz, $CDCl_3$)** δ 188.8, 168.1, 167.7, 149.4, 134.8, 134.0, 133.2, 132.2, 132.0, 131.3, 130.3, 129.3, 129.0, 128.6, 127.6, 35.8, 31.8. **HRMS (ESI)** calculated $[M+H]^+$ for $C_{19}H_{18}NO_3$: 308.1281, found: 308.1284. **FTIR (cm^{-1})** 2961, 2923, 1714, 1698, 1611, 1490, 1370, 1251, 1158.

(c) Synthesis of Styrene Derivative 12a

(*P*)-2-(2-(*tert*-Butyl)phenyl)-4-vinylisoindoline-1,3-dione (12a)

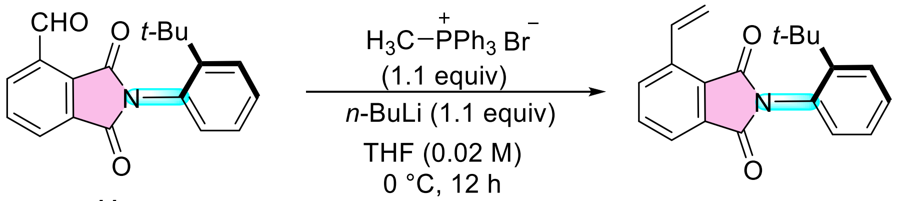

11a **12a**

Compound **12a** was prepared using the modified literature procedure.[10] An oven-dried screw-capped test tube equipped with a magnetic stir bar, was charged with methyl triphenylphosphonium bromide (39.3 mg, 0.11 mmol), anhydrous THF (5.0 mL) and *n*-BuLi (2.5 mol/L in hexane; 45 μL, 0.11 mmol) was added. The mixture was stirred at 0 °C for 30 minutes, followed by THF solution (0.5 mL) of (*P*)-2-(2-(*tert*-butyl)phenyl)-1,3-dioxoisoindoline-4-carbaldehyde **11a** (0.031g, 0.1 mmol) was added dropwise and stirring at 0 °C for 12 h, until the reaction was complete as indicated by TLC. The reaction mixture was then quenched with H_2O (5 mL), extracted with CH_2Cl_2 (3×5 mL). Drying (Na_2SO_4) and evaporation of the solvent gave a residue that was purified by silica gel column chromatography (Pet. ether /EtOAc = 80/20) to afford (*P*)-2-(2-(*tert*-butyl)phenyl)-4-vinylisoindoline-1,3-dione **12a** as pale yellow solid (0.021 g, 66% yield).

R$_f$ (Pet. ether /EtOAc = 90/10): 0.42; er = 94:6, $[\alpha]_D^{25}$ = 1.7 (c 1.0, $CHCl_3$). **HPLC** (CHIRALPAK IA, *n*-hexane/IPA = 98:2, flow rate = 0.7 mL/min, λ = 254 nm) t_R = 11.5 min (minor), 12.2 min (major).

^{1}H NMR (400 MHz, $CDCl_3$) δ 7.98 (d, *J* = 8.0 Hz, 1H), 7.87-7.80 (m, 2H), 7.73 (d, *J* = 7.7 Hz, 1H), 7.64-7.62 (m, 1H), 7.45-7.41 (m, 1H), 7.33-7.29 (m, 1H), 7.00 (d, J_1 = 7.7 Hz, J_2 =

1.1 Hz, 1H), 6.05 (d, J = 17.8 Hz, 1H), 5.61 (d, J = 11.1 Hz, 1H), 1.32 (s, 9H). **^{13}C NMR (100 MHz, $CDCl_3$)** δ 169.2, 168.6, 149.5, 137.0, 134.1, 133.0, 131.5, 130.9, 130.5, 130.0, 129.9, 128.8, 127.4, 127.2, 123.0, 119.6, 35.7, 31.8. **HRMS (ESI)** calculated $[M+Na]^+$ for $C_{20}H_{19}NO_2Na$: 328.1308, found: 328.1312. **FTIR (cm^{-1})** 3020, 2964, 1712, 1488, 1375, 1212, 1100, 925, 747.

(d) Conversion of Bromide to Azide

(*P*)-4-(Azidomethyl)-2-(2-(*tert*-butyl)phenyl)isoindoline-1,3-dione (13a)

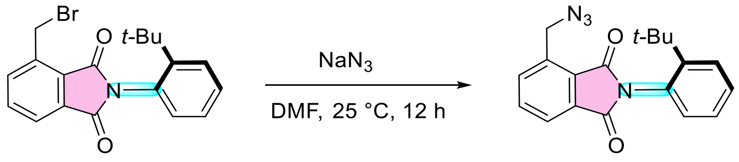

10a **13a**

Compound **13a** was prepared using the modified literature procedure.[11] In an oven-dried screw-capped test tube equipped with a magnetic stir bar, (*P*)-4-(bromomethyl)-2-(2-(*tert*-butyl)phenyl) isoindoline-1,3-dione **10a** (0.037 g, 0.10 mmol) was dissolved in dry DMF (1.0 mL). NaN_3 (0.033 g, 0.50 mmol) was added to the above solution, and the mixture was stirred at 25 °C for 12 h. After completion, the reaction mixture was diluted with EtOAc (10 mL), washed with H_2O (5 mL × 3), brine (5 mL × 2) and the organic layer was separated and dried over Na_2SO_4. The crude residue was purified using flash silica gel column chromatography (Pet. ether /EtOAc = 80/20) to afford (*P*)-4-(azidomethyl)-2-(2-(*tert*-butyl)phenyl)isoindoline-1,3-dione **13a** as pale yellow solid (0.032 g, 94% yield).

R_f (Pet. ether /EtOAc = 90/10): 0.42; er = 98:2, **HPLC** (CHIRALPAK IA, *n*-hexane/IPA = 95:5, flow rate = 1.0 mL/min, λ = 254 nm) t_R = 8.4 min (minor), 9.8 min (major).

^{1}H NMR (400 MHz, $CDCl_3$) δ 7.93-7.82 (m, 3H), 7.64 (d, J = 8.0 Hz, 1H), 7.44 (t, J = 7.6 Hz, 1H), 7.31 (t, J = 7.4 Hz, 1H), 7.00 (d, J = 8.0 Hz, 1H), 5.04 (d, J = 15.0 Hz, 1H), 4.94 (d, J = 14.9 Hz, 1H), 1.32 (s, 9H). **^{13}C NMR (100 MHz, $CDCl_3$)** δ 168.8, 168.3, 149.4, 135.5, 134.8, 134.4, 133.0, 131.4, 130.0, 129.6, 128.9, 128.8, 127.4, 123.6, 49.6, 35.7, 31.8. **HRMS (ESI)** calculated $[M+Na]^+$ for $C_{19}H_{18}N_4O_2Na$: 357.1322, found: 357.1325. **FTIR (cm^{-1})** 2961, 2894, 1715, 1595, 1505, 1363, 1250, 1066, 754.

(e) Synthesis of Benzotriazole 14a

(*P*)-4-((1*H*-Benzo[*d*][1,2,3]triazol-1-yl)methyl)-2-(2-(*tert*-butyl)phenyl)isoindoline-1,3-dione (14a)

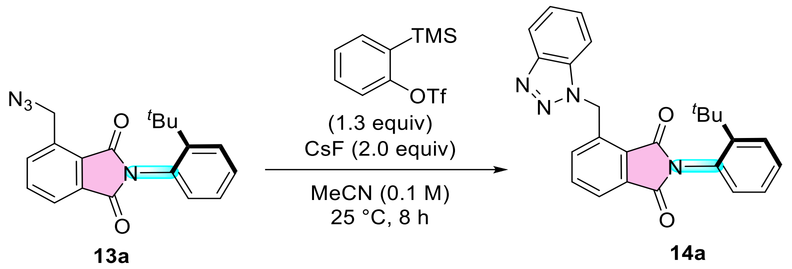

Compound **14a** was prepared using the modified literature procedure.[12] In an oven-dried screw-capped test tube equipped with a magnetic stir bar was added CsF (0.030 g, 0.20 mmol) inside the glove box. To this was added the solution of (*P*)-4-(azidomethyl)-2-(2-(*tert*-butyl)phenyl)isoindoline-1,3-dione **13a** (0.033 g, 0.10 mmol) and 2-(trimethylsilyl)phenyl trifluoromethanesulfonate (0.039g, 0.13 mmol) in dry MeCN (1.0 mL). Then, the reaction mixture was stirred at room temperature for 8 h followed by flash column chromatography (Pet.ether/ EtOAc = 85/15) of the crude reaction mixture using silica gel affording (*P*)-4-((1*H*-benzo[d][1,2,3]triazol-1-yl)methyl)-2-(2-(*tert*-butyl)phenyl)isoindoline-1,3-dione **14a** as pale yellow solid (0.039 g, 95% yield).

R_f (Pet. ether /EtOAc = 85/15): 0.25; er = 98:2, $[\alpha]_D^{25}$ = -22.5 (c 1.0, $CHCl_3$). **HPLC** (CHIRALPAK IA, *n*-hexane/IPA = 90:10, flow rate = 1.0 mL/min, λ = 254 nm) t_R = 13.1 min (minor), 15.7 min (major).

^{1}H NMR (400 MHz, $CDCl_3$) δ 8.08 (d, *J* = 8.1 Hz, 1H), 7.91 (d, *J* = 7.2 Hz, 1H), 7.71-7.62 (m, 3H), 7.58 (d, *J* = 7.8 Hz, 1H), 7.49-7.42 (m, 2H), 7.39-7.33 (m, 2H), 7.05 (d, *J* = 7.7 Hz, 1H), 6.51 (d, *J* = 16.0 Hz, 1H), 6.39 (d, *J* = 16.2 Hz, 1H), 1.34 (s, 9H). **^{13}C NMR (100 MHz, $CDCl_3$)** δ 169.4, 168.0, 149.4, 146.1, 135.1, 134.9, 134.6, 133.1, 132.8, 131.4, 130.2, 129.5, 129.0, 128.5, 128.0, 127.6, 124.3, 124.1, 120.2, 109.9, 45.9, 35.7, 31.8. **HRMS (ESI)** calculated $[M+H]^+$ for $C_{25}H_{23}N_4O_2$: 411.1816, found: 411.1823. **FTIR (cm^{-1})** 2961, 2924, 1711, 1608, 1487, 1374, 1221, 1160, 1051, 805.

(f) Bromination of N-Aryl Maleimide 9a

(*P*)-3-Bromo-1-(2-(*tert*-butyl)phenyl)-4-methyl-1*H*-pyrrole-2,5-dione (9f)

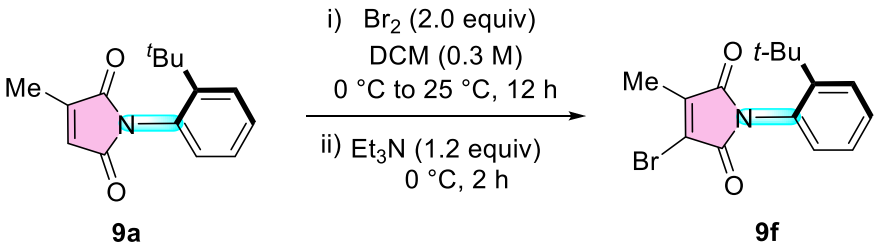

Compound **9f** was prepared using the modified literature procedure.[13] In an oven-dried screw-capped test tube equipped with a magnetic stir bar, (*P*)-1-(2-(*tert*-butyl)phenyl)-3-methyl-1*H*-pyrrole-2,5-dione **9a** (0.049 g, 0.20 mmol) was dissolved in dry DCM (0.6 mL) and cooled to 0 °C. Br_2 (21.0 µL, 0.4 mmol) was added dropwise to the above solution, and the mixture was allowed to warm to room temperature and stirred for 12 h. After completion, the reaction mixture was again cooled to 0 °C, and Et_3N (34 µL, 0.24 mmol) was added dropwise and stirred at the same temperature for 2 h. After completion of the reaction, the mixture was diluted with H_2O (5 mL), and extracted with EtOAc (3x 10 mL), washed with brine (2x 5 mL), and the organic layer dried over Na_2SO_4. The crude residue was purified using flash silica gel column chromatography (Pet. ether /EtOAc = 80/20) to afford (*P*)-3-bromo-1-(2-(*tert*-butyl)phenyl)-4-methyl-1*H*-pyrrole-2,5-dione **9f** as pale yellow solid (0.060 g, 92% yield). ***R_f*** (Pet. ether /EtOAc = 90/10): 0.65; er = 90:10, $[\alpha]_D^{25}$ = -1.7 (c 1.0, $CHCl_3$). **HPLC** (CHIRALCELL OJ-H, *n*-hexane/IPA = 99:1, flow rate = 1.0 mL/min, λ = 254 nm) t_R = 12.1 min (minor), 20.0 min (major).

(g) Synthesis of N-Aryl Aminomaleimide 15a

(*P*)-1-(2-(*tert*-Butyl)phenyl)-3-(butylamino)-4-methyl-1*H*-pyrrole-2,5-dione (15a)

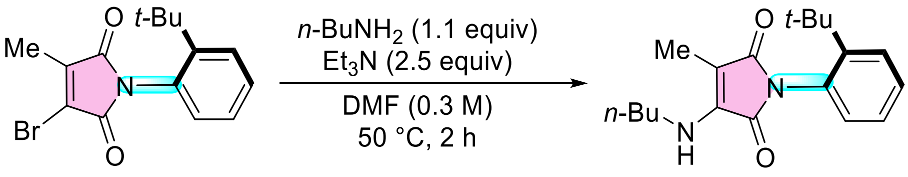

9f **15a**

Compound **15a** was prepared using the modified literature procedure.[13] In an oven-dried screw-capped test tube equipped with a magnetic stir bar afford (*P*)-3-bromo-1-(2-(*tert*-butyl)phenyl)-4-methyl-1*H*-pyrrole-2,5-dione **9f** (0.032 g, 0.10 mmol) was dissolved in dry DMF (0.3 mL) and the mixture was stirred at 25 °C for 5 min. Then *n*-$BuNH_2$ (0.081 g, 1.1 equiv) was added to the reaction mixture and stirred at 50 °C for 2 h. After completion of the reaction, the mixture was diluted with EtOAc (10 mL), washed with H_2O (5 mL × 3), brine (5 mL × 2), and the organic layer was separated and dried over Na_2SO_4. The crude residue was purified using flash silica gel column chromatography (Pet. ether /EtOAc = 80/20) to afford (*P*)-1-(2-(*tert*-butyl)phenyl)-3-(butylamino)-4-methyl-1*H*-pyrrole-2,5-dione **15a** as pale yellow solid (0.013 g, 41% yield).

R_f (Pet. ether /EtOAc = 90/10): 0.34; er = 94:6, $[\alpha]_D^{25}$ = 13.5 (c 1.0, $CHCl_3$). **HPLC** (CHIRALCELL OJ-H, *n*-hexane/IPA = 98:2, flow rate = 1.0 mL/min, λ = 254 nm) t_R = 22.7 min (major), 27.3 min (minor).

^{1}H NMR (400 MHz, $CDCl_3$) δ 7.59 (d, *J* = 8.1 Hz, 1H), 7.39 (t, *J* = 7.8 Hz, 1H), 7.29 (d, *J* = 7.1 Hz, 1H), 6.97 (d, *J* = 7.6 Hz, 1H), 5.17 (bs, 1H), 3.51 (q, *J* = 6.7 Hz, 2H), 2.08 (s, 3H), 1.71-1.64 (m, 2H), 1.52-1.43 (m, 2H), 1.35 (s, 9H), 1.01 (t, *J* = 7.2 Hz, 3H). **^{13}C NMR (100 MHz, $CDCl_3$)** δ 174.4, 168.8, 149.9, 143.2, 131.6, 130.7, 129.5, 128.4, 127.2, 94.8, 43.6, 35.5, 32.8, 31.6, 20.0, 13.9, 7.5. **HRMS (ESI)** calculated $[M+Na]^+$ for $C_{19}H_{26}N_2O_2Na$: 337.1886, found: 337.1890. **FTIR (cm^{-1})** 2956, 2856, 1707, 1654, 1524, 1491, 1448, 1282, 1185, 1100.

(h) Hydrogenation of 9a

(*P*, *R*)-1-(2-(*tert*-Butyl)phenyl)-3-methylpyrrolidine-2,5-dione (16a)

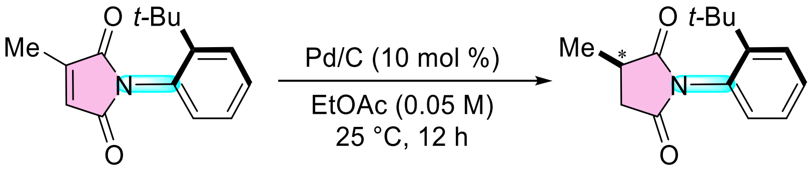

9a **16a**

Compound **16a** was prepared by using the modified literature procedure.[14] In an oven dried screw-capped test tube equipped with a magnetic stir bar, (*P*)-1-(2-(*tert*-butyl)phenyl)-3-methyl-1*H*-pyrrole-2,5-dione **9a** (0.048 g, 0.2 mmol) and Pd-C catalyst (0.003 g, 0.02 mmol) was dissolved in EtOAc (4.0 mL, 0.05 M). The reaction mixture was stirred vigorously under H_2 at 1 atm for 12 h followed by flash column chromatography (Pet.ether/ EtOAc = 70/30) of the crude reaction mixture using silica gel afforded (*P*, *R*)-1-(2-(*tert*-butyl)phenyl)-3-methylpyrrolidine-2,5-dione **16a** as yellow oil (0.048 g, 99% yield).

R_f (Pet. ether /EtOAc = 90/10): 0.25; er = 92:8, dr = >20:1, $[\alpha]_D^{25}$ = 9.9 (c 1.0, $CHCl_3$). **HPLC** (CHIRALCELL OJ-H, *n*-hexane/IPA = 80:20, flow rate = 1.0 mL/min, λ = 254 nm) t_R = 8.6 min (minor), 9.9 min (major).

^{1}H NMR (400 MHz, $CDCl_3$) δ 7.57 (d, *J* = 8.2 Hz, 1H), 7.38 (t, *J* = 7.7 Hz, 1H), 7.30-7.25 (m, 1H), 6.84 (d, *J* = 7.7 Hz, 1H), 3.11-2.97 (m, 2H), 2.49 (dd, J_1 = 17.5 Hz, J_2 = 4.1 Hz, 1H), 1.45 (d, *J* = 7.2 Hz, 3H), 1.30 (s, 9H). **^{13}C NMR (100 MHz, $CDCl_3$)** δ 180.5, 176.9, 148.1, 130.8, 130.7, 129.9, 128.9, 127.6, 37.2, 35.8, 35.4, 31.8, 16.8. **HRMS (ESI)** calculated $[M+Na]^+$ for $C_{15}H_{19}NO_2Na$: 268.1308, found: 268.1314. **FTIR (cm^{-1})** 2952, 2850, 1701, 1658, 1500, 1470, 1455, 1202, 1080, 754.

3. Supplementary Figures

3.1 ^{1}H and ^{13}C NMR Spectra of Phthalamic/Maleamic Acid Derivatives

2-((2-(*tert*-Butyl)phenyl)carbamoyl)-6-methylbenzoic acid (1a)

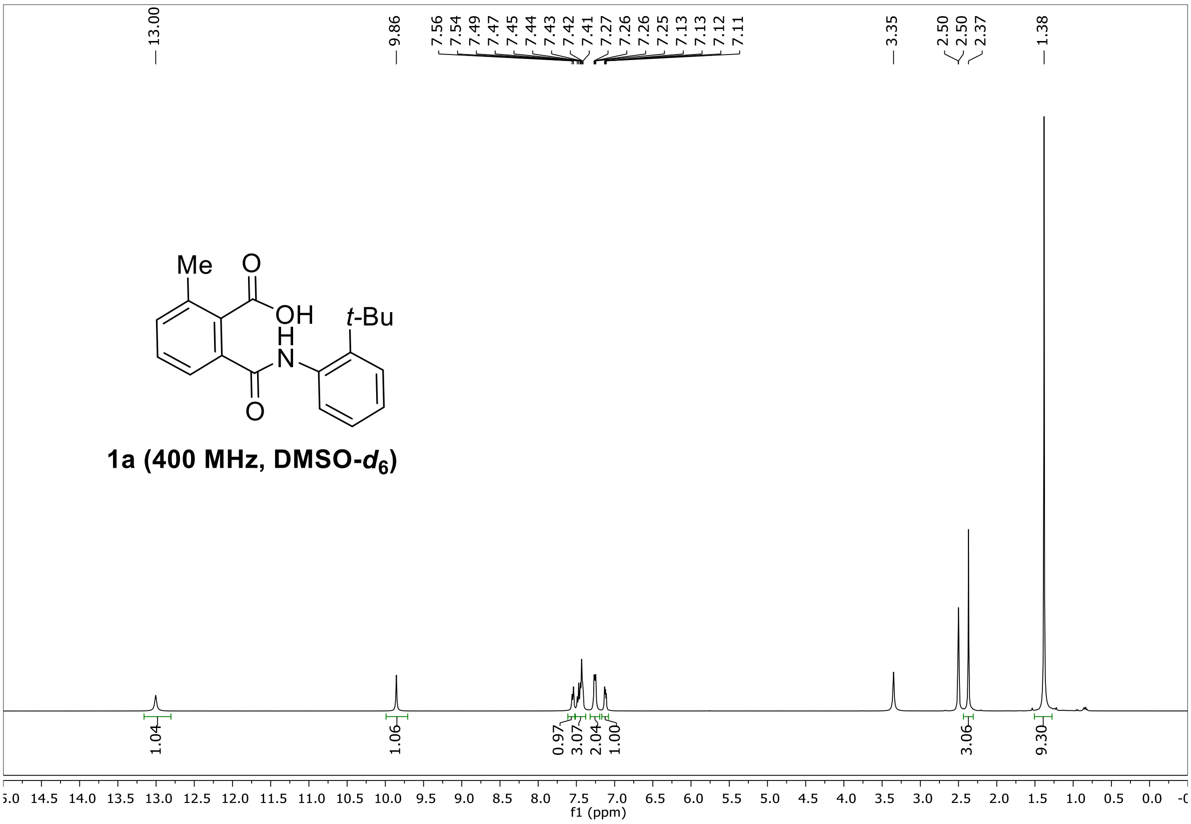

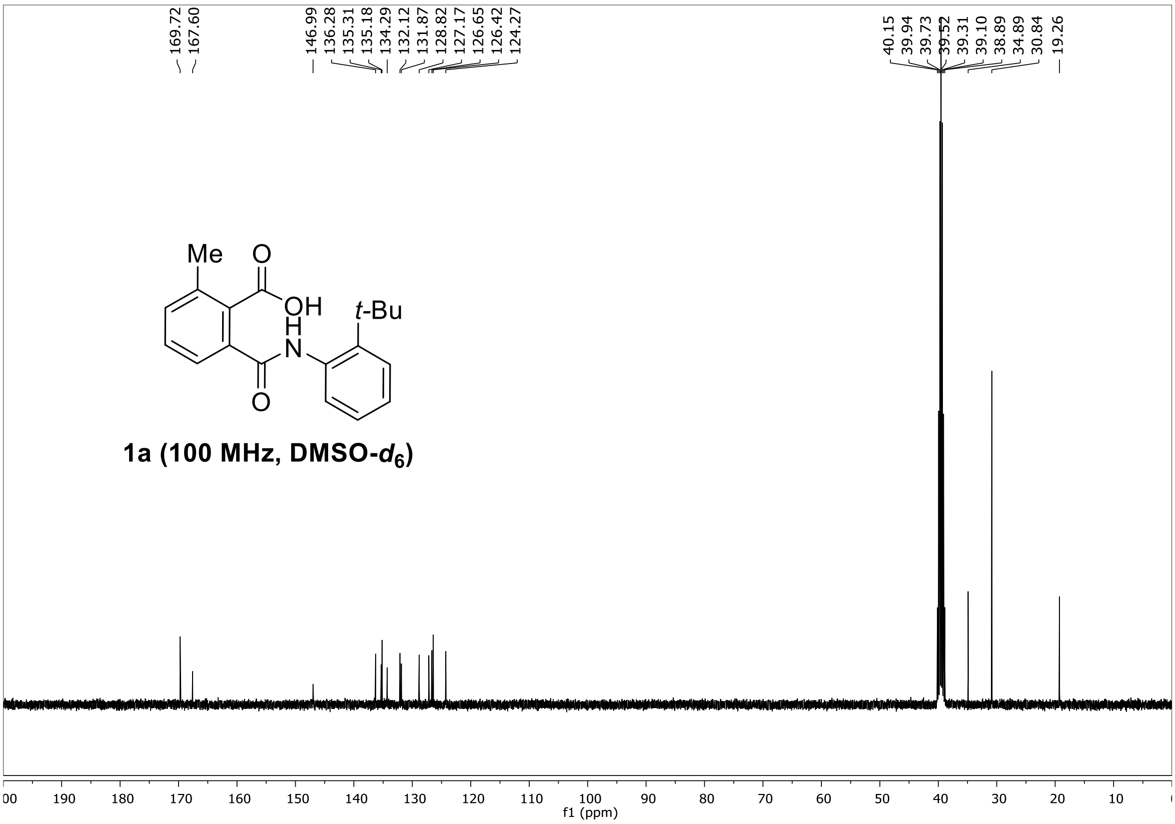

2-((2-(*tert*-Butyl)phenyl)carbamoyl)-3-methylbenzoic acid (1a′)

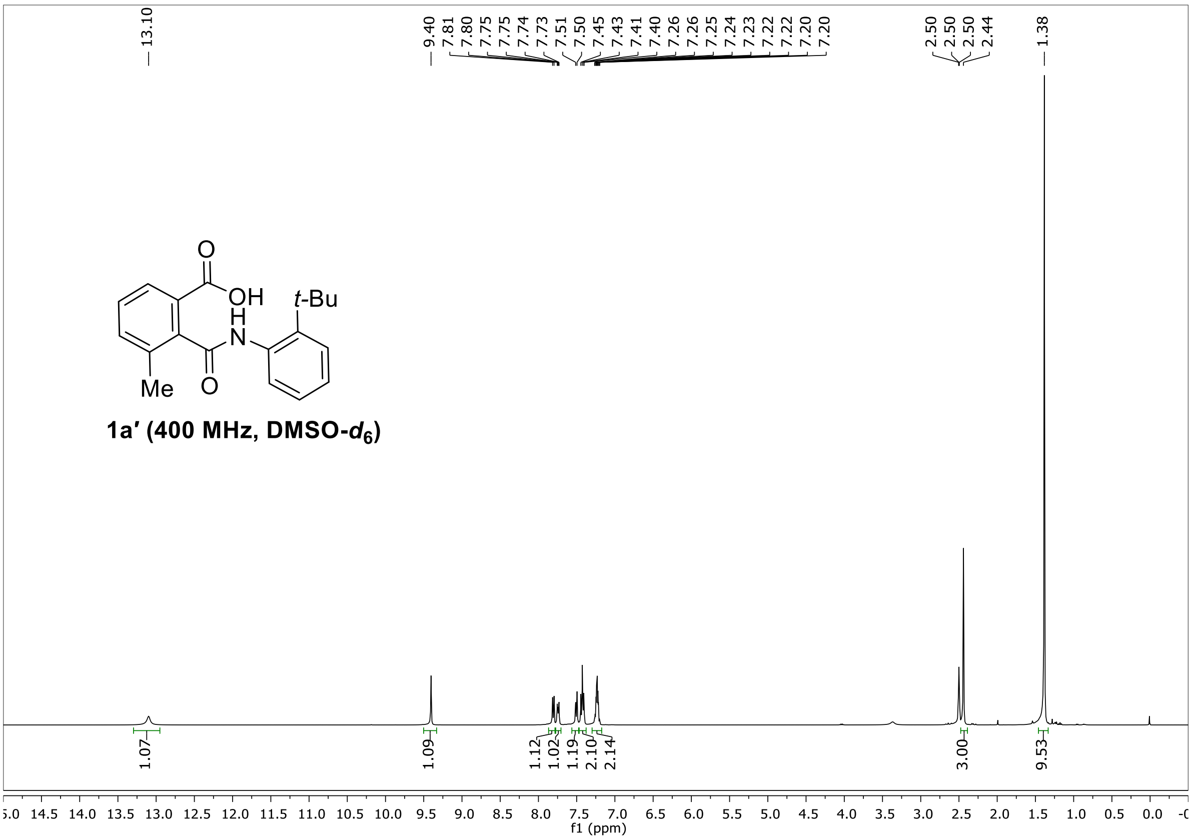

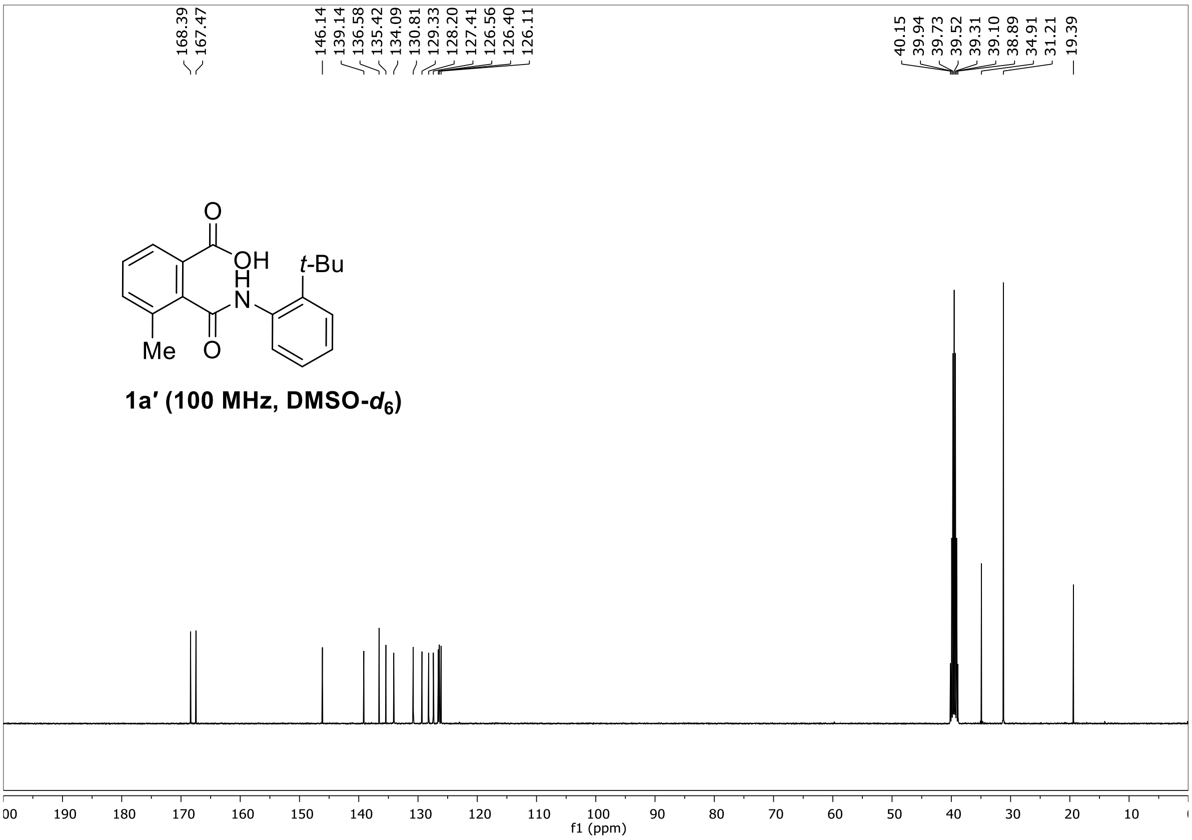

2-((2-(*tert*-Butyl)-4-methoxyphenyl)carbamoyl)-6-methylbenzoic acid (1b)

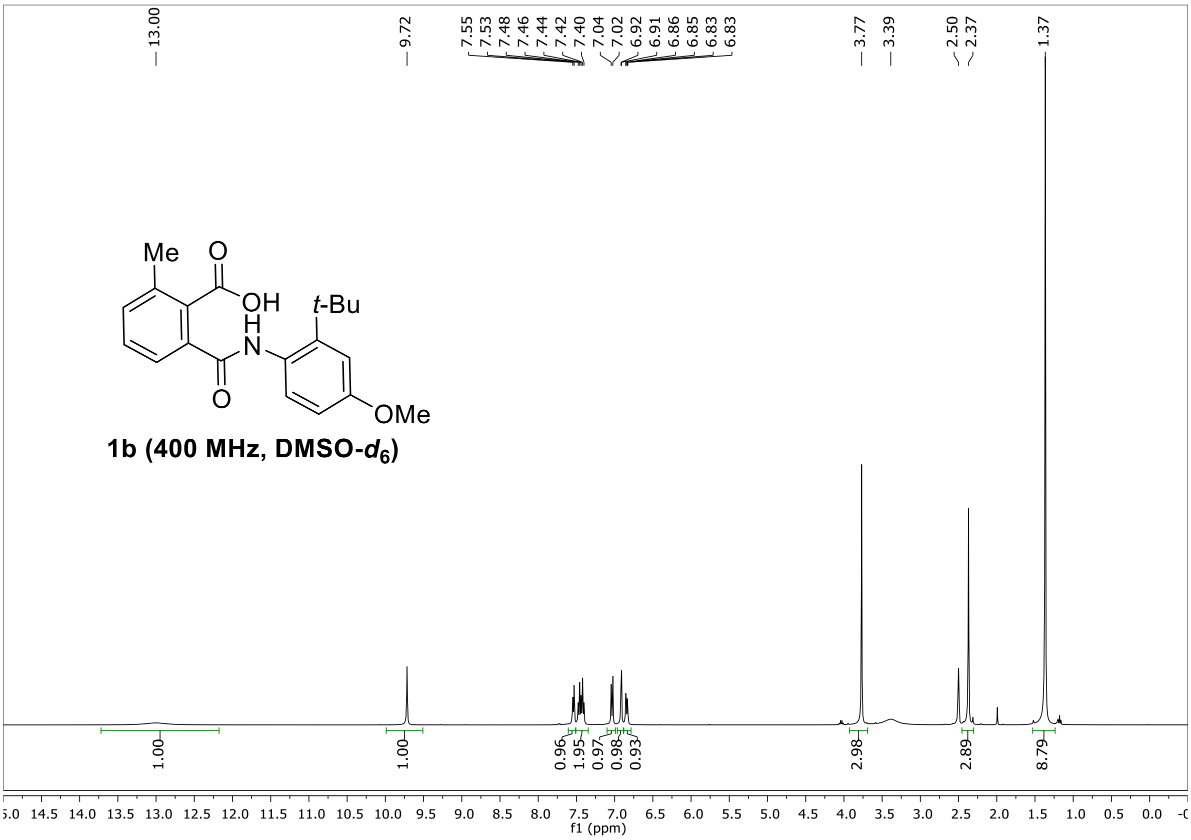

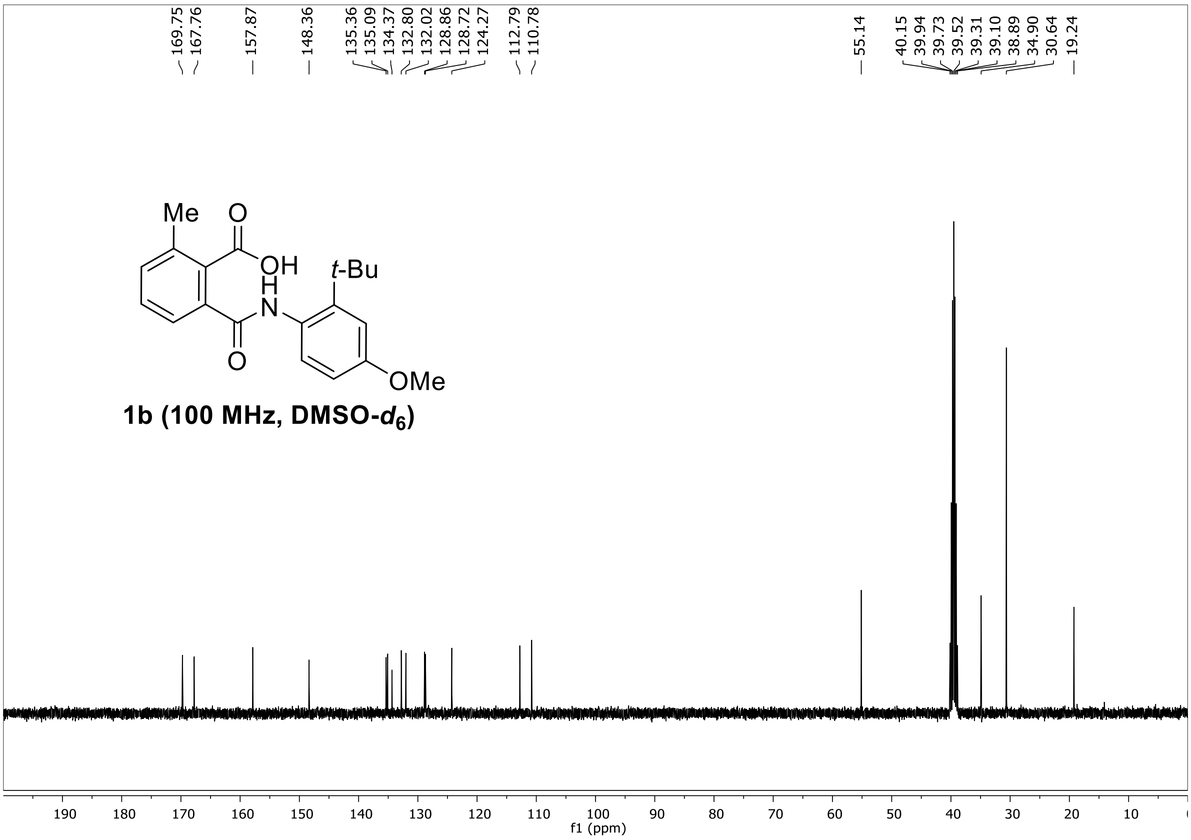

2-((4-(Benzyloxy)-2-(*tert*-butyl)phenyl)carbamoyl)-6-methylbenzoic acid (1c)

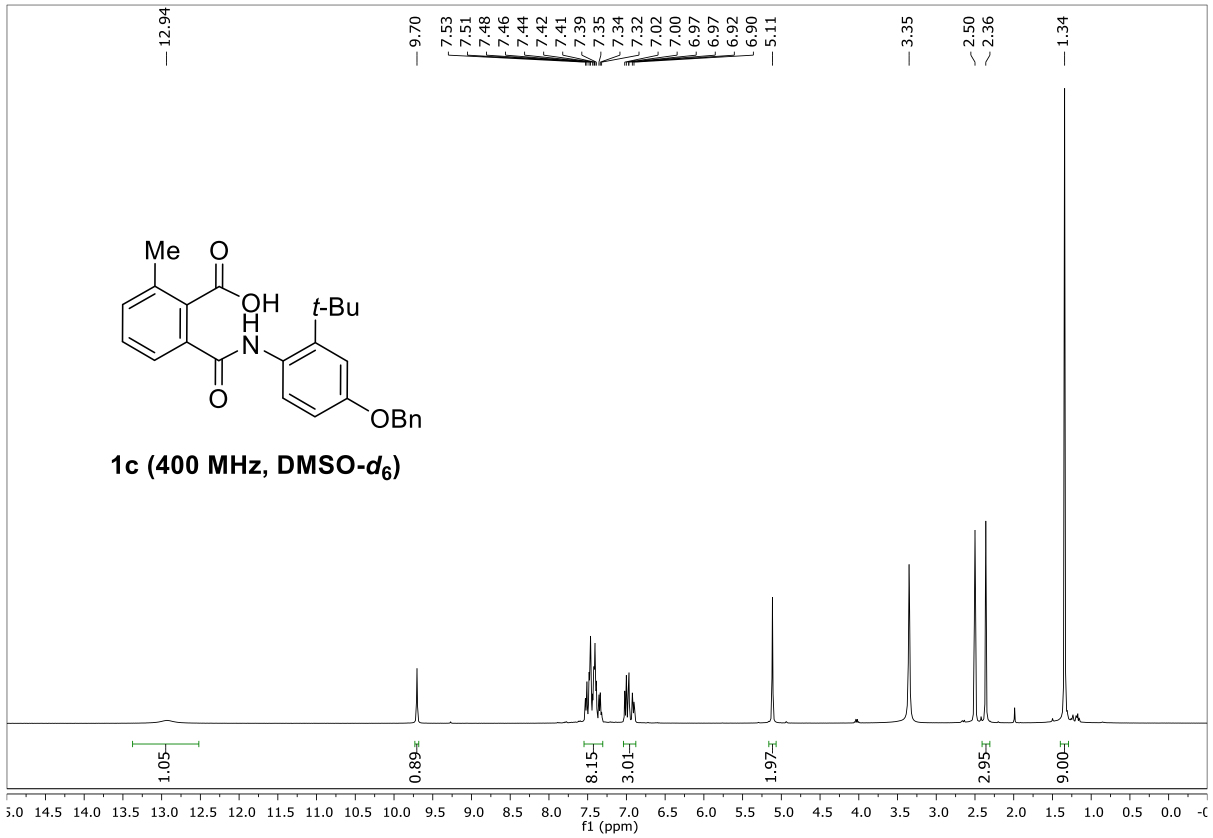

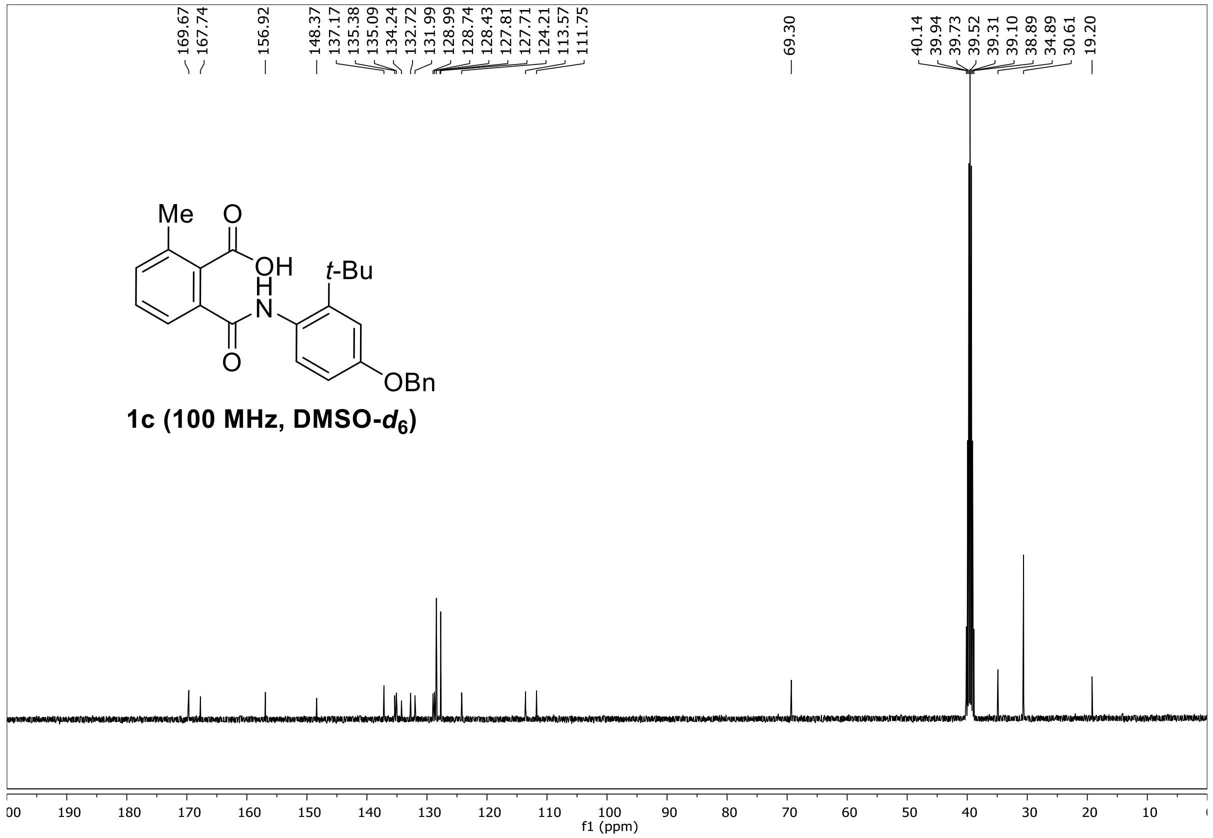

2-((4-Bromo-2-(*tert*-butyl)phenyl)carbamoyl)-6-methylbenzoic acid (1d)

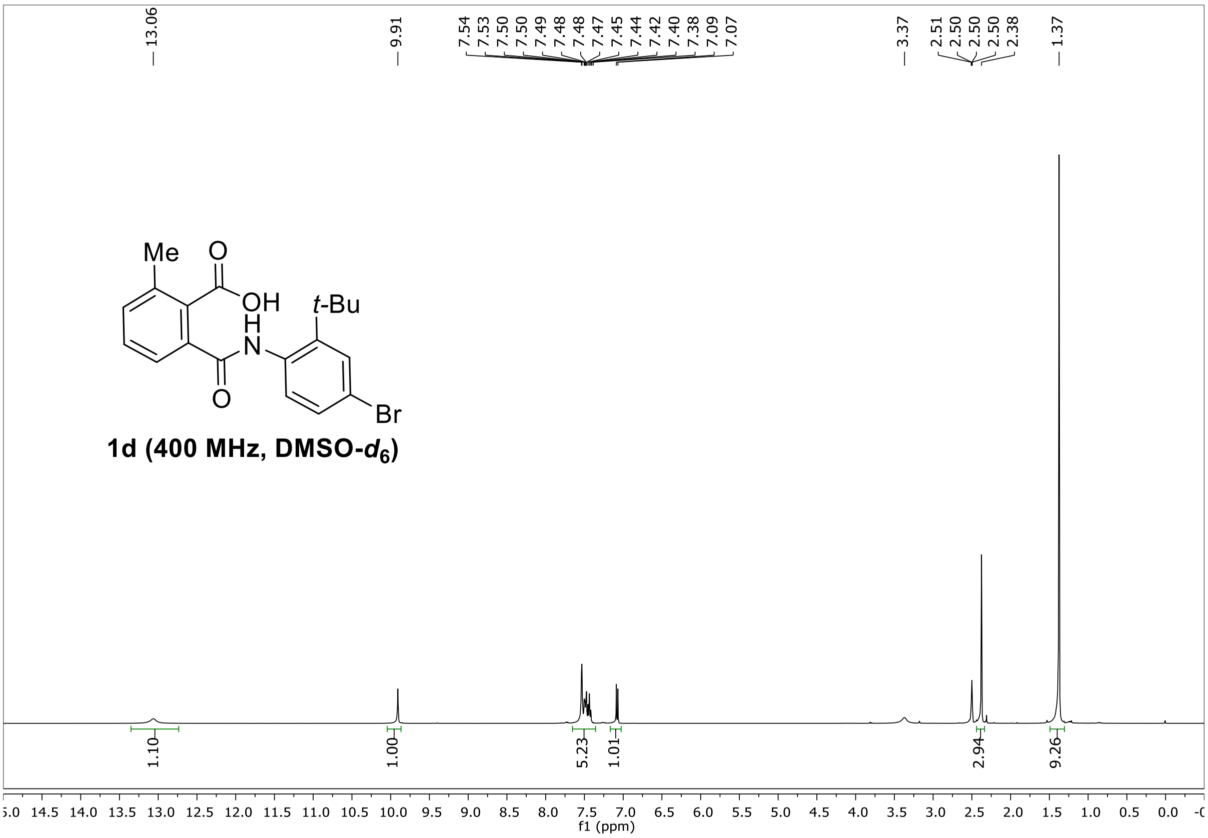

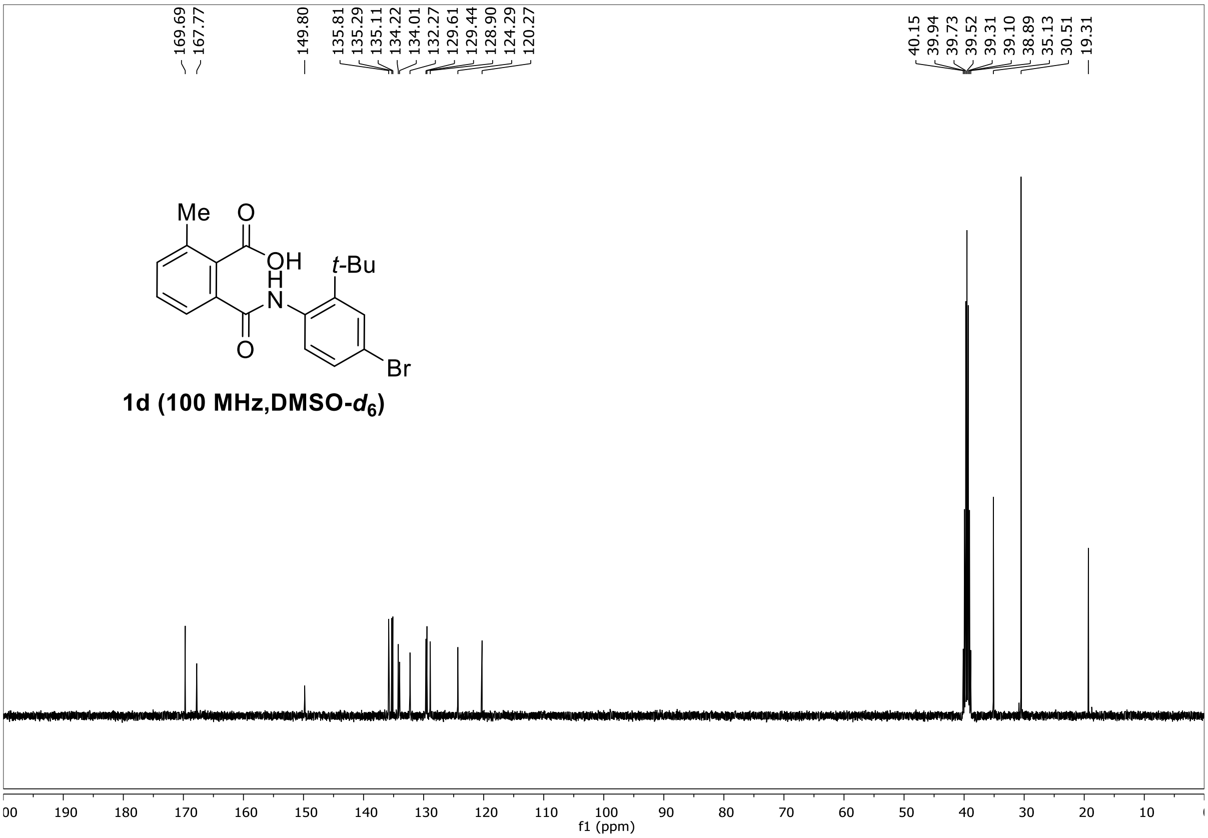

2-((4-Bromo-2-(*tert*-butyl)phenyl)carbamoyl)-6-methylbenzoic acid (1e)

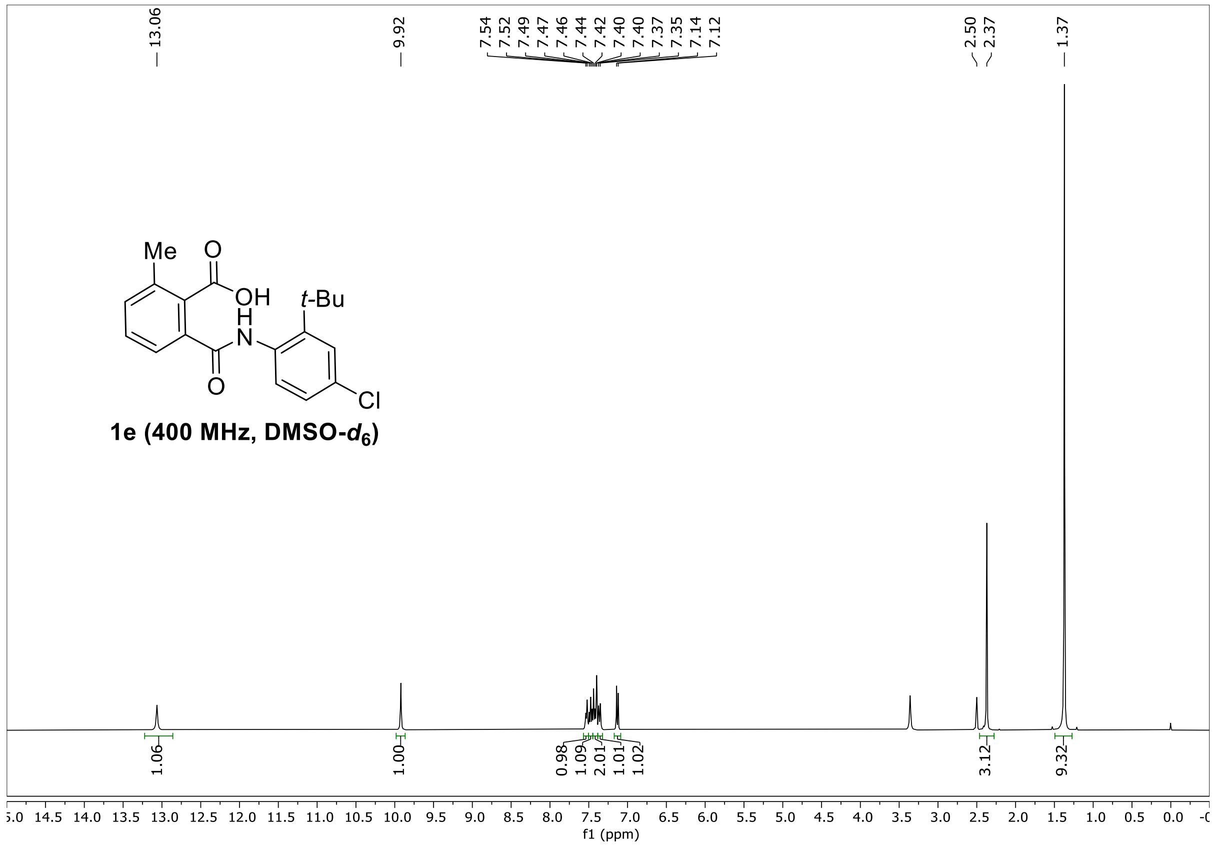

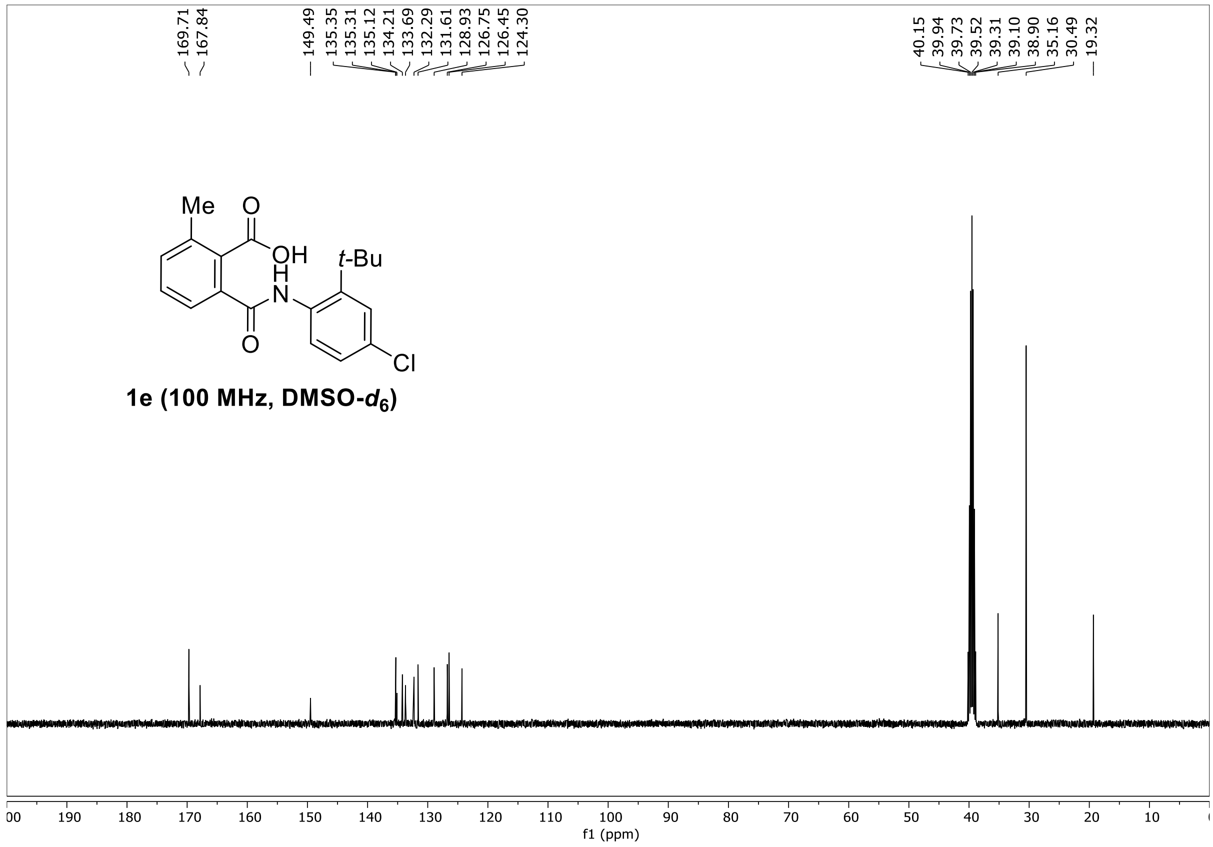

2-((2-(*tert*-Butyl)-4-iodophenyl)carbamoyl)-6-methylbenzoic acid (1f)

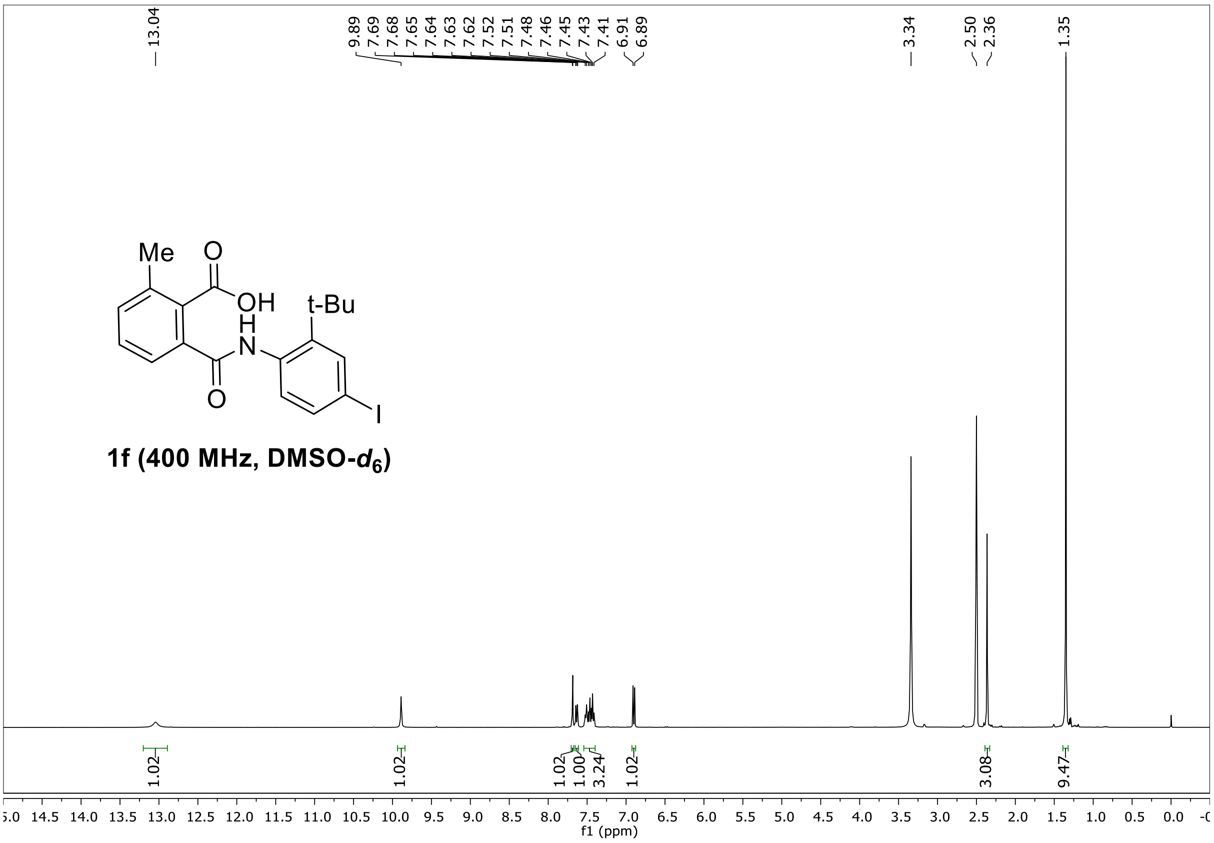

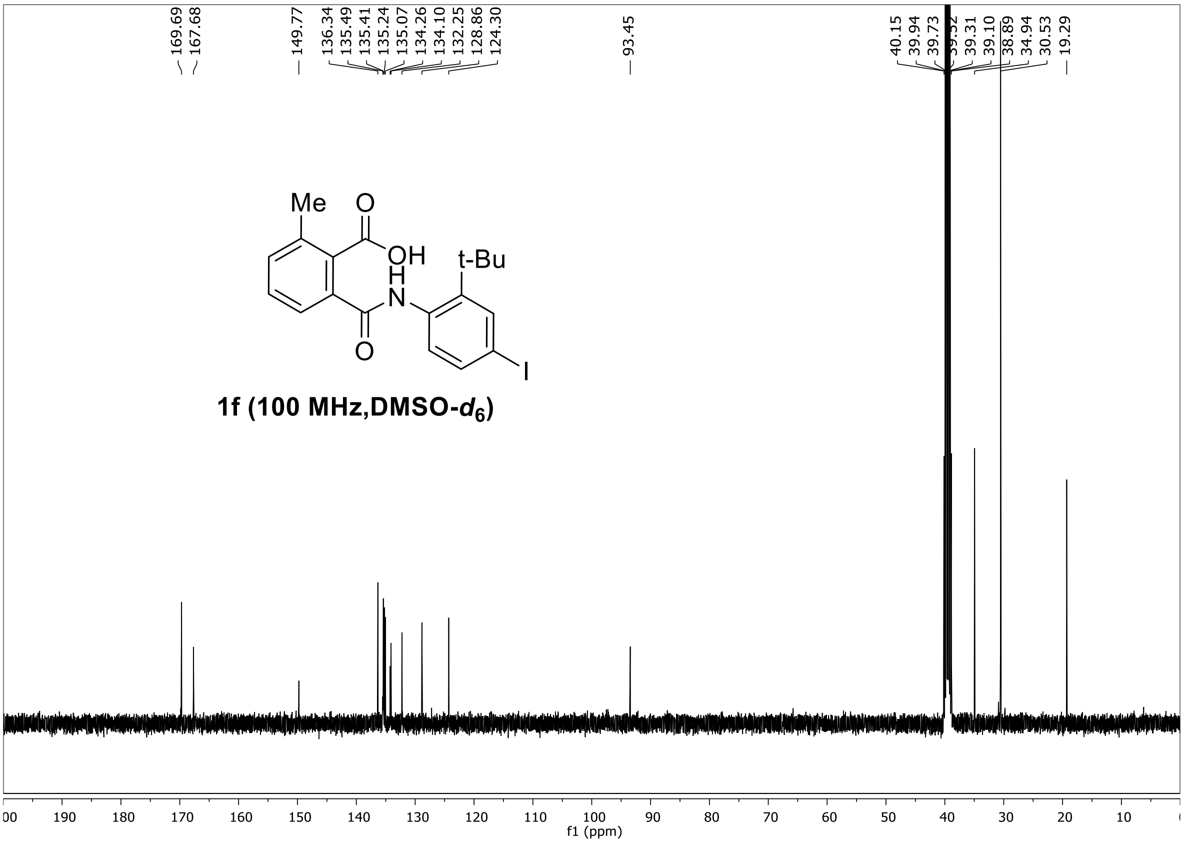

2-((3-(*tert*-Butyl)-[1,1'-biphenyl]-4-yl)carbamoyl)-6-methylbenzoic acid (1g)

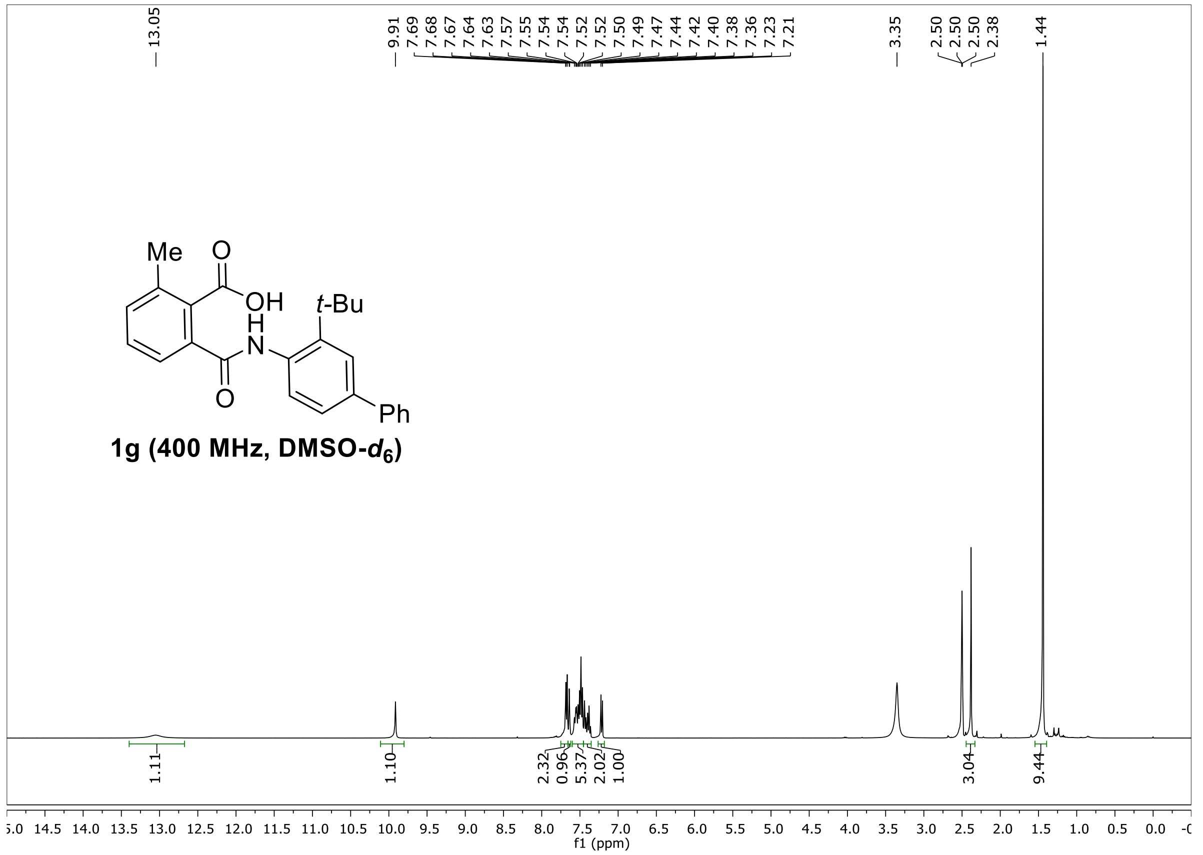

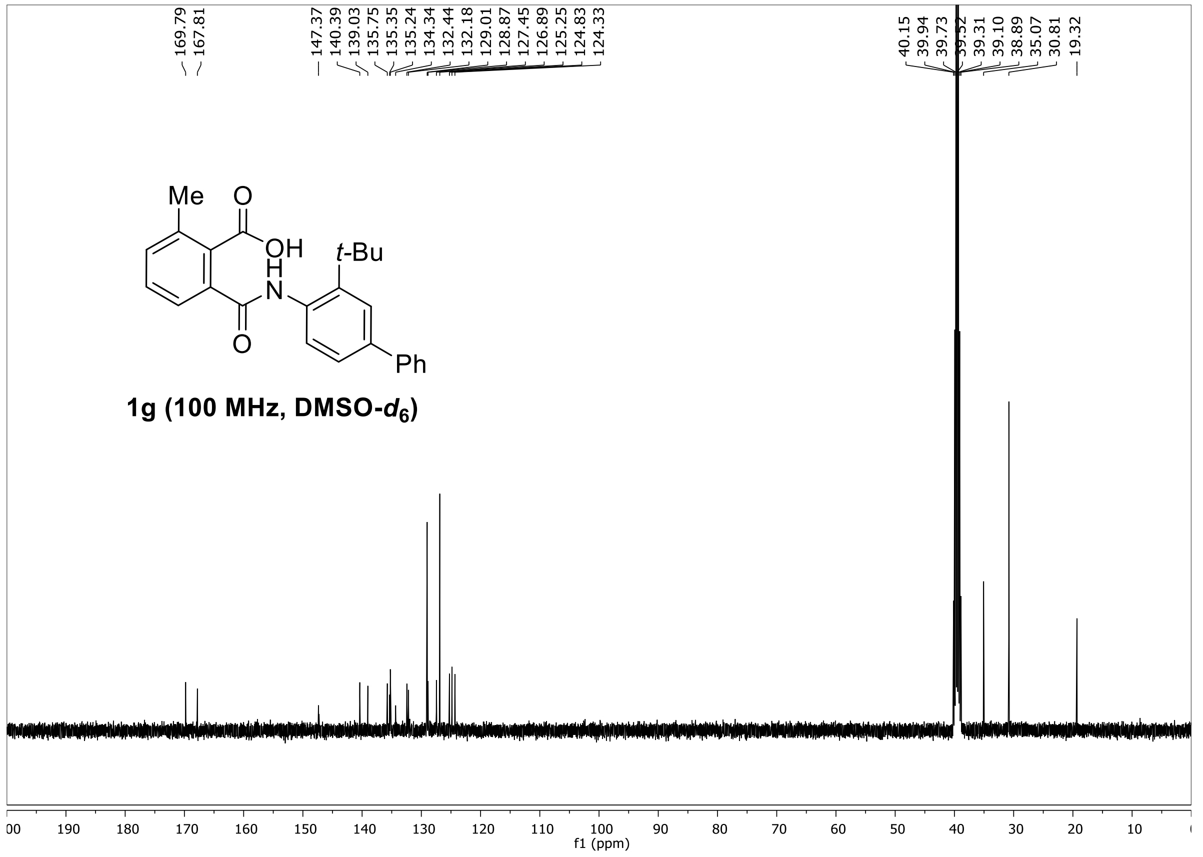

2-((2-(*tert*-Butyl)-4-(ethoxycarbonyl)phenyl)carbamoyl)-6-methylbenzoic acid (1h)

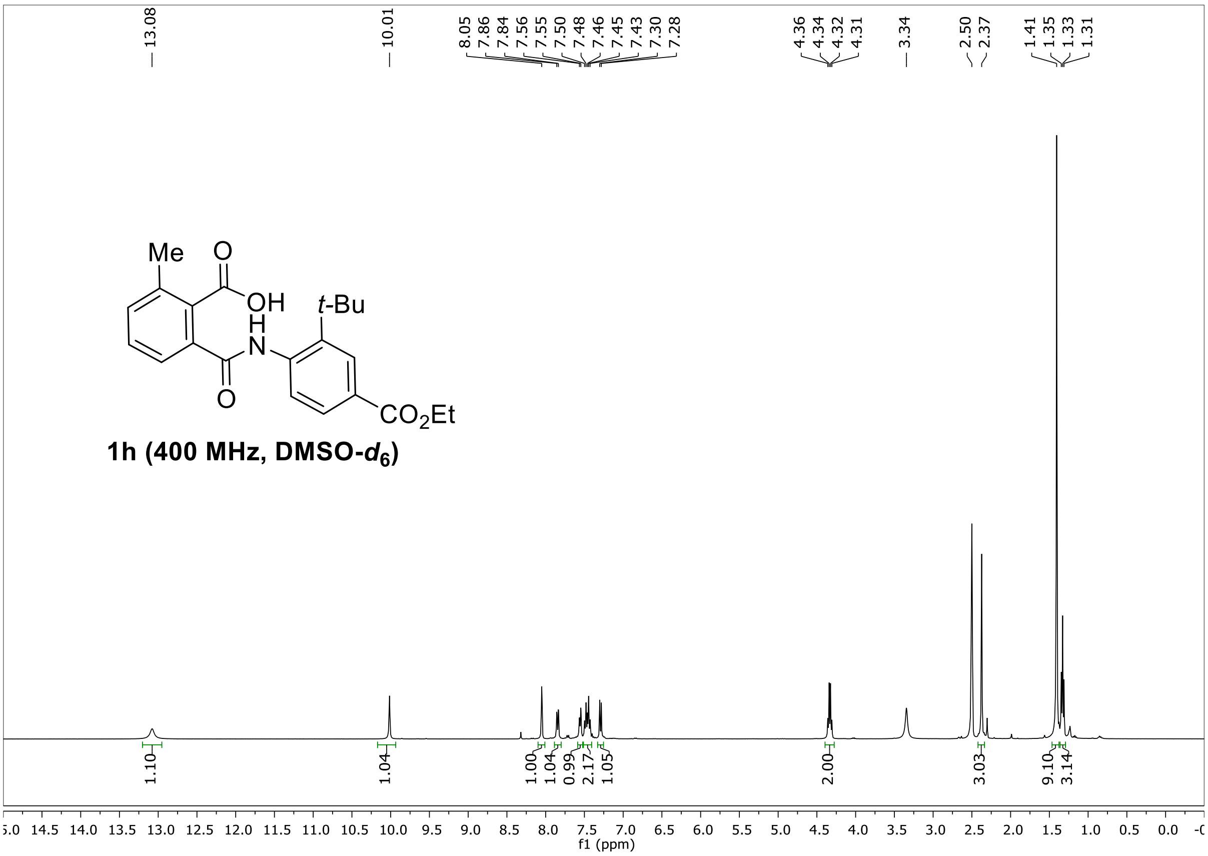

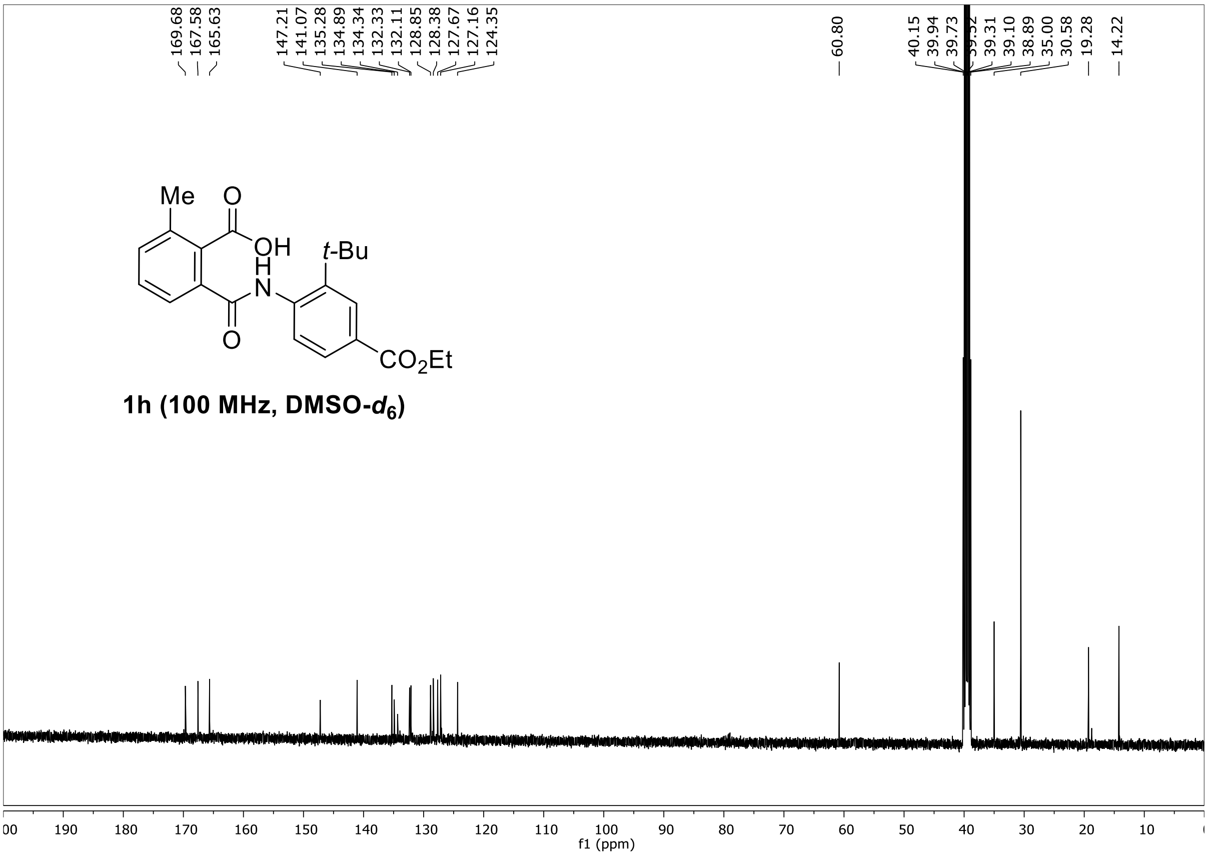

2-((2-(*tert*-Butyl)-4-(thiophen-3-yl)phenyl)carbamoyl)-6-methylbenzoic acid (1i)

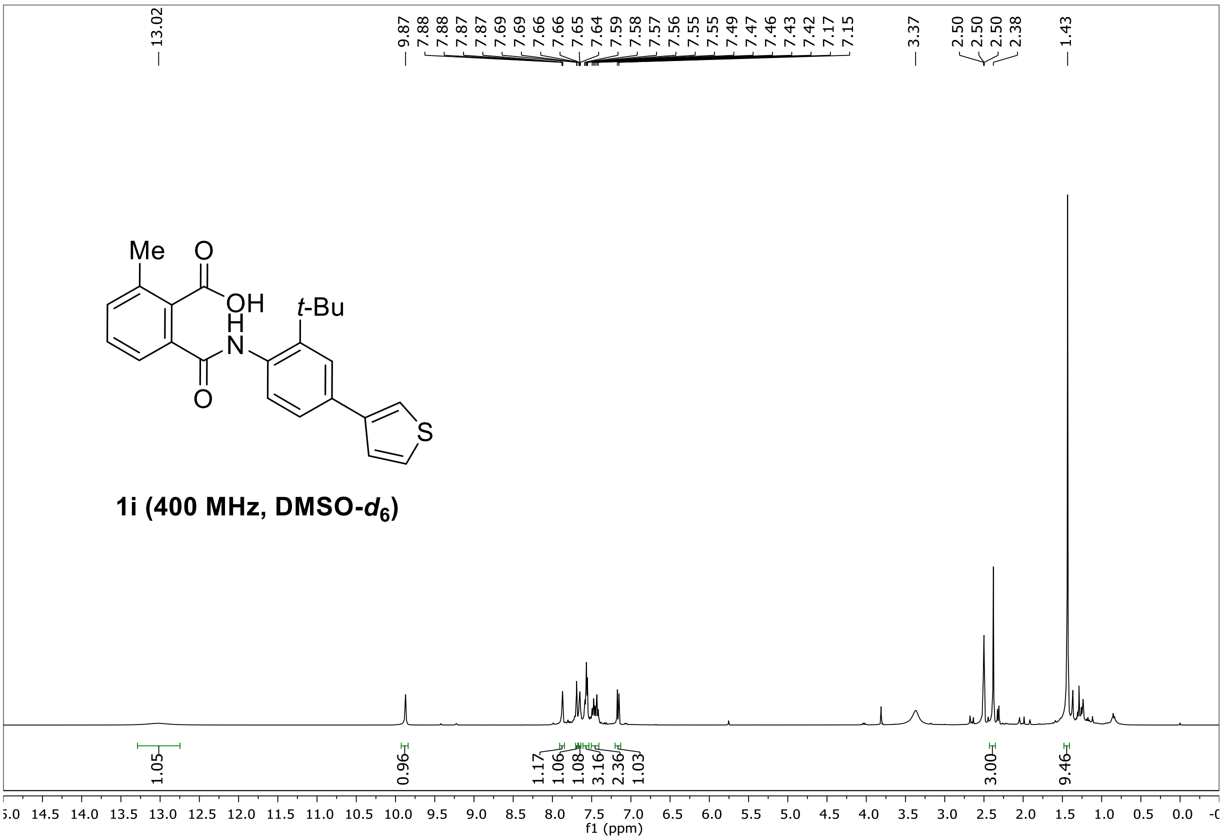

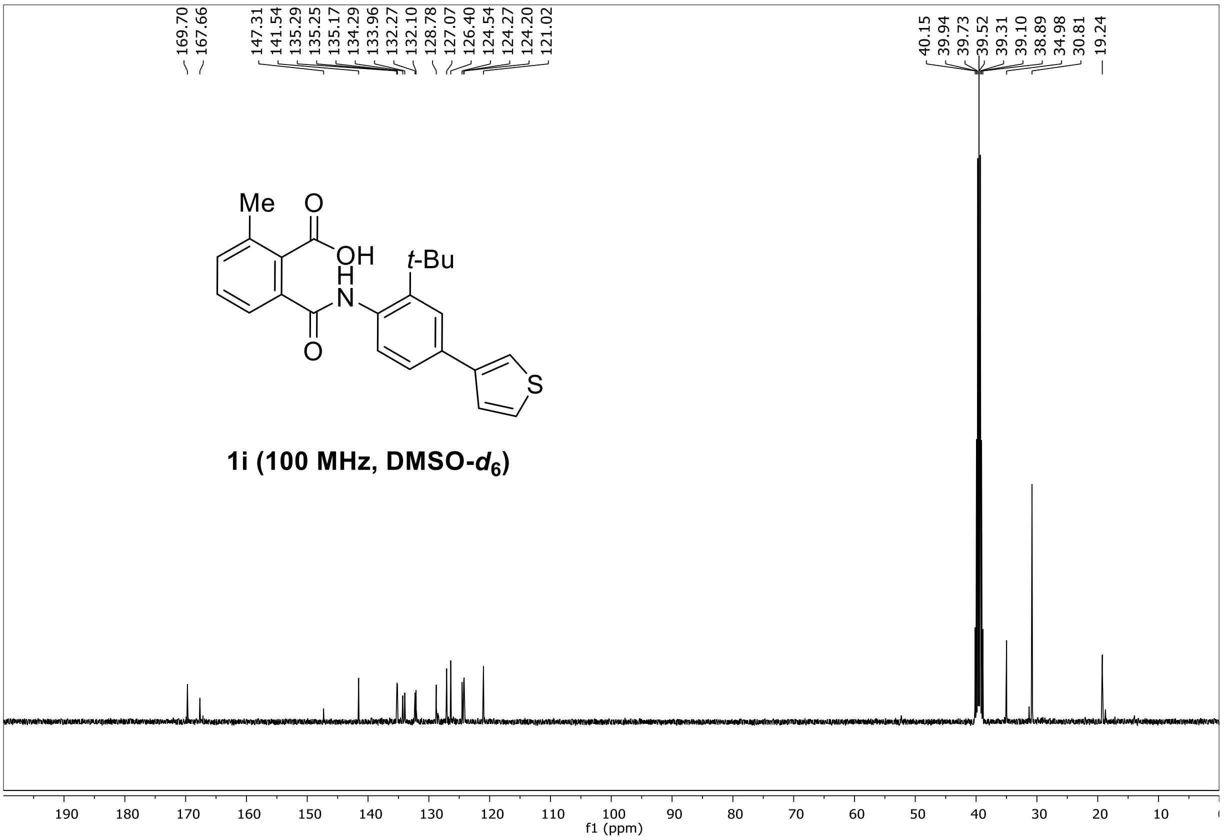

(*E*)-2-((2-(*tert*-Butyl)-4-styrylphenyl)carbamoyl)-6-methylbenzoic acid (1j)

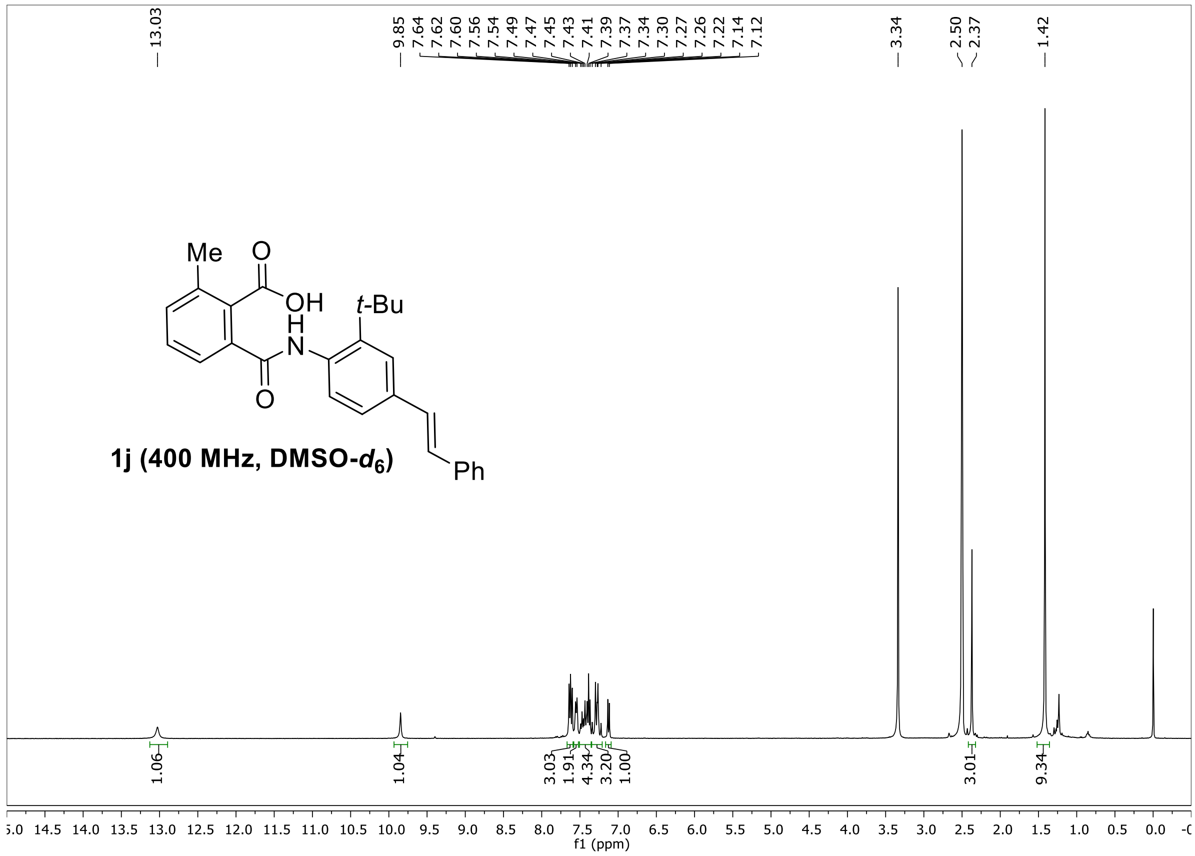

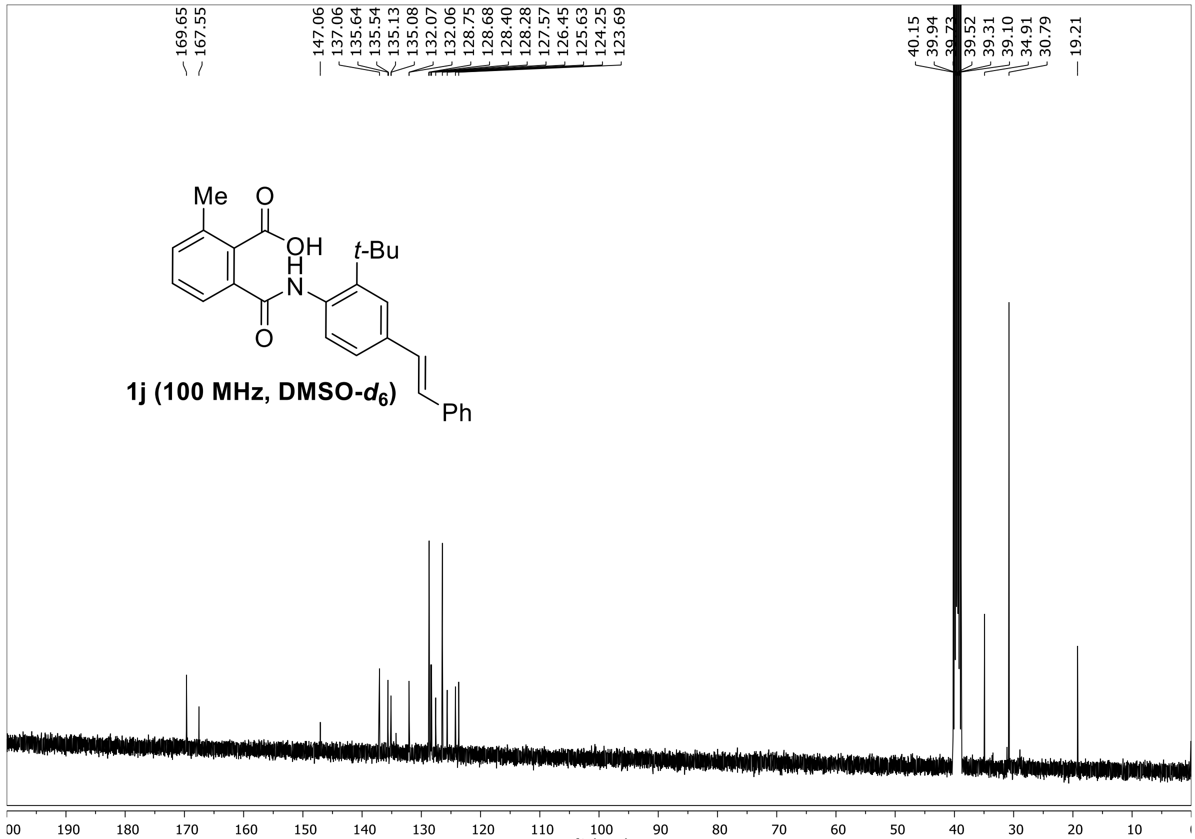

(*E*)-2-((2-(*tert*-Butyl)-4-(3-methoxy-3-oxoprop-1-en-1-yl)phenyl)carbamoyl)-6-methylbenzoic acid (1k)

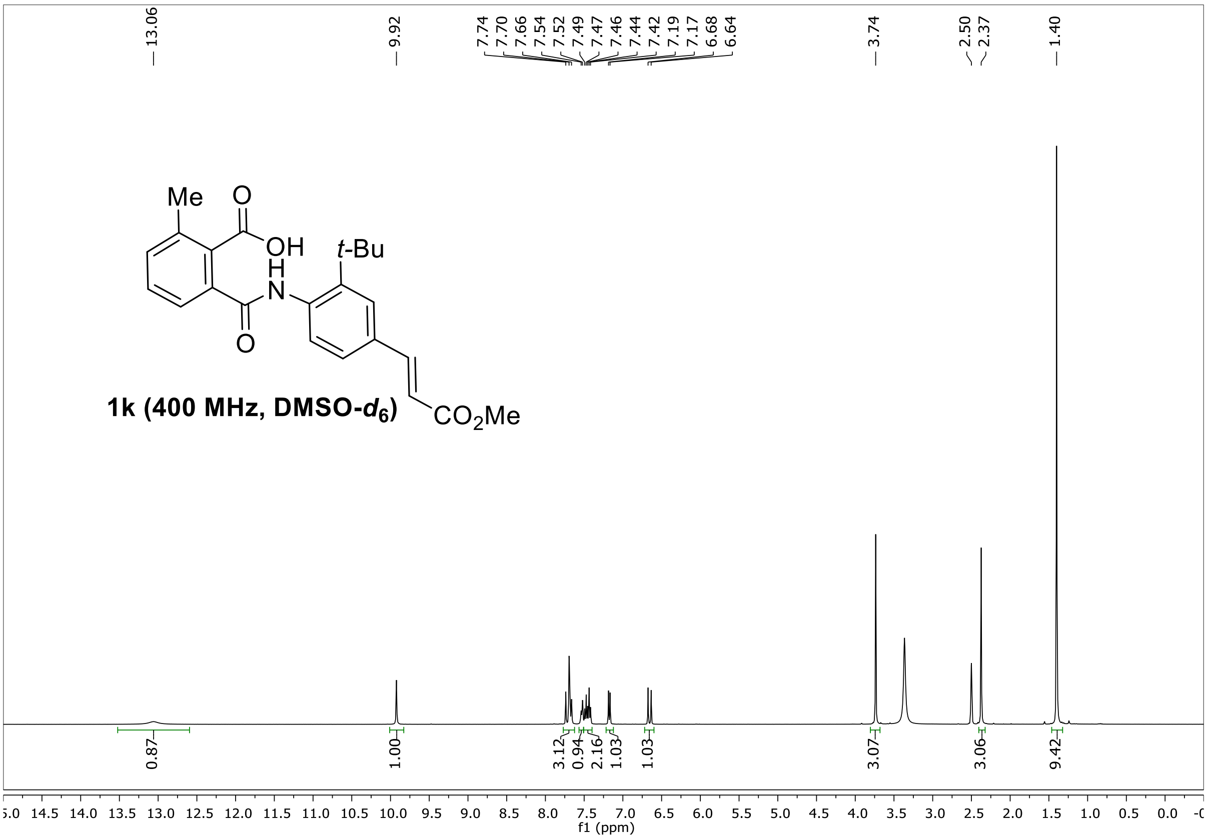

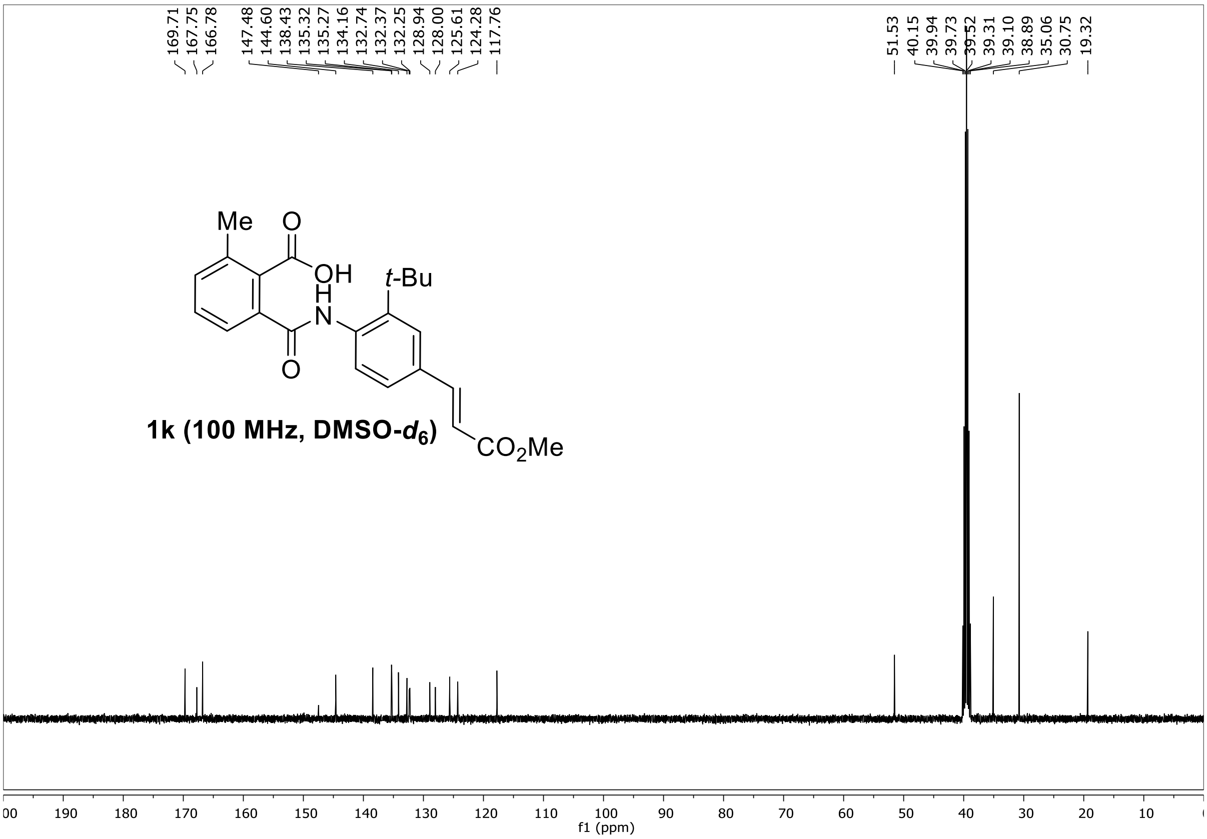

2-((2-(*tert*-Butyl)-4-(phenylethynyl)phenyl)carbamoyl)-6-methylbenzoic acid (1l)

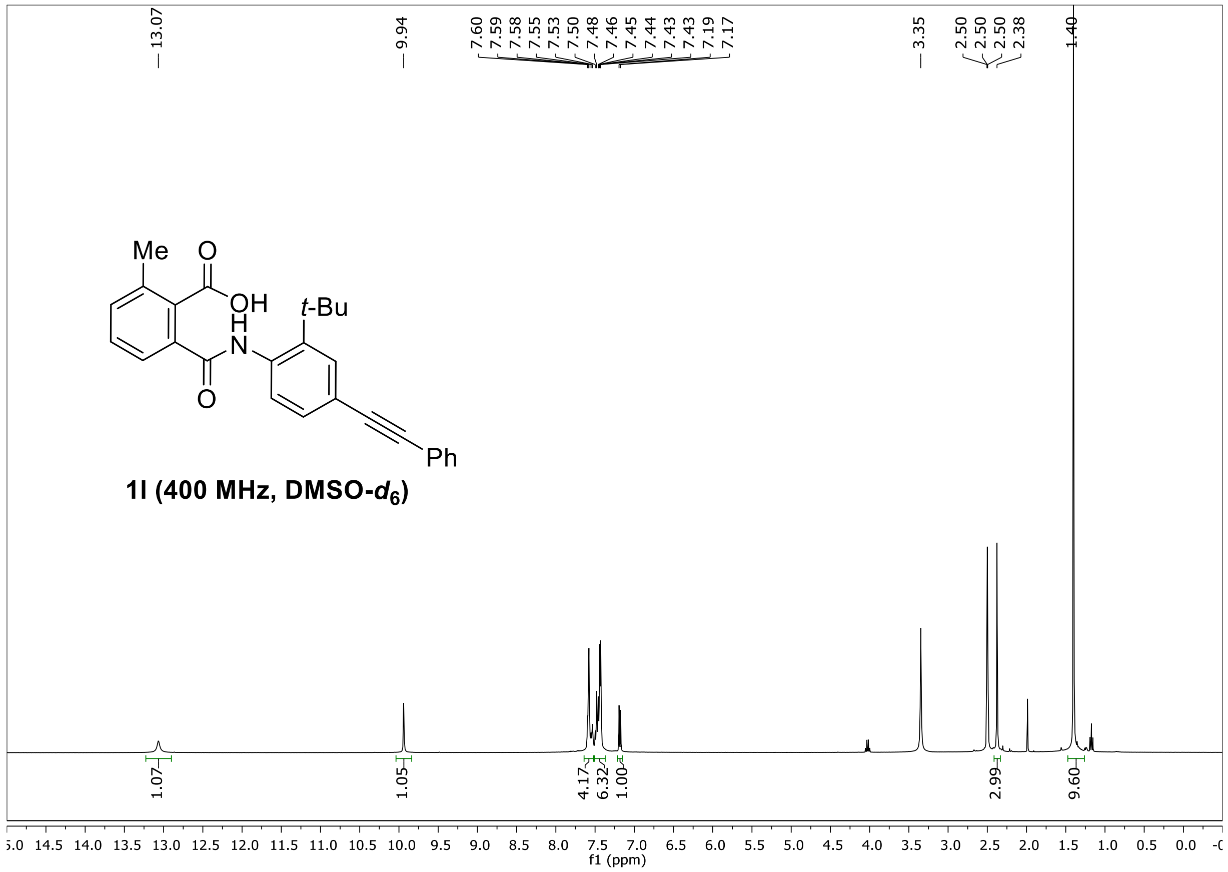

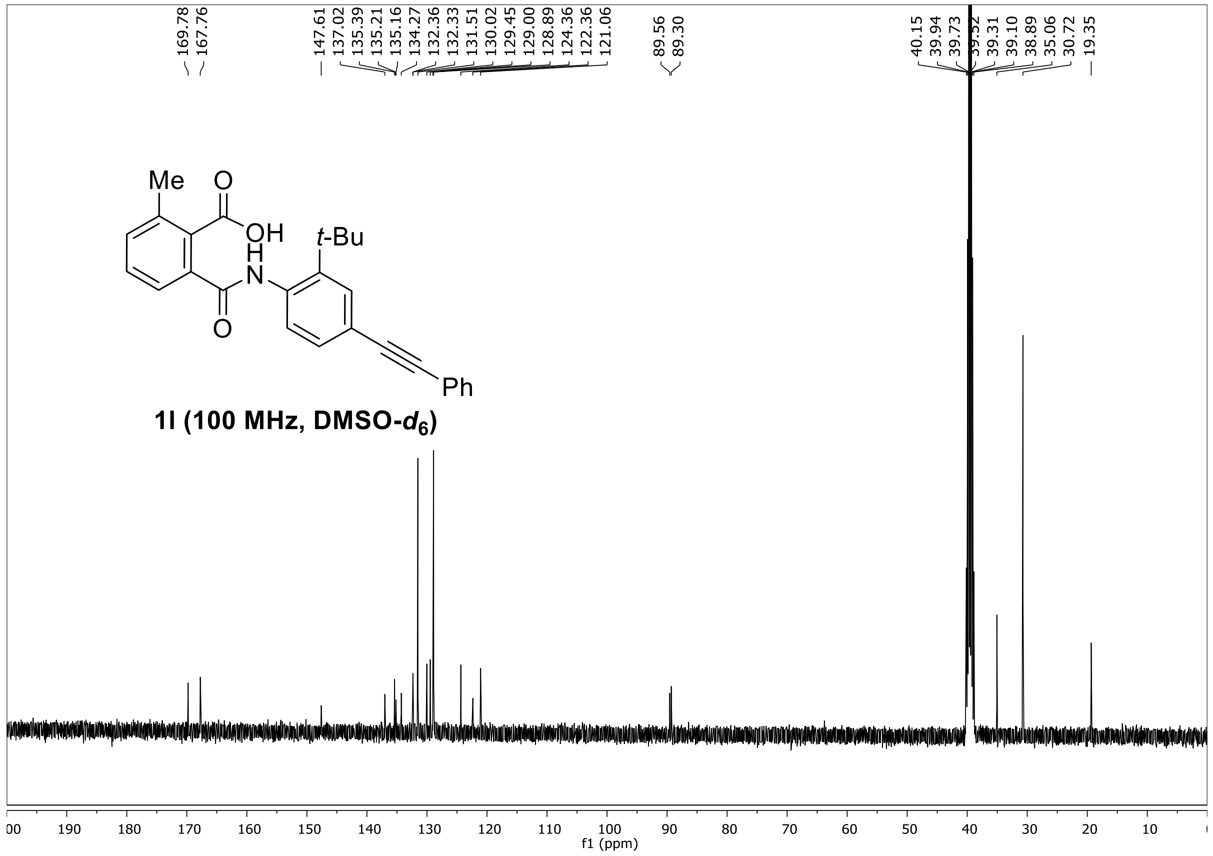

2-((2-(*tert*-Butyl)-5-nitrophenyl)carbamoyl)-6-methylbenzoic acid (1m)

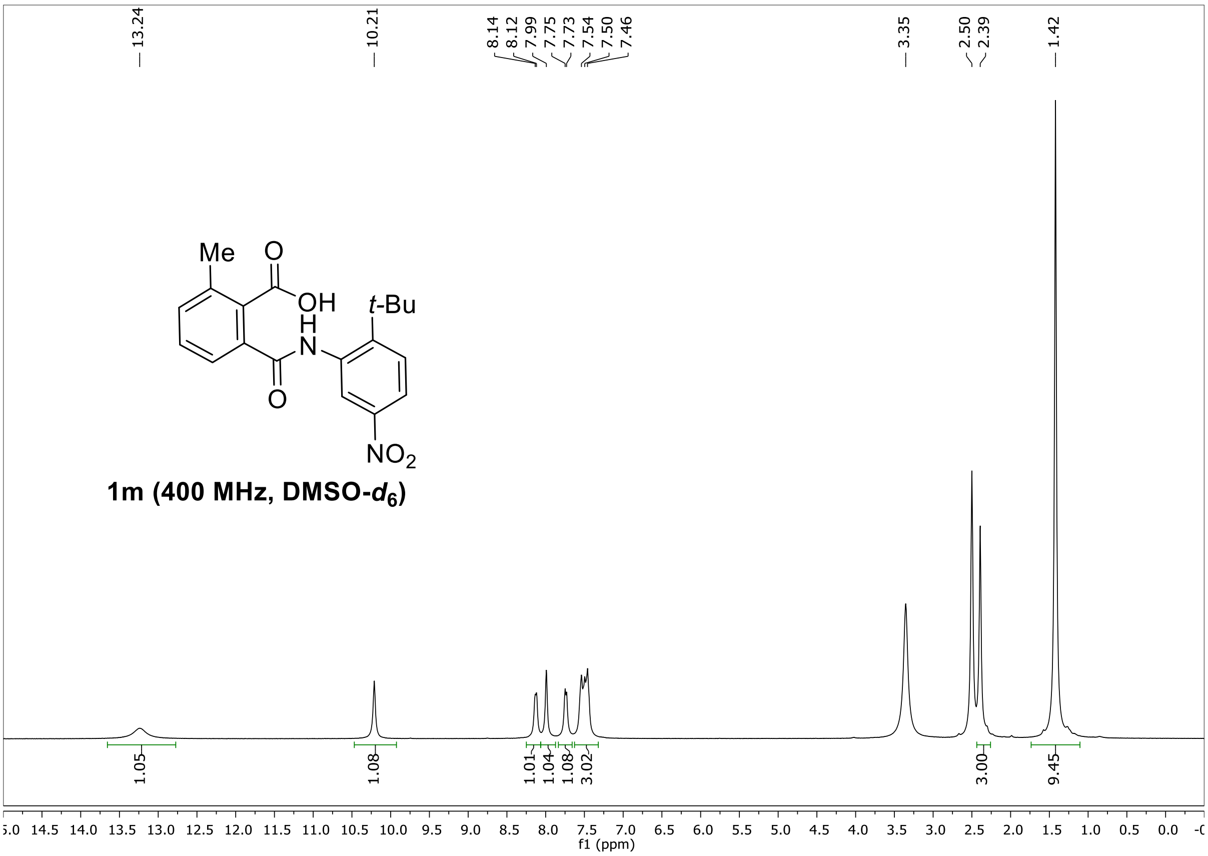

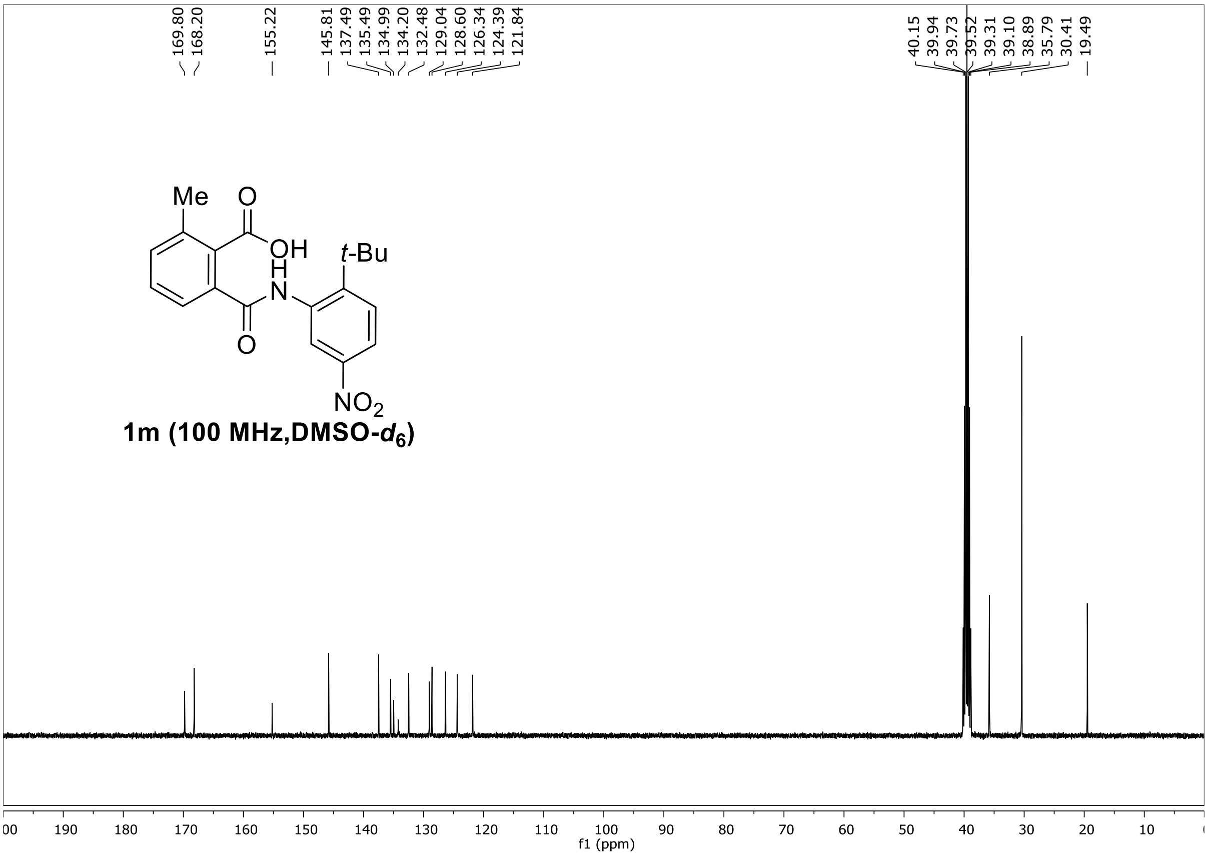

2-Methyl-6-((4-methyl-2-(2-phenylpropan-2-yl)phenyl)carbamoyl)benzoic acid (1n)

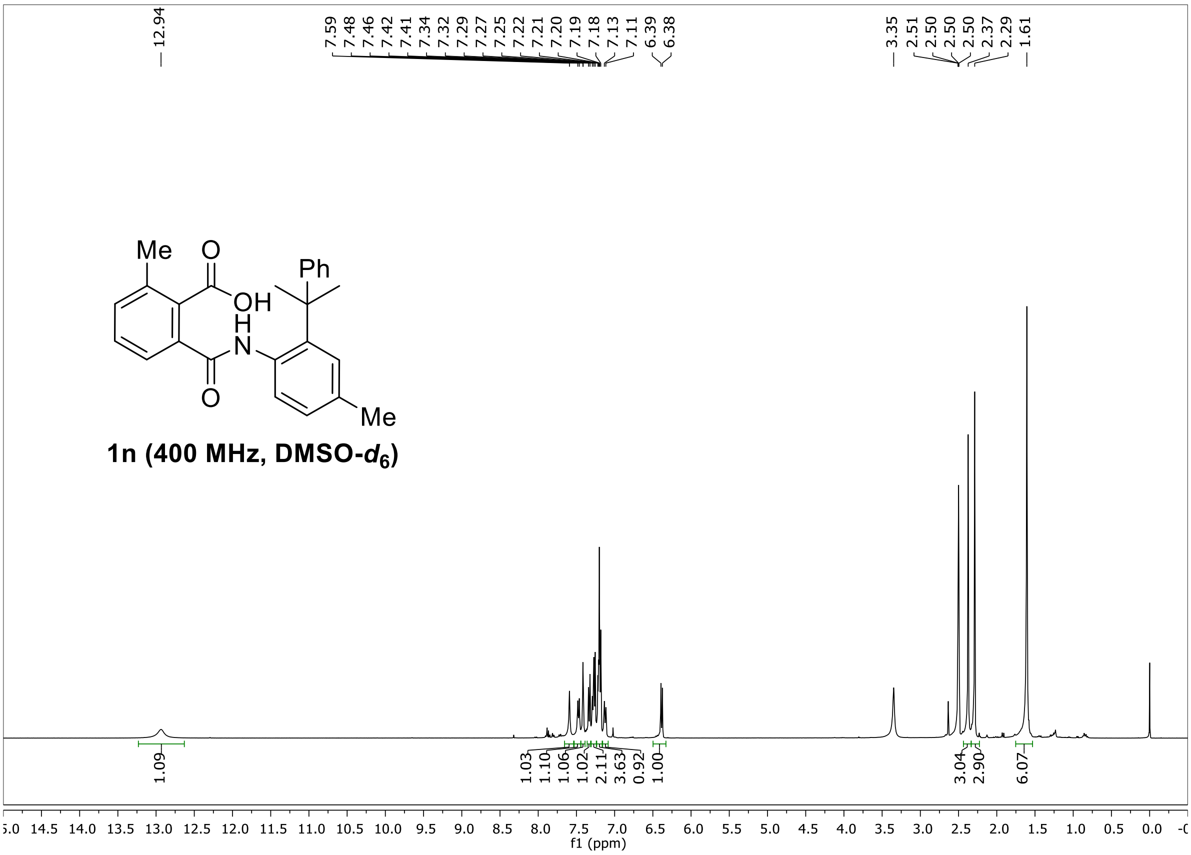

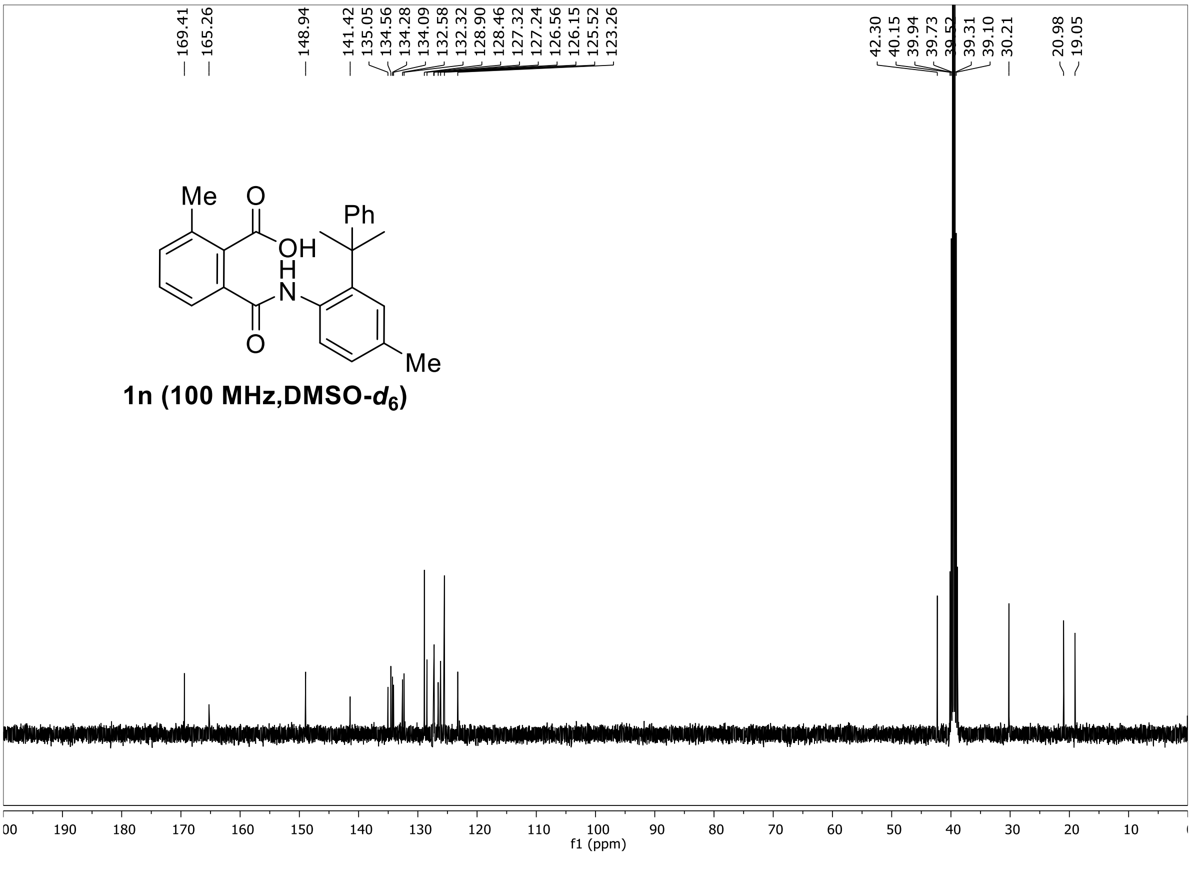

2-((2-(1-Methoxy-2-methylpropan-2-yl)phenyl)carbamoyl)-6-methylbenzoic acid (1o)

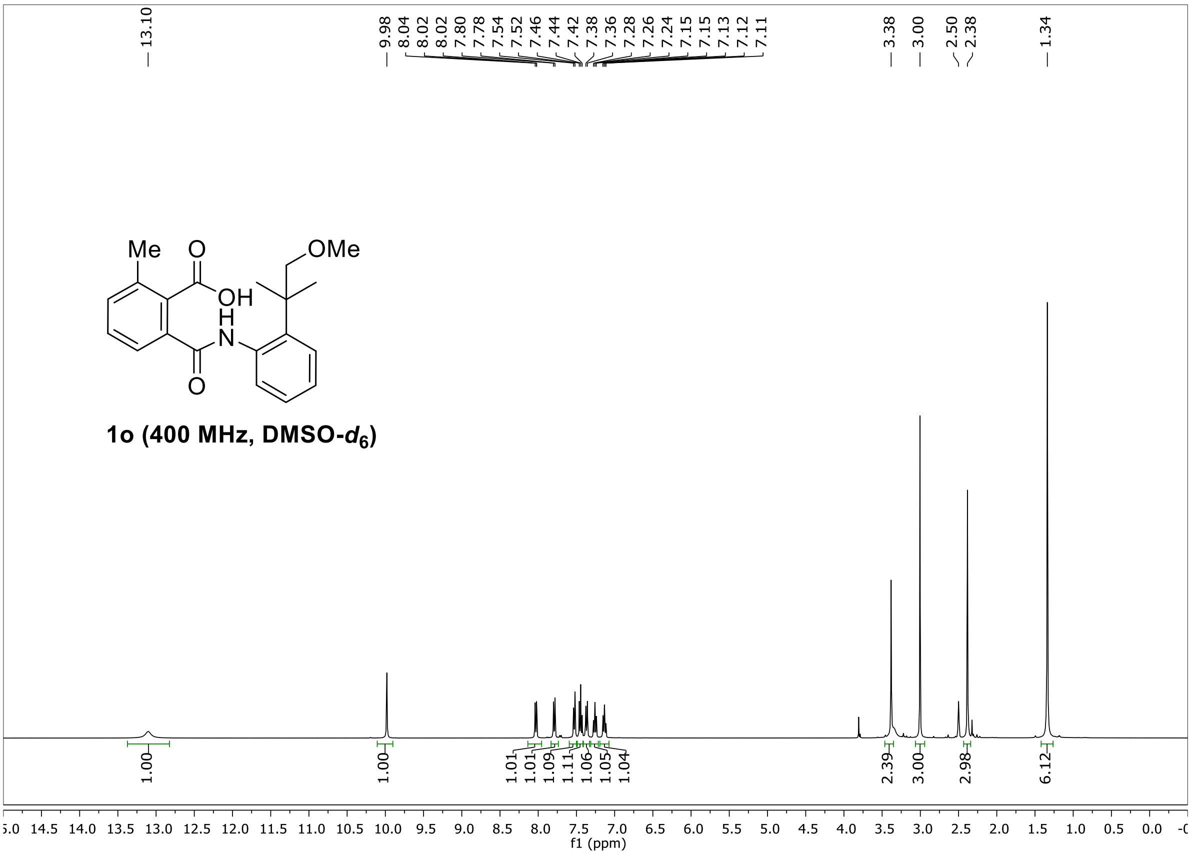

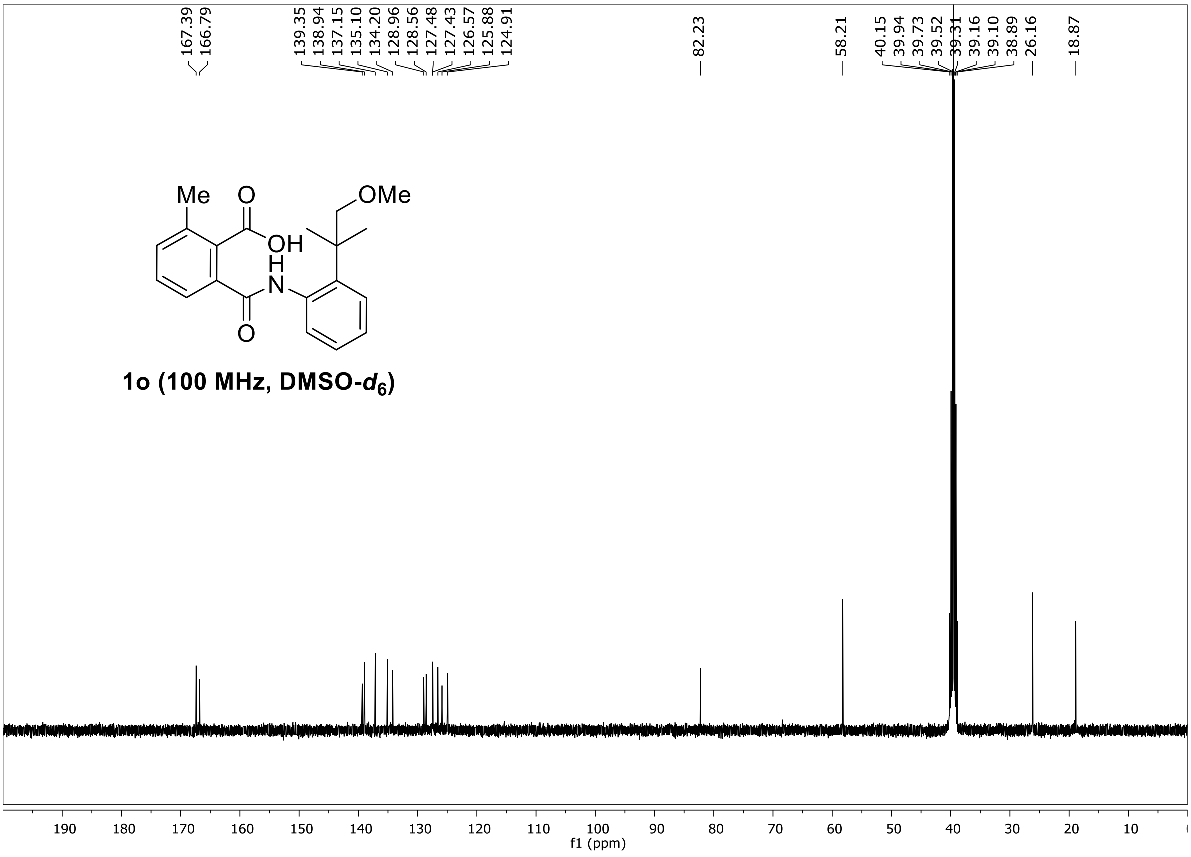

2-((2-(Methoxydiphenylmethyl)phenyl)carbamoyl)-6-methylbenzoic acid (1p)

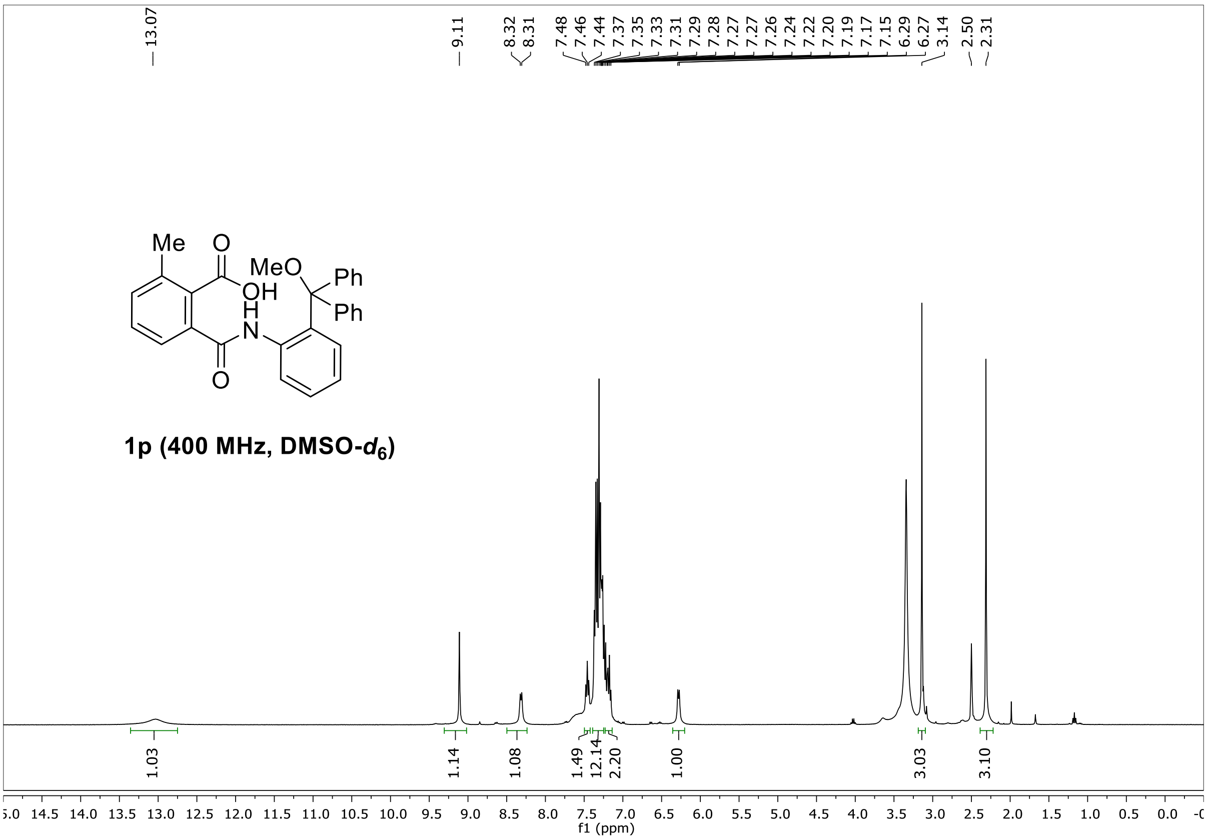

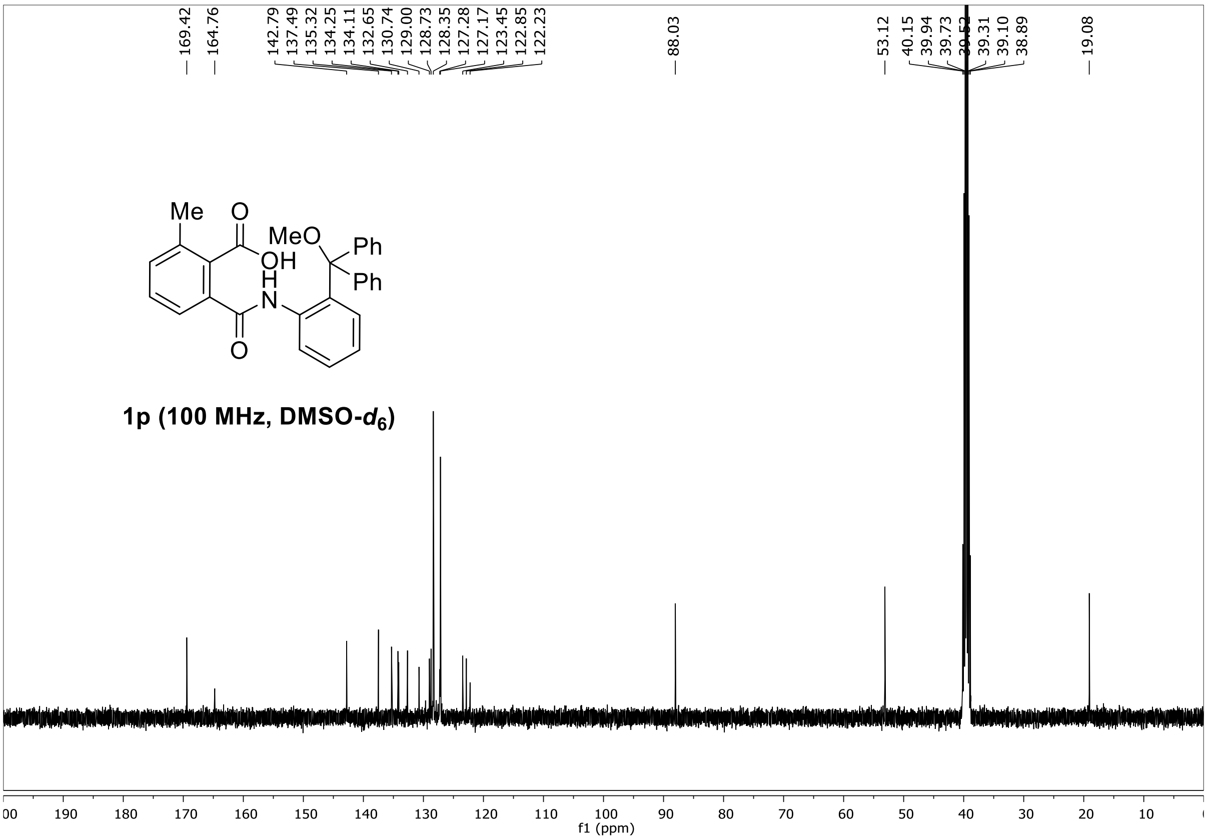

2-((2-(Di(thiophen-2-yl)((trimethylsilyl)oxy)methyl)phenyl)carbamoyl)-6-methylbenzoic acid (1q)

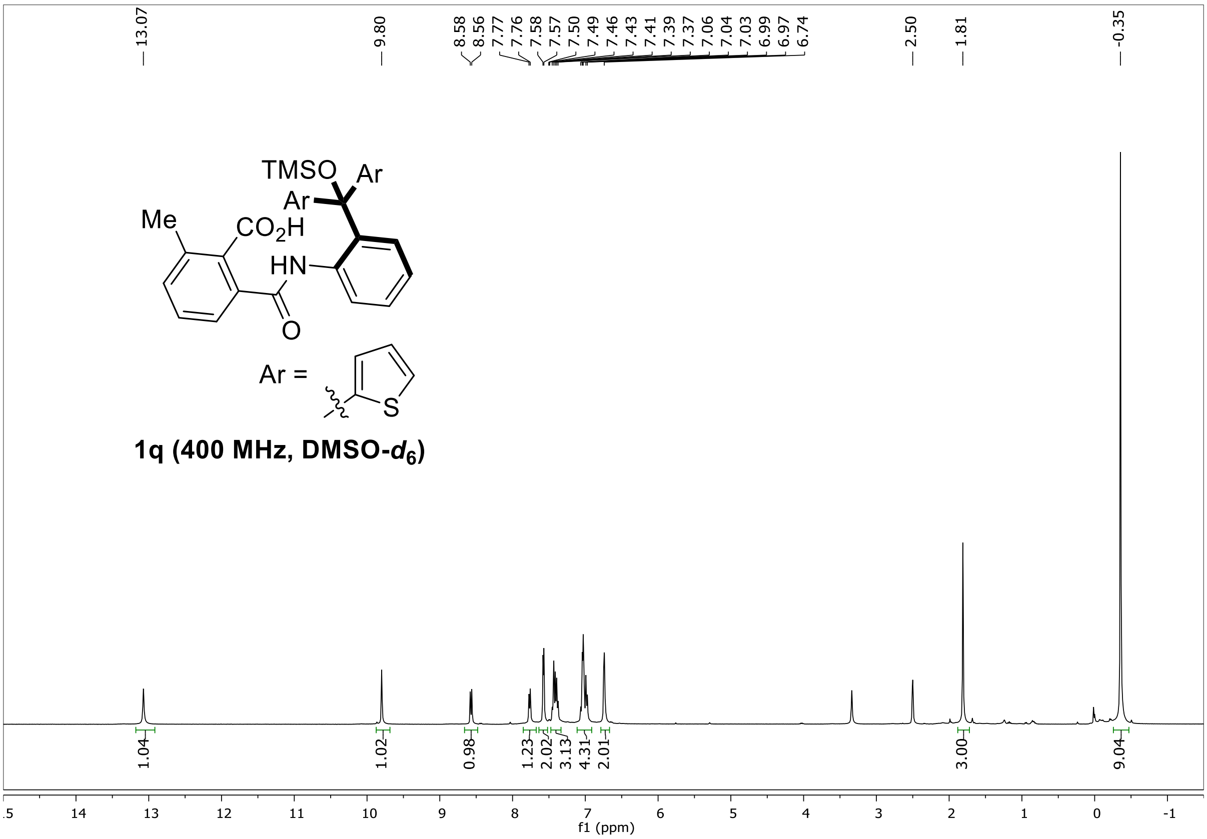

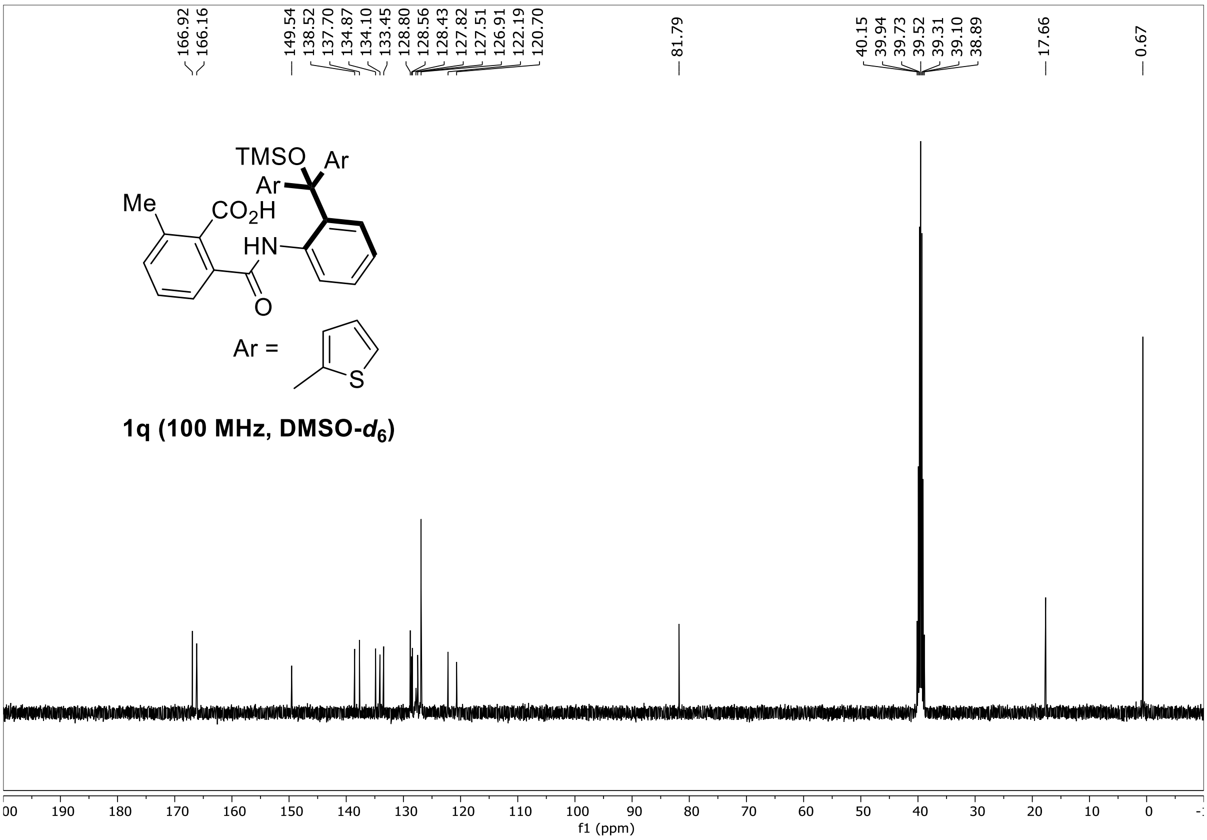

2-Methyl-6-((2-(phenylsulfonyl)phenyl)carbamoyl)benzoic acid (1r)

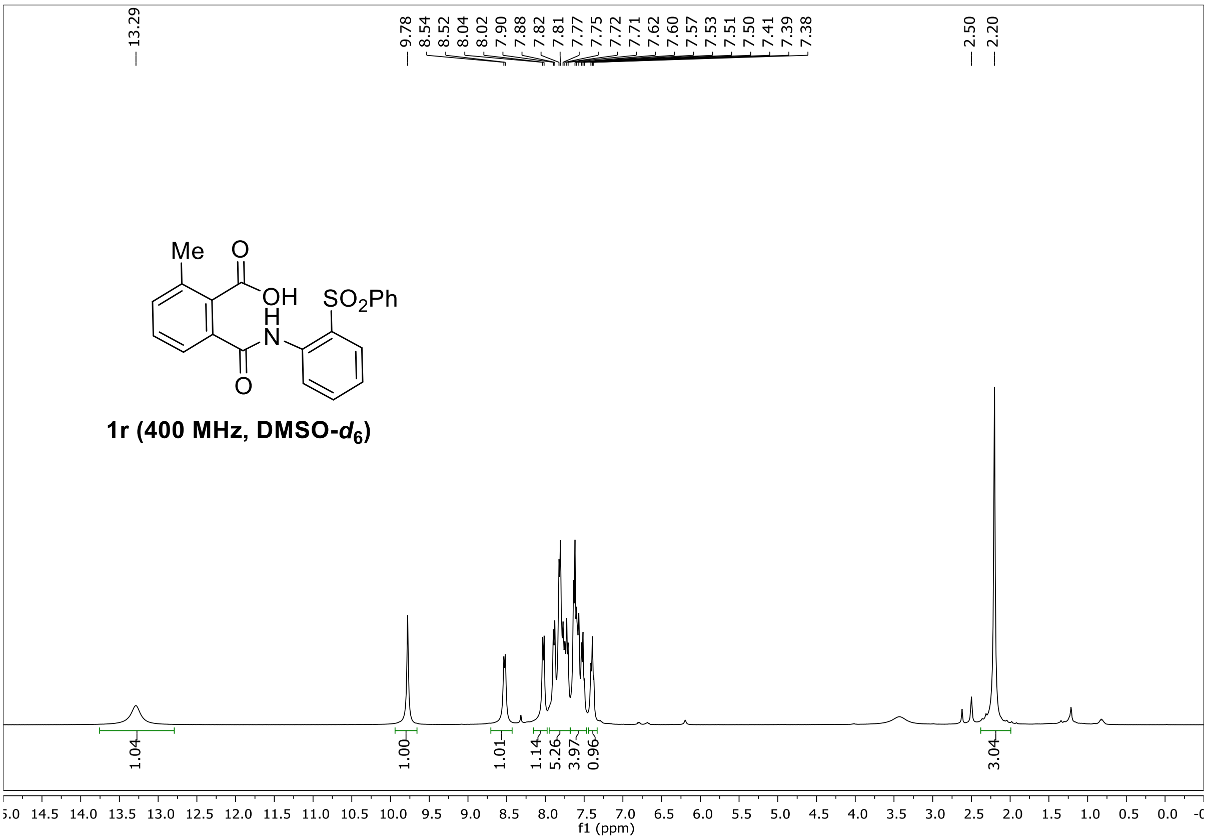

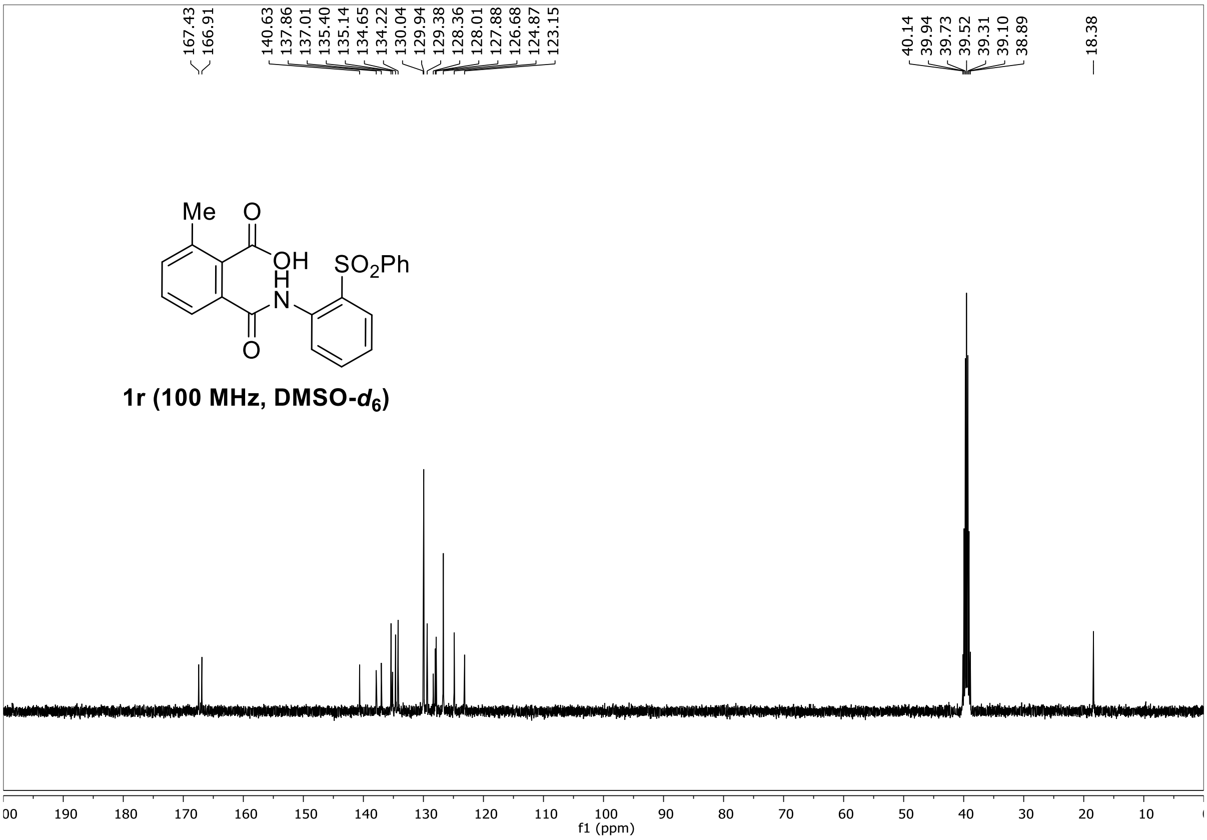

2-((2-(*tert*-Butyl)phenyl)carbamoyl)-6-methoxybenzoic acid (1s)

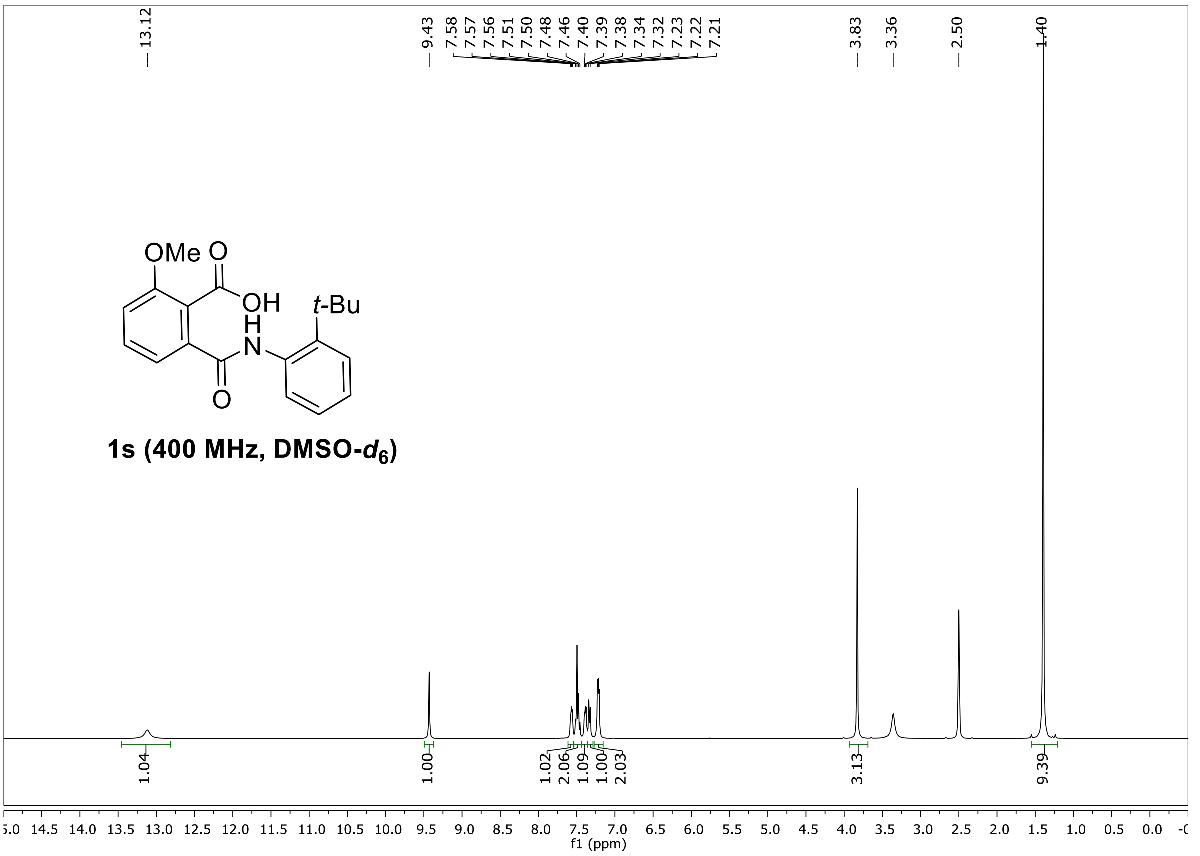

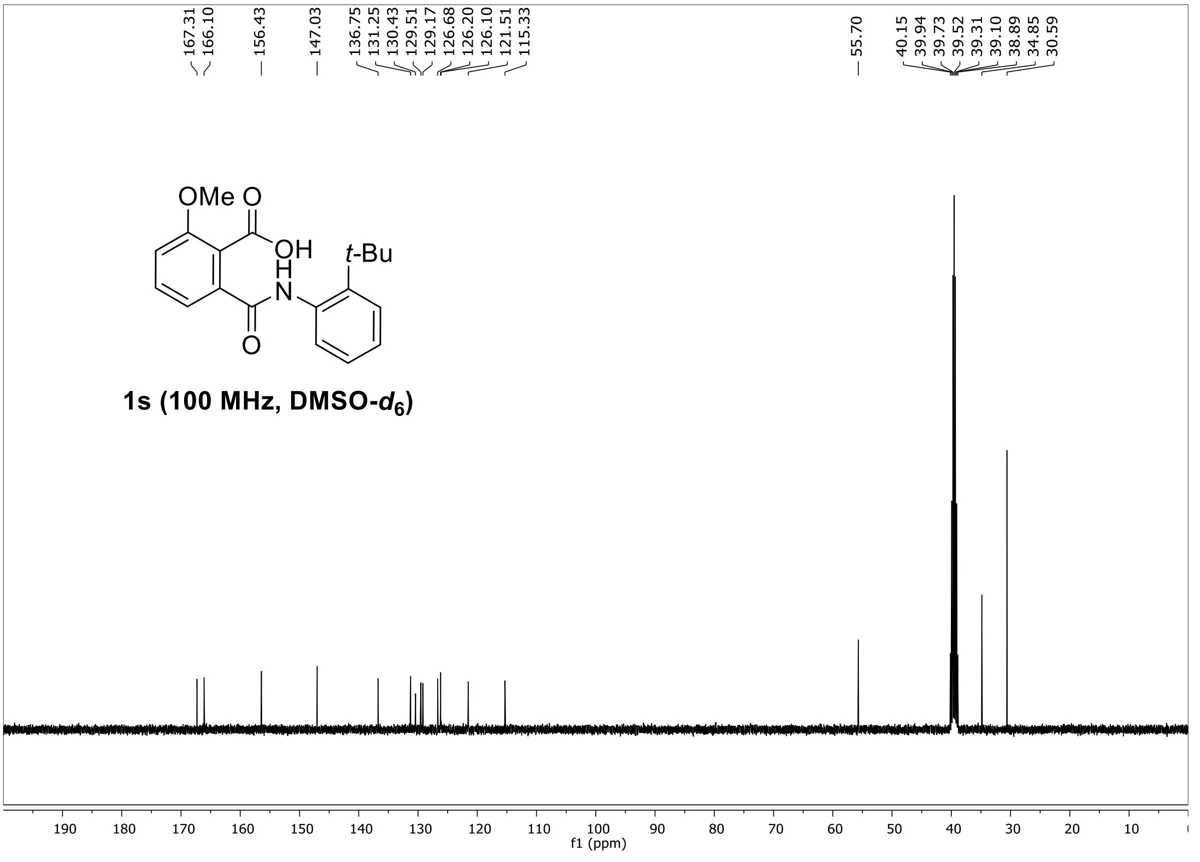

2-(Benzyloxy)-6-((2-(*tert*-butyl)phenyl)carbamoyl)benzoic acid (1t)

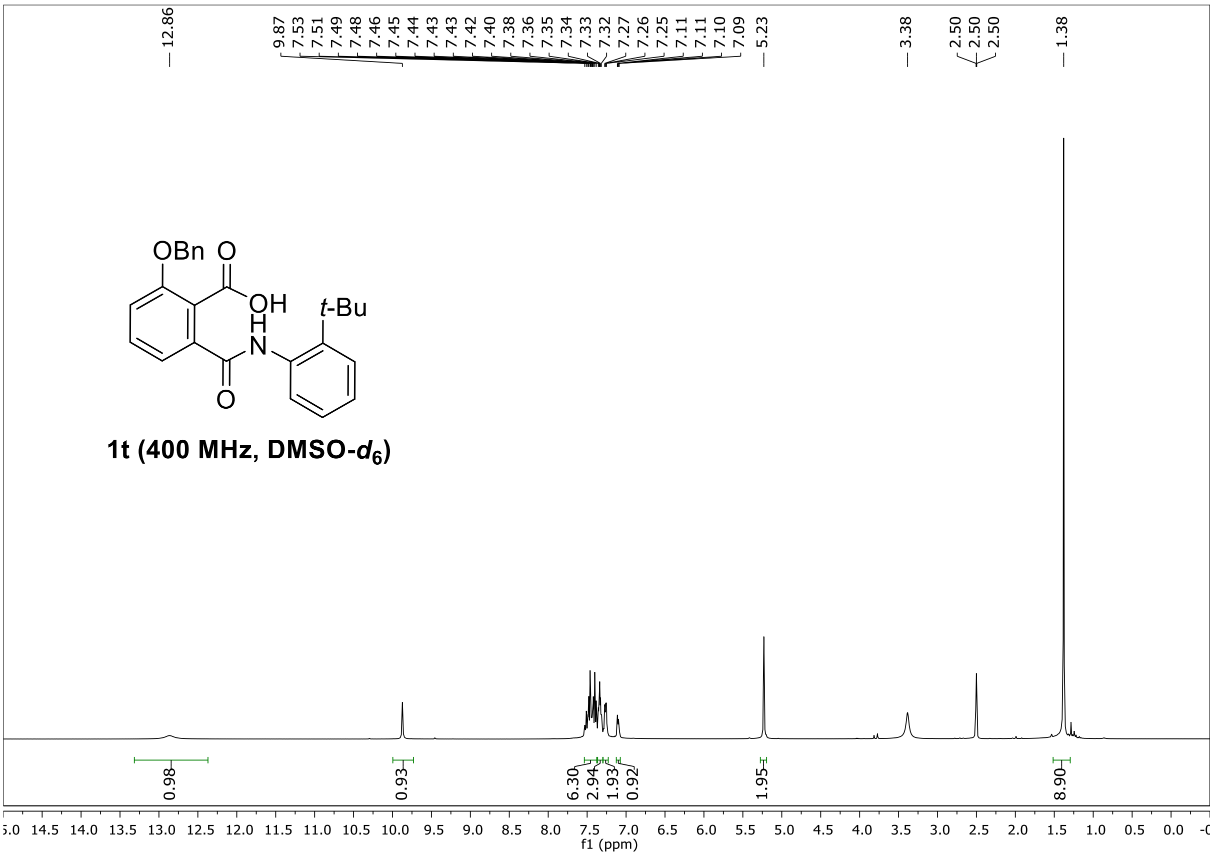

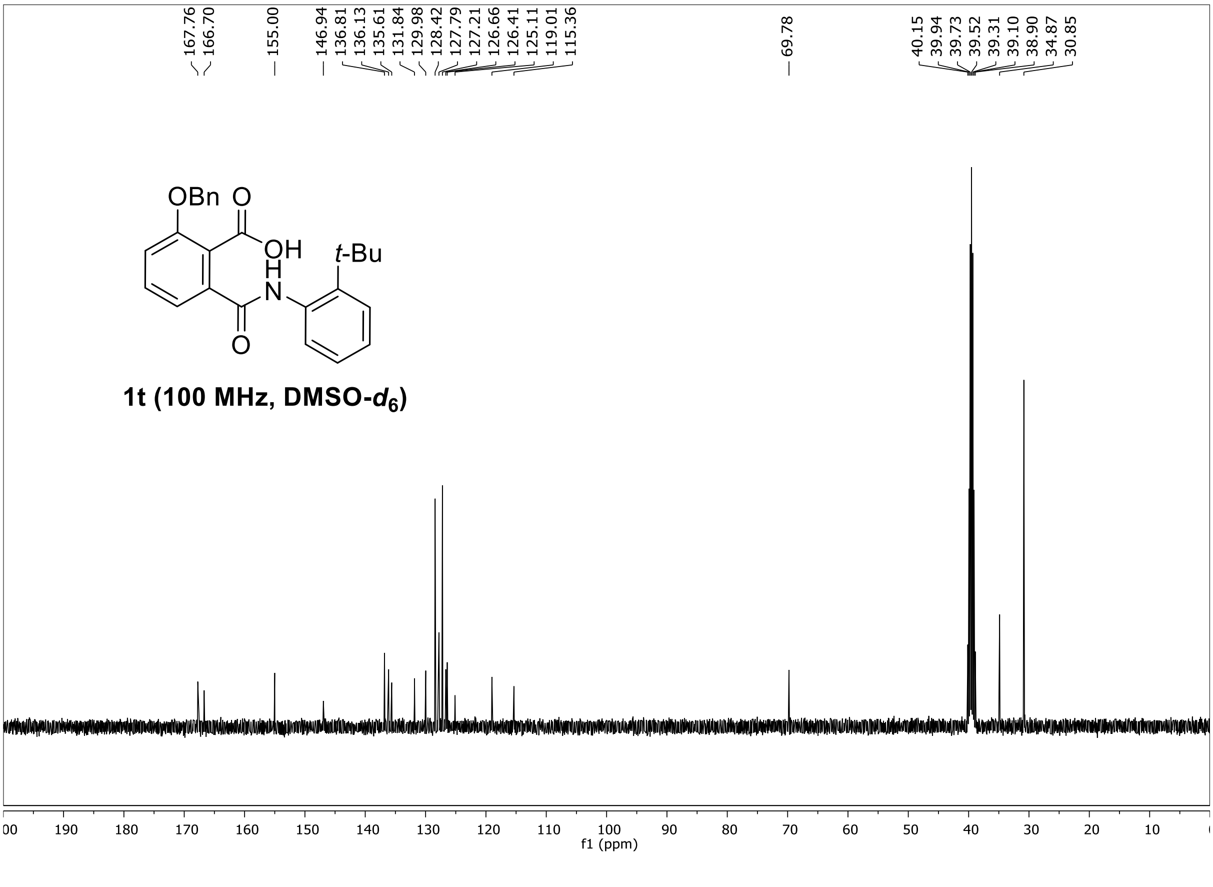

2-(Allyloxy)-6-((2-(*tert*-butyl)phenyl)carbamoyl)benzoic acid (1u)

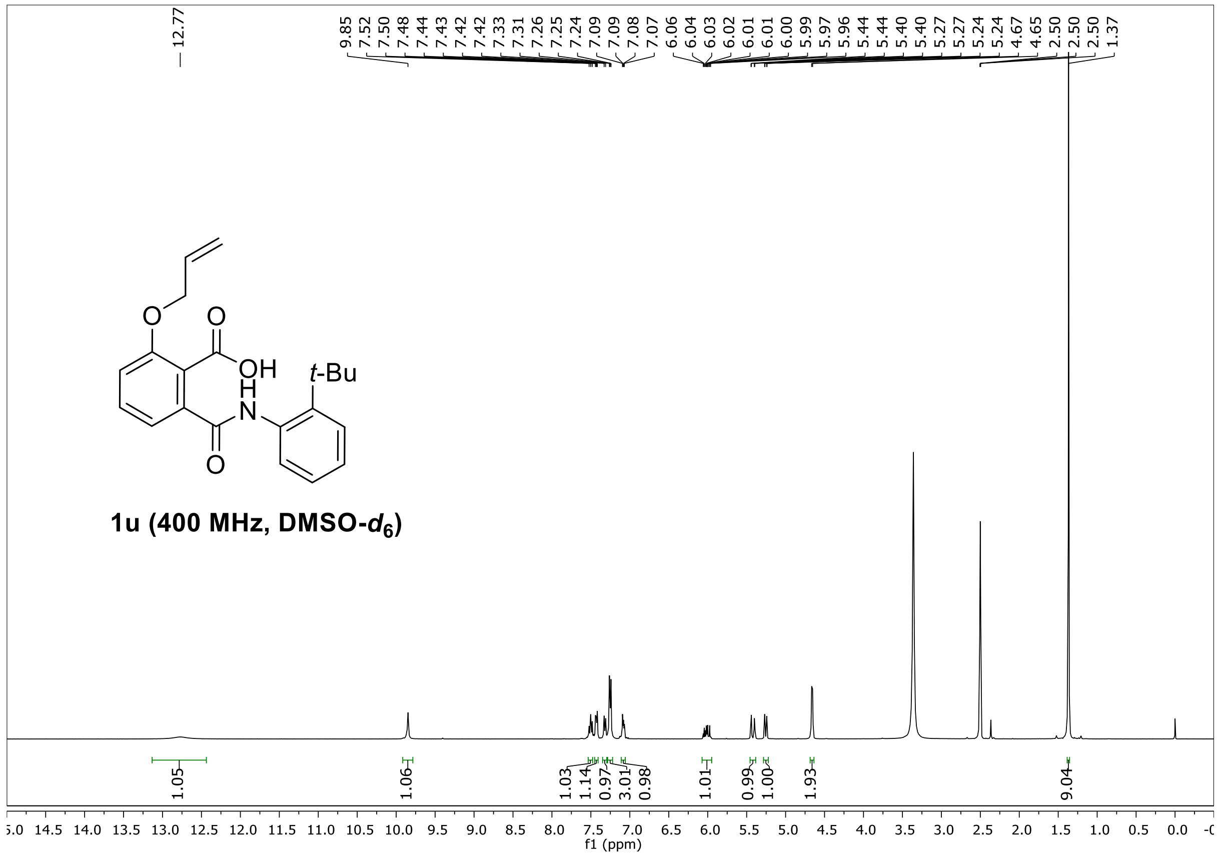

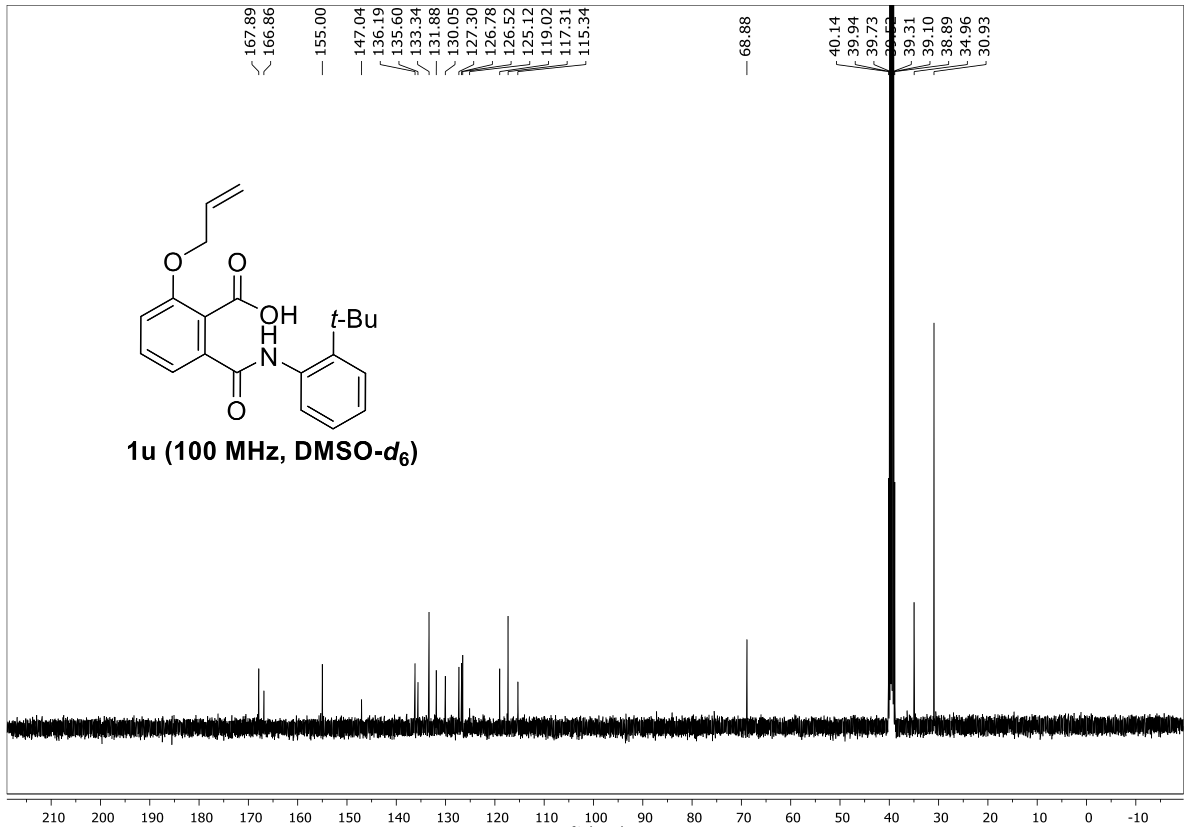

2-((2-(*tert*-Butyl)phenyl)carbamoyl)-6-hydroxybenzoic acid (1v)

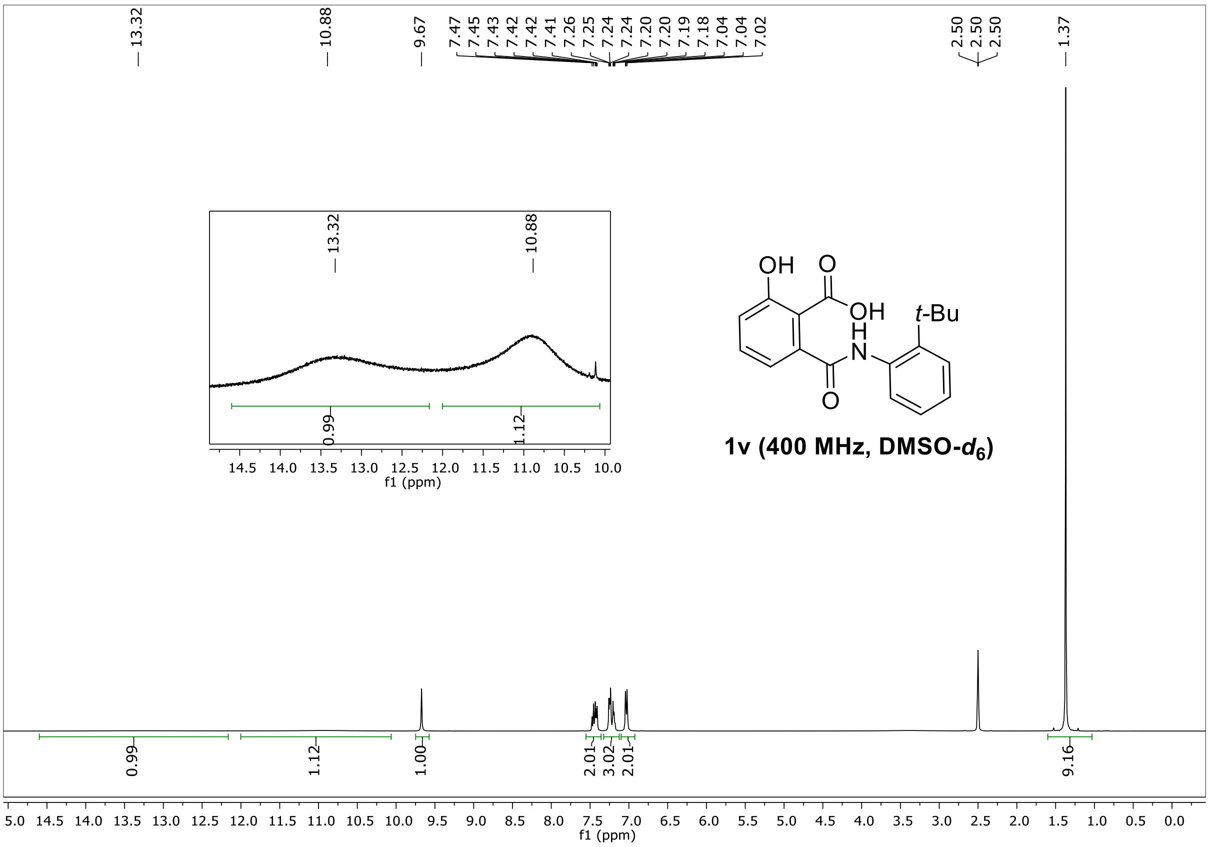

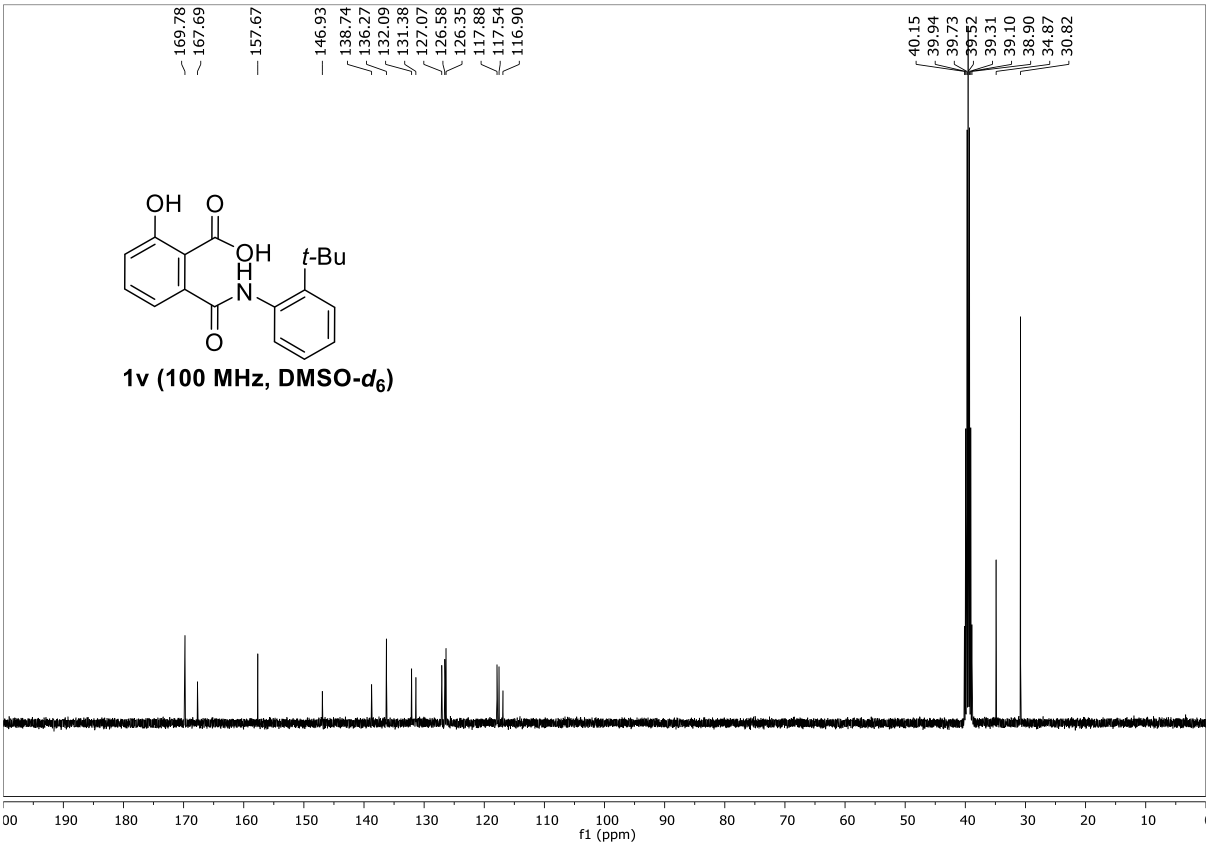

2-((2-(*tert*-Butyl)phenyl)carbamoyl)-6-chlorobenzoic acid (1w)

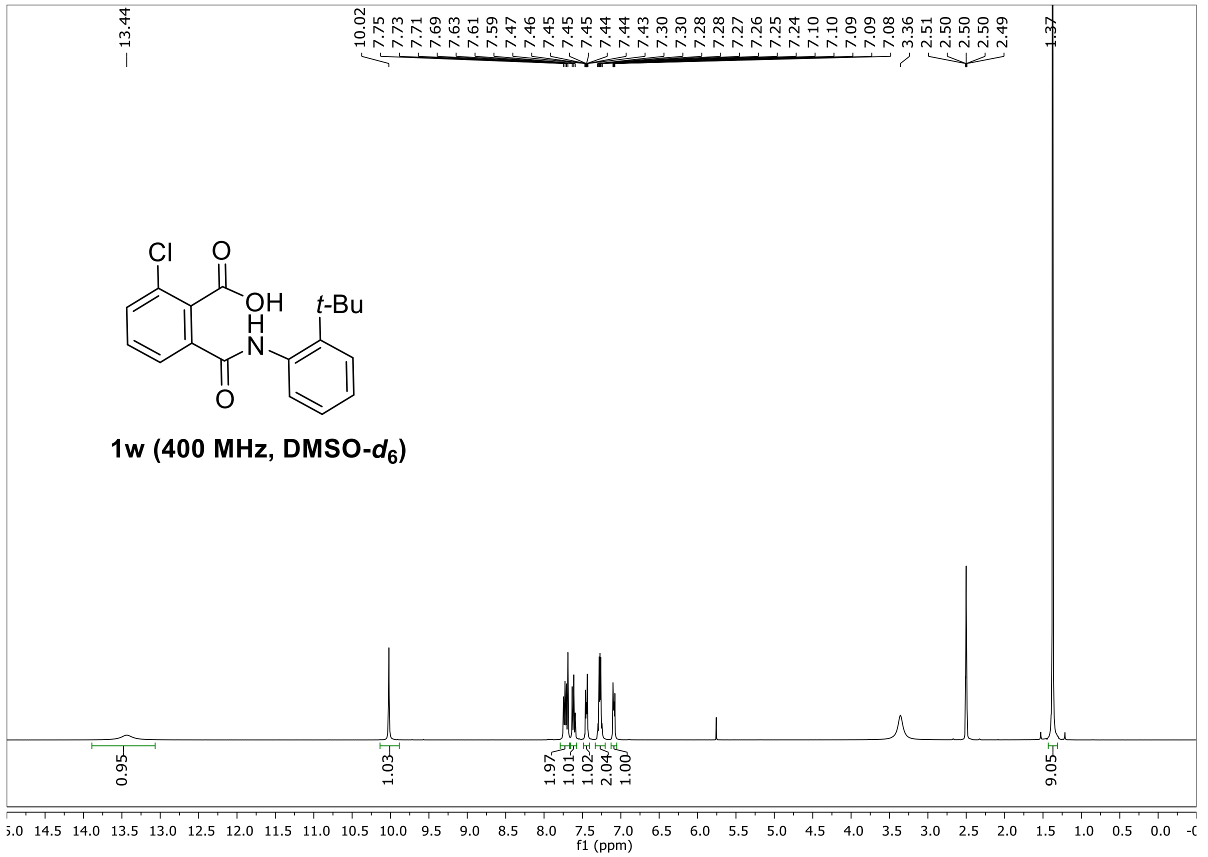

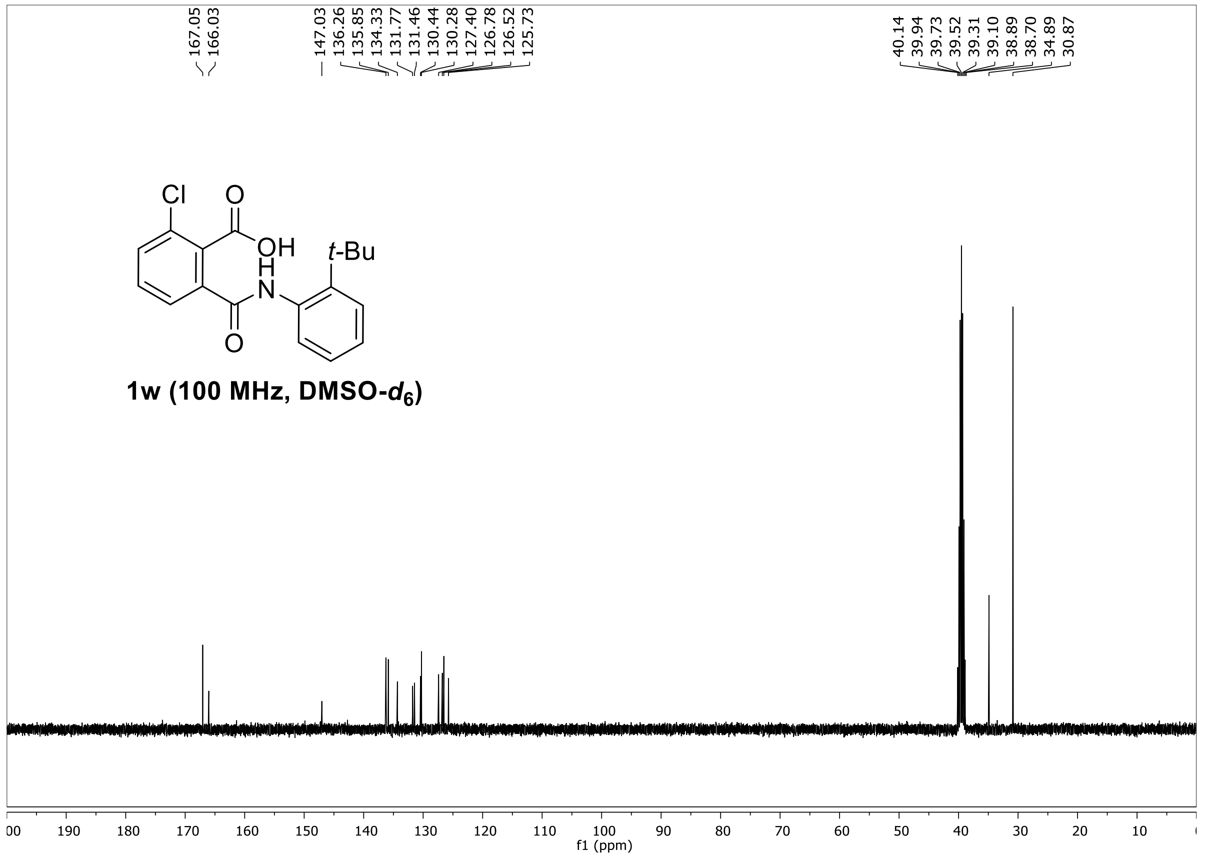

2-Bromo-6-((2-(*tert*-butyl)phenyl)carbamoyl)benzoic acid (1x)

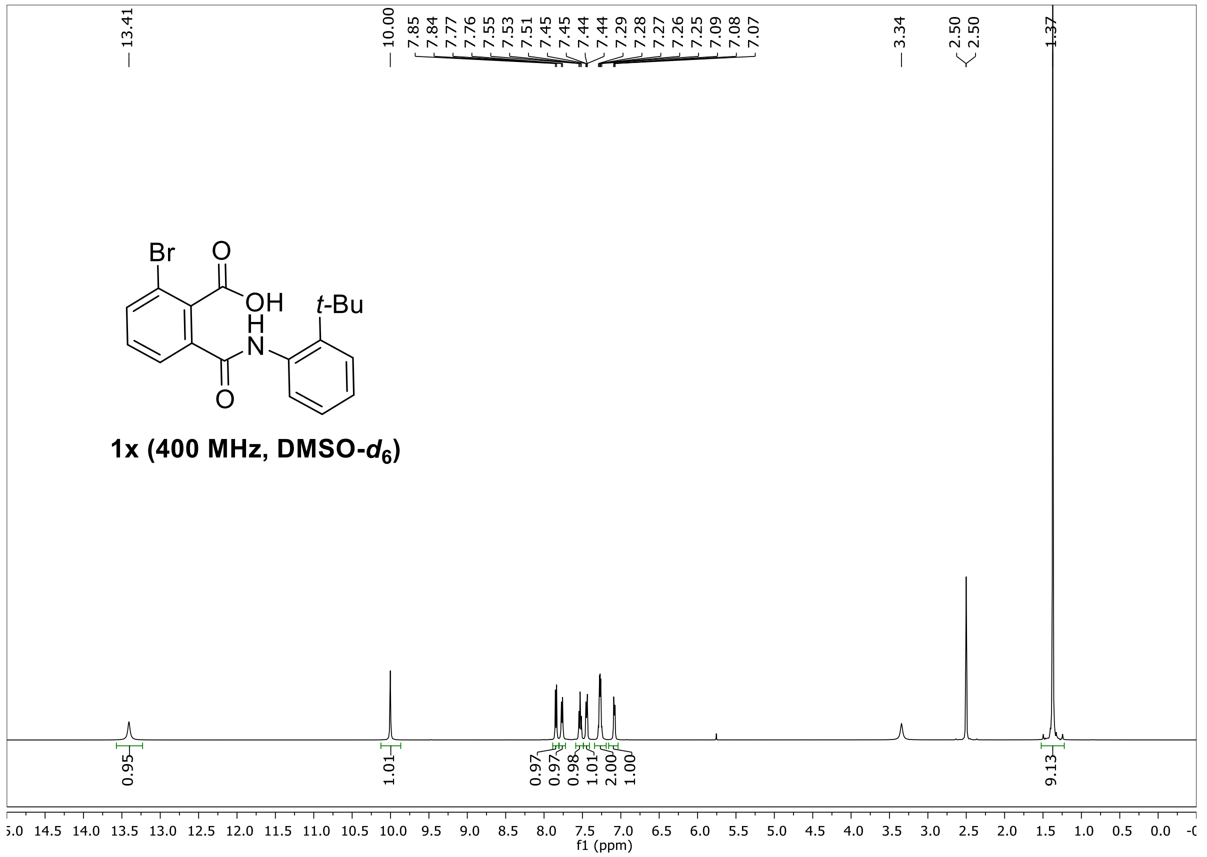

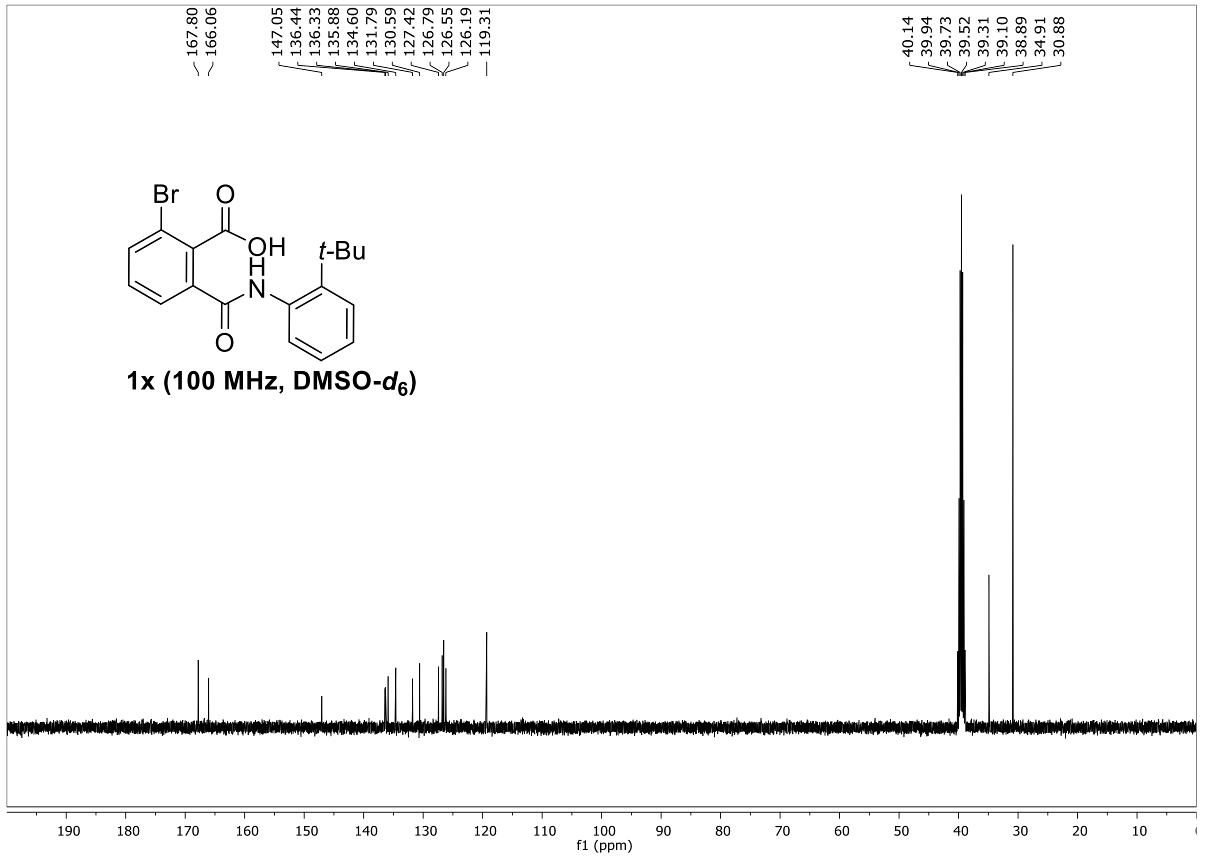

2-((2-(*tert*-Butyl)phenyl)carbamoyl)-6-nitrobenzoic acid (1y)

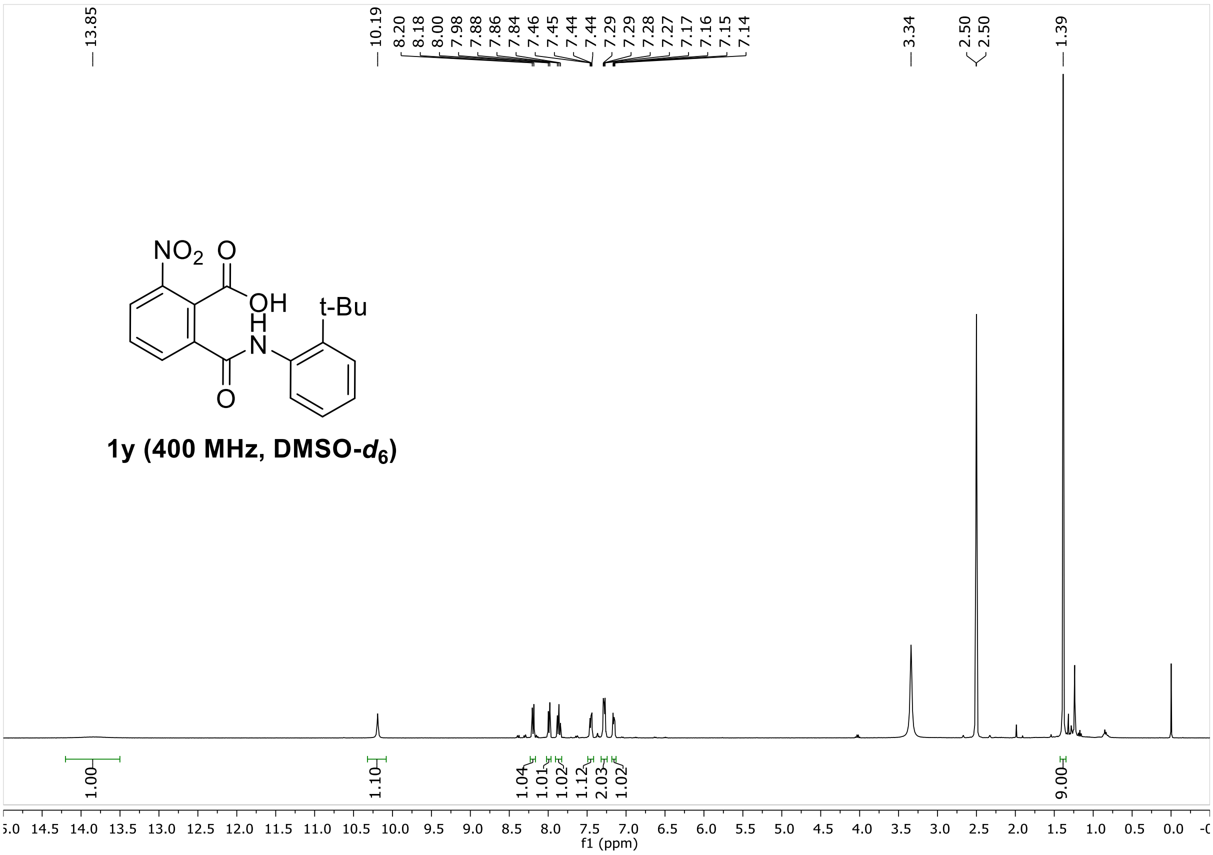

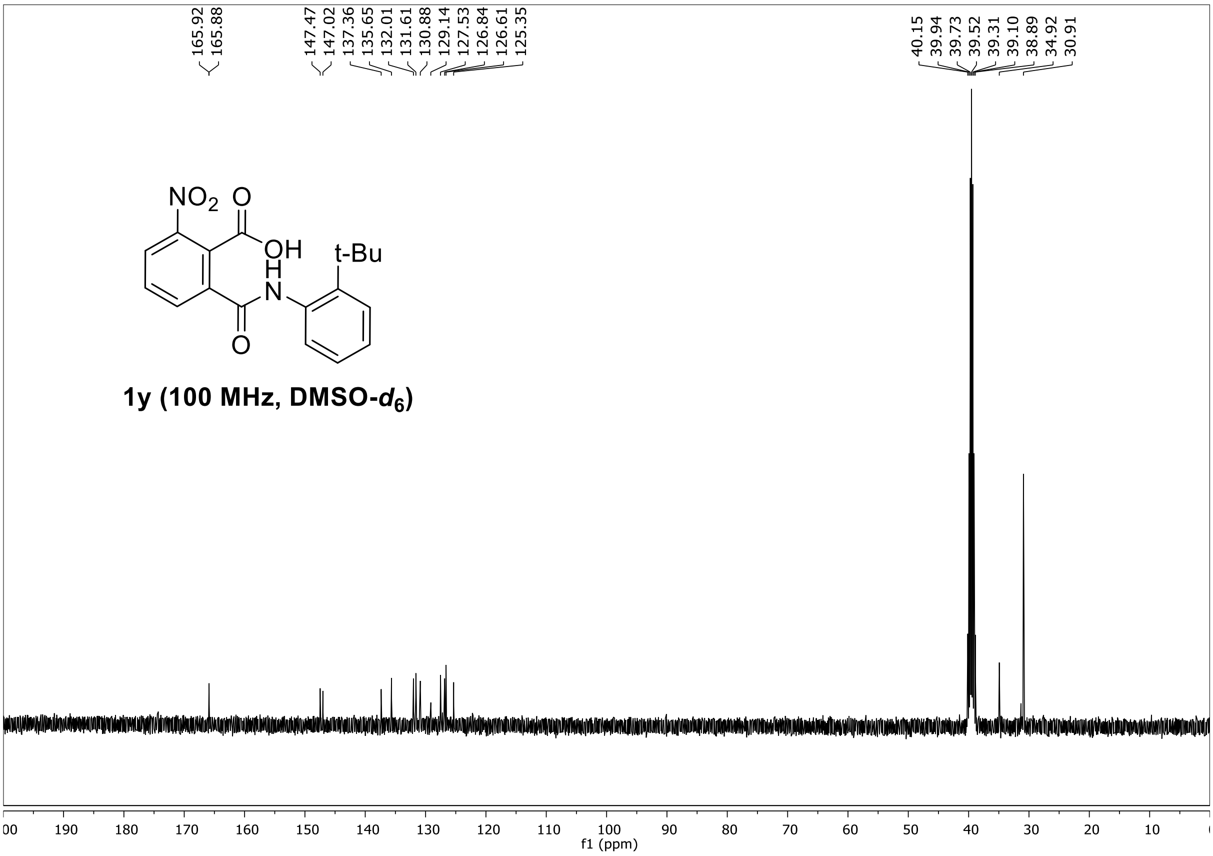

2-((2-(*tert*-Butyl)phenyl)carbamoyl)-1-naphthoic acid (1z)

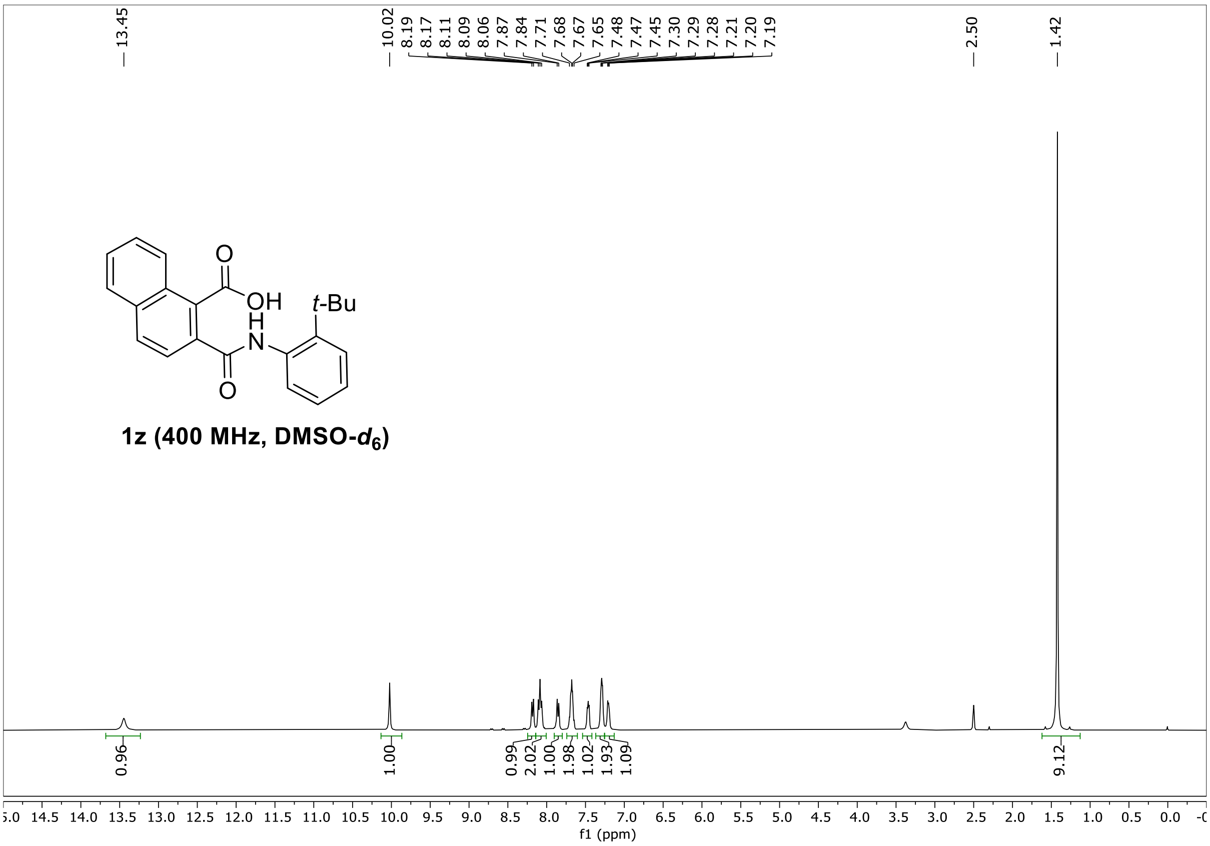

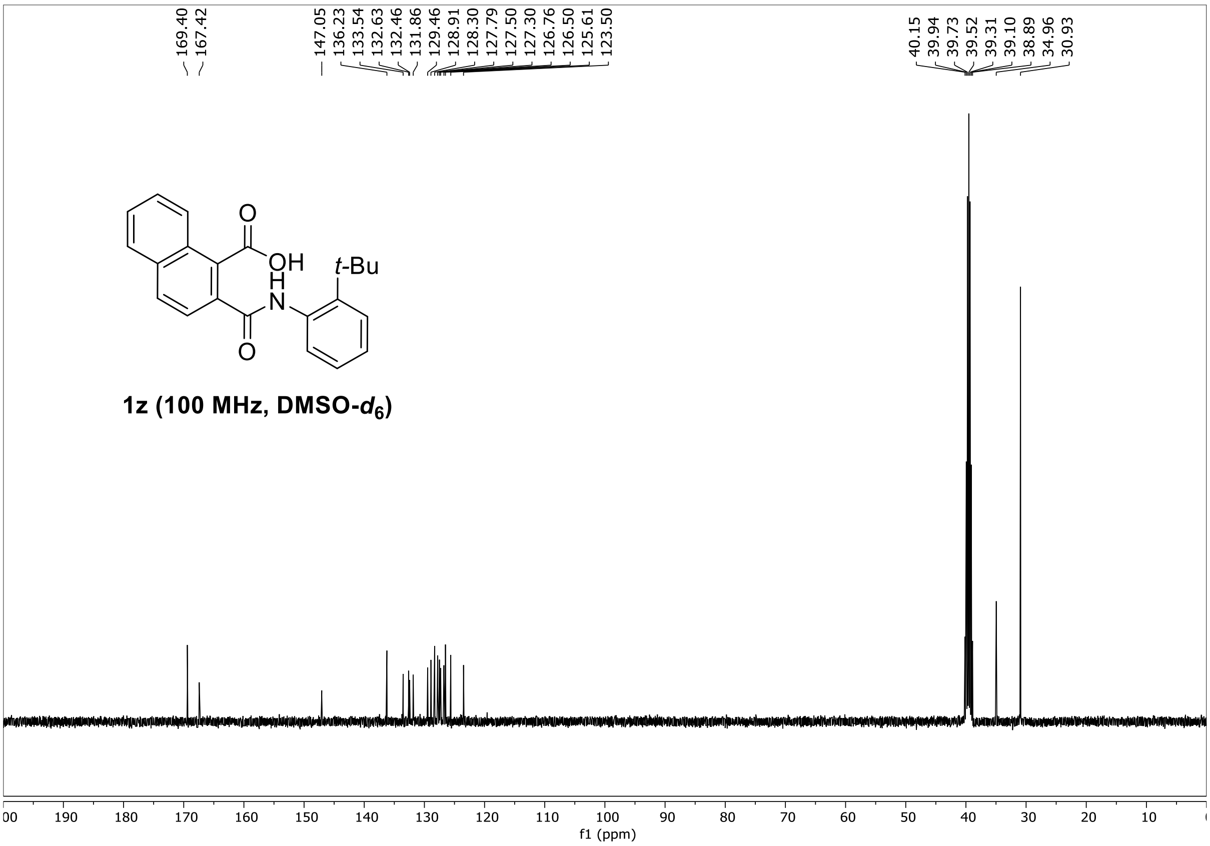

6-((2-(*tert*-Butyl)phenyl)carbamoyl)-2-methoxy-3-nitrobenzoic acid (1aa)

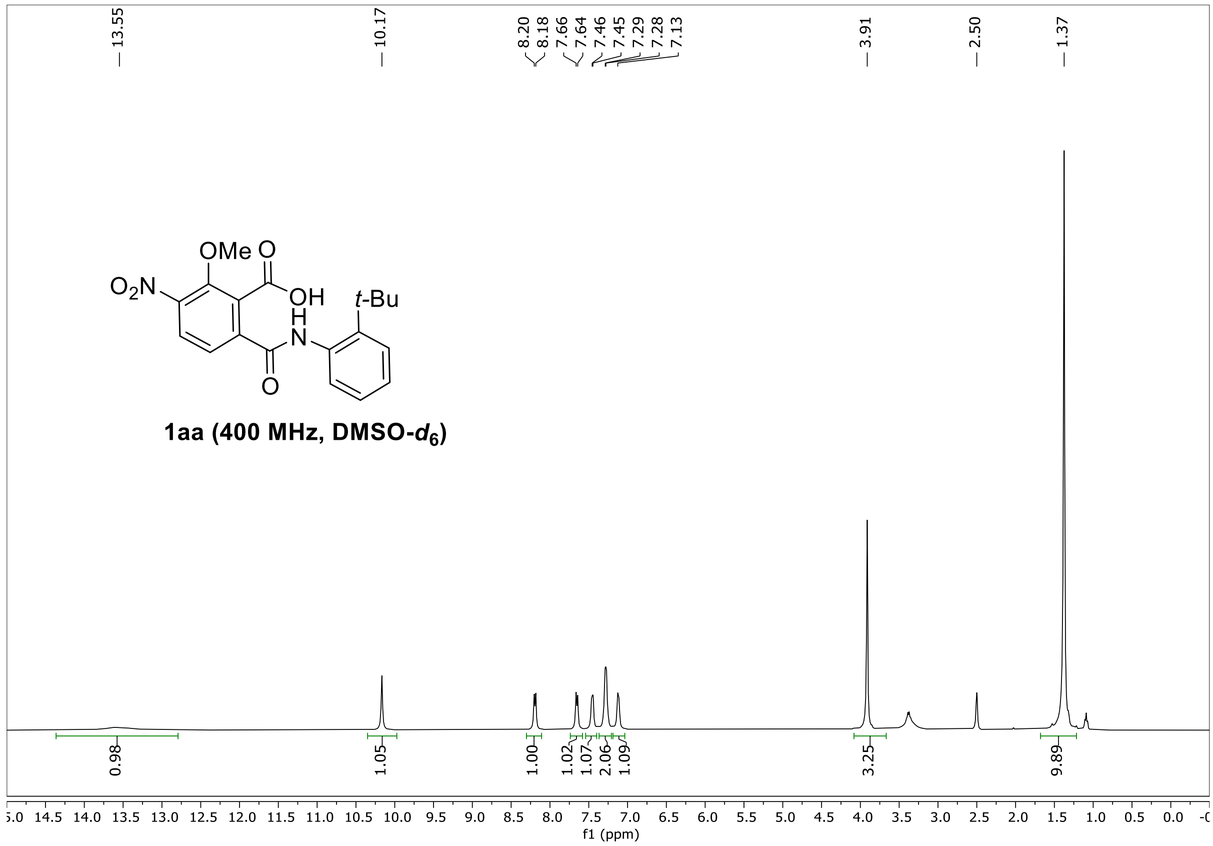

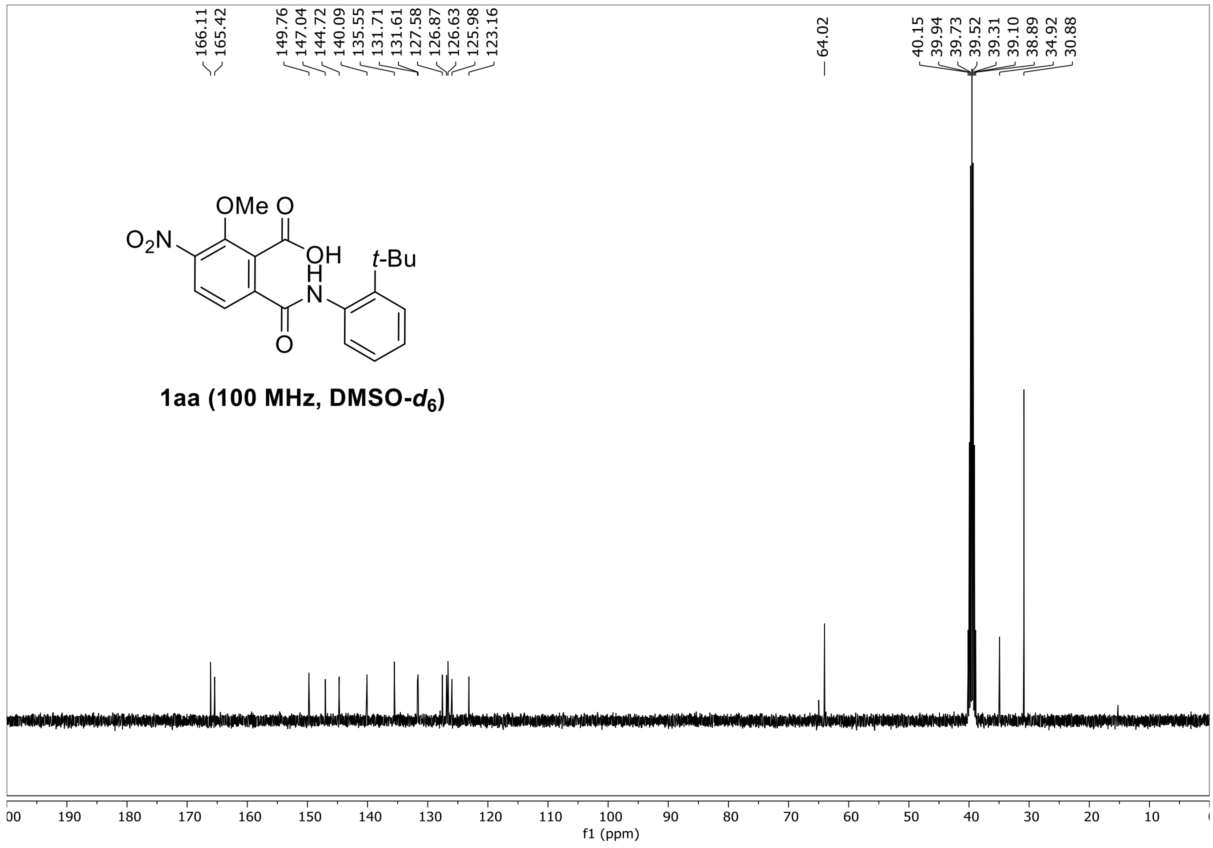

2-((2-(*tert*-Butyl)phenyl)carbamoyl)-6-methoxy-3-nitrobenzoic acid (1ab)

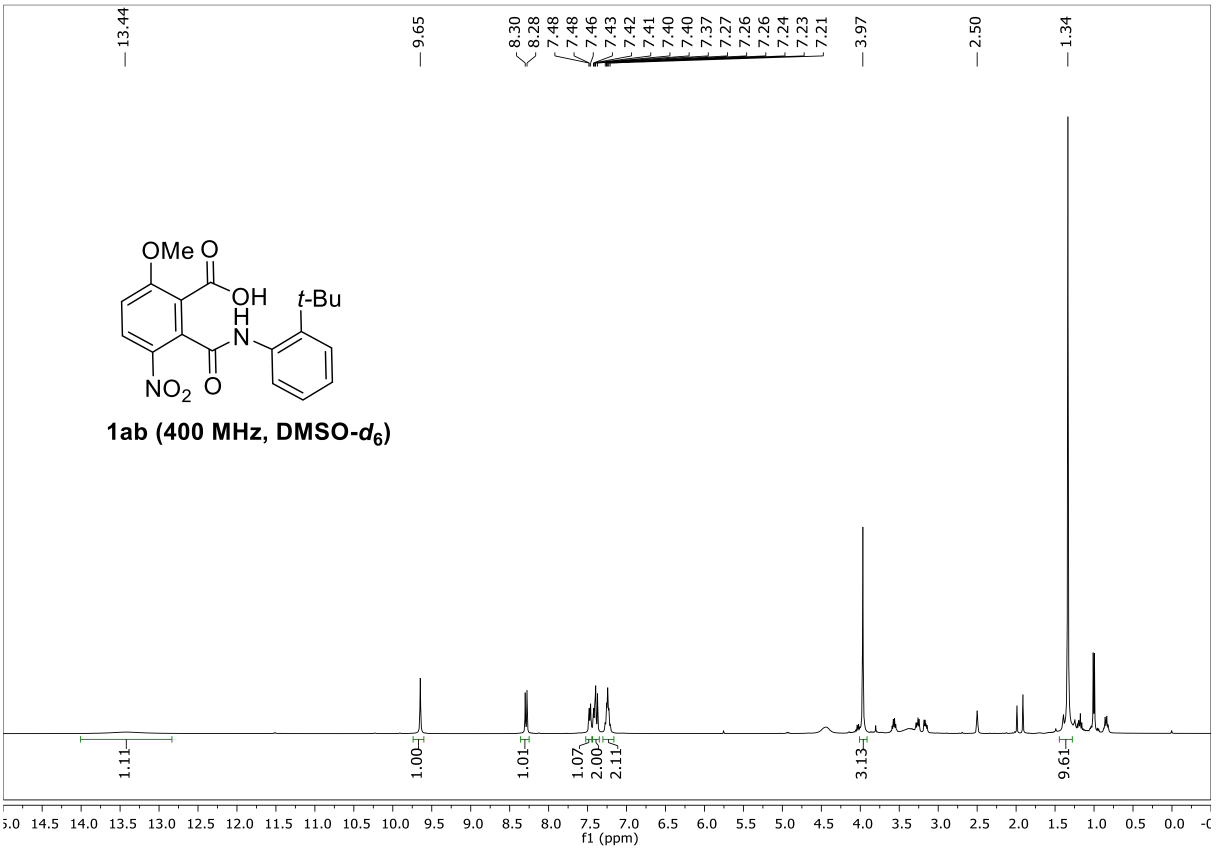

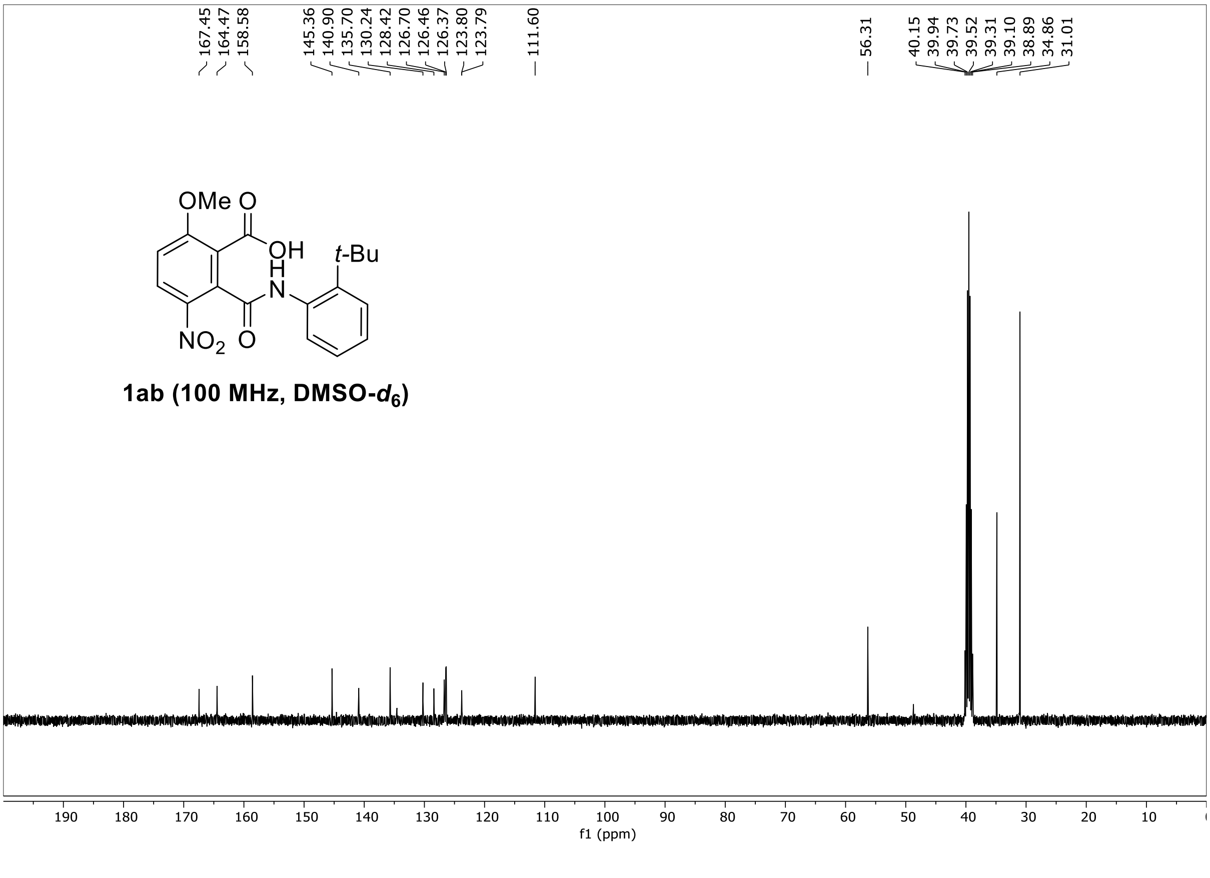

2-((2-(*tert*-Butyl)phenyl)carbamoyl)-4-phenyl-1-naphthoic acid (1ac)

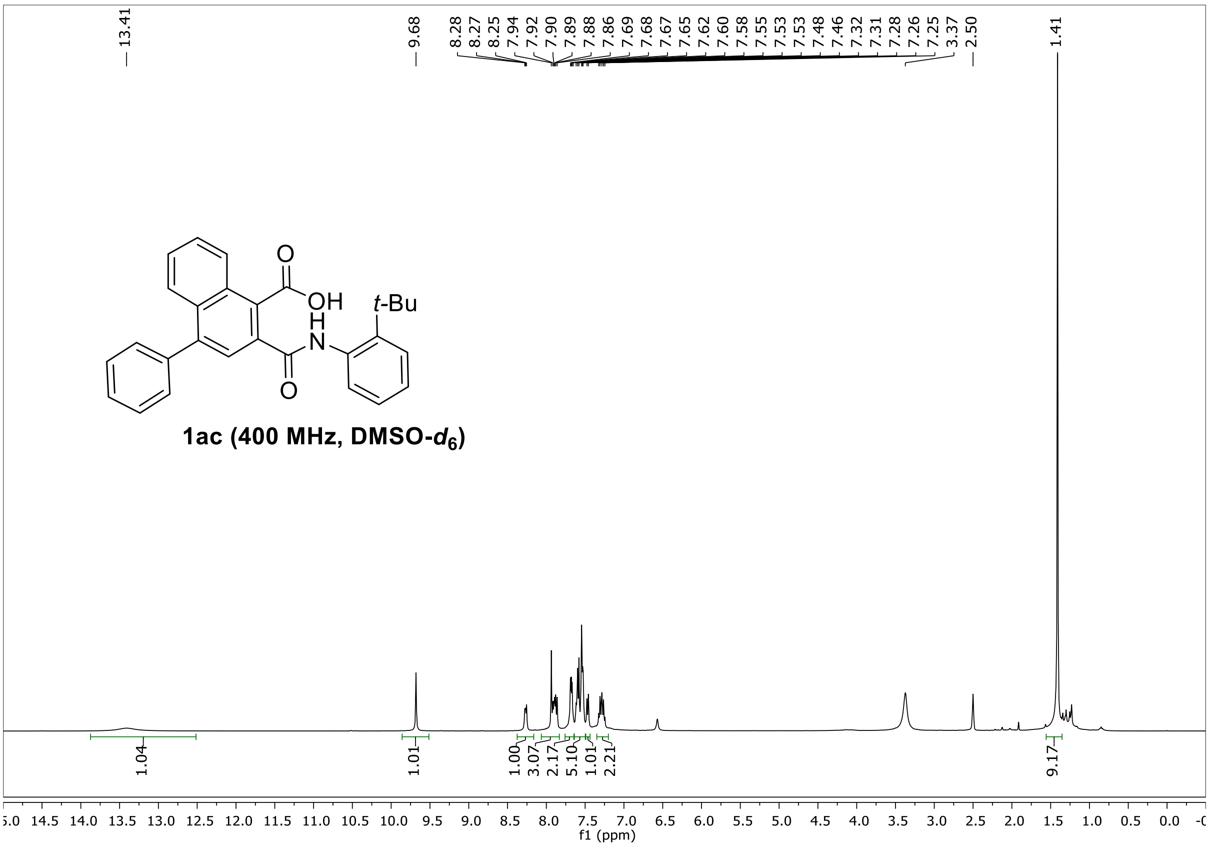

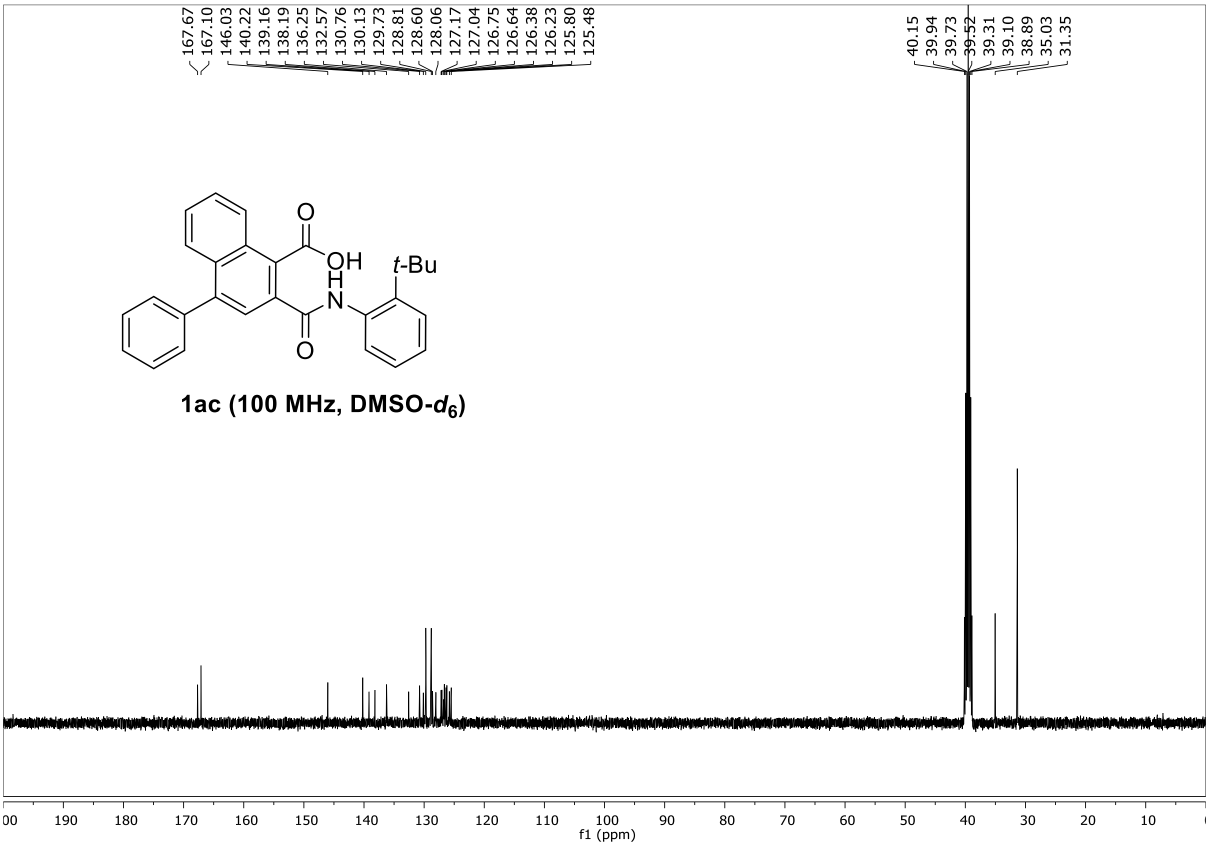

3-((2-(*tert*-Butyl)phenyl)carbamoyl)picolinic acid (1ad)

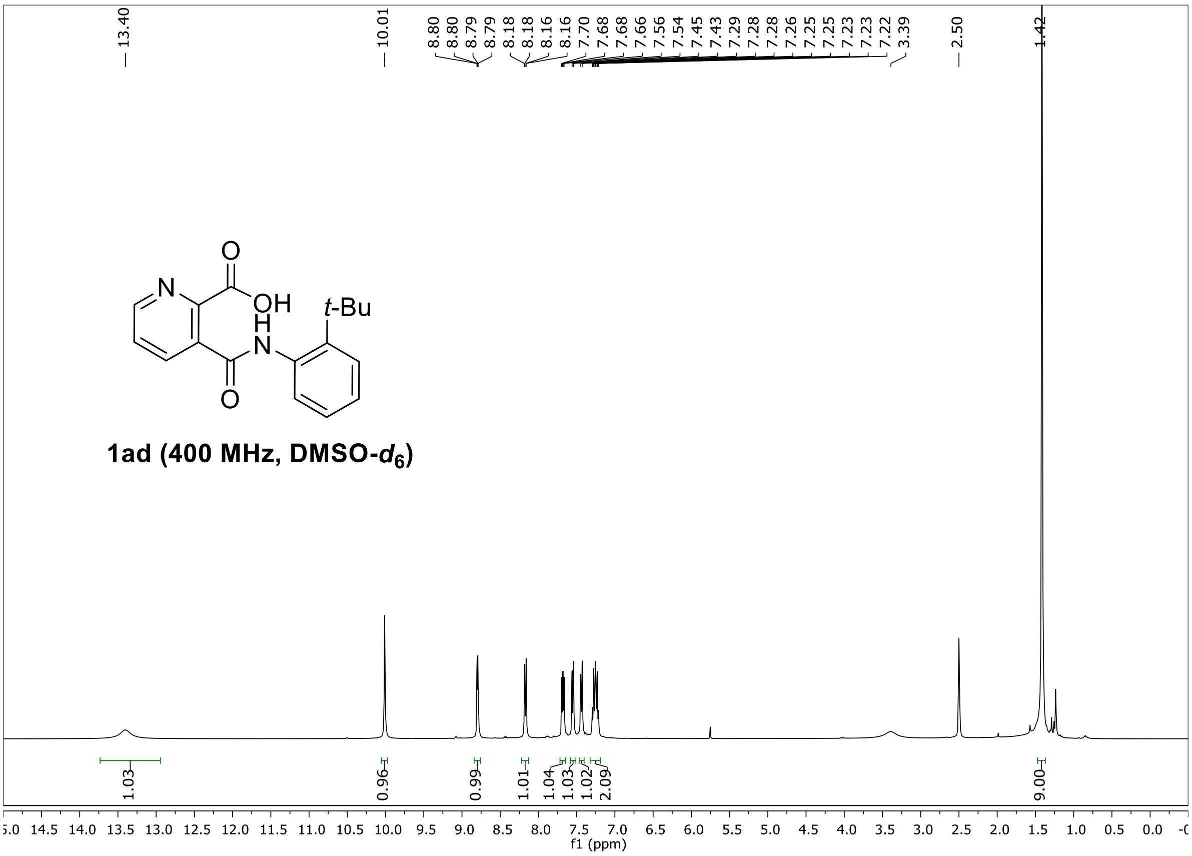

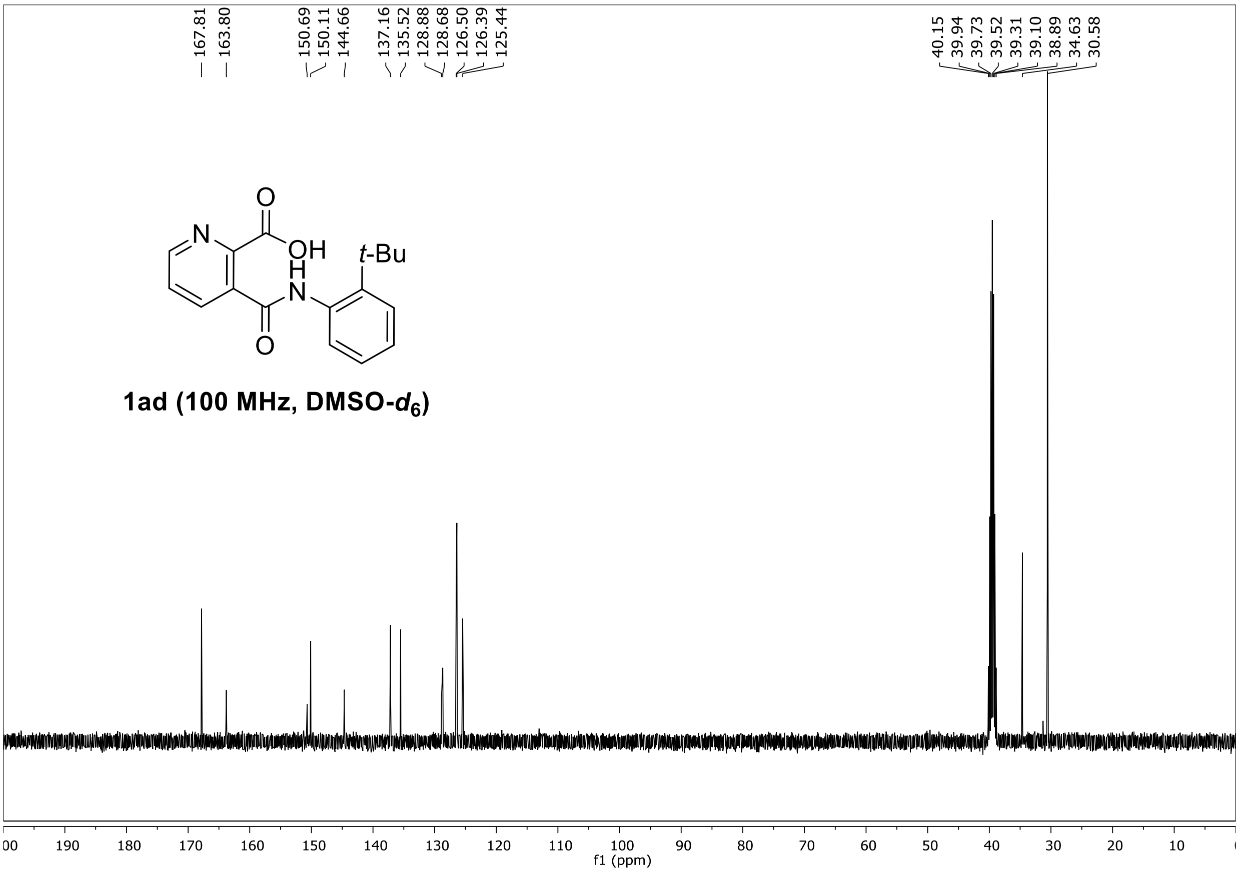

(*Z*)-4-((2-(*tert*-Butyl)phenyl)amino)-2-methyl-4-oxobut-2-enoic acid (8a)

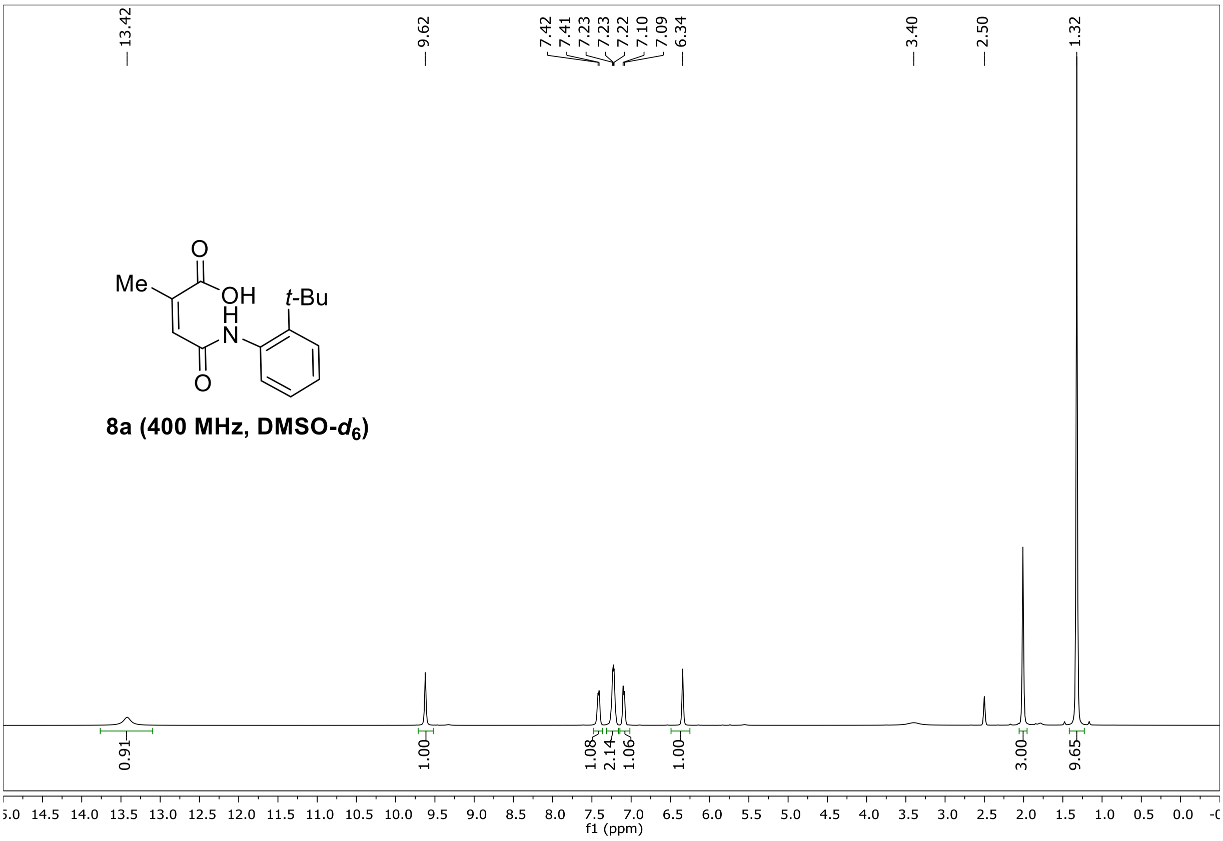

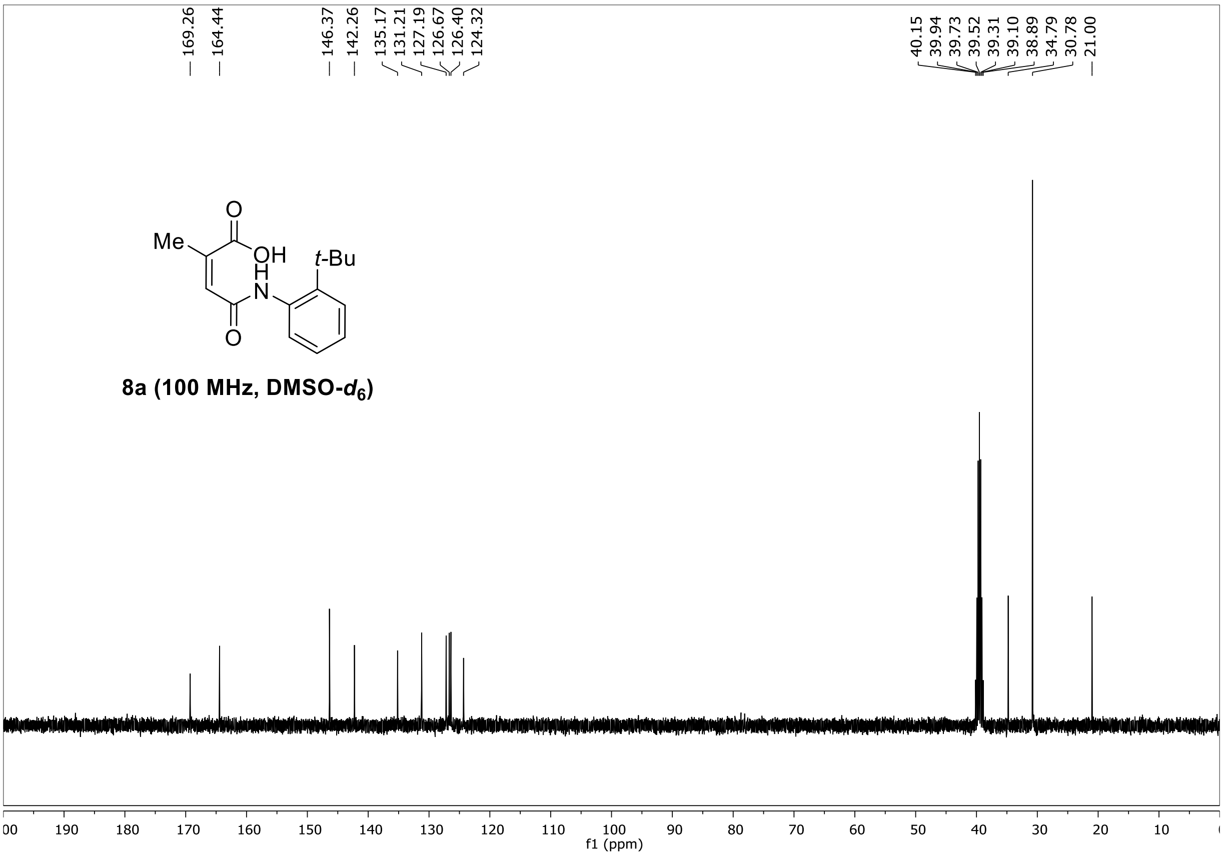

(*Z*)-4-((4-Bromo-2-(*tert*-butyl)phenyl)amino)-2-methyl-4-oxobut-2-enoicacid (8b)

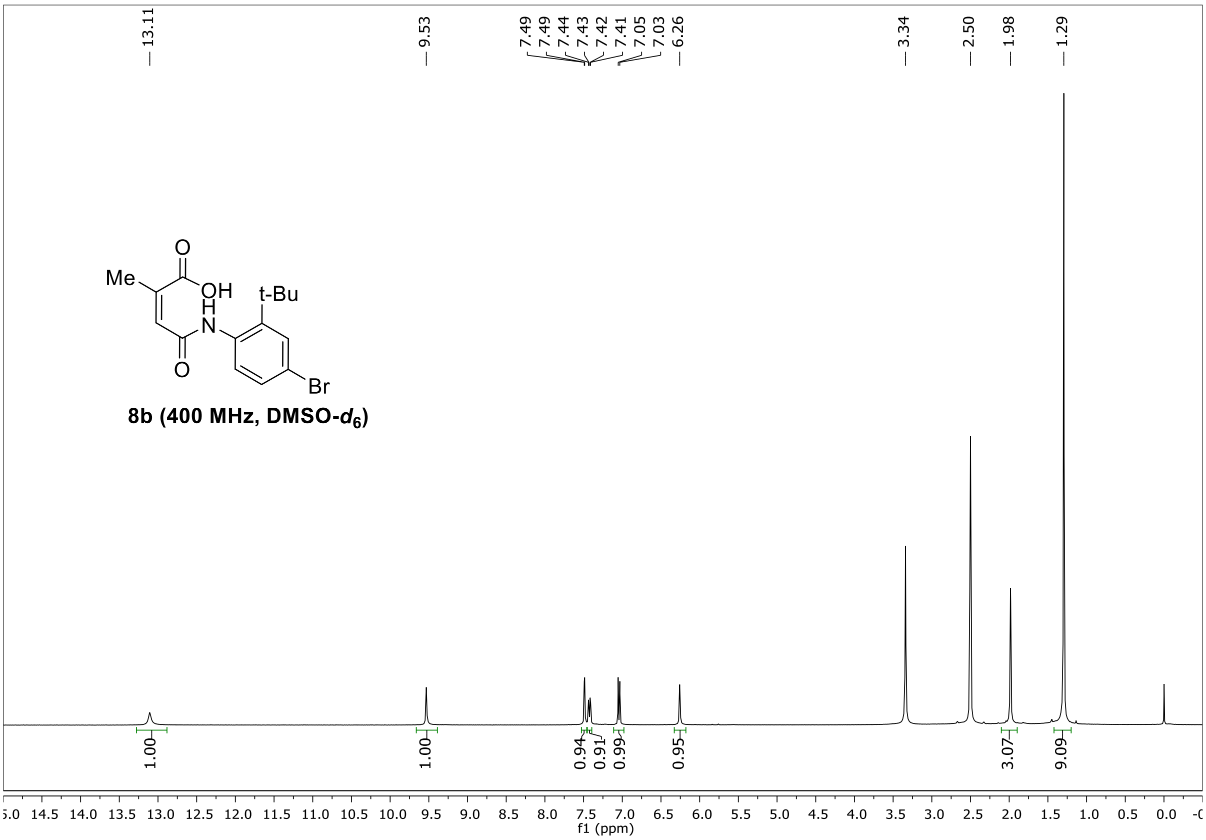

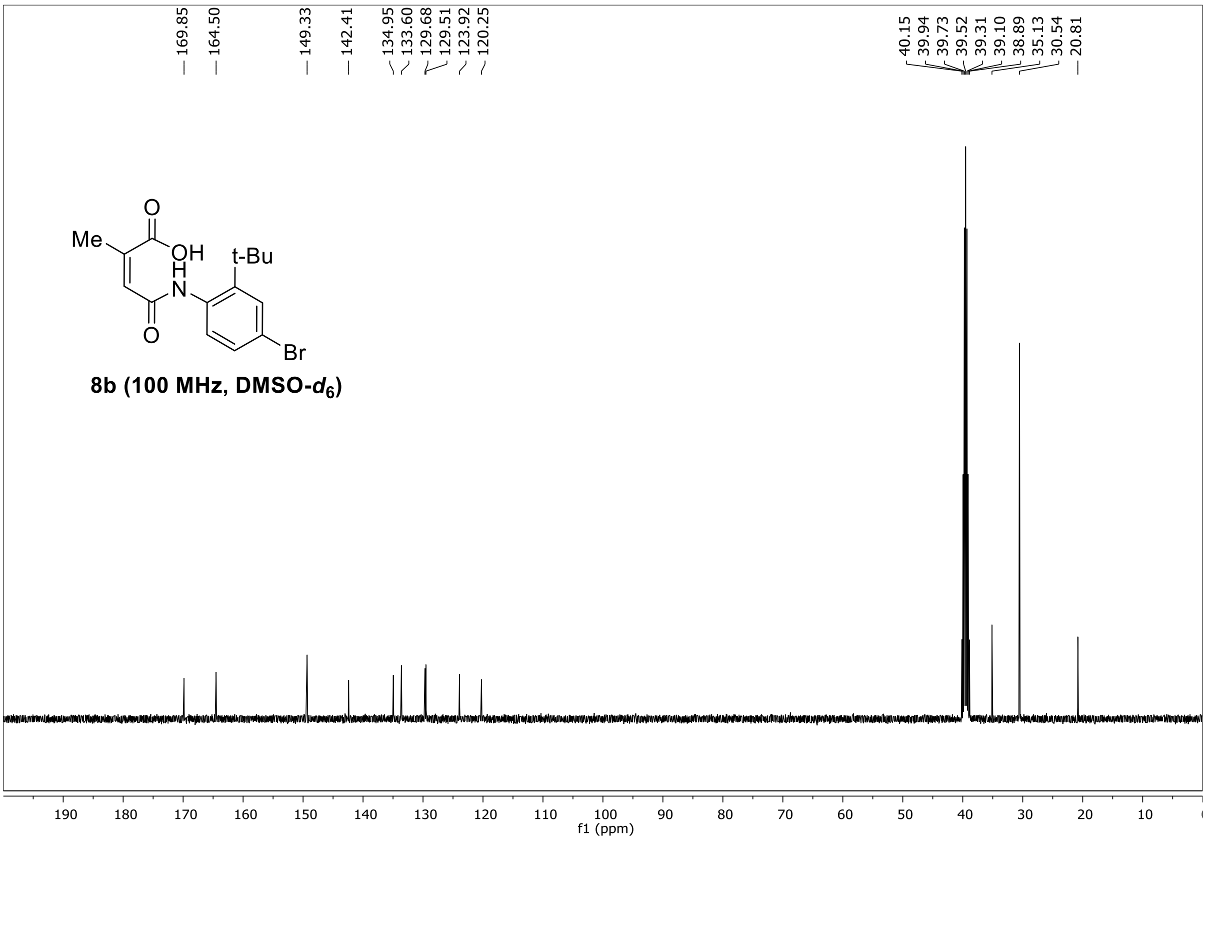

(*Z*)-4-((2-(*tert*-Butyl)-4-iodophenyl)amino)-2-methyl-4-oxobut-2-enoic acid (8c)

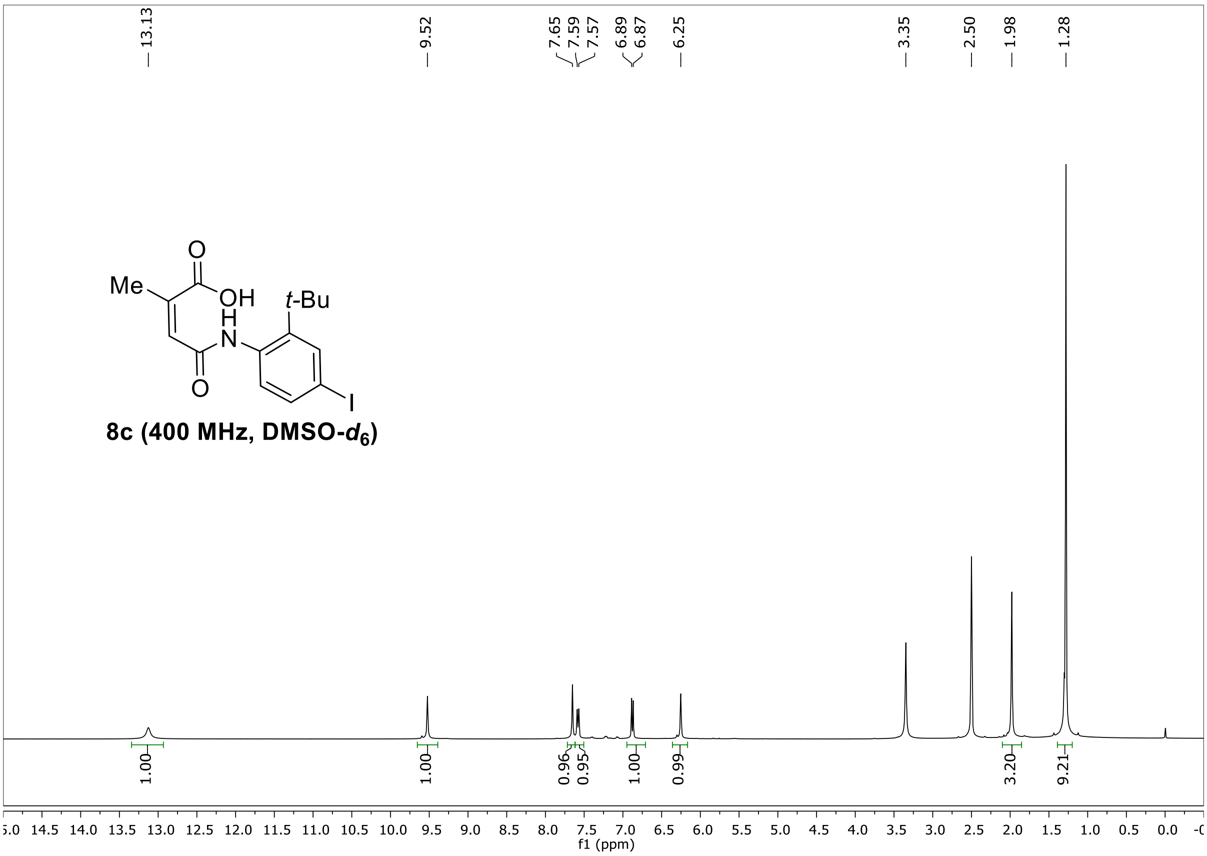

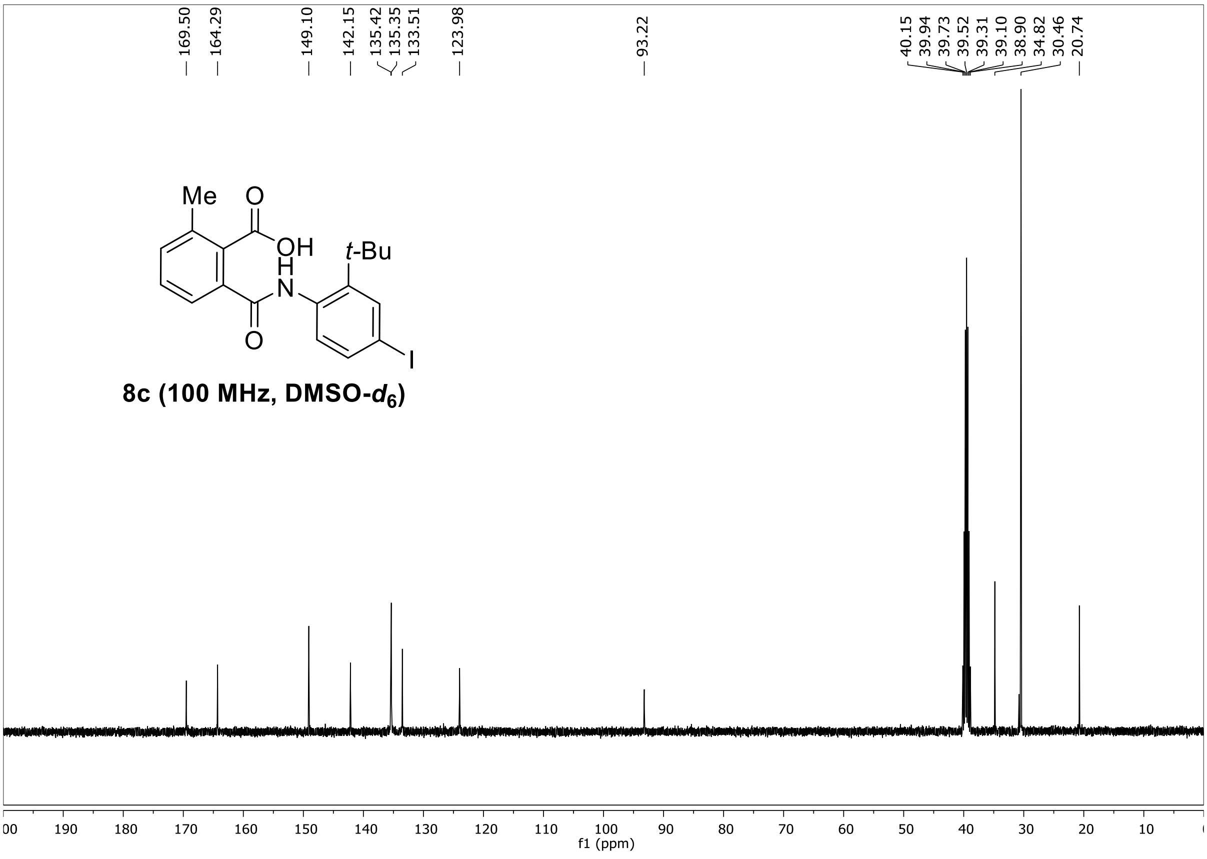

(*Z*)-4-((2-(*tert*-Butyl)-4-(ethoxycarbonyl)phenyl)amino)-2-methyl-4-oxobut-2-enoic acid (8d)

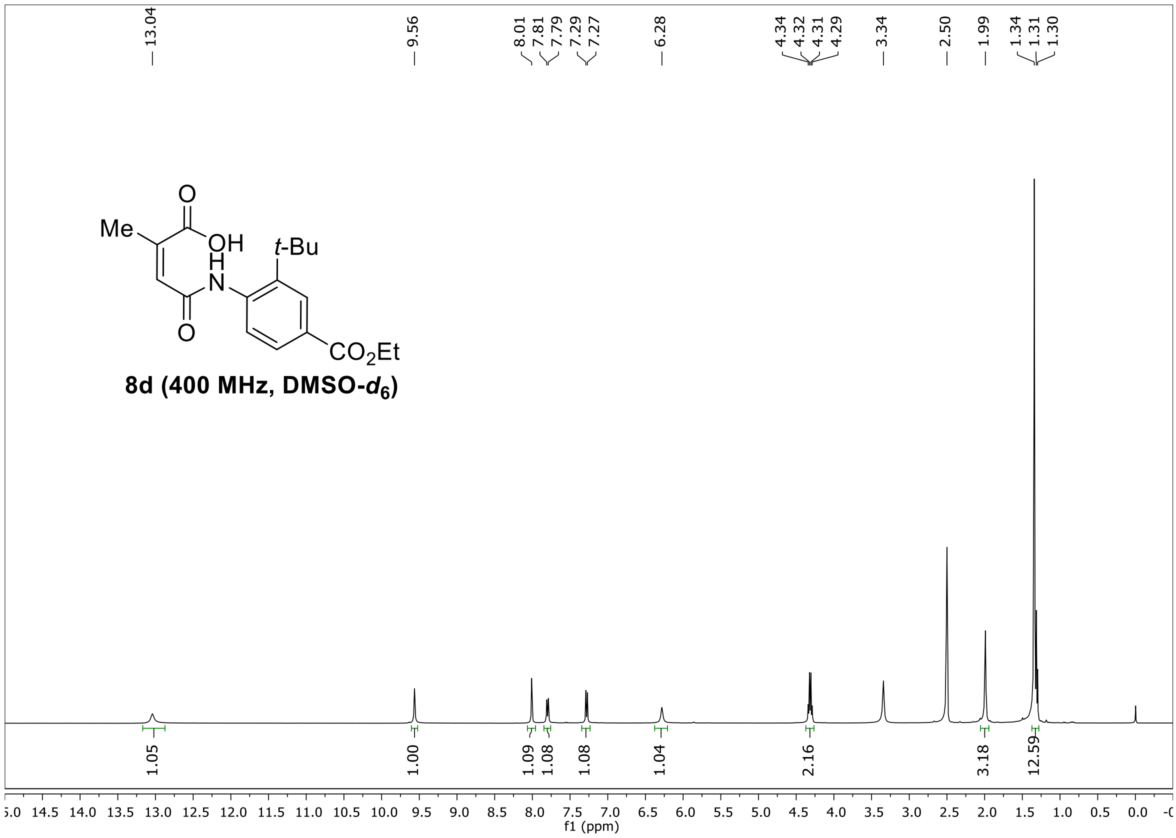

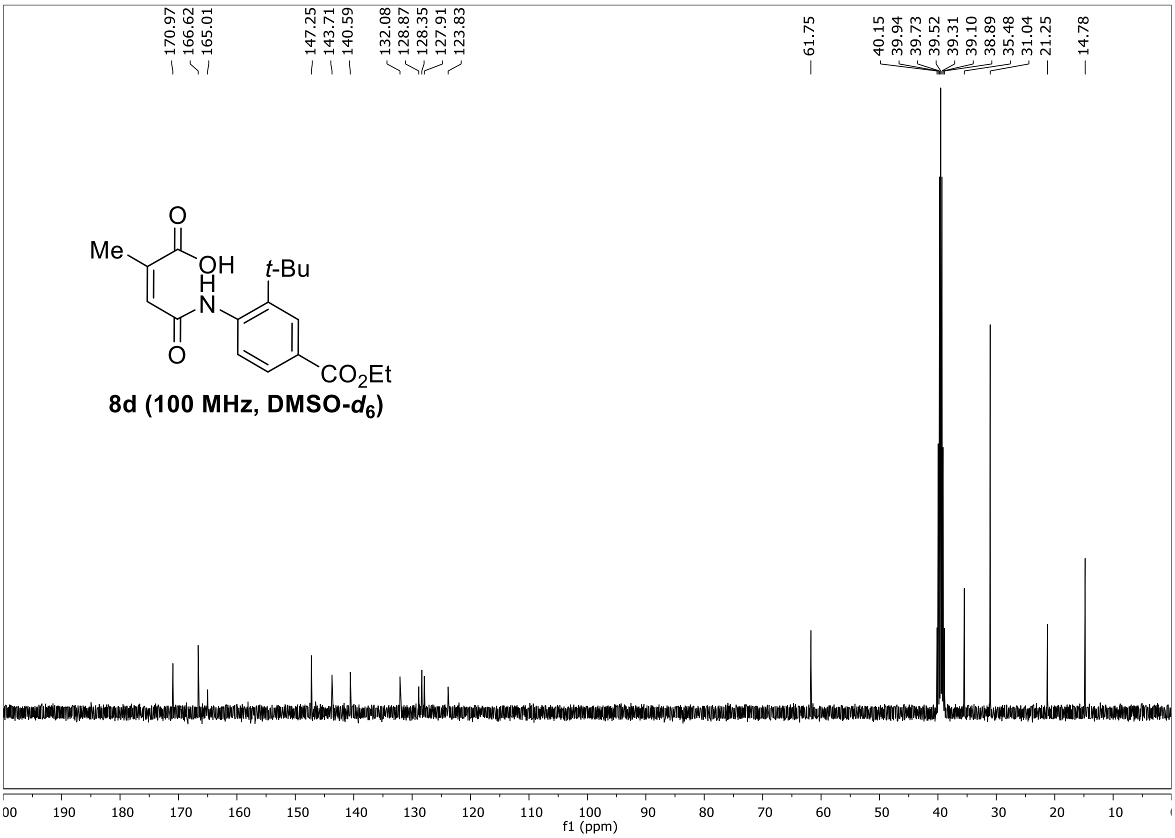

(*Z*)-2-Methyl-4-((4-methyl-2-(2-phenylpropan-2-yl)phenyl)amino)-4-oxobut-2-enoic acid (8e)

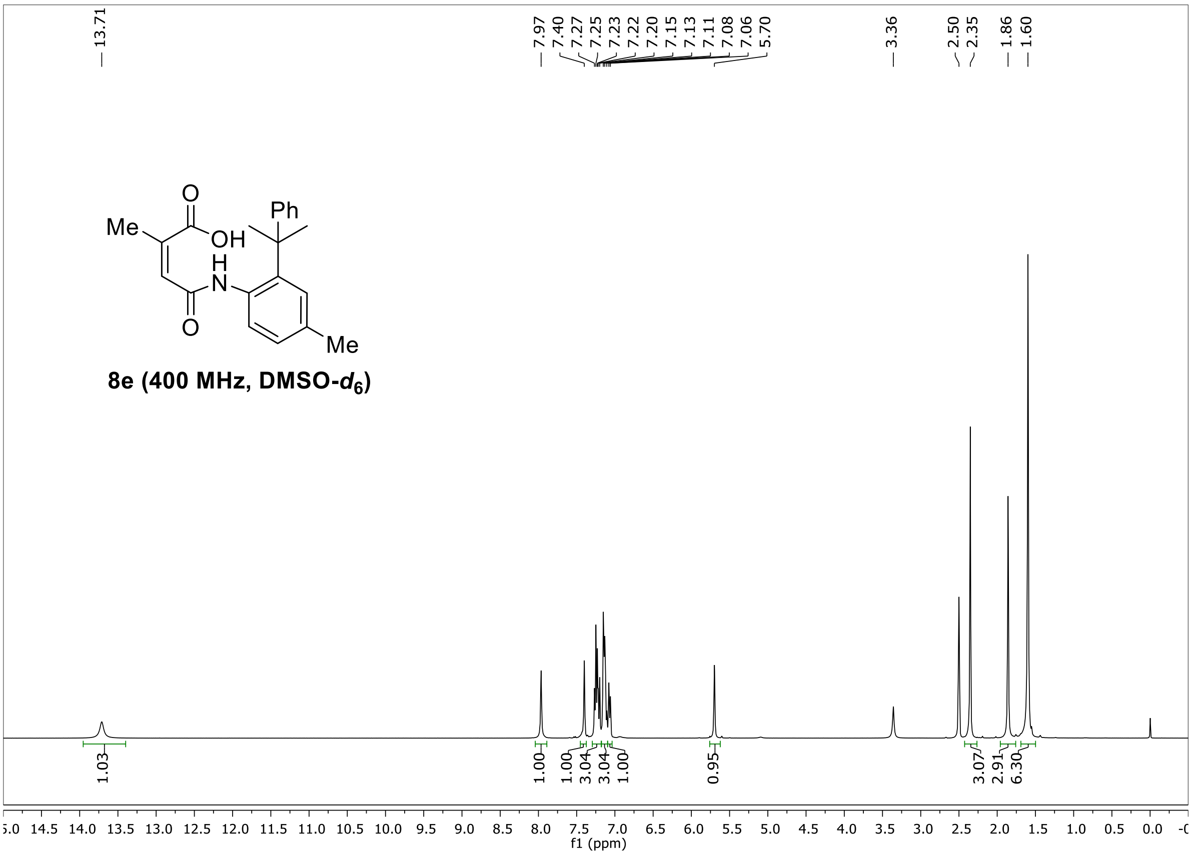

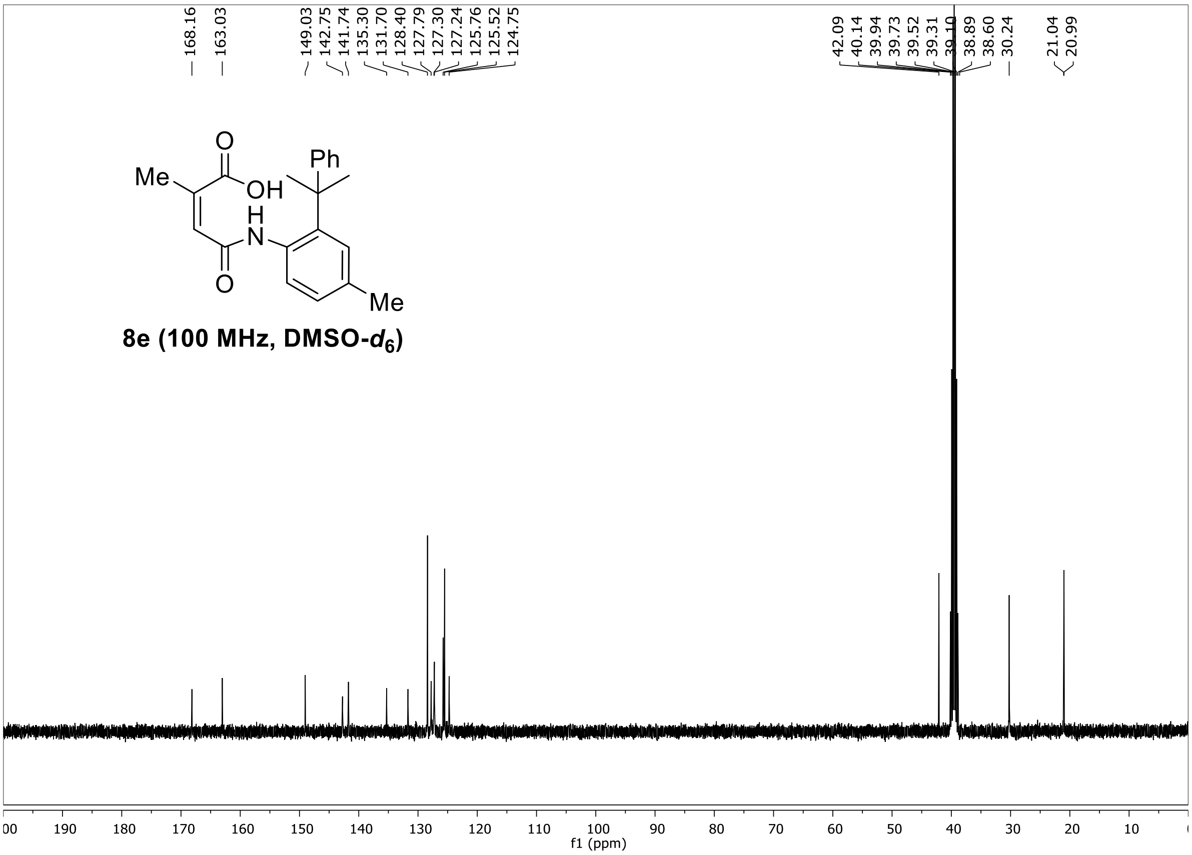

(*E*)-3-Bromo-4-((2-(*tert*-butyl)phenyl)amino)-2-methyl-4-oxobut-2-enoic acid (8f)

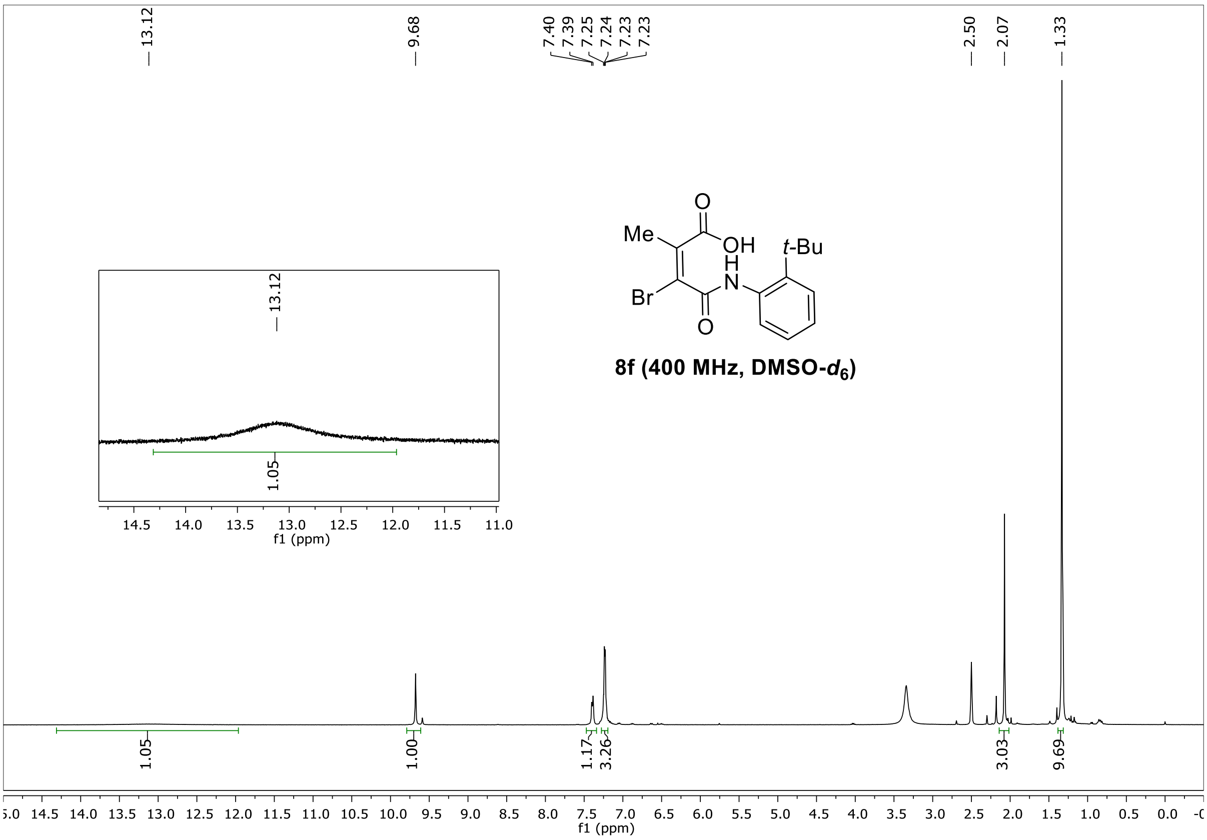

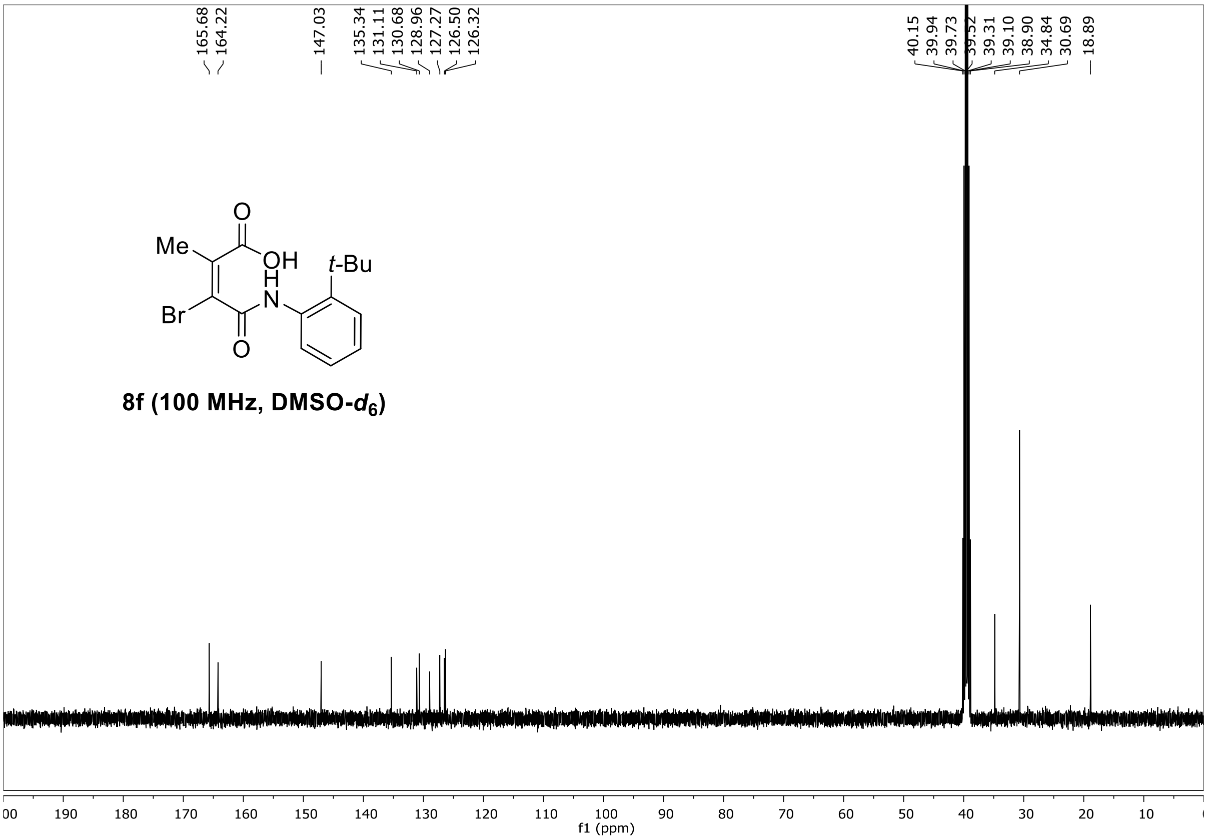

3.2 ¹H and ¹³C NMR Spectra of *N*-Aryl Phthalimide/Maleimide Derivatives

(*P*)-2-(2-(*tert*-Butyl)phenyl)-4-methylisoindoline-1,3-dione (2a)

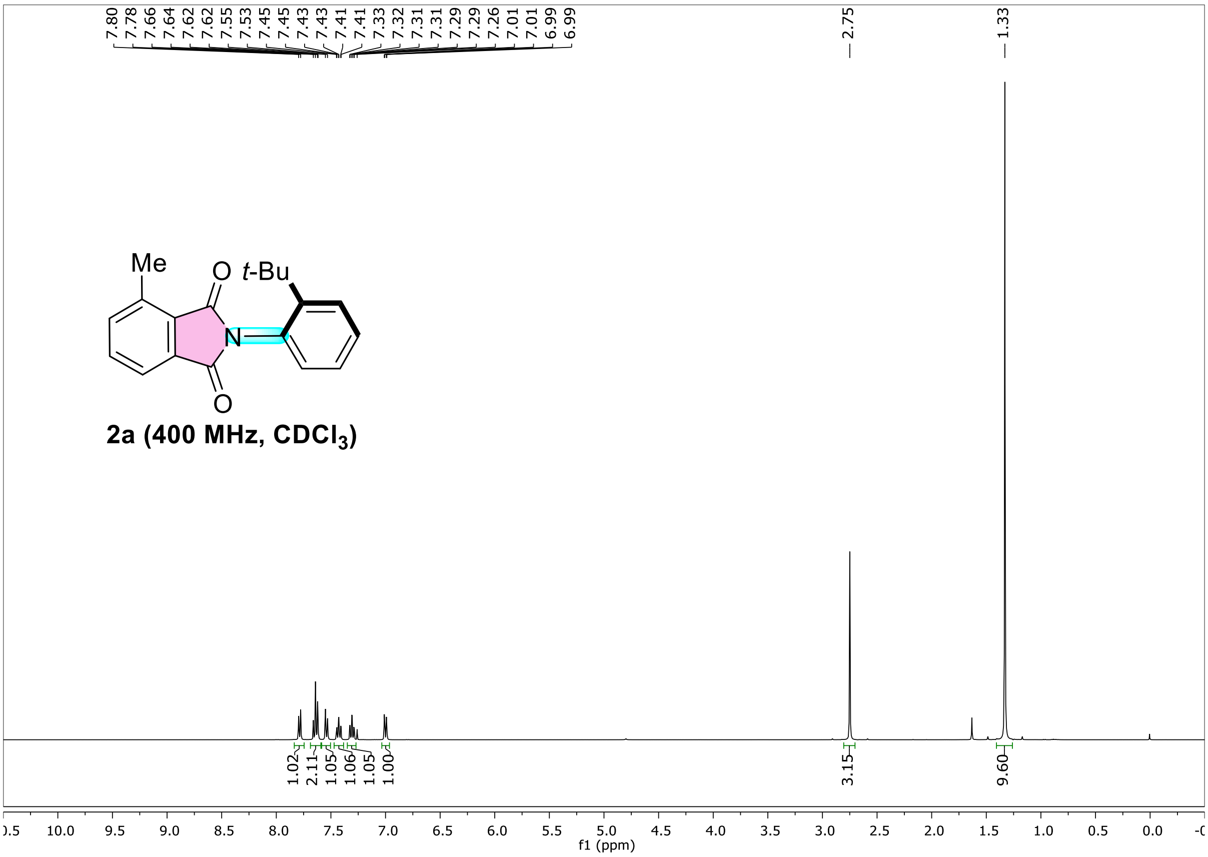

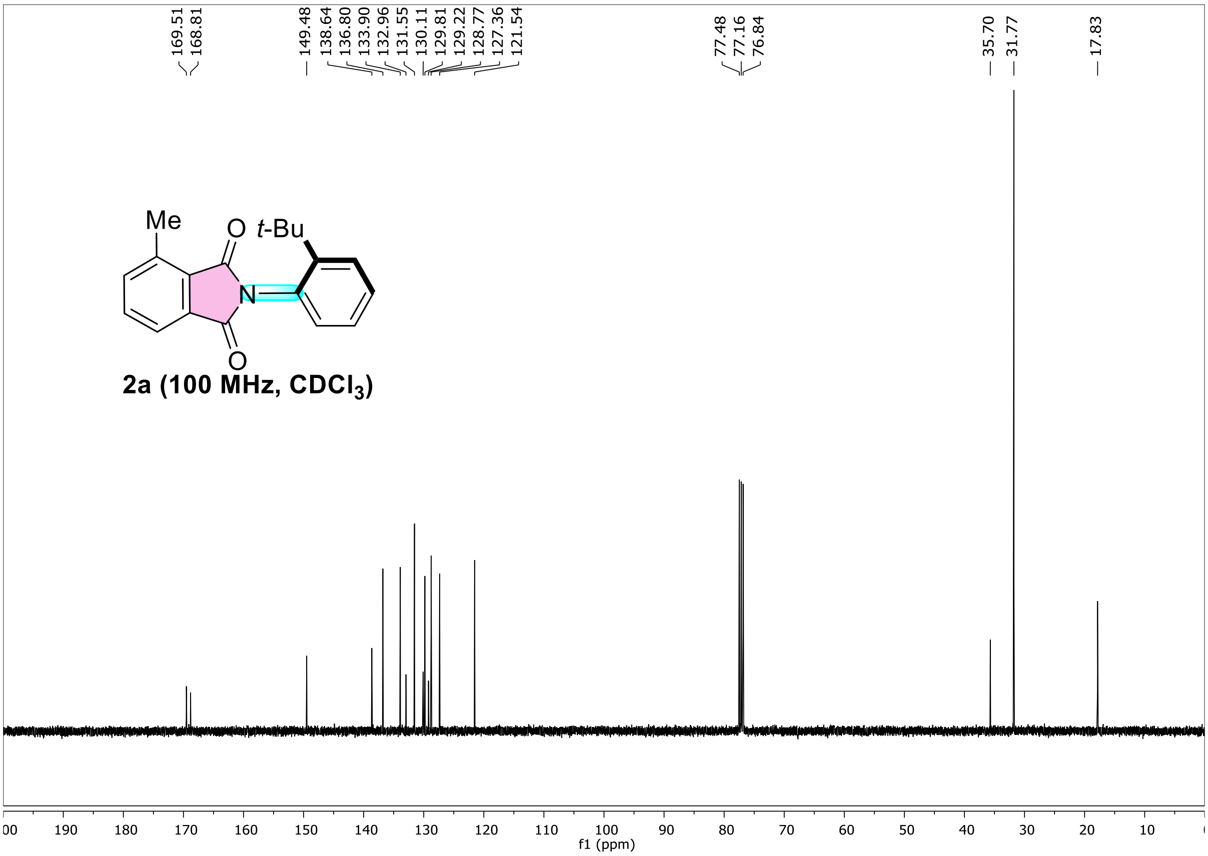

(*P*)-2-(2-(*tert*-Butyl)-4-methoxyphenyl)-4-methylisoindoline-1,3-dione (2b)

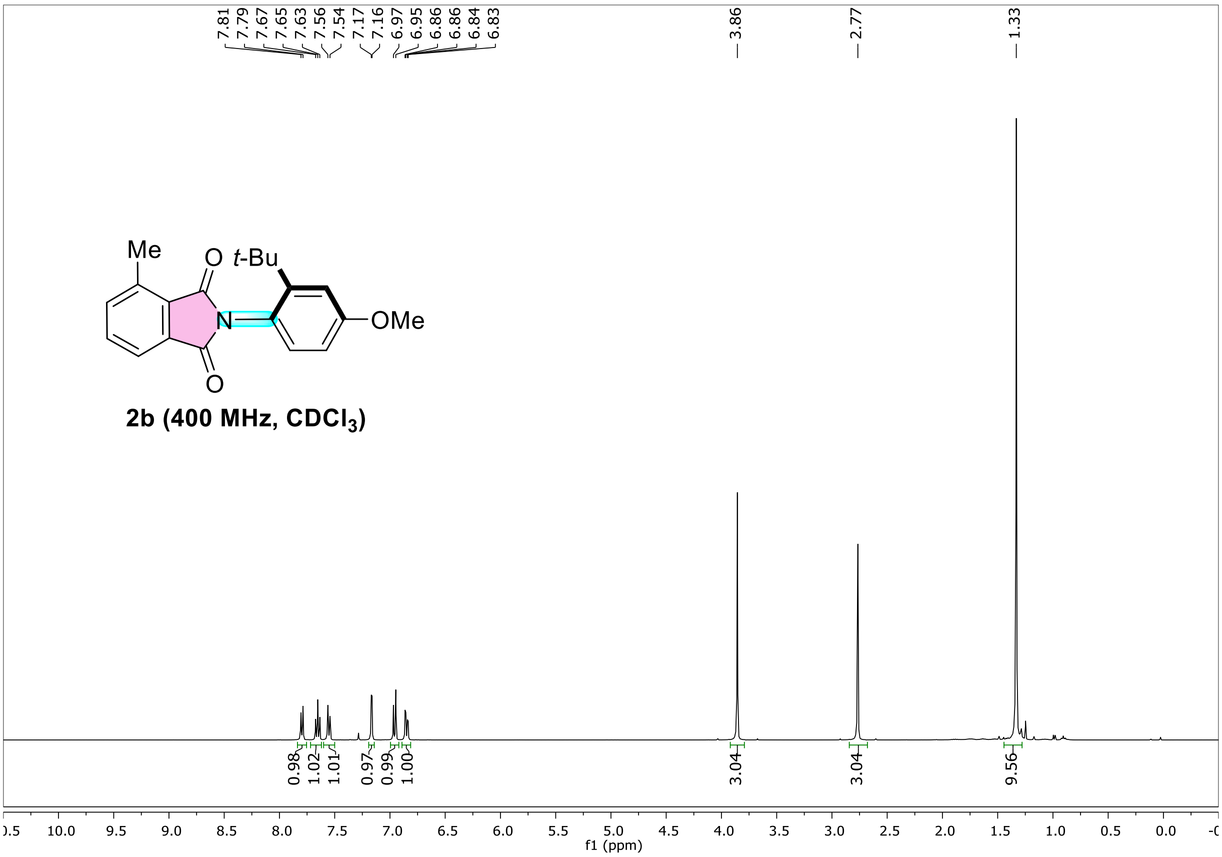

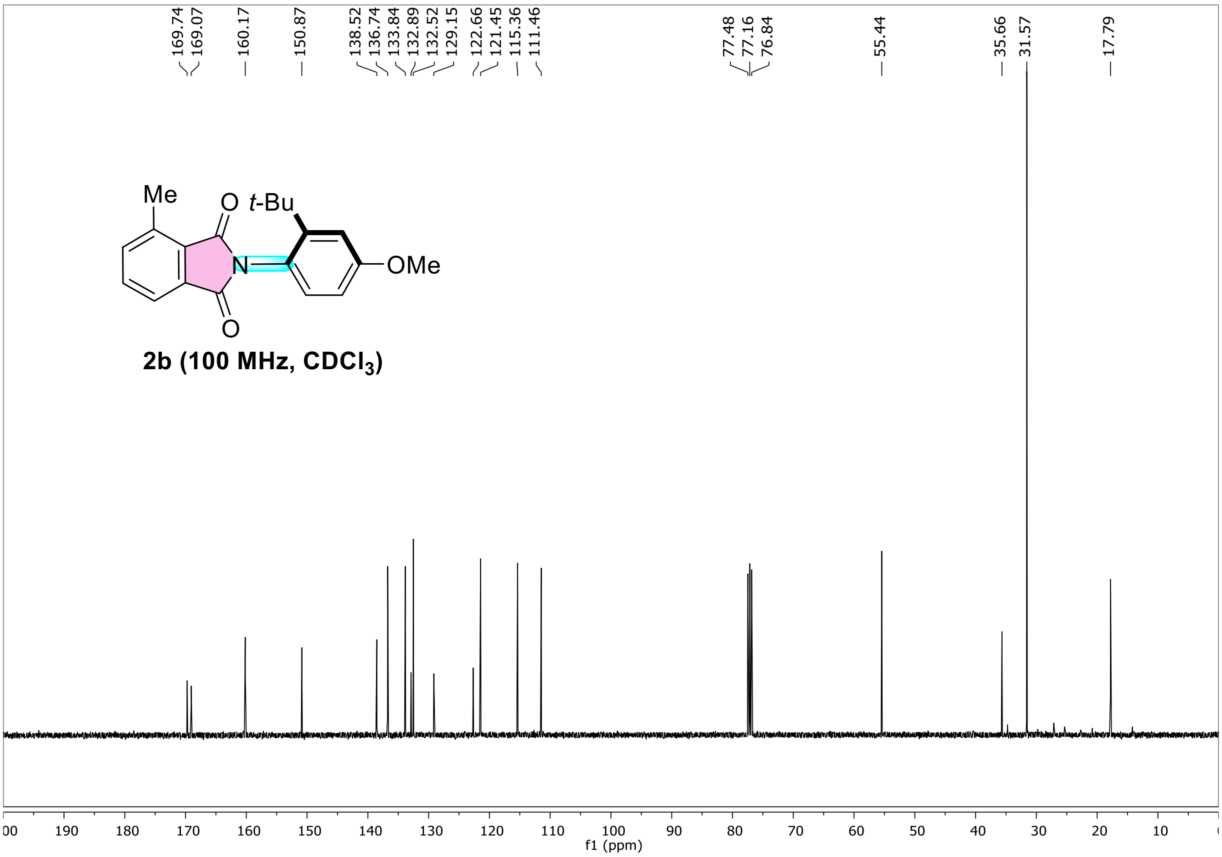

(*P*)-2-(4-(Benzyloxy)-2-(*tert*-butyl)phenyl)-4-methylisoindoline-1,3-dione (2c)

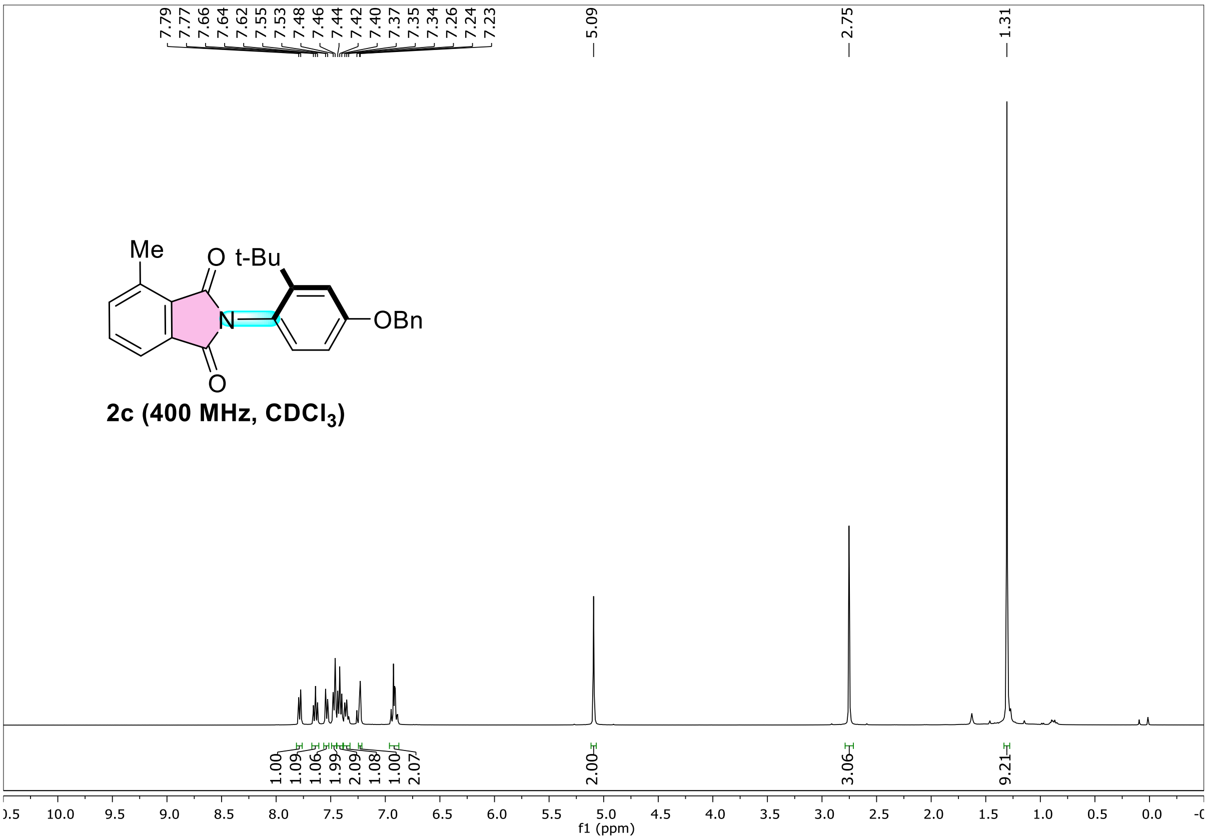

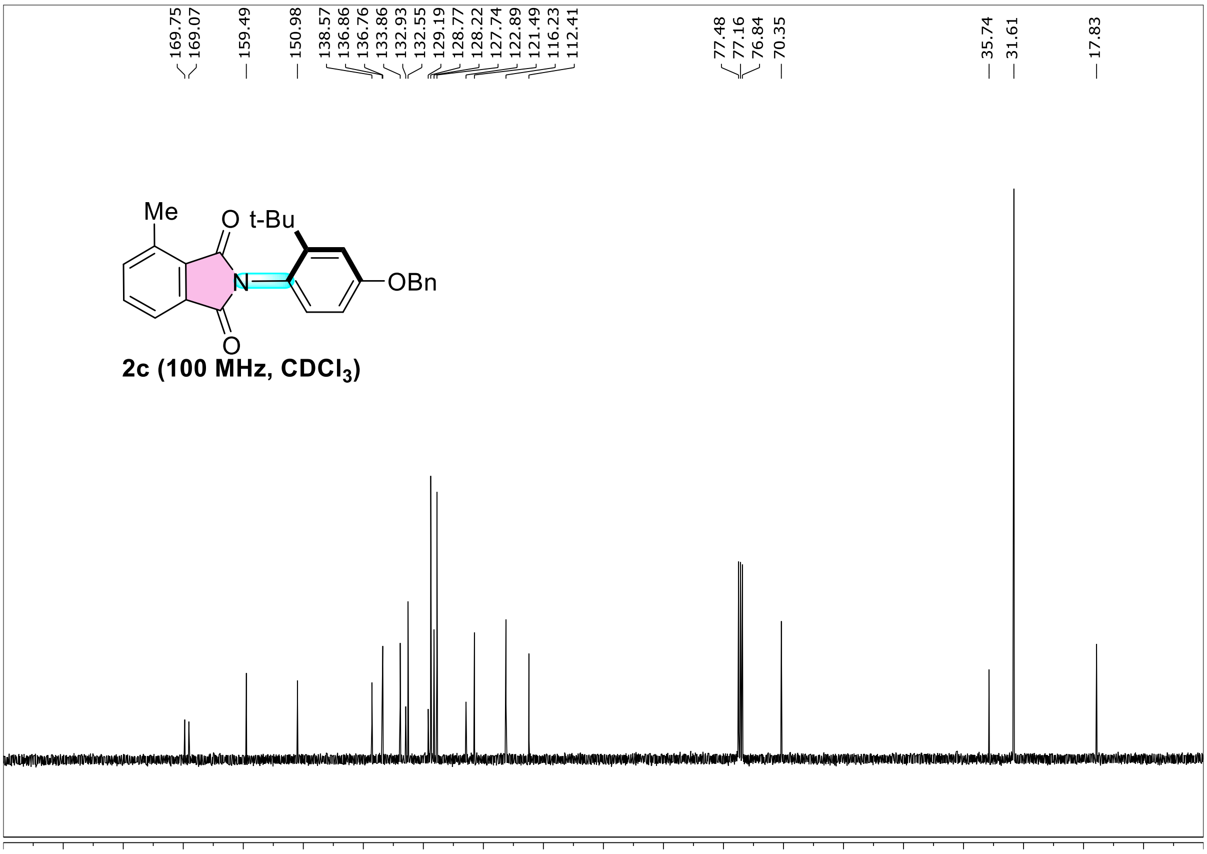

(*P*)-2-(4-Bromo-2-(*tert*-butyl)phenyl)-4-methylisoindoline-1,3-dione (2d)

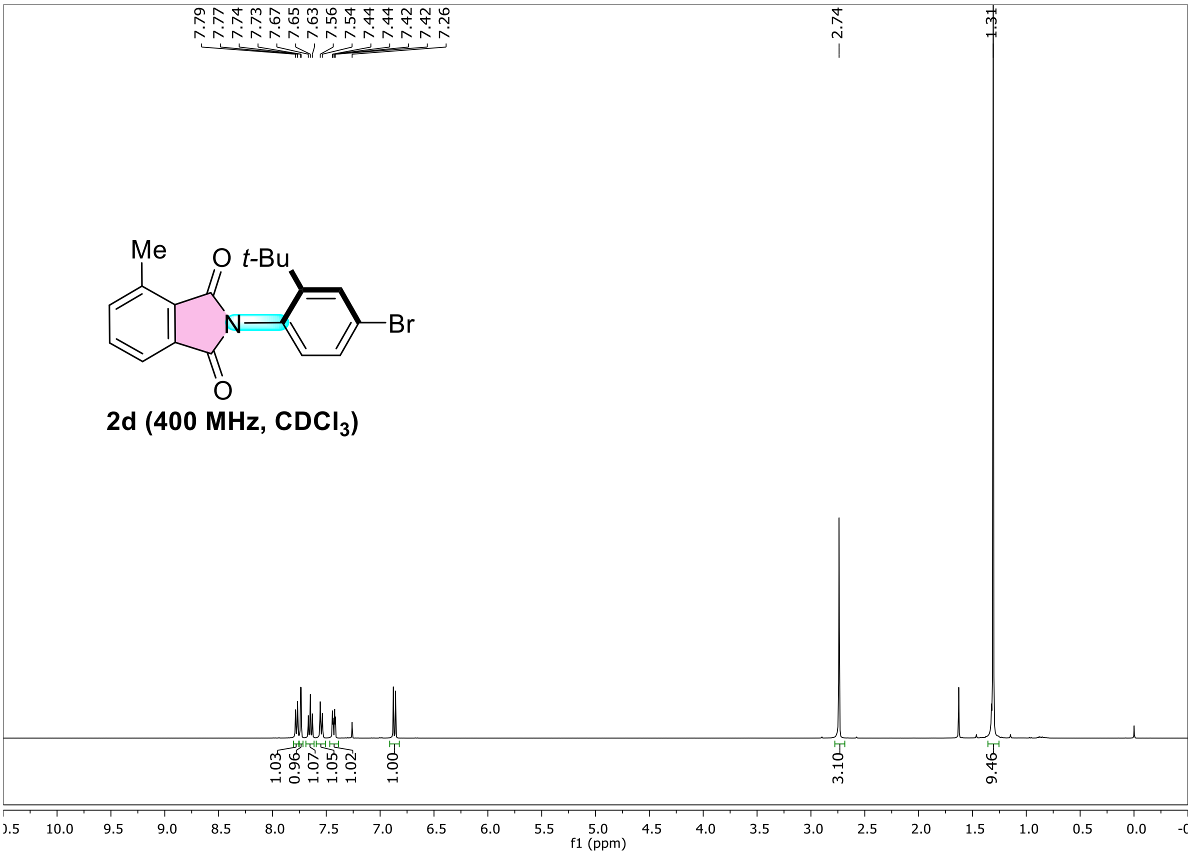

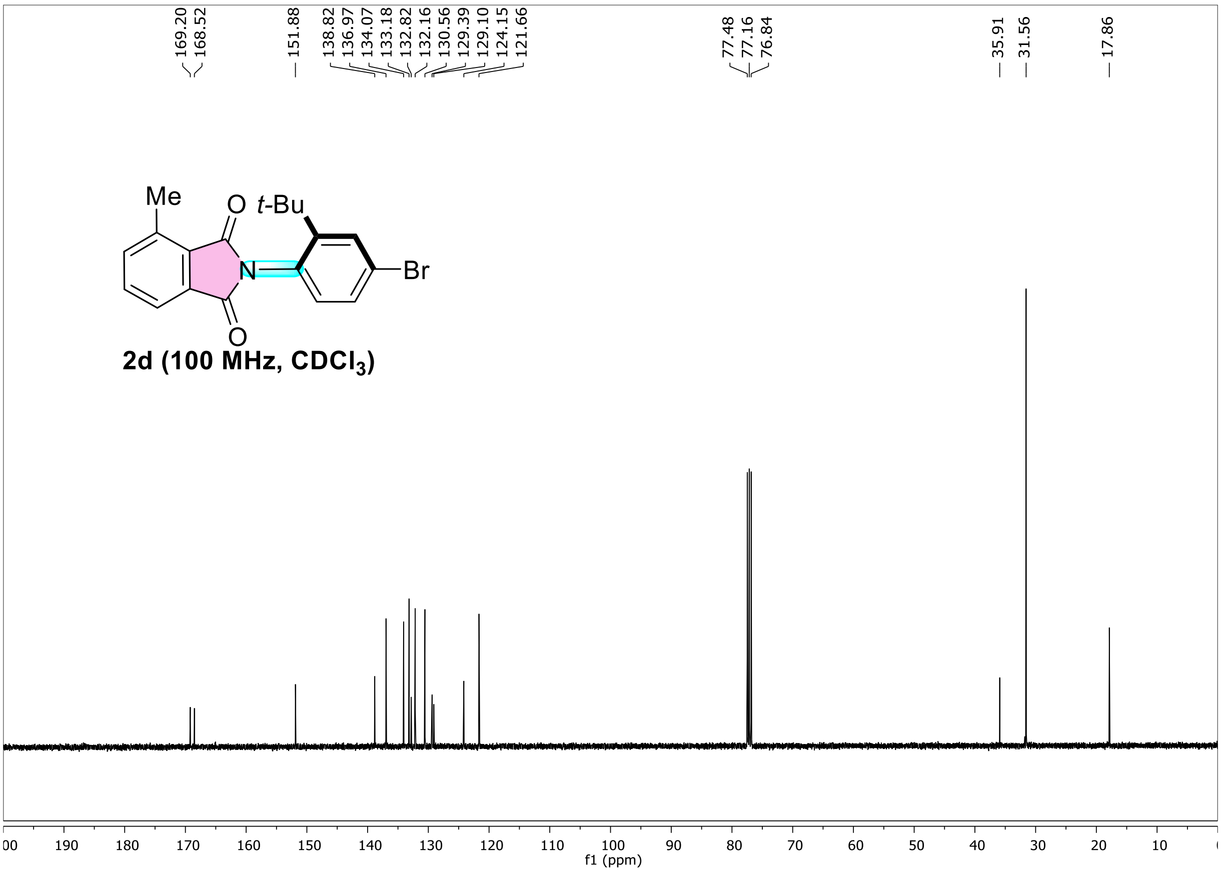

(*P*)-2-(2-(*tert*-Butyl)-4-chlorophenyl)-4-methylisoindoline-1,3-dione (2e)

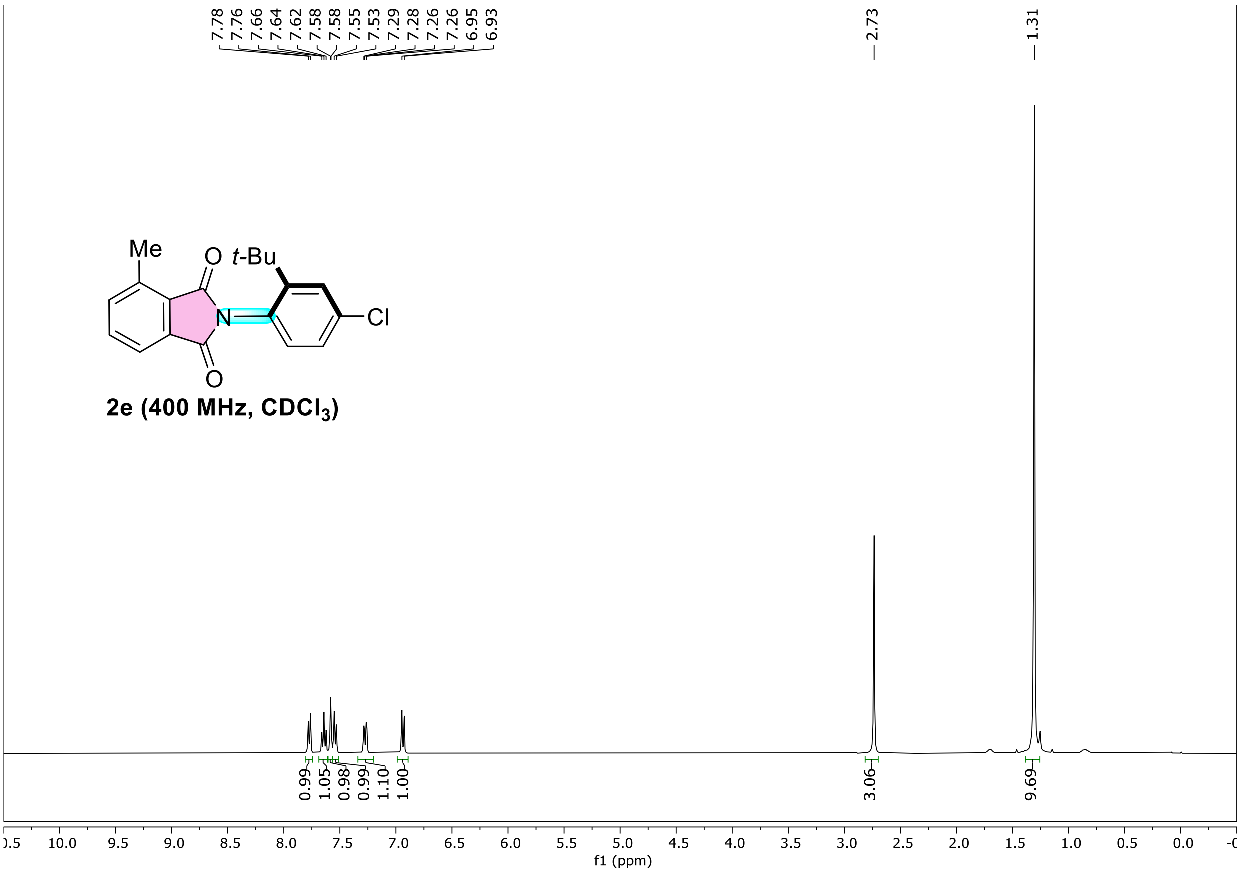

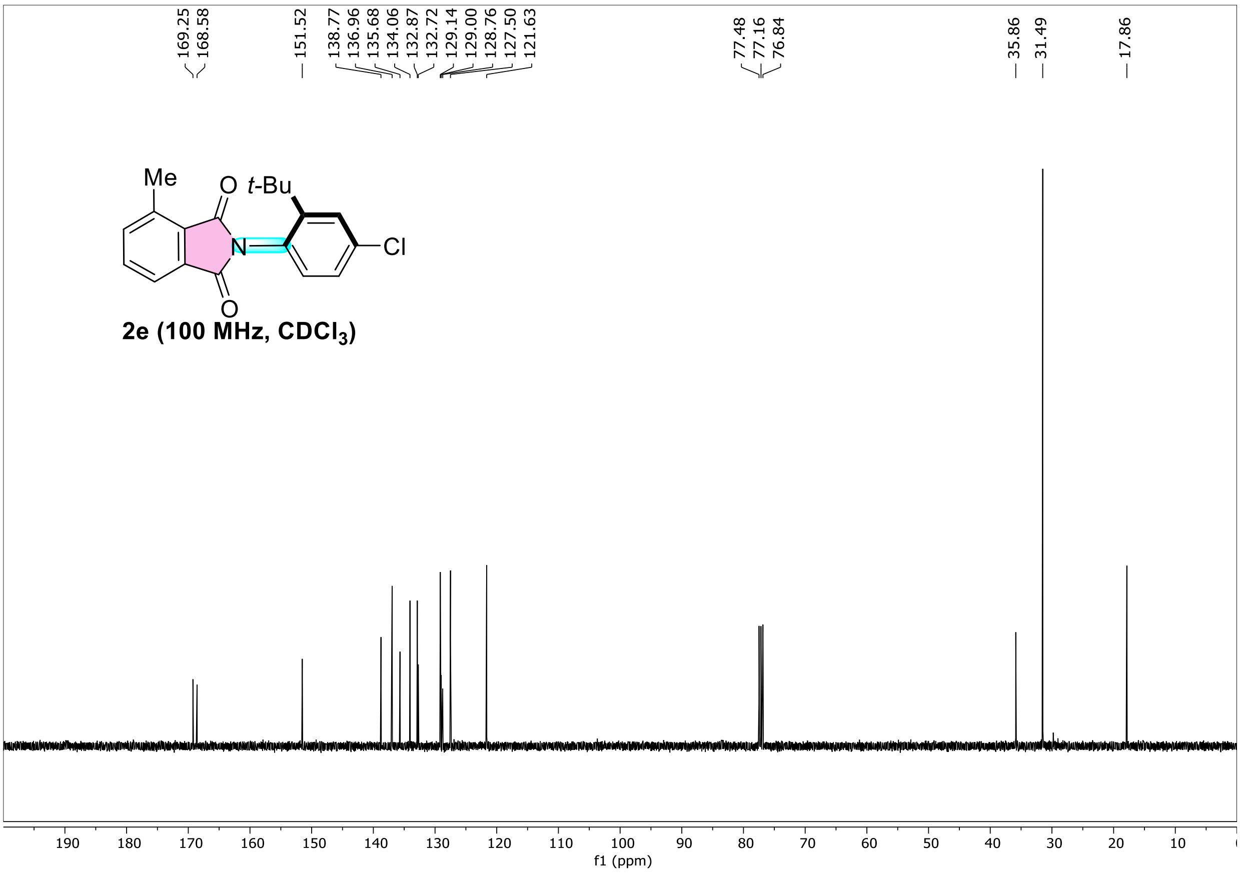

(*P*)-2-(2-(*tert*-Butyl)-4-iodophenyl)-4-methylisoindoline-1,3-dione (2f)

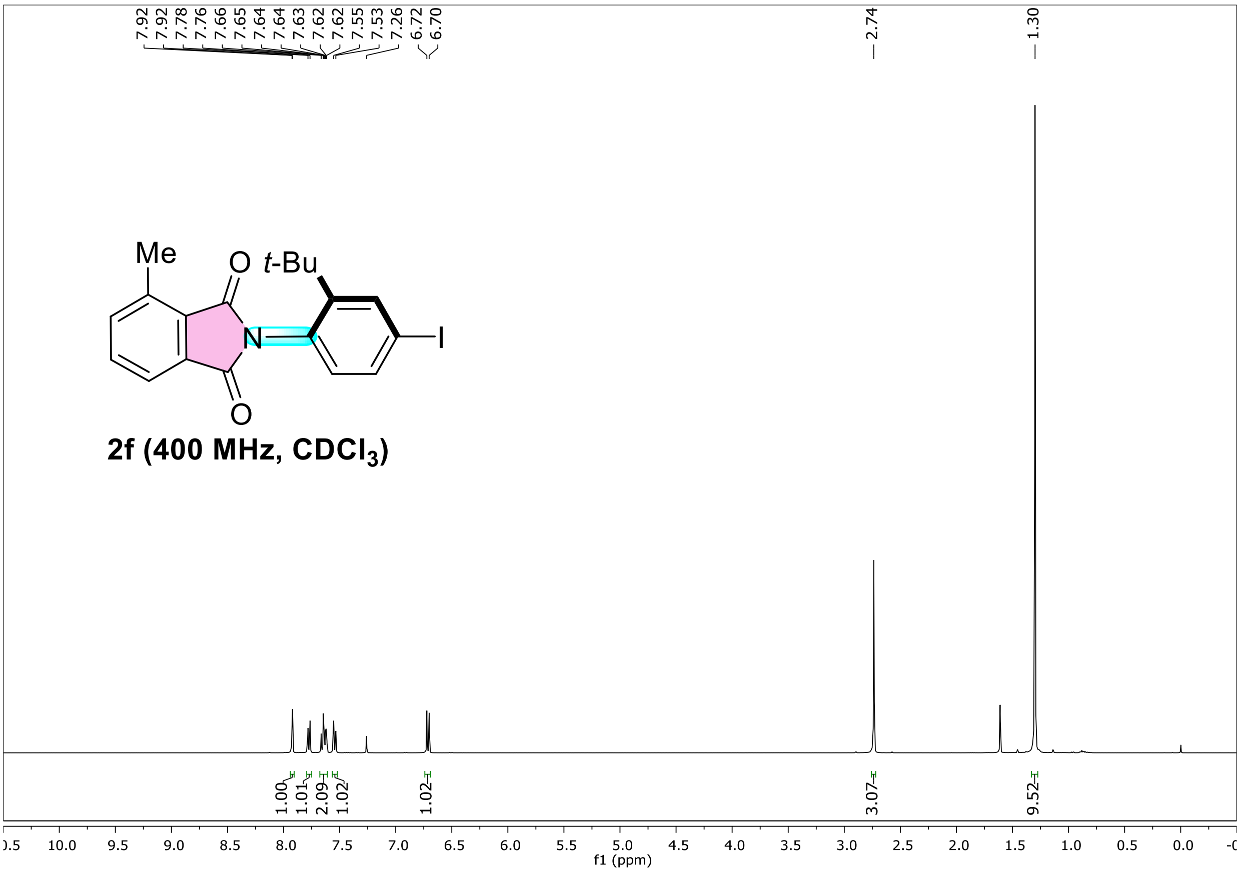

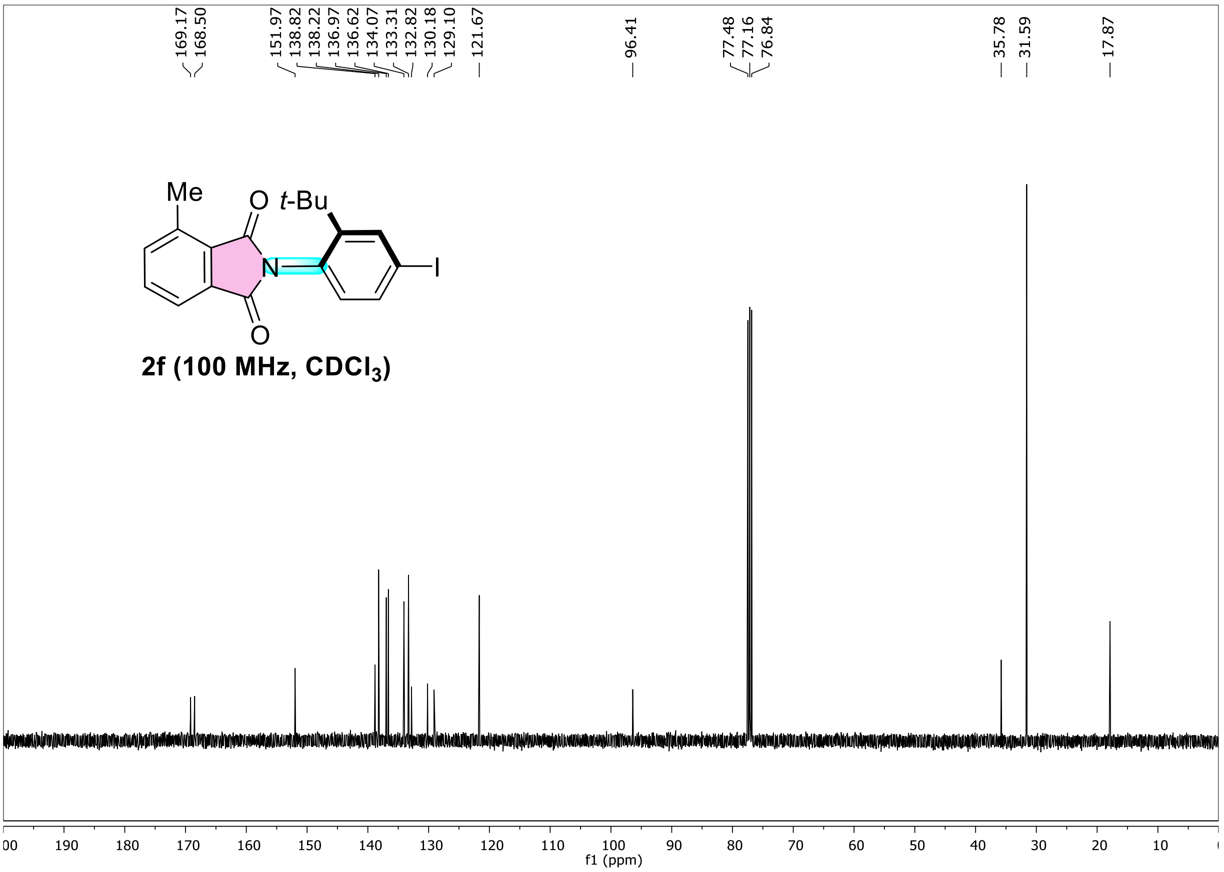

(*P*)-2-(3-(*tert*-Butyl)-[1,1'-biphenyl]-4-yl)-4-methylisoindoline-1,3-dione (2g)

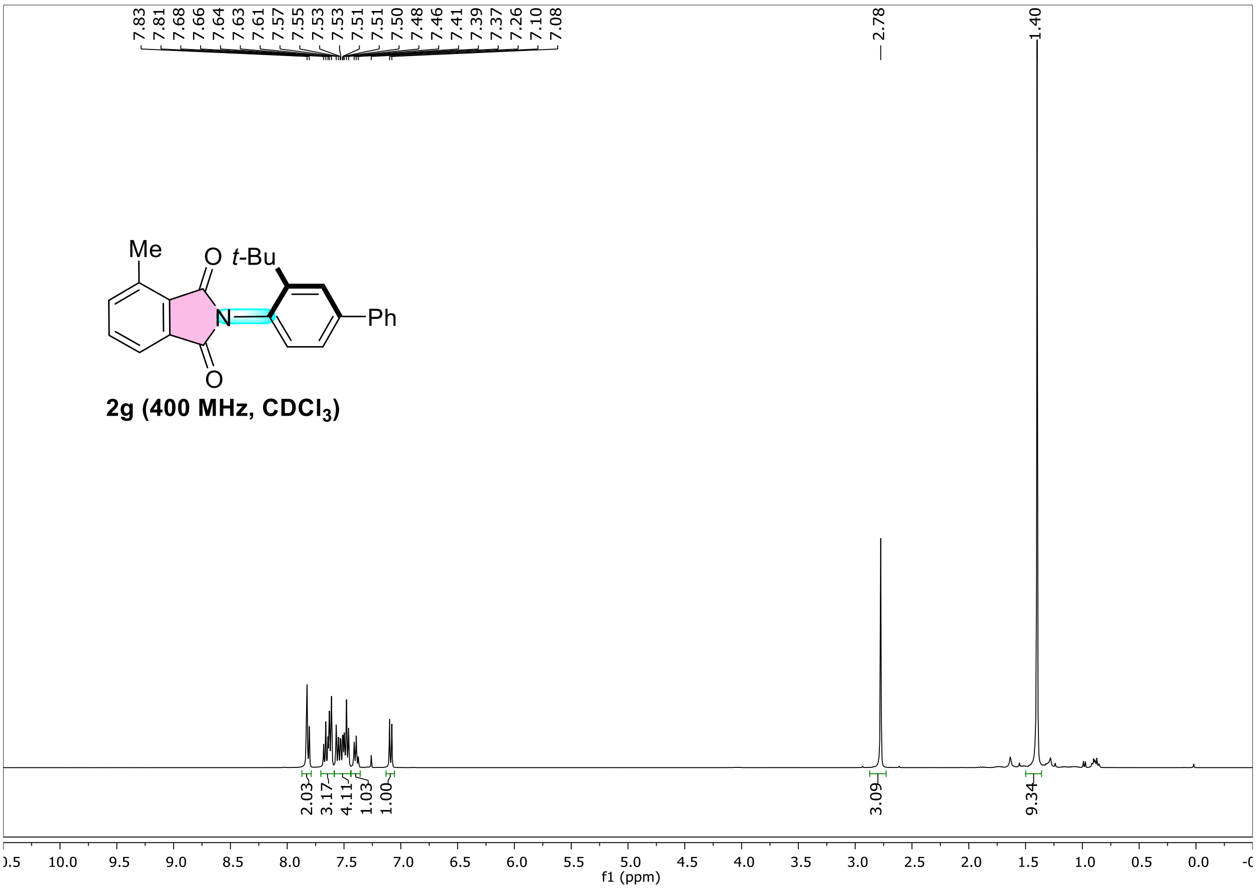

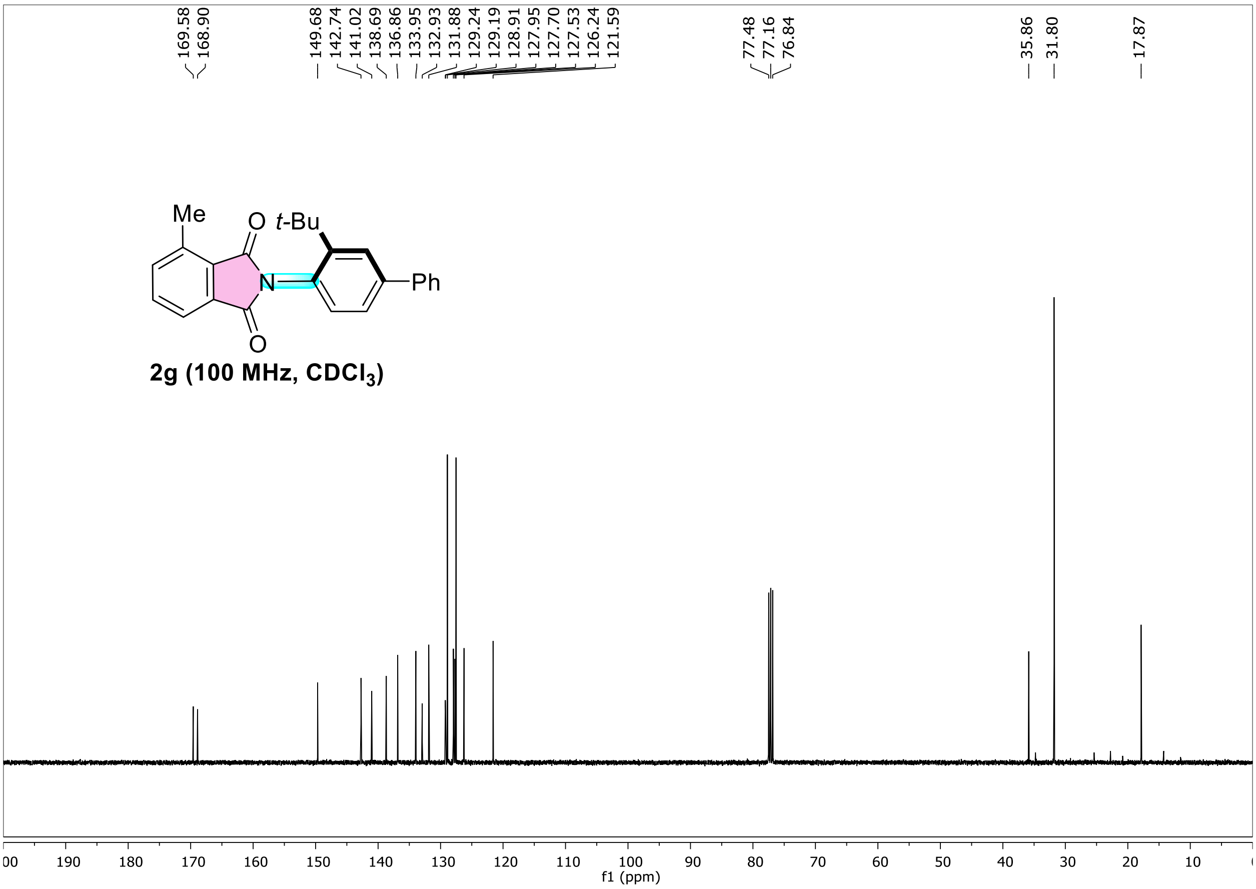

(*P*)-Ethyl 3-(*tert*-butyl)-4-(4-methyl-1,3-dioxoisoindolin-2-yl)benzoate (2h)

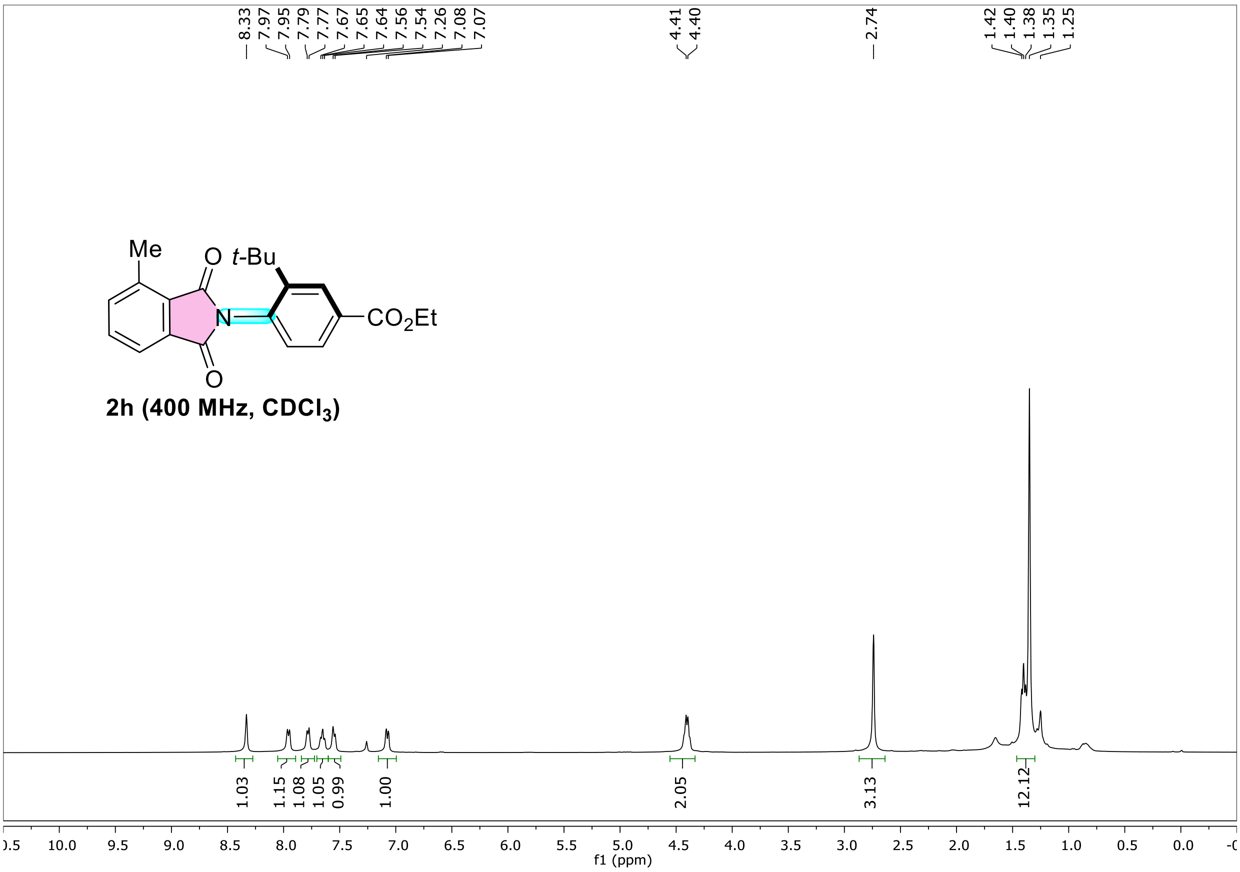

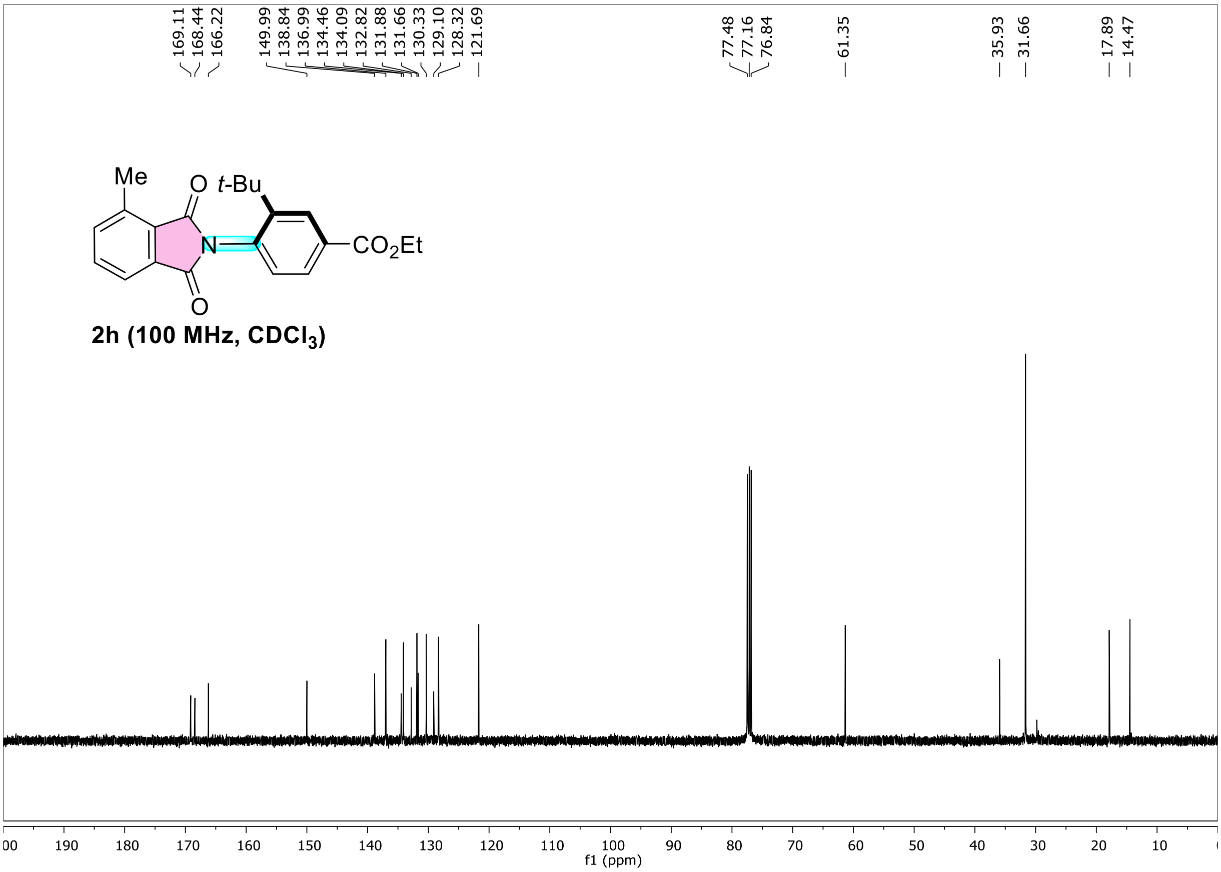

(*P*)-2-(2-(*tert*-Butyl)-4-(thiophen-3-yl)phenyl)-4-methylisoindoline-1,3-dione (2i)

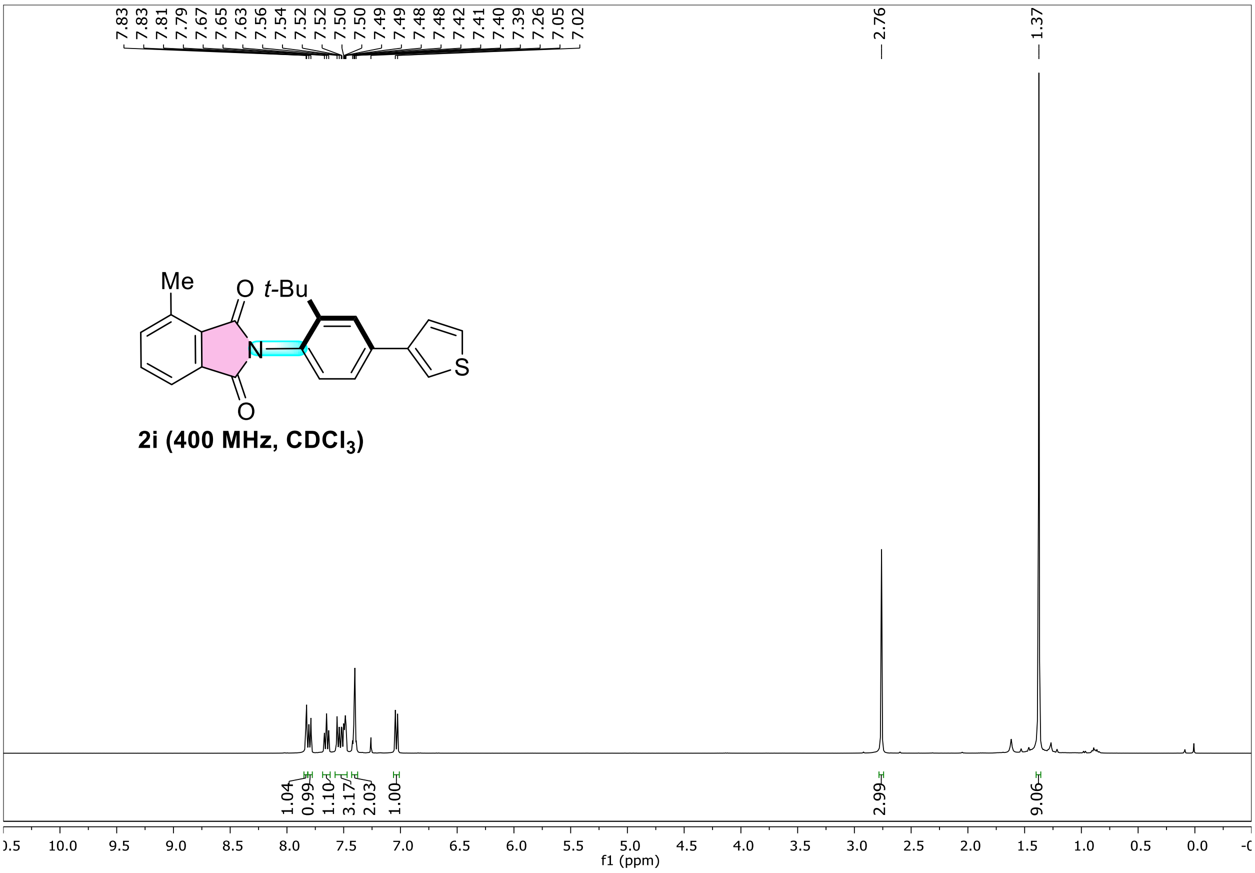

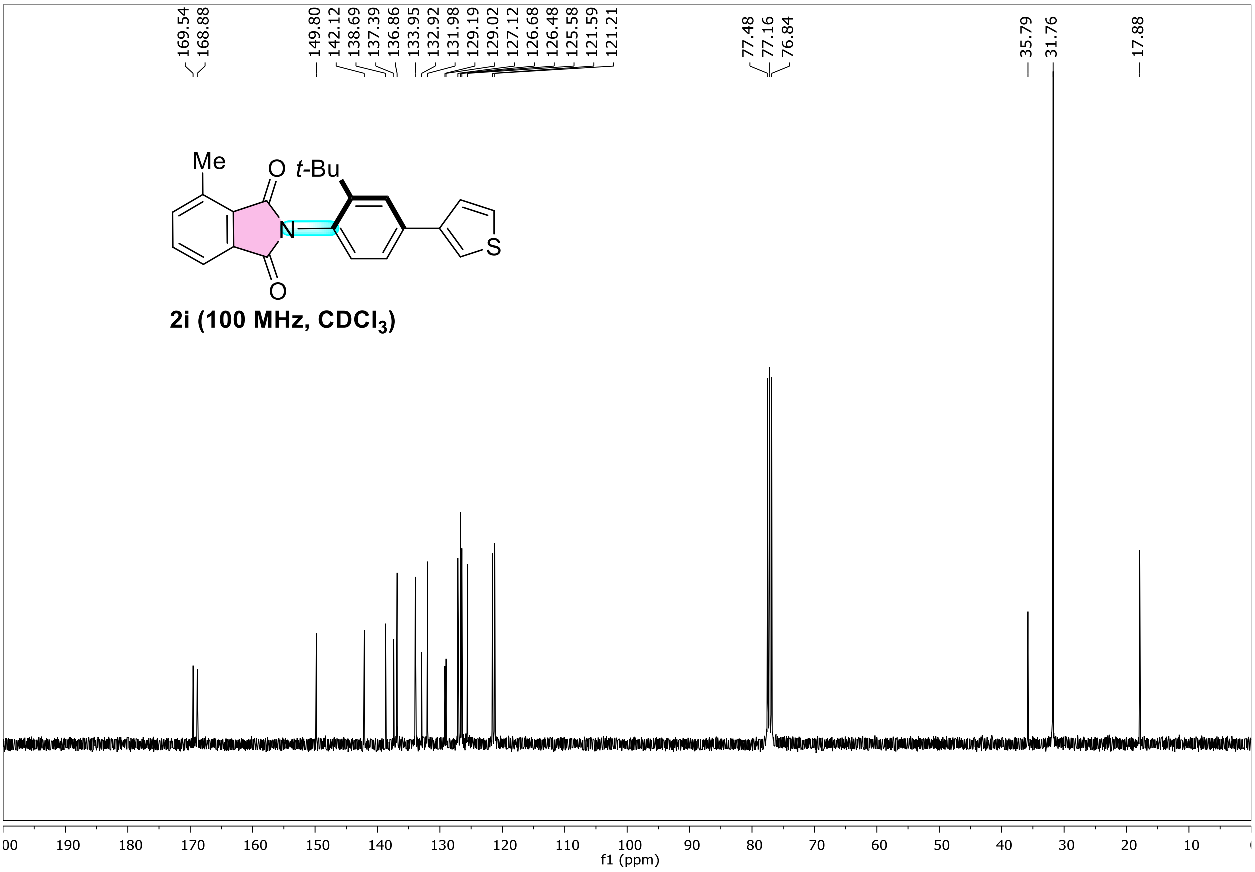

(*P*, *E*)-2-(2-(*tert*-Butyl)-4-styrylphenyl)-4-methylisoindoline-1,3-dione (2j)

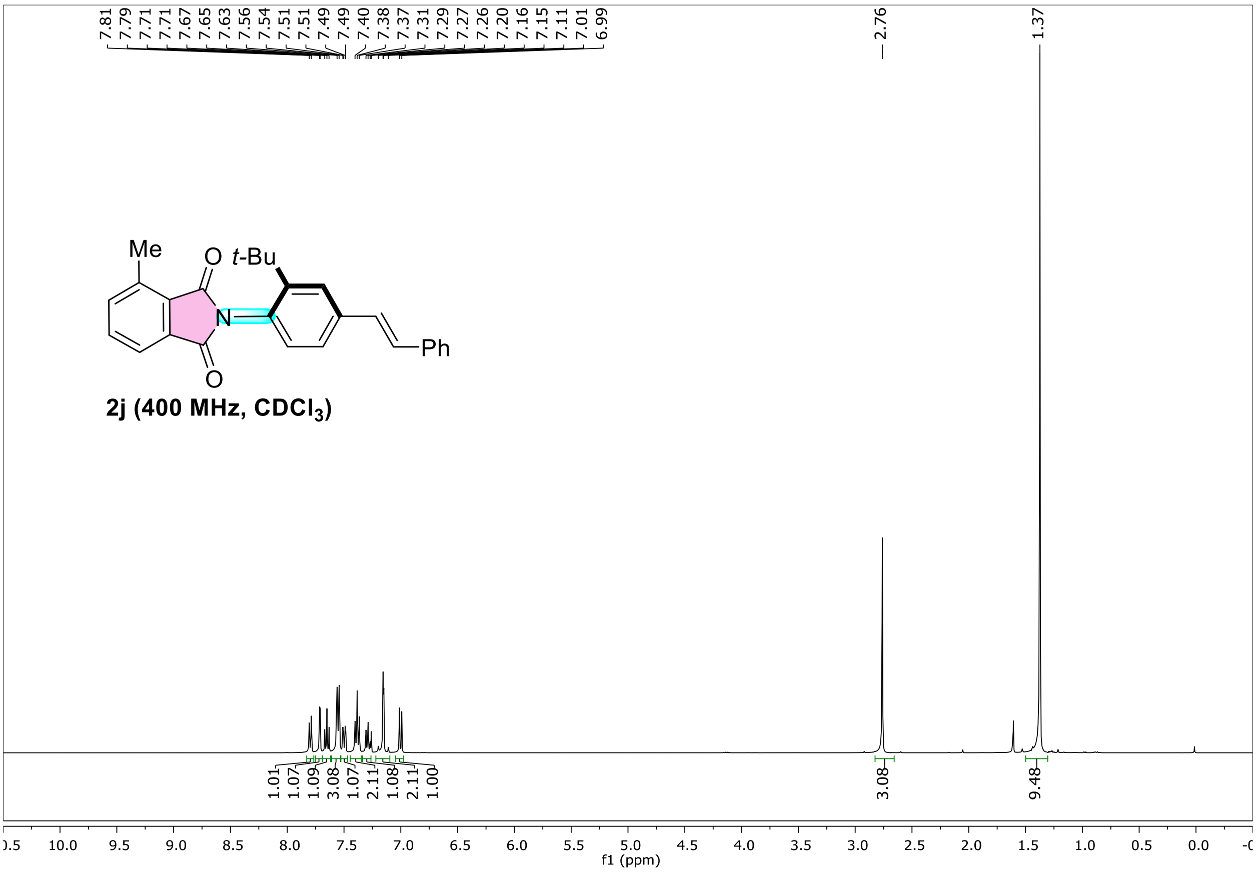

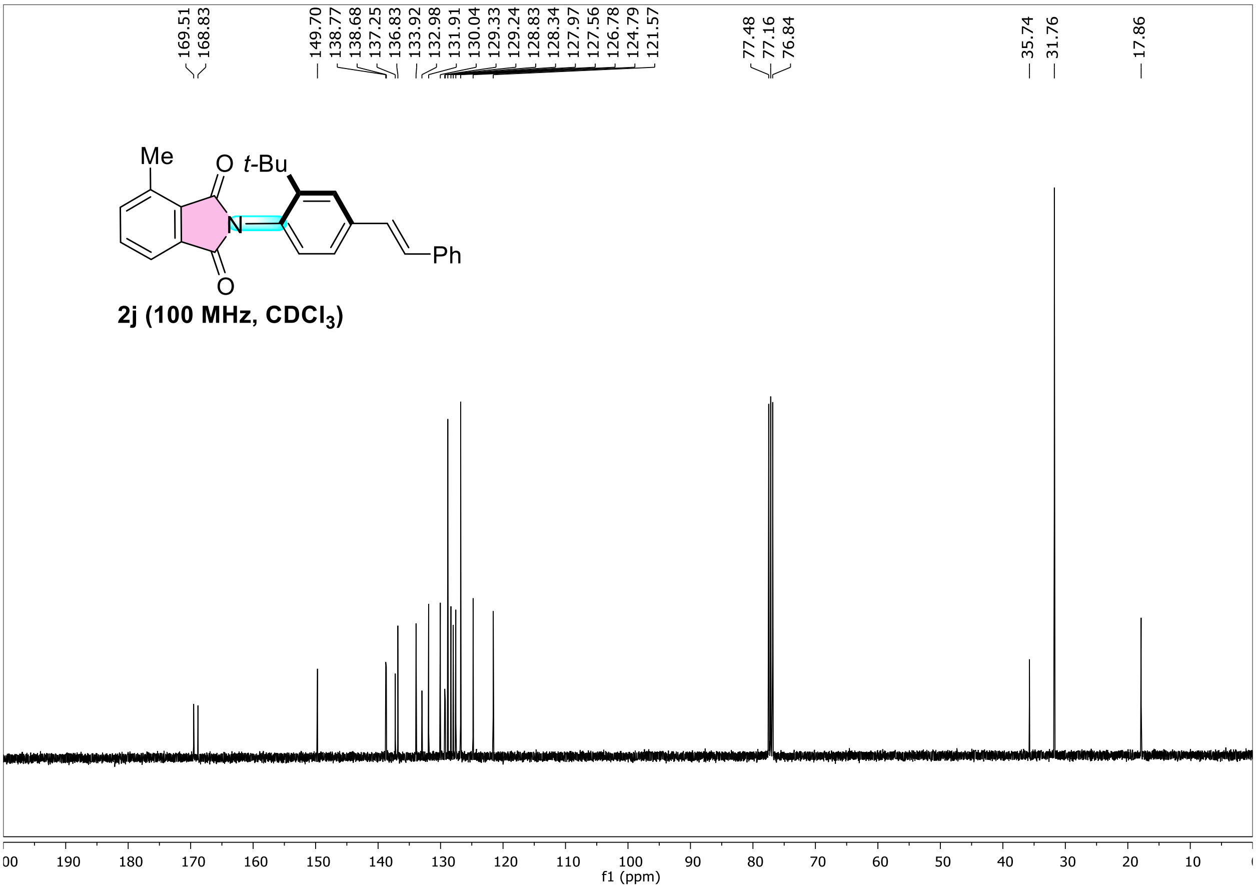

(*P*)-Methyl-(*E*)-3-(3-(*tert*-butyl)-4-(4-methyl-1,3-dioxoisoindolin-2-yl)phenyl)acrylate (2k)

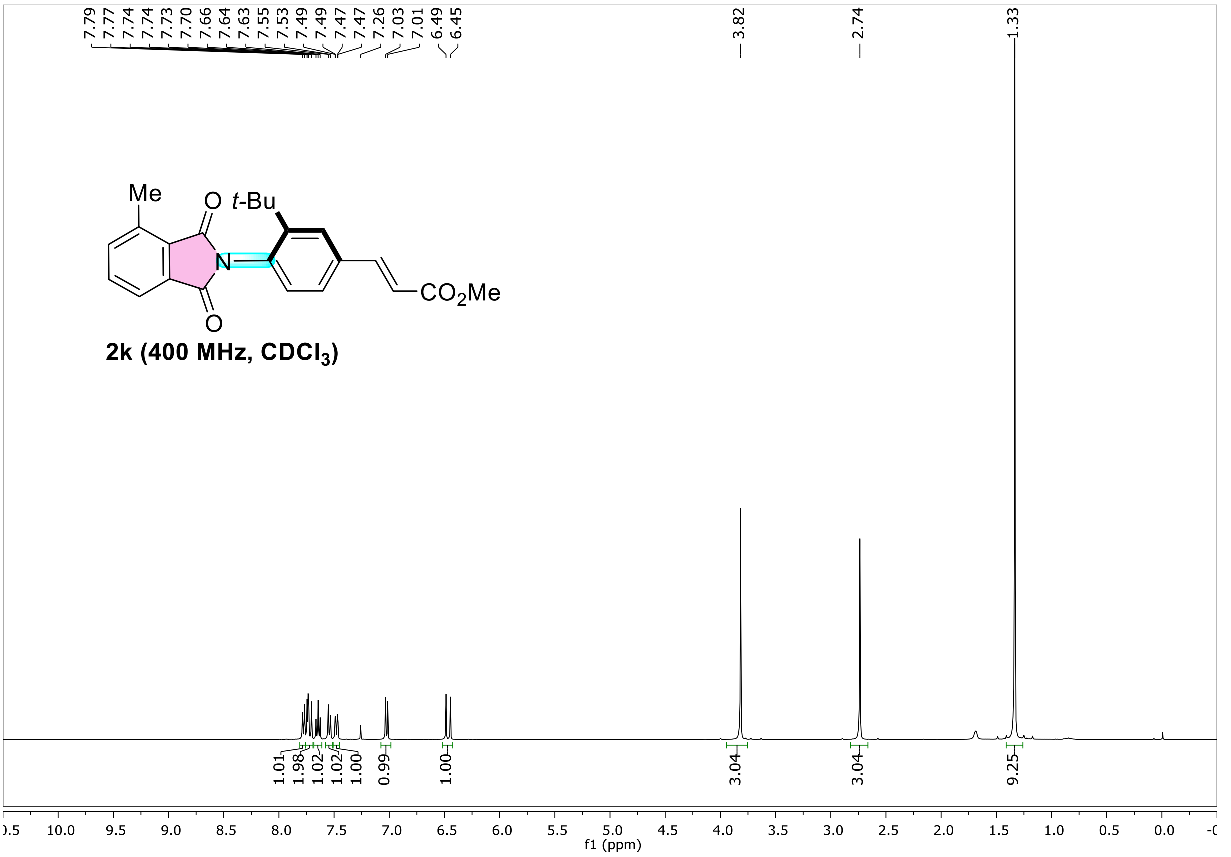

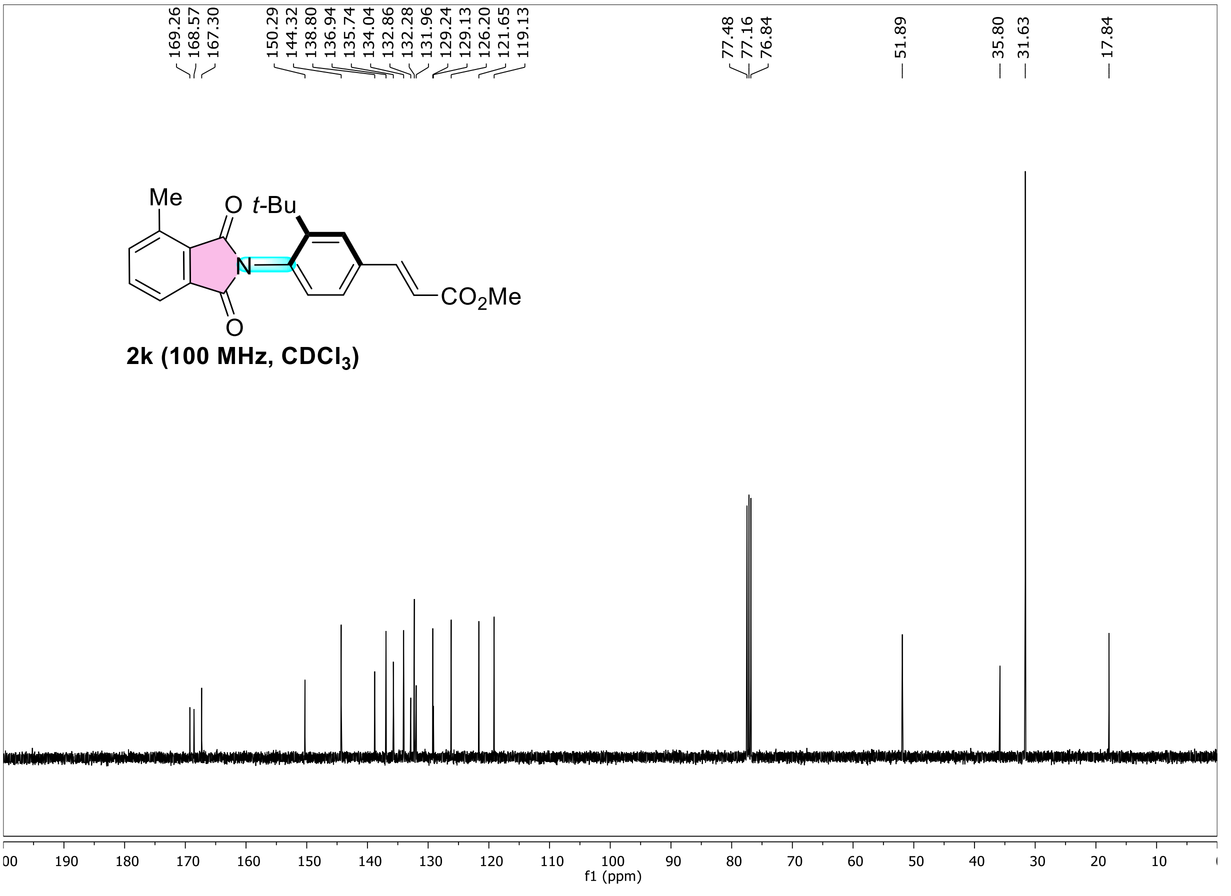

(*P*)-2-(2-(*tert*-Butyl)-4-(phenylethynyl)phenyl)-4-methylisoindoline-1,3-dione (2l)

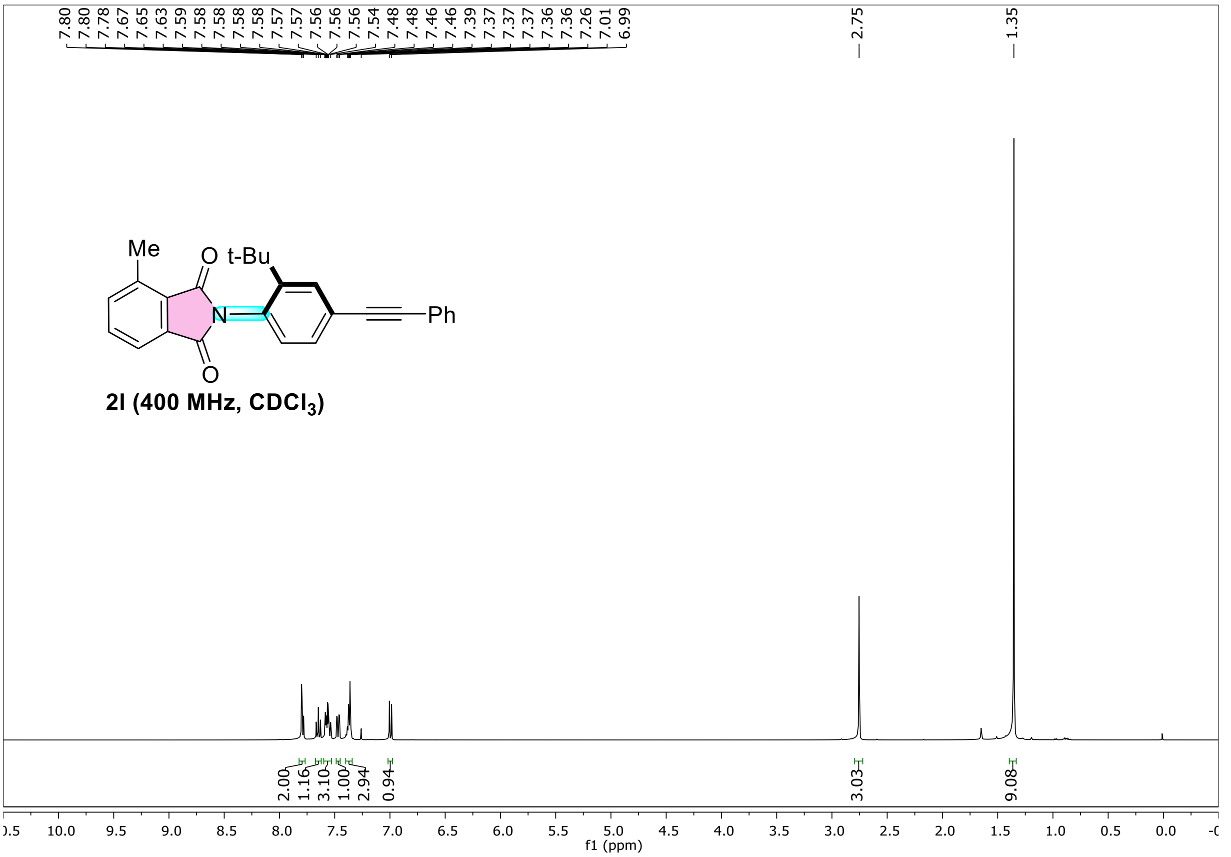

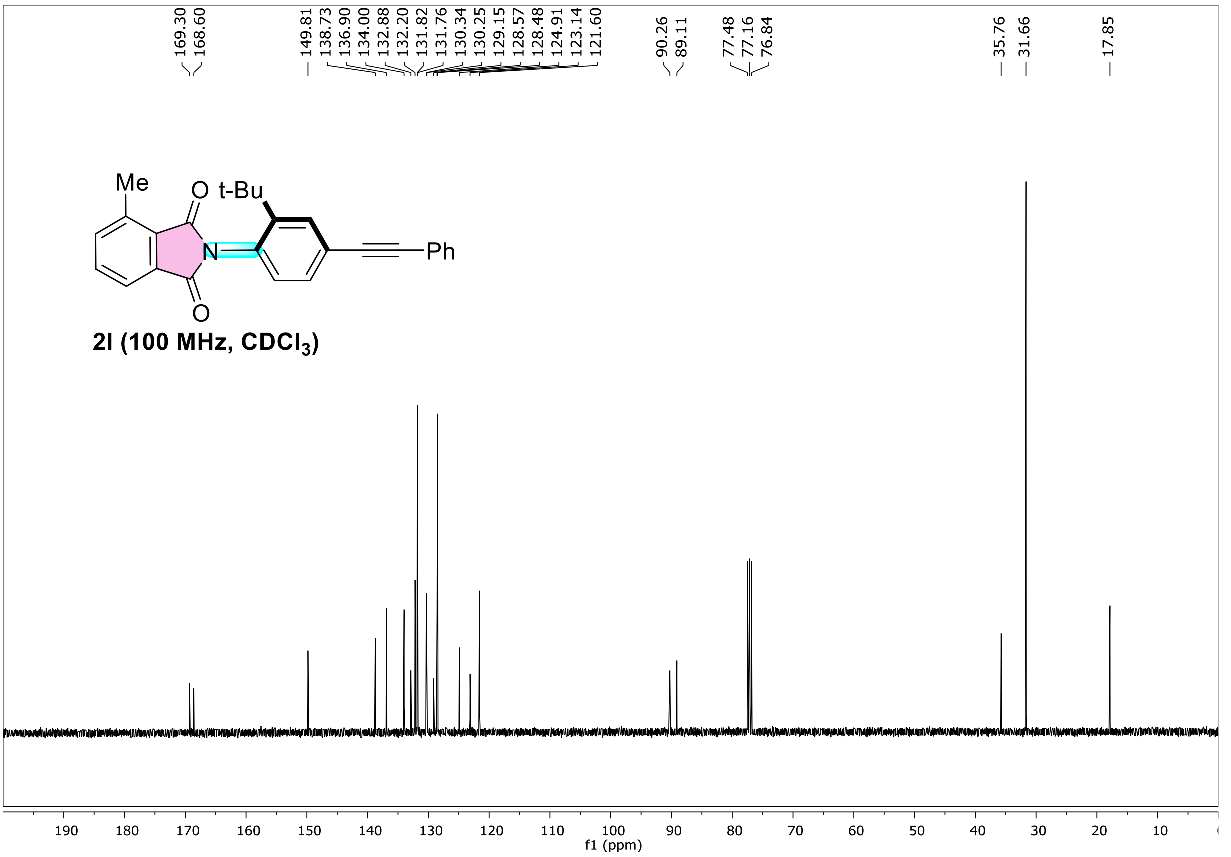

(*P*)-2-(2-(*tert*-Butyl)-5-nitrophenyl)-4-methylisoindoline-1,3-dione (2m)

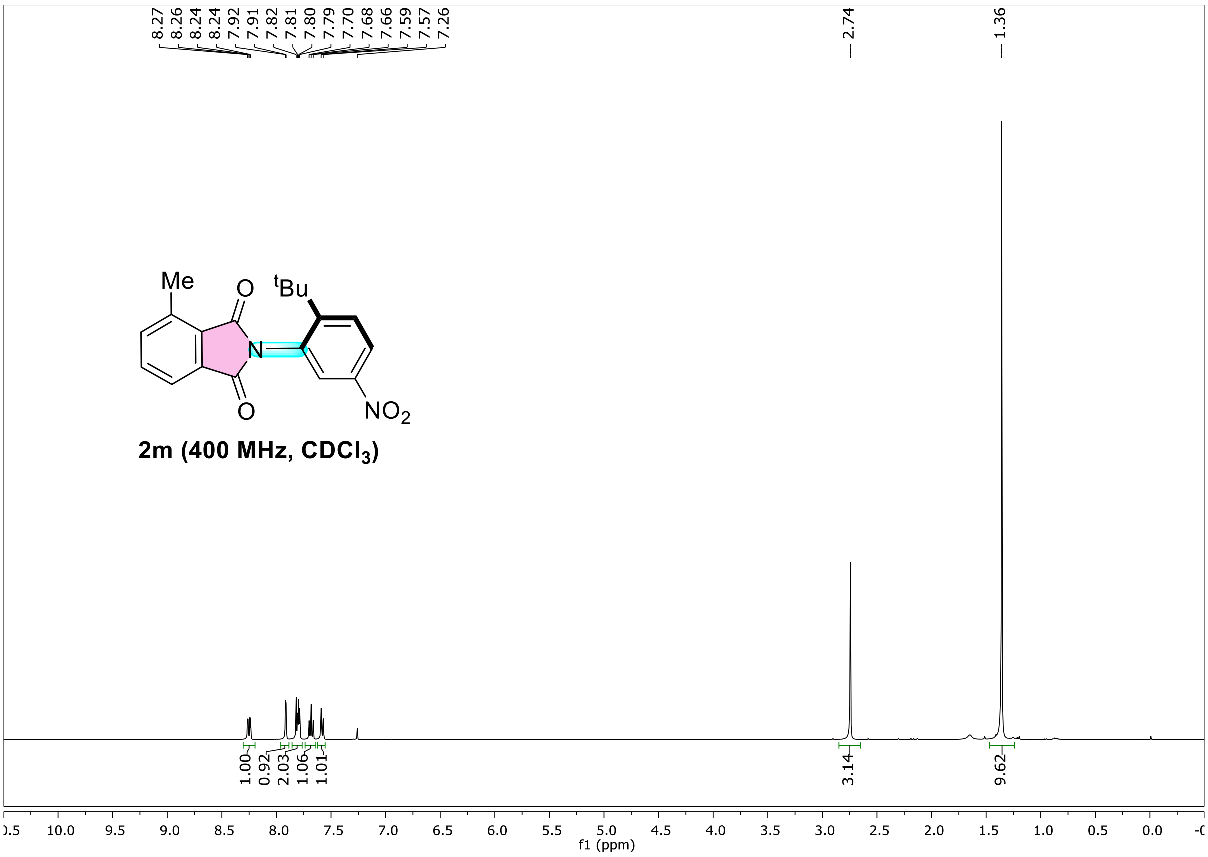

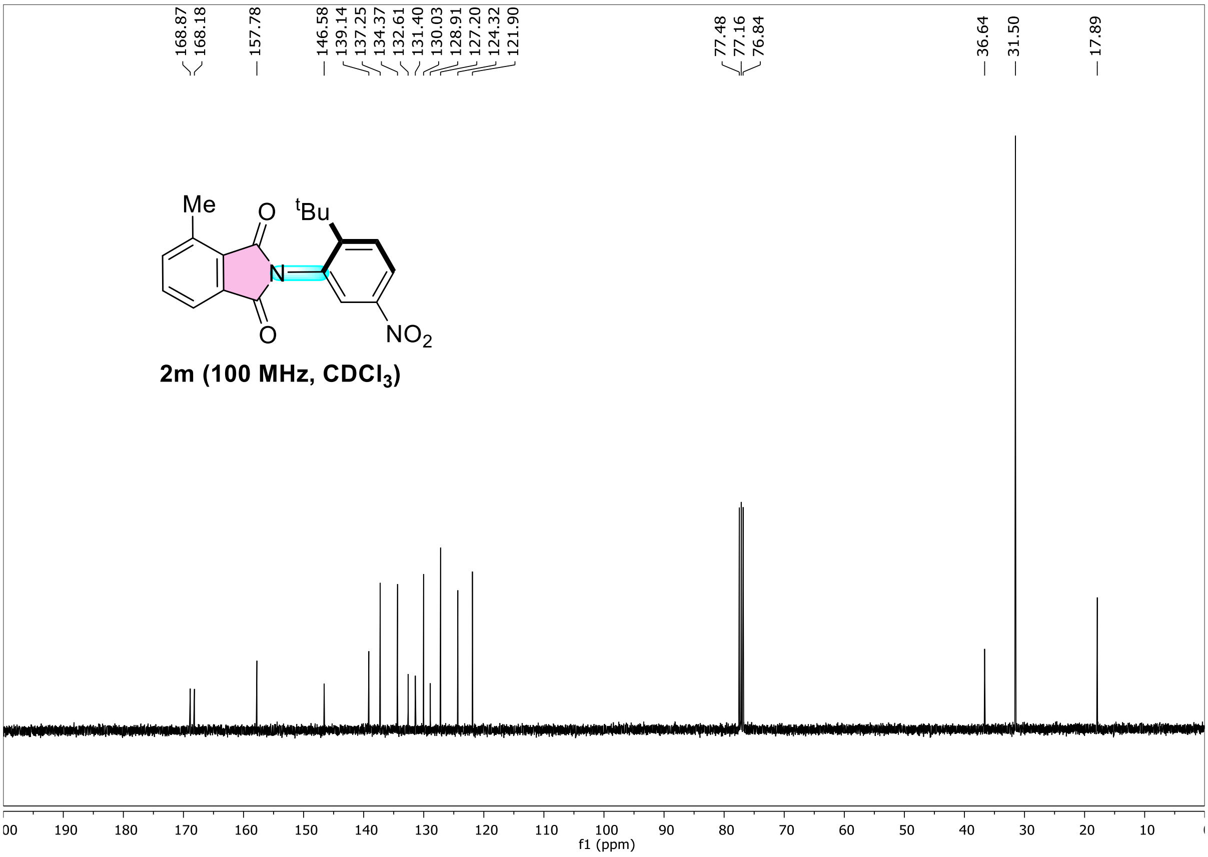

(*P*)-4-Methyl-2-(4-methyl-2-(2-phenylpropan-2-yl)phenyl)isoindoline-1,3-dione (2n)

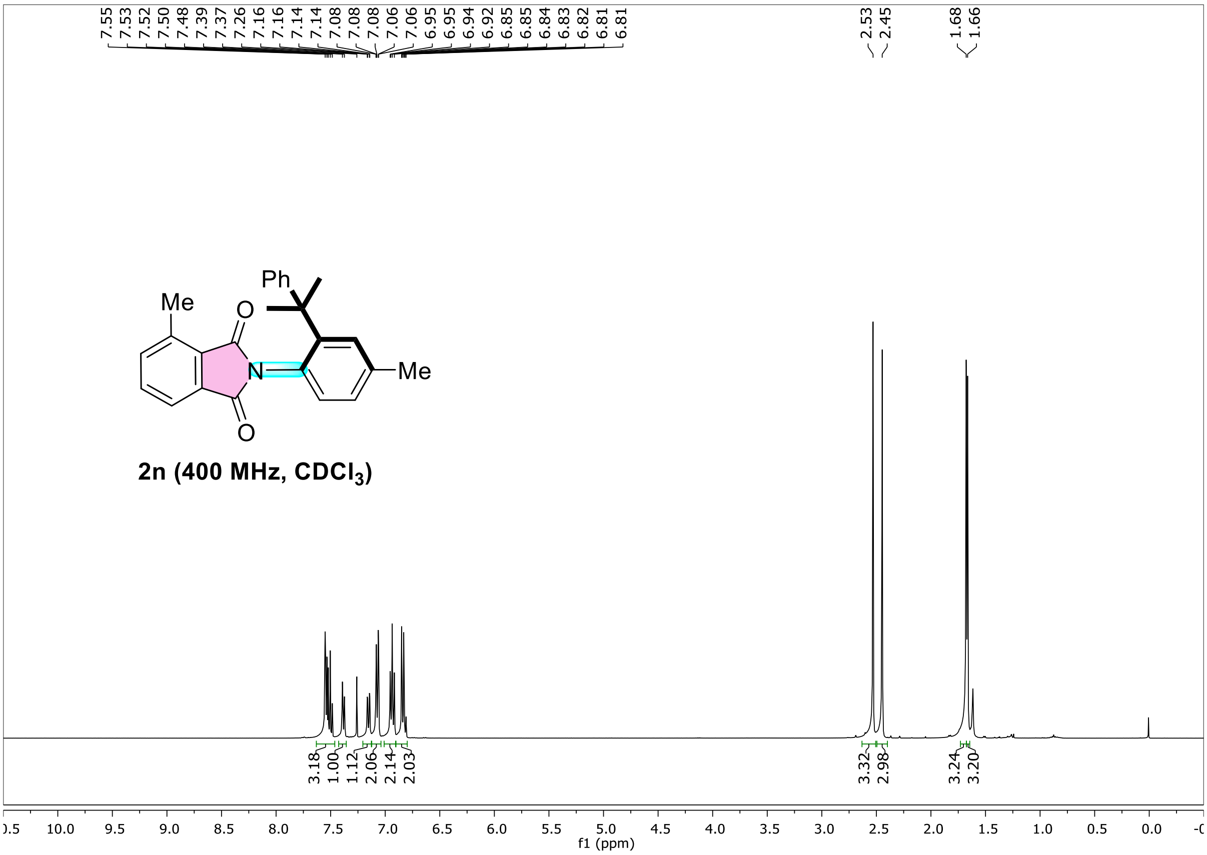

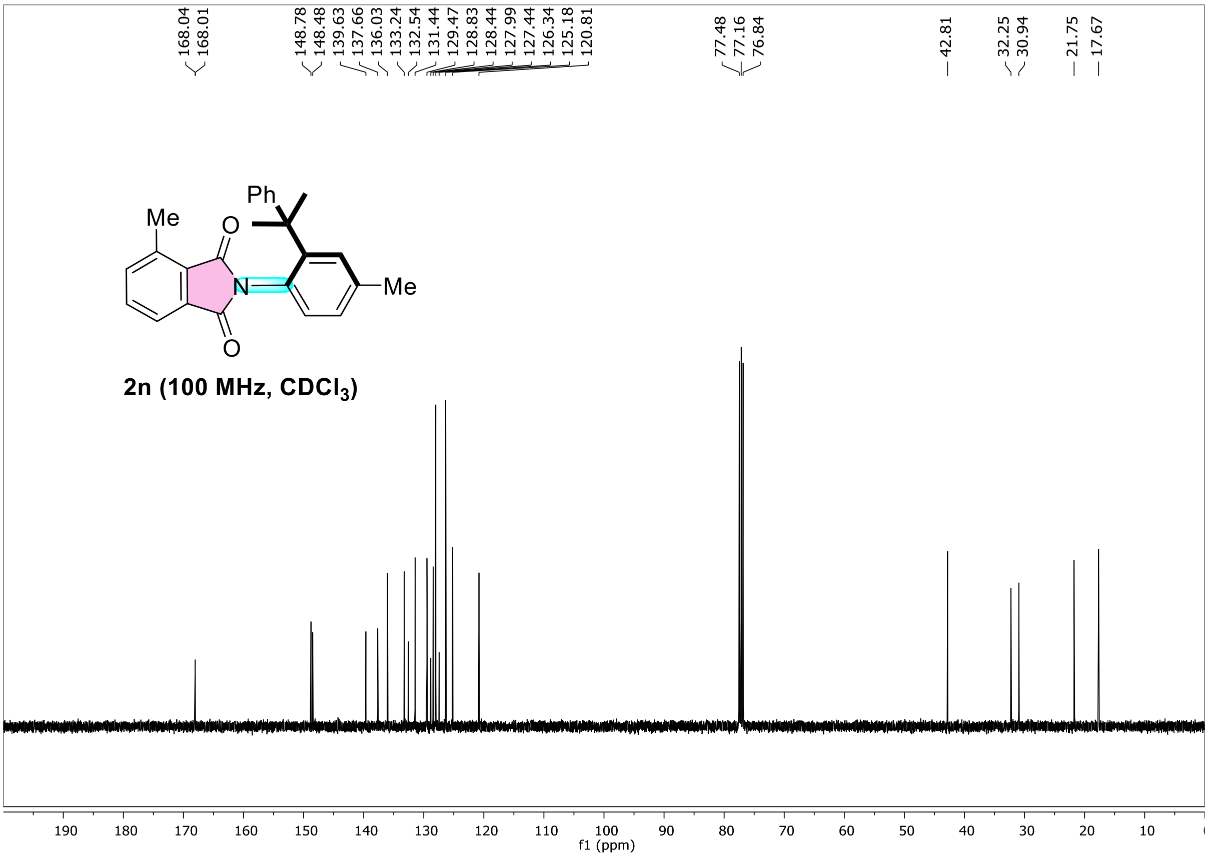

(*P*)-2-(2-(1-Methoxy-2-methylpropan-2-yl)-4-methylphenyl)-4-methylisoindoline-1,3-dione (2o)

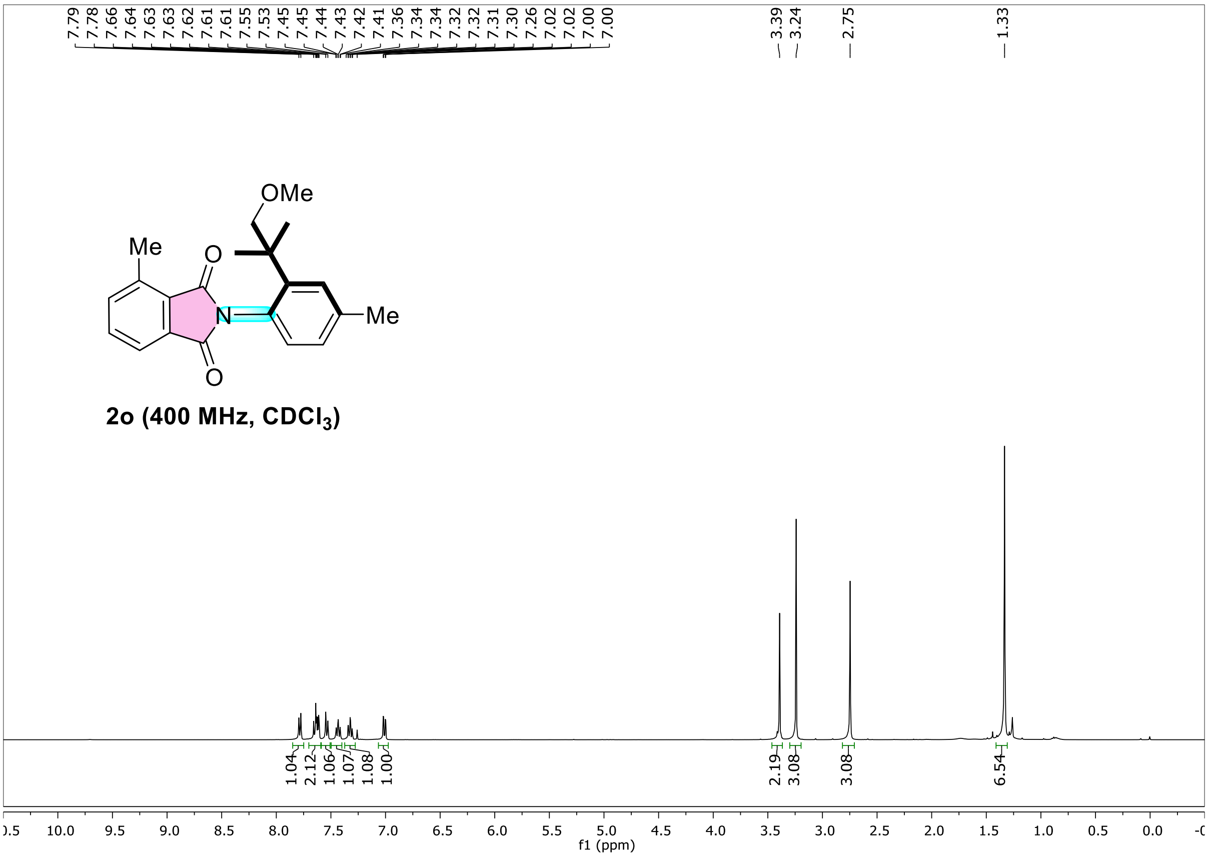

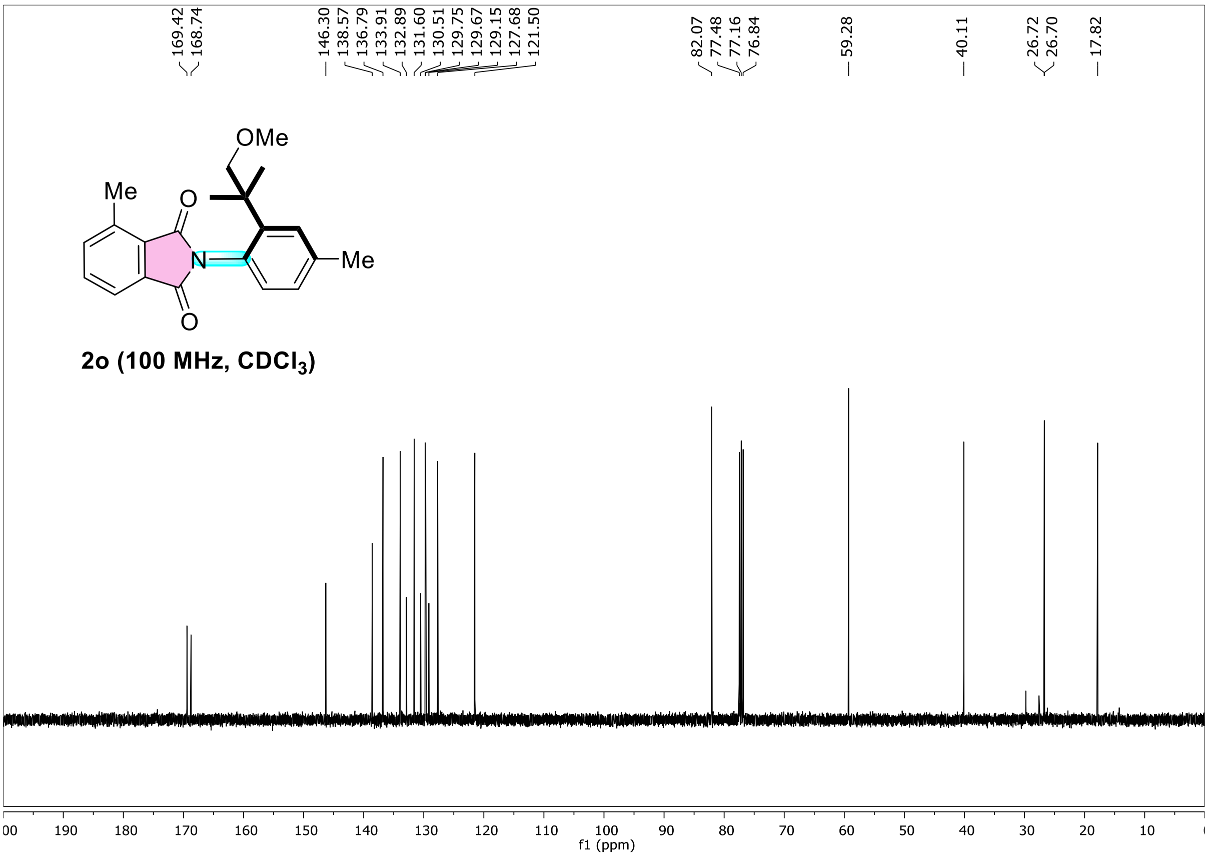

(*P*)- 2-(2-(Methoxydiphenylmethyl)phenyl)-4-methylisoindoline-1,3-dione (2p)

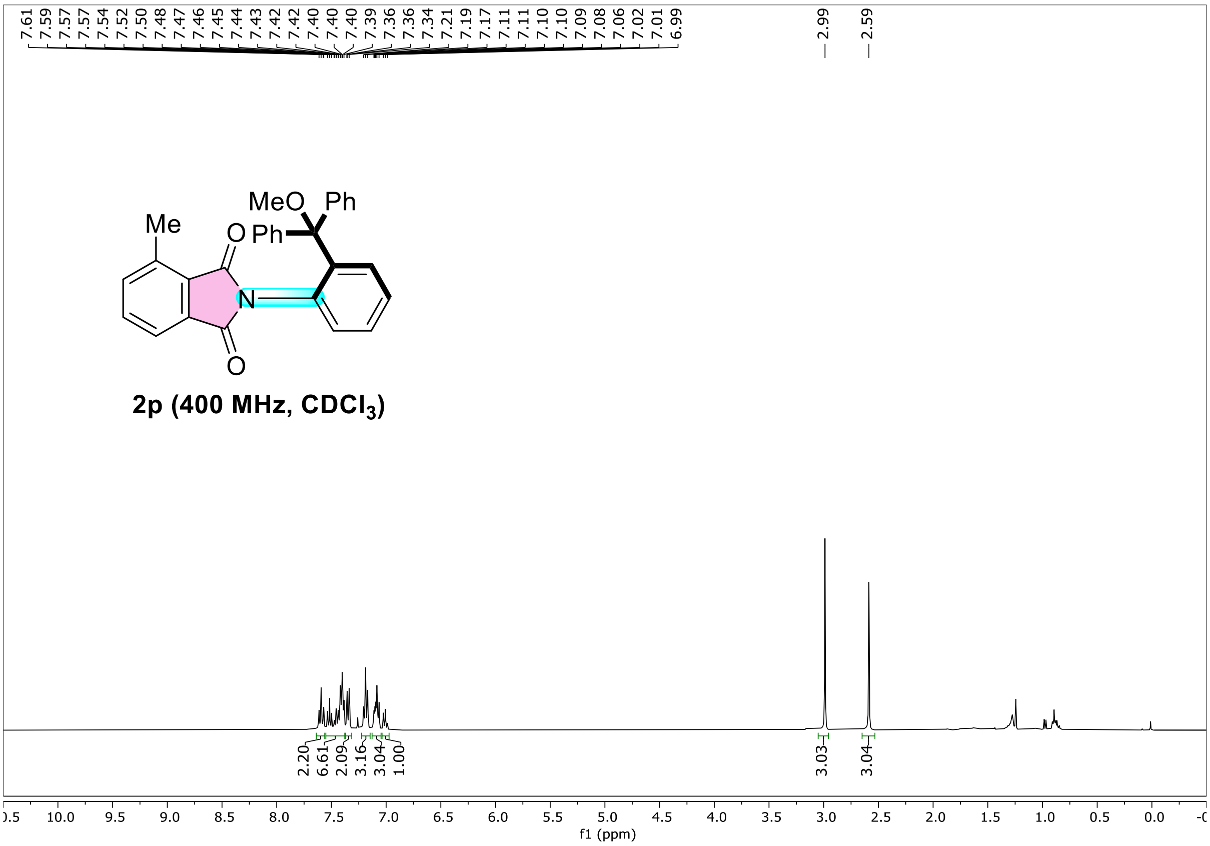

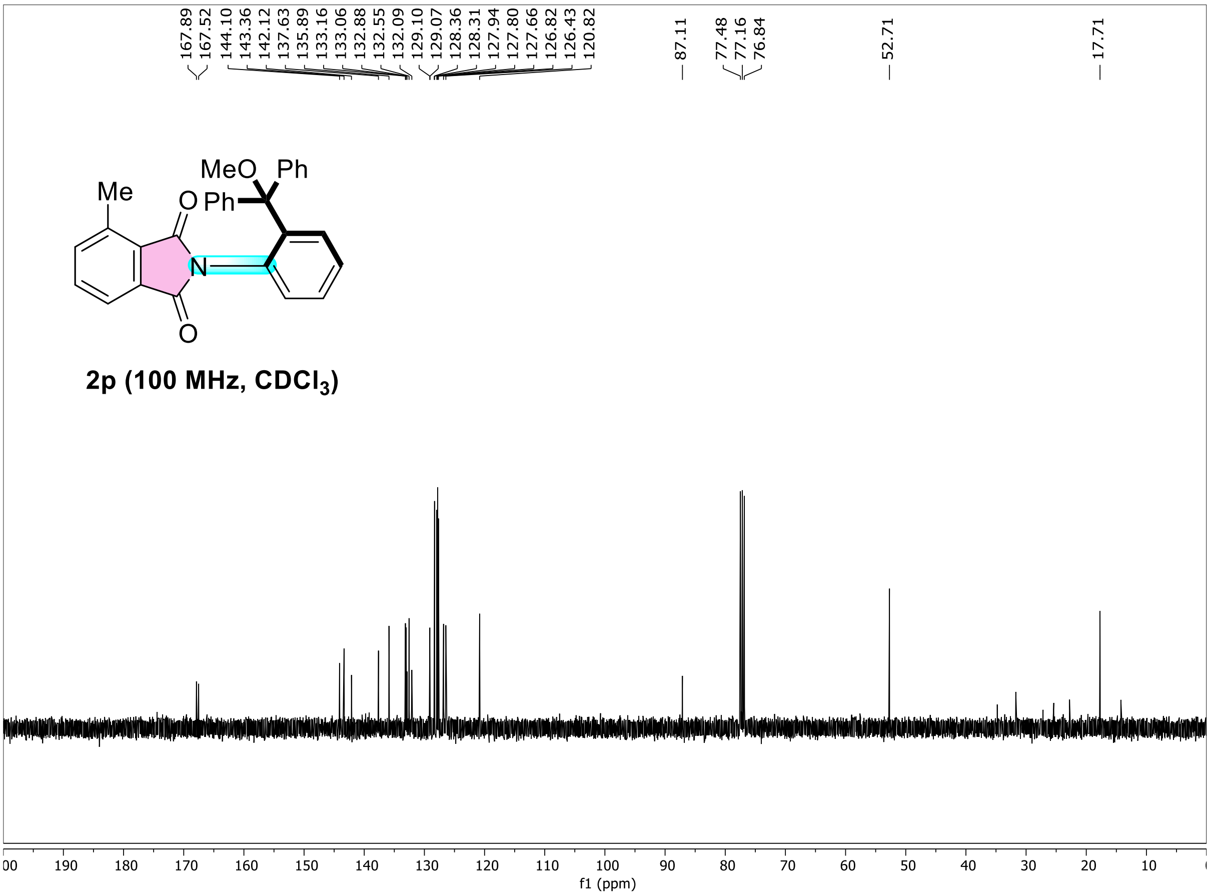

(*P*)-2-(2-(Di(thiophen-2-yl)((trimethylsilyl)oxy)methyl)phenyl)-4-methylisoindoline-1,3-dione (2q)

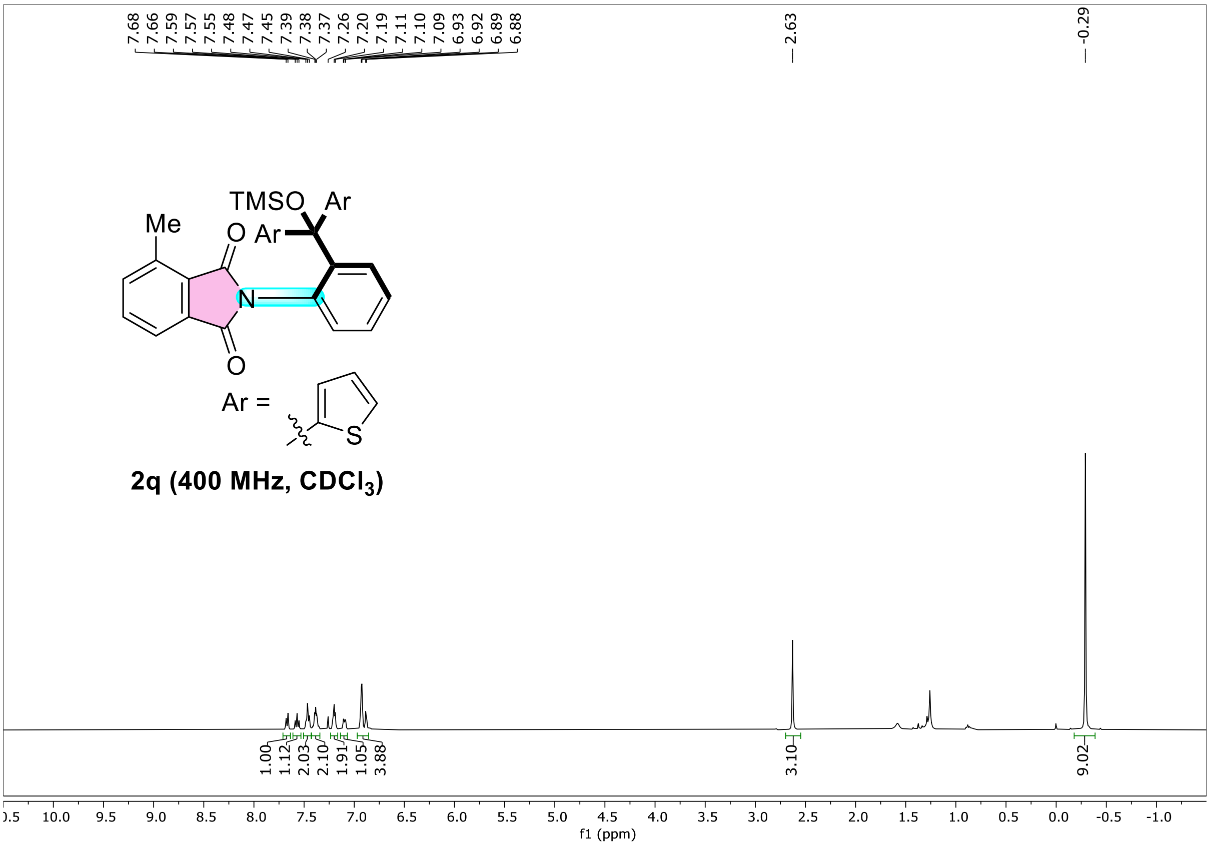

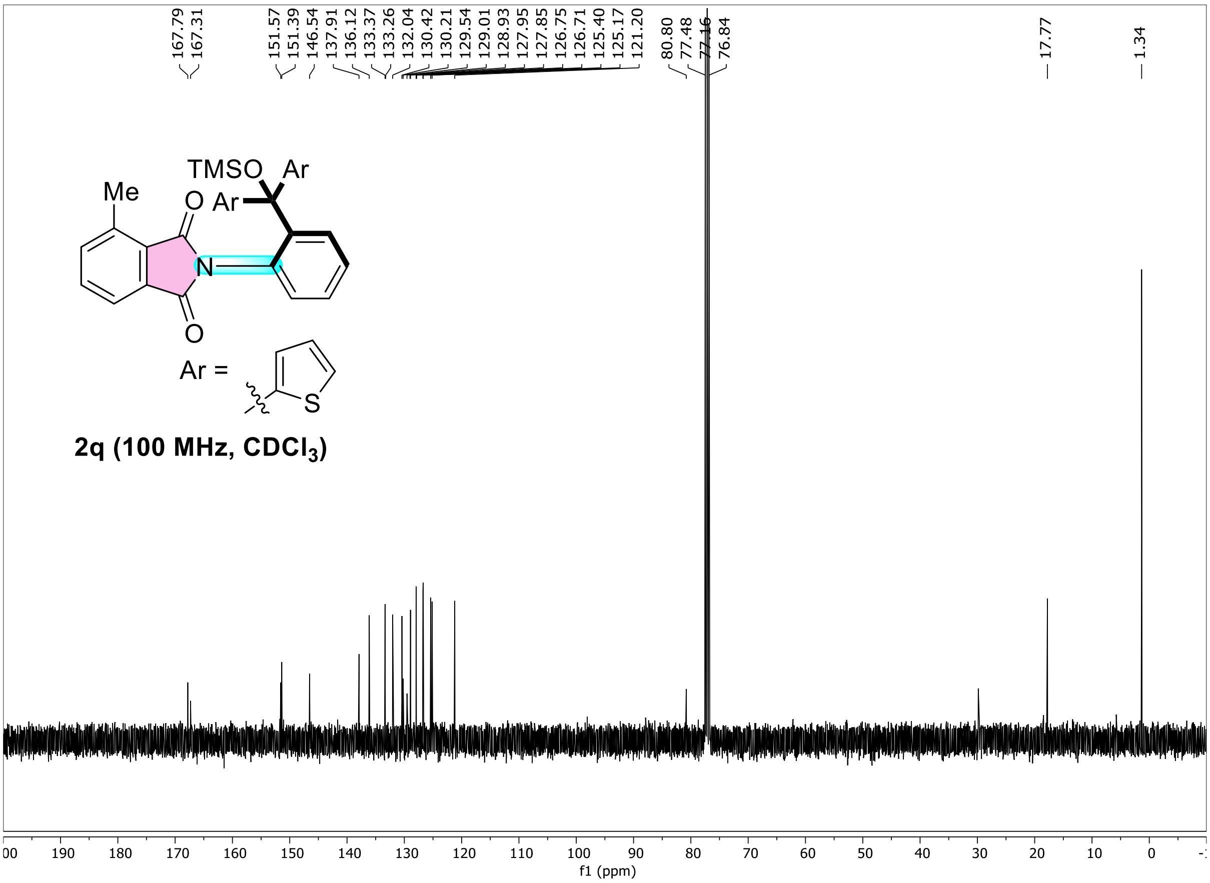

(*P*)-4-Methyl-2-(2-(phenylsulfonyl)phenyl)isoindoline-1,3-dione (2r)

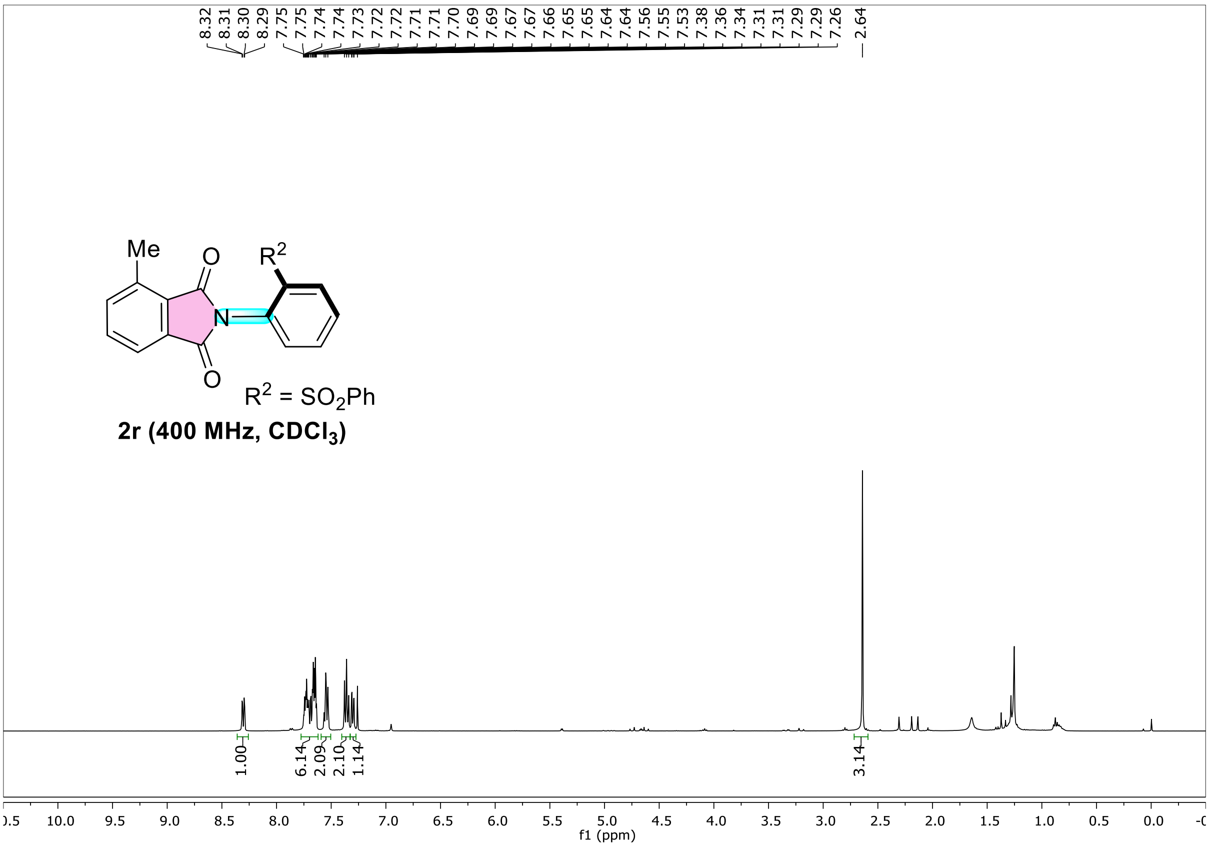

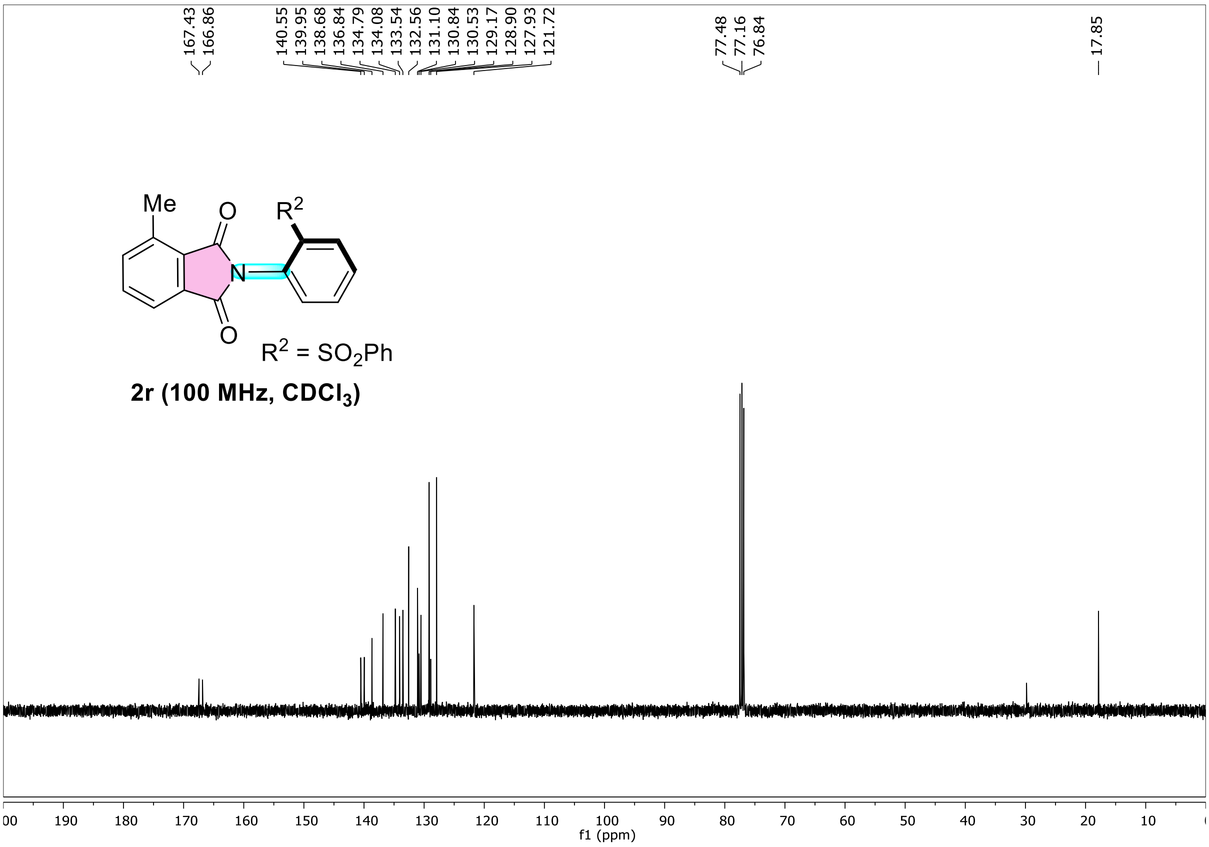

(*P*)-2-(2-(*tert*-Butyl)phenyl)-4-methoxyisoindoline-1,3-dione (2s)

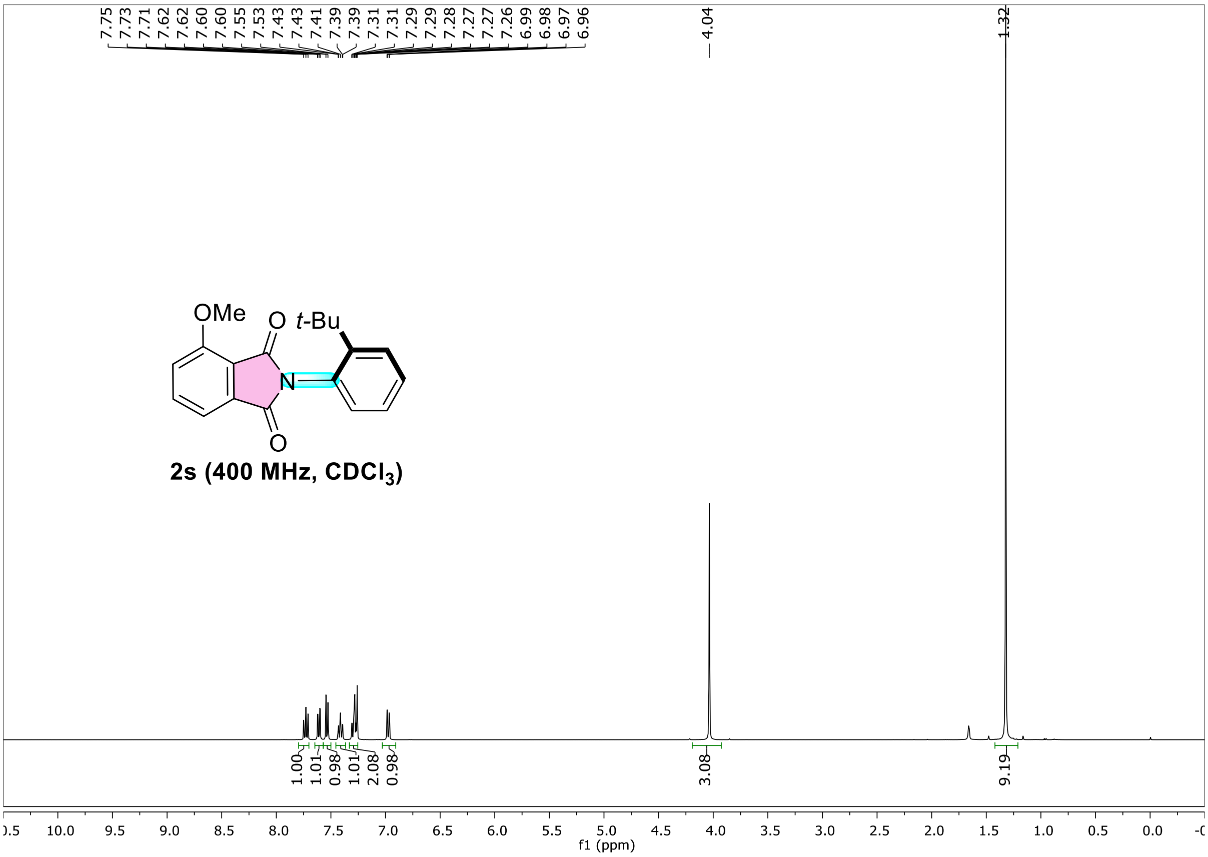

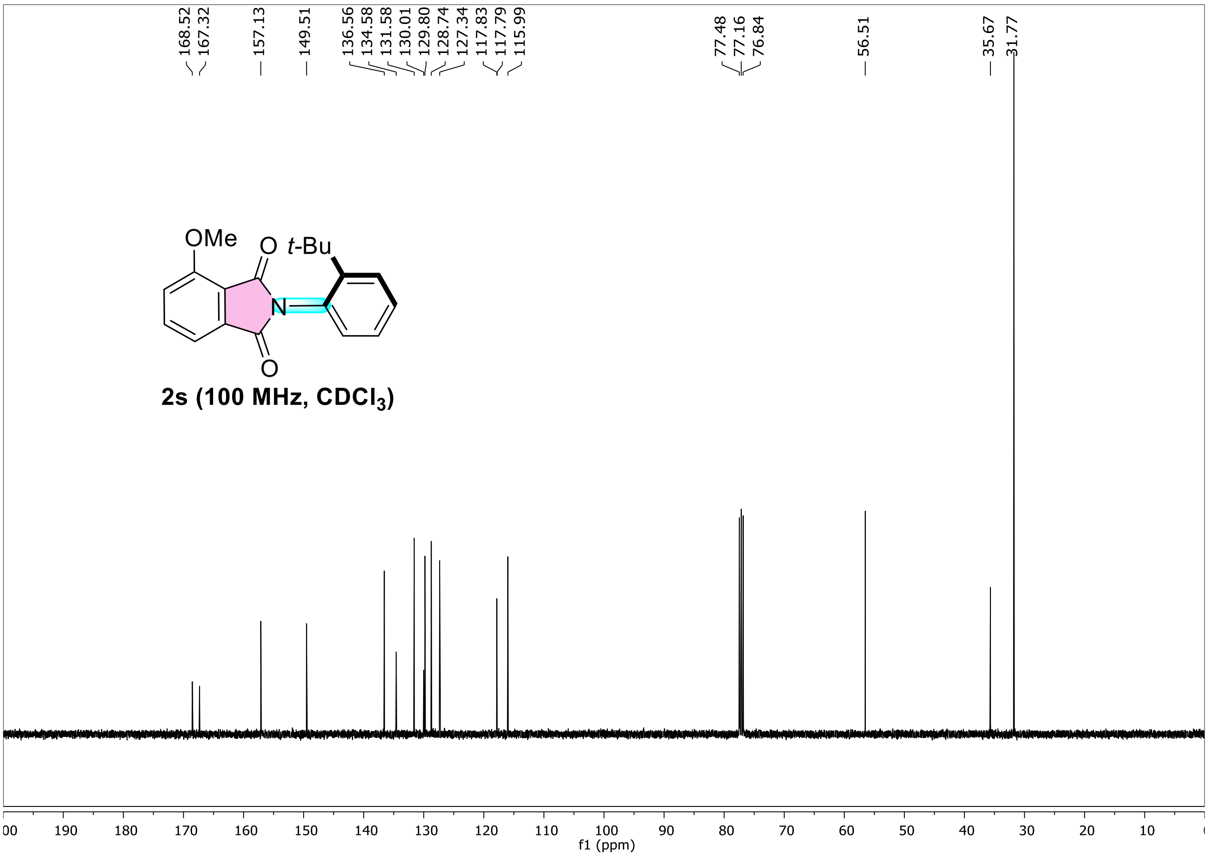

(*P*)-4-(Benzyloxy)-2-(2-(*tert*-butyl)phenyl)isoindoline-1,3-dione (2t)

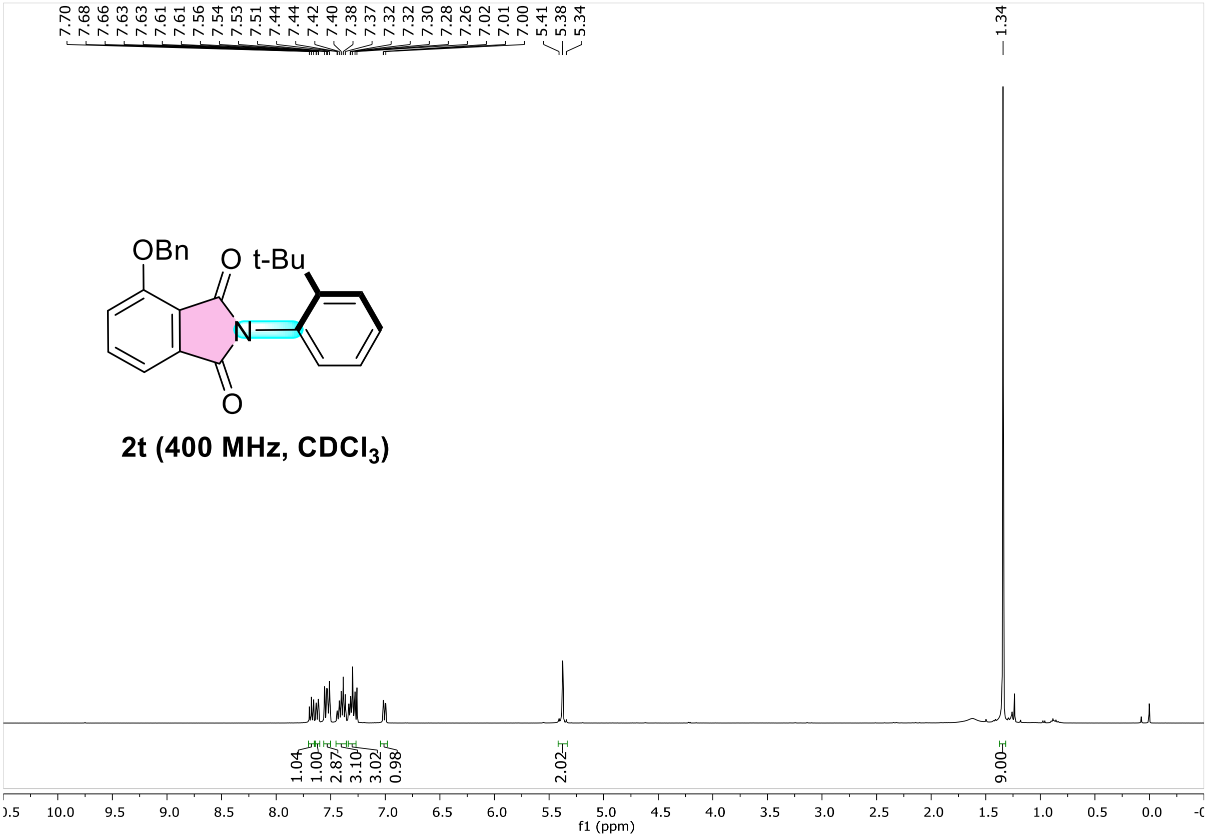

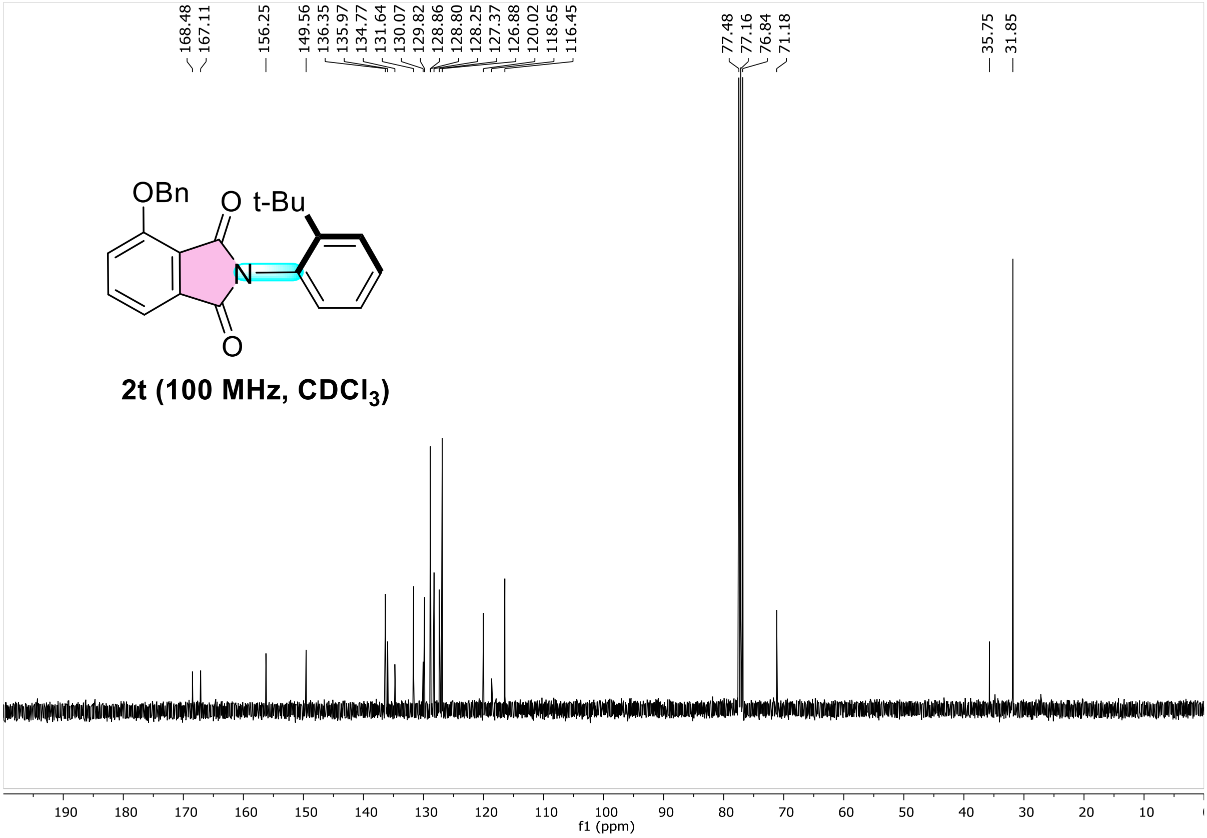

(*P*)-4-(Allyloxy)-2-(2-(*tert*-butyl)phenyl)isoindoline-1,3-dione (2u)

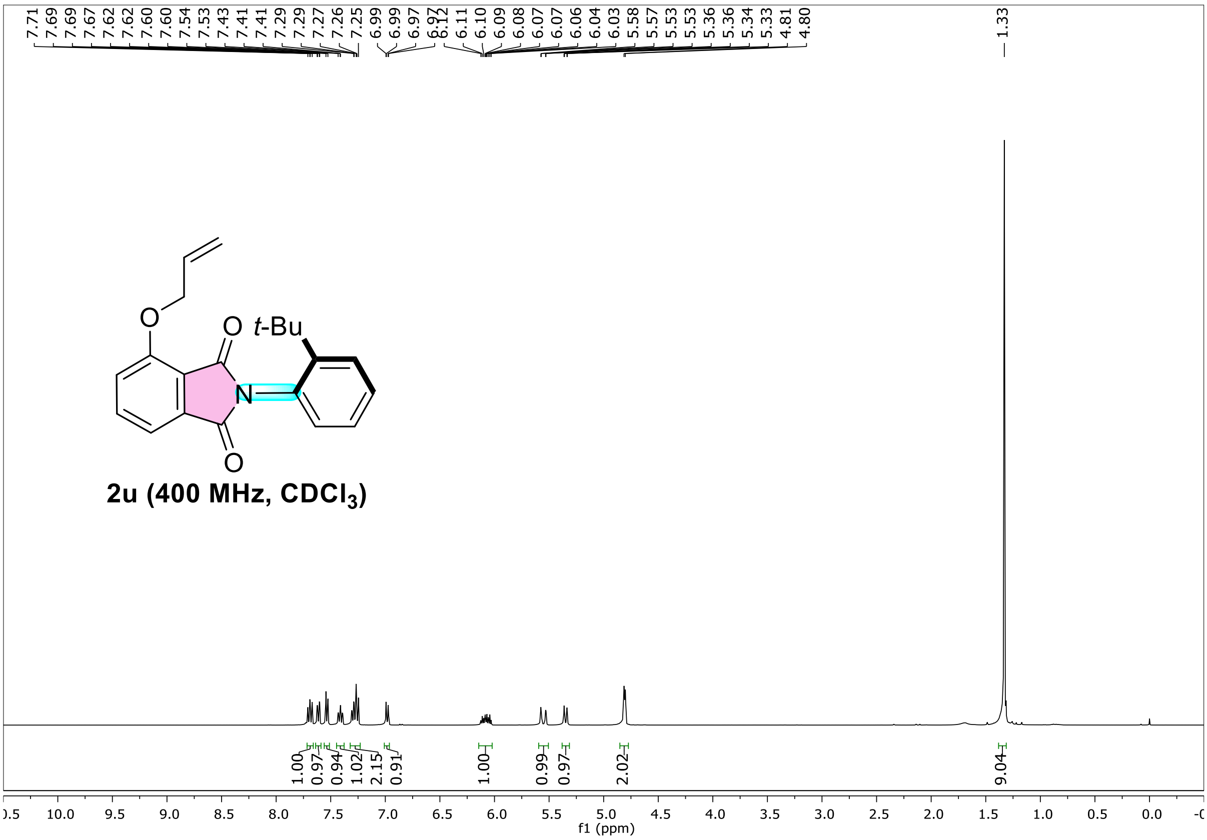

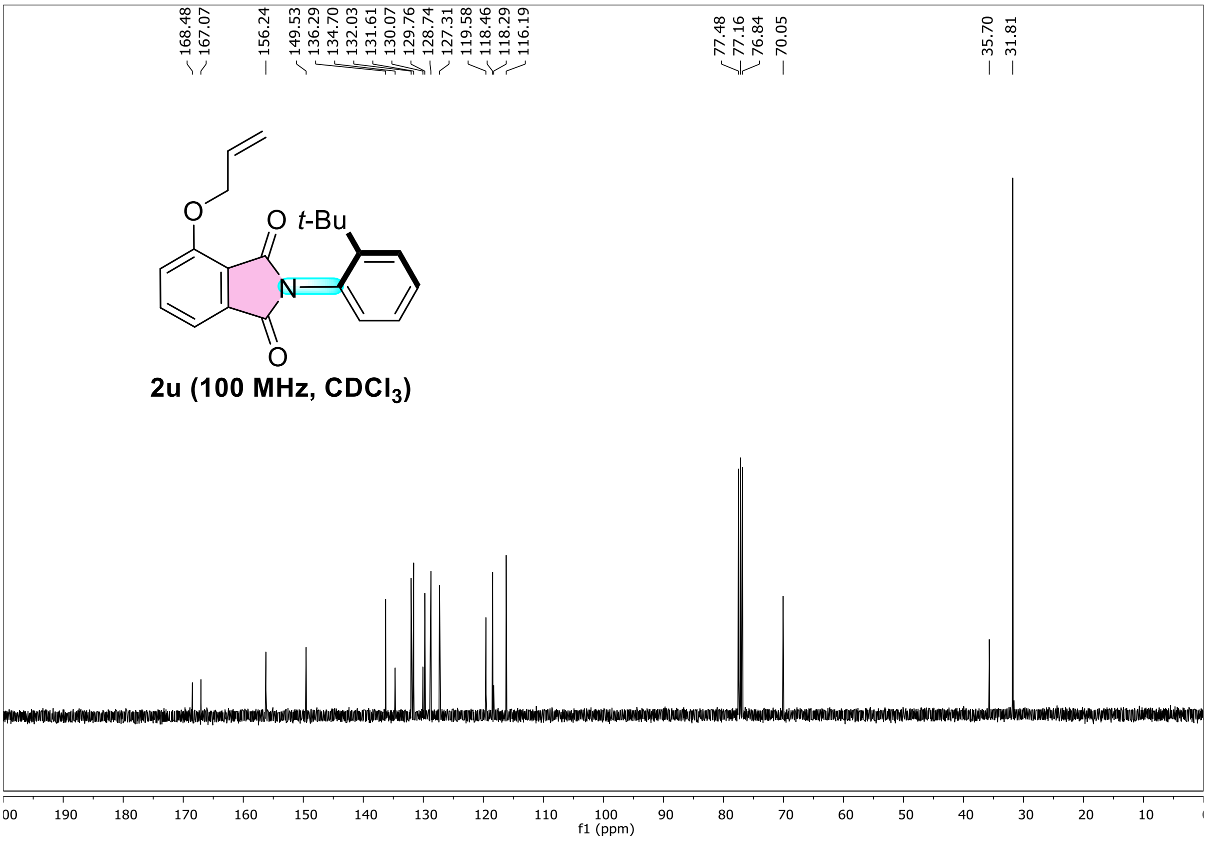

(*P*)- 2-(2-(*tert*-Butyl)phenyl)-1,3-dioxoisoindolin-4-yl pivalate (2v)

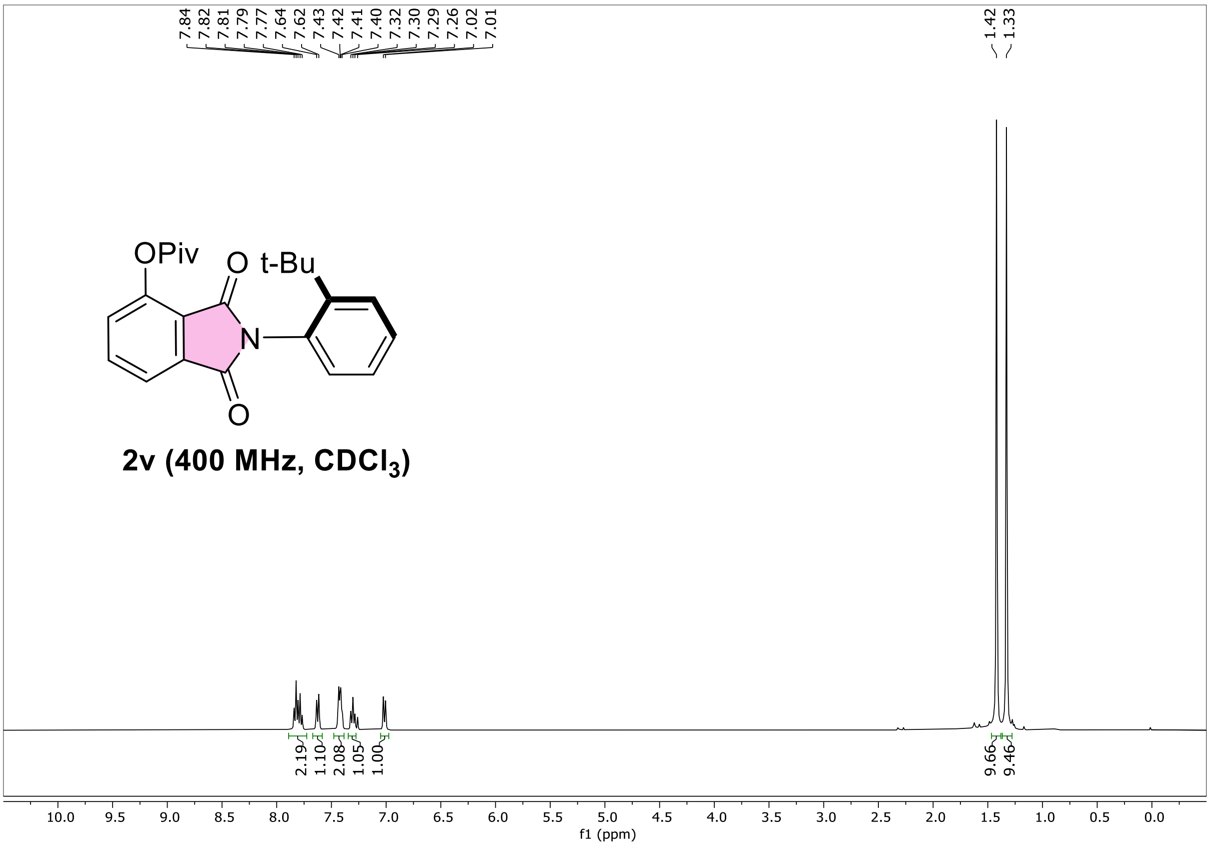

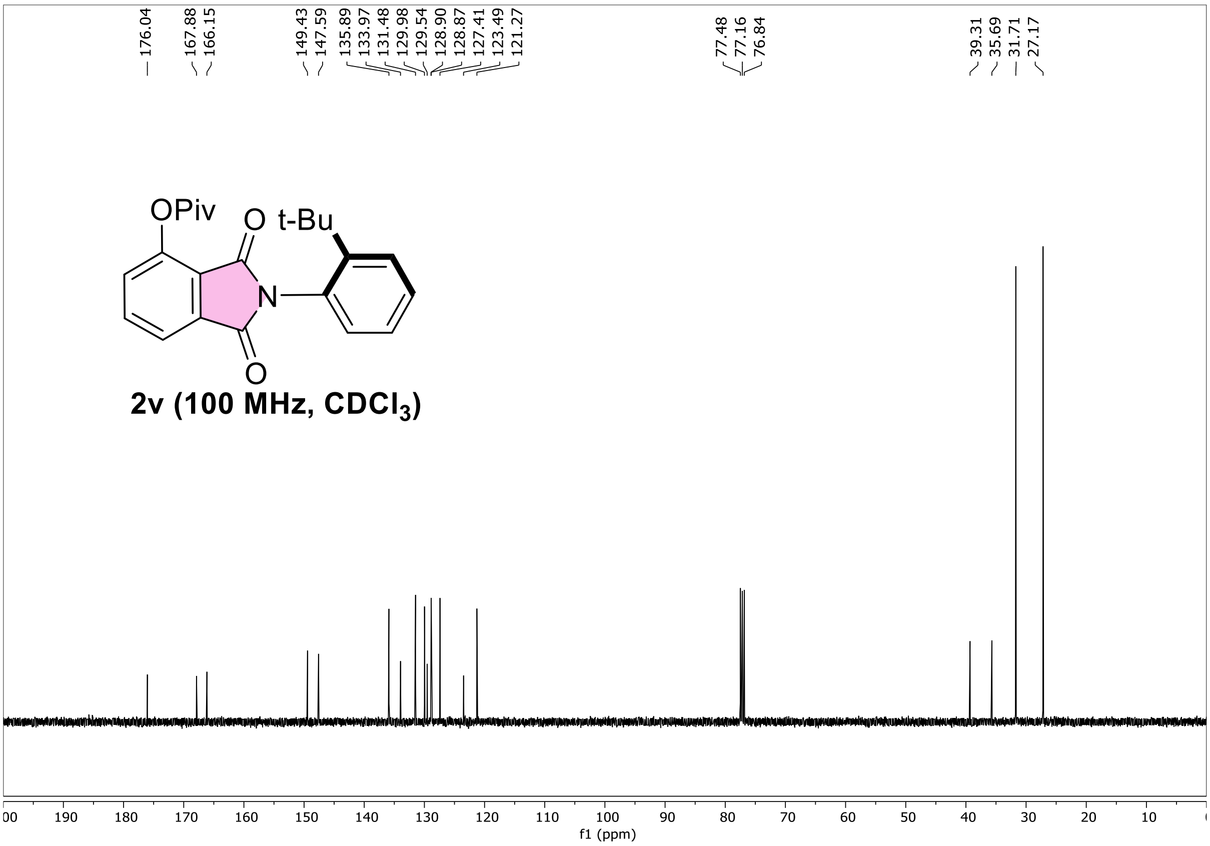

(*P*)-2-(2-(*tert*-Butyl)phenyl)-4-chloroisoindoline-1,3-dione (2w)

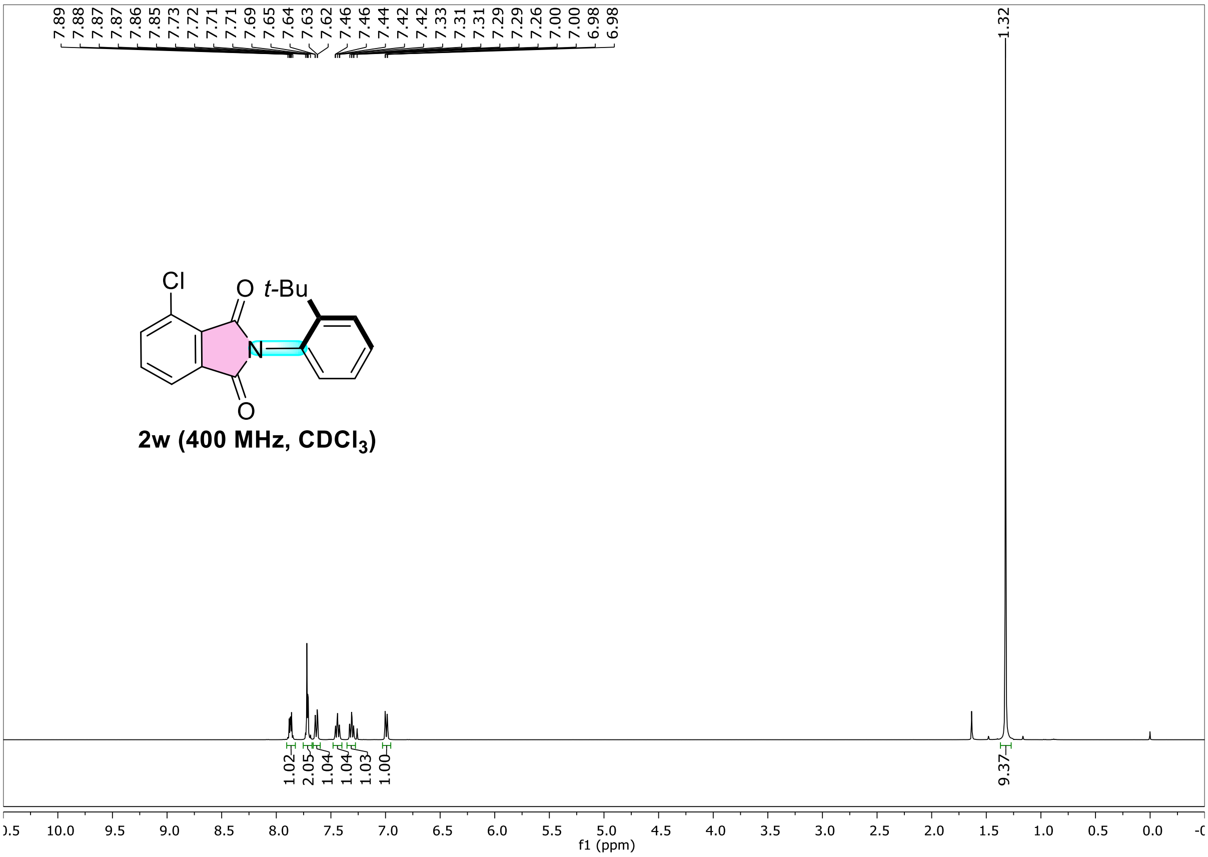

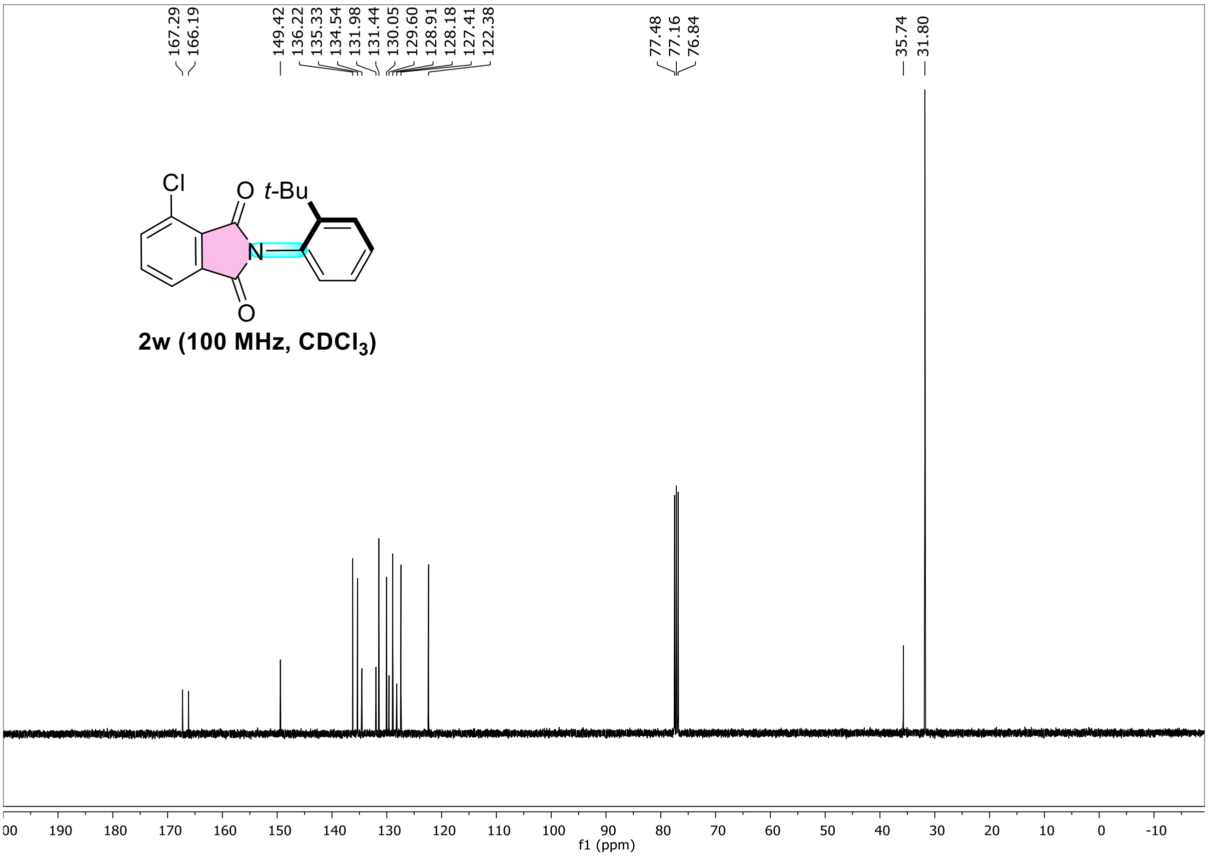

(*P*) 4-Bromo-2-(2-(*tert*-butyl)phenyl)isoindoline-1,3-dione (2x)

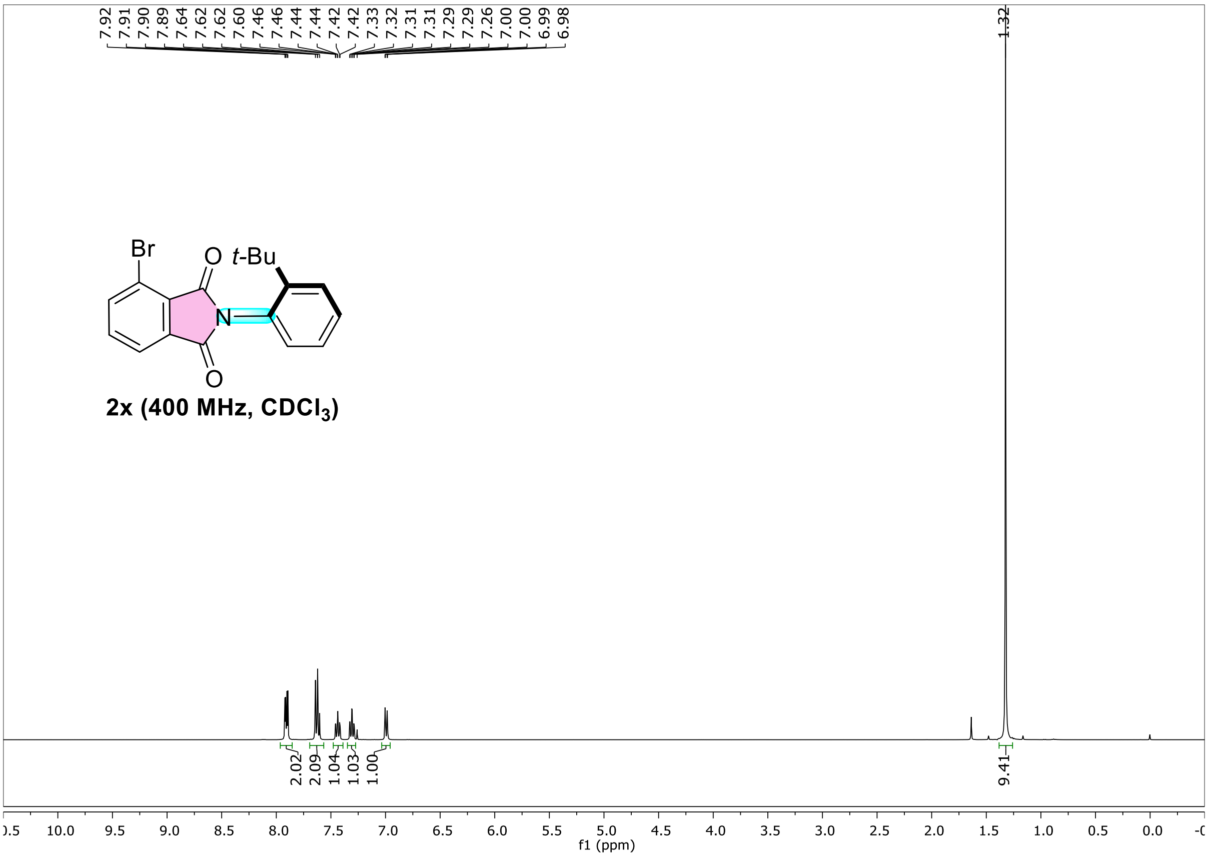

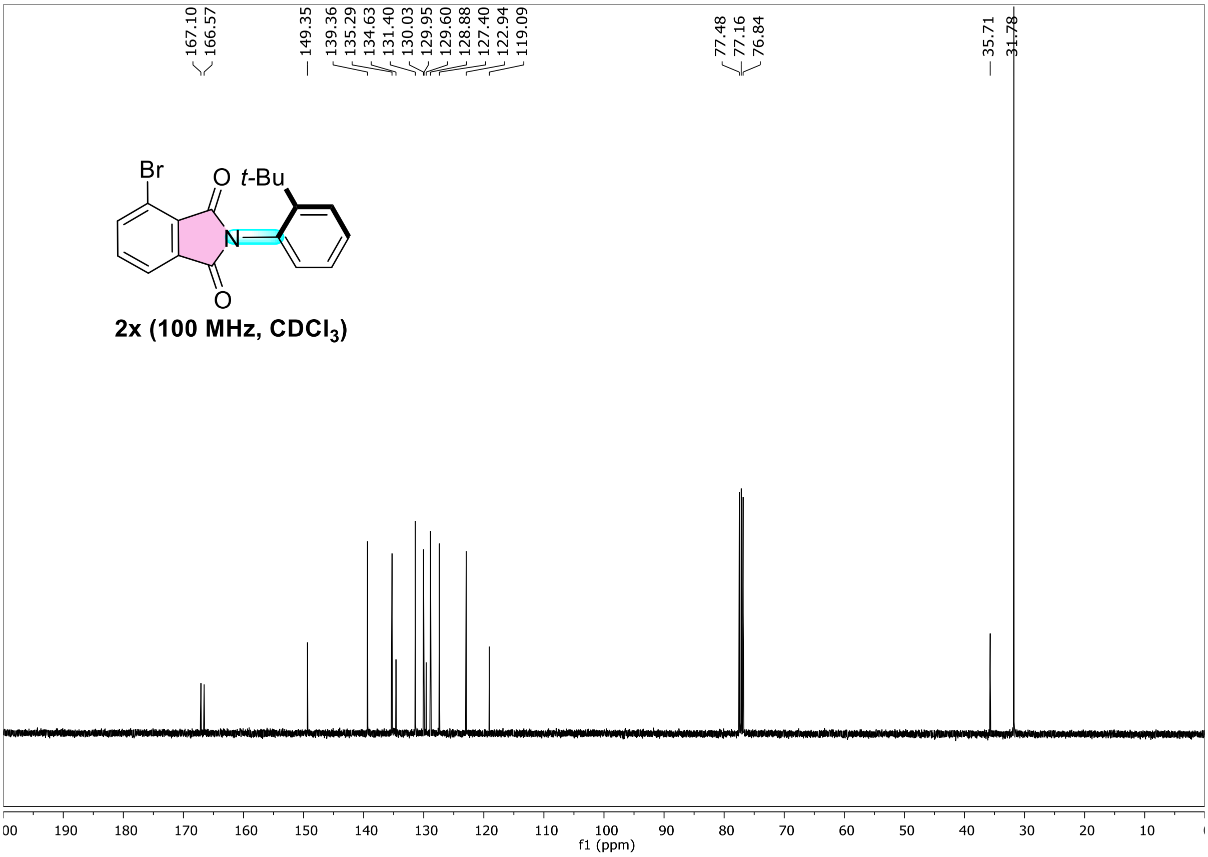

(*P*)-2-(2-(*tert*-Butyl)phenyl)-4-nitroisoindoline-1,3-dione (2y)

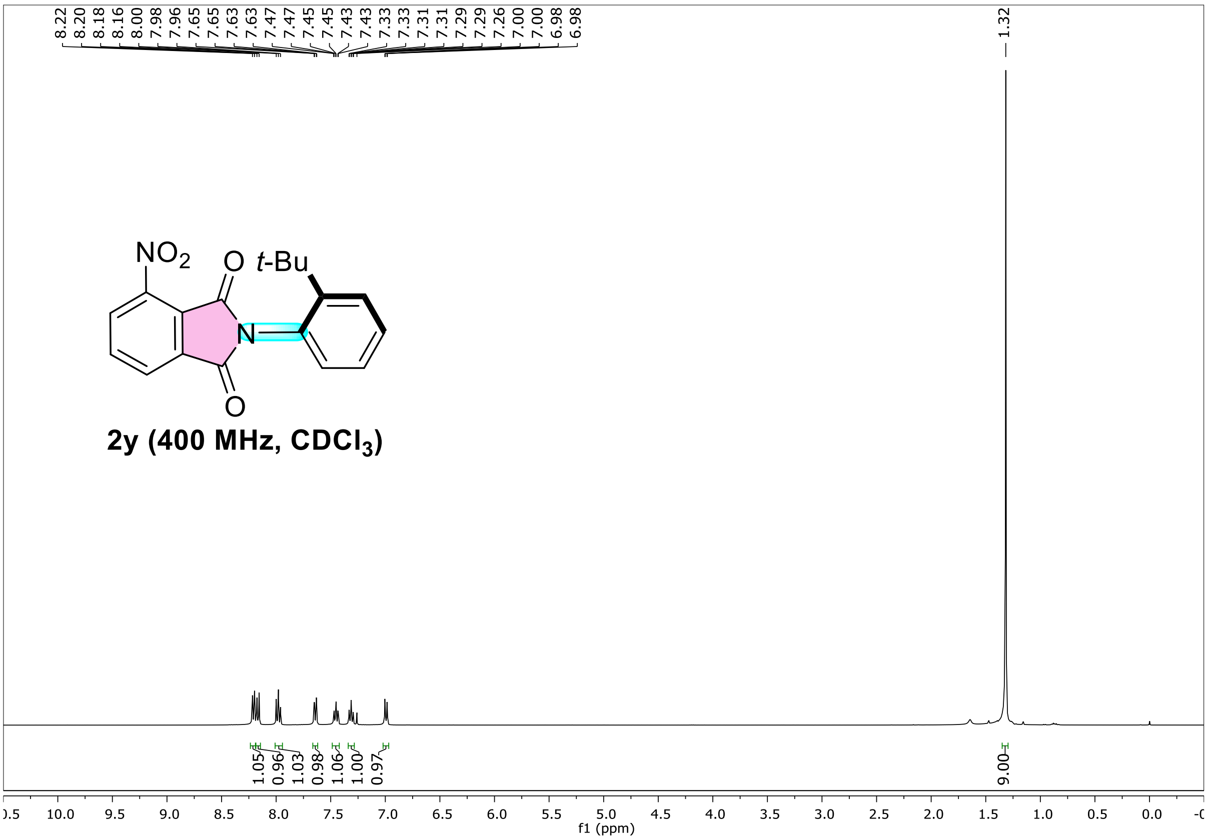

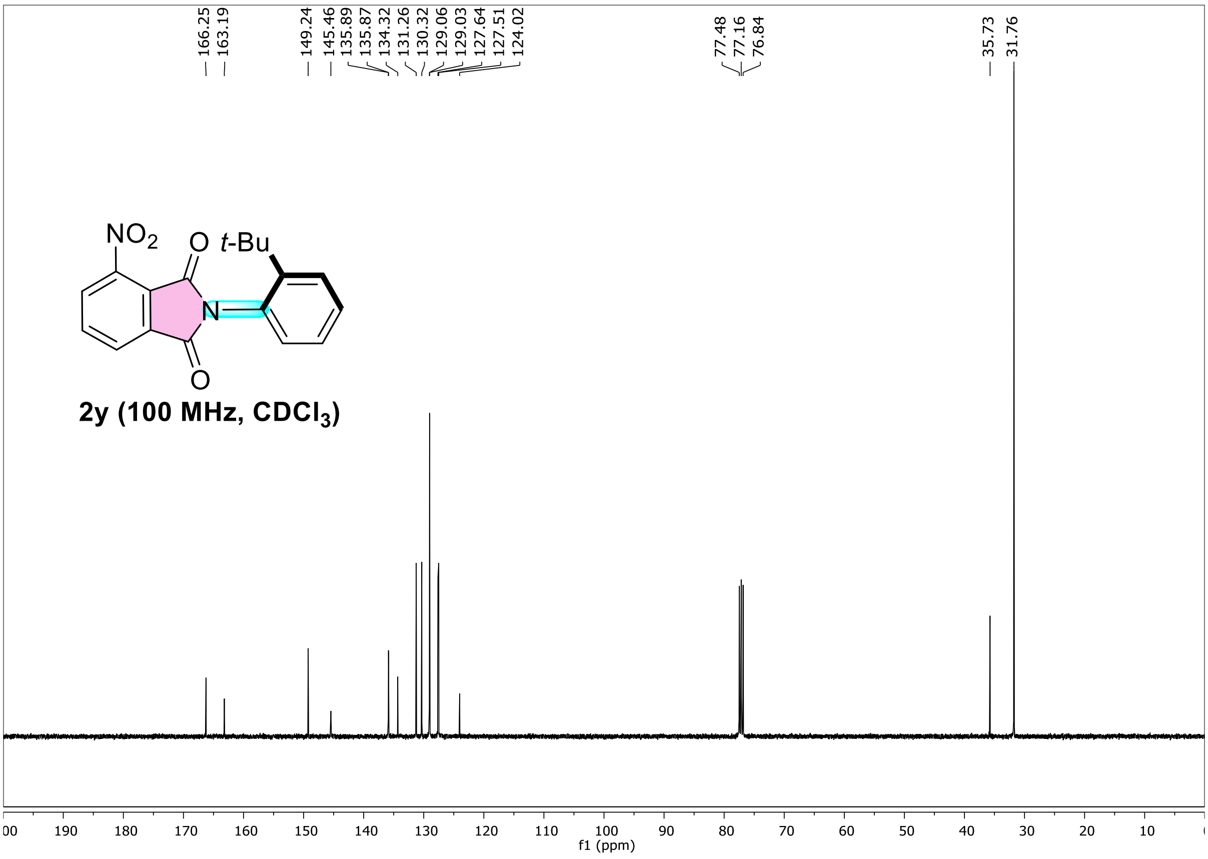

(*P*)-2-(2-(*tert*-Butyl)phenyl)-1*H*-benzo[e]isoindole-1,3(2*H*)-dione (2z)

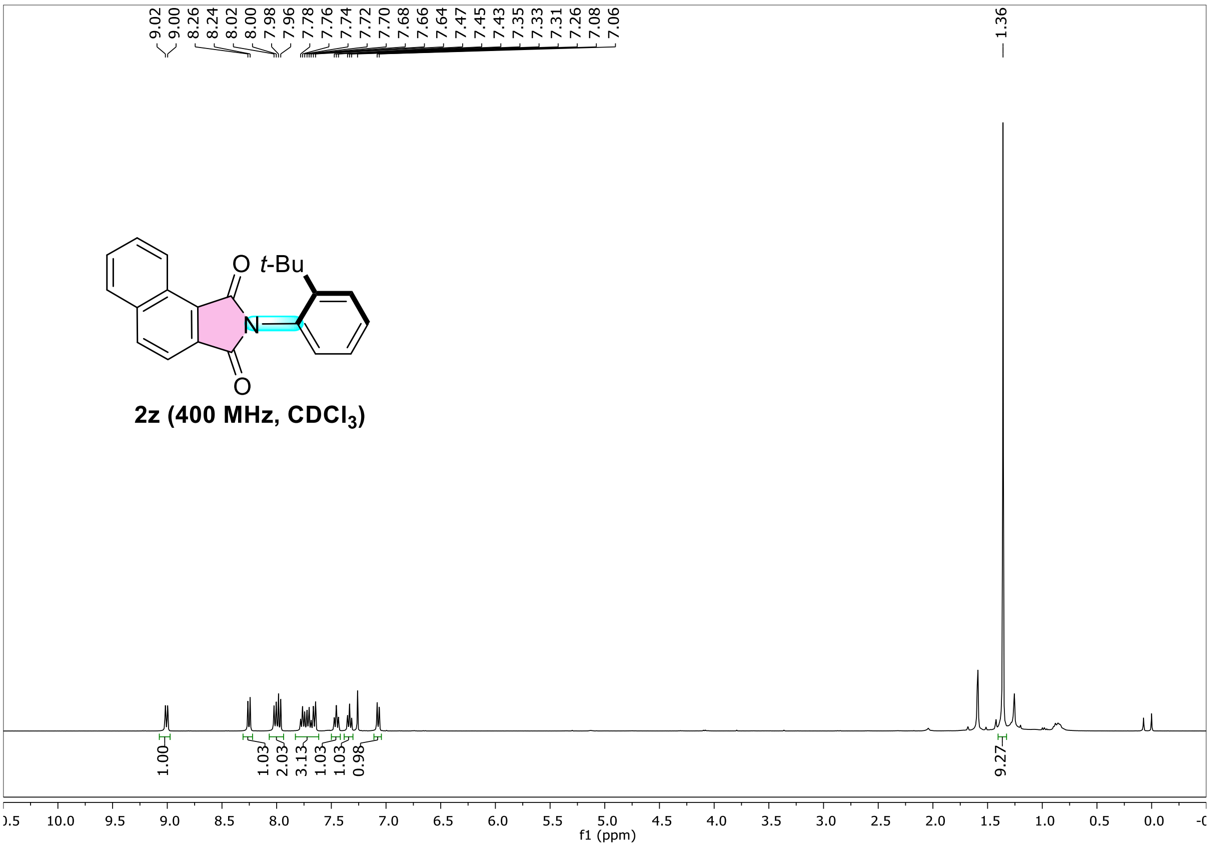

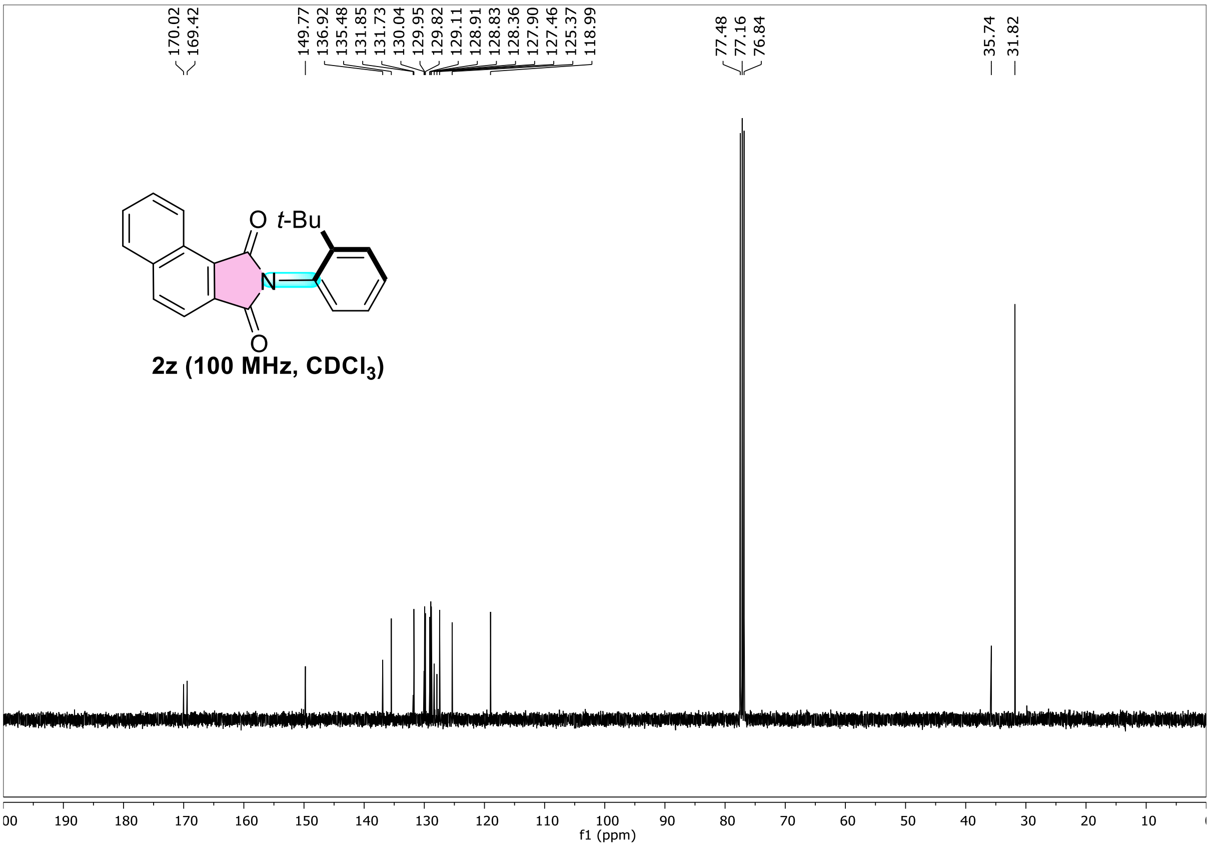

(*P*)-2-(2-(*tert*-Butyl)phenyl)-4-methoxy-5-nitroisoindoline-1,3-dione (2aa)

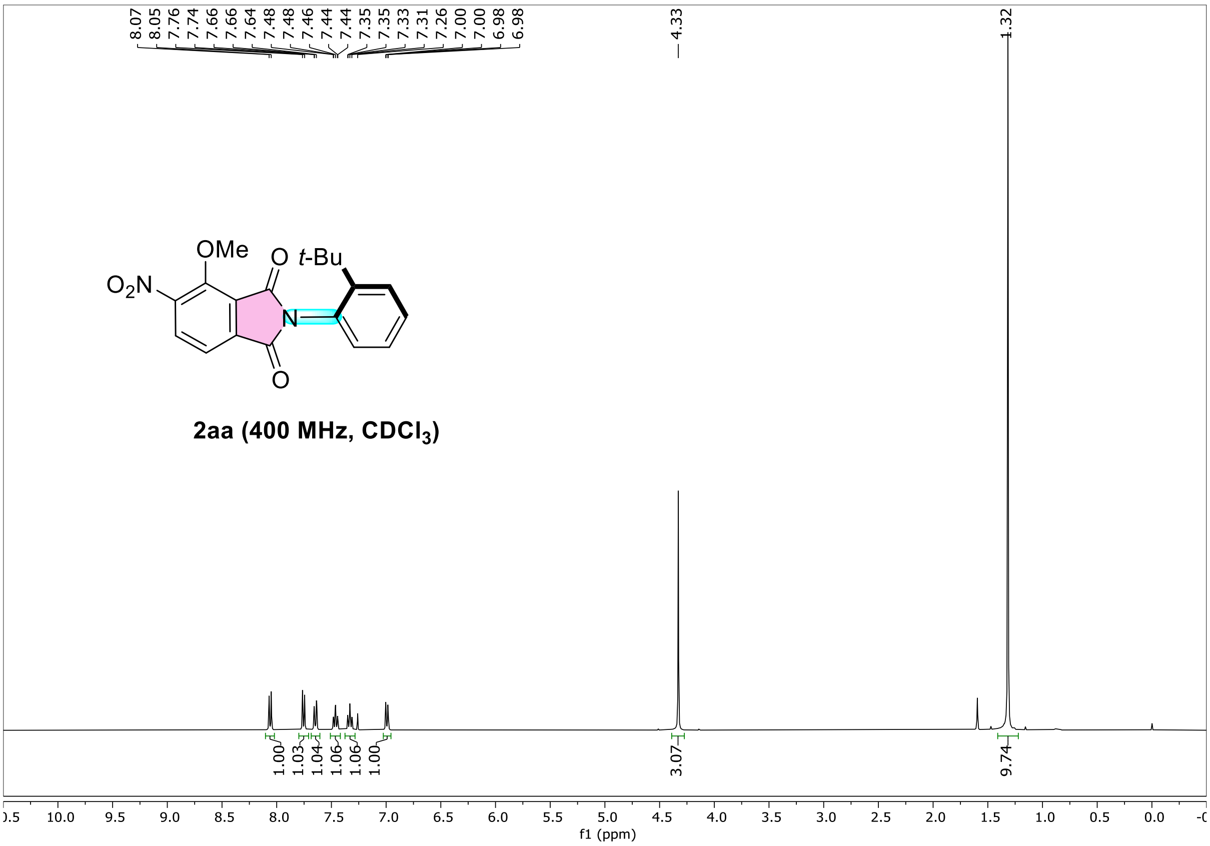

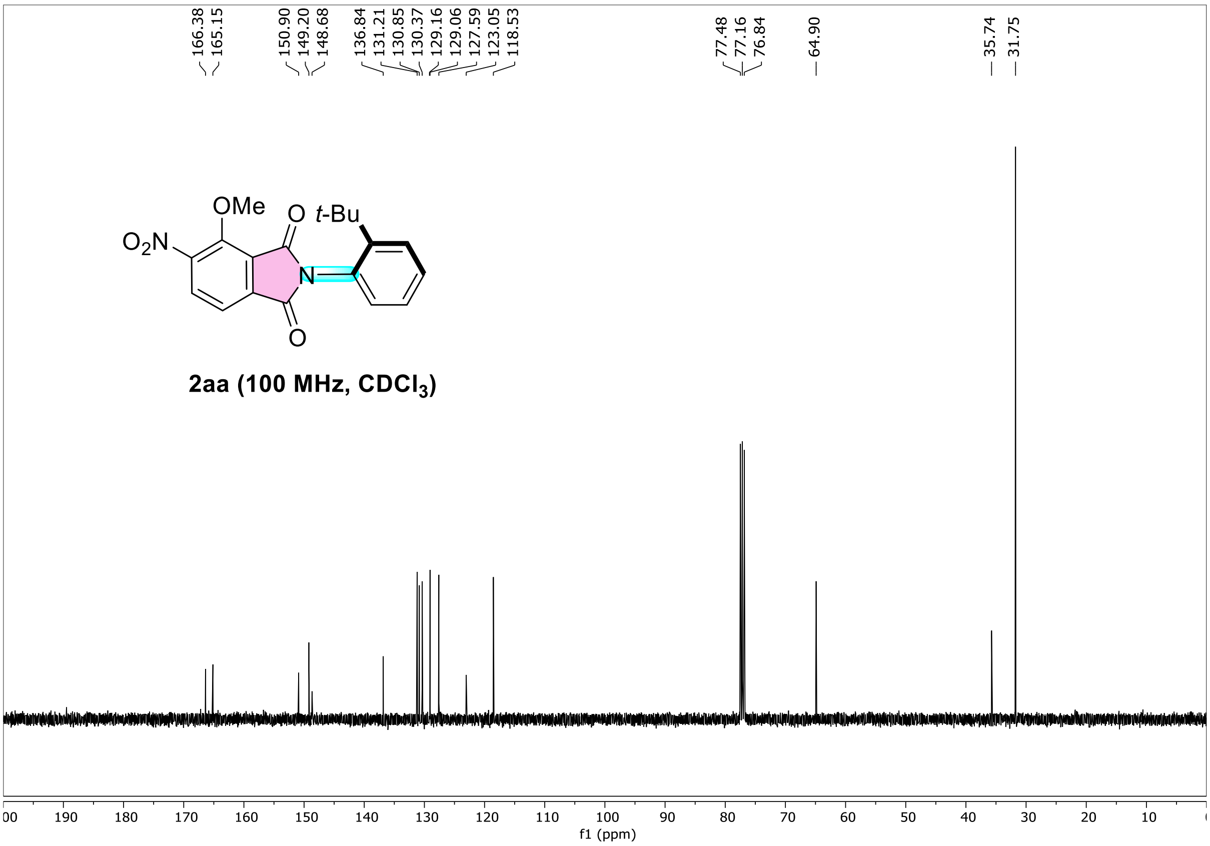

(*P*)-2-(2-(*tert*-Butyl)phenyl)-4-methoxy-7-nitroisoindoline-1,3-dione (2ab)

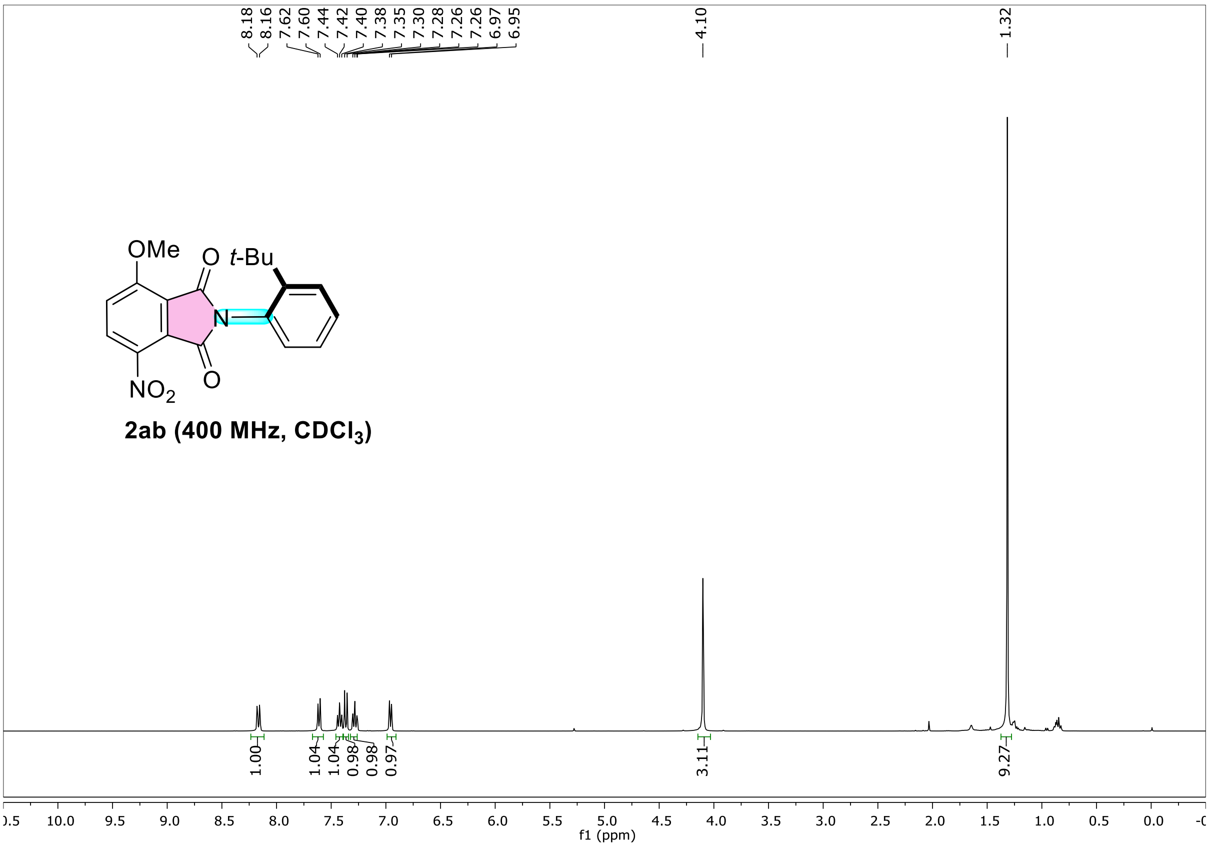

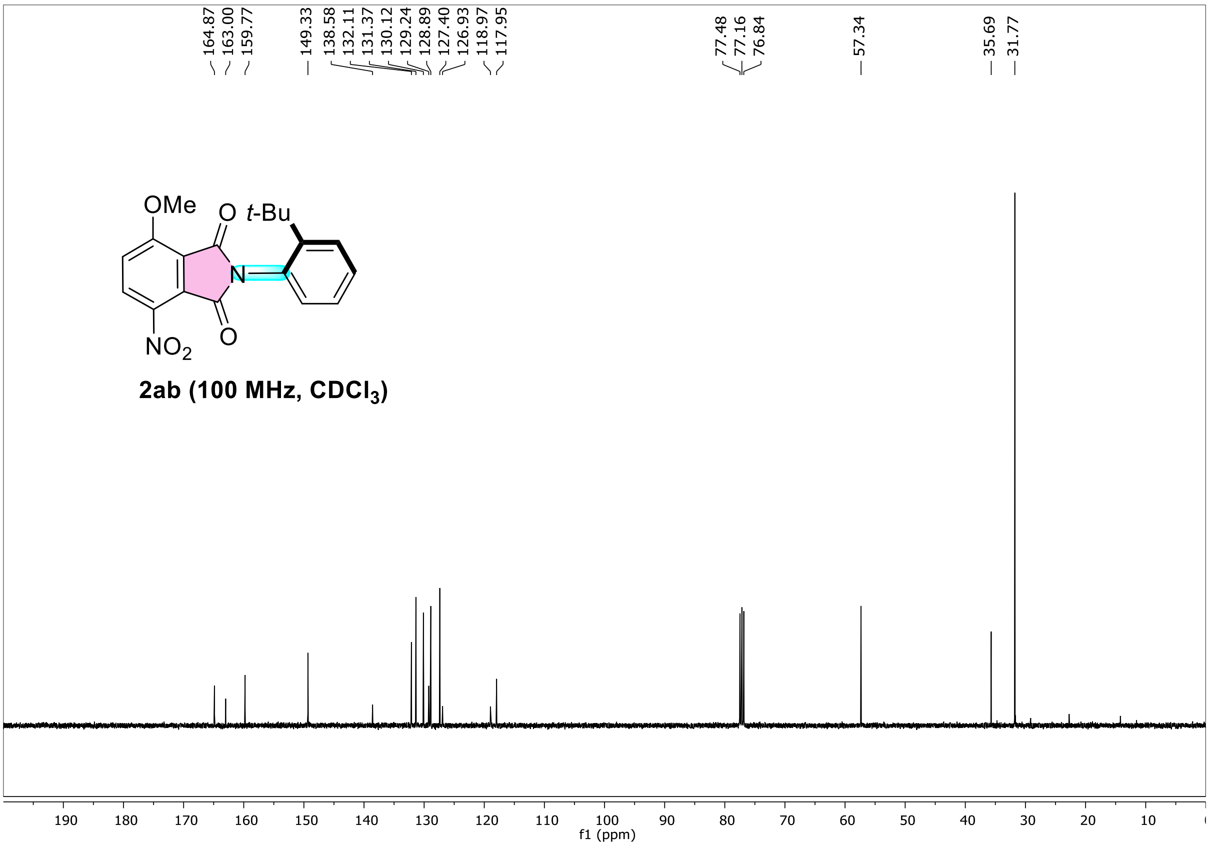

(*P*)-2-(2-(*tert*-Butyl)phenyl)-1*H*-benzo[*e*]isoindole-1,3(2*H*)-dione (2ac)

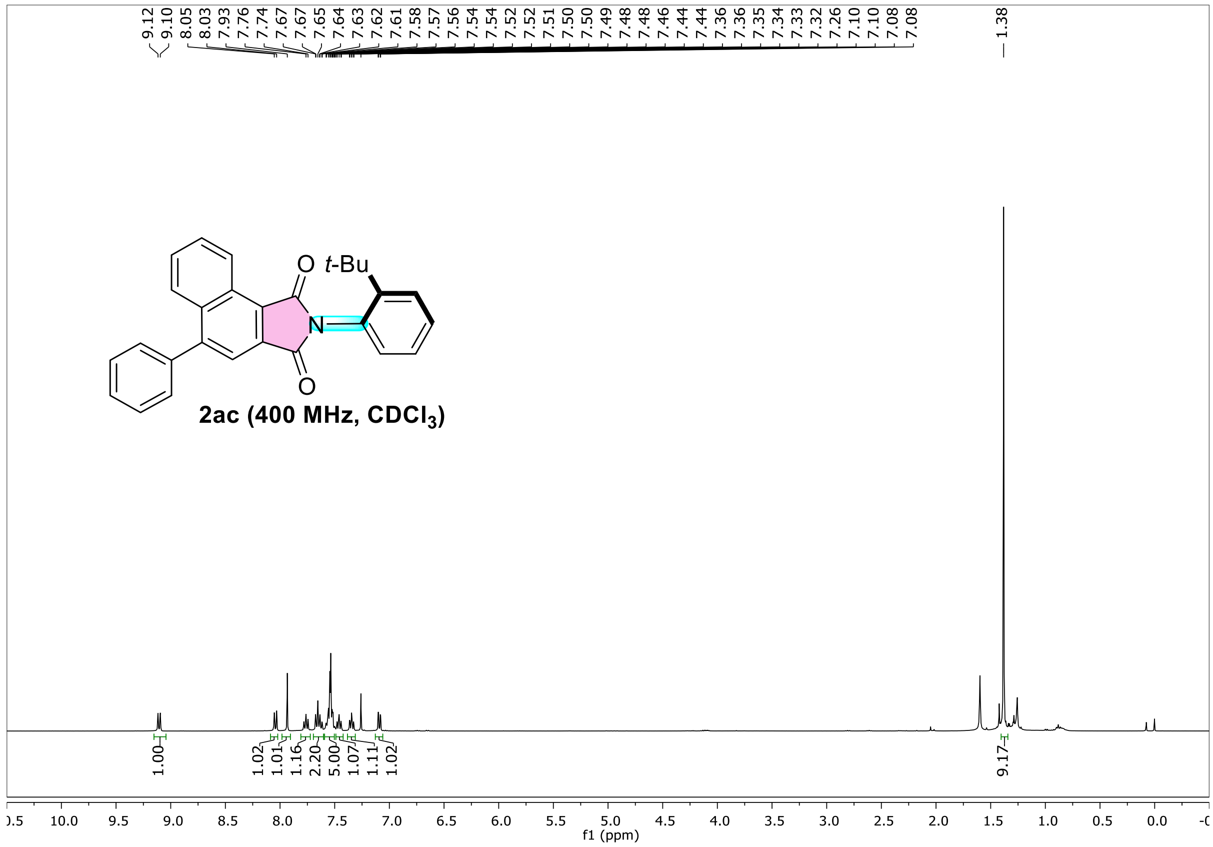

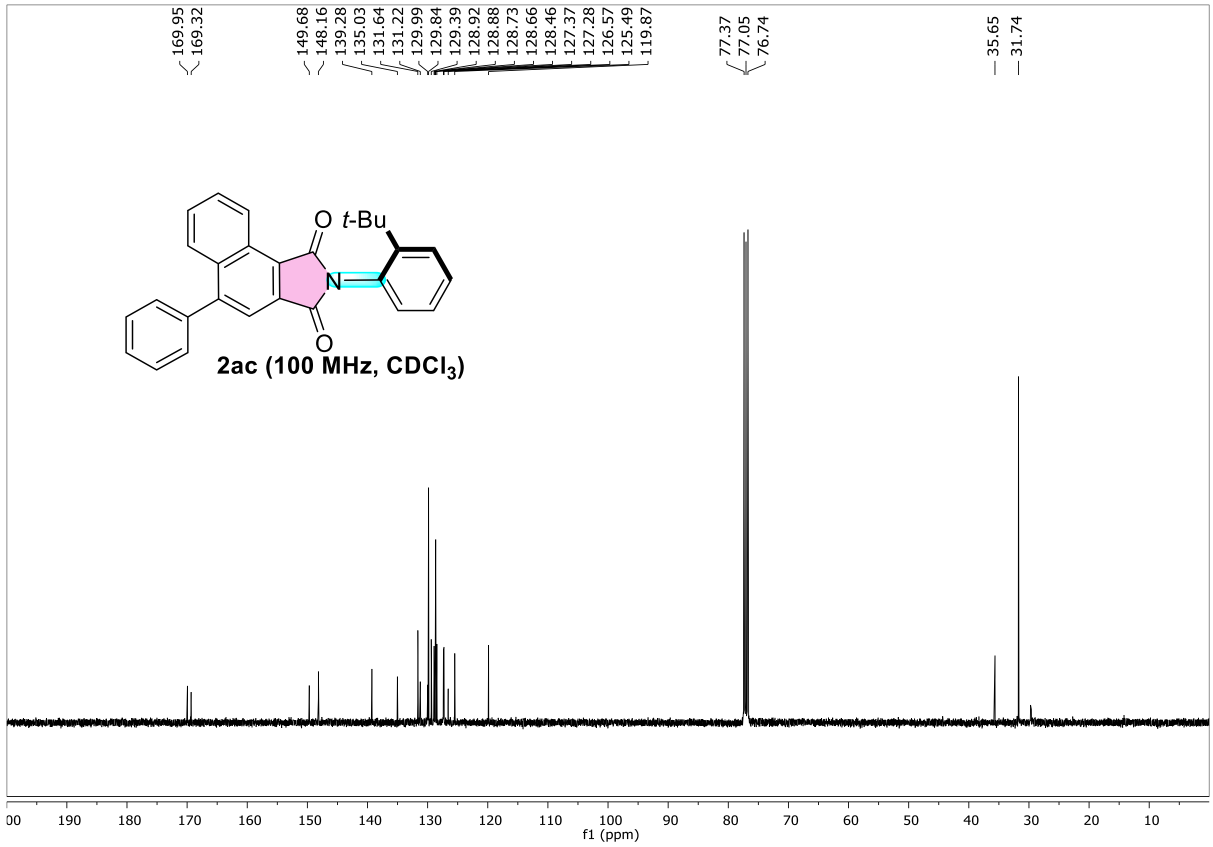

(*P*)-6-(2-(*tert*-Butyl)phenyl)-5*H*-pyrrolo[3,4-b]pyridine-5,7(6*H*)-dione (2ad)

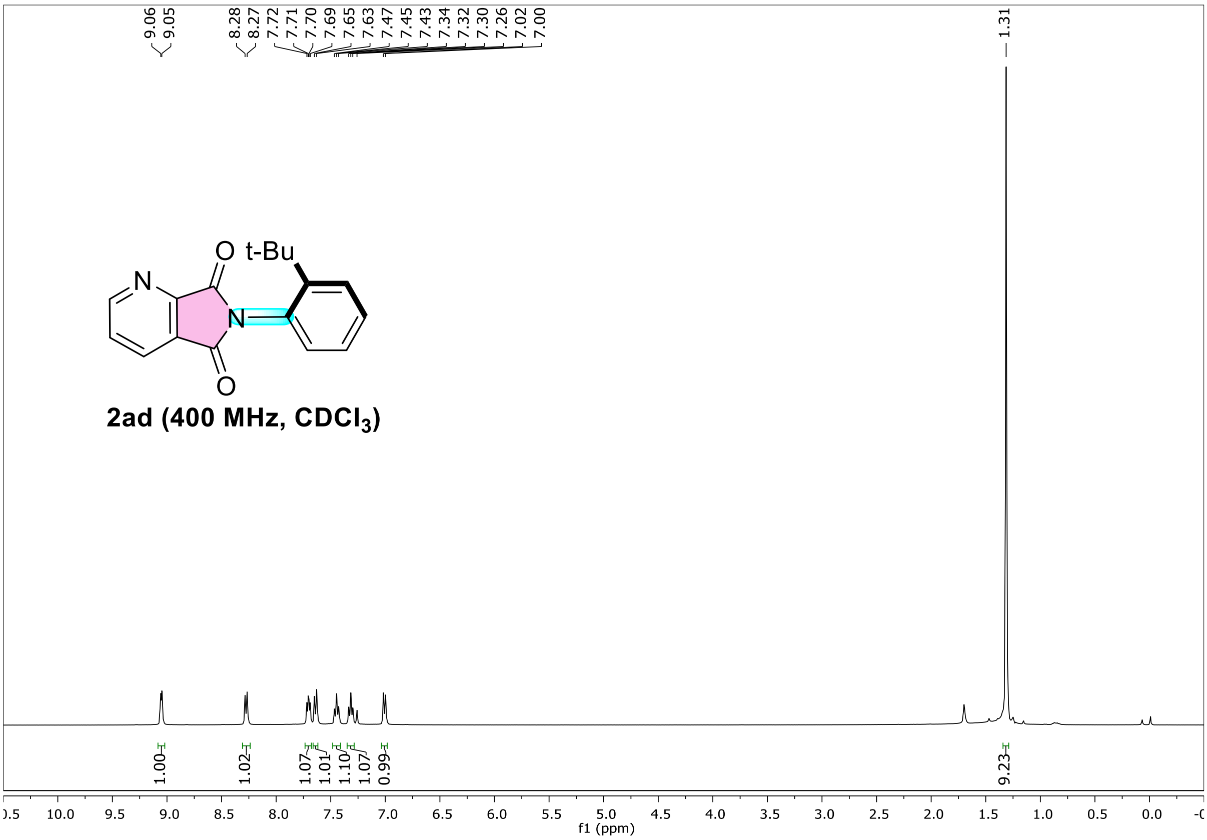

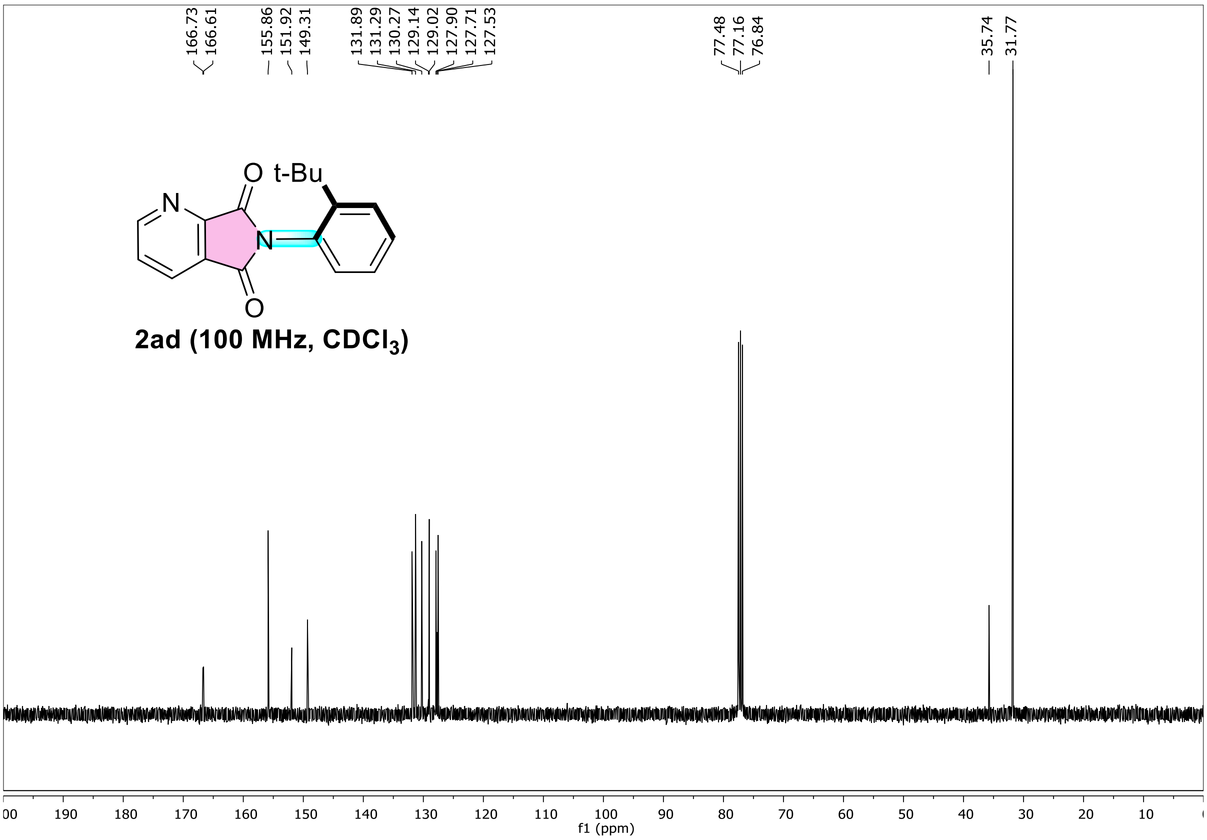

(*P*)-2-(2-(*tert*-Butyl)phenyl)-4-methylisoindoline-1,3-dione (9a)

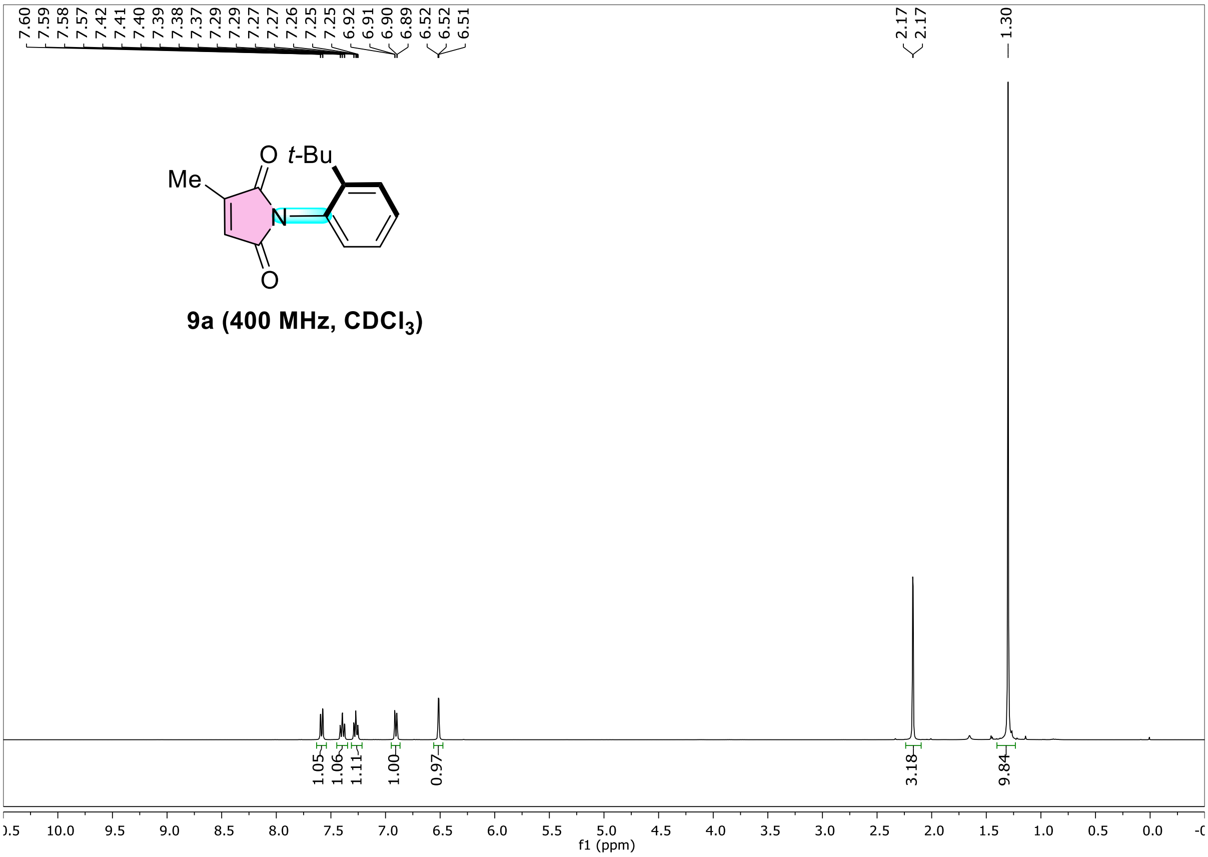

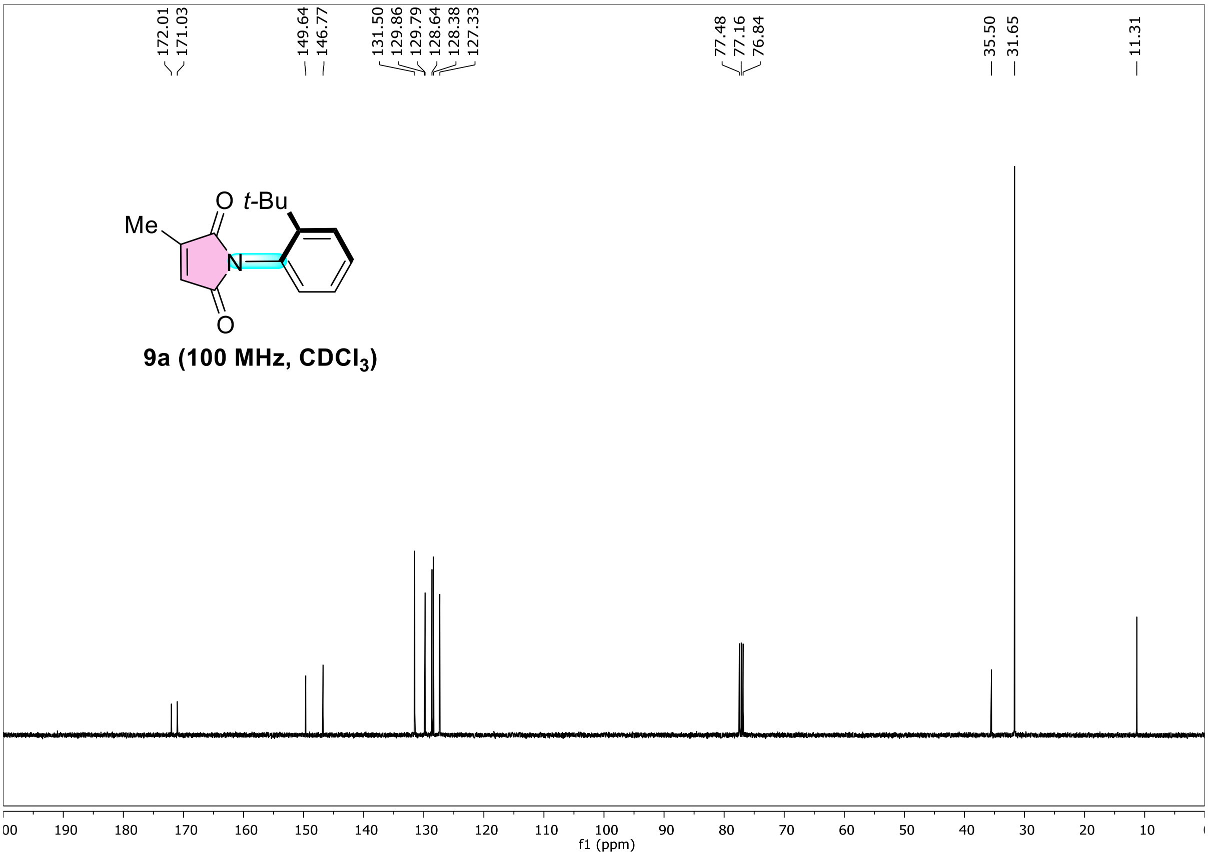

(*P*)-1-(4-Bromo-2-(*tert*-butyl)phenyl)-3-methyl-1*H*-pyrrole-2,5-dione (9b)

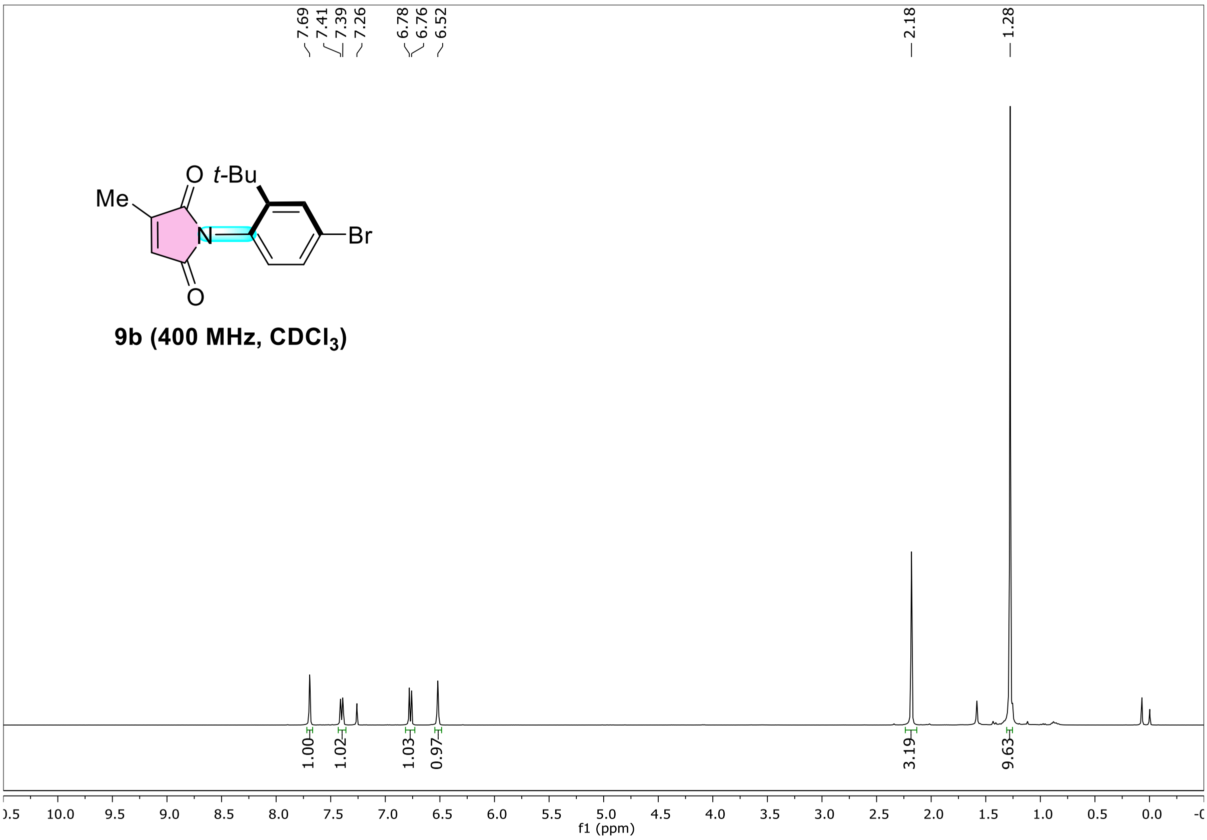

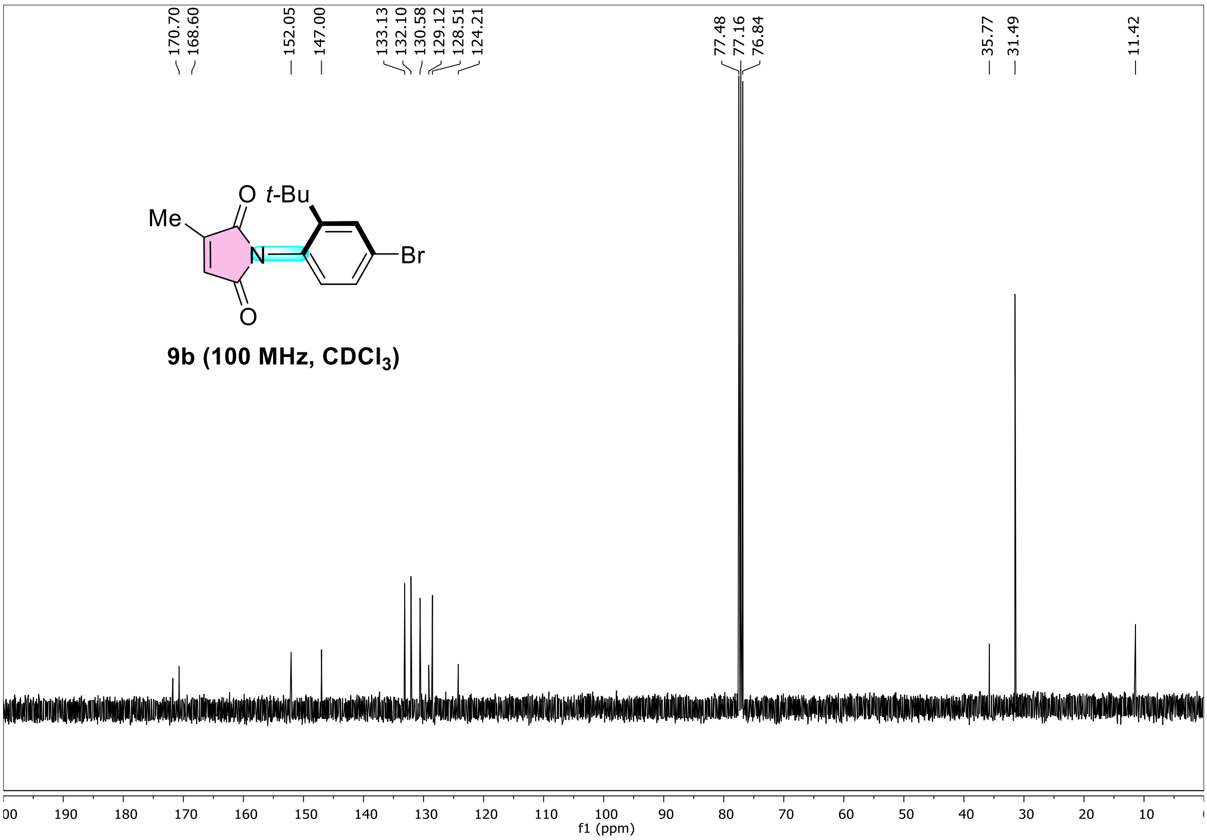

(*P*)-1-(2-(*tert*-Butyl)-4-iodophenyl)-3-methyl-1*H*-pyrrole-2,5-dione (9c)

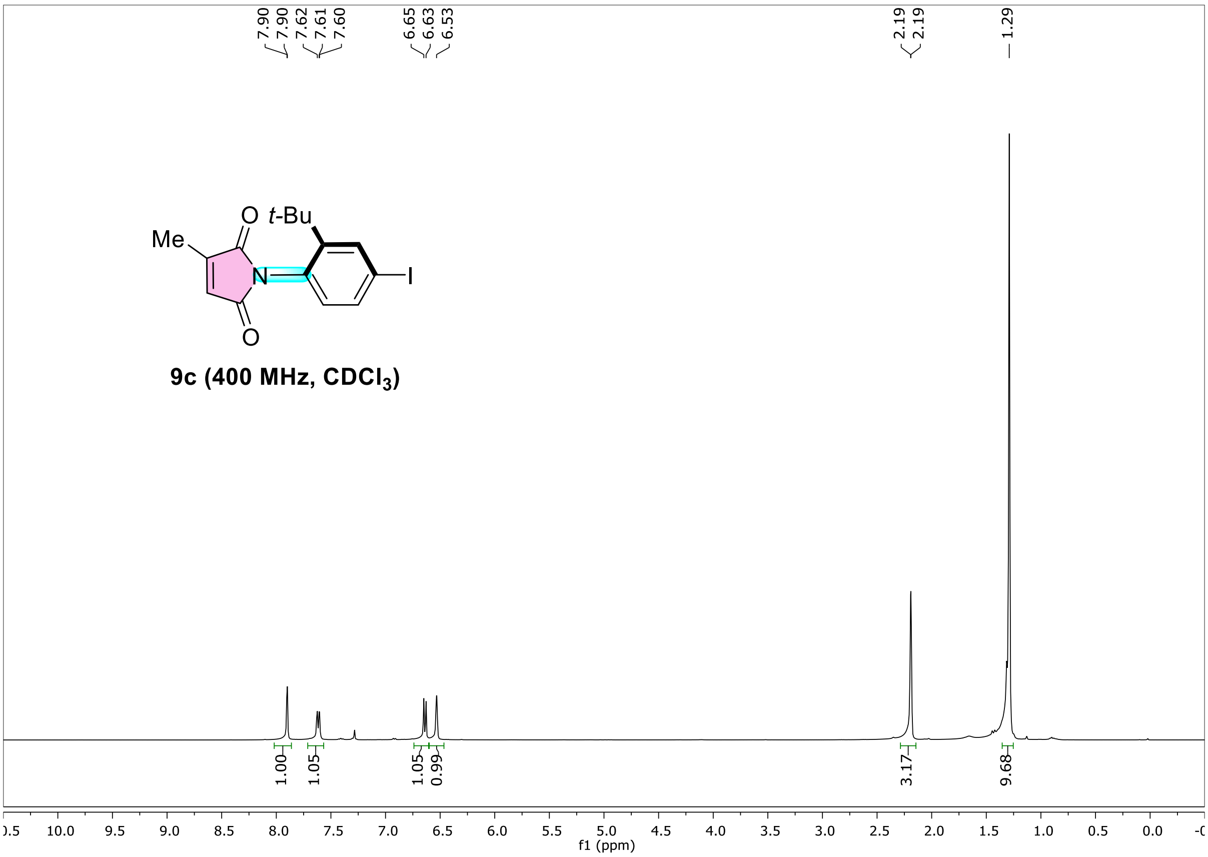

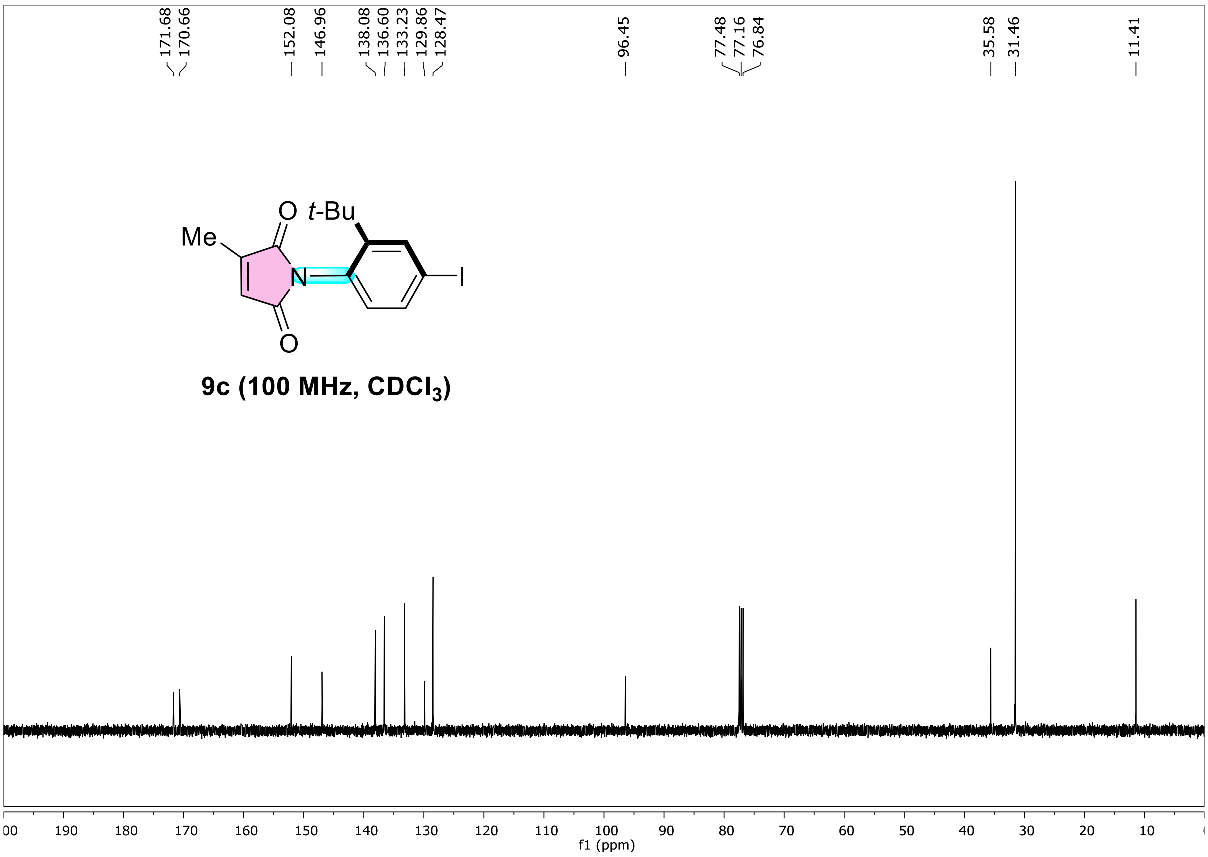

(*P*)-Ethyl-3-(*tert*-butyl)-4-(3-methyl-2,5-dioxo-2,5-dihydro-1*H*-pyrrol-1-yl)benzoate (9d)

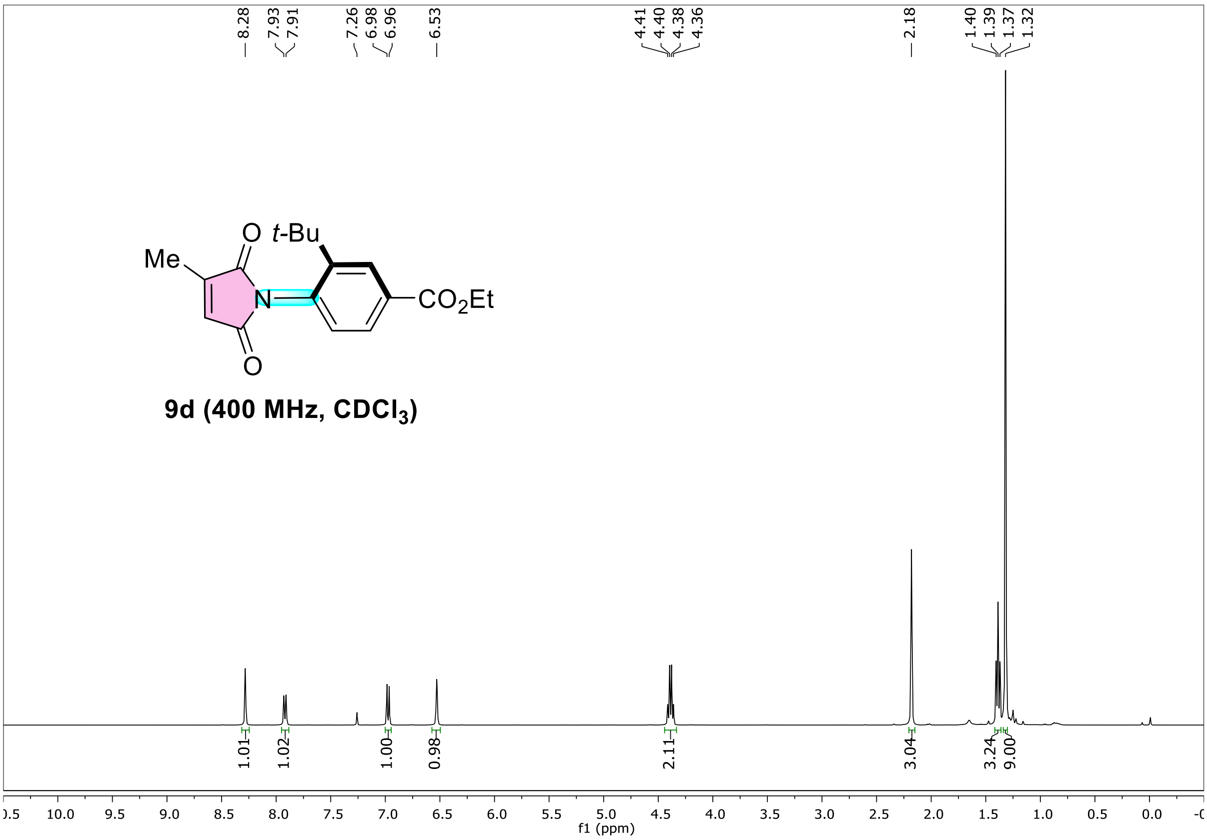

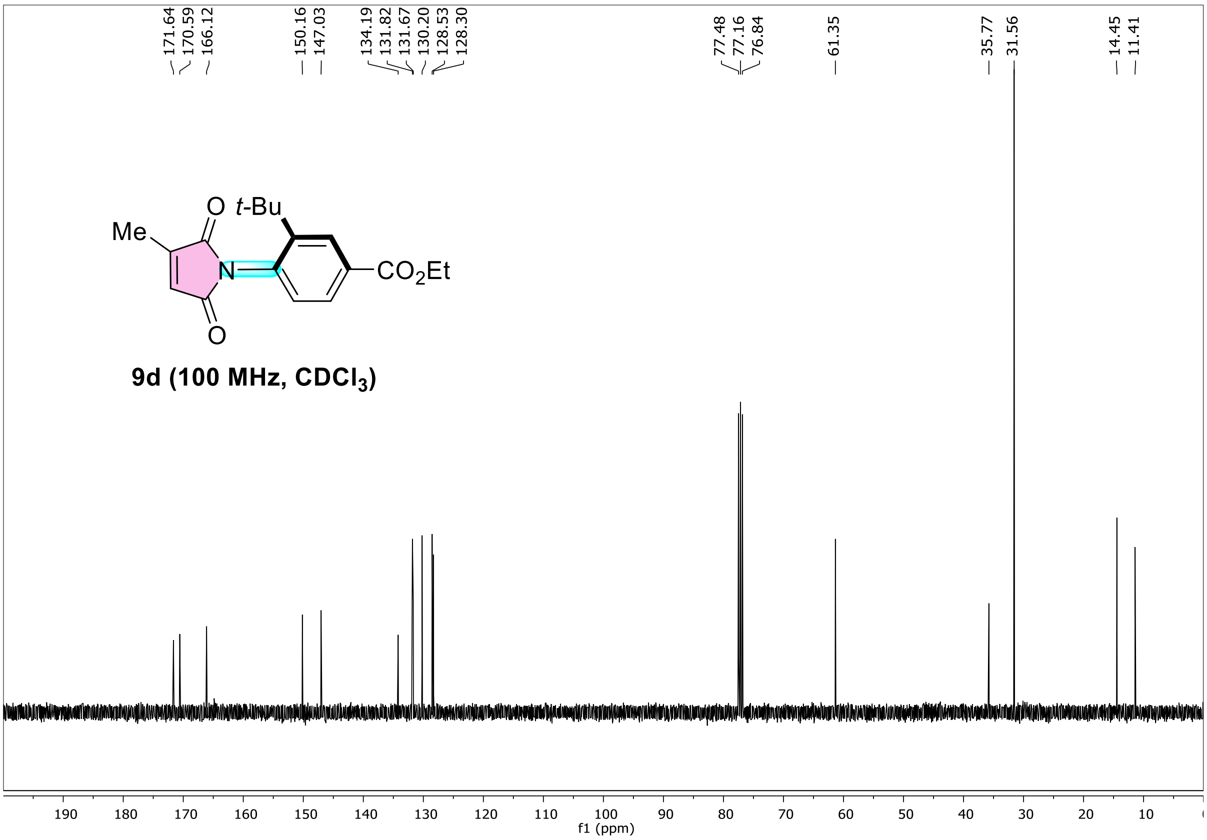

(*P*)-3-Methyl-1-(4-methyl-2-(2-phenylpropan-2-yl)phenyl)-1*H*-pyrrole-2,5-dione (9e)

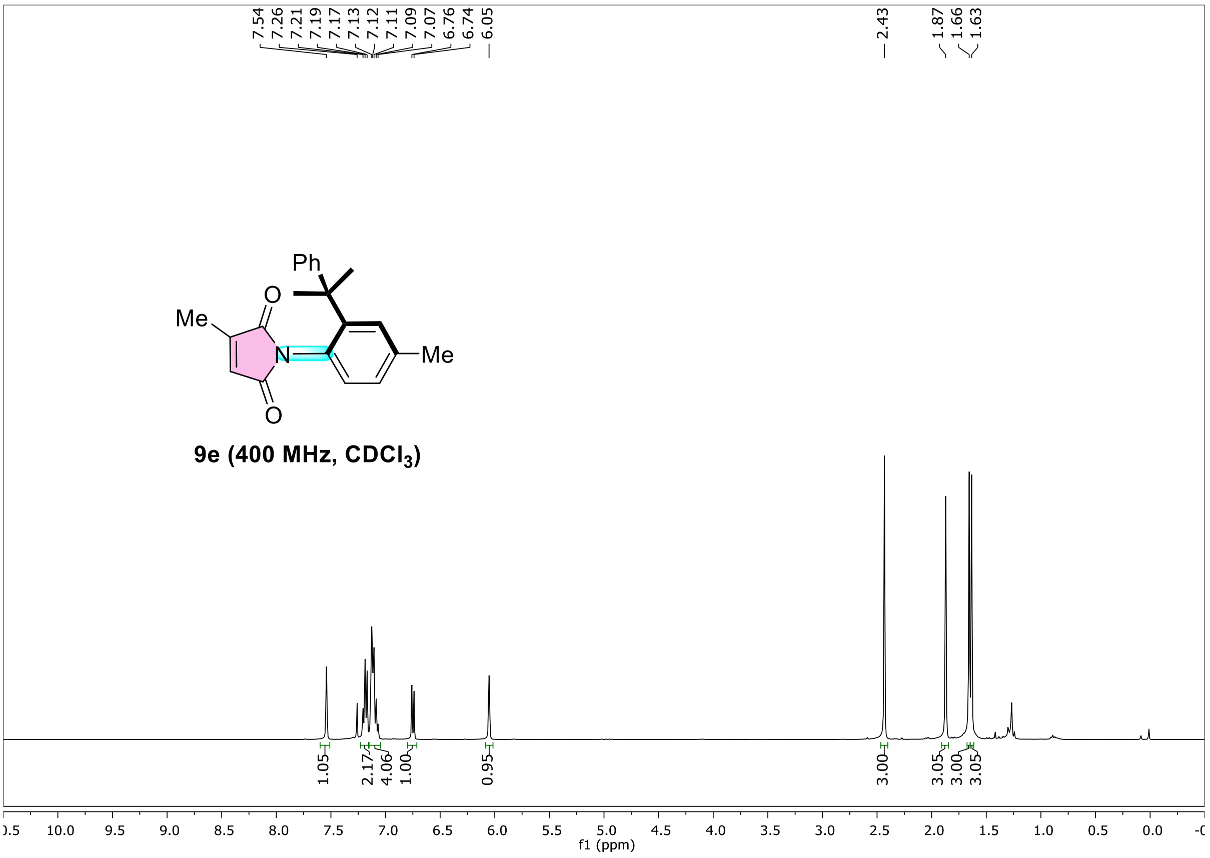

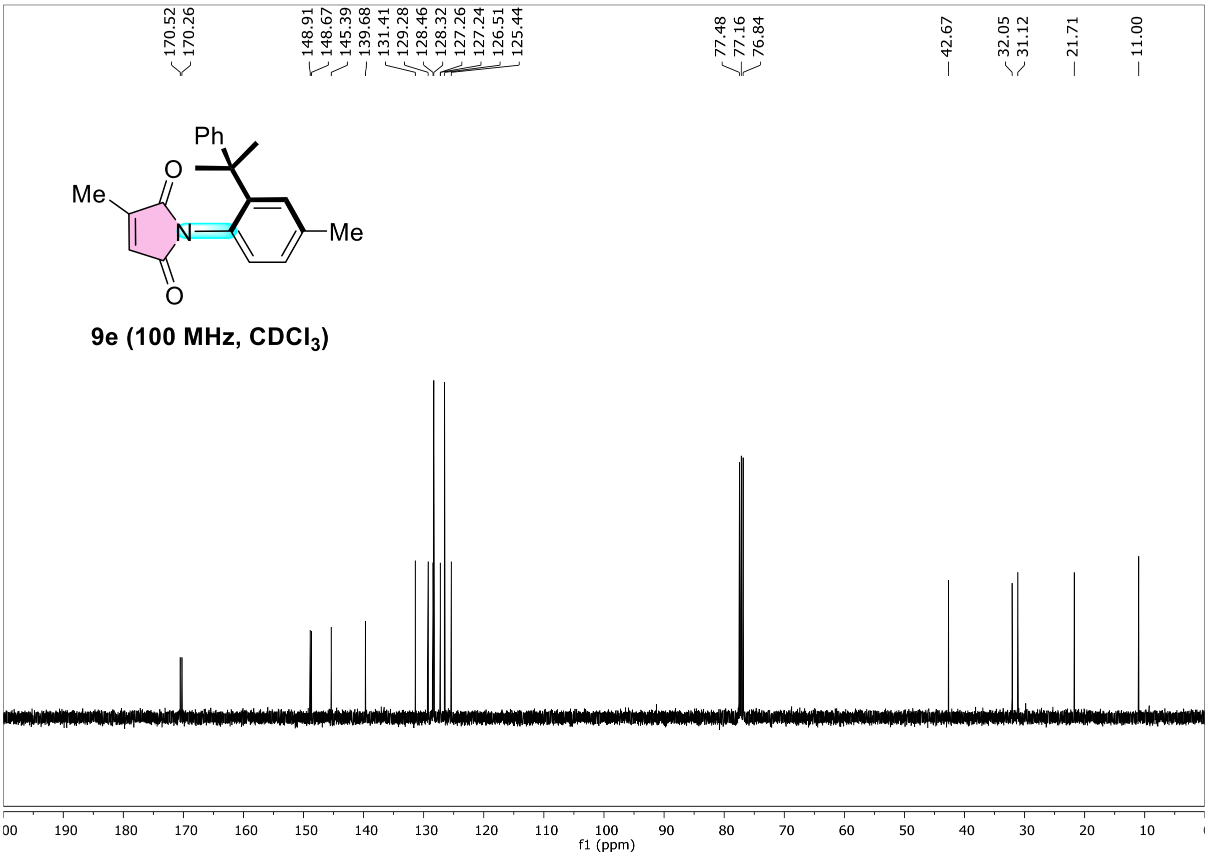

(*P*)-3-Bromo-1-(2-(*tert*-butyl)phenyl)-4-methyl-1*H*-pyrrole-2,5-dione (9f)

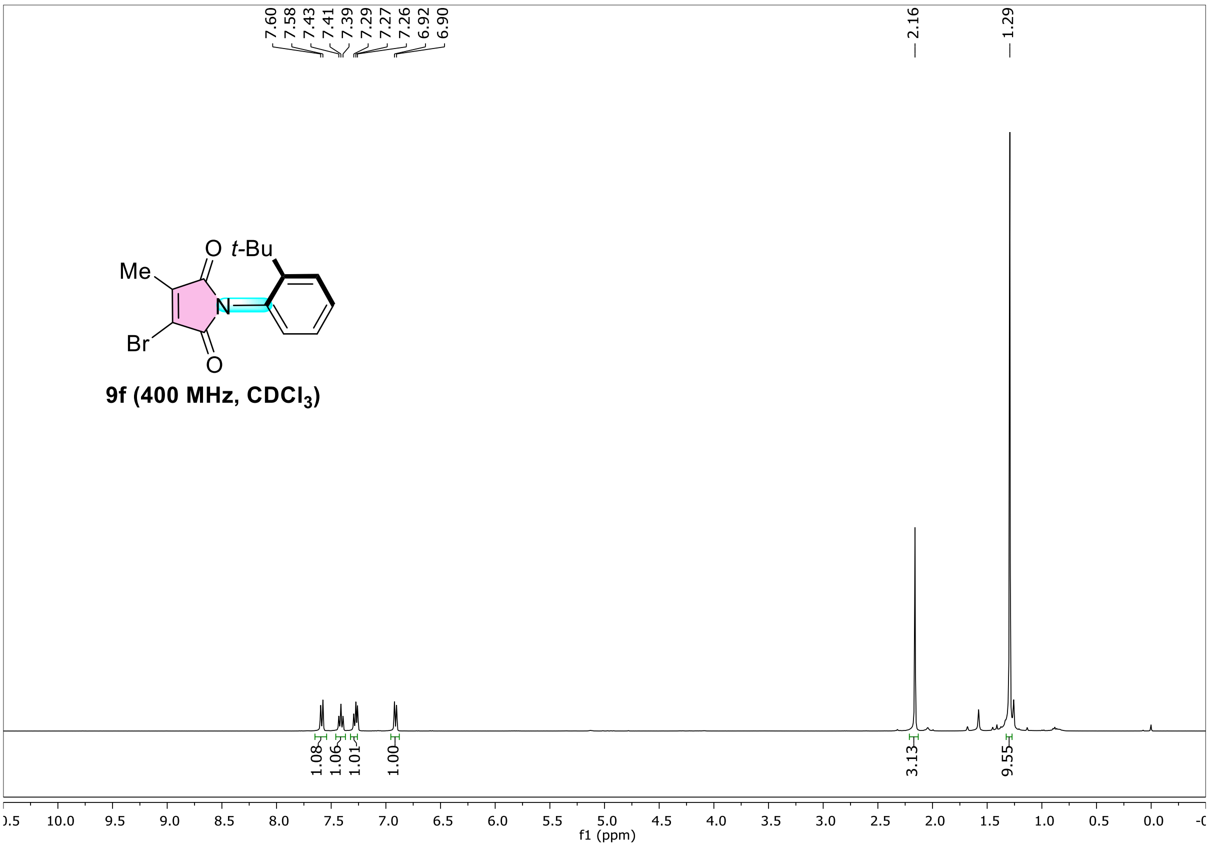

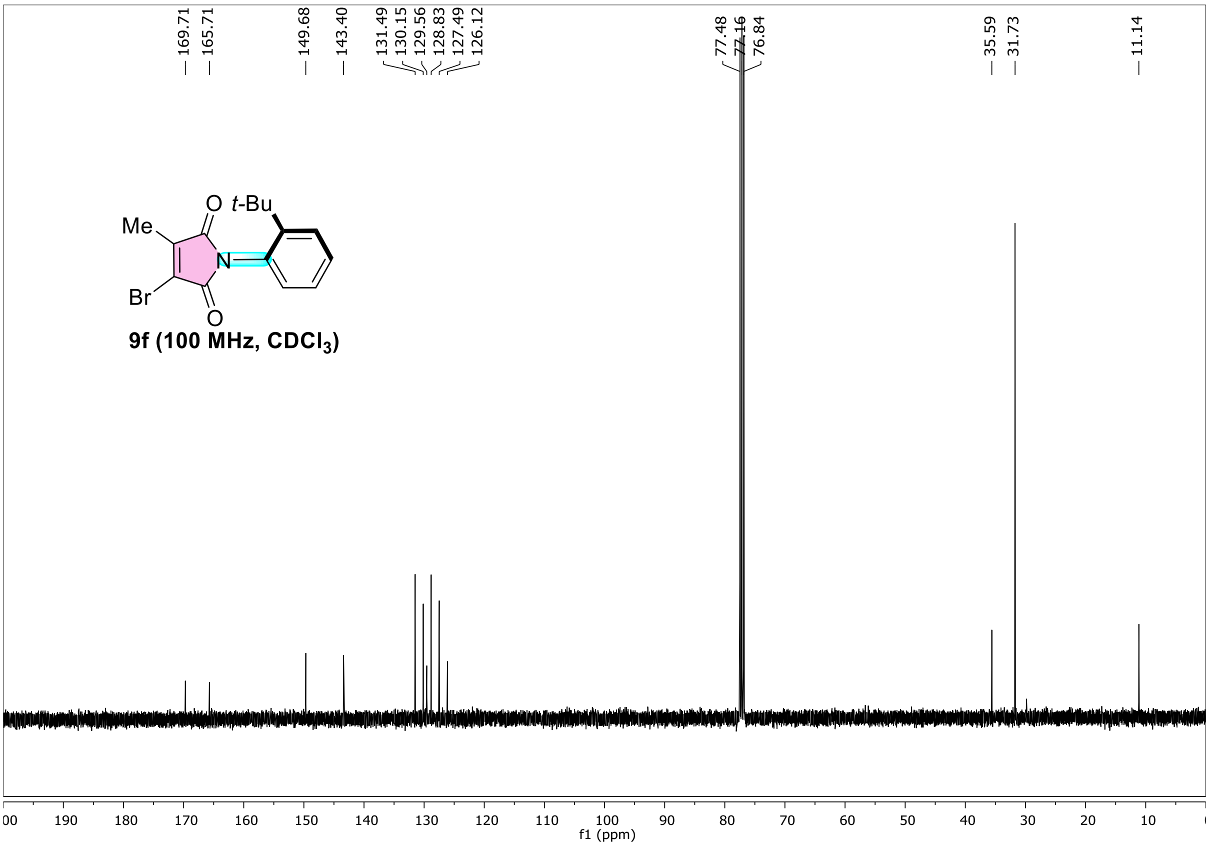

(*P*)-4-(Bromomethyl)-2-(2-(*tert*-butyl)phenyl)isoindoline-1,3-dione (10a)

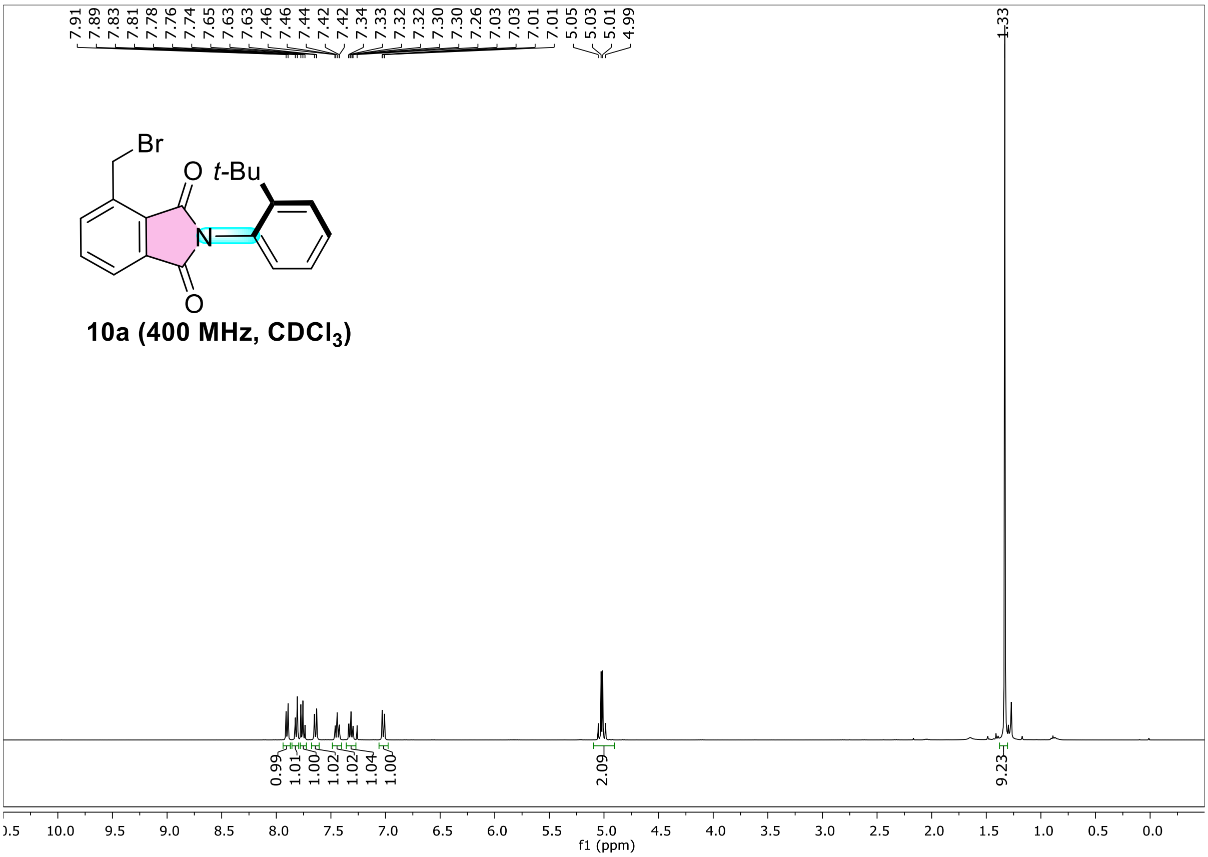

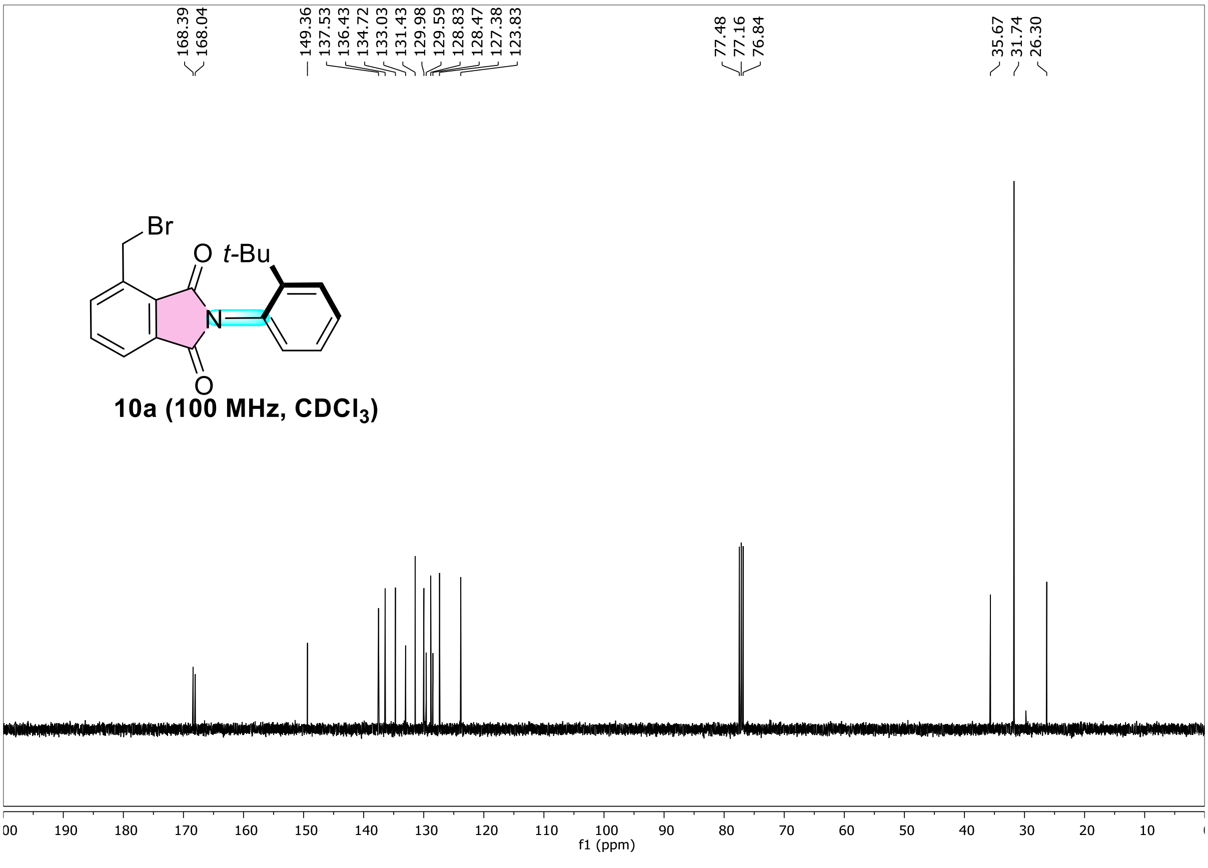

(*P*)-2-(2-(*tert*-Butyl)phenyl)-1,3-dioxoisoindoline-4-carbaldehyde (11a)

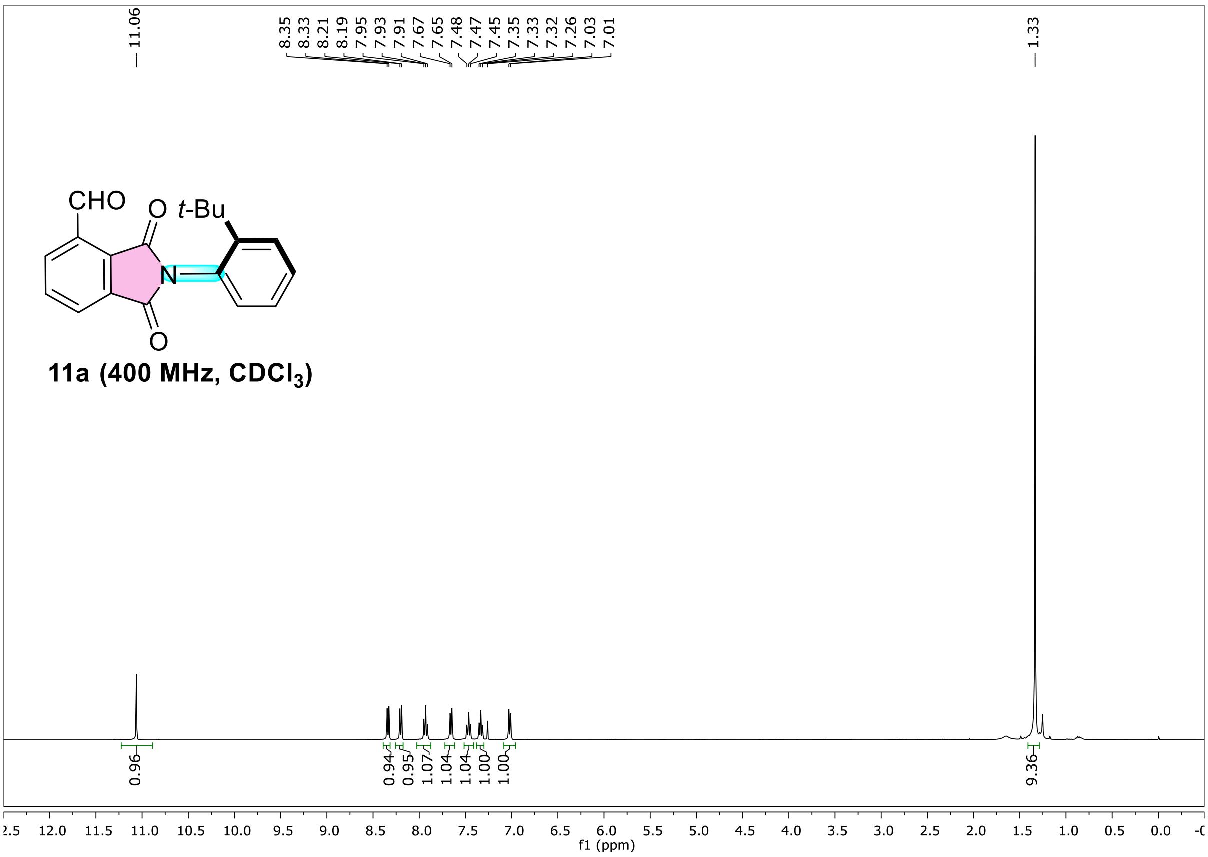

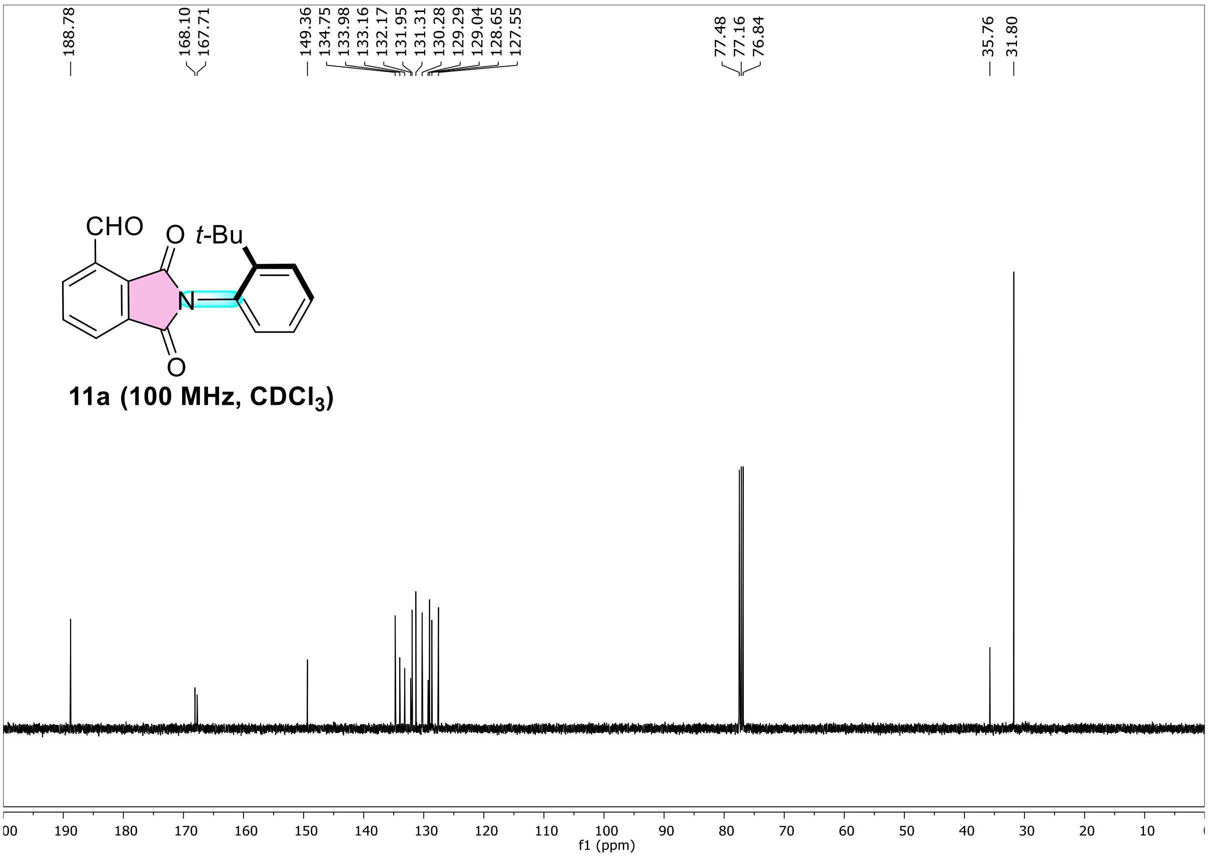

(*P*)-2-(2-(*tert*-Butyl)phenyl)-4-vinylisoindoline-1,3-dione (12a)

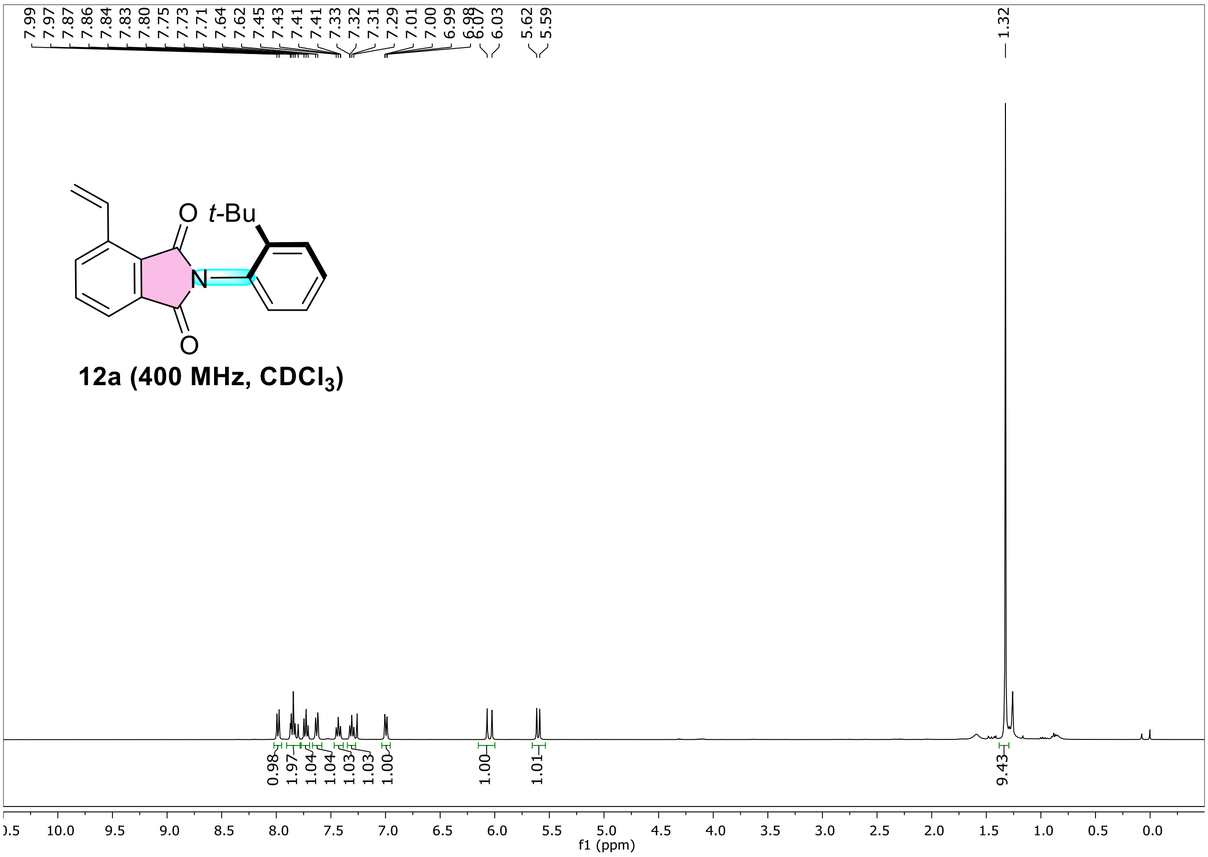

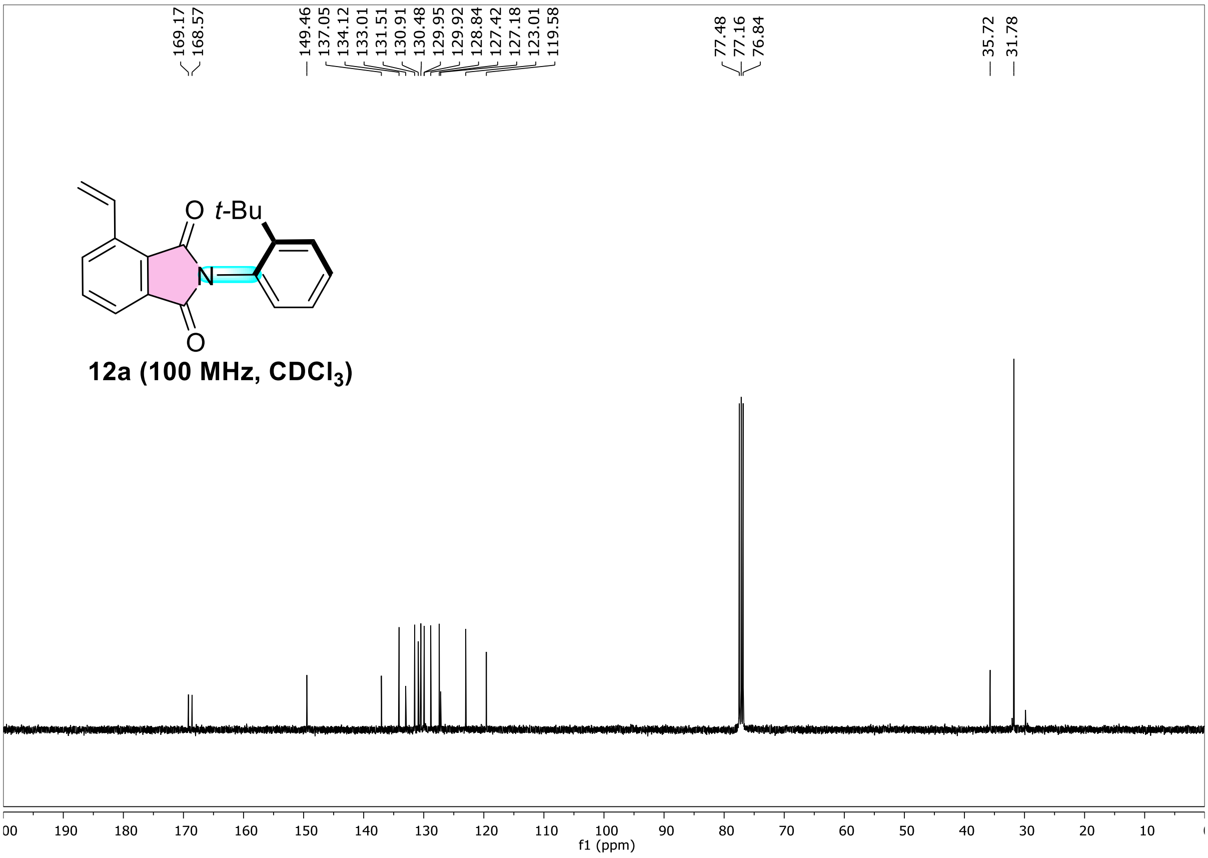

(*P*)-4-(Azidomethyl)-2-(2-(*tert*-butyl)phenyl)isoindoline-1,3-dione (13a)

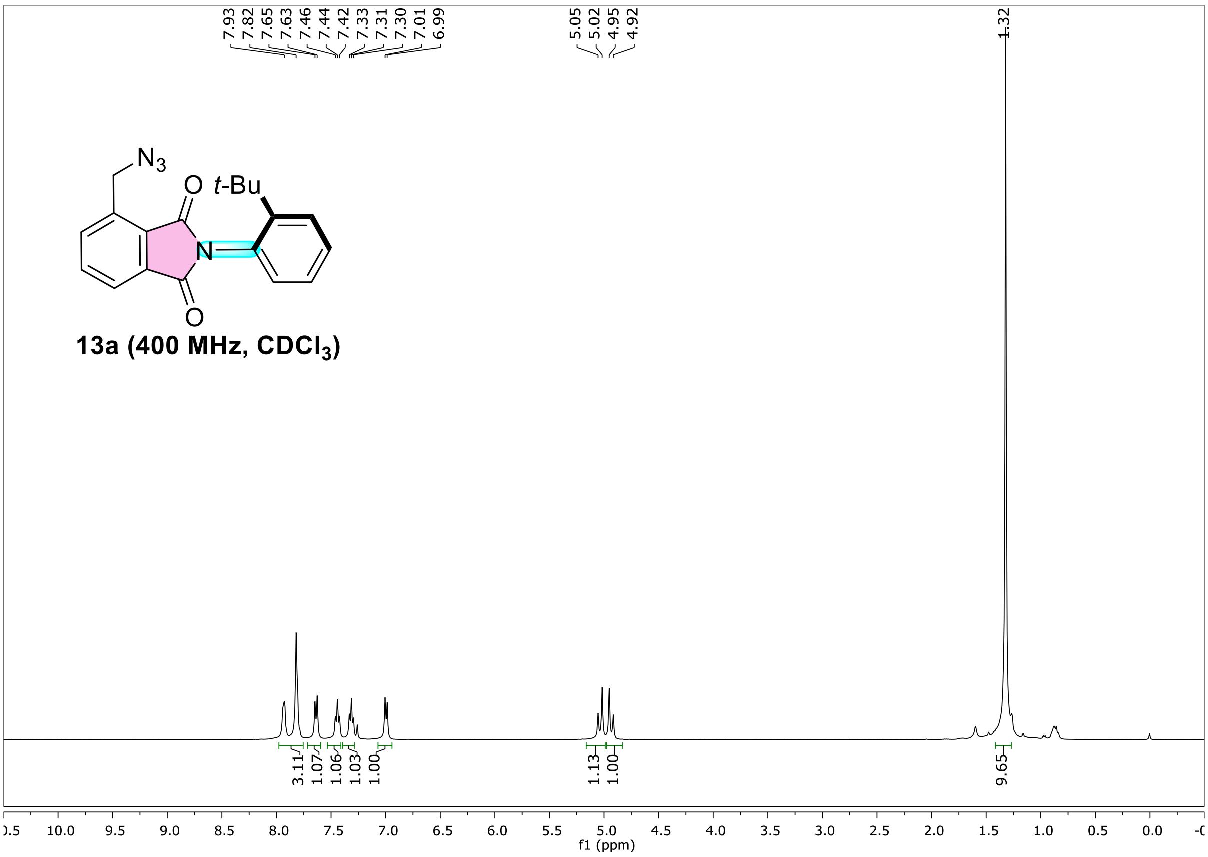

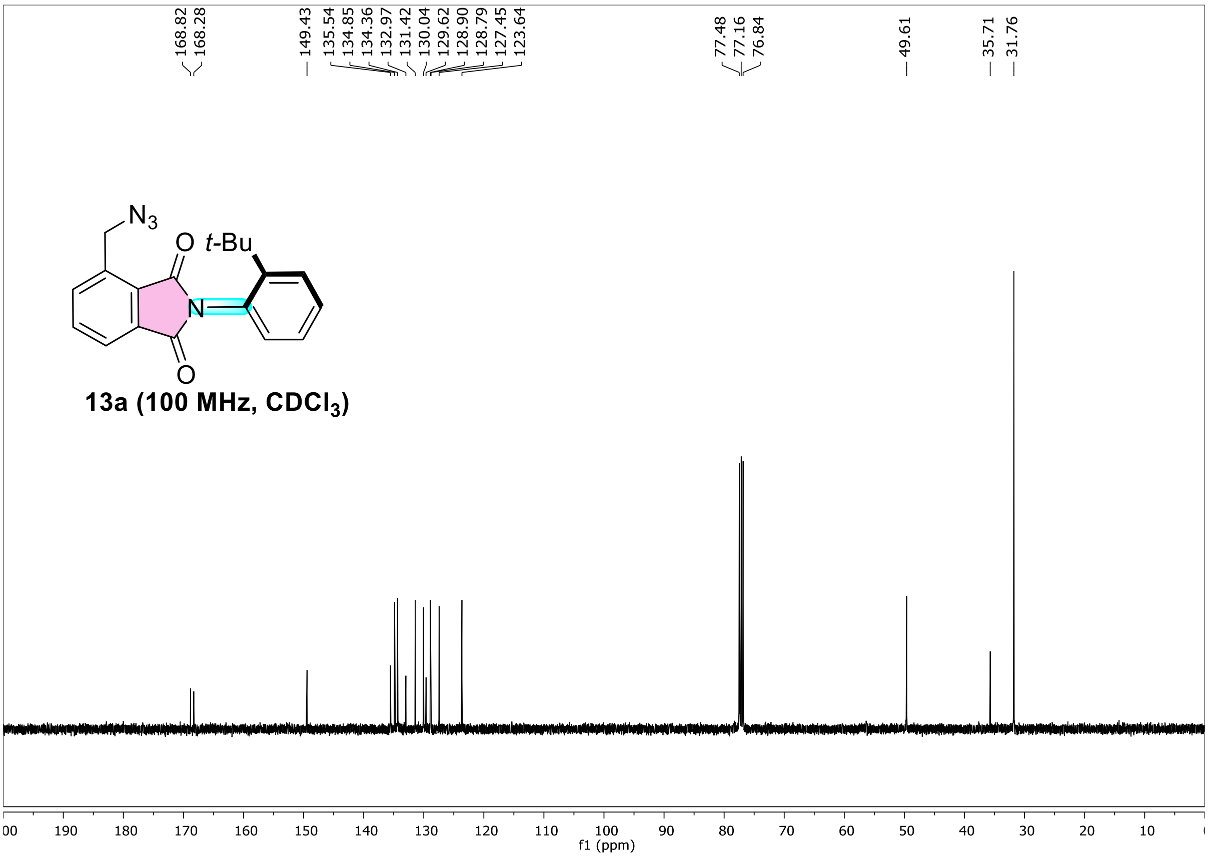

(*P*)-4-((1*H*-Benzo[*d*][1,2,3]triazol-1-yl)methyl)-2-(2-(*tert*-butyl)phenyl)isoindoline-1,3-dione (14a)

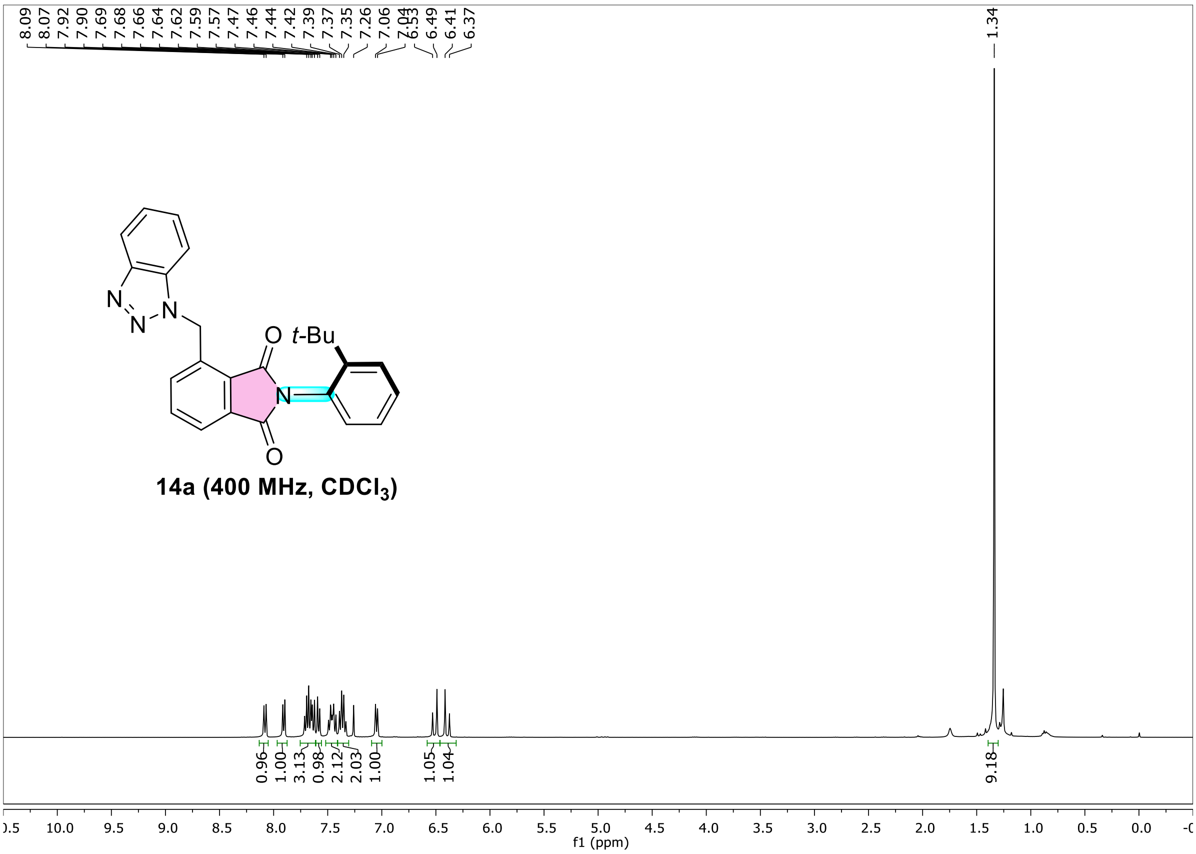

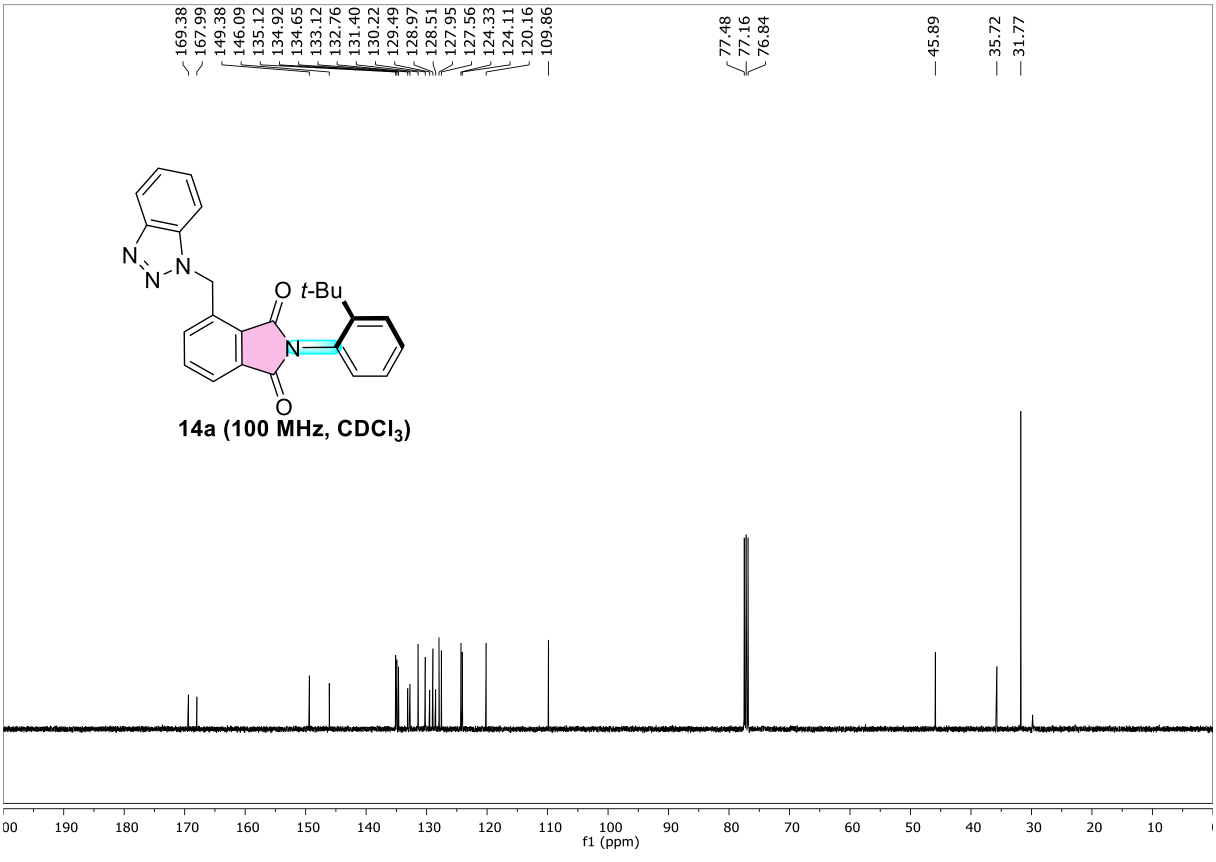

(*P*)-1-(2-(*tert*-Butyl)phenyl)-3-(butylamino)-4-methyl-1*H*-pyrrole-2,5-dione (15a)

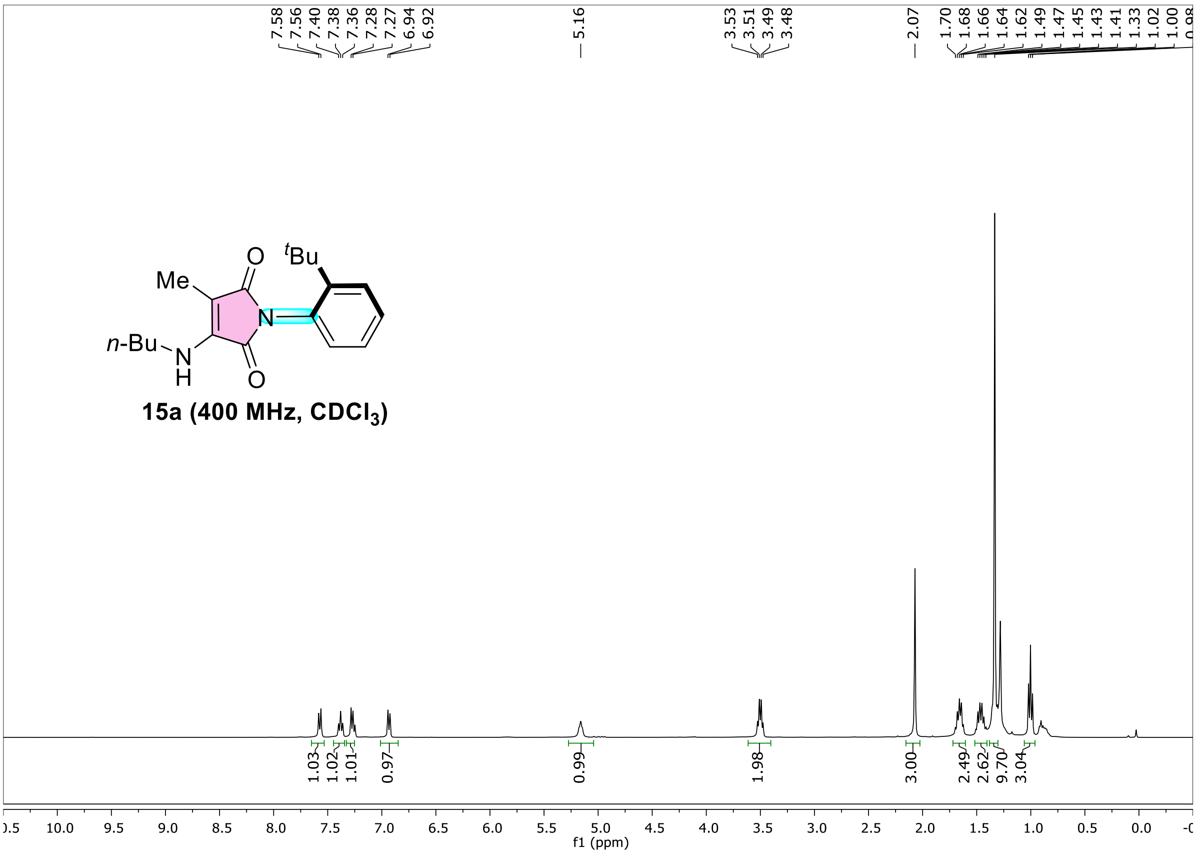

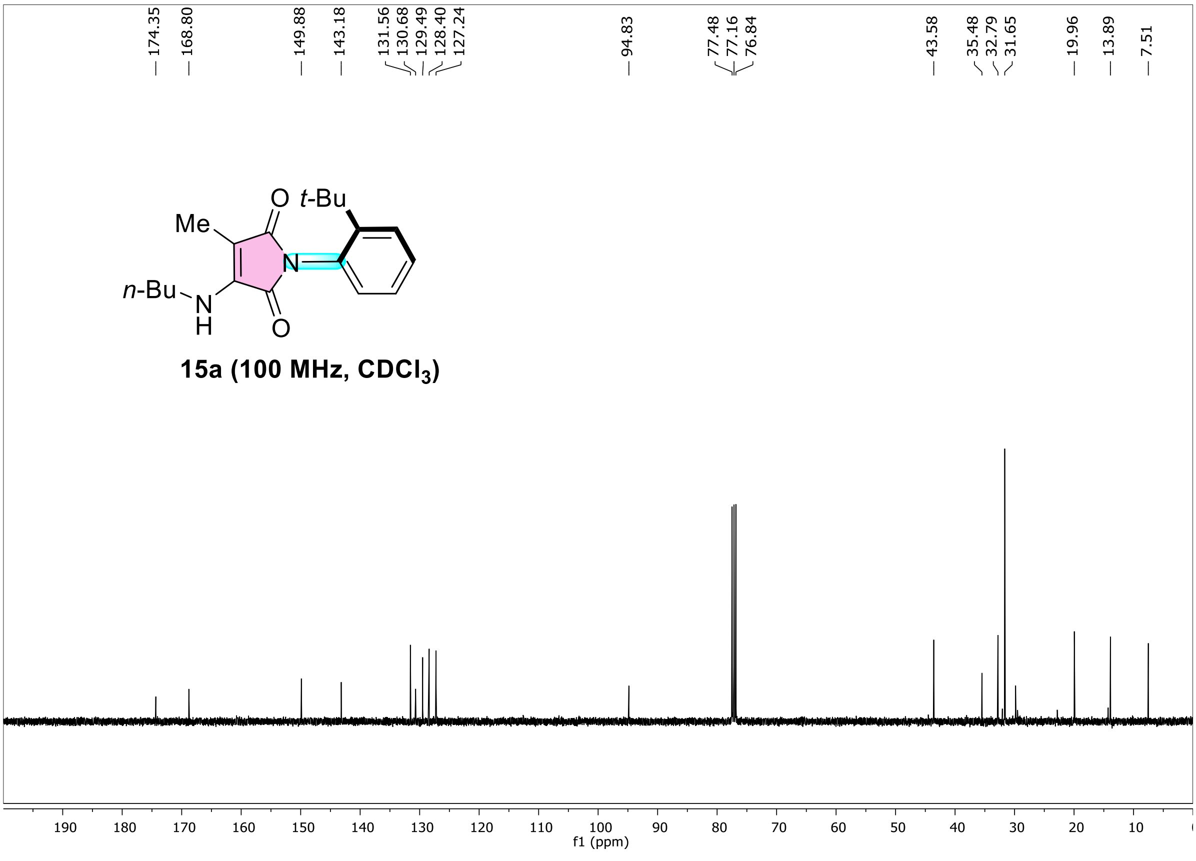

(*P*, *R*)-1-(2-(*tert*-Butyl)phenyl)-3-methylpyrrolidine-2,5-dione (16a)

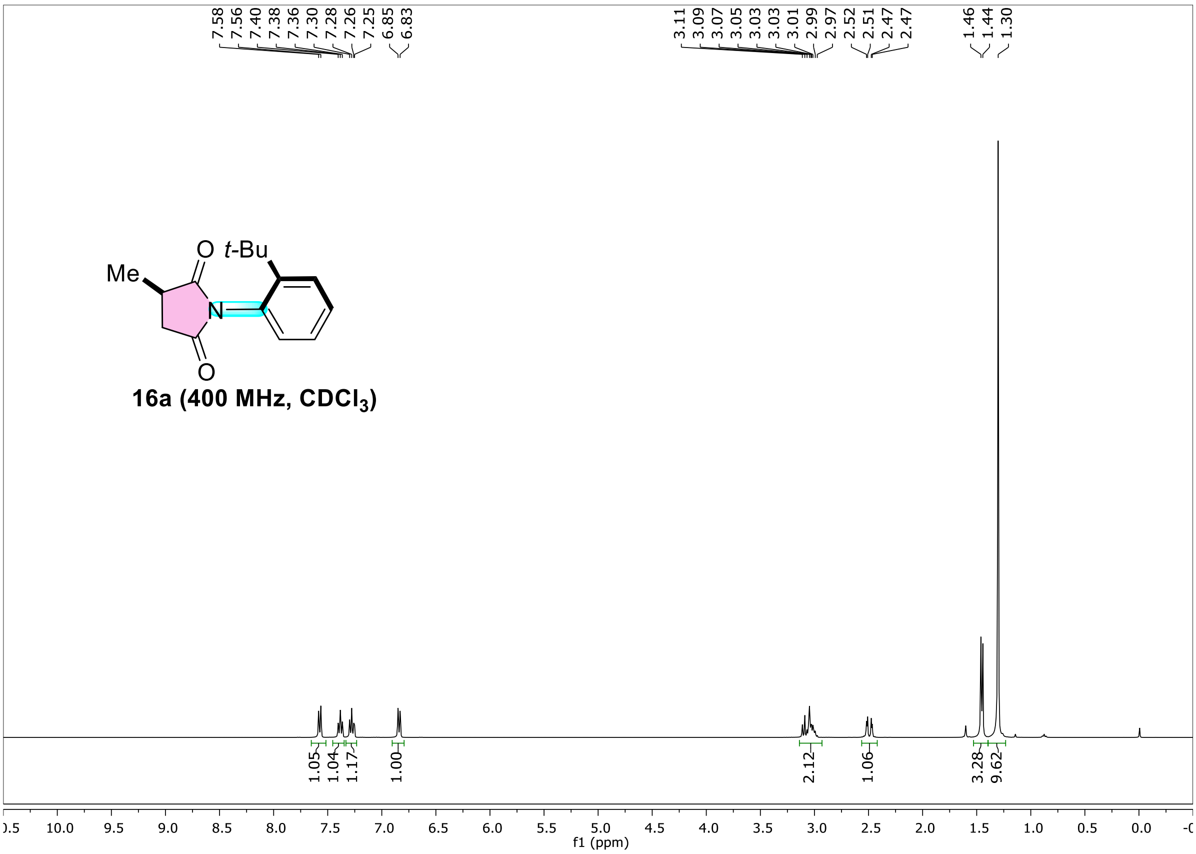

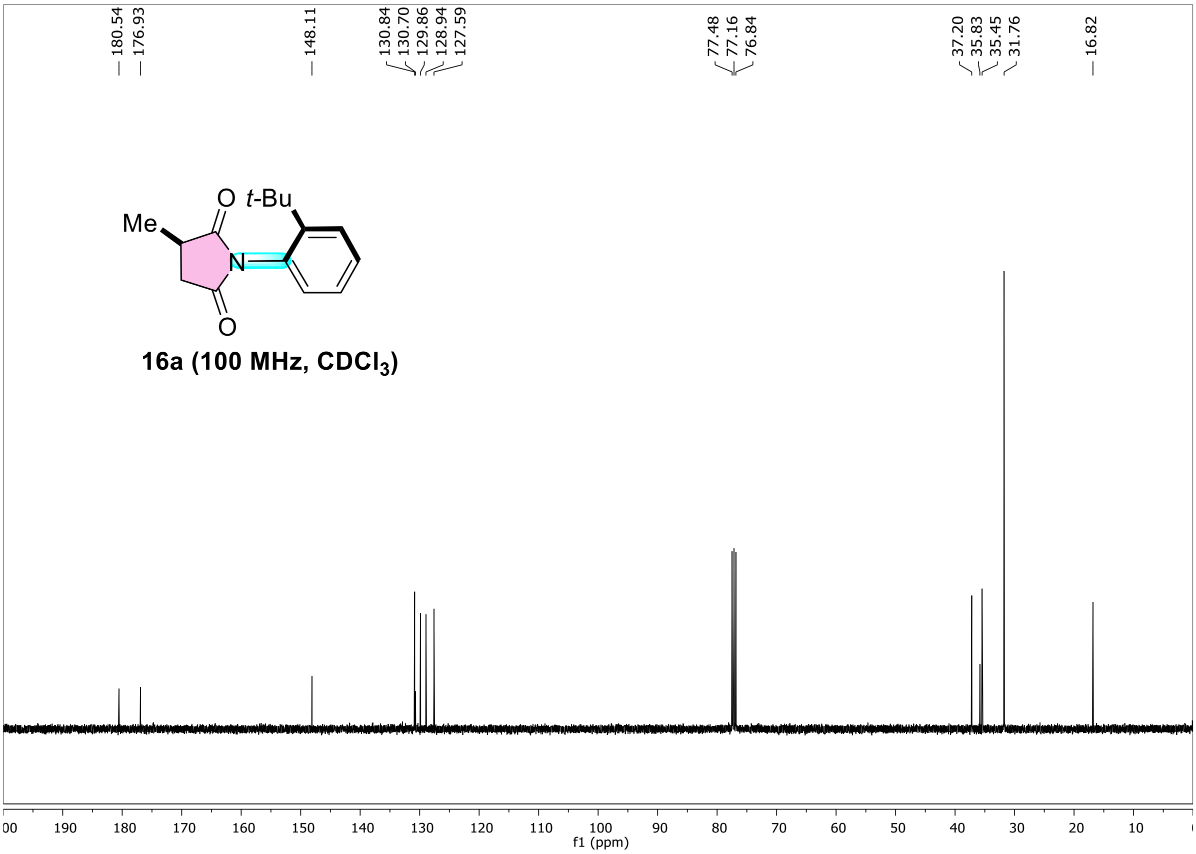

3.3 HPLC-Chromatogram of *N*-Aryl Phthalimide/Maleimide Derivatives

(*P*)-2-(2-(*tert*-Butyl)phenyl)-4-methylisoindoline-1,3-dione (2a)

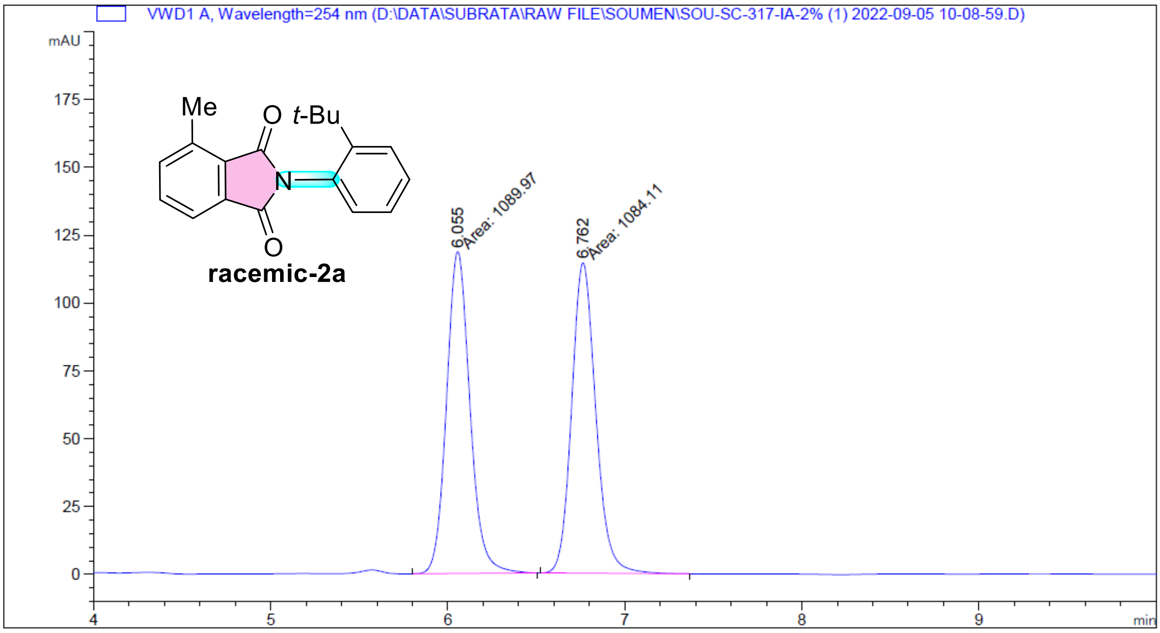

Peak #	RetTime [min]	Type	Width [min]	Area [mAU*s]	Height [mAU]	Area %
1	6.055	MM	0.1531	1089.96899	118.62458	50.1348
2	6.762	MM	0.1579	1084.10950	114.39774	49.8652

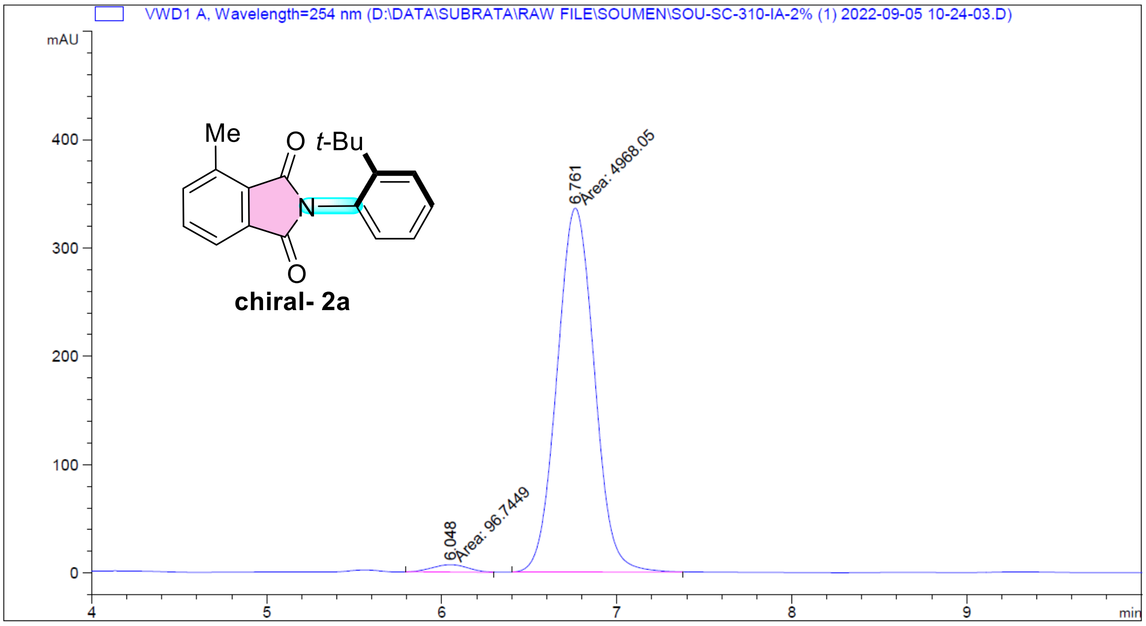

Peak #	RetTime [min]	Type	Width [min]	Area [mAU*s]	Height [mAU]	Area %
1	6.048	MM	0.2356	96.74489	6.84496	1.9101
2	6.761	MM	0.2465	4968.04688	335.92059	98.0899

Sample Info : CHIRALPAK IA, 2% IPA:HEXANE, 1.0 mL/min, 254 nm

(*M*)-2-(2-(*tert*-Butyl)phenyl)-4-methylisoindoline-1,3-dione (*ent*-2a)

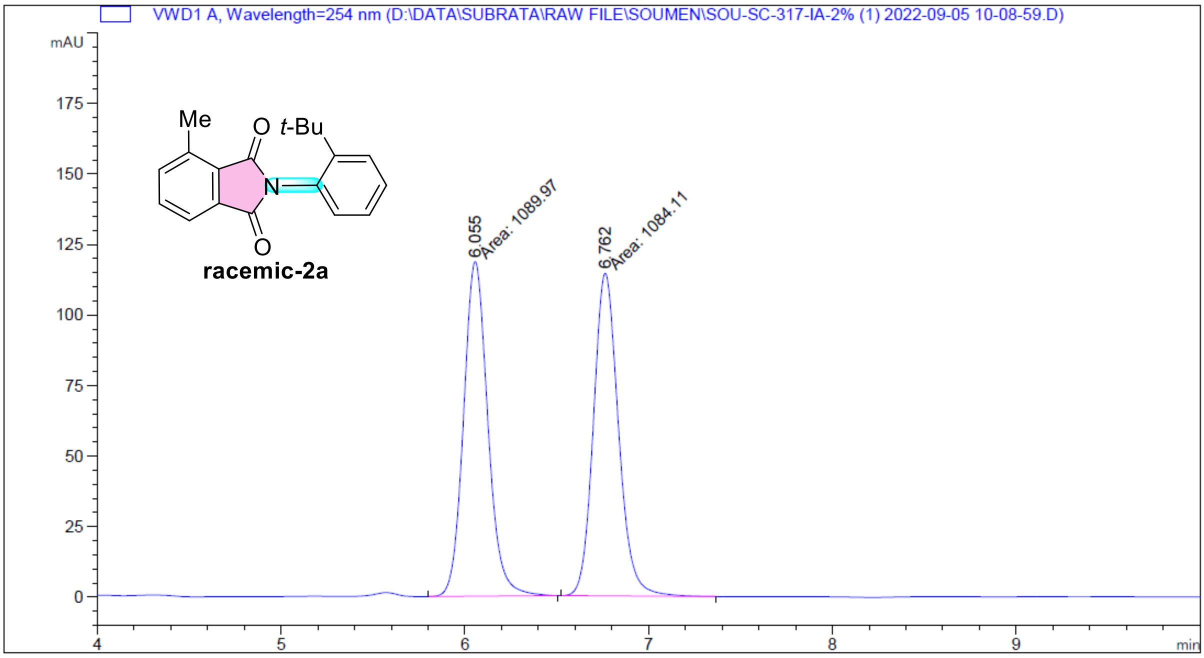

Peak #	RetTime [min]	Type	Width [min]	Area [mAU*s]	Height [mAU]	Area %
1	6.055	MM	0.1531	1089.96899	118.62458	50.1348
2	6.762	MM	0.1579	1084.10950	114.39774	49.8652

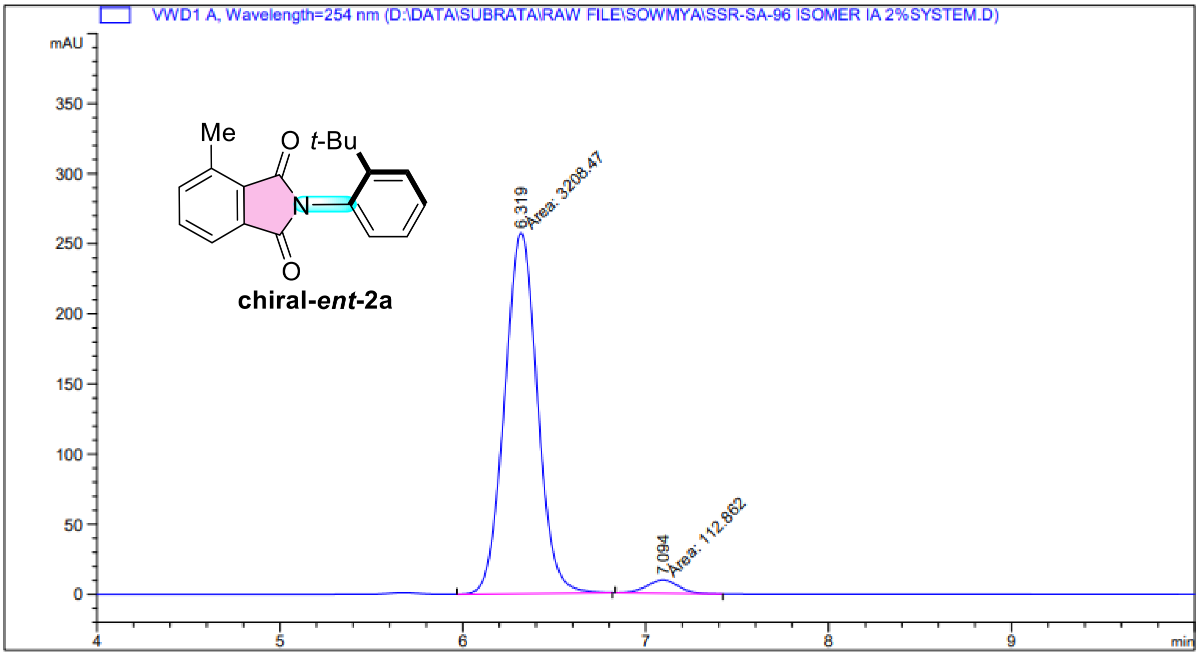

Peak #	RetTime [min]	Type	Width [min]	Area [mAU*s]	Height [mAU]	Area %
1	6.319	MM	0.2080	3208.46533	257.10028	96.6019
2	7.094	MM	0.1991	112.86174	9.44951	3.3981

Sample Info : CHIRALPAK IA, 2% IPA:HEXANE, 1.0 mL/min, 254 nm

(*P*)- 2-(2-(*tert*-Butyl)-4-methoxyphenyl)-4-methylisoindoline-1,3-dione (2b)

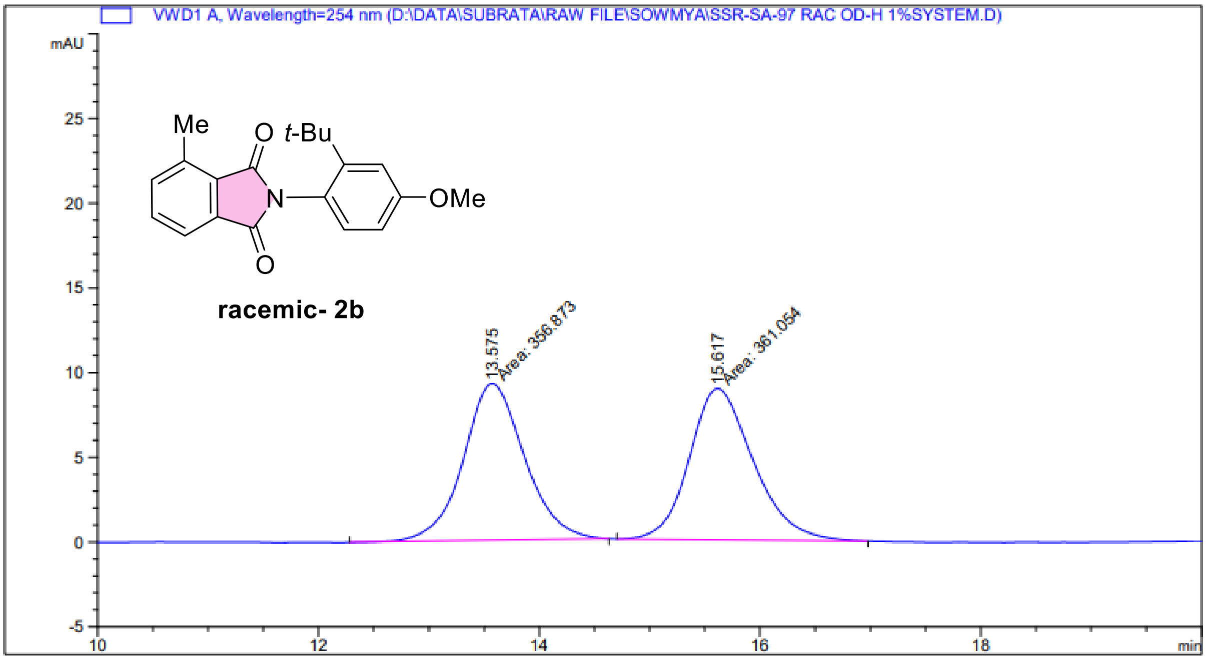

Peak #	RetTime [min]	Type	Width [min]	Area [mAU*s]	Height [mAU]	Area %
1	13.575	MM	0.6435	356.87299	9.24303	49.7088
2	15.617	MM	0.6731	361.05374	8.94019	50.2912

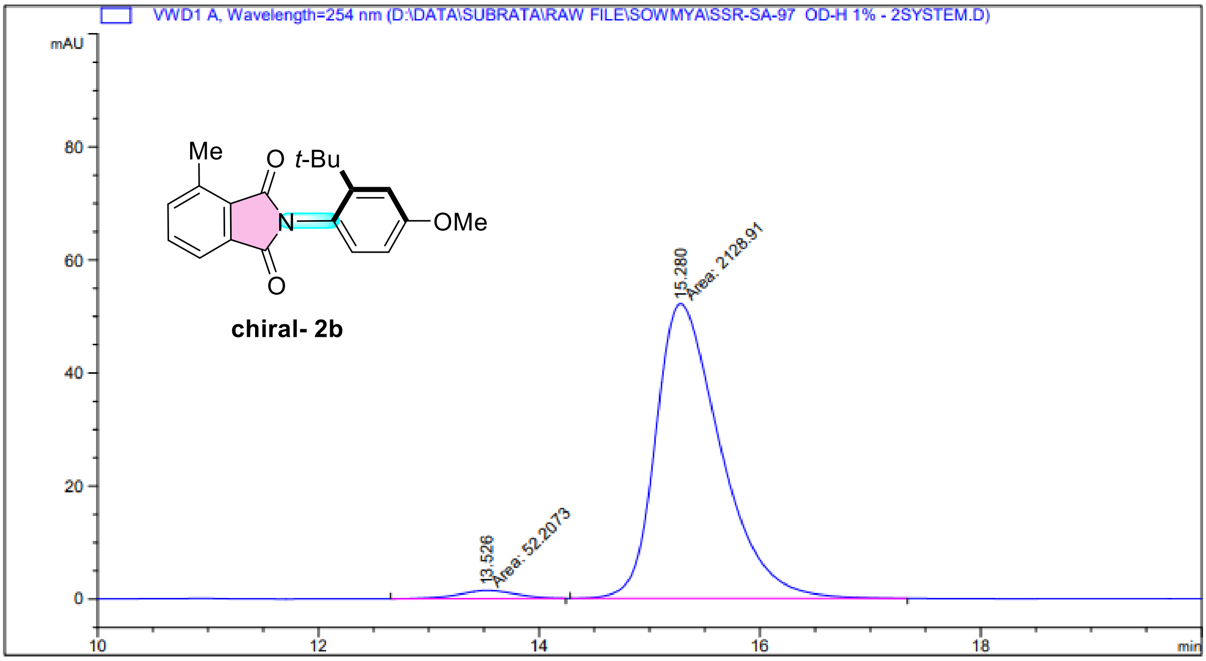

Peak #	RetTime [min]	Type	Width [min]	Area [mAU*s]	Height [mAU]	Area %
1	13.526	MM	0.6045	52.20726	1.43929	2.3936
2	15.280	MM	0.6809	2128.90723	52.10727	97.6064

Sample Info : CHIRALPAK OD-H, 1% IPA:HEXANE, 1.0 mL/min, 254 nm

(*P*)-2-(4-(Benzyloxy)-2-(*tert*-butyl)phenyl)-4-methylisoindoline-1,3-dione (2c)

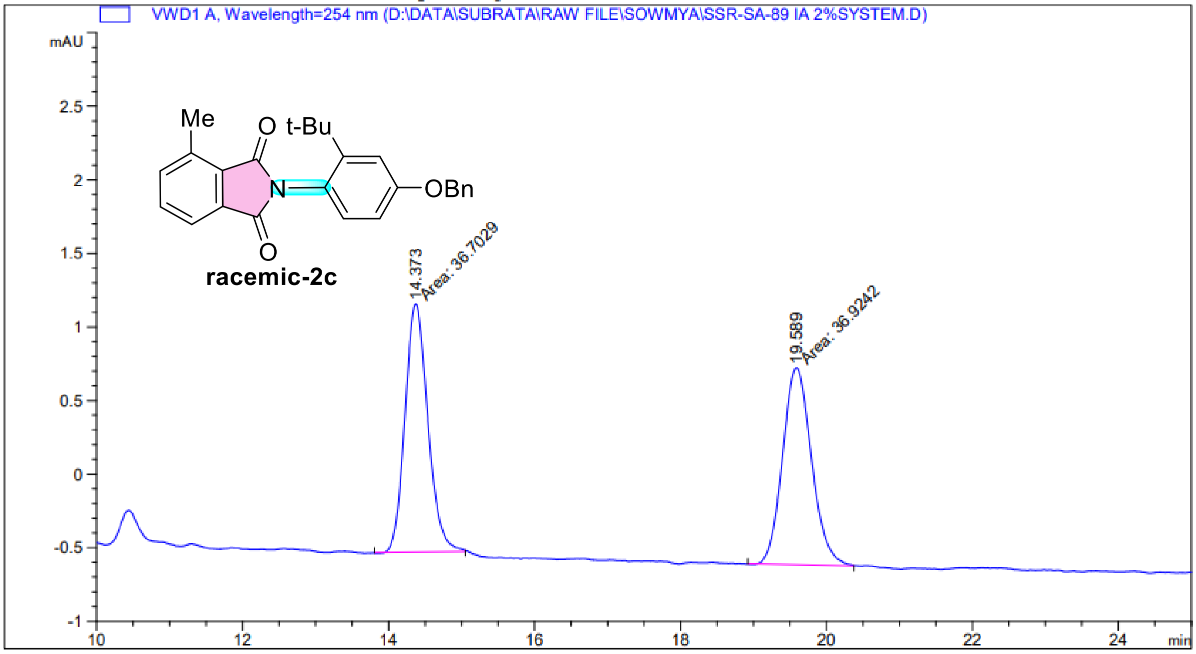

Peak #	RetTime [min]	Type	Width [min]	Area [mAU*s]	Height [mAU]	Area %
1	14.373	MM	0.3630	36.70287	1.68498	49.8497
2	19.589	MM	0.4600	36.92416	1.33792	50.1503

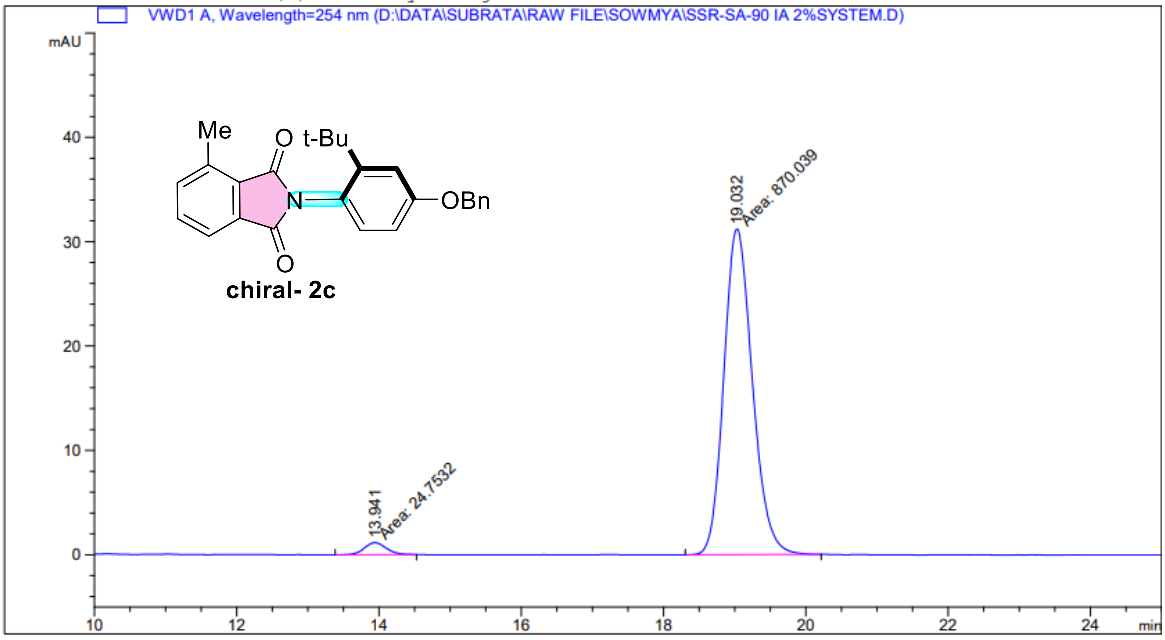

Peak #	RetTime [min]	Type	Width [min]	Area [mAU*s]	Height [mAU]	Area %
1	13.941	MM	0.3592	24.75318	1.14846	2.7664
2	19.032	MM	0.4646	870.03918	31.21436	97.2336

Sample Info : CHIRALPAK IA, 2% IPA:HEXANE, 1.0 mL/min, 254 nm

(*P*)-2-(4-Bromo-2-(*tert*-butyl)phenyl)-4-methylisoindoline-1,3-dione (2d)

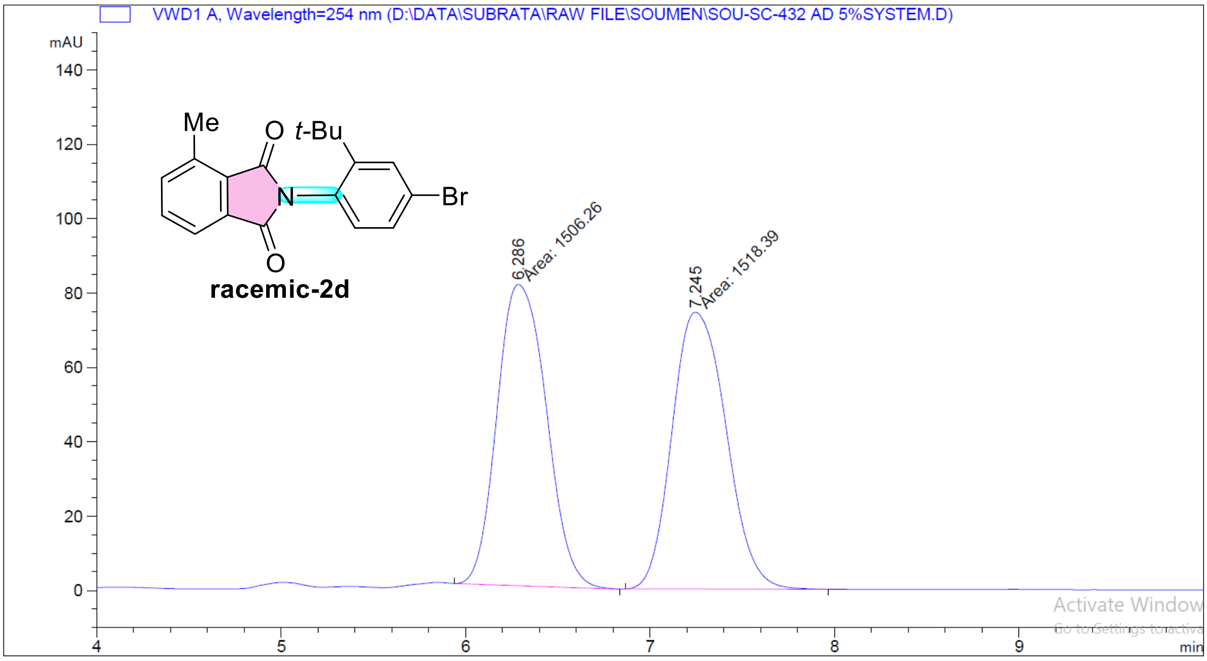

Peak #	RetTime [min]	Type	Width [min]	Area [mAU*s]	Height [mAU]	Area %
1	6.286	MM	0.3099	1506.25916	81.00315	49.7995
2	7.245	MM	0.3400	1518.38623	74.43159	50.2005

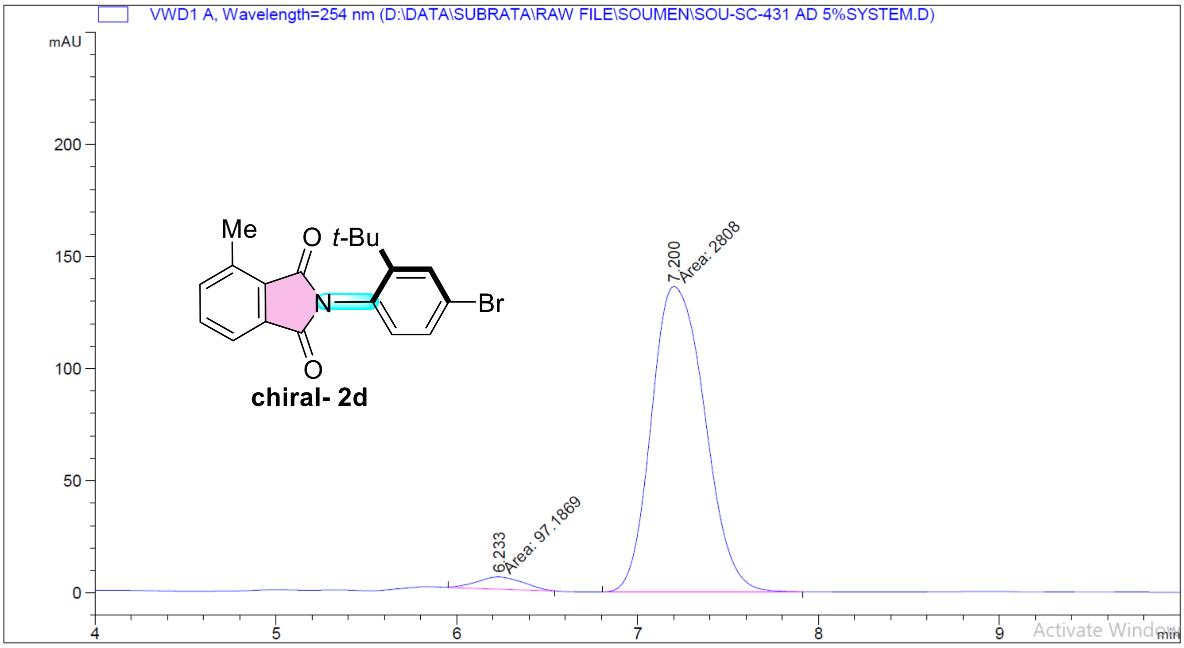

Peak #	RetTime [min]	Type	Width [min]	Area [mAU*s]	Height [mAU]	Area %
1	6.233	MM	0.2967	97.18694	5.45919	3.3453
2	7.200	MM	0.3430	2807.99561	136.42490	96.6547

Sample Info : CHIRALPAK AD, 5% IPA:HEXANE, 1.0 mL/min, 254 nm

(*P*)- 2-(2-(*tert*-Butyl)-4-chlorophenyl)-4-methylisoindoline-1,3-dione (2e)

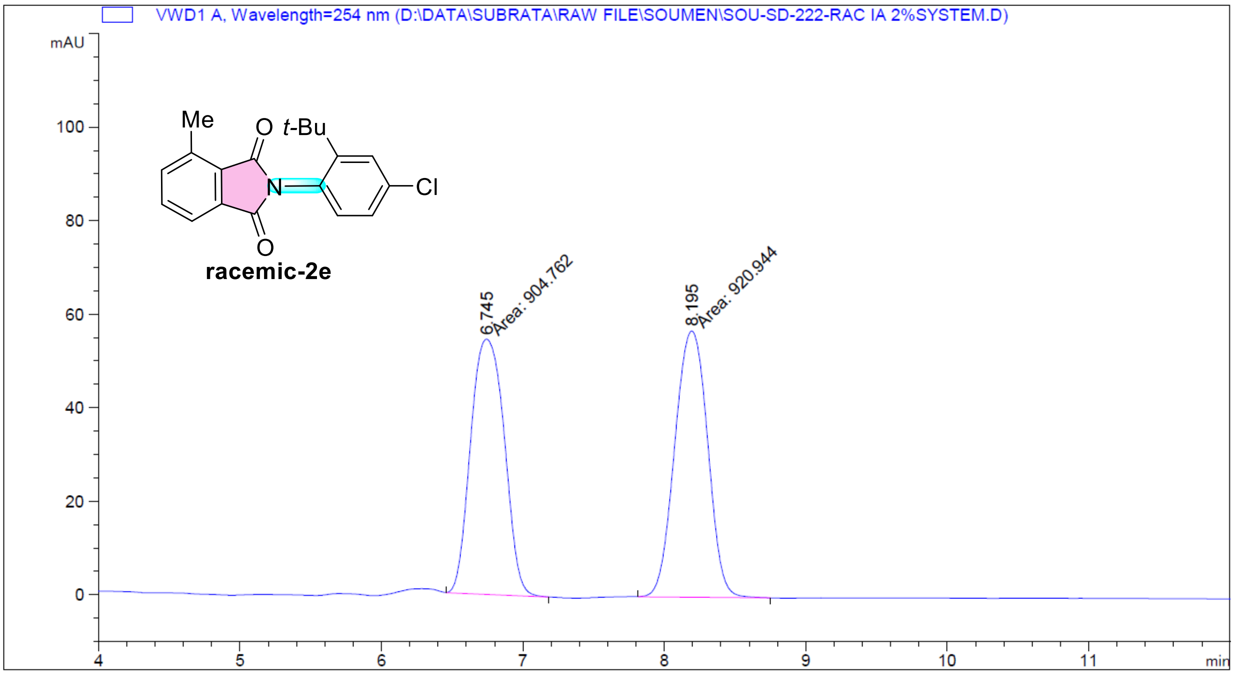

Peak #	RetTime [min]	Type	Width [min]	Area [mAU*s]	Height [mAU]	Area %
1	6.745	MM	0.2762	904.76202	54.58923	49.5568
2	8.195	MM	0.2695	920.94379	56.94939	50.4432

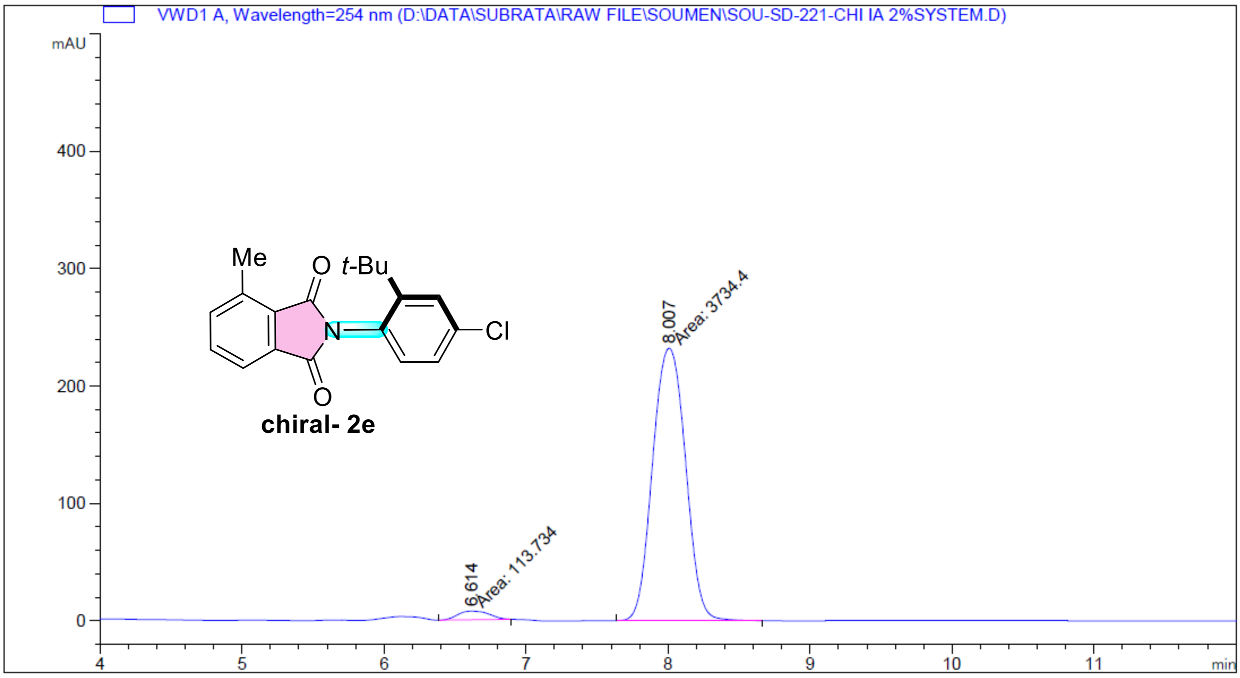

Peak #	RetTime [min]	Type	Width [min]	Area [mAU*s]	Height [mAU]	Area %
1	6.614	MM	0.2539	113.73364	7.46589	2.9556
2	8.007	MM	0.2682	3734.39575	232.05663	97.0444

Sample Info : CHIRALPAK IA , 2% IPA-HEXANE, 1.0 mL/min, 254 nm

(*P*)-2-(2-(*tert*-Butyl)-4-iodophenyl)-4-methylisoindoline-1,3-dione(2f)

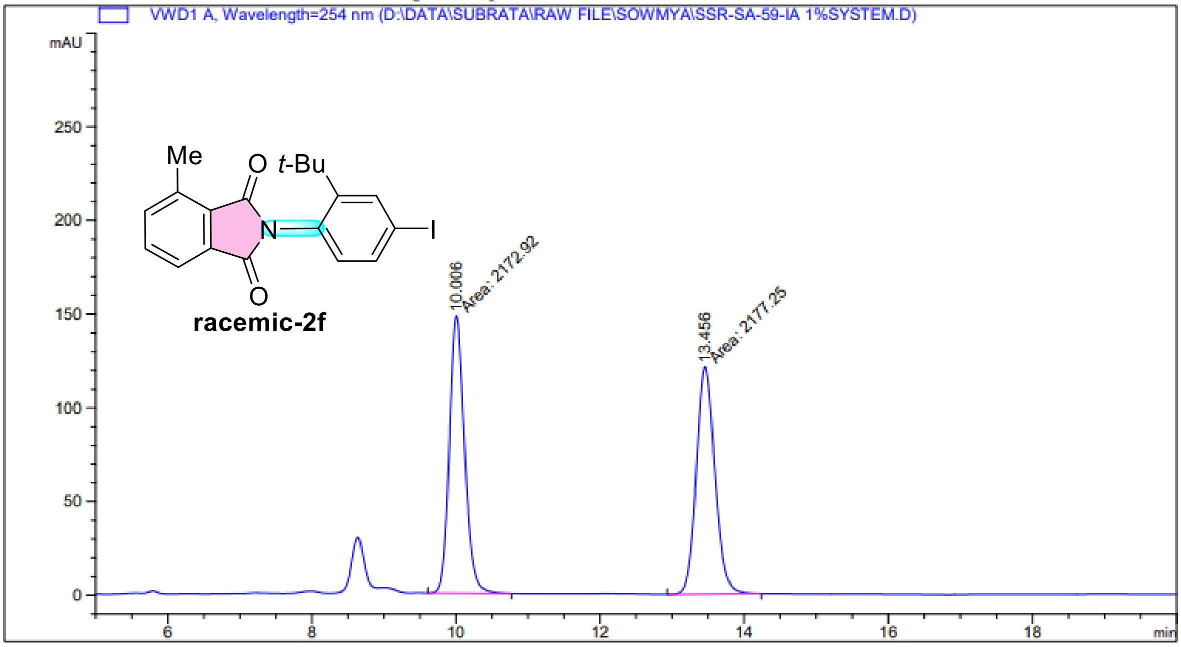

Peak #	RetTime [min]	Type	Width [min]	Area [mAU*s]	Height [mAU]	Area %
1	10.006	MM	0.2445	2172.92188	148.11237	49.9503
2	13.456	MM	0.2990	2177.24805	121.34298	50.0497

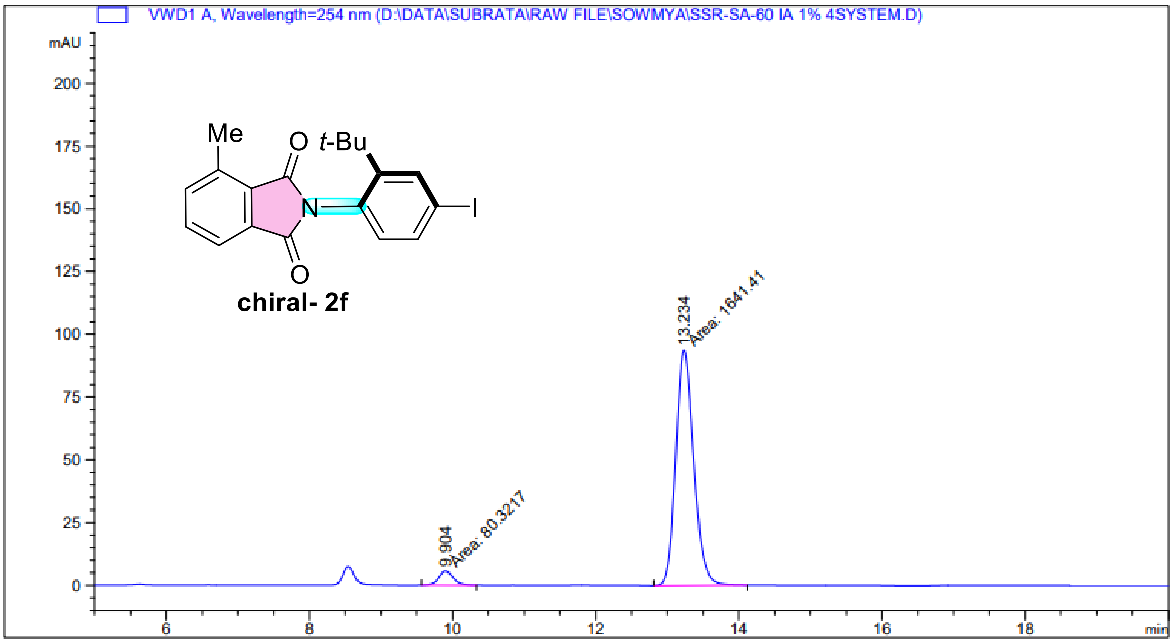

Peak #	RetTime [min]	Type	Width [min]	Area [mAU*s]	Height [mAU]	Area %
1	9.904	MM	0.2369	80.32173	5.65166	4.6652
2	13.234	MM	0.2918	1641.40540	93.74986	95.3348

Sample Info : CHIRALPAK IA, 1% IPA:HEXANE, 1.0 mL/min, 254 nm

(*P*)-2-(3-(*tert*-Butyl)-[1,1'-biphenyl]-4-yl)-4-methylisoindoline-1,3-dione (2g)

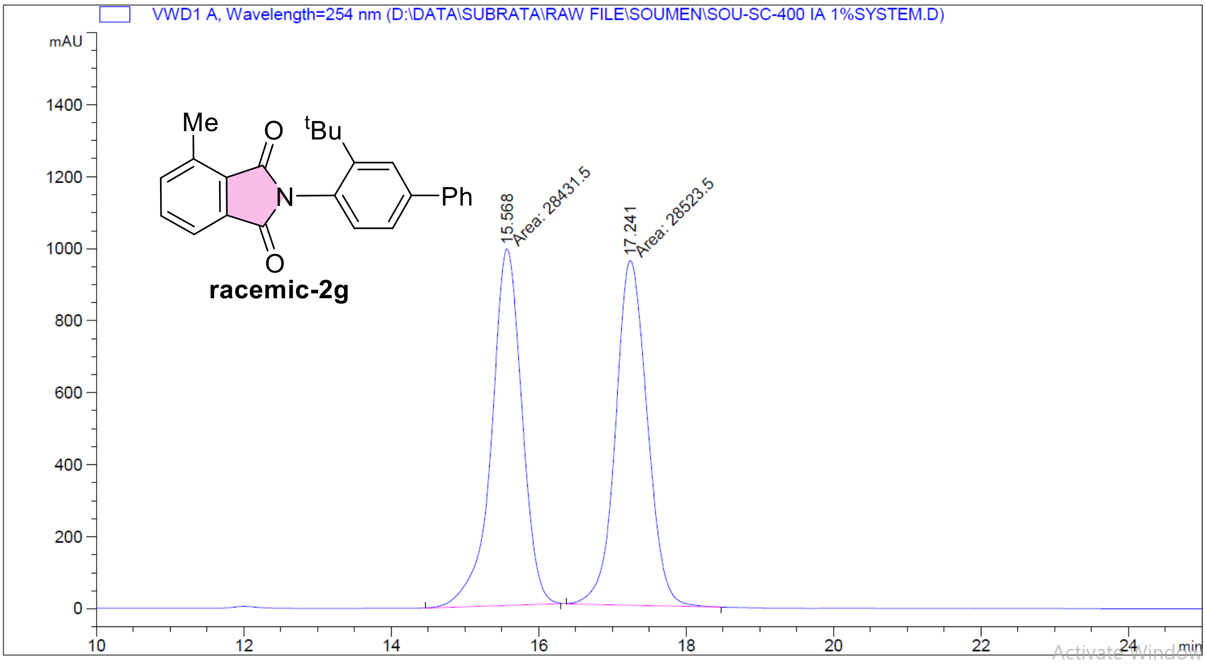

Peak #	RetTime [min]	Type	Width [min]	Area [mAU*s]	Height [mAU]	Area %
1	15.568	MM	0.4788	2.84315e4	989.58740	49.9192
2	17.241	MM	0.4967	2.85235e4	957.09796	50.0808

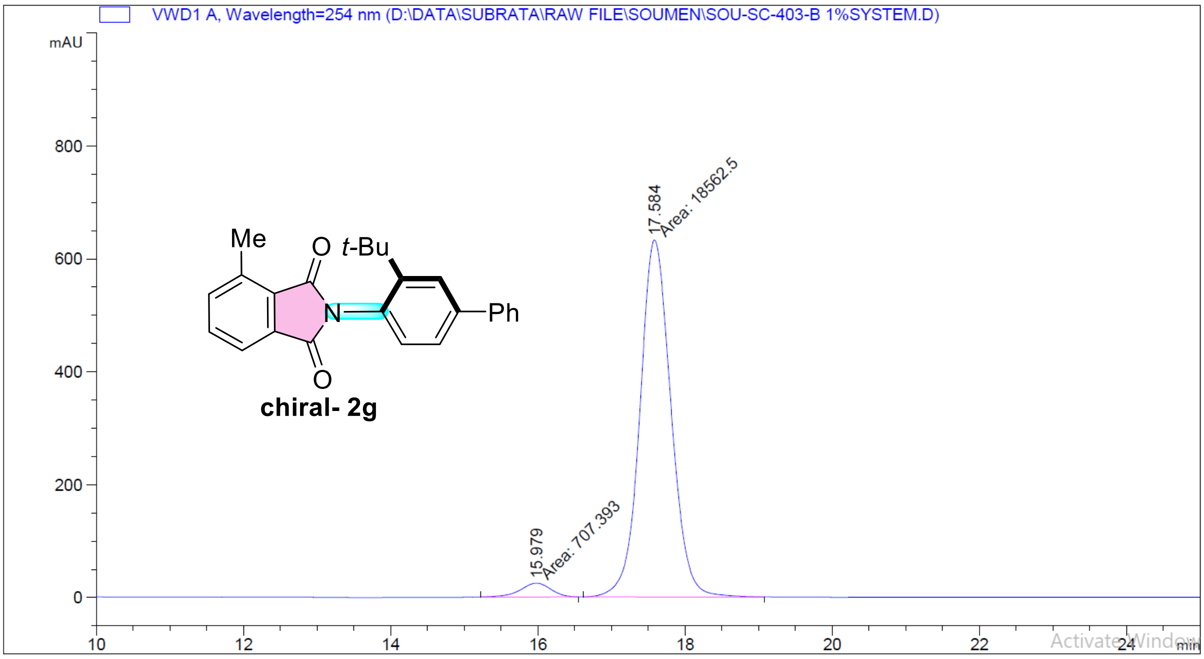

Peak #	RetTime [min]	Type	Width [min]	Area [mAU*s]	Height [mAU]	Area %
1	15.979	MM	0.4810	707.39319	24.51213	3.6710
2	17.584	MM	0.4892	1.85625e4	632.38507	96.3290

Sample Info : CHIRALPAK IA, 1% IPA:HEXANE, 1.0 mL/min, 254 nm

(*P*)-Ethyl 3-(*tert*-butyl)-4-(4-methyl-1,3-dioxoisoindolin-2-yl)benzoate (2h)

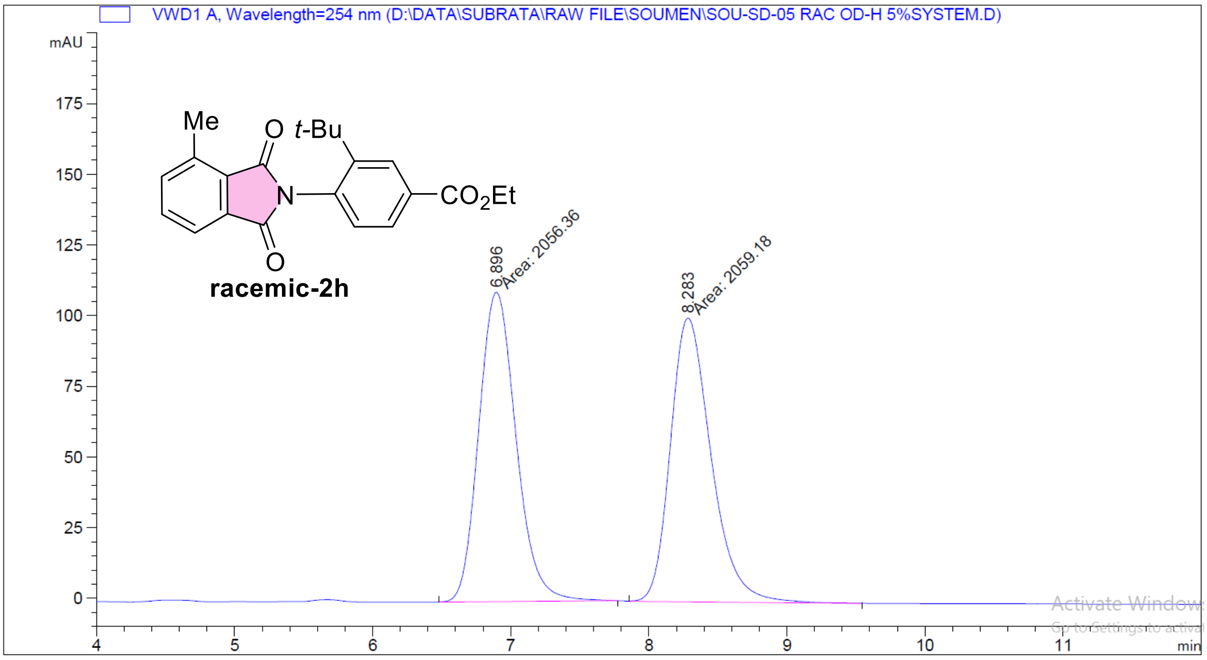

Peak #	RetTime [min]	Type	Width [min]	Area [mAU*s]	Height [mAU]	Area %
1	6.896	MM	0.3132	2056.35864	109.43506	49.9657
2	8.283	MM	0.3418	2059.18311	100.40502	50.0343

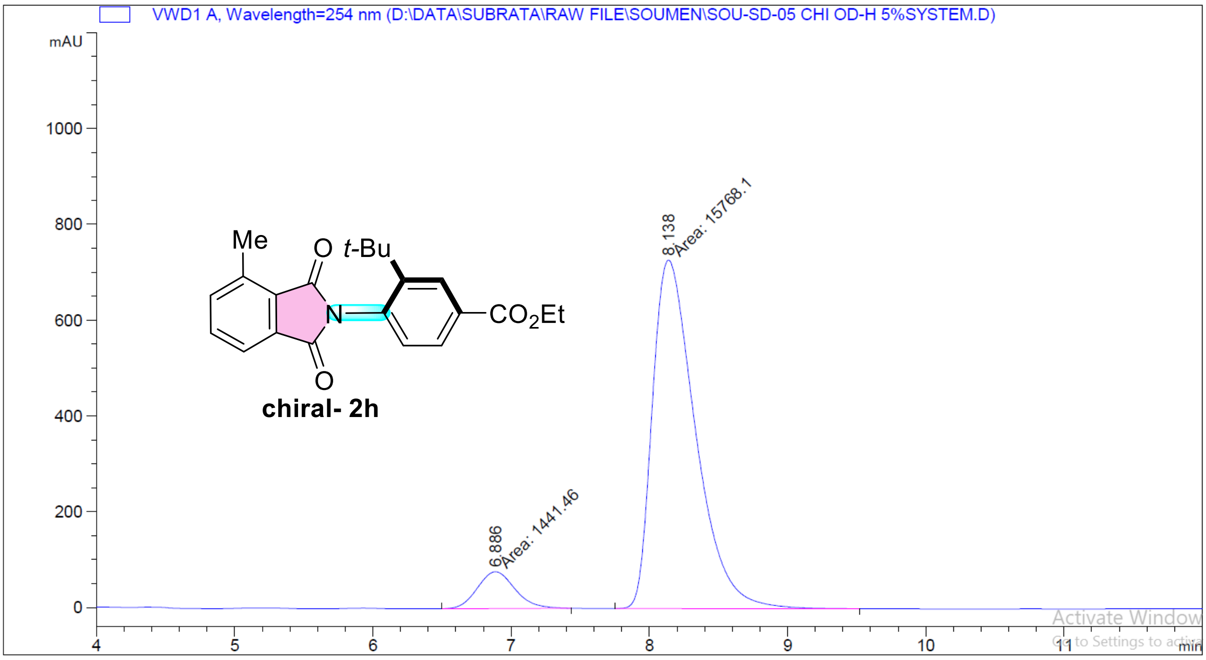

Peak #	RetTime [min]	Type	Width [min]	Area [mAU*s]	Height [mAU]	Area %
1	6.886	MM	0.3133	1441.45776	76.67589	8.3759
2	8.138	MM	0.3611	1.57681e4	727.81946	91.6241

Sample Info : CHIRALCELL OD-H, 5% IPA-HEXANE, 1.0 mL/min, 254 nm

(*P*)-2-(2-(*tert*-Butyl)-4-(thiophen-3-yl)phenyl)-4-methylisoindoline-1,3-dione (2i)

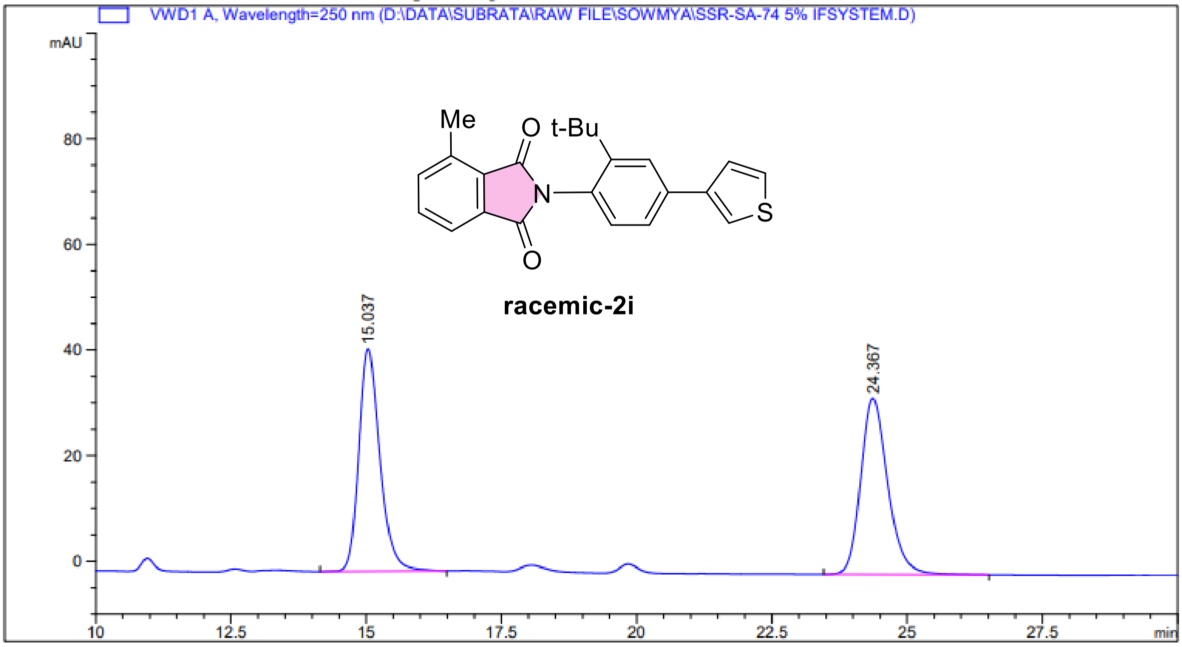

Peak #	RetTime [min]	Type	Width [min]	Area [mAU*s]	Height [mAU]	Area %
1	15.037	BB	0.4139	1144.90747	42.15334	49.6152
2	24.367	BB	0.5313	1162.66504	33.38386	50.3848

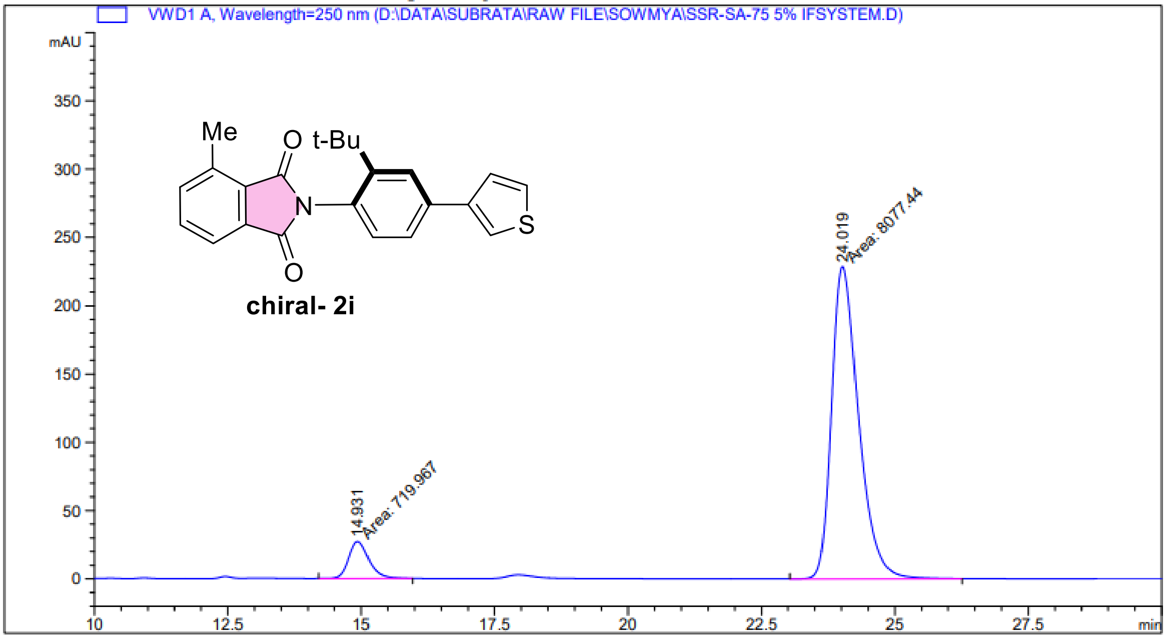

Peak #	RetTime [min]	Type	Width [min]	Area [mAU*s]	Height [mAU]	Area %
1	14.931	MM	0.4424	719.96722	27.12518	8.1839
2	24.019	MM	0.5889	8077.43896	228.59941	91.8161

Sample Info : CHIRALPAK IF, 5% IPA:HEXANE, 1.0 mL/min, 254 nm

(*P*, *E*)-2-(2-(*tert*-Butyl)-4-styrylphenyl)-4-methylisoindoline-1,3-dione (2j)

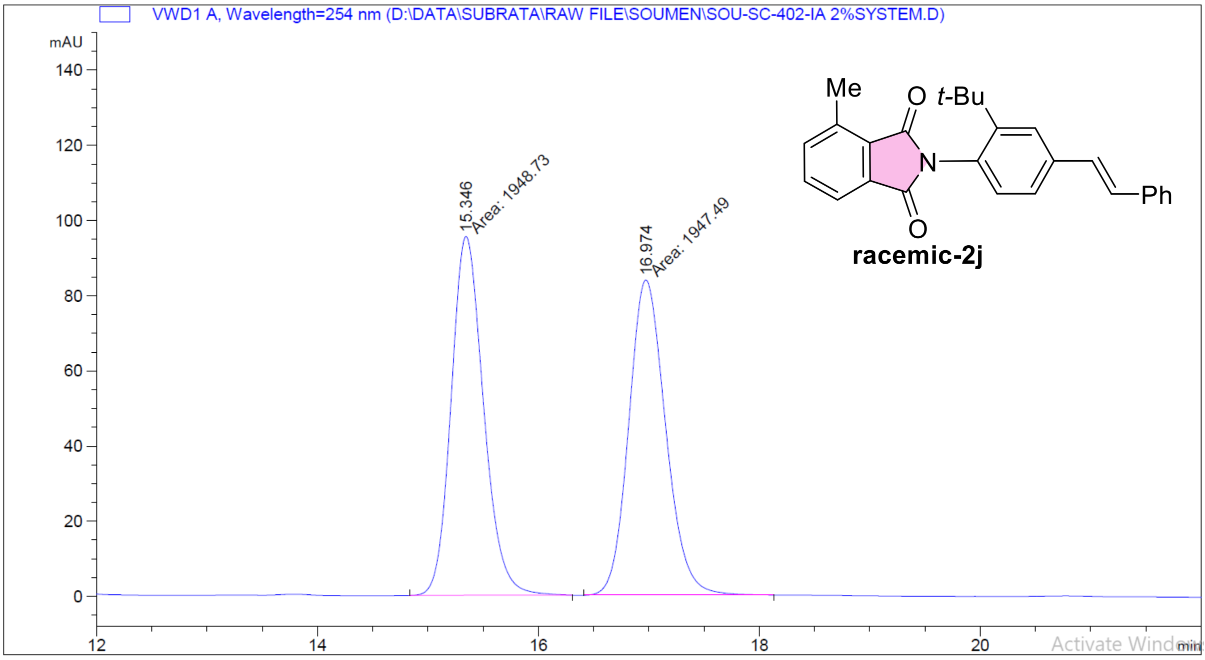

Peak #	RetTime [min]	Type	Width [min]	Area [mAU*s]	Height [mAU]	Area %
1	15.346	MM	0.3403	1948.72693	95.45241	50.0159
2	16.974	MM	0.3871	1947.48621	83.85280	49.9841

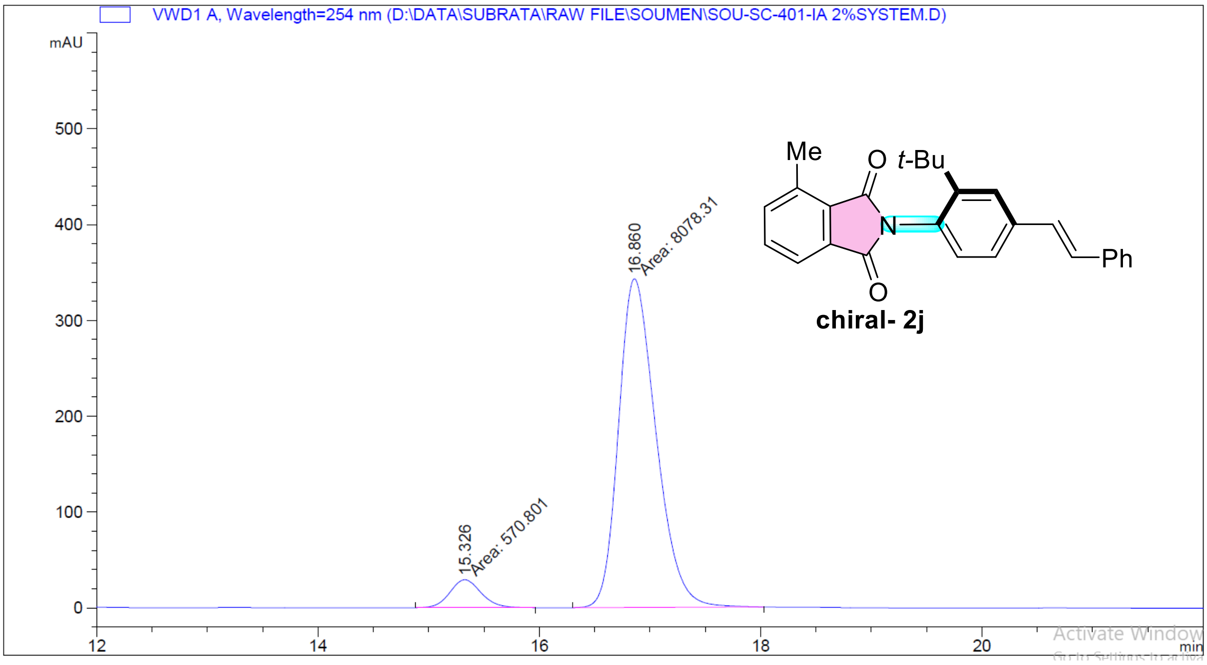

Peak #	RetTime [min]	Type	Width [min]	Area [mAU*s]	Height [mAU]	Area %
1	15.326	MM	0.3270	570.80060	29.09135	6.5995
2	16.860	MM	0.3924	8078.30859	343.11649	93.4005

Sample Info : CHIRALPAK IA, 2% IPA:HEXANE, 1.0 mL/min, 254 nm

(*P*)-Methyl-(*E*)-3-(3-(*tert*-butyl)-4-(4-methyl-1,3-dioxoisoindolin-2-yl)phenyl)acrylate (2k)

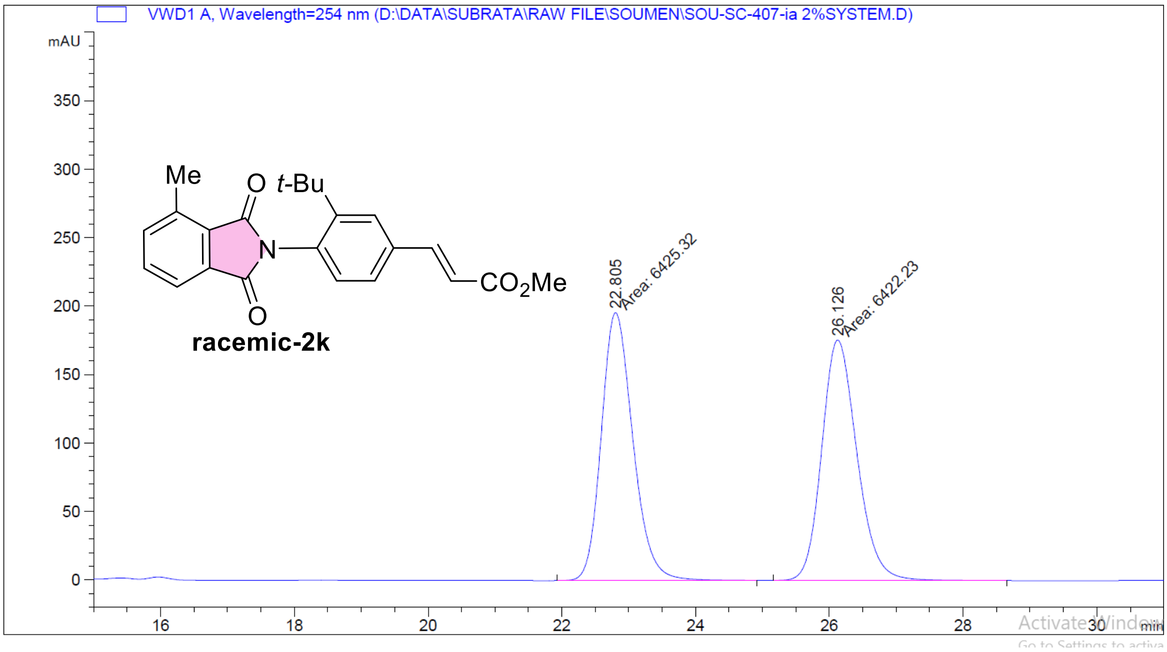

Peak #	RetTime [min]	Type	Width [min]	Area [mAU*s]	Height [mAU]	Area %
1	22.805	MM	0.5475	6425.31982	195.58173	50.0120
2	26.126	MM	0.6096	6422.23242	175.57921	49.9880

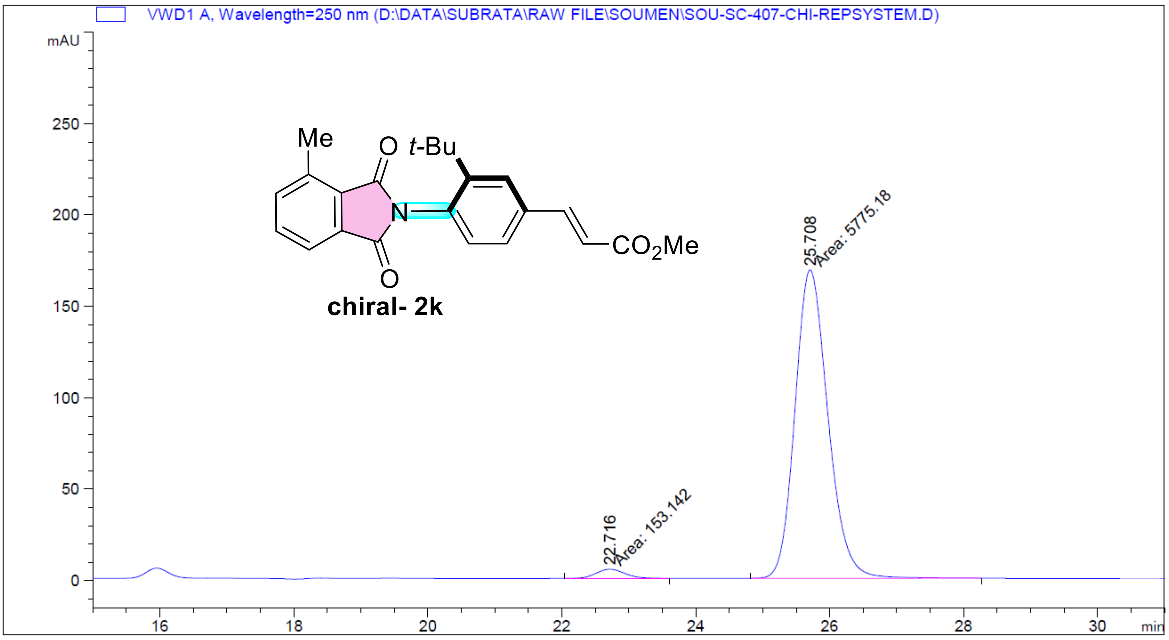

Peak #	RetTime [min]	Type	Width [min]	Area [mAU*s]	Height [mAU]	Area %
1	22.716	MM	0.5024	153.14238	5.08053	2.5832
2	25.708	MM	0.5705	5775.17822	168.70857	97.4168

Sample Info : CHIRALPAK IA, 2% IPA:HEXANE, 1.0 mL/min, 254 nm

(*P*)-2-(2-(*tert*-Butyl)-4-(phenylethynyl)phenyl)-4-methylisoindoline-1,3-dione (2l)

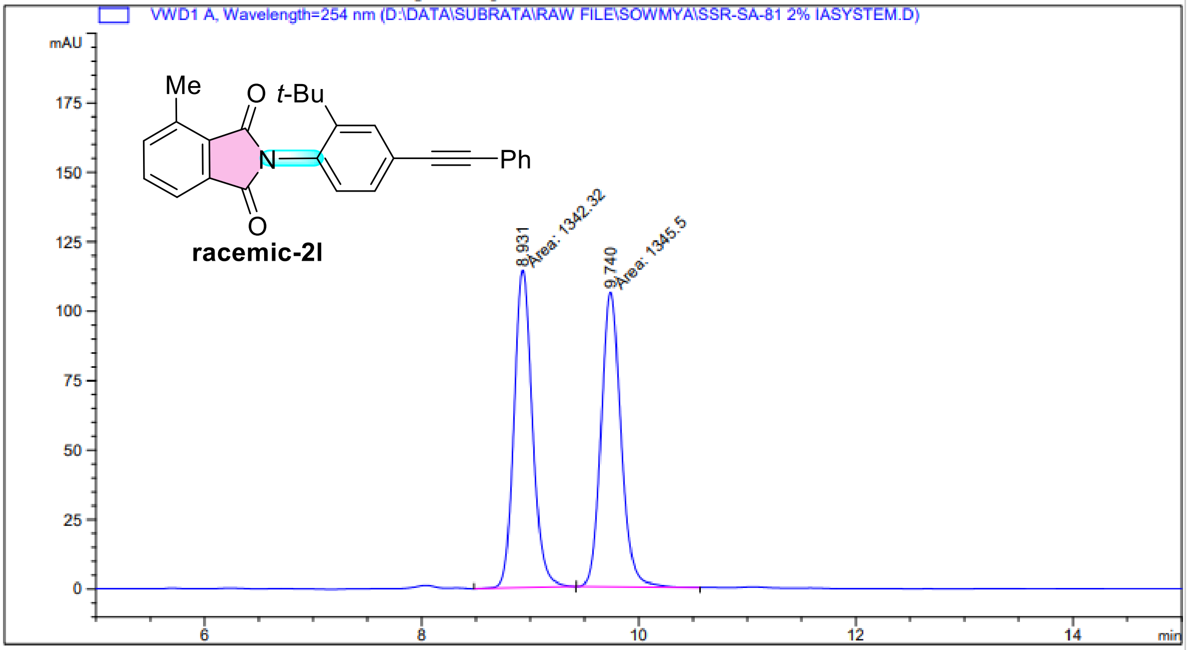

Peak #	RetTime [min]	Type	Width [min]	Area [mAU*s]	Height [mAU]	Area %
1	8.931	MM	0.1961	1342.31531	114.10629	49.9408
2	9.740	MM	0.2116	1345.49939	106.00050	50.0592

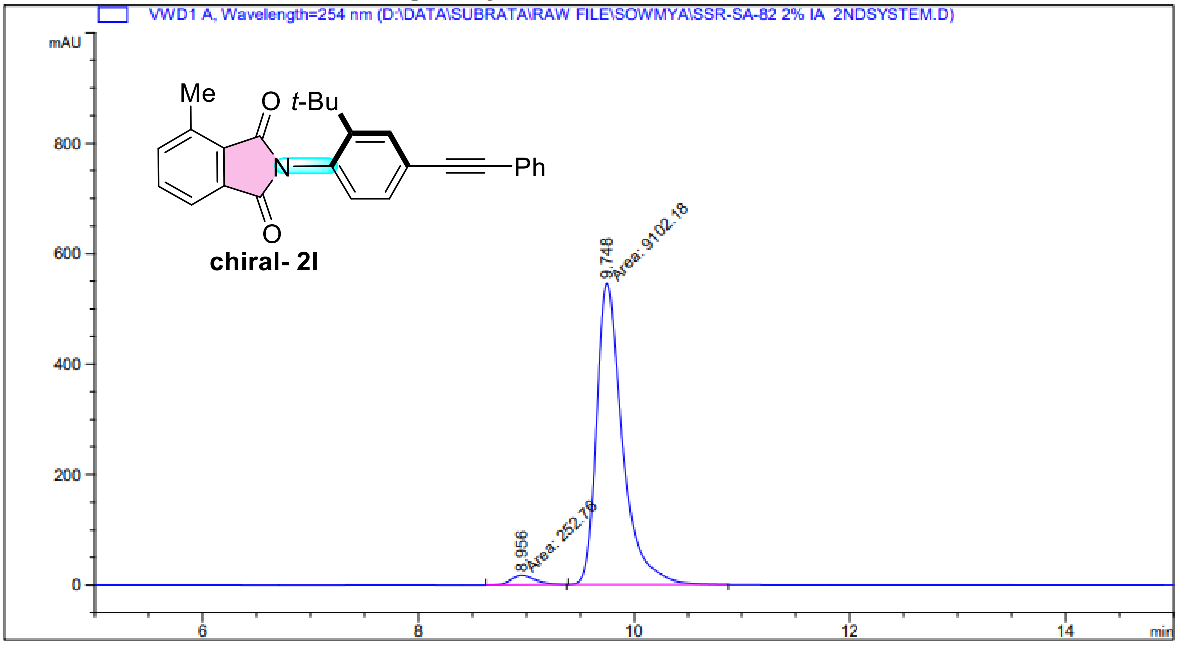

Peak #	RetTime [min]	Type	Width [min]	Area [mAU*s]	Height [mAU]	Area %
1	8.956	MM	0.2517	252.76041	16.73903	2.7019
2	9.748	MM	0.2785	9102.18359	544.73523	97.2981

Sample Info : CHIRALPAK IA, 2% IPA:HEXANE, 1.0 mL/min, 254 nm

(*P*)-2-(2-(*tert*-Butyl)-5-nitrophenyl)-4-methylisoindoline-1,3-dione (2m)

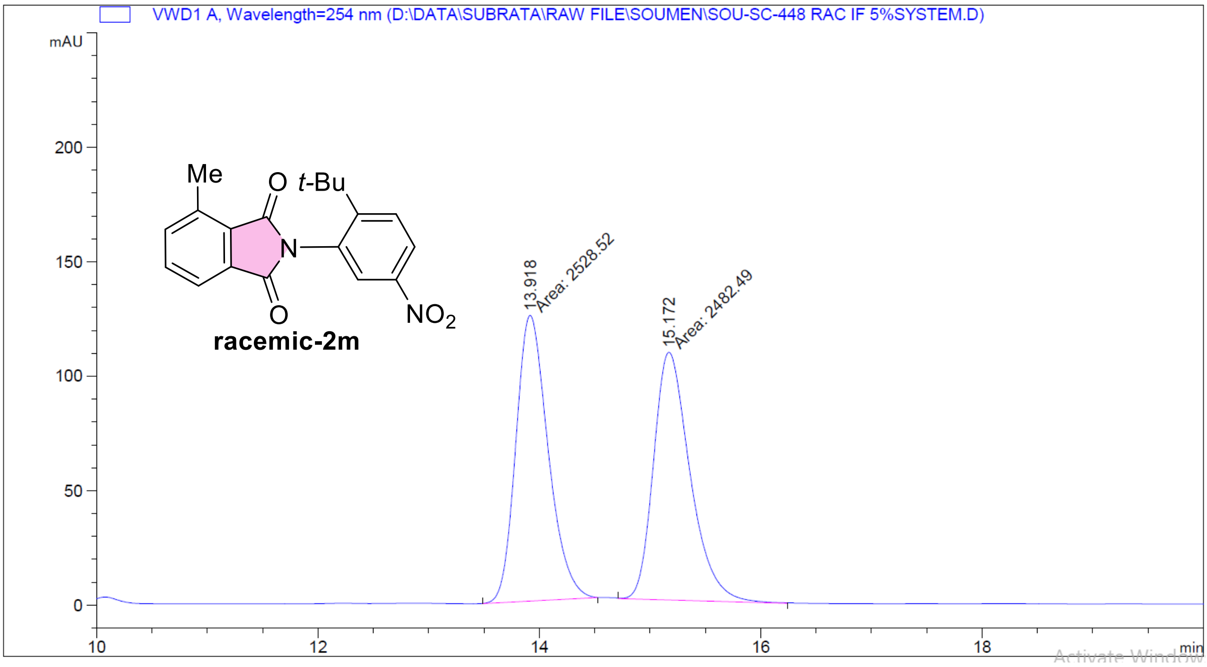

Peak #	RetTime [min]	Type	Width [min]	Area [mAU*s]	Height [mAU]	Area %
1	13.918	MM	0.3377	2528.52222	124.80212	50.4593
2	15.172	MM	0.3824	2482.49487	108.19350	49.5407

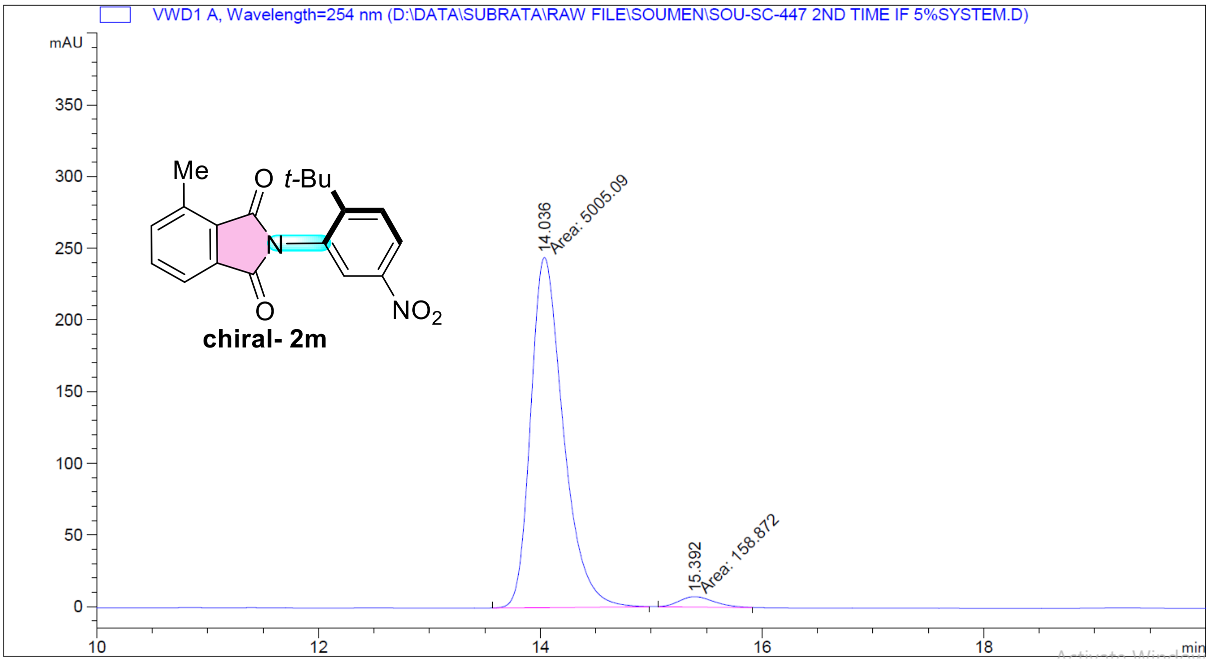

Peak #	RetTime [min]	Type	Width [min]	Area [mAU*s]	Height [mAU]	Area %
1	14.036	MM	0.3415	5005.09229	244.23552	96.9234
2	15.392	MM	0.3634	158.87206	7.28603	3.0766

Sample Info : CHIRALPAK IF, 5% IPA:HEXANE, 1.0 mL/min, 254 nm

(*P*)-4-Methyl-2-(4-methyl-2-(2-phenylpropan-2-yl)phenyl)isoindoline-1,3-dione (2n)

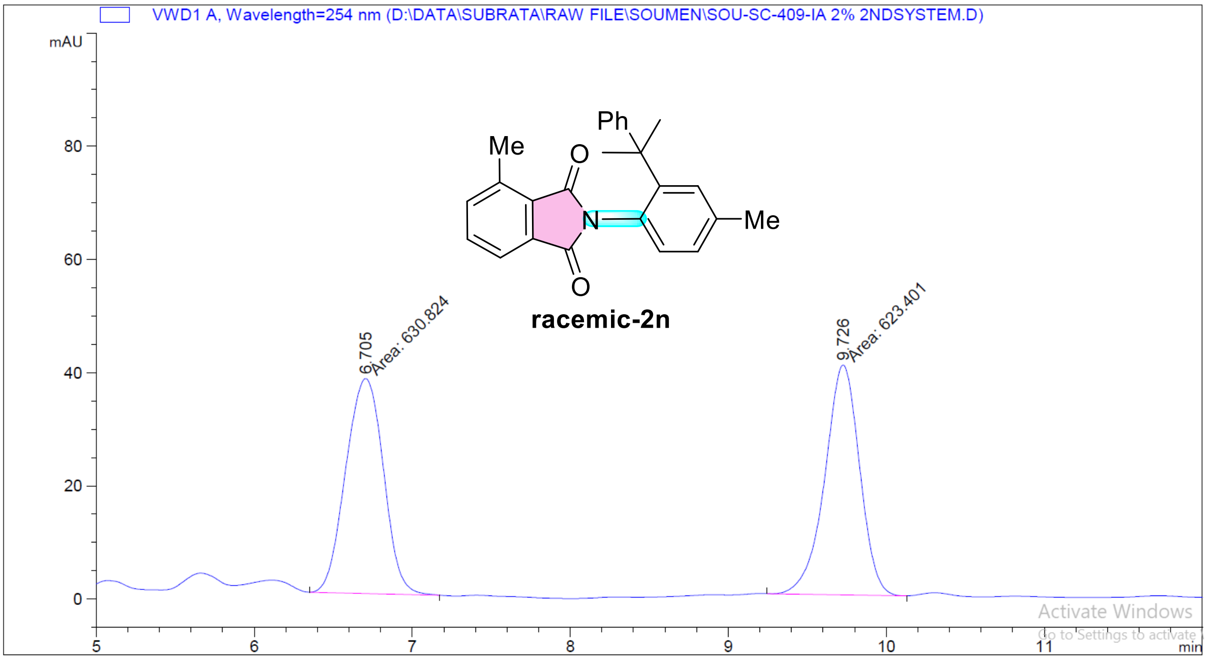

Peak #	RetTime [min]	Type	Width [min]	Area [mAU*s]	Height [mAU]	Area %
1	6.705	MM	0.2768	630.82367	37.98196	50.2959
2	9.726	MM	0.2559	623.40118	40.60841	49.7041

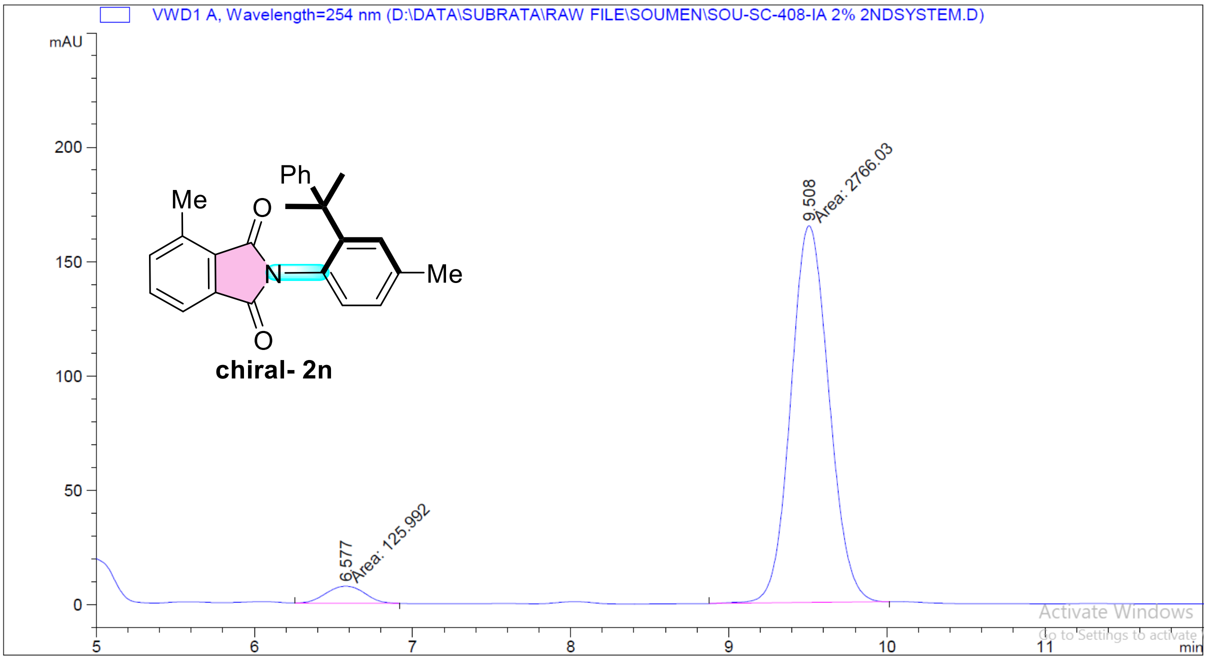

Peak #	RetTime [min]	Type	Width [min]	Area [mAU*s]	Height [mAU]	Area %
1	6.577	MM	0.2828	125.99235	7.42576	4.3566
2	9.508	MM	0.2798	2766.02856	164.77017	95.6434

Sample Info : CHIRALPAK IA, 2% IPA:HEXANE, 1.0 mL/min, 254 nm

(*P*)-2-(2-(1-Methoxy-2-methylpropan-2-yl)-4-methylphenyl)-4-methylisoindoline-1,3-dione (2o)

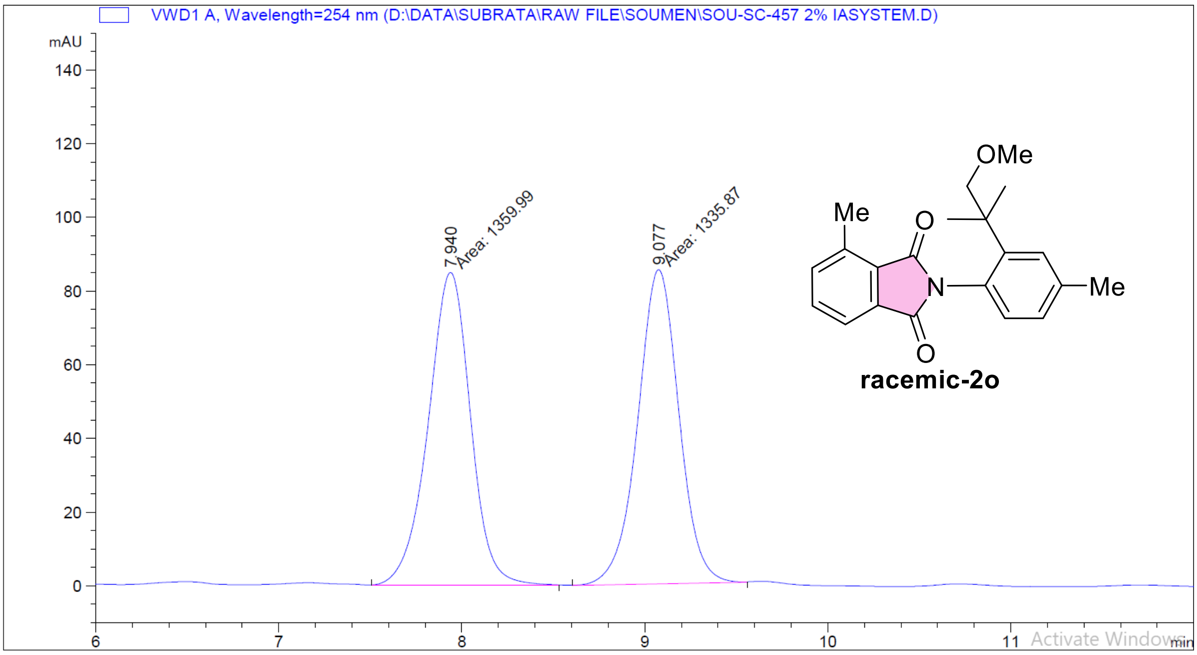

Peak #	RetTime [min]	Type	Width [min]	Area [mAU*s]	Height [mAU]	Area %
1	7.940	MM	0.2673	1359.98779	84.79097	50.4472
2	9.077	MM	0.2611	1335.87439	85.26174	49.5528

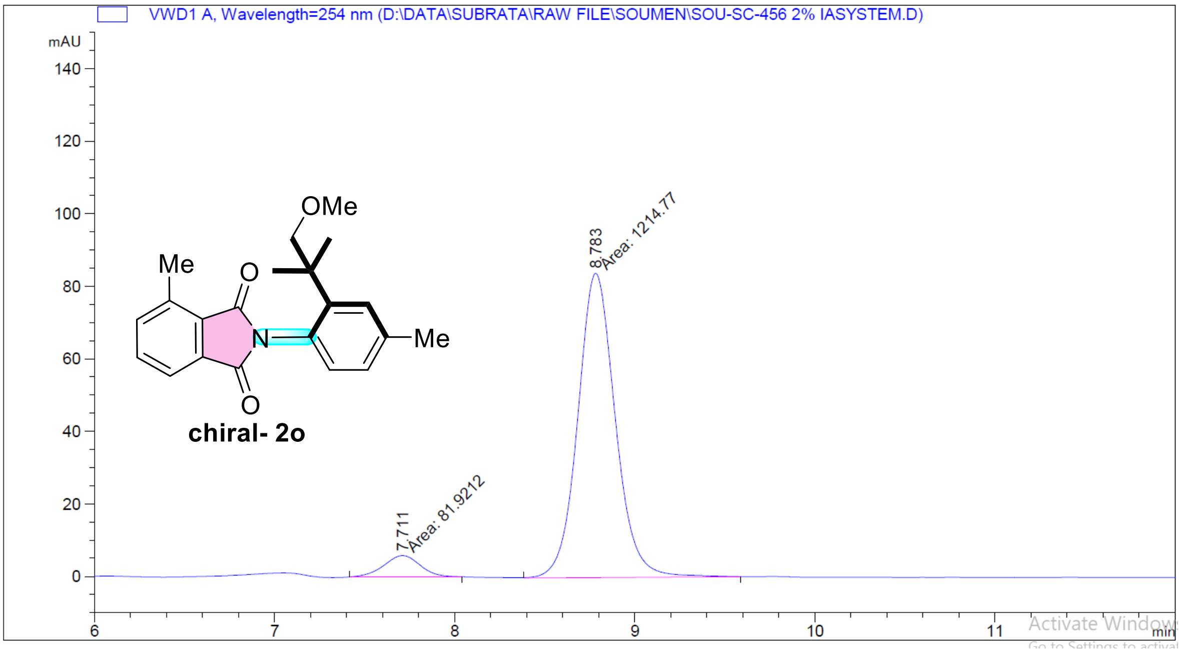

Peak #	RetTime [min]	Type	Width [min]	Area [mAU*s]	Height [mAU]	Area %
1	7.711	MM	0.2325	81.92123	5.87193	6.3177
2	8.783	MM	0.2414	1214.77185	83.87275	93.6823

Sample Info : CHIRALPAK IA, 2% IPA:HEXANE, 1mL/min, 254 nm

(*P*)-2-(2-(Methoxydiphenylmethyl)phenyl)-4-methylisoindoline-1,3-dione (2p)

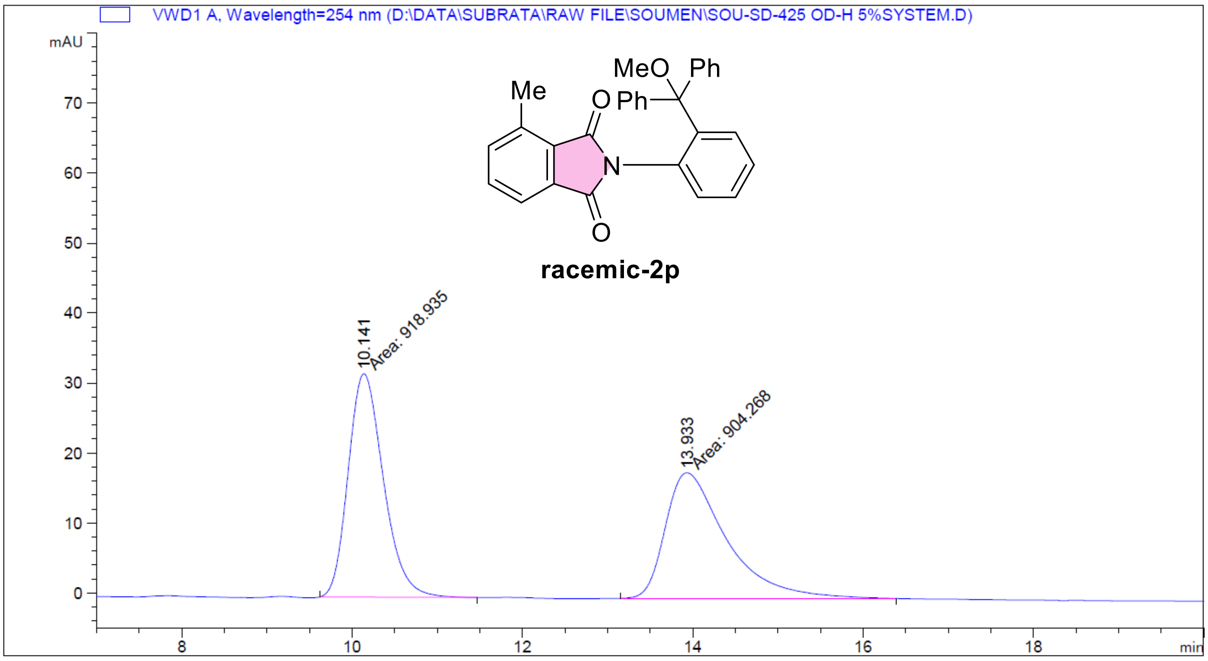

Peak #	RetTime [min]	Type	Width [min]	Area [mAU*s]	Height [mAU]	Area %
1	10.141	MM	0.4799	918.93488	31.91458	50.4022
2	13.933	MM	0.8374	904.26770	17.99833	49.5978

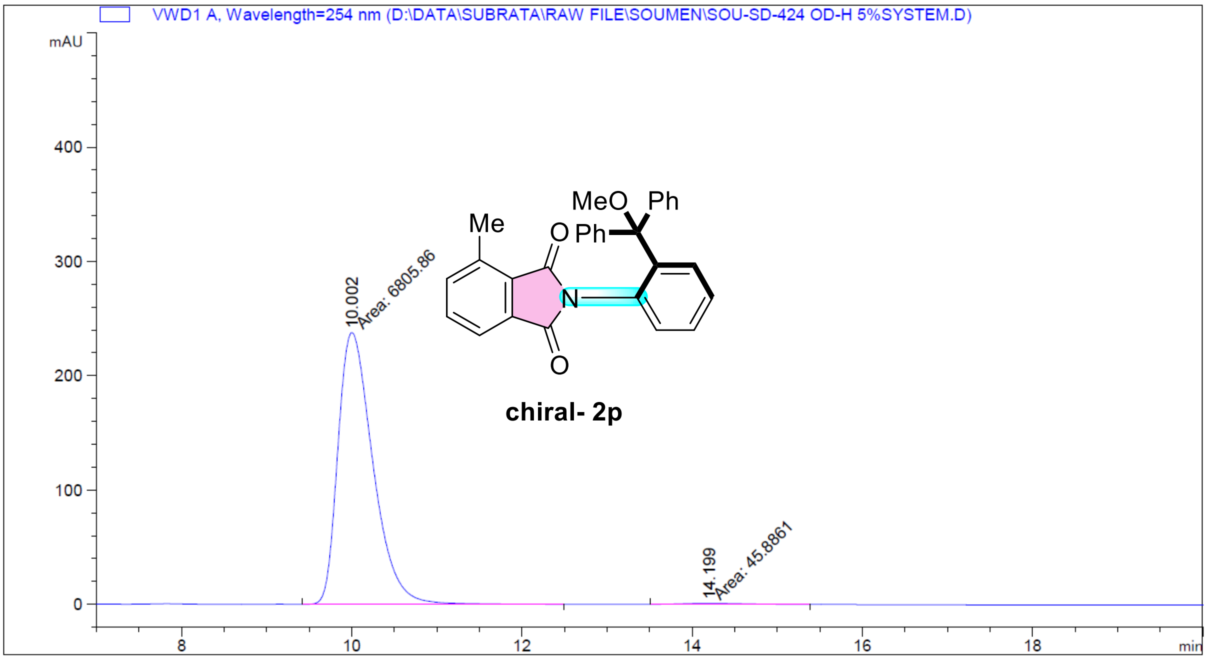

Peak #	RetTime [min]	Type	Width [min]	Area [mAU*s]	Height [mAU]	Area %
1	10.002	MM	0.4768	6805.85645	237.90344	99.3303
2	14.199	MM	0.8475	45.88609	9.02376e-1	0.6697

Sample Info : CHIRALPAK OD-H, 5% IPA-Hexane, 1.0 mL/min, 254 nm

(*P*)-2-(2-(Di(thiophen-2-yl)((trimethylsilyl)oxy)methyl)phenyl)-4-methylisoindoline-1,3-dione (2q)

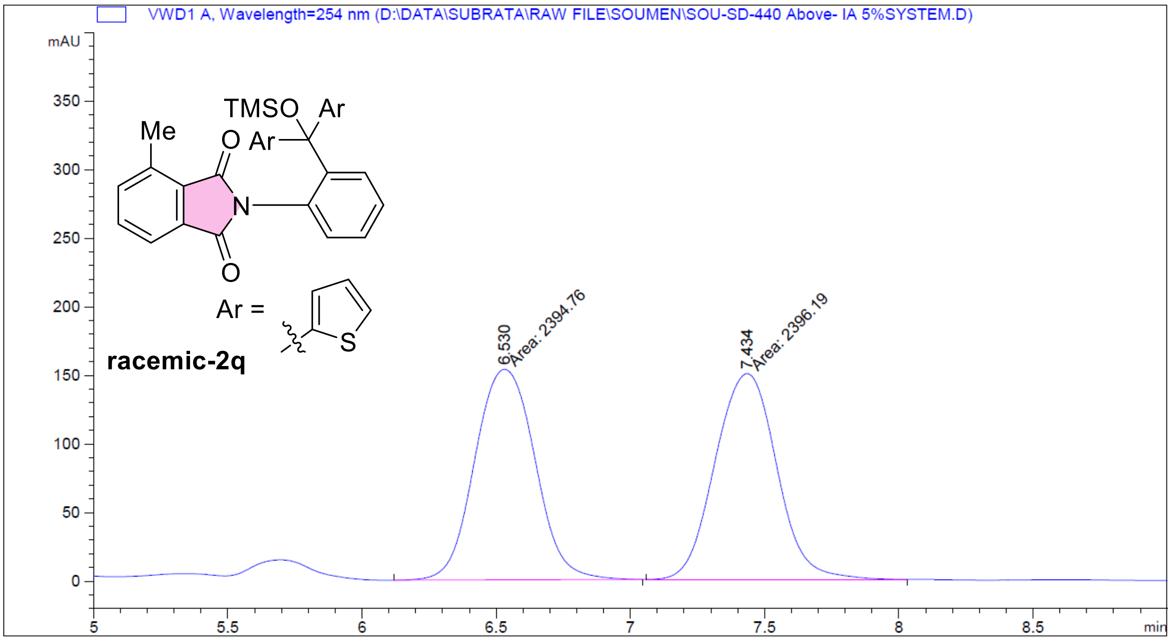

Peak #	RetTime [min]	Type	Width [min]	Area [mAU*s]	Height [mAU]	Area %
1	6.530	MM	0.2601	2394.76025	153.43054	49.9851
2	7.434	MM	0.2663	2396.19165	149.98123	50.0149

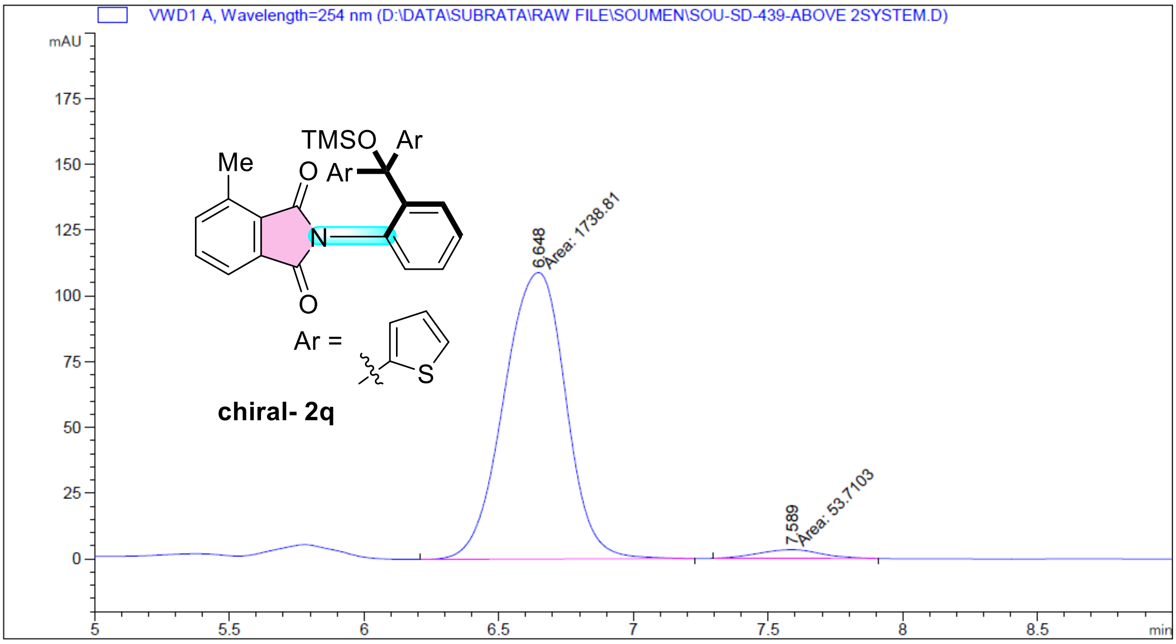

Peak #	RetTime [min]	Type	Width [min]	Area [mAU*s]	Height [mAU]	Area %
1	6.648	MM	0.2661	1738.81018	108.89382	97.0036
2	7.589	MM	0.2687	53.71027	3.33162	2.9964

Sample Info : CHIRALPAK IA, 5% IPA-Hexane, 1.0 mL/min, 254 nm

(*P*)-4-Methyl-2-(2-(phenylsulfonyl)phenyl)isoindoline-1,3-dione (2r)

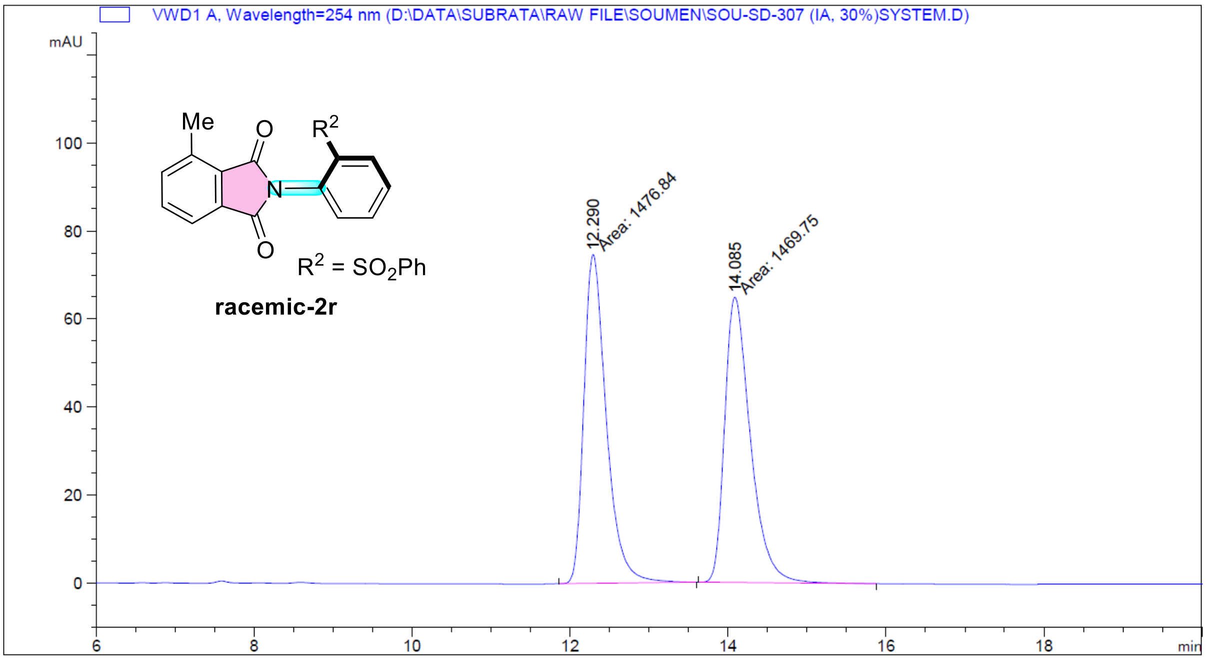

Peak #	RetTime [min]	Type	Width [min]	Area [mAU*s]	Height [mAU]	Area %
1	12.290	MM	0.3294	1476.83997	74.73285	50.1203
2	14.085	MM	0.3778	1469.75305	64.83873	49.8797

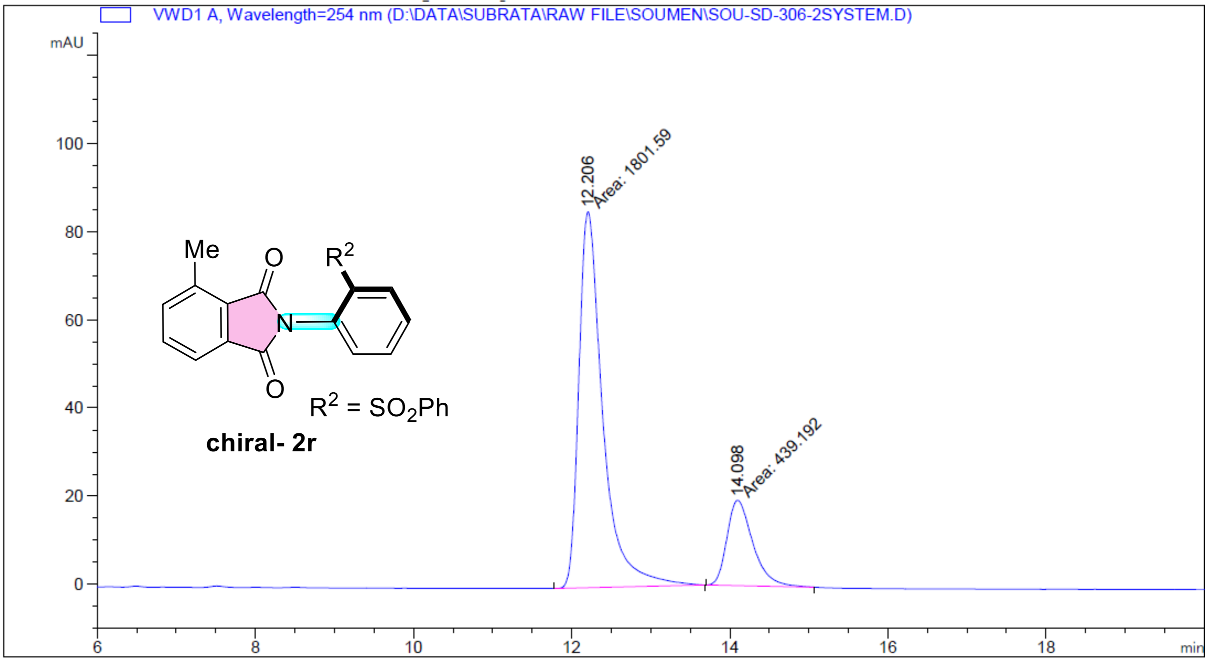

Peak #	RetTime [min]	Type	Width [min]	Area [mAU*s]	Height [mAU]	Area %
1	12.206	MM	0.3521	1801.59094	85.28042	80.4001
2	14.098	MM	0.3789	439.19214	19.31852	19.5999

Sample Info : CHIRALPAK IA, 30% IPA-HEXANE, 1.0 mL/min, 254 nm

(*P*)-2-(2-(*tert*-Butyl)phenyl)-4-methoxyisoindoline-1,3-dione (2s)

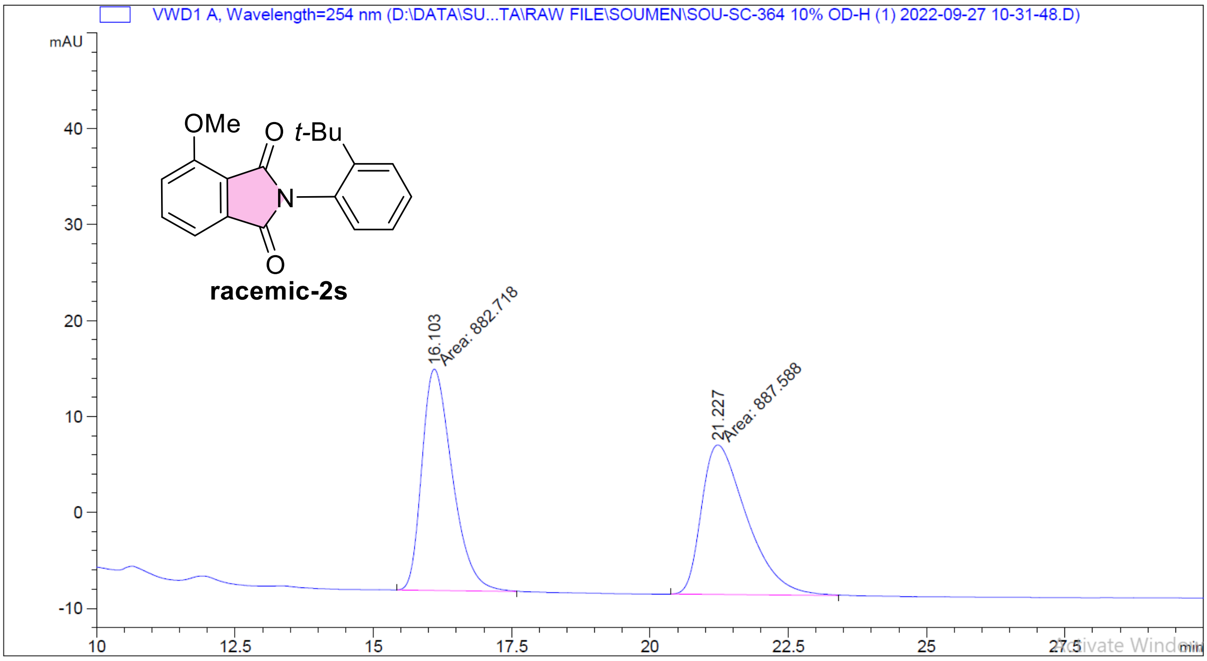

Peak #	RetTime [min]	Type	Width [min]	Area [mAU*s]	Height [mAU]	Area %
1	16.103	MM	0.6377	882.71777	23.07106	49.8624
2	21.227	MM	0.9497	887.58795	15.57708	50.1376

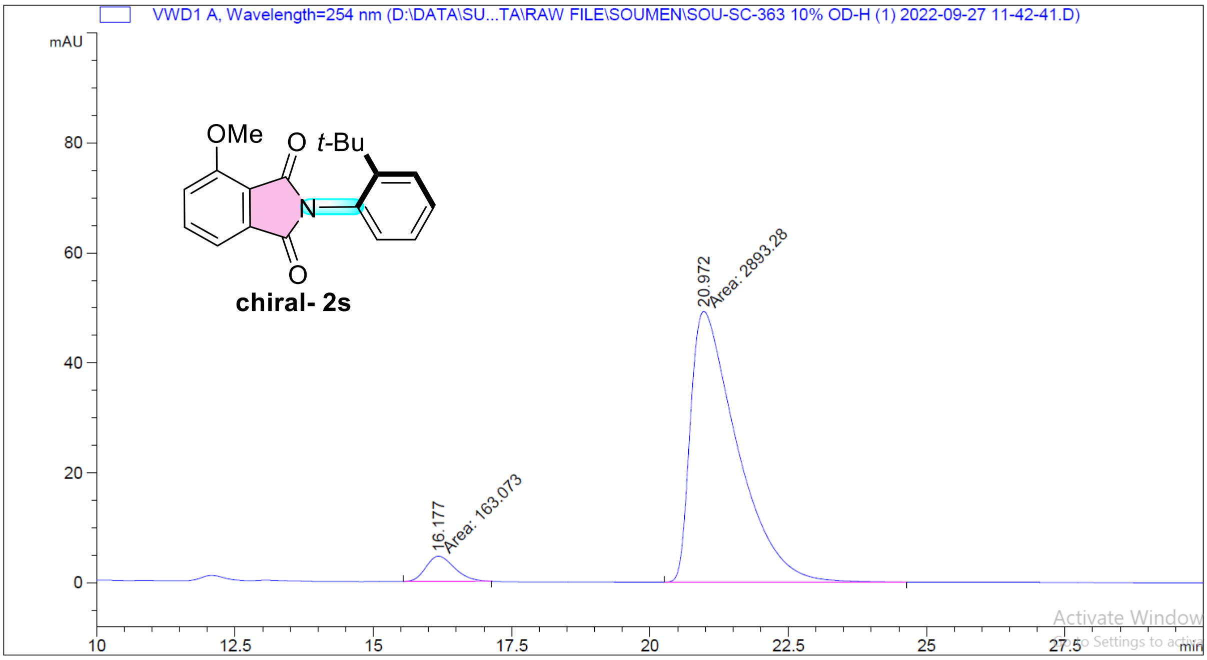

Peak #	RetTime [min]	Type	Width [min]	Area [mAU*s]	Height [mAU]	Area %
1	16.177	MM	0.5945	163.07344	4.57166	5.3356
2	20.972	MM	0.9792	2893.28198	49.24765	94.6644

Sample Info : CHIRALCELL OD-H, 10% IPA:HEXANE, 1.0 mL/min, 254 nm

(*P*)-4-(Benzyloxy)-2-(2-(*tert*-butyl)phenyl)isoindoline-1,3-dione (2t)

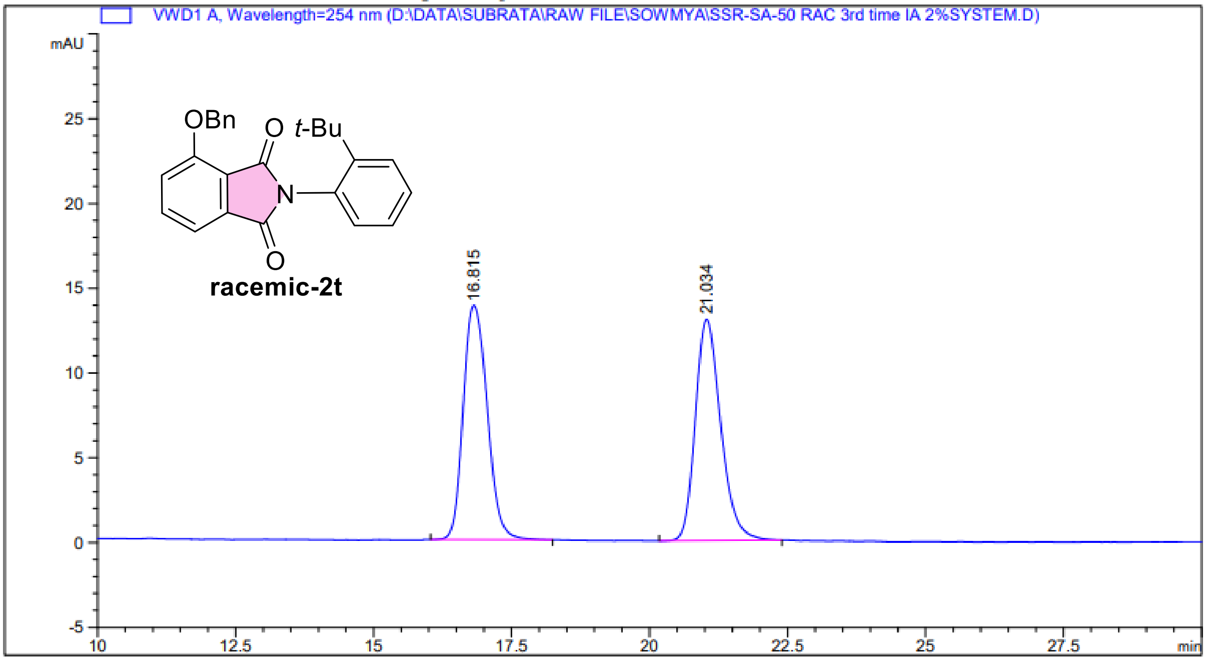

Peak #	RetTime [min]	Type	Width [min]	Area [mAU*s]	Height [mAU]	Area %
1	16.815	BB	0.4841	418.29794	13.81976	49.9430
2	21.034	BB	0.4894	419.25220	13.06344	50.0570

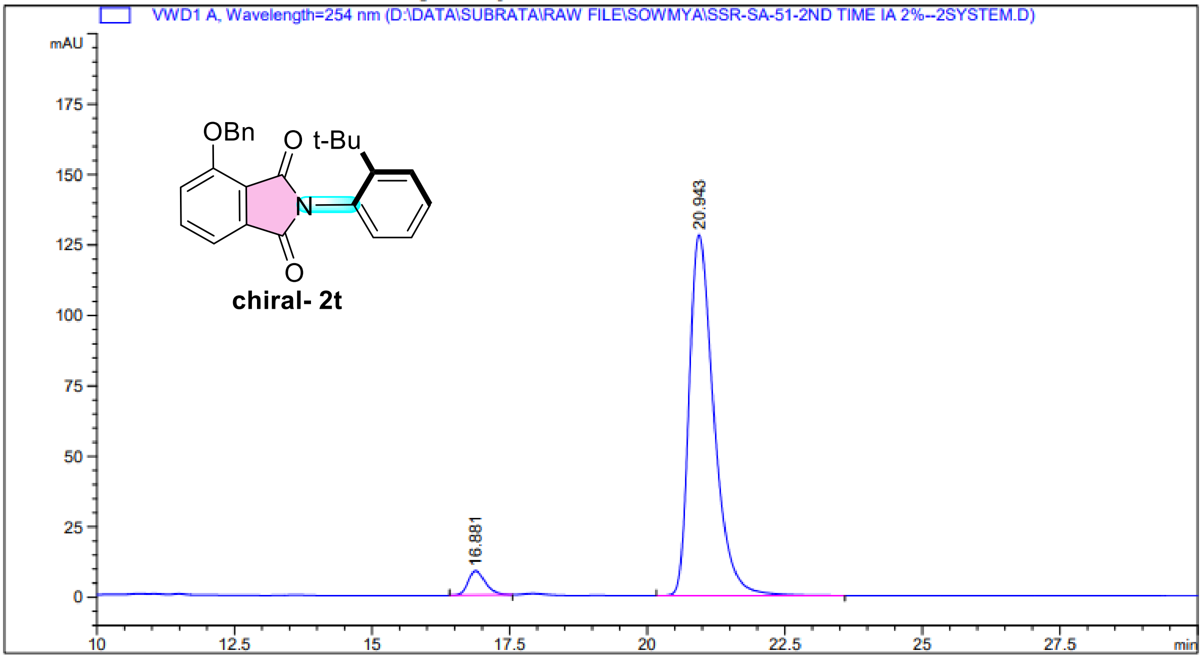

Peak #	RetTime [min]	Type	Width [min]	Area [mAU*s]	Height [mAU]	Area %
1	16.881	BB	0.3309	186.66551	8.61196	4.4740
2	20.943	BB	0.4750	3985.58936	128.09442	95.5260

Sample Info : CHIRALPAK IA, 2% IPA:HEXANE, 1.0 mL/min, 254 nm

(*P*)-4-(Allyloxy)-2-(2-(*tert*-butyl)phenyl)isoindoline-1,3-dione (2u)

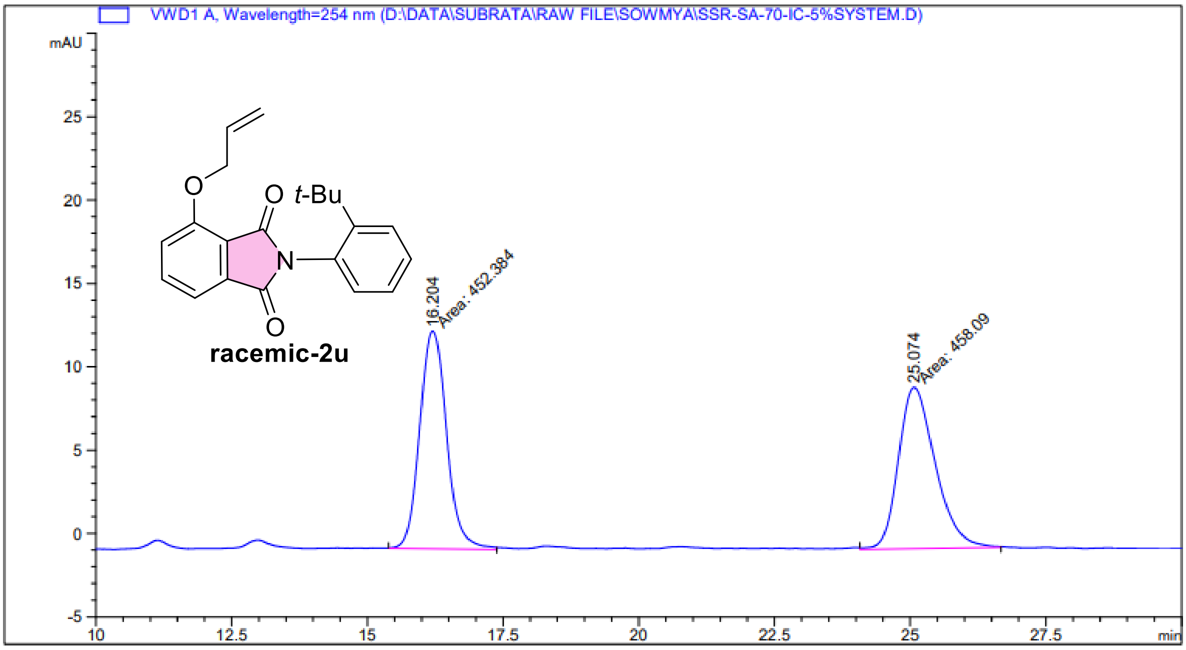

Peak #	RetTime [min]	Type	Width [min]	Area [mAU*s]	Height [mAU]	Area %
1	16.204	MM	0.5766	452.38419	13.07597	49.6867
2	25.074	MM	0.7888	458.08990	9.67850	50.3133

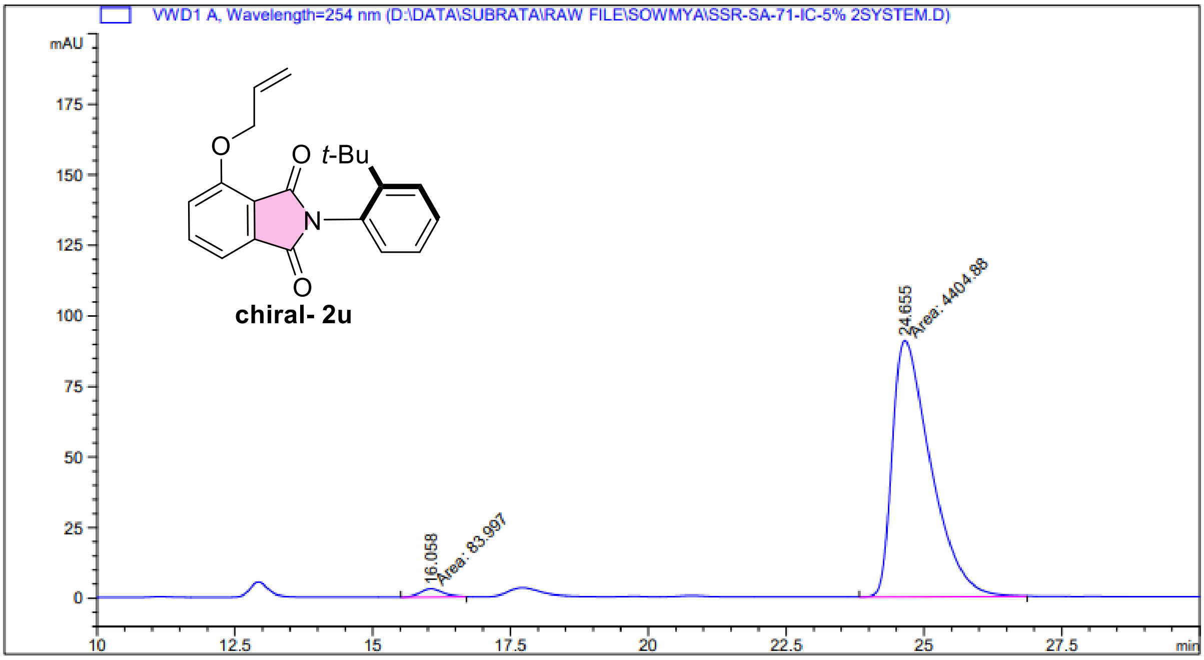

Peak #	RetTime [min]	Type	Width [min]	Area [mAU*s]	Height [mAU]	Area %
1	16.058	MM	0.4784	83.99697	2.92617	1.8712
2	24.655	MM	0.8090	4404.88330	90.74216	98.1288

Sample Info : CHIRALPAK IC, 5% IPA:HEXANE, 1.0 mL/min, 254 nm

(*P*)- 2-(2-(*tert*-Butyl)phenyl)-1,3-dioxoisoindolin-4-yl pivalate (2v)

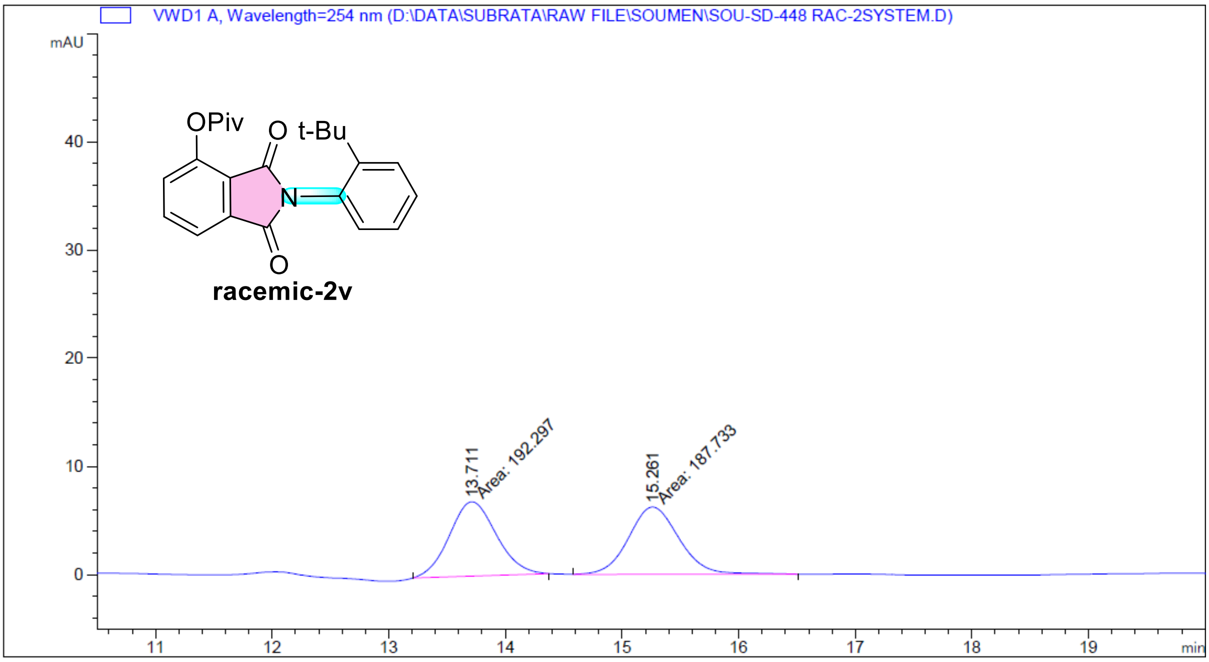

Peak #	RetTime [min]	Type	Width [min]	Area [mAU*s]	Height [mAU]	Area %
1	13.711	MM	0.4668	192.29721	6.86614	50.6004
2	15.261	MM	0.5031	187.73349	6.21976	49.3996

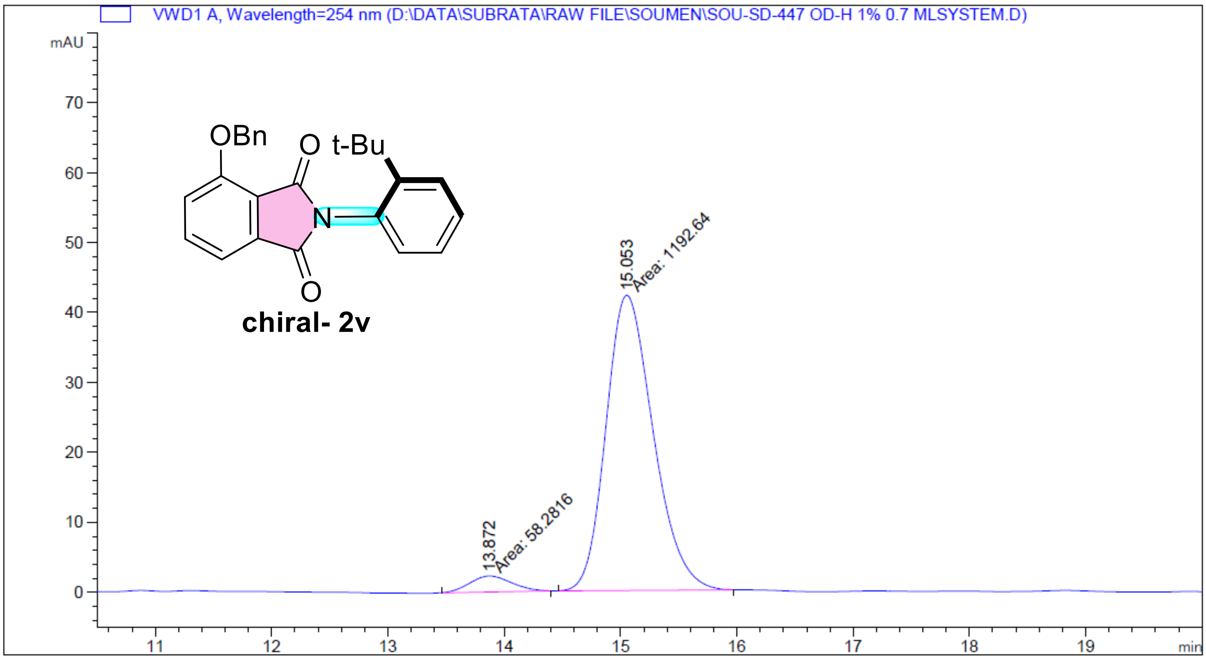

Peak #	RetTime [min]	Type	Width [min]	Area [mAU*s]	Height [mAU]	Area %
1	13.872	MM	0.4214	58.28156	2.30489	4.6591
2	15.053	MM	0.4710	1192.64197	42.20625	95.3409

Sample Info : CHIRALCELL OD-H, 1% IPA-Hexane, 0.7 mL/min, 254 nm

(*P*)-2-(2-(*tert*-Butyl)phenyl)-4-chloroisoindoline-1,3-dione (2w)

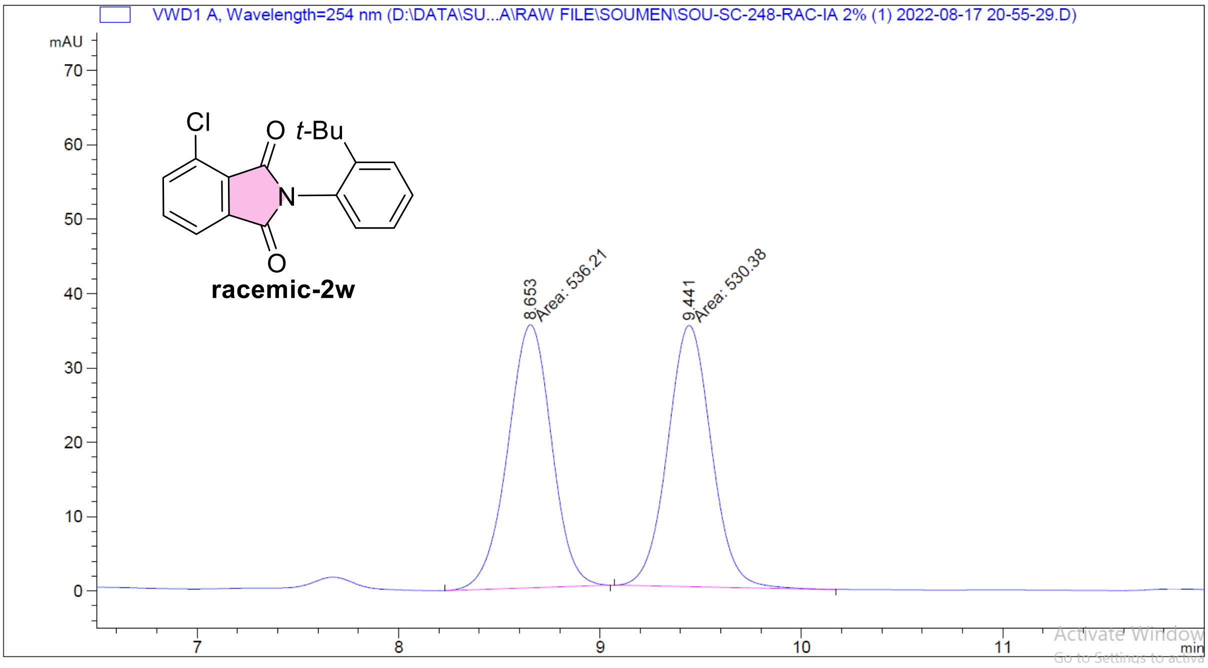

Peak #	RetTime [min]	Type	Width [min]	Area [mAU*s]	Height [mAU]	Area %
1	8.653	MM	0.2527	536.20966	35.35997	50.2733
2	9.441	MM	0.2516	530.37988	35.13015	49.7267

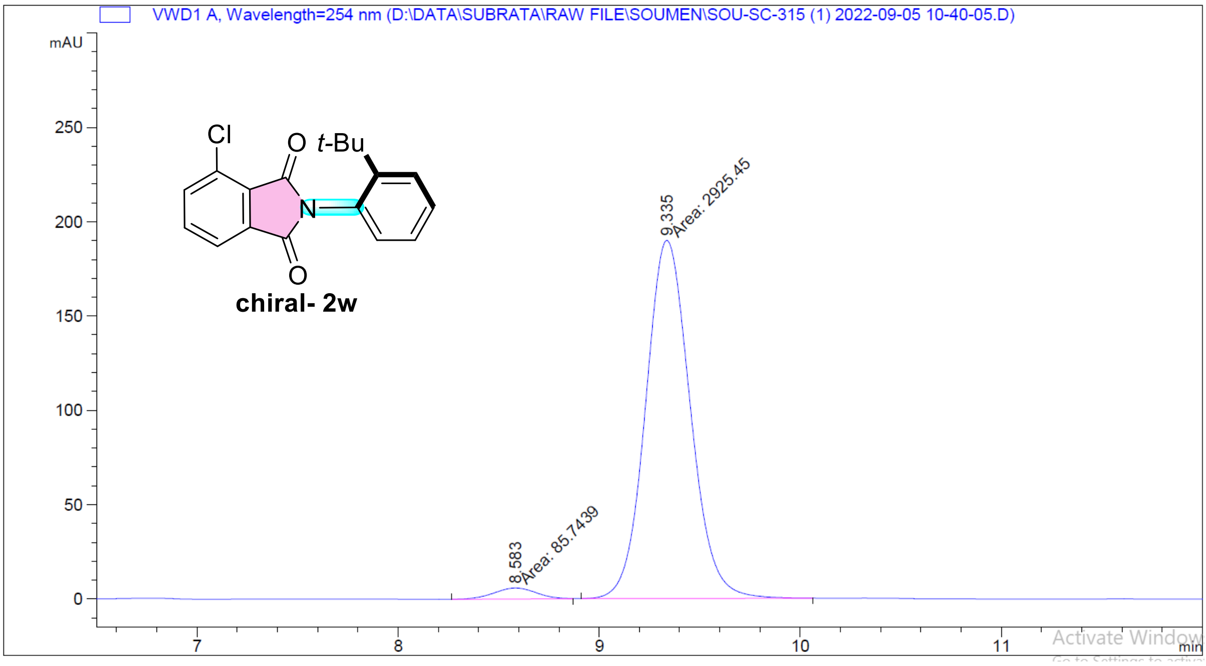

Peak #	RetTime [min]	Type	Width [min]	Area [mAU*s]	Height [mAU]	Area %
1	8.583	MM	0.2449	85.74387	5.83497	2.8475
2	9.335	MM	0.2568	2925.44727	189.85373	97.1525

Sample Info : CHIRALPAK IA, 2% IPA:HEXANE, 1.0 mL/min, 254 nm

(*P*)-4-Bromo-2-(2-(*tert*-butyl)phenyl)isoindoline-1,3-dione (2x)

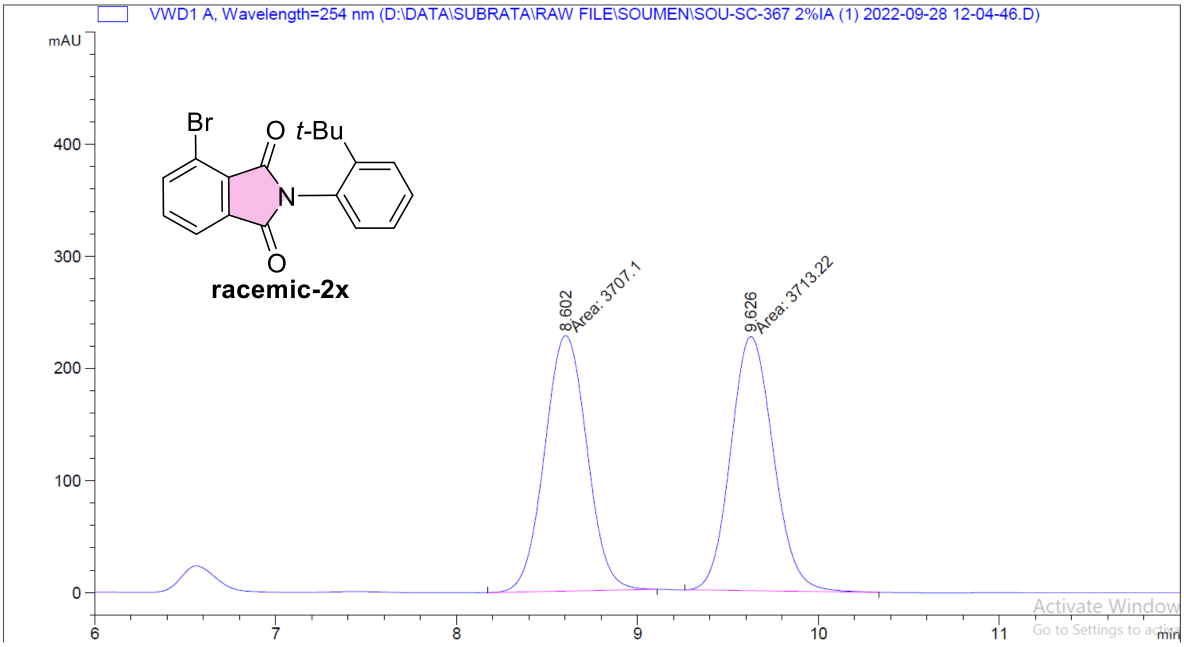

Peak #	RetTime [min]	Type	Width [min]	Area [mAU*s]	Height [mAU]	Area %
1	8.602	MM	0.2711	3707.10034	227.89015	49.9587
2	9.626	MM	0.2730	3713.22363	226.68172	50.0413

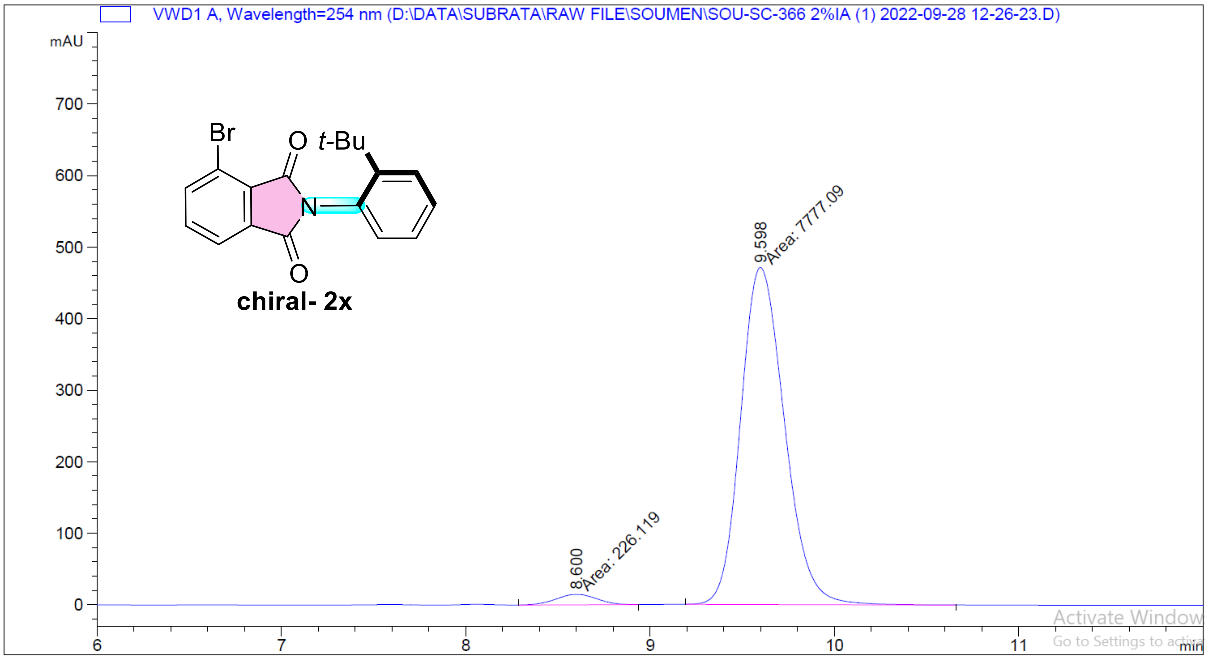

Peak #	RetTime [min]	Type	Width [min]	Area [mAU*s]	Height [mAU]	Area %
1	8.600	MM	0.2566	226.11894	14.68499	2.8254
2	9.598	MM	0.2752	7777.08838	470.91446	97.1746

Sample Info : CHIRALPAK IA, 2% IPA:HEXANE, 1.0 mL/min, 254 nm

(*P*)-2-(2-(*tert*-Butyl)phenyl)-4-nitroisoindoline-1,3-dione (2y)

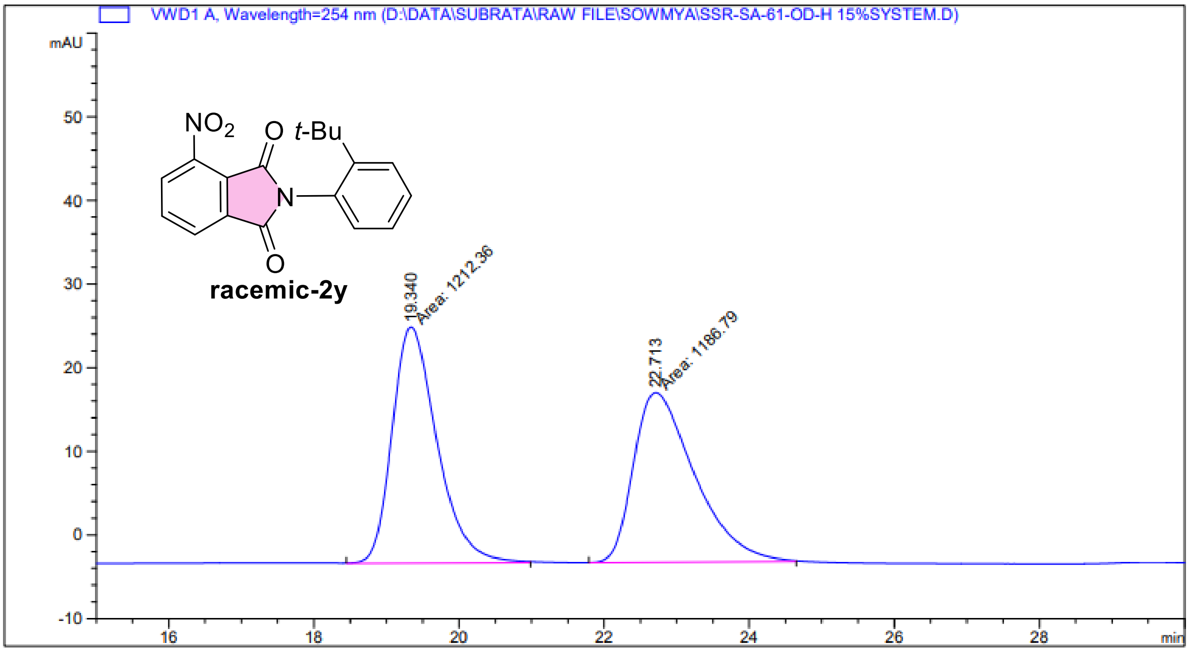

Peak #	RetTime [min]	Type	Width [min]	Area [mAU*s]	Height [mAU]	Area %
1	19.340	MM	0.7163	1212.35803	28.21025	50.5329
2	22.713	MM	0.9746	1186.78992	20.29467	49.4671

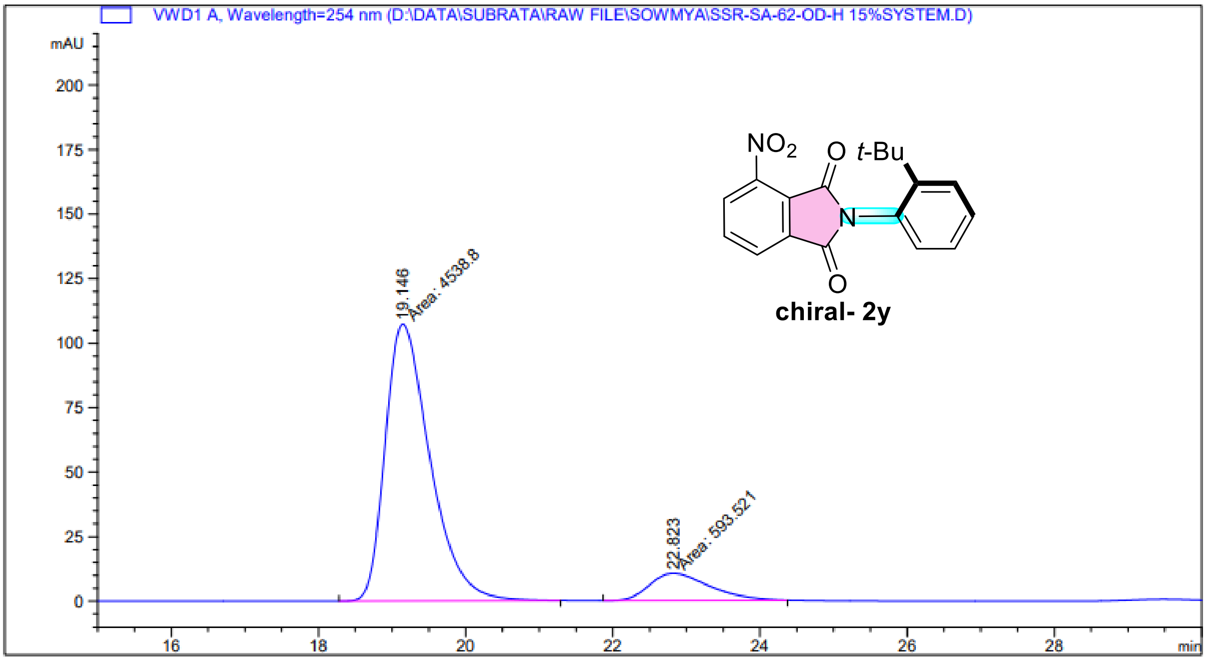

Peak #	RetTime [min]	Type	Width [min]	Area [mAU*s]	Height [mAU]	Area %
1	19.146	MM	0.7050	4538.80127	107.29459	88.4356
2	22.823	MM	0.9415	593.52106	10.50711	11.5644

Sample Info : CHIRALCEL OD-H, 15% IPA:HEXANE, 1.0 mL/min, 254 nm

(*P*)-2-(2-(*tert*-Butyl)phenyl)-1*H*-benzo[e]isoindole-1,3(2*H*)-dione (2z)

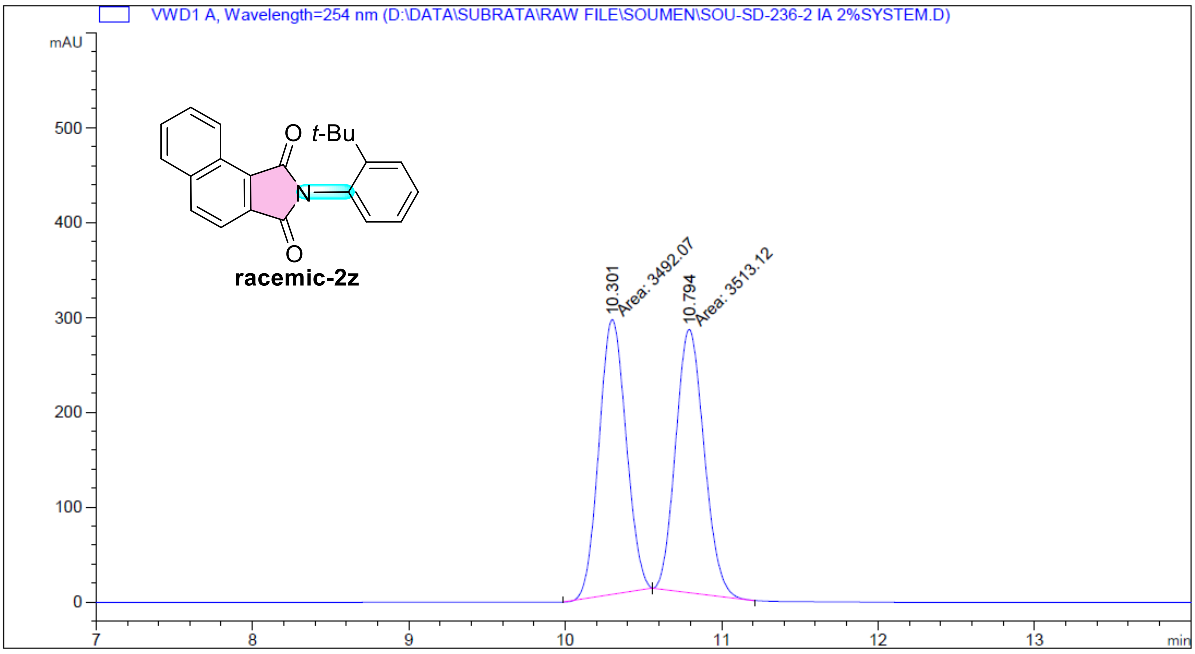

Peak #	RetTime [min]	Type	Width [min]	Area [mAU*s]	Height [mAU]	Area %
1	10.301	MM	0.2008	3492.07080	289.82520	49.8497
2	10.794	MM	0.2110	3513.12305	277.45319	50.1503

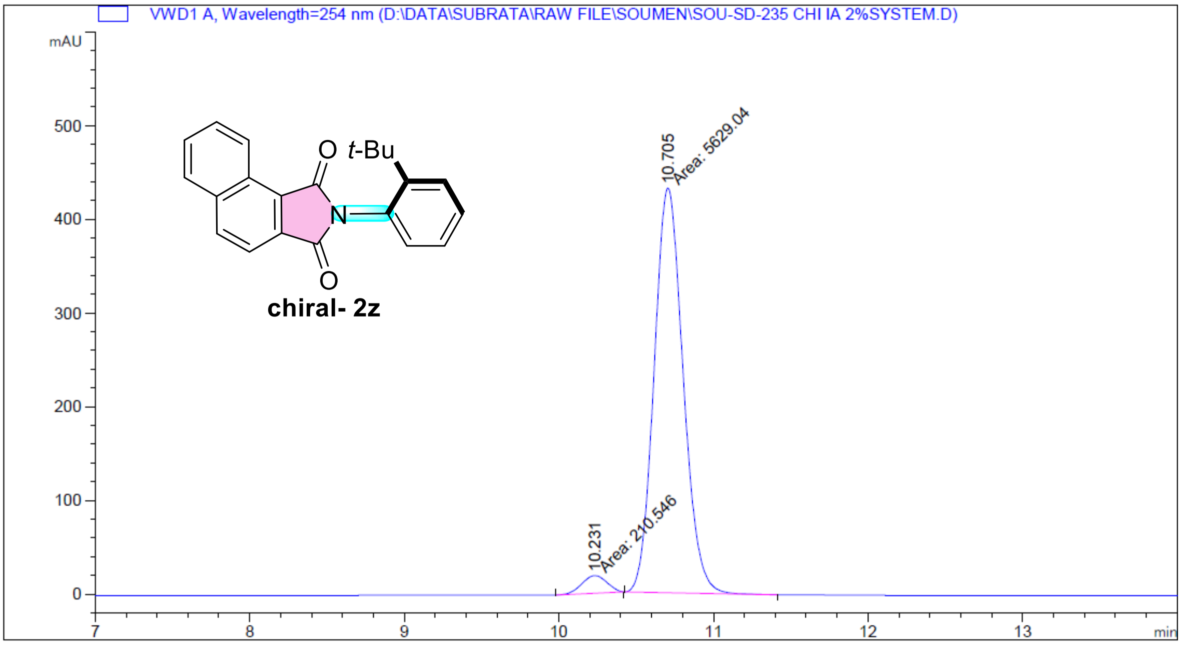

Peak #	RetTime [min]	Type	Width [min]	Area [mAU*s]	Height [mAU]	Area %
1	10.231	MM	0.1865	210.54604	18.81774	3.6055
2	10.705	MM	0.2172	5629.03564	431.98587	96.3945

Sample Info : CHIRALPAK IA, 2% IPA:HEXANE, 1.0 mL/min, 254 nm

(*P*)-2-(2-(*tert*-Butyl)phenyl)-4-methoxy-5-nitroisoindoline-1,3-dione (2aa)

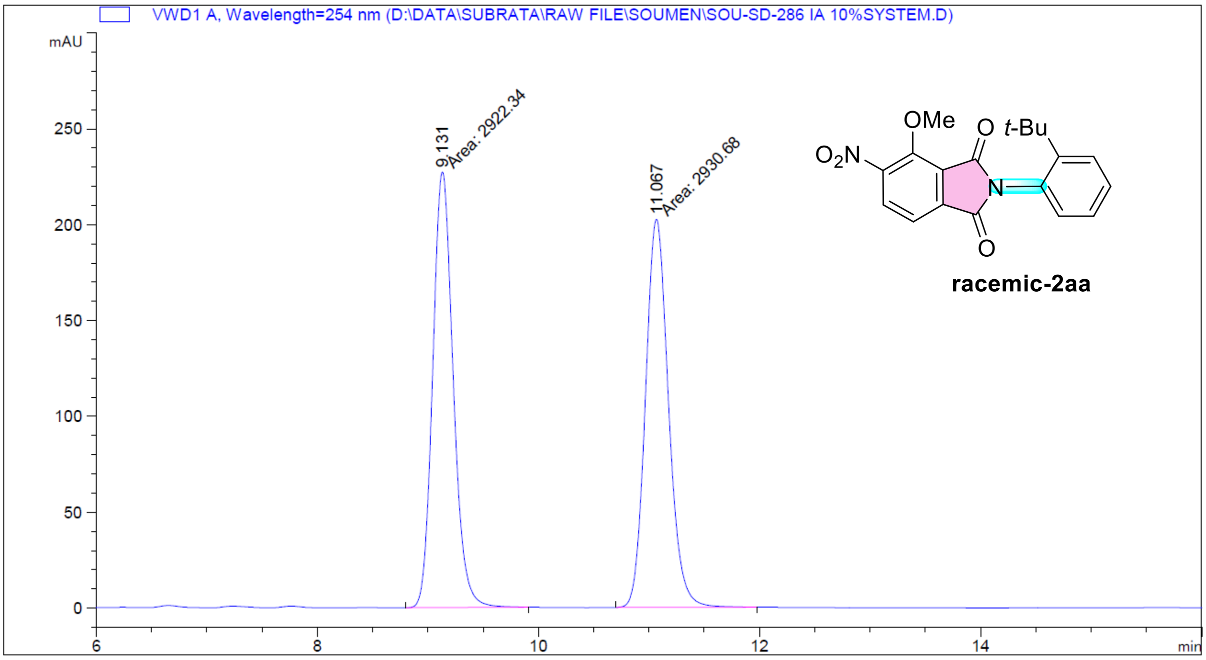

Peak #	RetTime [min]	Type	Width [min]	Area [mAU*s]	Height [mAU]	Area %
1	9.131	MM	0.2143	2922.33984	227.26608	49.9288
2	11.067	MM	0.2412	2930.67725	202.47787	50.0712

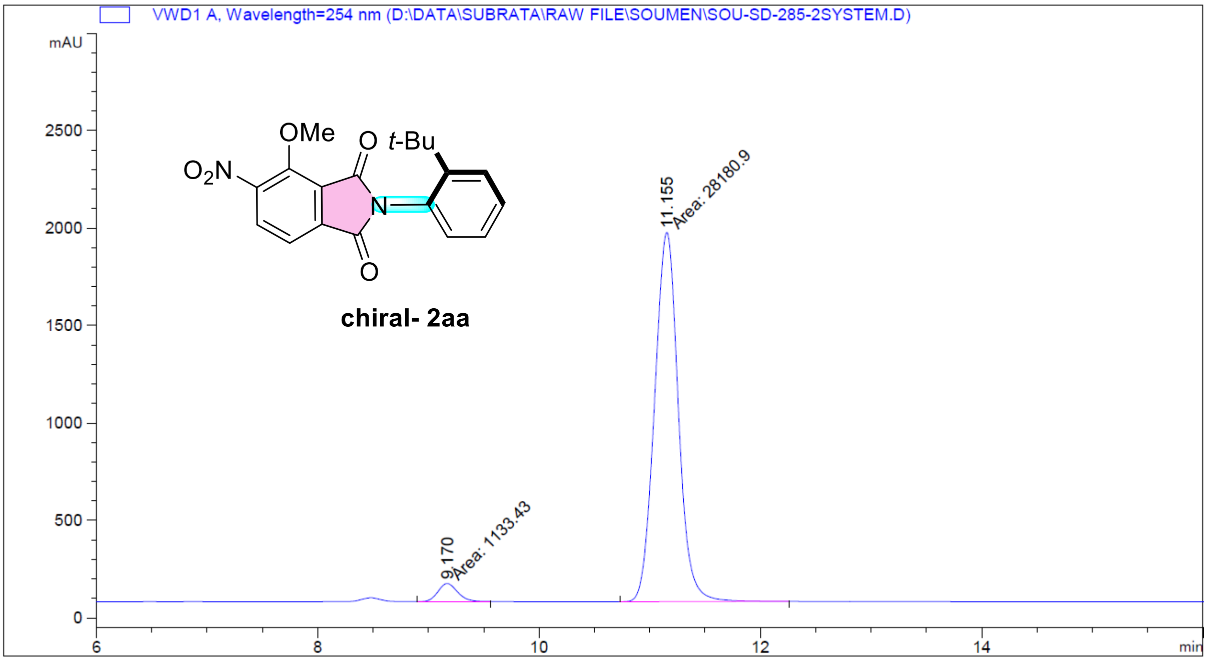

Peak #	RetTime [min]	Type	Width [min]	Area [mAU*s]	Height [mAU]	Area %
1	9.170	MM	0.2048	1133.42798	92.24187	3.8665
2	11.155	MM	0.2480	2.81809e4	1893.53357	96.1335

Sample Info : CHIRALCELL OD-H , 10% IPA-HEXANE, 1.0 mL/min, 254 nm

(*P*)-2-(2-(*tert*-Butyl)phenyl)-4-methoxy-7-nitroisoindoline-1,3-dione (2ab)

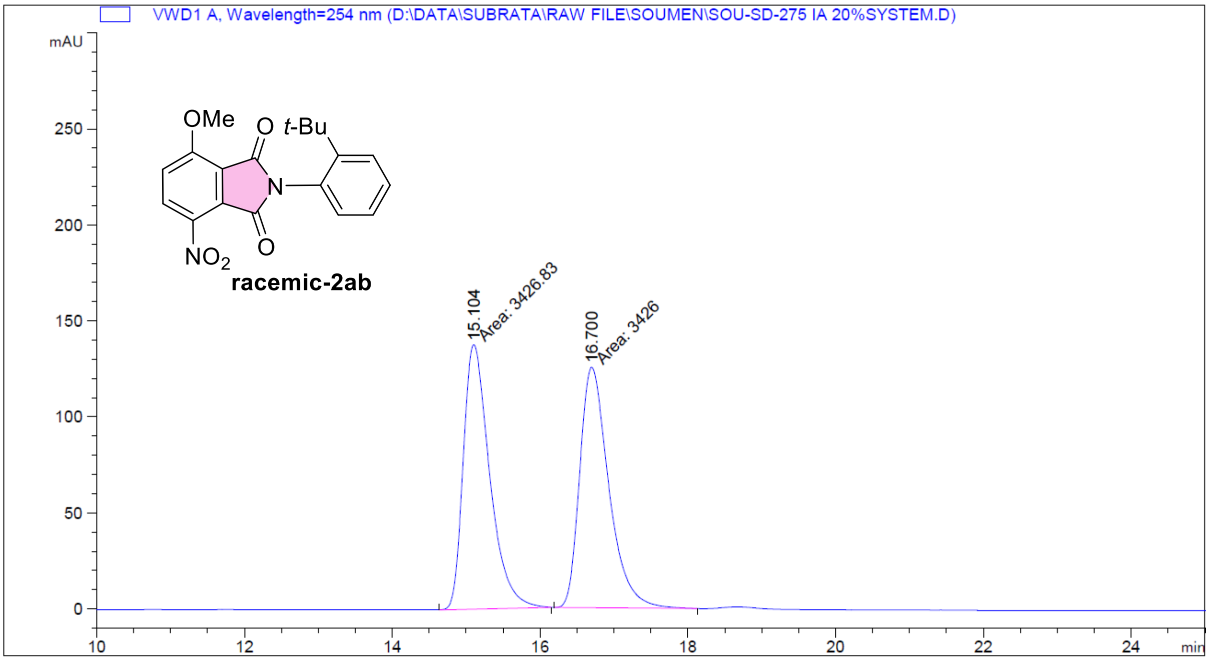

Peak #	RetTime [min]	Type	Width [min]	Area [mAU*s]	Height [mAU]	Area %
1	15.104	MM	0.4146	3426.83154	137.74782	50.0061
2	16.700	MM	0.4557	3425.99902	125.29250	49.9939

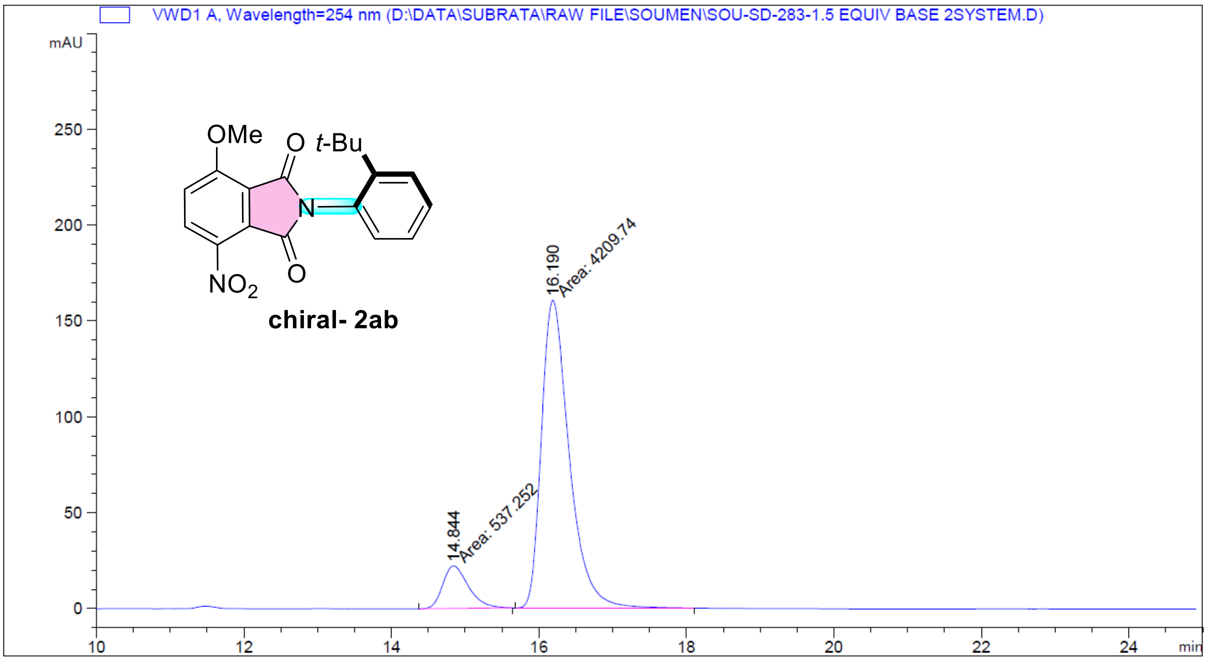

Peak #	RetTime [min]	Type	Width [min]	Area [mAU*s]	Height [mAU]	Area %
1	14.844	MM	0.4041	537.25153	22.15563	11.3177
2	16.190	MM	0.4372	4209.73633	160.47209	88.6823

Sample Info : CHIRALPAK IA , 20% IPA-HEXANE, 1.0 mL/min, 254 nm

(*P*)-2-(2-(*tert*-Butyl)phenyl)-1*H*-benzo[*e*]isoindole-1,3(2*H*)-dione (2ac)

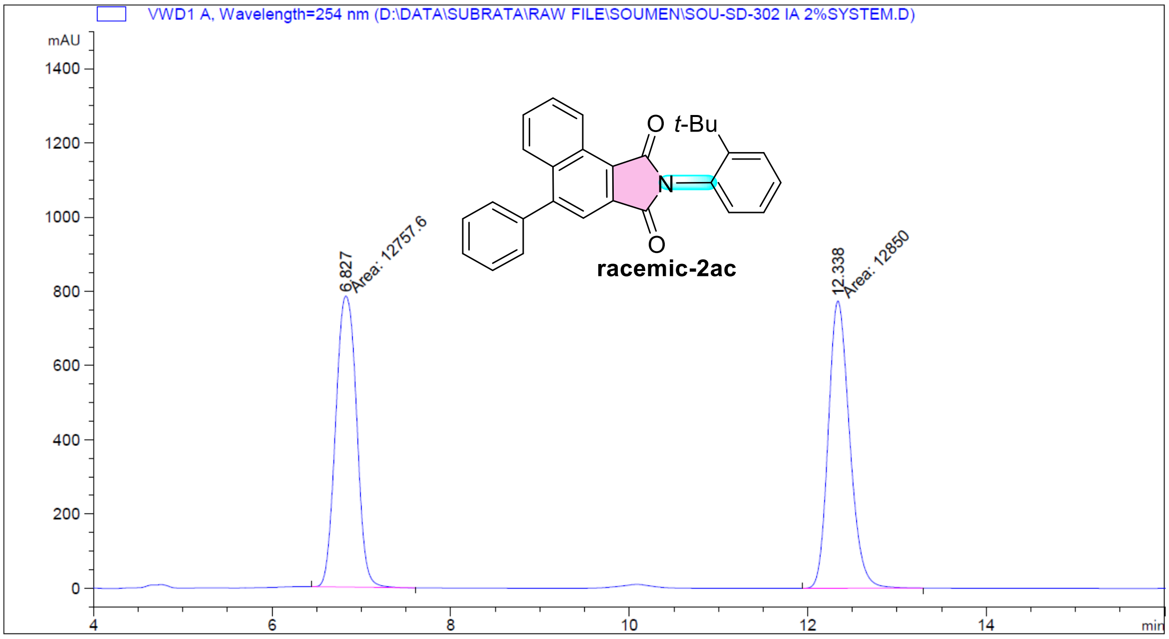

Peak #	RetTime [min]	Type	Width [min]	Area [mAU*s]	Height [mAU]	Area %
1	6.827	MM	0.2711	1.27576e4	784.27466	49.8196
2	12.338	MM	0.2766	1.28500e4	774.31384	50.1804

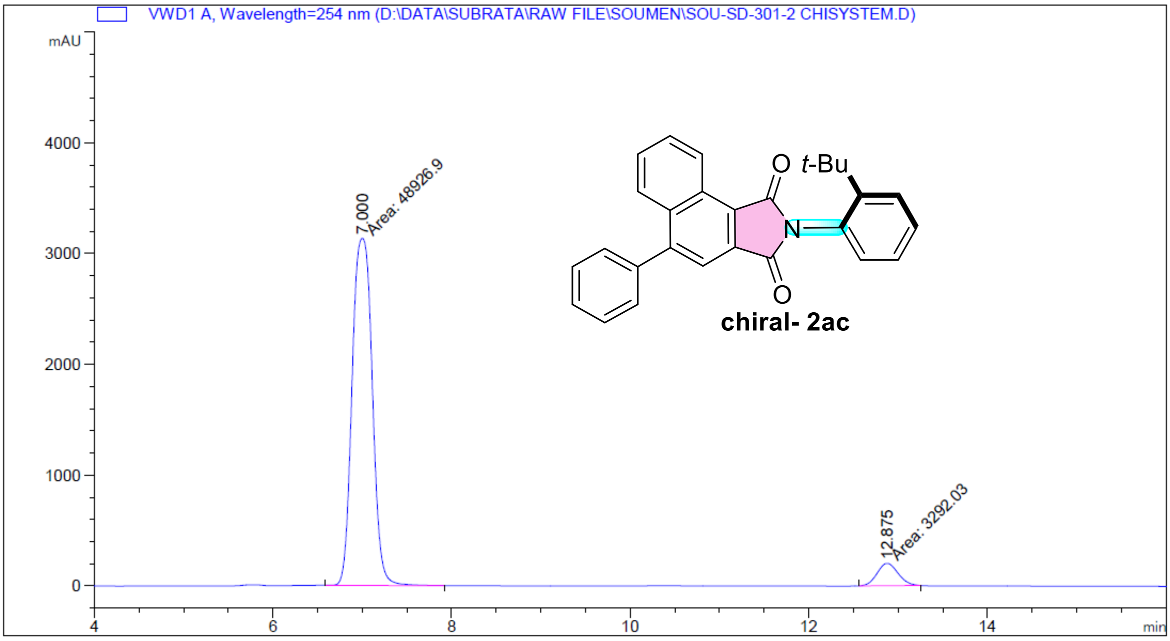

Peak #	RetTime [min]	Type	Width [min]	Area [mAU*s]	Height [mAU]	Area %
1	7.000	MM	0.2603	4.89269e4	3133.05200	93.6957
2	12.875	MM	0.2715	3292.02783	202.12033	6.3043

Sample Info : CHIRALPAK IA, 2% IPA-HEXANE, 1.0 mL/min, 254 nm

(*P*)-6-(2-(*tert*-Butyl)phenyl)-5*H*-pyrrolo[3,4-b]pyridine-5,7(6*H*)-dione (2ad)

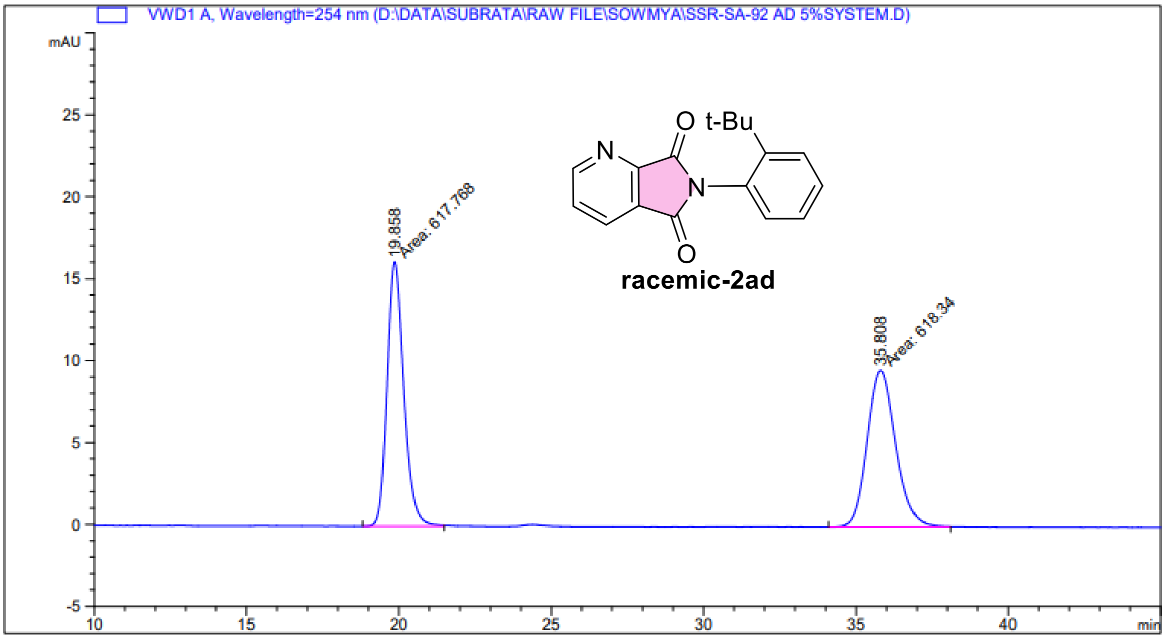

Peak #	RetTime [min]	Type	Width [min]	Area [mAU*s]	Height [mAU]	Area %
1	19.858	MM	0.6381	617.76752	16.13573	49.9769
2	35.808	MM	1.0787	618.33978	9.55355	50.0231

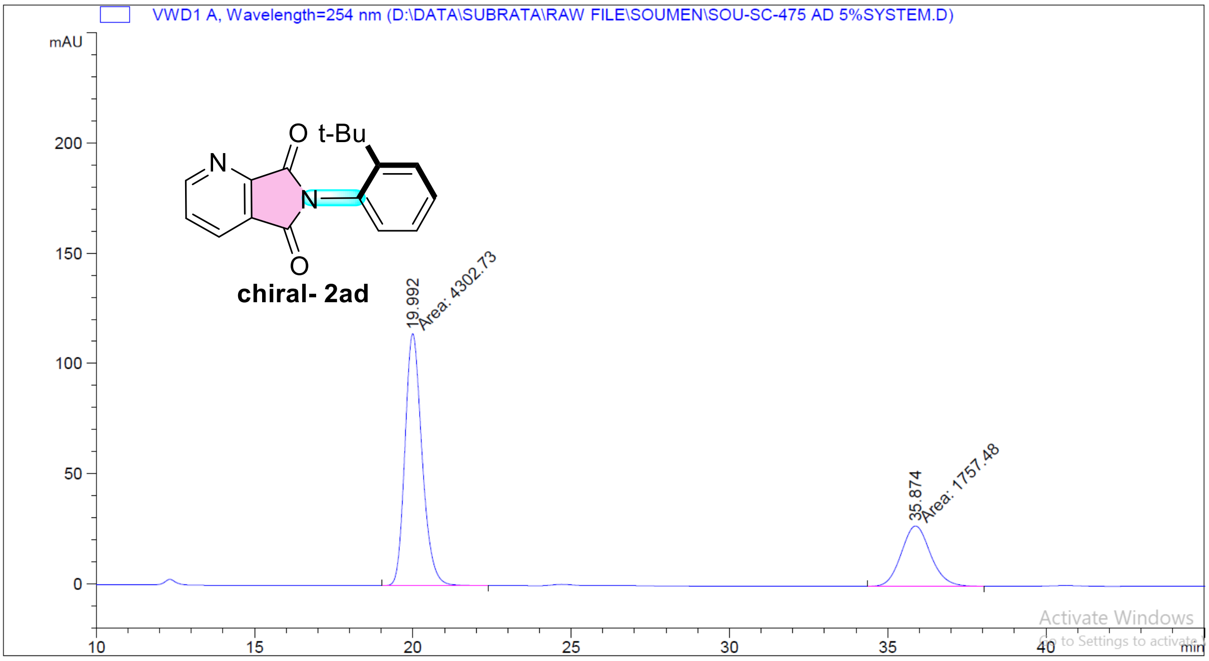

Peak #	RetTime [min]	Type	Width [min]	Area [mAU*s]	Height [mAU]	Area %
1	19.992	MM	0.6273	4302.72900	114.31154	70.9997
2	35.874	MM	1.0730	1757.47925	27.29798	29.0003

Sample Info : CHIRALPAK AD, 5% IPA:HEXANE, 1.0 mL/min, 254 nm

(*P*)-2-(2-(*tert*-Butyl)phenyl)-4-methylisoindoline-1,3-dione (9a)

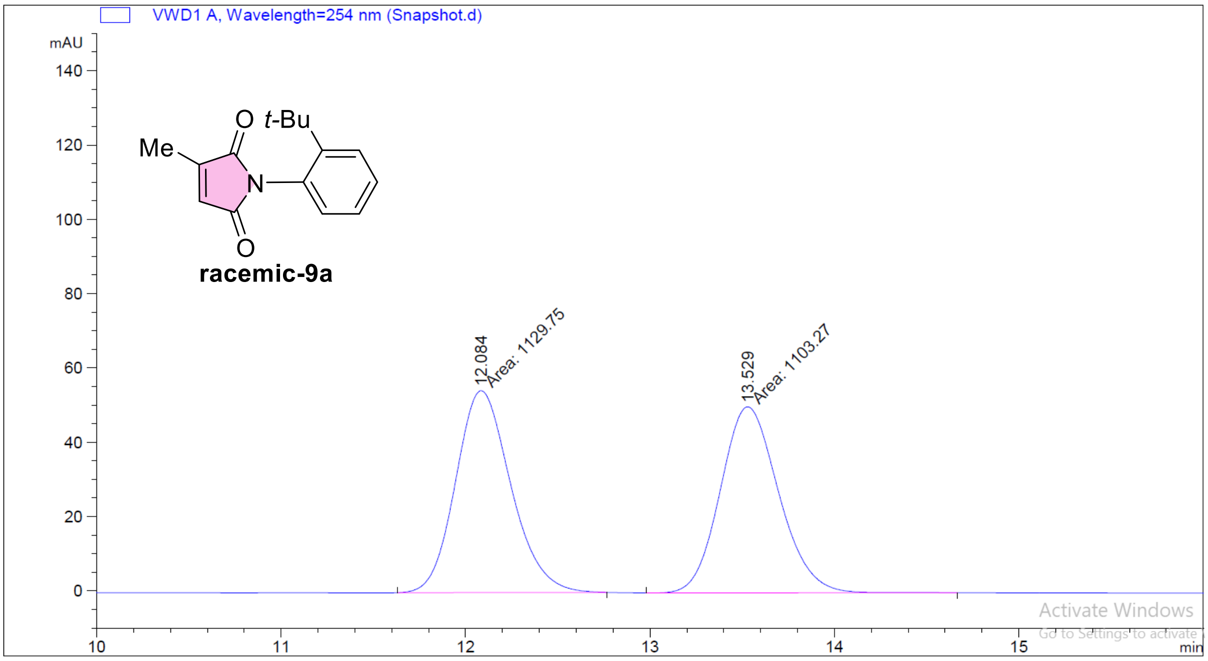

Peak #	RetTime [min]	Type	Width [min]	Area [mAU*s]	Height [mAU]	Area %
1	12.084	MM	0.3466	1129.74976	54.33095	50.5929
2	13.529	MM	0.3670	1103.27002	50.09952	49.4071

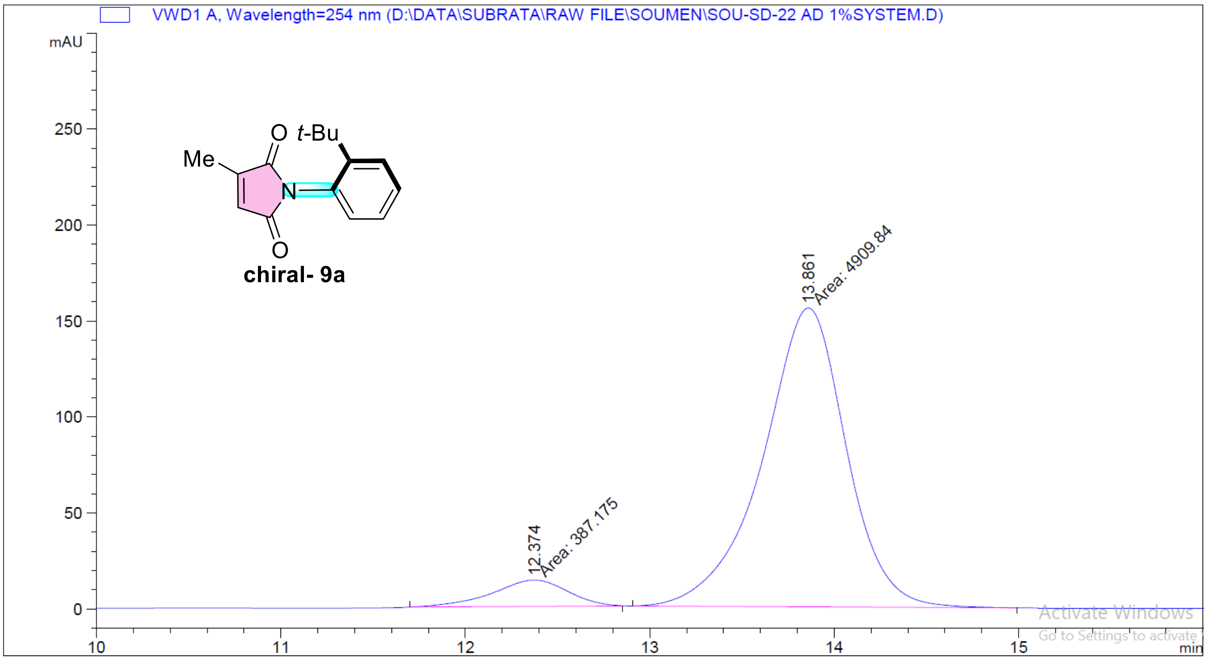

Peak #	RetTime [min]	Type	Width [min]	Area [mAU*s]	Height [mAU]	Area %
1	12.374	MM	0.4720	387.17474	13.67162	7.3093
2	13.861	MM	0.5257	4909.83643	155.66438	92.6907

Sample Info : CHIRALPAK AD, 1% IPA-HEXANE, 0.7 mL/min, 254 nm

(*P*)-1-(4-Bromo-2-(*tert*-butyl)phenyl)-3-methyl-1*H*-pyrrole-2,5-dione (9b)

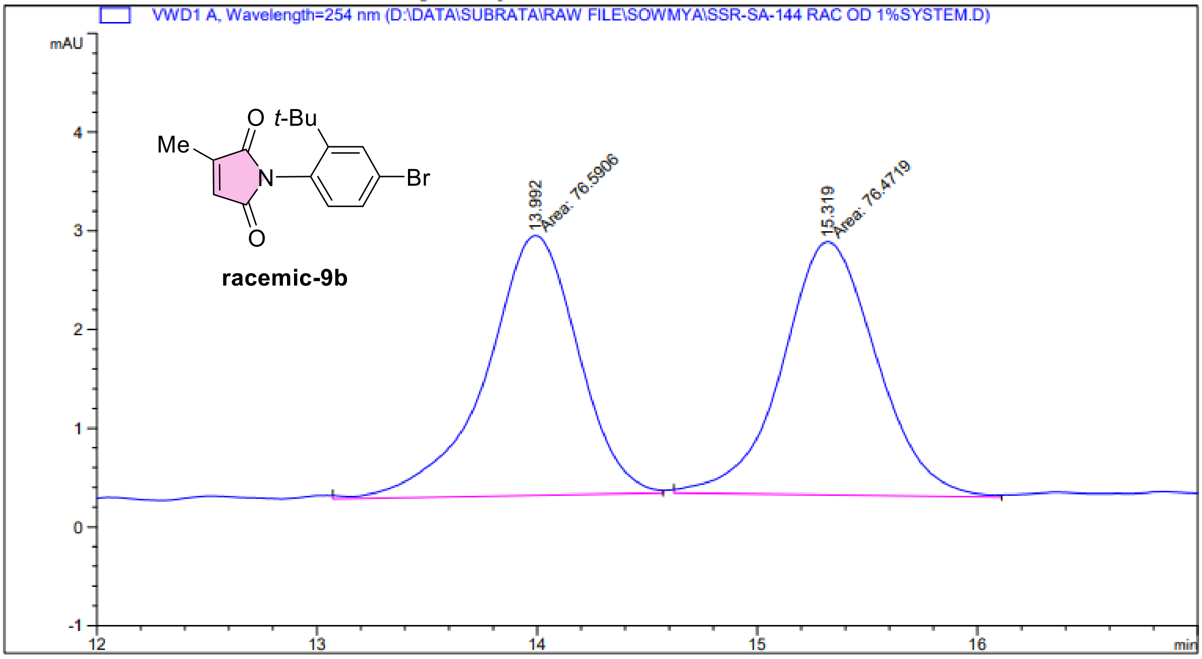

Peak #	RetTime [min]	Type	Width [min]	Area [mAU*s]	Height [mAU]	Area %
1	13.992	MM	0.4848	76.59062	2.63305	50.0388
2	15.319	MM	0.4969	76.47193	2.56475	49.9612

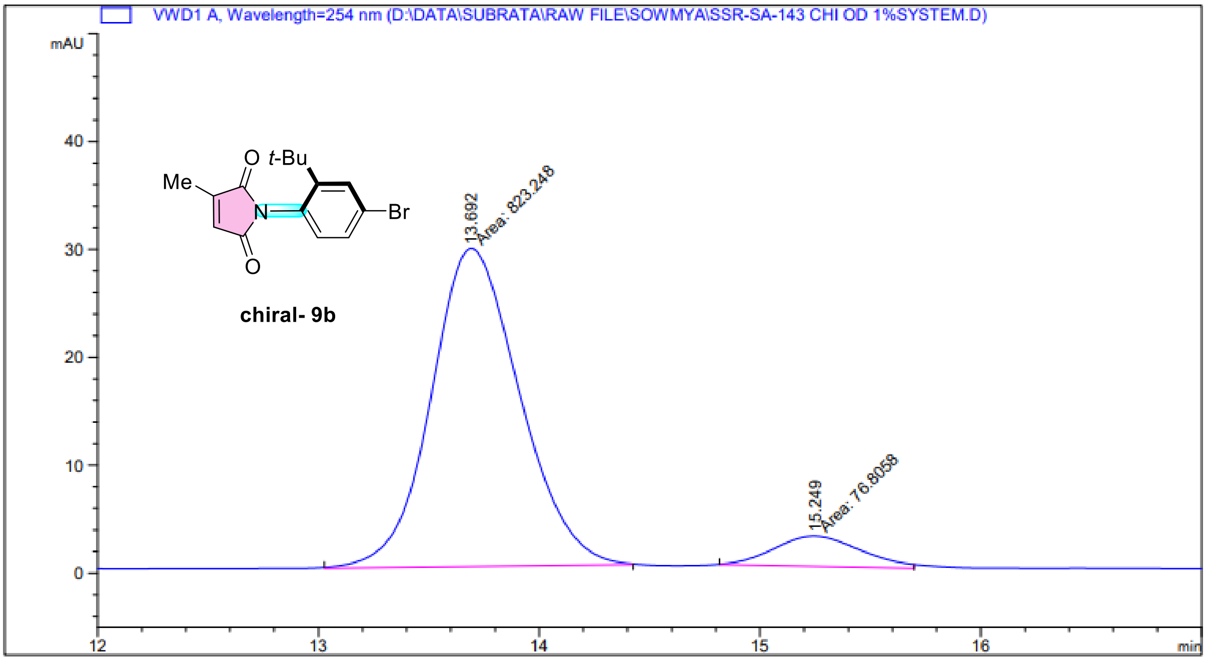

Peak #	RetTime [min]	Type	Width [min]	Area [mAU*s]	Height [mAU]	Area %
1	13.692	MM	0.4655	823.24835	29.47831	91.4665
2	15.249	MM	0.4554	76.80576	2.81118	8.5335

Sample Info : CHIRALCELL OD-H, 1 % IPA-HEXANE, 1.0 mL/min, 254 nm

(*P*)-1-(2-(*tert*-Butyl)-4-iodophenyl)-3-methyl-1*H*-pyrrole-2,5-dione (9c)

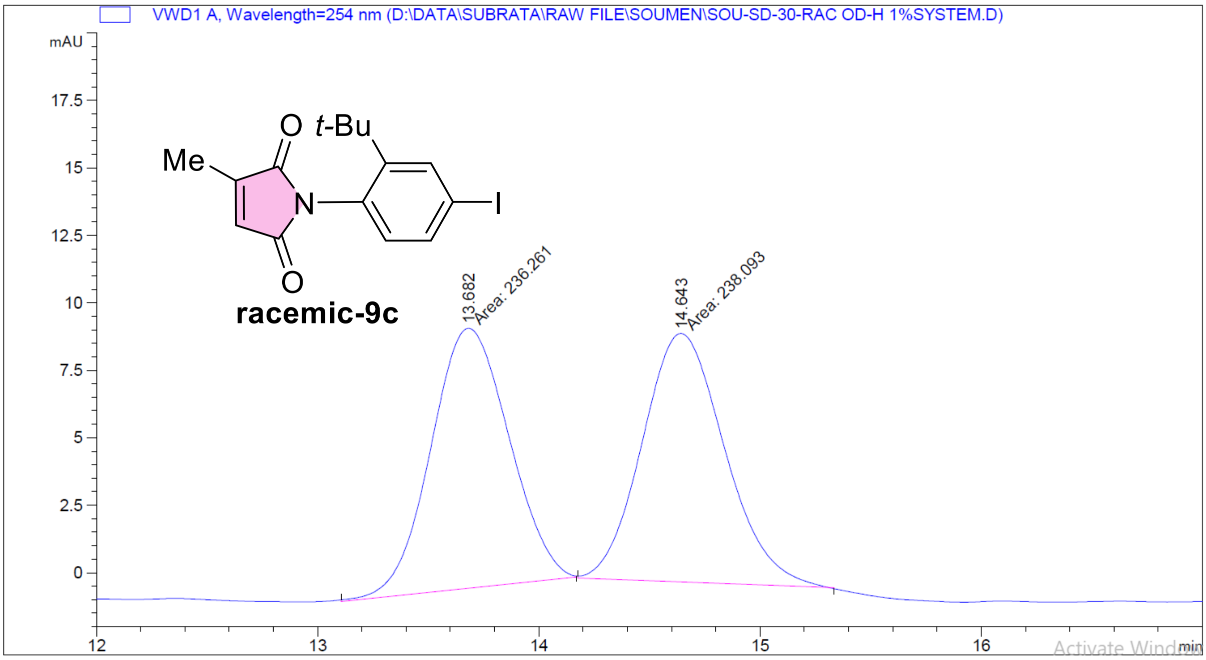

Peak #	RetTime [min]	Type	Width [min]	Area [mAU*s]	Height [mAU]	Area %
1	13.682	MM	0.4088	236.26070	9.63264	49.8069
2	14.643	MM	0.4311	238.09280	9.20505	50.1931

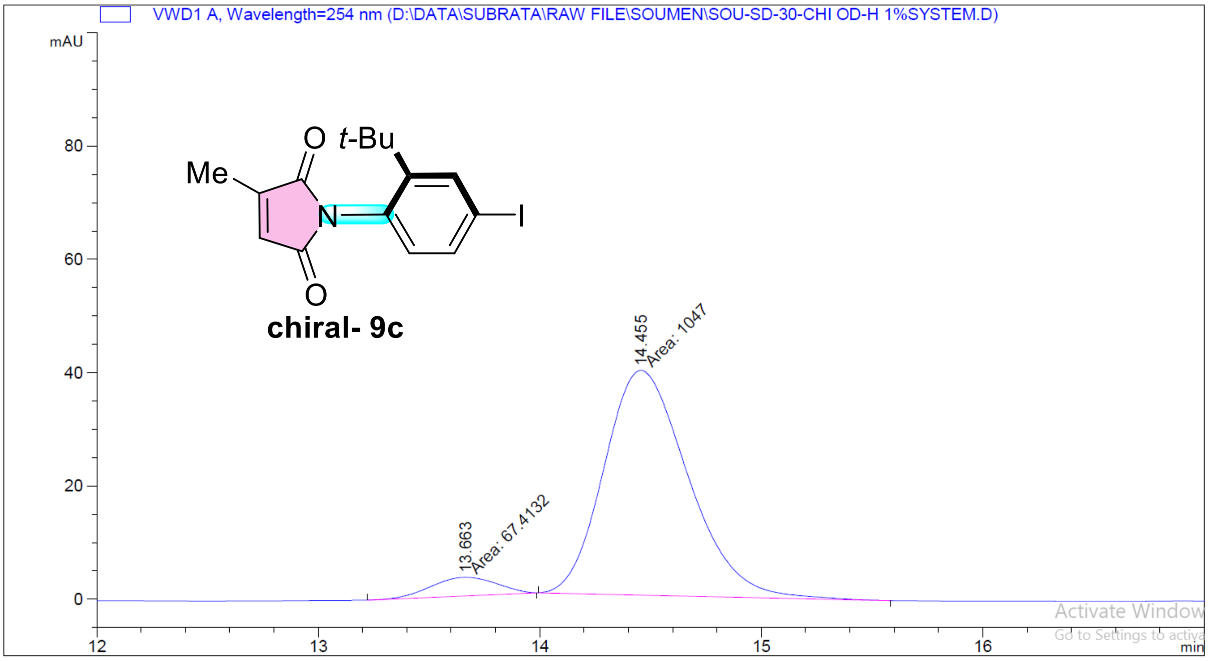

Peak #	RetTime [min]	Type	Width [min]	Area [mAU*s]	Height [mAU]	Area %
1	13.663	MM	0.3418	67.41315	3.28744	6.0492
2	14.455	MM	0.4399	1047.00330	39.66589	93.9508

Sample Info : CHIRALCELL OD-H, 1 % IPA-HEXANE, 1.0 mL/min, 254 nm

(*P*)-Ethyl-3-(*tert*-butyl)-4-(3-methyl-2,5-dioxo-2,5-dihydro-1*H*-pyrrol-1-yl)benzoate (9d)

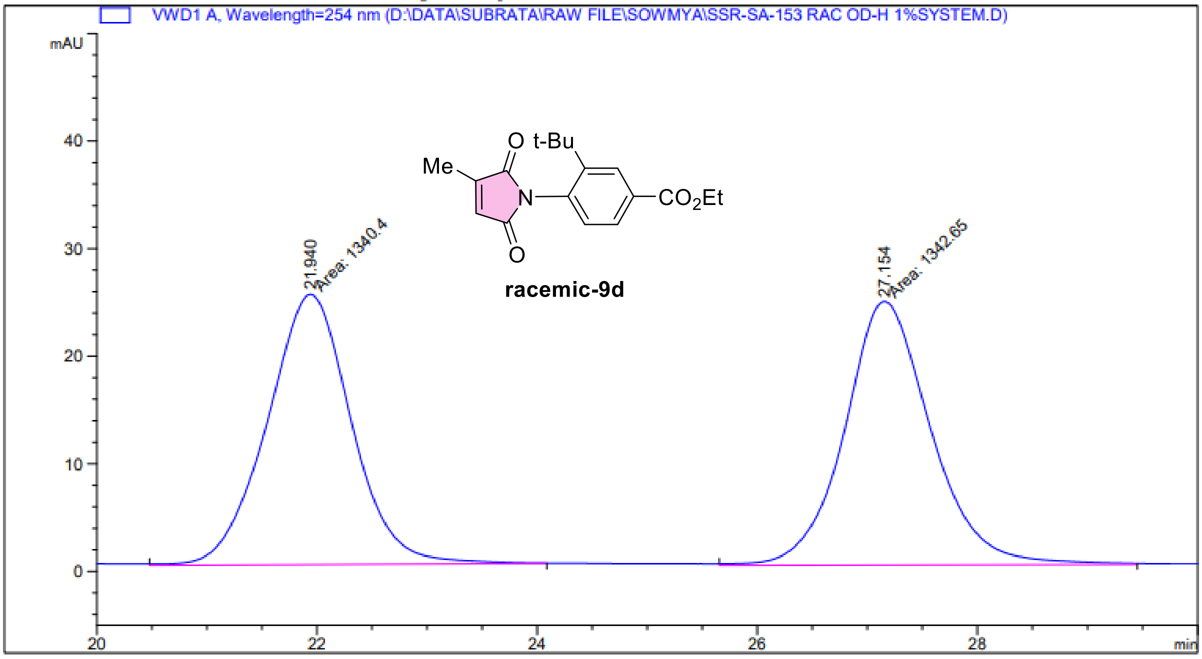

Peak #	RetTime [min]	Type	Width [min]	Area [mAU*s]	Height [mAU]	Area %
1	21.940	MM	0.8887	1340.40454	25.13757	49.9581
2	27.154	MM	0.9124	1342.65356	24.52471	50.0419

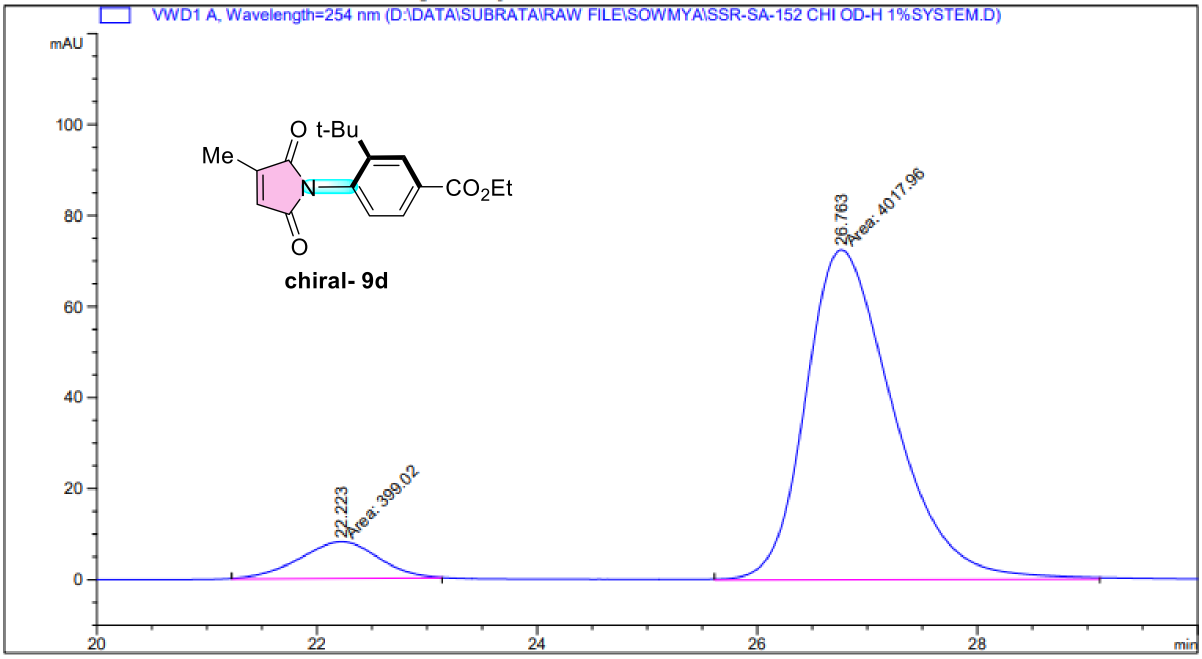

Peak #	RetTime [min]	Type	Width [min]	Area [mAU*s]	Height [mAU]	Area %
1	22.223	MM	0.8292	399.01950	8.02058	9.0338
2	26.763	MM	0.9245	4017.95996	72.43388	90.9662

Sample Info : CHIRALCEL OD-H, 1% IPA-HEXANE, 0.5 mL/min, 254 nm

(*P*)-3-Methyl-1-(4-methyl-2-(2-phenylpropan-2-yl)phenyl)-1*H*-pyrrole-2,5-dione (9e)

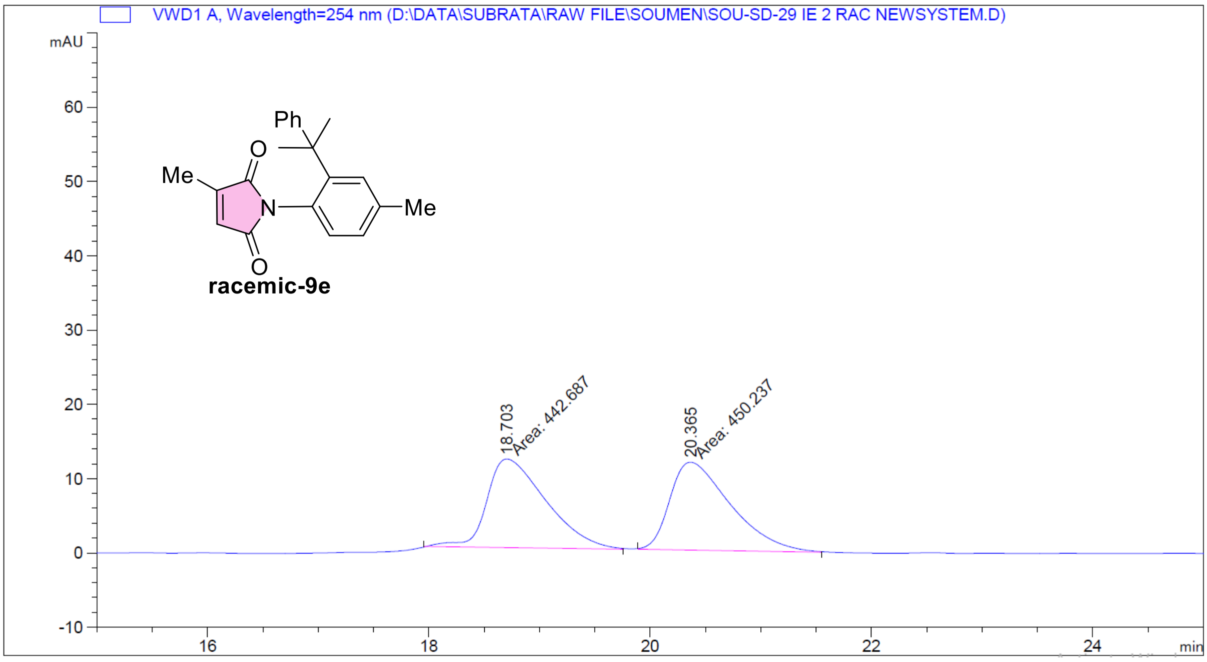

Peak #	RetTime [min]	Type	Width [min]	Area [mAU*s]	Height [mAU]	Area %
1	18.703	MM	0.6183	442.68732	11.93288	49.5772
2	20.365	MM	0.6345	450.23749	11.82659	50.4228

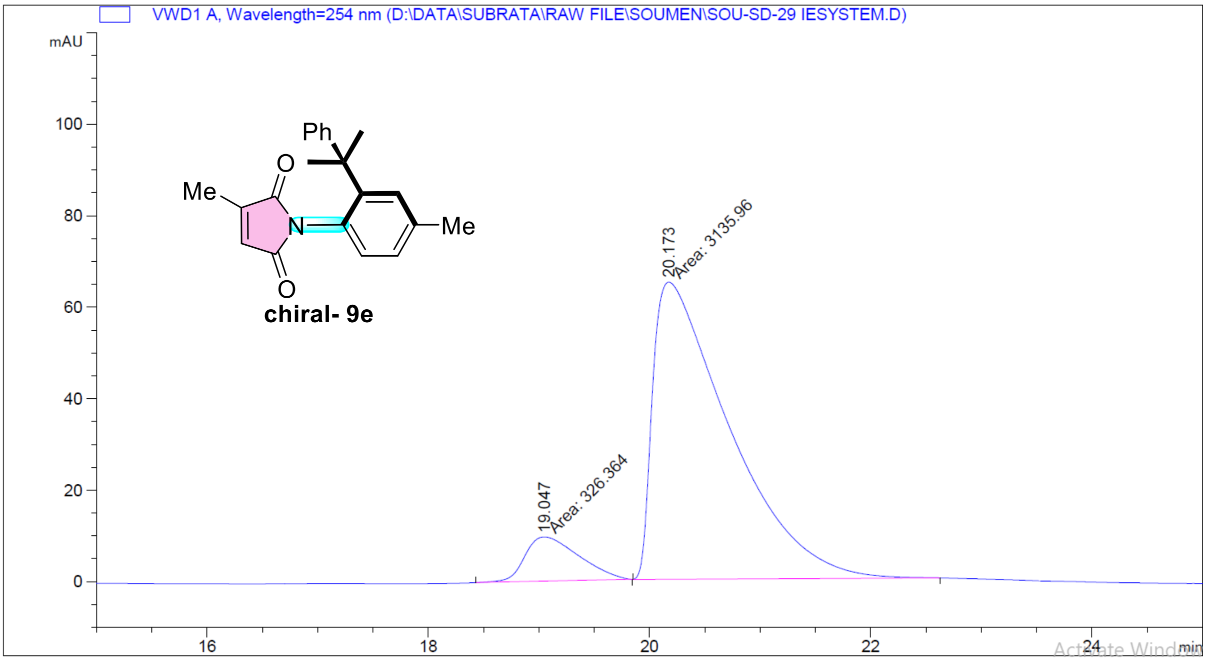

Peak #	RetTime [min]	Type	Width [min]	Area [mAU*s]	Height [mAU]	Area %
1	19.047	MM	0.5620	326.36429	9.67838	9.4262
2	20.173	MM	0.8045	3135.96265	64.96745	90.5738

Sample Info : CHIRALPAK IE, 1% IPA-HEXANE, 1.0 mL/min, 254 nm

(*P*)-3-Bromo-1-(2-(*tert*-butyl)phenyl)-4-methyl-1*H*-pyrrole-2,5-dione (9f)

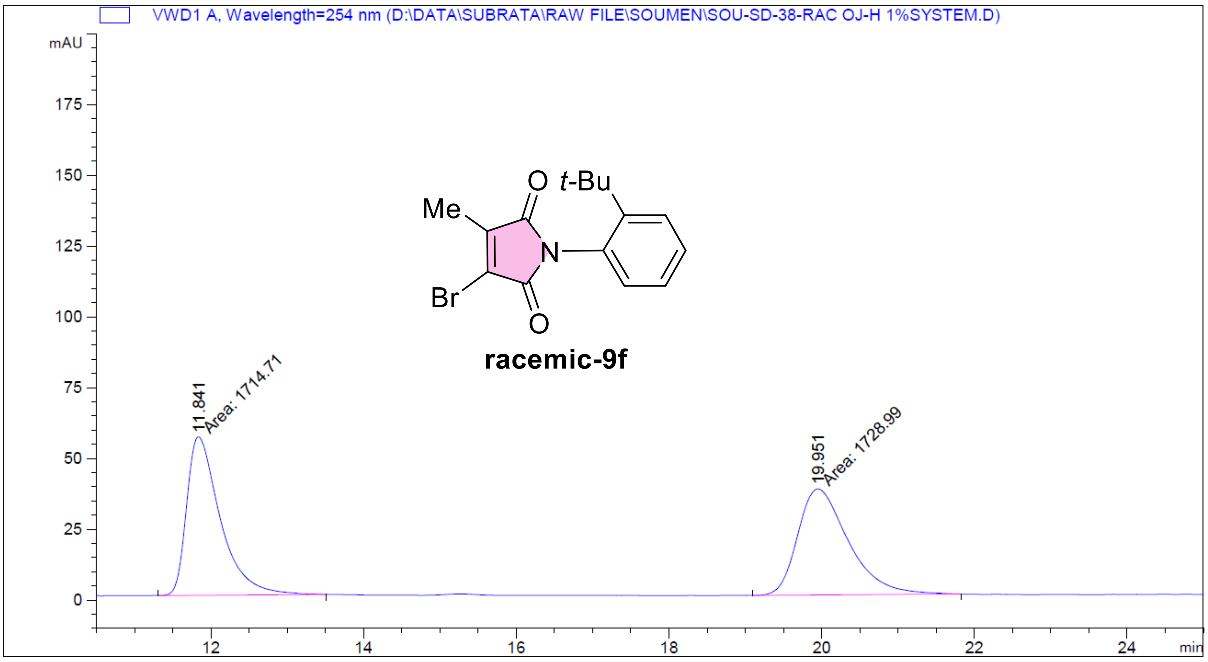

Peak #	RetTime [min]	Type	Width [min]	Area [mAU*s]	Height [mAU]	Area %
1	11.841	MM	0.5108	1714.71497	55.94942	49.7927
2	19.951	MM	0.7699	1728.99023	37.43118	50.2073

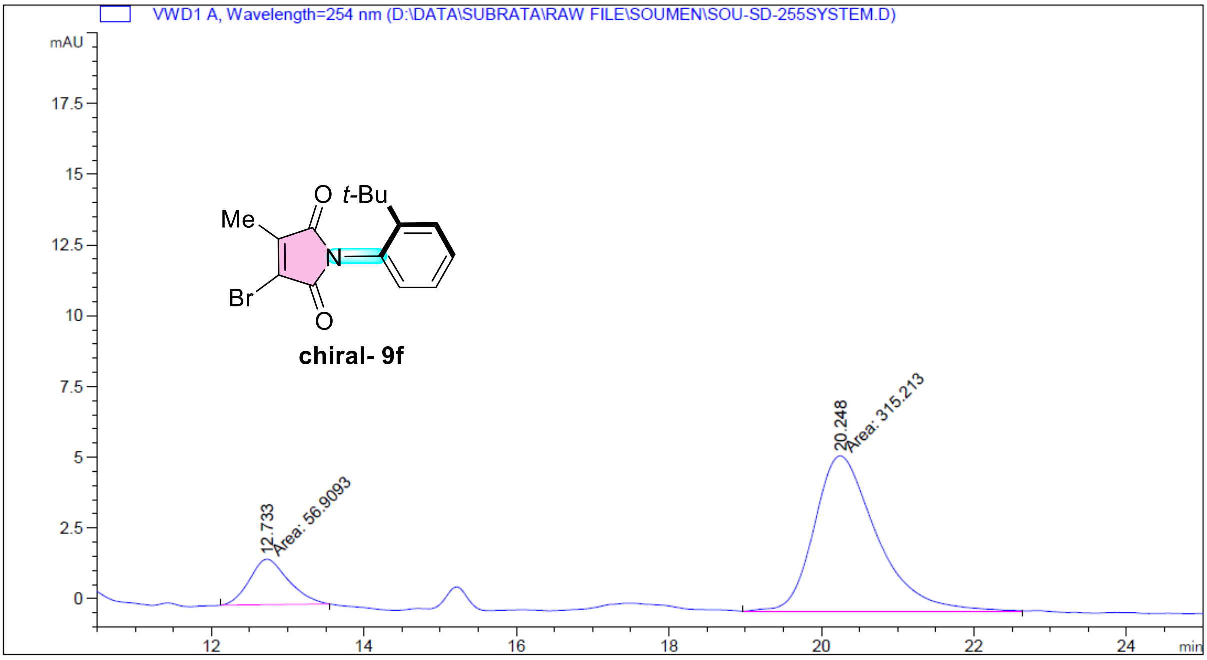

Peak #	RetTime [min]	Type	Width [min]	Area [mAU*s]	Height [mAU]	Area %
1	12.733	MM	0.5915	56.90931	1.60366	15.2932
2	20.248	MM	0.9551	315.21289	5.50044	84.7068

Sample Info : CHIRALCELL OJ-H, 1% IPA-HEXANE, 1.0 mL/min, 254 nm

(*P*)-4-(Bromomethyl)-2-(2-(*tert*-butyl)phenyl)isoindoline-1,3-dione (10a)

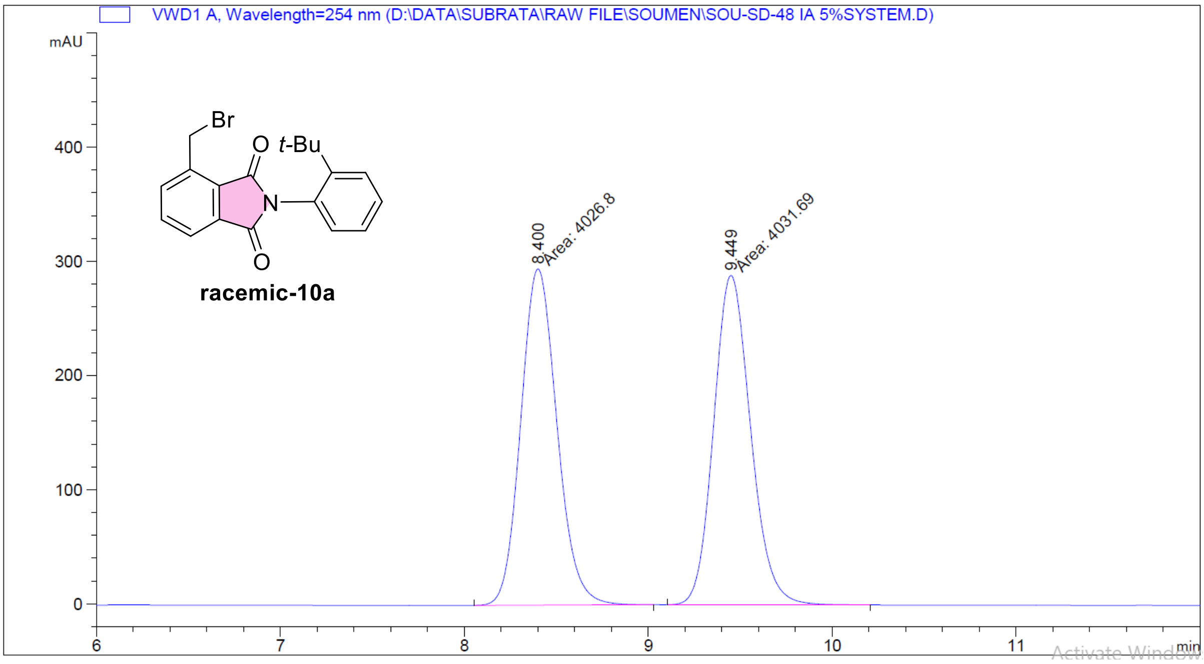

Peak #	RetTime [min]	Type	Width [min]	Area [mAU*s]	Height [mAU]	Area %
1	8.400	MM	0.2279	4026.79565	294.51895	49.9696
2	9.449	MM	0.2329	4031.69189	288.49084	50.0304

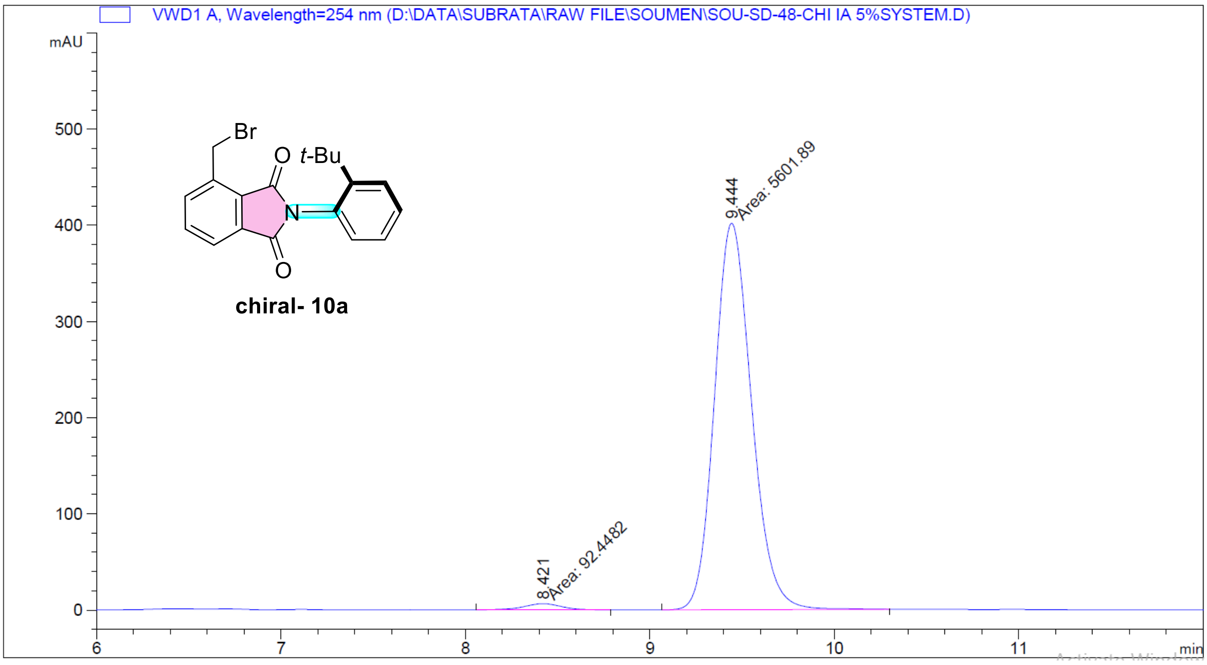

Peak #	RetTime [min]	Type	Width [min]	Area [mAU*s]	Height [mAU]	Area %
1	8.421	MM	0.2381	92.44825	6.47175	1.6235
2	9.444	MM	0.2323	5601.88623	401.84967	98.3765

Sample Info : CHIRALPAK IA 5% IPA-HEXANE, 1.0 mL/min, 254 nm

(*P*)-2-(2-(*tert*-Butyl)phenyl)-1,3-dioxoisoindoline-4-carbaldehyde (11a)

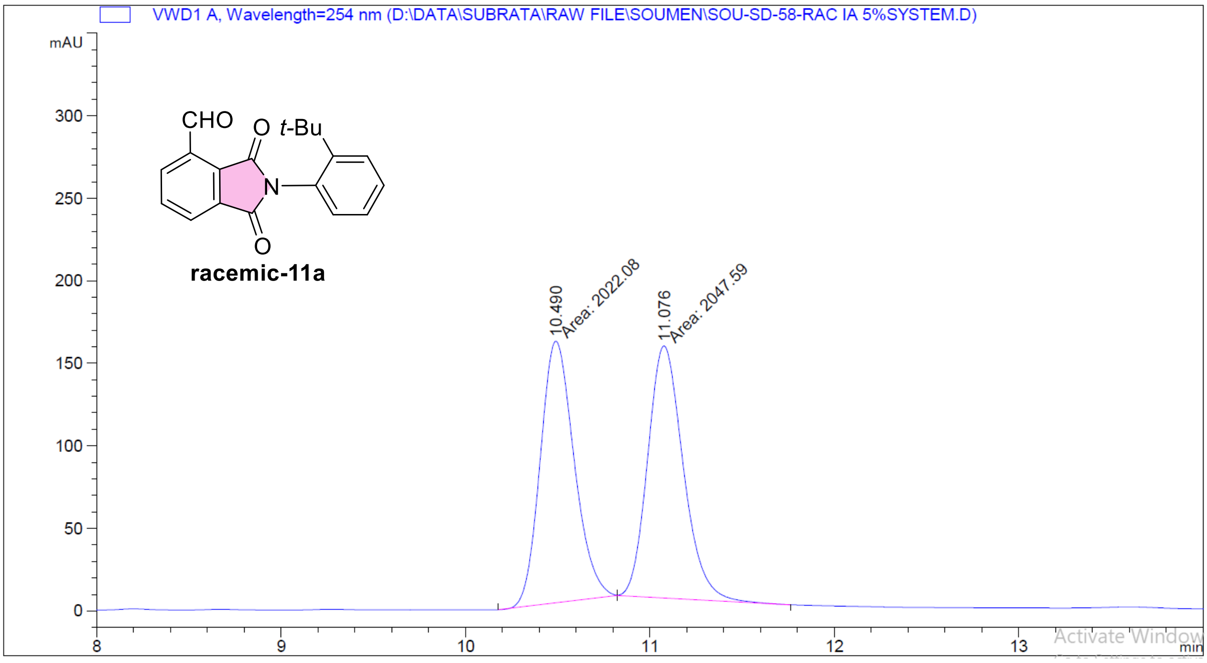

Peak #	RetTime [min]	Type	Width [min]	Area [mAU*s]	Height [mAU]	Area %
1	10.490	MM	0.2126	2022.08105	158.49521	49.6866
2	11.076	MM	0.2235	2047.58984	152.68442	50.3134

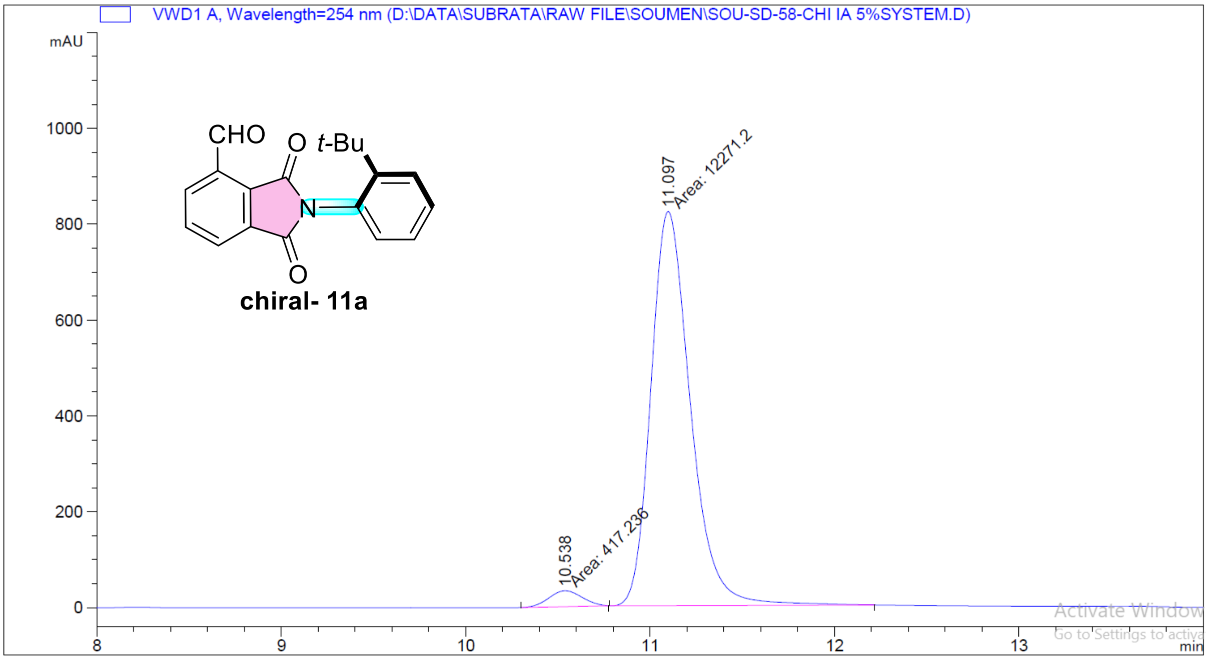

Peak #	RetTime [min]	Type	Width [min]	Area [mAU*s]	Height [mAU]	Area %
1	10.538	MM	0.2084	417.23563	33.36221	3.2883
2	11.097	MM	0.2486	1.22712e4	822.79041	96.7117

Sample Info : CHIRALPAK IA, 5% IPA-HEXANE, 1.0 mL/min, 254 nm

(*P*)-2-(2-(*tert*-Butyl)phenyl)-4-vinylisoindoline-1,3-dione (12a)

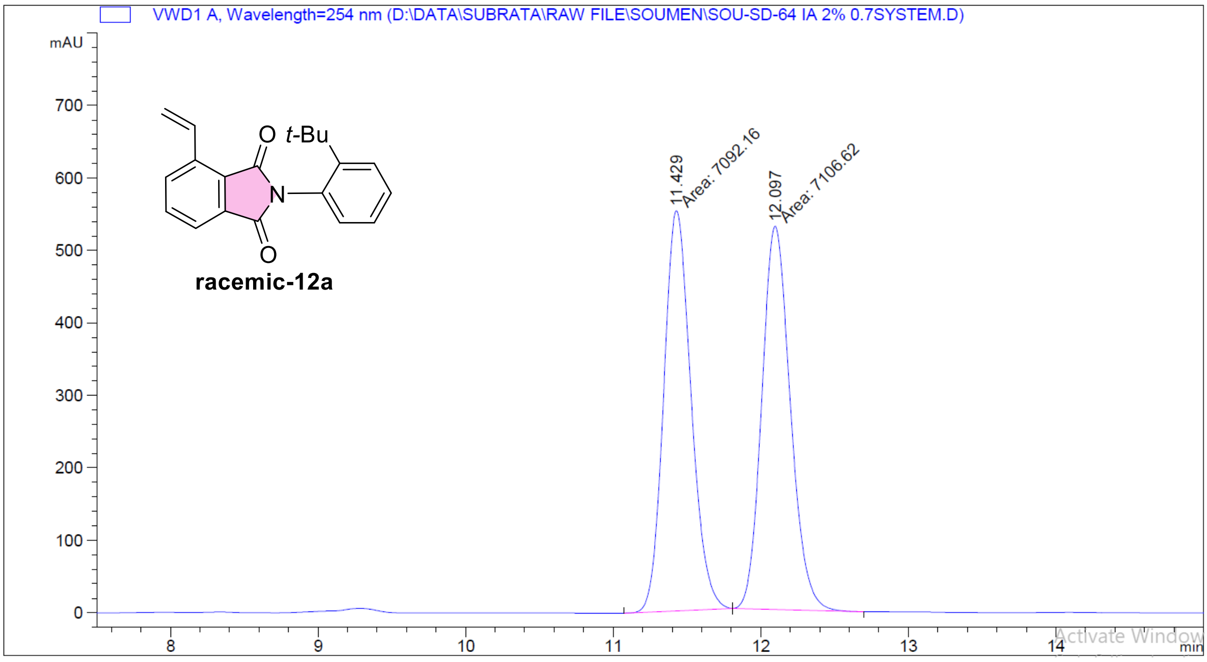

Peak #	RetTime [min]	Type	Width [min]	Area [mAU*s]	Height [mAU]	Area %
1	11.429	MM	0.2140	7092.16113	552.23627	49.9491
2	12.097	MM	0.2239	7106.62012	529.09509	50.0509

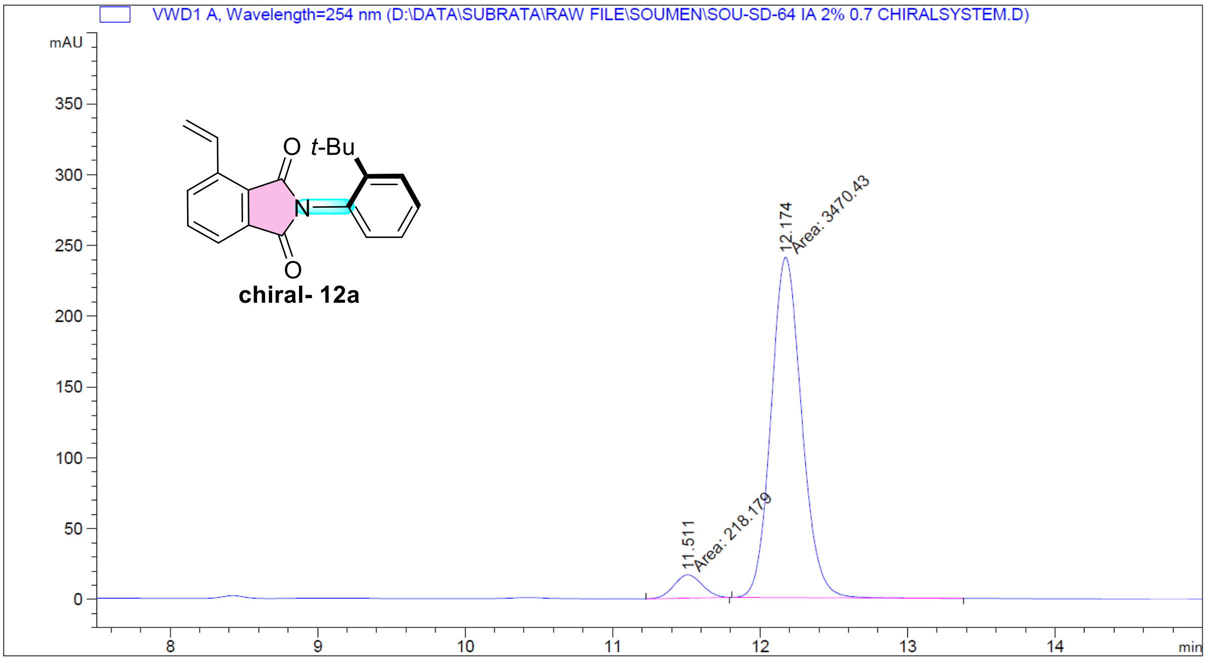

Peak #	RetTime [min]	Type	Width [min]	Area [mAU*s]	Height [mAU]	Area %
1	11.511	MM	0.2211	218.17912	16.44950	5.9149
2	12.174	MM	0.2406	3470.42847	240.42242	94.0851

Sample Info : CHIRAlPAK IA, 2% IPA-HEXANE, 0.7 mL/min, 254 nm

(*P*)-4-(Azidomethyl)-2-(2-(*tert*-butyl)phenyl)isoindoline-1,3-dione (13a)

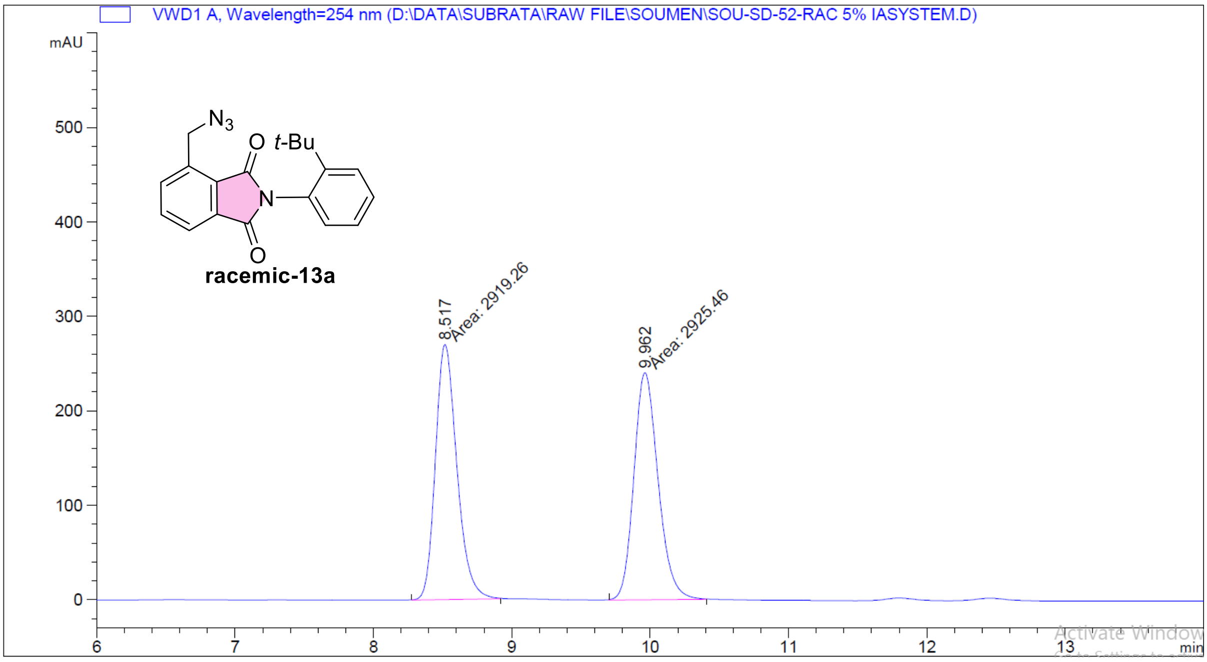

Peak #	RetTime [min]	Type	Width [min]	Area [mAU*s]	Height [mAU]	Area %
1	8.517	MM	0.1802	2919.26025	270.04202	49.9470
2	9.962	MM	0.2028	2925.45654	240.41580	50.0530

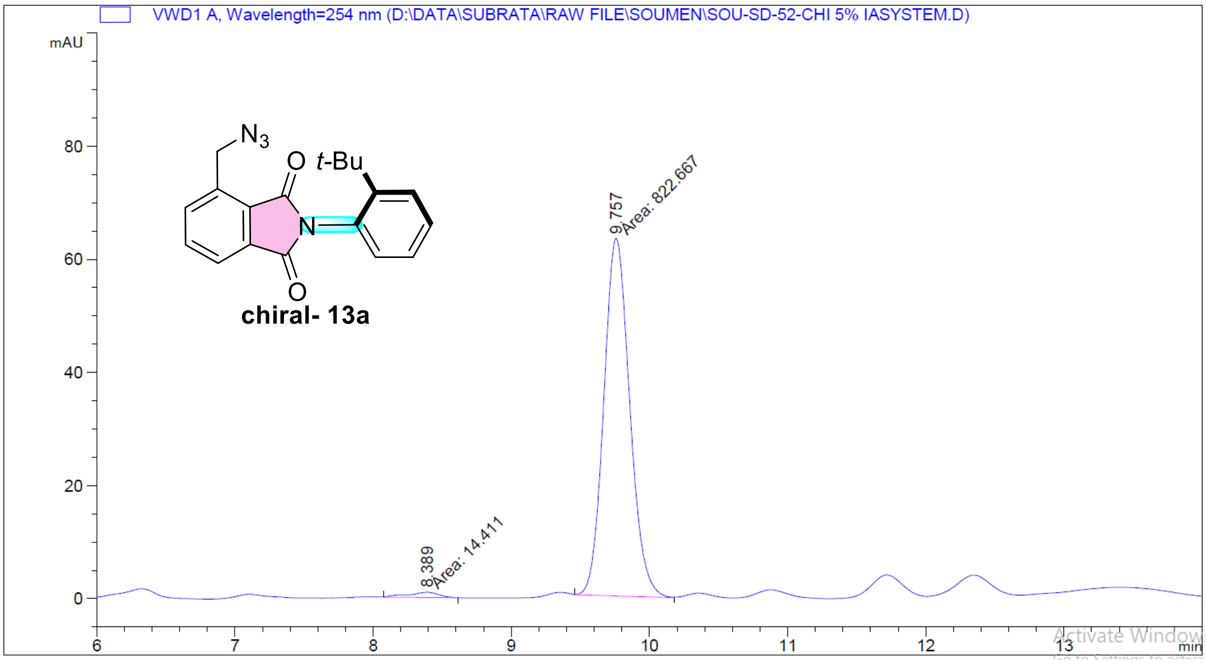

Peak #	RetTime [min]	Type	Width [min]	Area [mAU*s]	Height [mAU]	Area %
1	8.389	MM	0.2585	14.41102	9.29058e-1	1.7216
2	9.757	MM	0.2169	822.66742	63.21178	98.2784

Sample Info : CHIRALPAK IA, 5% IPA-HEXANE,1.0 mL/min, 254 nm

(*P*)-4-((1*H*-Benzo[*d*][1,2,3]triazol-1-yl)methyl)-2-(2-(*tert*-butyl)phenyl)isoindoline-1,3-dione (14a)

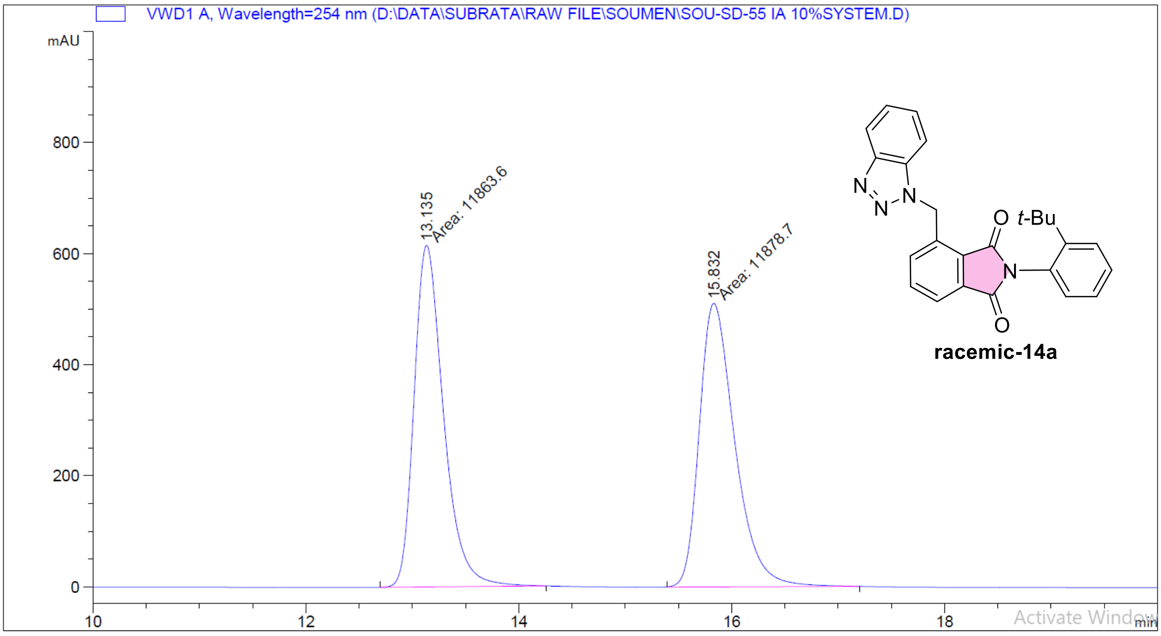

Peak #	RetTime [min]	Type	Width [min]	Area [mAU*s]	Height [mAU]	Area %
1	13.135	MM	0.3214	1.18636e4	615.26337	49.9683
2	15.832	MM	0.3875	1.18787e4	510.91086	50.0317

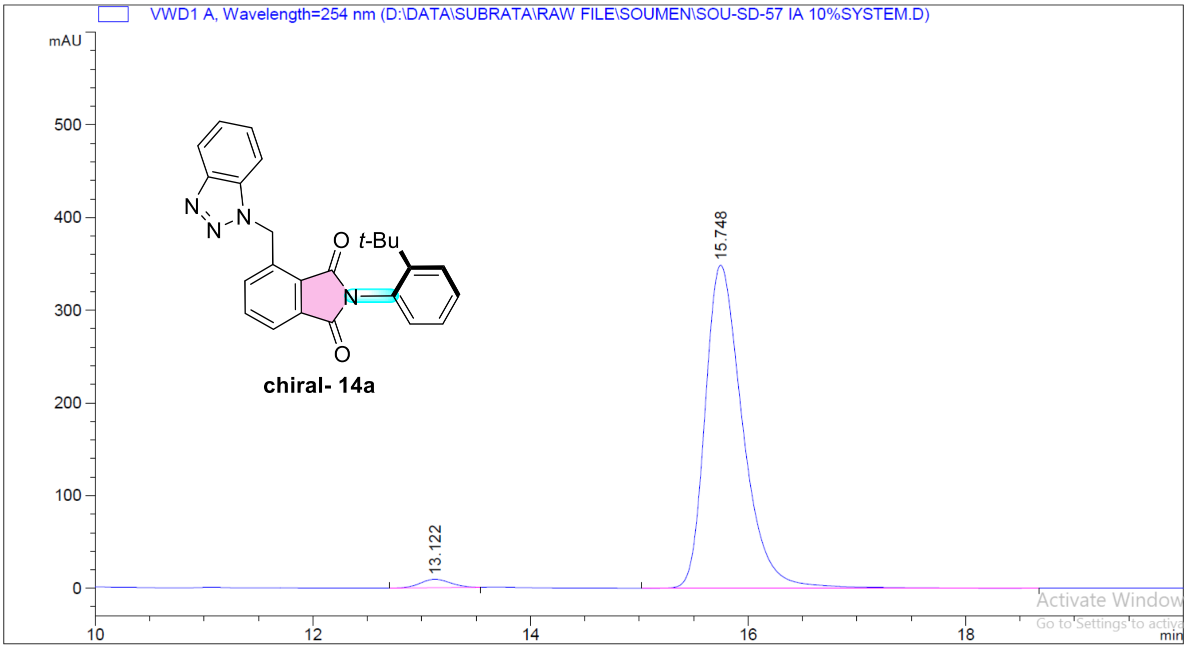

Peak #	RetTime [min]	Type	Width [min]	Area [mAU*s]	Height [mAU]	Area %
1	13.122	BB	0.2971	172.92986	9.04202	2.0265
2	15.748	BB	0.3658	8360.70020	348.60822	97.9735

Sample Info : CHIRALPAK IA, 10% IPA-HEXANE, 1.0 mL/min, 254 nm

(*P*)-3-Bromo-1-(2-(*tert*-butyl)phenyl)-4-methyl-1*H*-pyrrole-2,5-dione (9f)

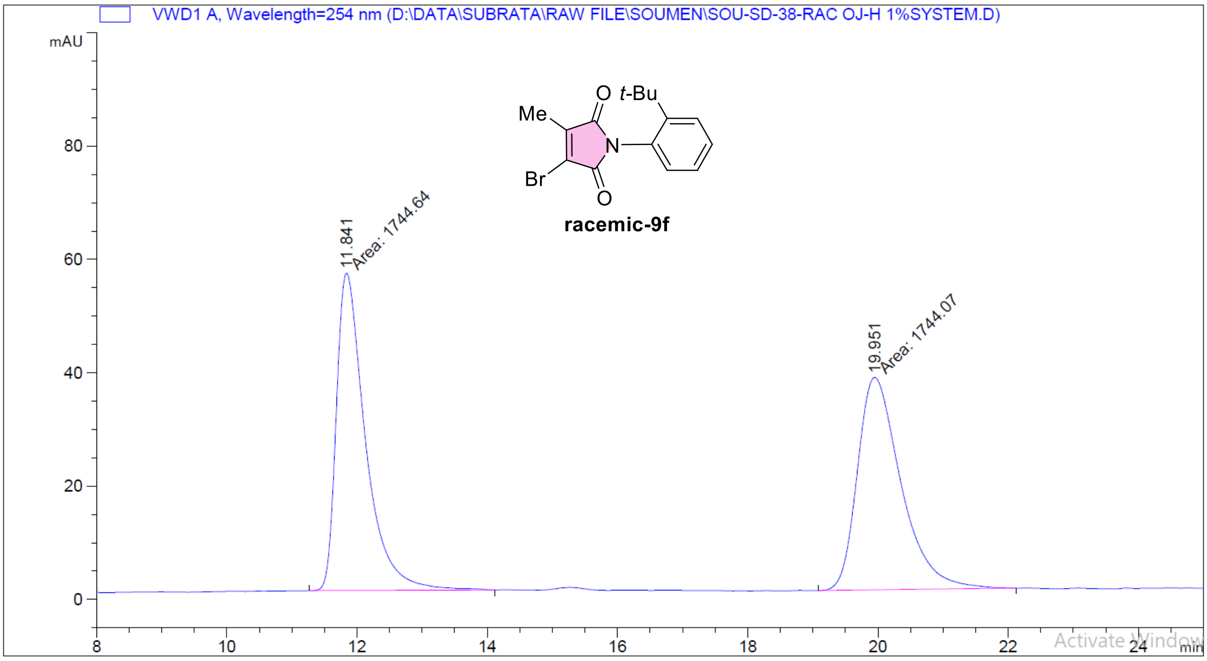

Peak #	RetTime [min]	Type	Width [min]	Area [mAU*s]	Height [mAU]	Area %
1	11.841	MM	0.5190	1744.64185	56.02587	50.0082
2	19.951	MM	0.7752	1744.06677	37.49576	49.9918

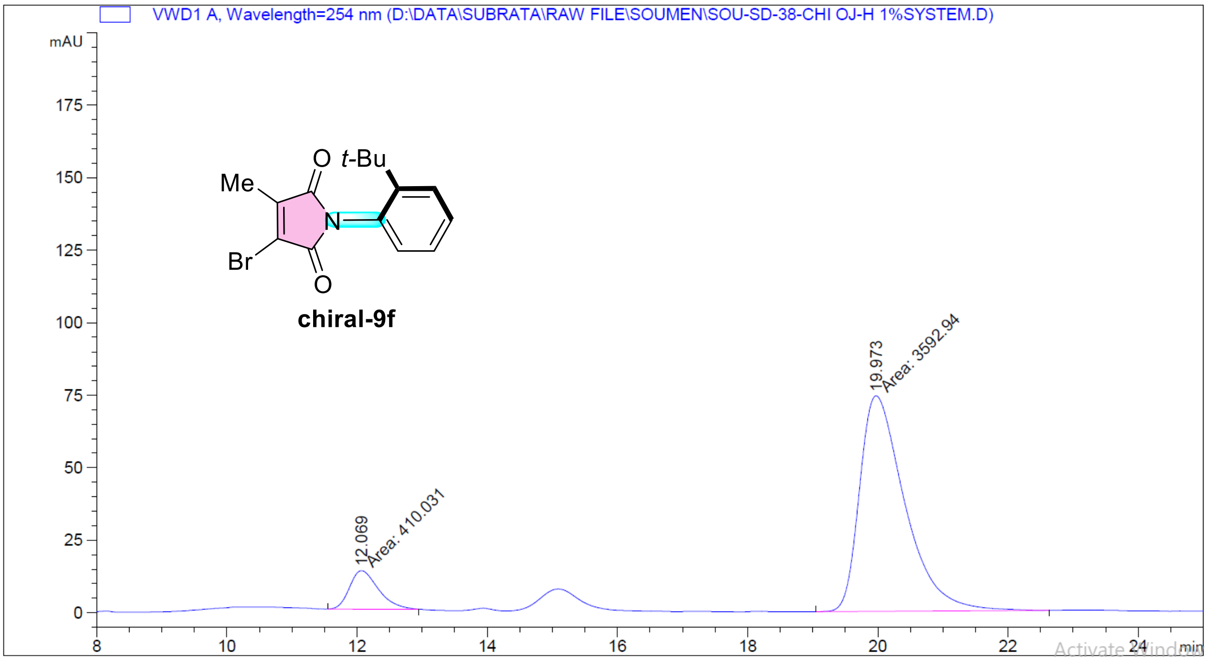

Peak #	RetTime [min]	Type	Width [min]	Area [mAU*s]	Height [mAU]	Area %
1	12.069	MM	0.5141	410.03088	13.29158	10.2432
2	19.973	MM	0.8057	3592.94482	74.31949	89.7568

Sample Info : CHIRALCELL OJ-H, 1% IPA-HEXANE, 1.0 mL/min, 254 nm

(*P*)-1-(2-(*tert*-Butyl)phenyl)-3-(butylamino)-4-methyl-1*H*-pyrrole-2,5-dione (15a)

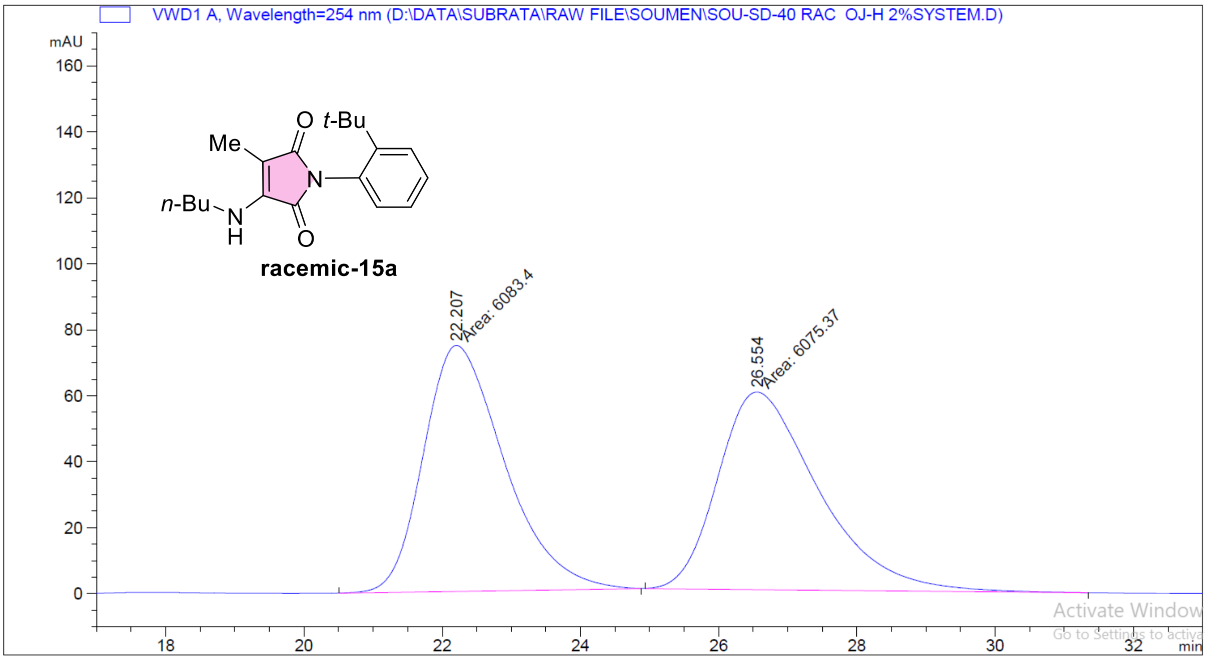

Peak #	RetTime [min]	Type	Width [min]	Area [mAU*s]	Height [mAU]	Area %
1	22.207	MM	1.3596	6083.40381	74.57314	50.0330
2	26.554	MM	1.6895	6075.36865	59.93422	49.9670

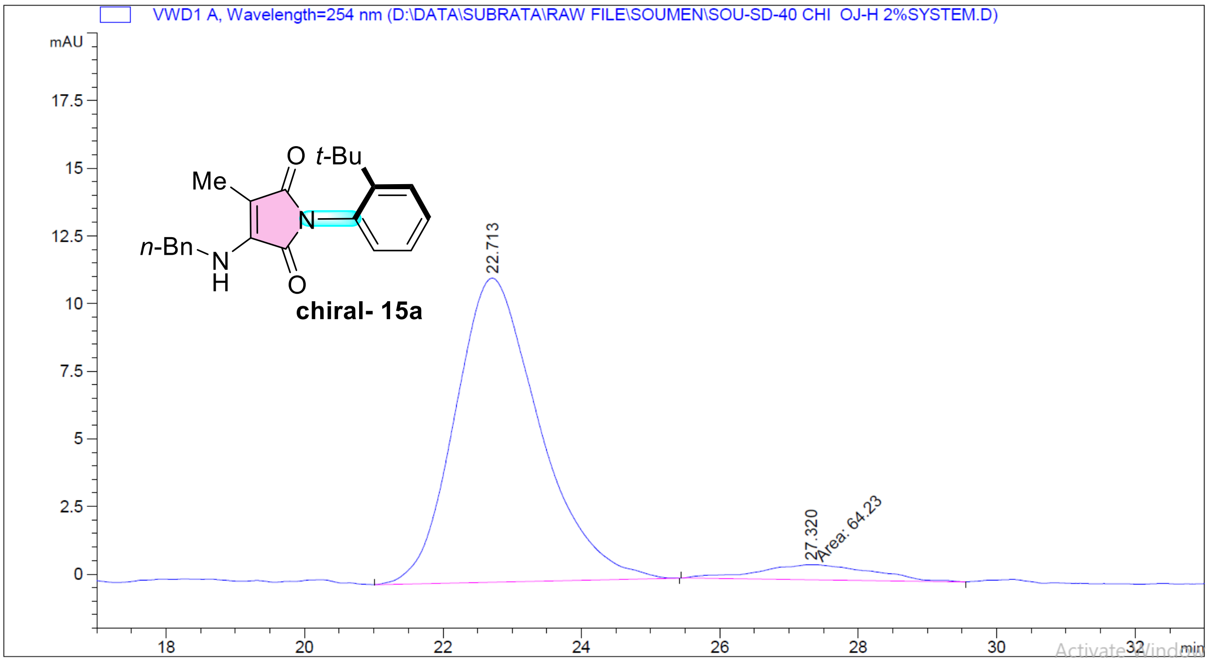

Peak #	RetTime [min]	Type	Width [min]	Area [mAU*s]	Height [mAU]	Area %
1	22.713	BB	1.1976	953.10779	11.23670	93.6865
2	27.320	MM	1.9121	64.23000	5.59849e-1	6.3135

Sample Info : CHIRALCEL OJ-H, 2% IPA-HEXANE, 1.0 mL/min, 254 nm

(*P*, *R*)-1-(2-(*tert*-Butyl)phenyl)-3-methylpyrrolidine-2,5-dione (16a)

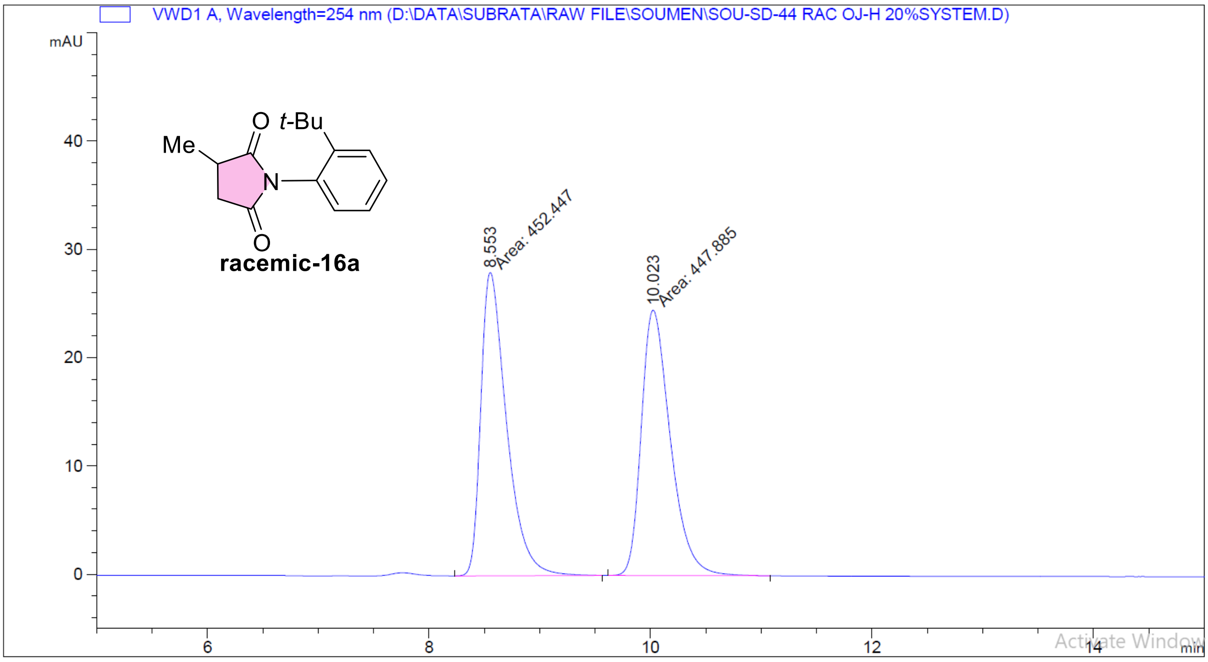

Peak #	RetTime [min]	Type	Width [min]	Area [mAU*s]	Height [mAU]	Area %
1	8.553	MM	0.2692	452.44745	28.00759	50.2534
2	10.023	MM	0.3045	447.88483	24.51153	49.7466

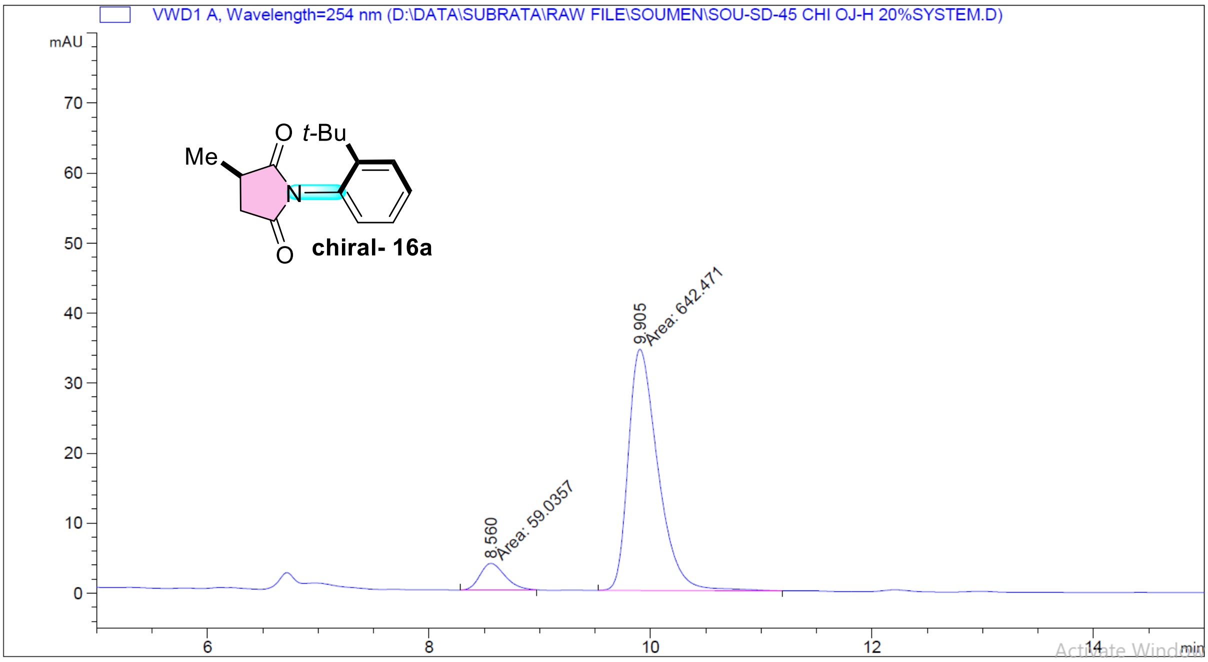

Peak #	RetTime [min]	Type	Width [min]	Area [mAU*s]	Height [mAU]	Area %
1	8.560	MM	0.2598	59.03570	3.78660	8.4156
2	9.905	MM	0.3109	642.47064	34.44249	91.5844

Sample Info : CHIRALCEL OJ-H, 20% IPA-HEXANE, 1.0 mL/min, 254 nm

4. Supplementary References

1. Struble, J. R. & Bode, J. W. Synthesis of a N -Mesityl Substituted Aminoindanol-Derived Triazolium Salt. *Org. Synth.* **87**, 362-376 (2010).
2. Matuszak, N., Muccioli, G. G., Labar, G. & Lambert, D. M. Synthesis and *in vitro* evaluation of *N*-substituted maleimide derivatives as selective monoglyceride lipase inhibitors *J. Med. Chem.* **52**, 7410-7420 (2009).
3. Guthrie, D. B. & Curran, D. P. Asymmetric Radical and Anionic Cyclizations of Axially Chiral Carbamates. *Org. Lett.* **11**, 249-251 (2009).
4. Gaussian 16, Revision C.01, Frisch, M. J.; Trucks, G. W., Schlegel, H. B., Scuseria, G. E., Robb, M. A., Cheeseman, J. R., Scalmani, G., Barone, V., Petersson, G. A., Nakatsuji, H., Li, X., Caricato, M., Marenich, A. V., Bloino, J., Janesko, B. G., Gomperts, R., Mennucci, B., Hratchian, H. P., Ortiz, J. V., Izmaylov, A. F., Sonnenberg, J. L., Williams, F. D., Lipparini, F., Egidi, F., Goings, J., Peng, B., Petrone, A., Henderson, T., Ranasinghe, D., Zakrzewski, V. G., Gao, J., Rega, N., Zheng, G., Liang, W., Hada, M., Ehara, M., Toyota, K., Fukuda, R., Hasegawa, J., Ishida, M., Nakajima, T., Honda, Y., Kitao, O., Nakai, H., Vreven, T., Throssell, K., Montgomery, J. A., Jr., Peralta, J. E., Ogliaro, F., Bearpark, M. J., Heyd, J. J., Brothers, E. N., Kudin, K. N., Staroverov, V. N., Keith, T. A., Kobayashi, R., Normand, J., Raghavachari, K., Rendell, A. P., Burant, J. C., Iyengar, S. S., Tomasi, J., Cossi, M., Millam, J. M., Klene, S78 M., Adamo, C., Cammi, R., Ochterski, J. W., Martin, R. L., Morokuma, K., Farkas, O., Foresman, J. B. & Fox, D. J. Gaussian, Inc., Wallingford CT, **2016**.
5. Hehre, W. J., Ditchfield, R. & People, J. A. Self-Consistent Molecular Orbital Methods. XII. Further Extensions of Gaussian-Type Basis Sets for Use in Molecular Orbital Studies of Organic Molecules. *J. Chem. Phys.* **56**, 2257-2261 (1972).
6. Hariharan, P. C. & People, J. A. The Influence of Polarization Functions on Molecular Orbital Hydrogenation Energies. *Theoret. Chim. Acta.* **28**, 213-222 (1973).
7. Fukui, K. The Path of Chemical Reactions - The IRC Approach. *Acc. Chem. Res.* **14**, 363-368 (1981).
8. Marenich, A. V., Cramer, C. J. & Truhlar, D. G. Universal solvation model based on solute electron density and on a continuum model of the solvent defined by the bulk dielectric constant and atomic surface tensions. *J. Phys. Chem. B* **113** (18), 6378-6396 (2009).
9. Mangani, S., Cancian, L., Leone, R., Pozzi, C., Lazzari, S., Luciani, R., Ferrari, S. & Costi, M. P. Identification of the Binding Modes of N-Phenylphthalimides Inhibiting

Bacterial Thymidylate Synthase through X-Ray Crystallography Screening. *J. Med. Chem.* **54**, 5454-5467 (2011).

10. Moorthy, J. N., Nidhi, S. & Kalyan, S. Oxidations with IBX: Benzyl Halides to Carbonyl Compounds, and the One-Pot Conversion of Olefins to 1, 2-Diketones. *Tetrahedron Lett.* **47**, 1757-1761 (2006).
11. Enders, D. & Klein, D. A novel enantioselective synthesis of α-azido ketones *Synlett* **6**, 719-720 (1999).
12. Shi, F., Waldo, J. P., Chen, Y. & Larock, R. C. Benzyne Click Chemistry: Synthesis of Benzotriazoles from Benzynes and Azides. *Org. Lett.* **10**, 2409-2412 (2008).
13. Barik, S., Das, R. C. Balanna, K. & Biju, A. T. Kinetic Resolution Approach to the Synthesis of C-N Axially Chiral *N*-aryl Aminomaleimides *via* NHC-Catalyzed [3+3] Annulation. *Org. Lett*. **24**, 5456-5461 (2022).
14. Stueckler, C., Reiter, T. C., Baudendistel, N. & Faber, K. Nicotinamide-independent asymmetric bioreduction of C C-bonds via disproportination of enones catalyzed by enoate reductases. *Tetrahedron* **66**, 663-667 (2010)
